# Supplementary figures and images for: Downregulation of EB1 impedes Cx43 localization and cardiac conduction after hypothermic ischemia-reperfusion in rats (part 1 of 5)
Source: PeerJ. 2025 Apr 14;13:e19276. doi: 10.7717/peerj.19276 (PMC12005192; doi:10.7717/peerj.19276)

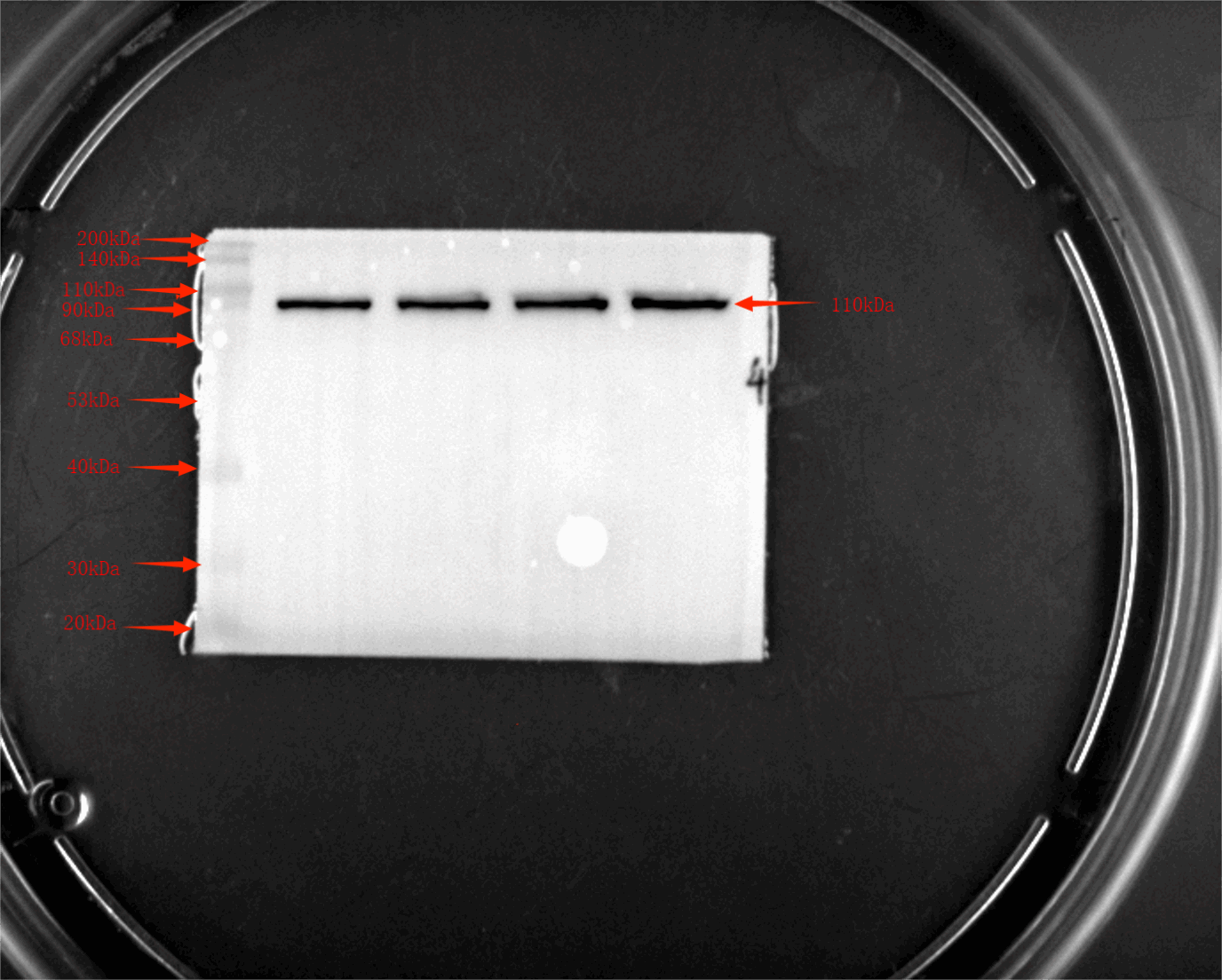

Supplement: Supplemental Information 3 [file peerj-13-19276-s003.zip › western blotting-marke/C I-R AAV9-CON AAV9-EB1 group western blot-membranal Cx43/1-ATPase-M-used(1).png]

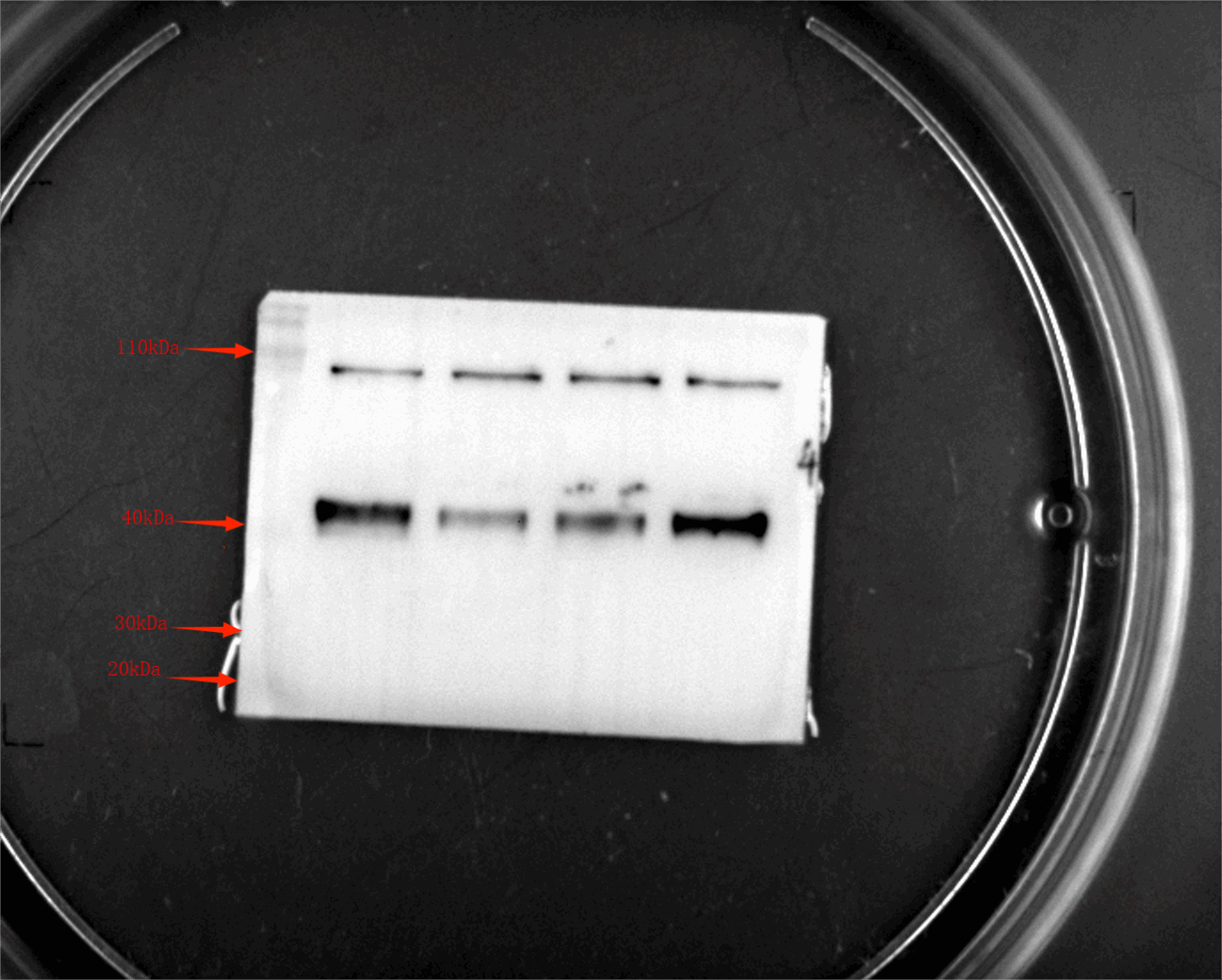

Supplement: Supplemental Information 3 [file peerj-13-19276-s003.zip › western blotting-marke/C I-R AAV9-CON AAV9-EB1 group western blot-membranal Cx43/1-Cx43-M-used(1).png]

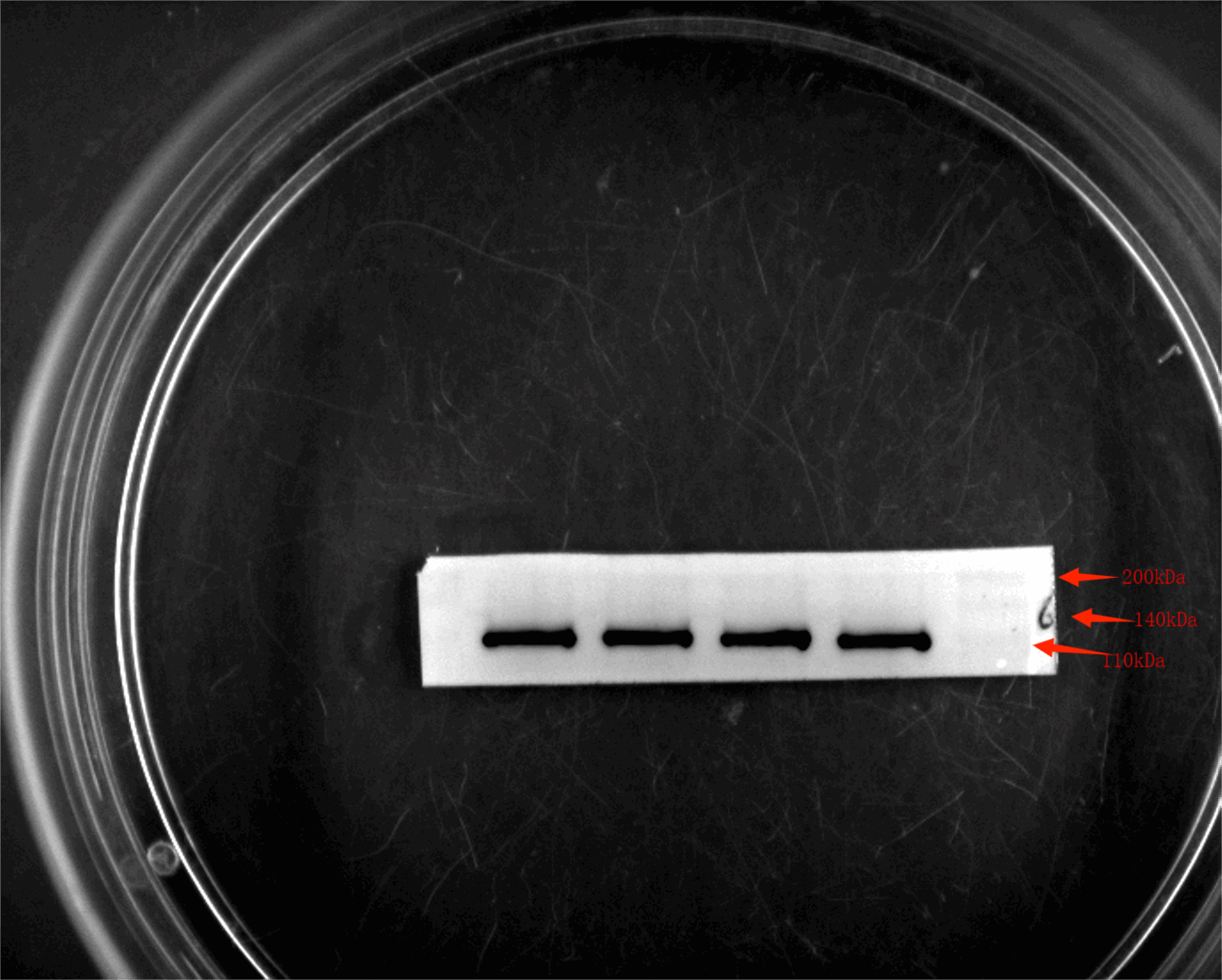

Supplement: Supplemental Information 3 [file peerj-13-19276-s003.zip › western blotting-marke/C I-R AAV9-CON AAV9-EB1 group western blot-membranal Cx43/2-ATPase-M(1).png]

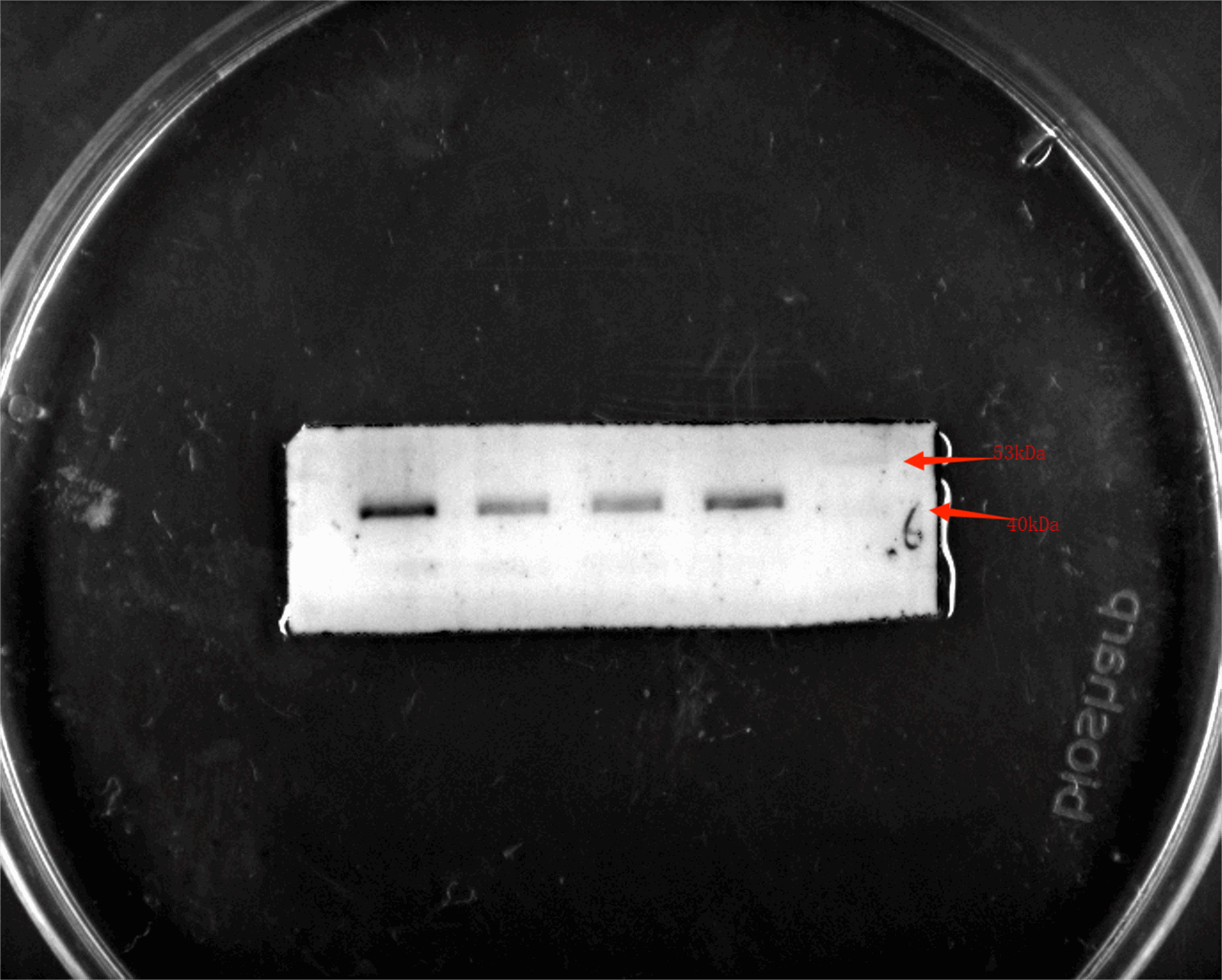

Supplement: Supplemental Information 3 [file peerj-13-19276-s003.zip › western blotting-marke/C I-R AAV9-CON AAV9-EB1 group western blot-membranal Cx43/2-Cx43-M(1).png]

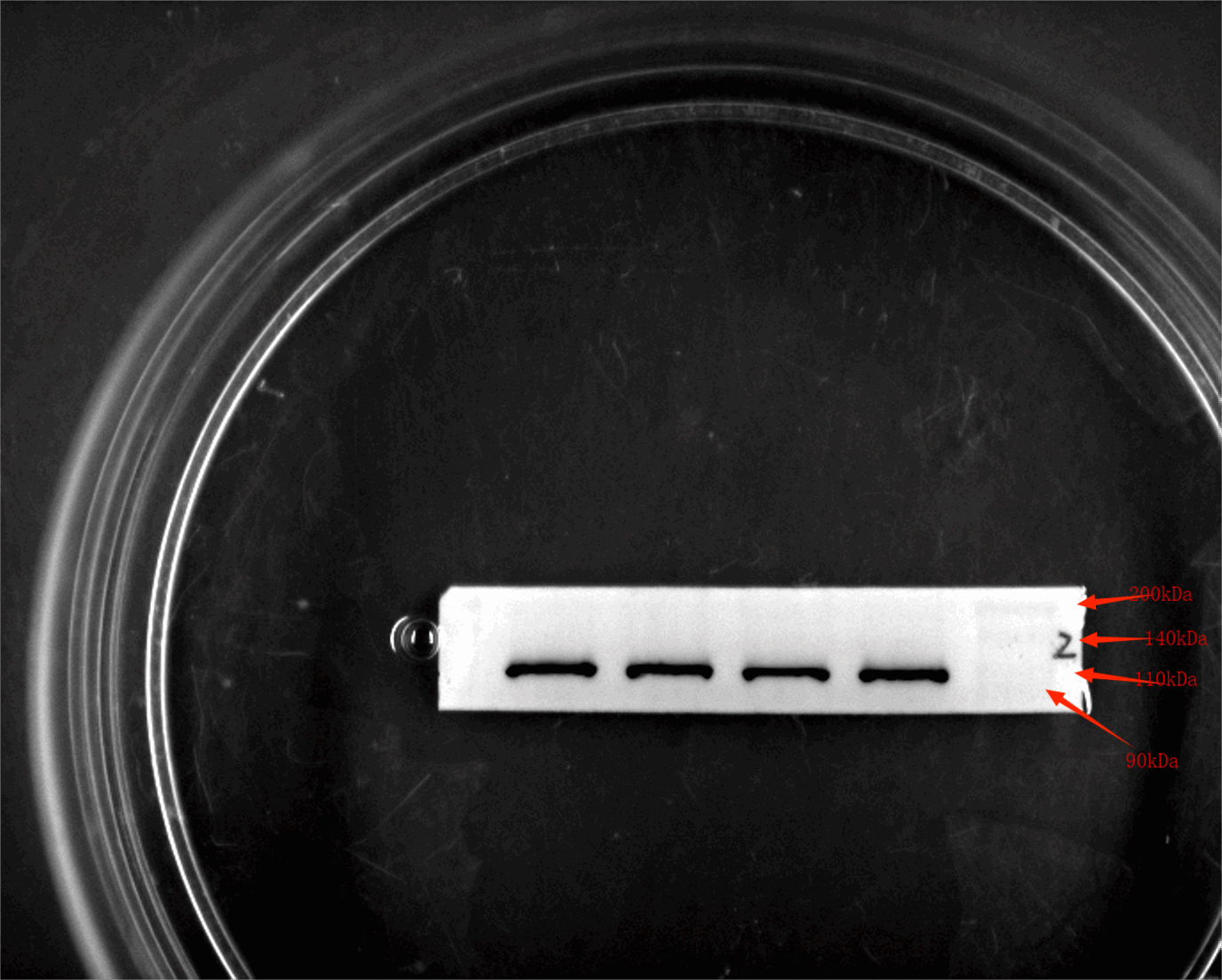

Supplement: Supplemental Information 3 [file peerj-13-19276-s003.zip › western blotting-marke/C I-R AAV9-CON AAV9-EB1 group western blot-membranal Cx43/3-ATPase-M(1).png]

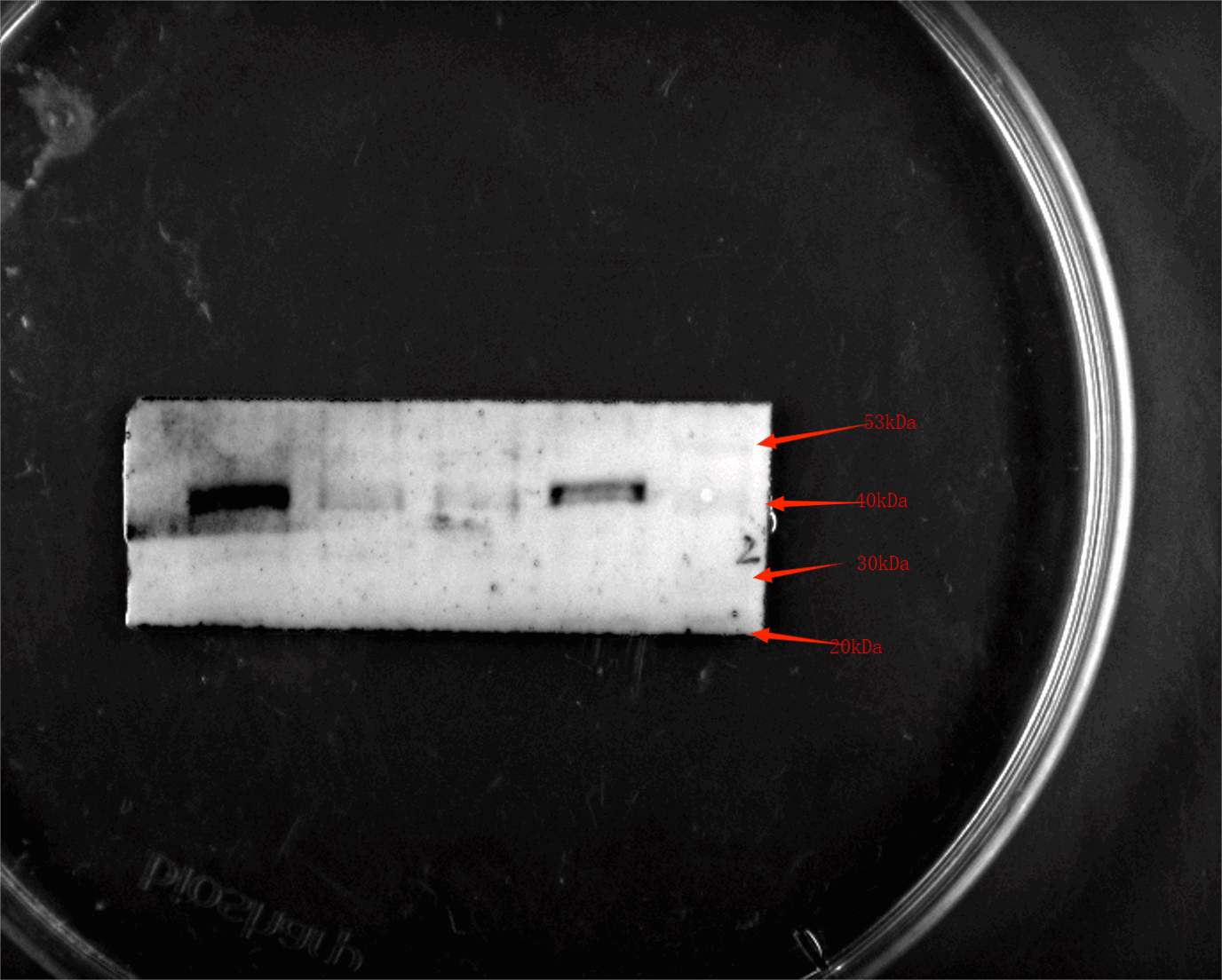

Supplement: Supplemental Information 3 [file peerj-13-19276-s003.zip › western blotting-marke/C I-R AAV9-CON AAV9-EB1 group western blot-membranal Cx43/3-Cx43-M(1).png]

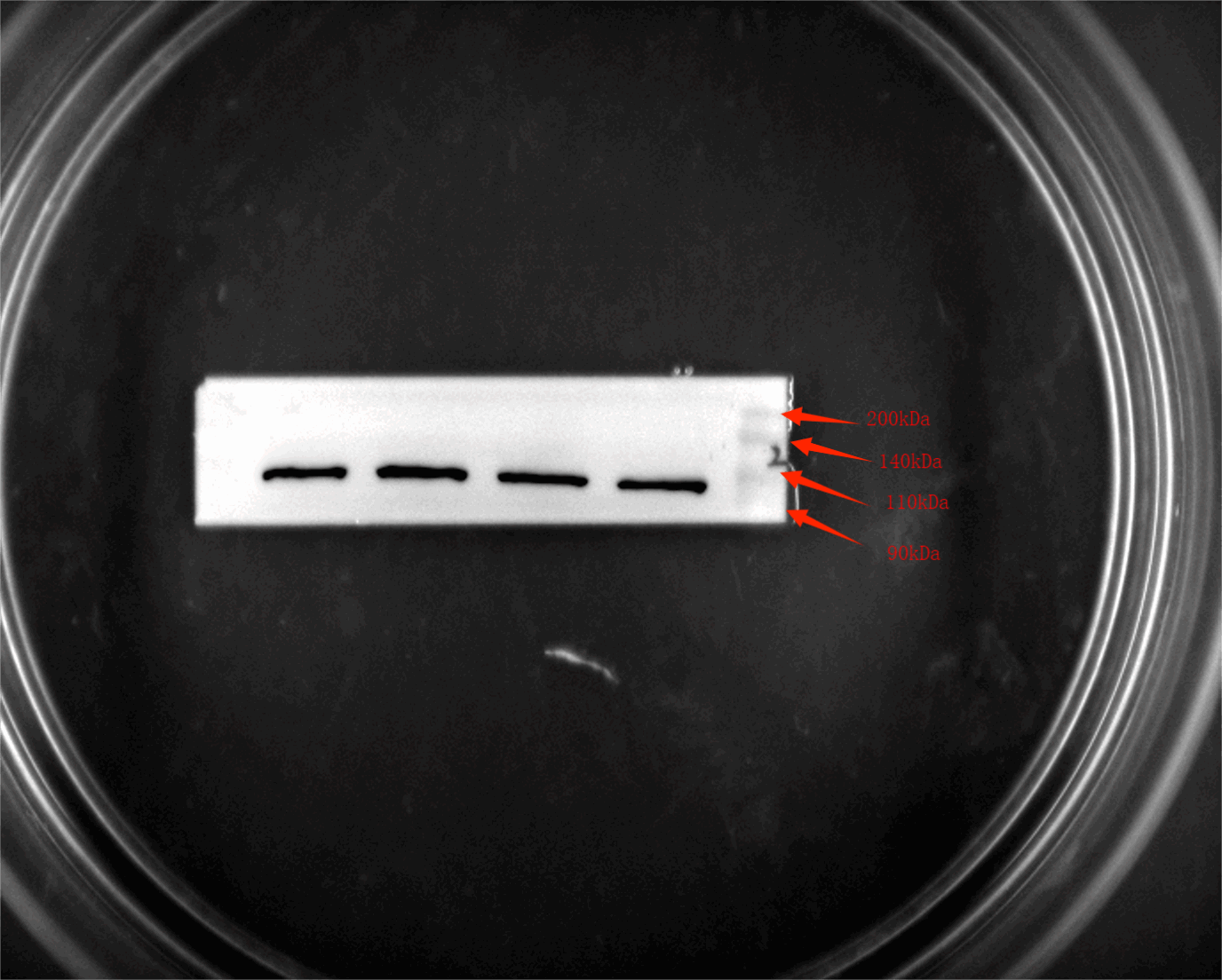

Supplement: Supplemental Information 3 [file peerj-13-19276-s003.zip › western blotting-marke/C I-R AAV9-CON AAV9-EB1 group western blot-membranal Cx43/4-ATPase-M(1).png]

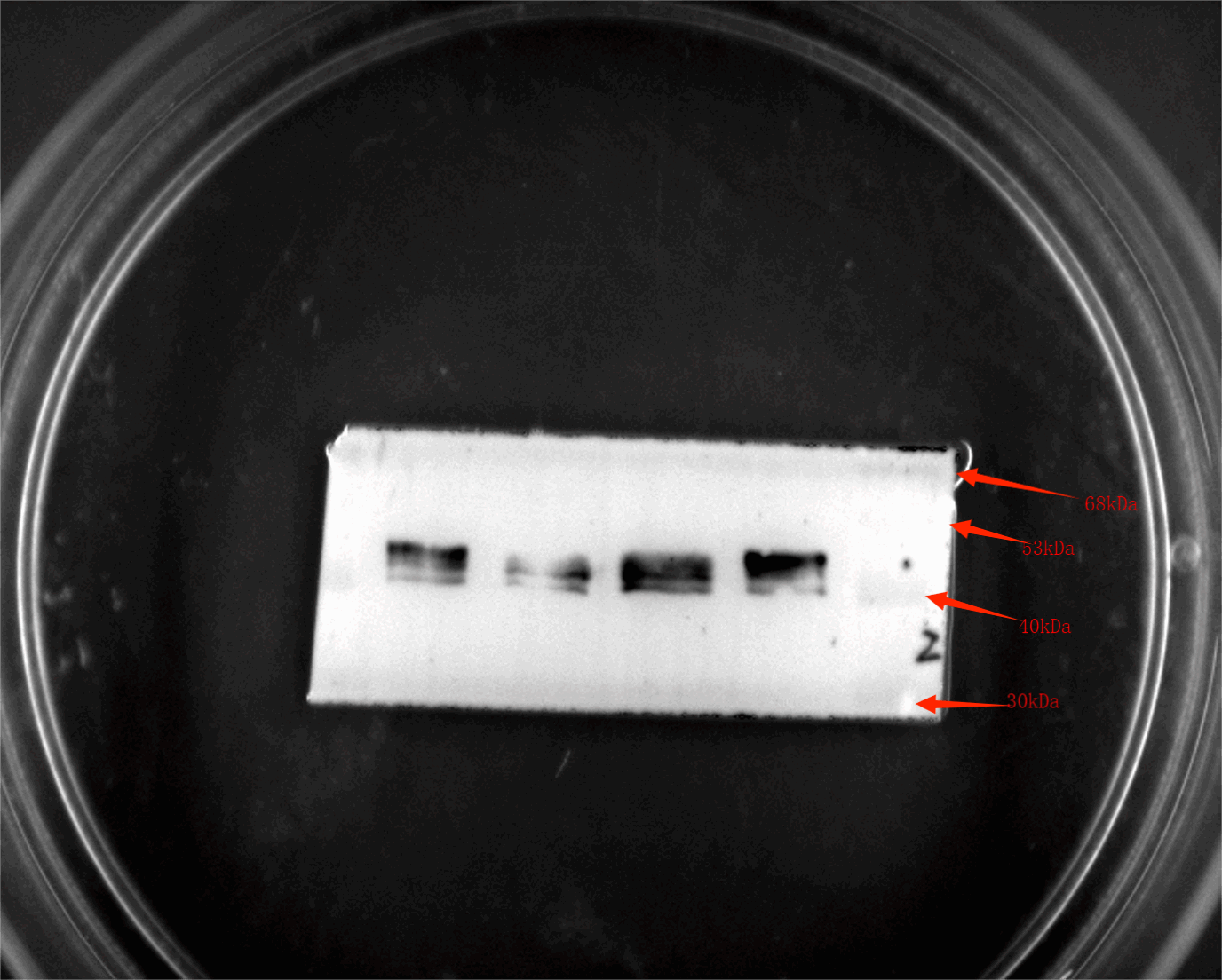

Supplement: Supplemental Information 3 [file peerj-13-19276-s003.zip › western blotting-marke/C I-R AAV9-CON AAV9-EB1 group western blot-membranal Cx43/4-Cx43-M(1).png]

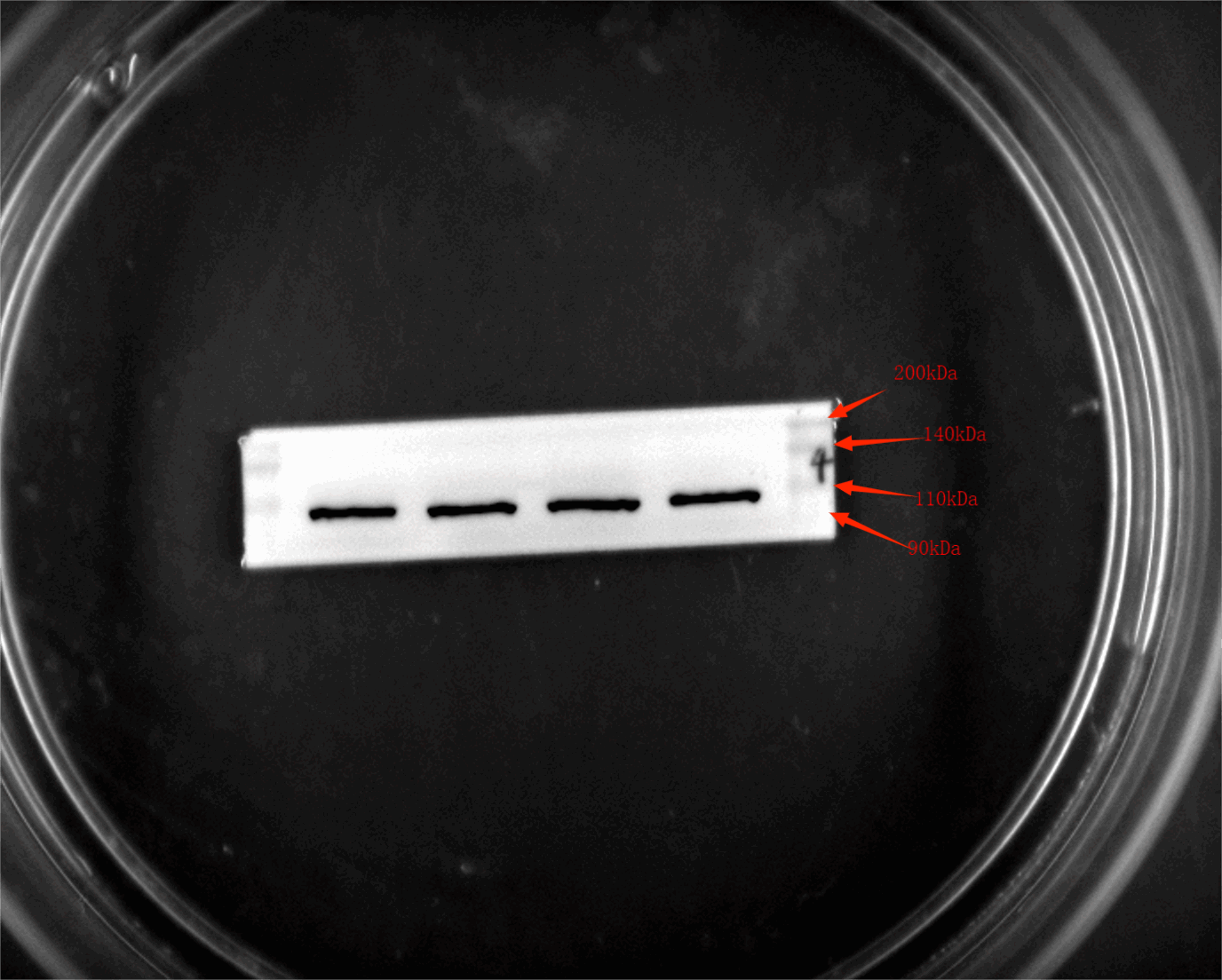

Supplement: Supplemental Information 3 [file peerj-13-19276-s003.zip › western blotting-marke/C I-R AAV9-CON AAV9-EB1 group western blot-membranal Cx43/5-ATPase-M(1).png]

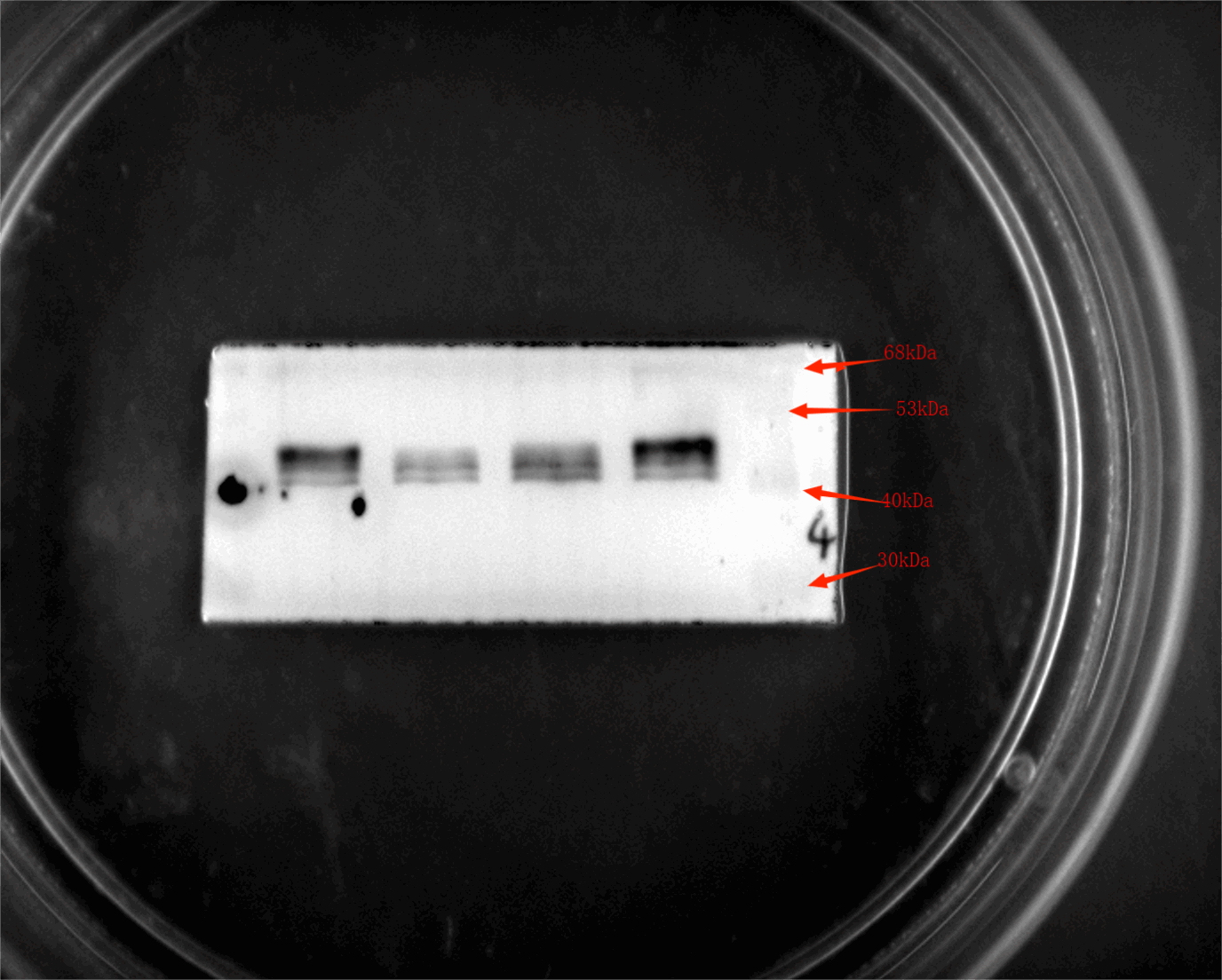

Supplement: Supplemental Information 3 [file peerj-13-19276-s003.zip › western blotting-marke/C I-R AAV9-CON AAV9-EB1 group western blot-membranal Cx43/5-Cx43-M(1).png]

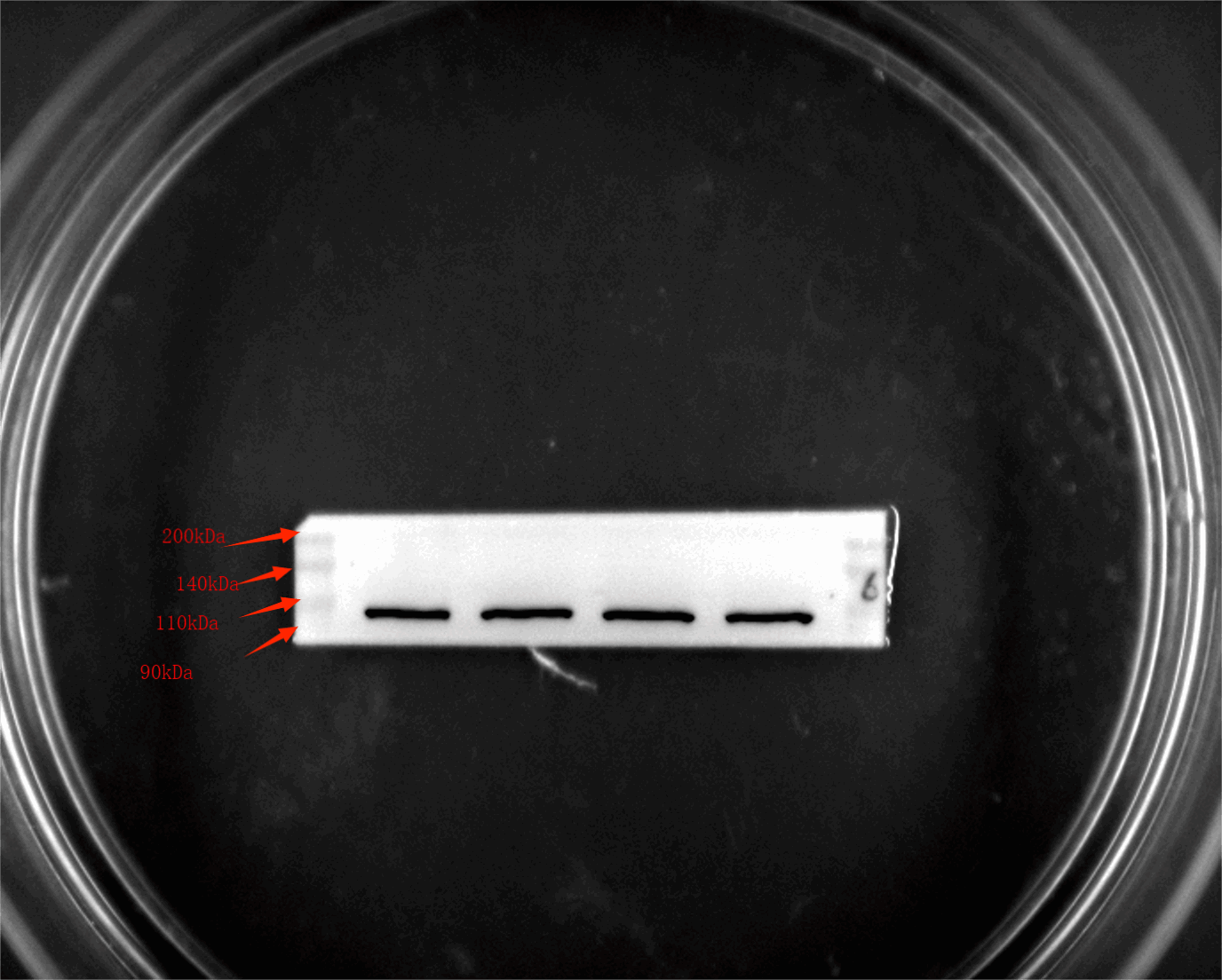

Supplement: Supplemental Information 3 [file peerj-13-19276-s003.zip › western blotting-marke/C I-R AAV9-CON AAV9-EB1 group western blot-membranal Cx43/6-ATPase-M(1).png]

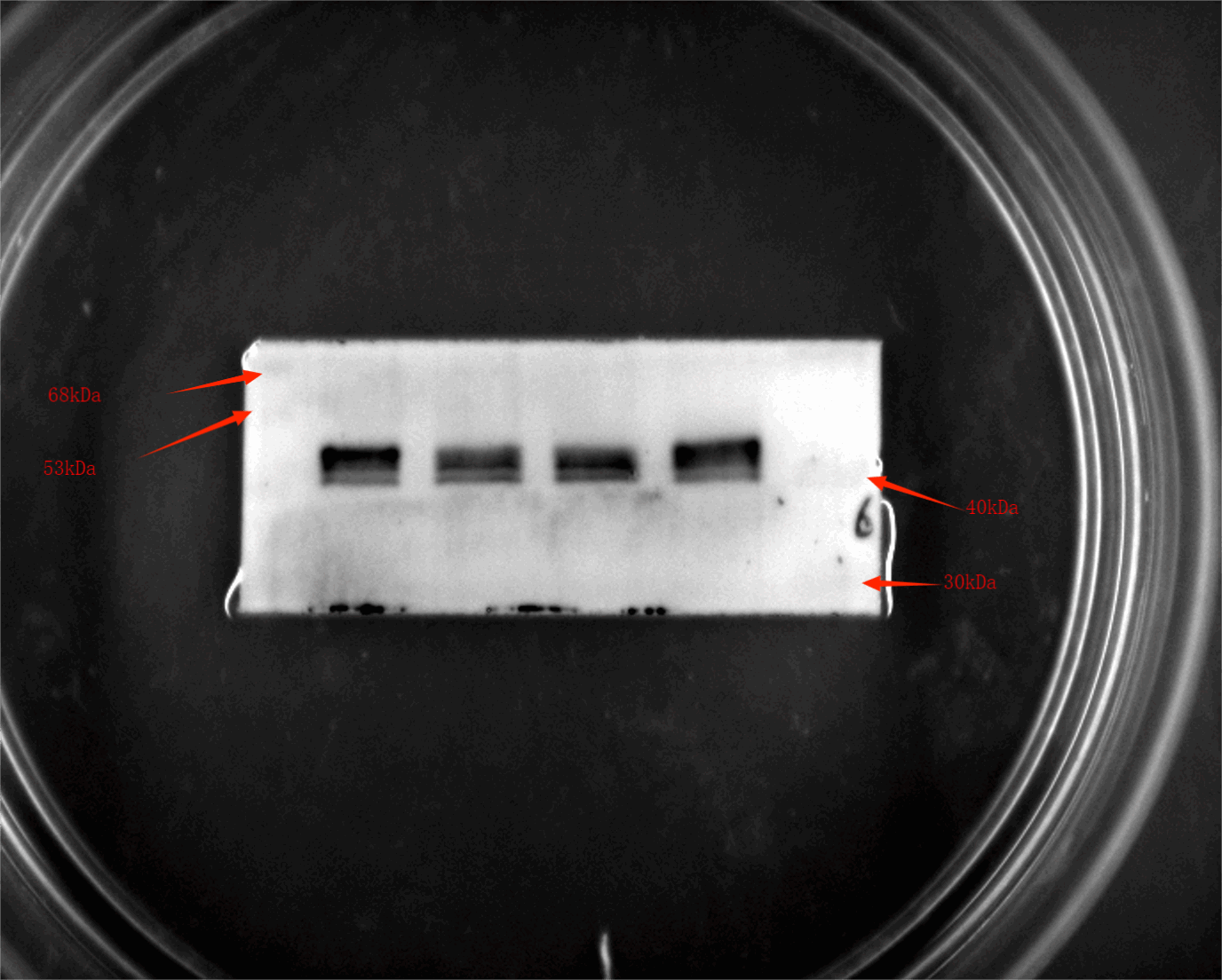

Supplement: Supplemental Information 3 [file peerj-13-19276-s003.zip › western blotting-marke/C I-R AAV9-CON AAV9-EB1 group western blot-membranal Cx43/6-Cx43-M(1).png]

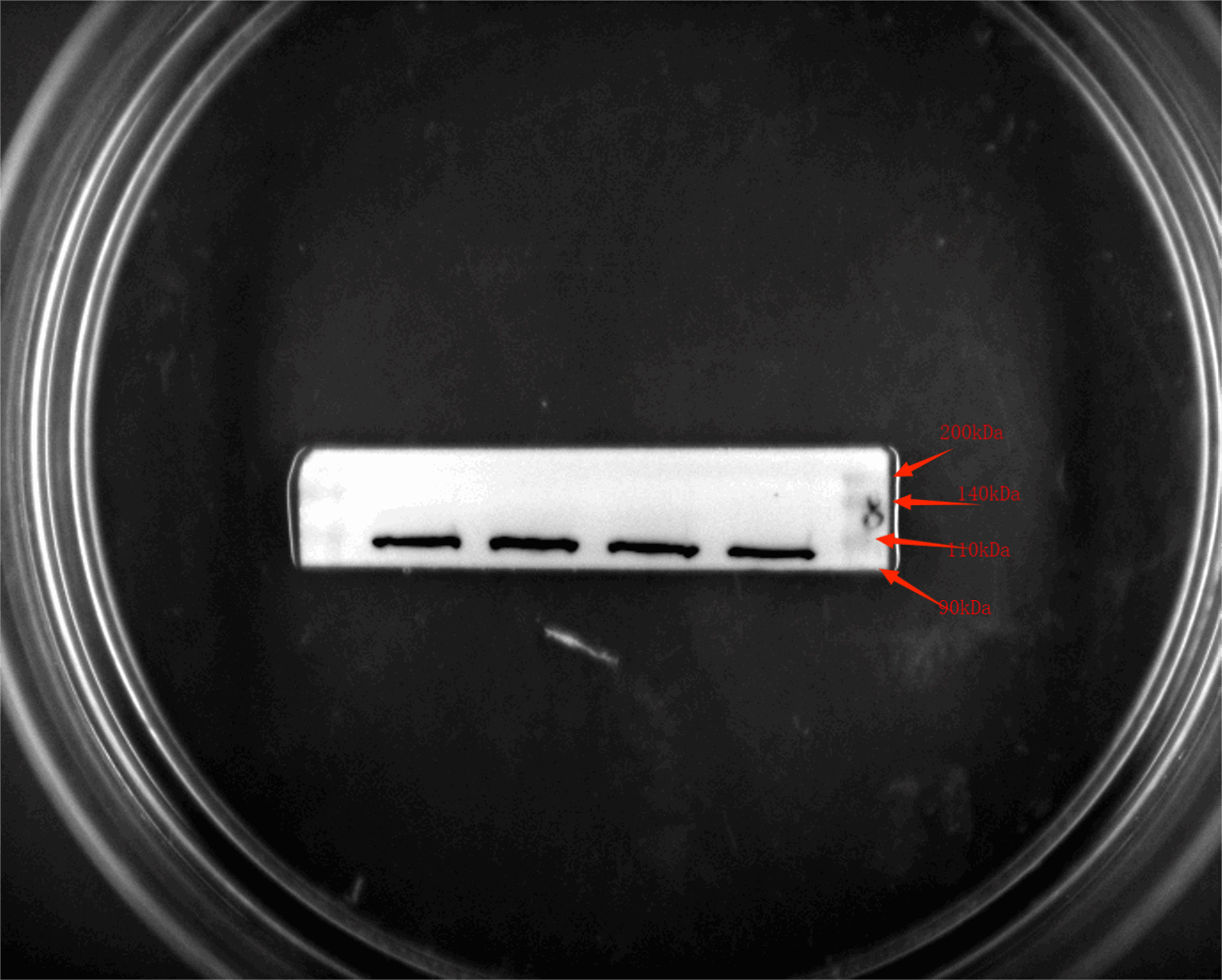

Supplement: Supplemental Information 3 [file peerj-13-19276-s003.zip › western blotting-marke/C I-R AAV9-CON AAV9-EB1 group western blot-membranal Cx43/7-ATPase-M(1).png]

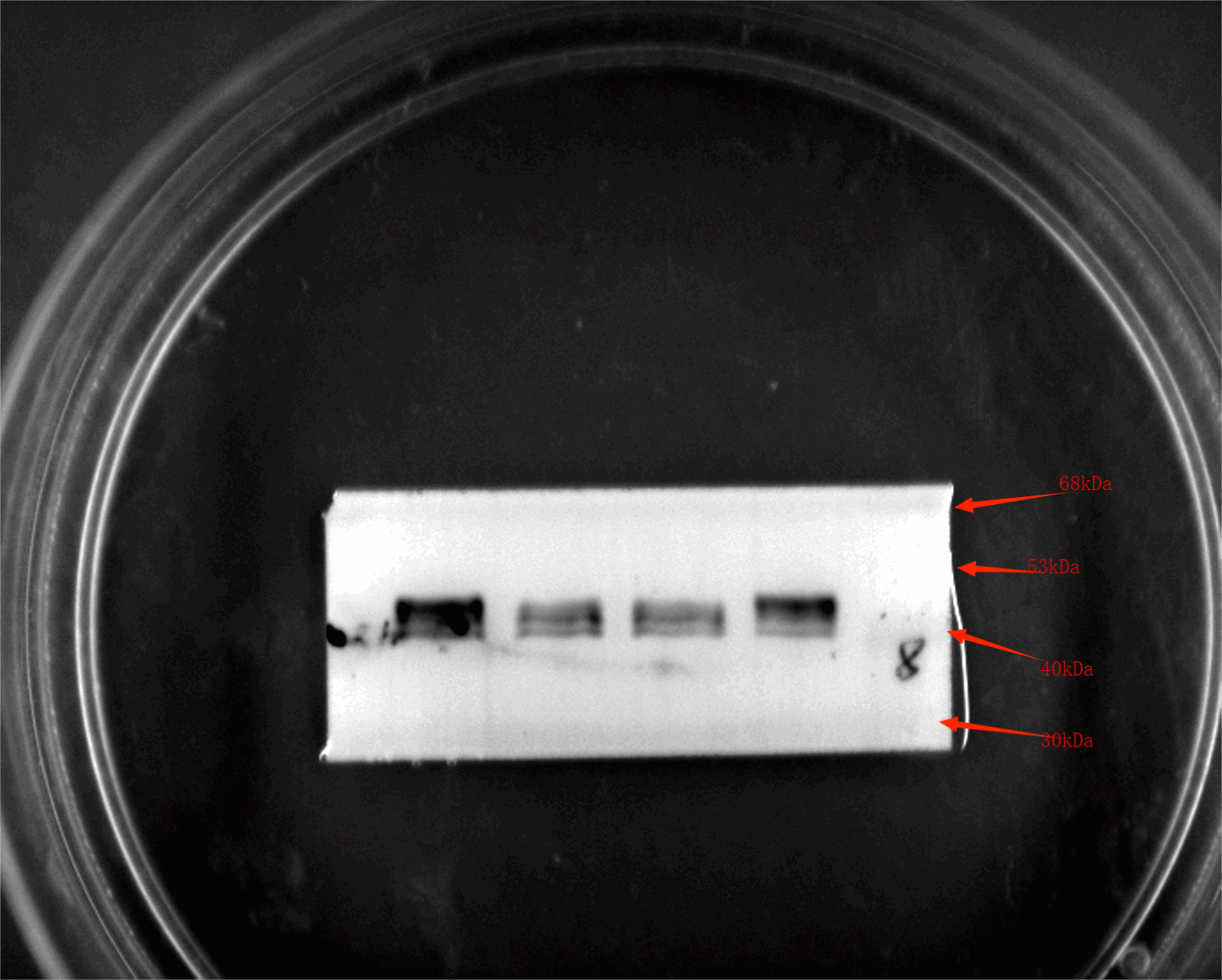

Supplement: Supplemental Information 3 [file peerj-13-19276-s003.zip › western blotting-marke/C I-R AAV9-CON AAV9-EB1 group western blot-membranal Cx43/7-Cx43-M(1).png]

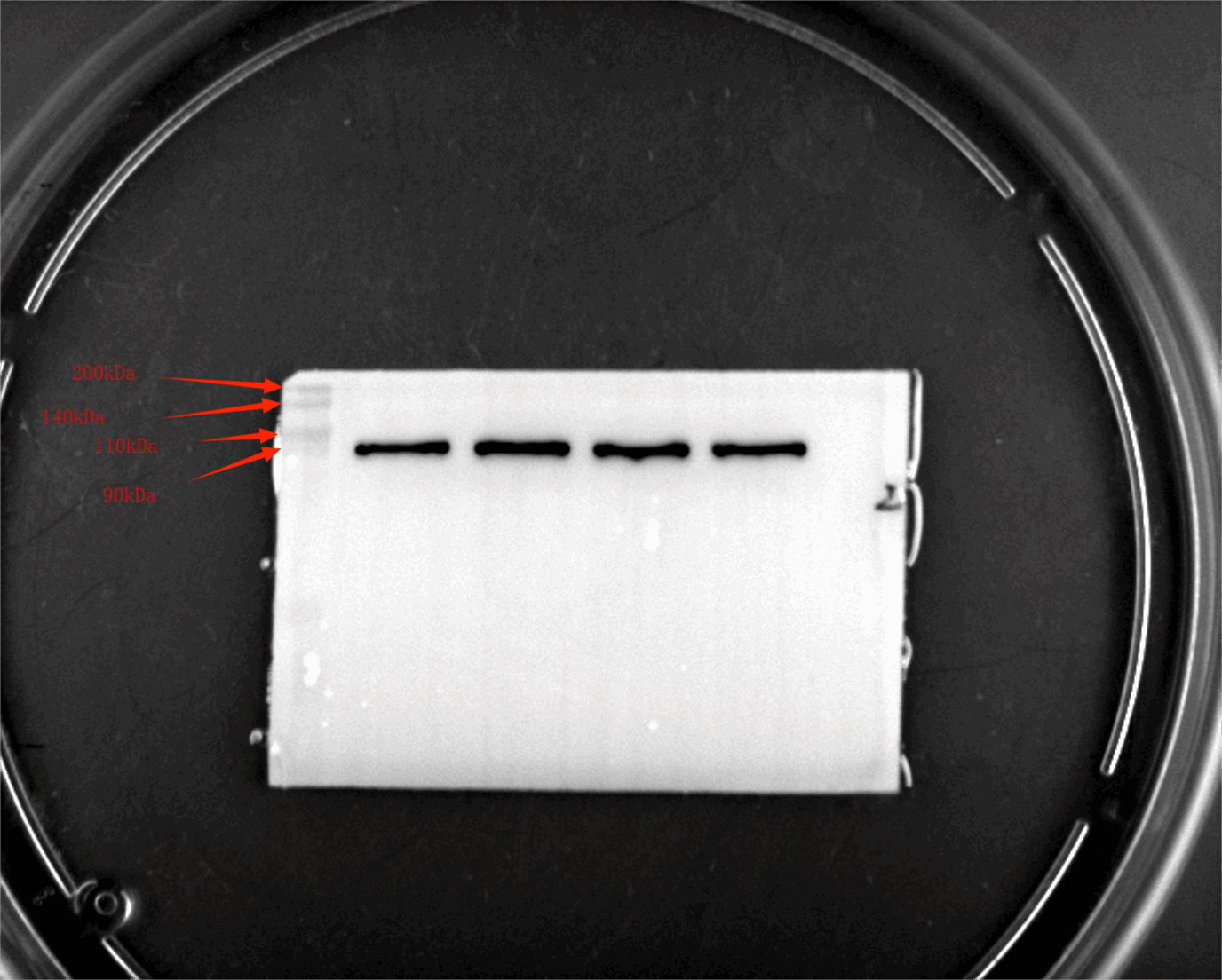

Supplement: Supplemental Information 3 [file peerj-13-19276-s003.zip › western blotting-marke/C I-R AAV9-CON AAV9-EB1 group western blot-membranal Cx43/8-ATPase-M(1).png]

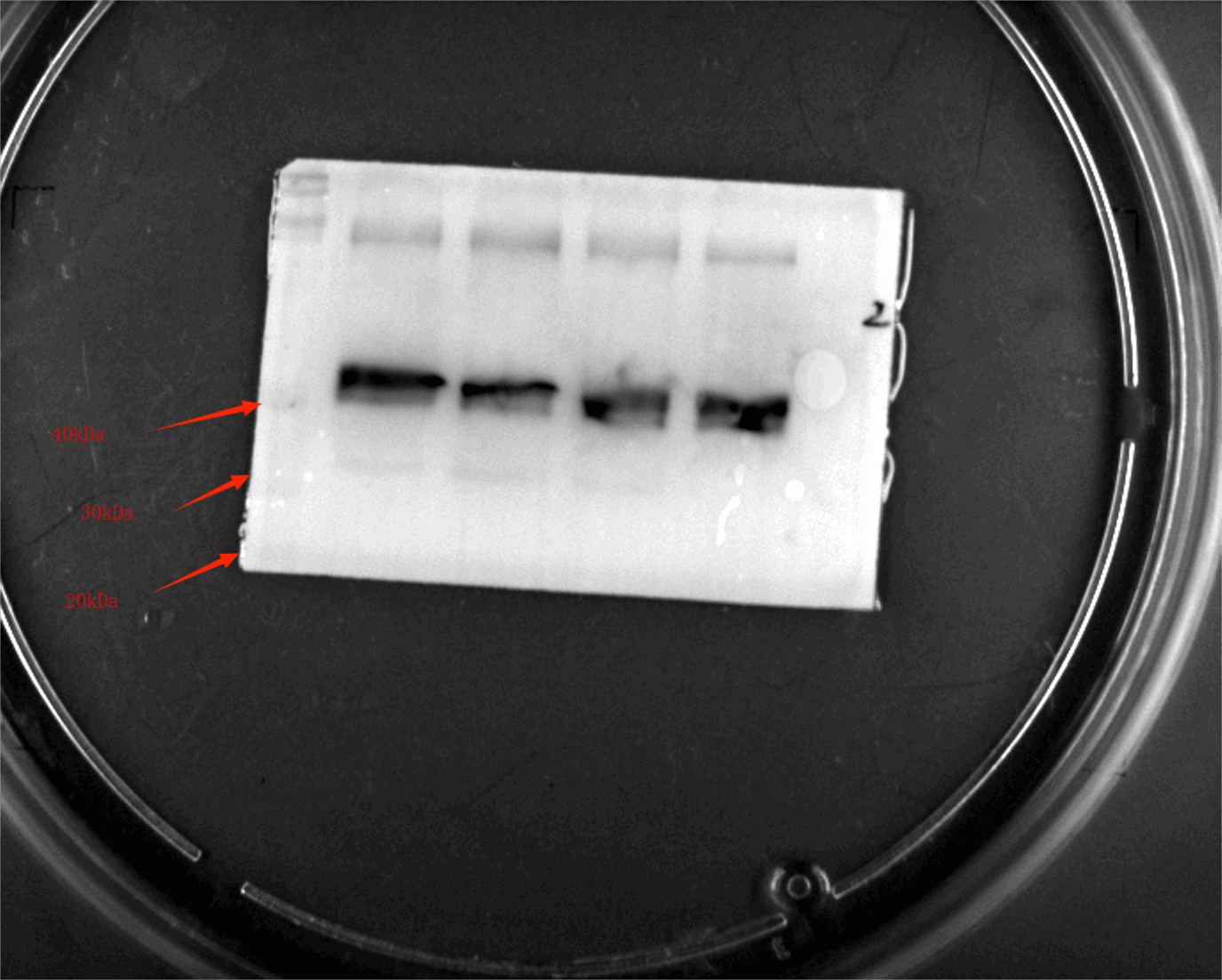

Supplement: Supplemental Information 3 [file peerj-13-19276-s003.zip › western blotting-marke/C I-R AAV9-CON AAV9-EB1 group western blot-membranal Cx43/8-Cx43-M(1).png]

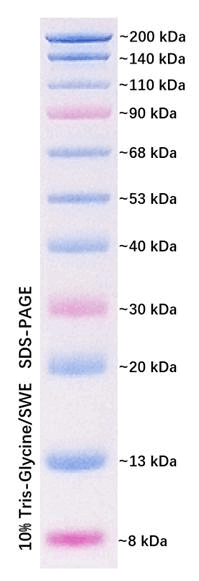

Supplement: Supplemental Information 3 [file peerj-13-19276-s003.zip › western blotting-marke/C I-R AAV9-CON AAV9-EB1 group western blot-membranal Cx43/marker reference diagram.png]

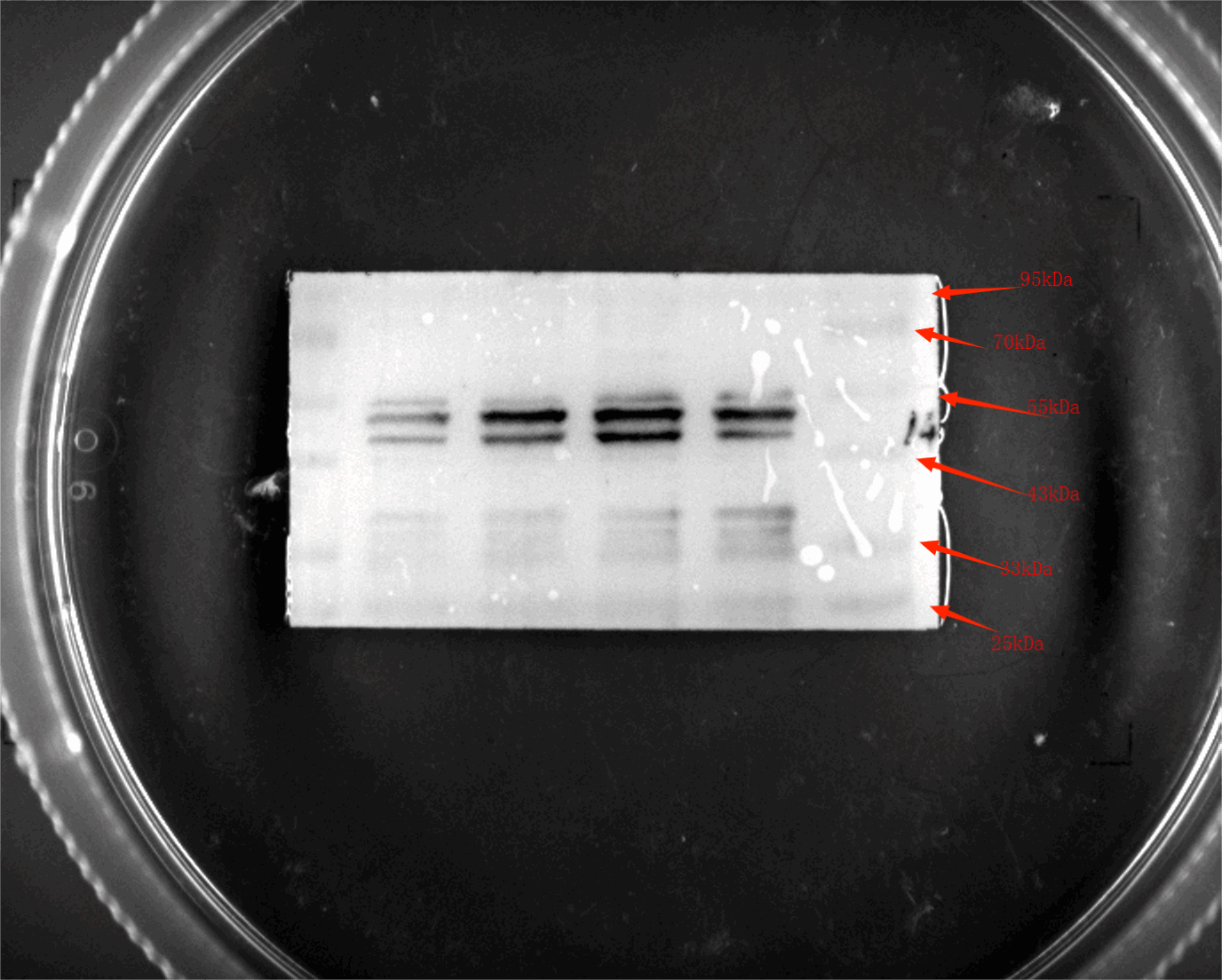

Supplement: Supplemental Information 3 [file peerj-13-19276-s003.zip › western blotting-marke/western blot-free tubulin/1-Free tubulin-M(1).png]

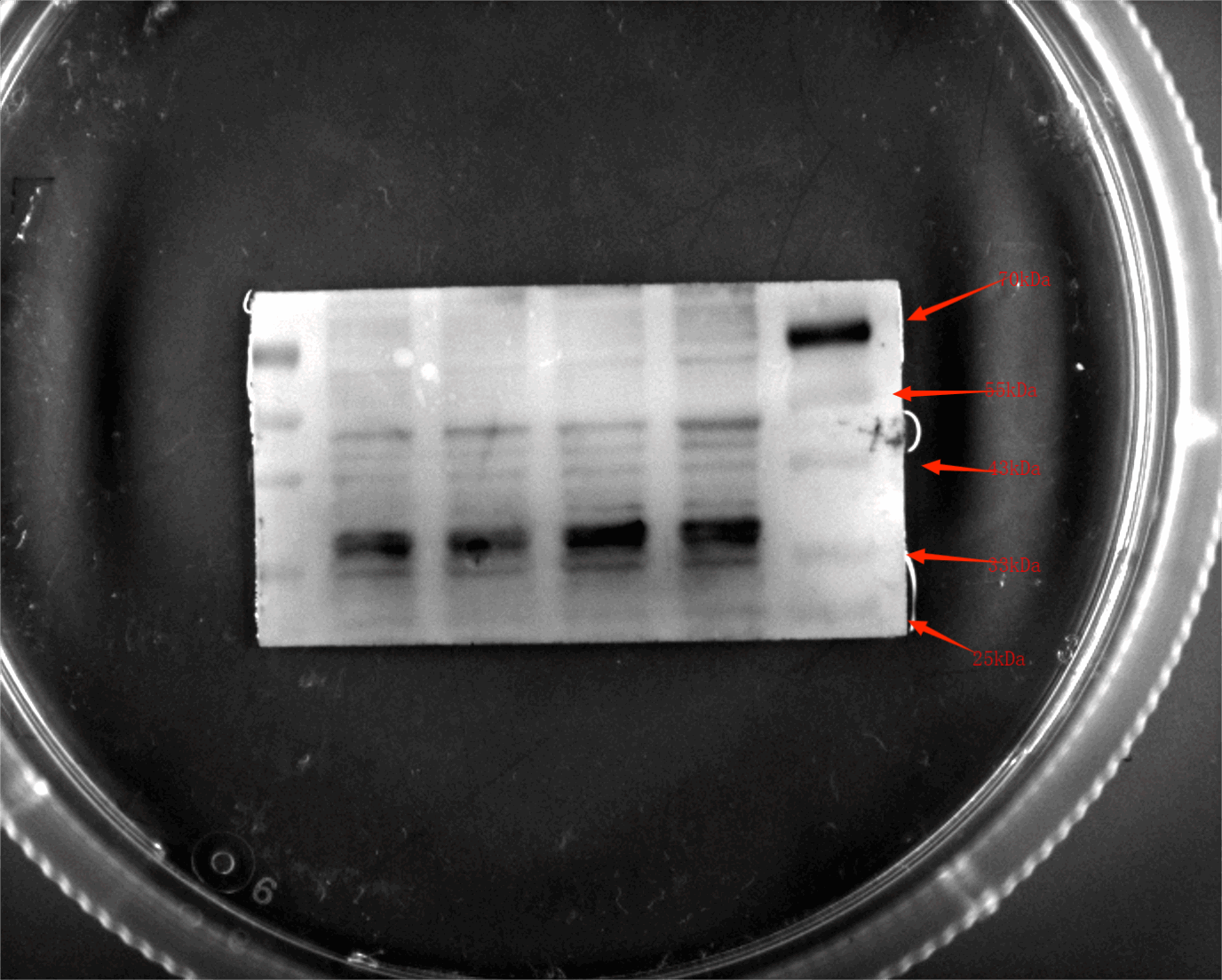

Supplement: Supplemental Information 3 [file peerj-13-19276-s003.zip › western blotting-marke/western blot-free tubulin/1-GAPDH-M(1).png]

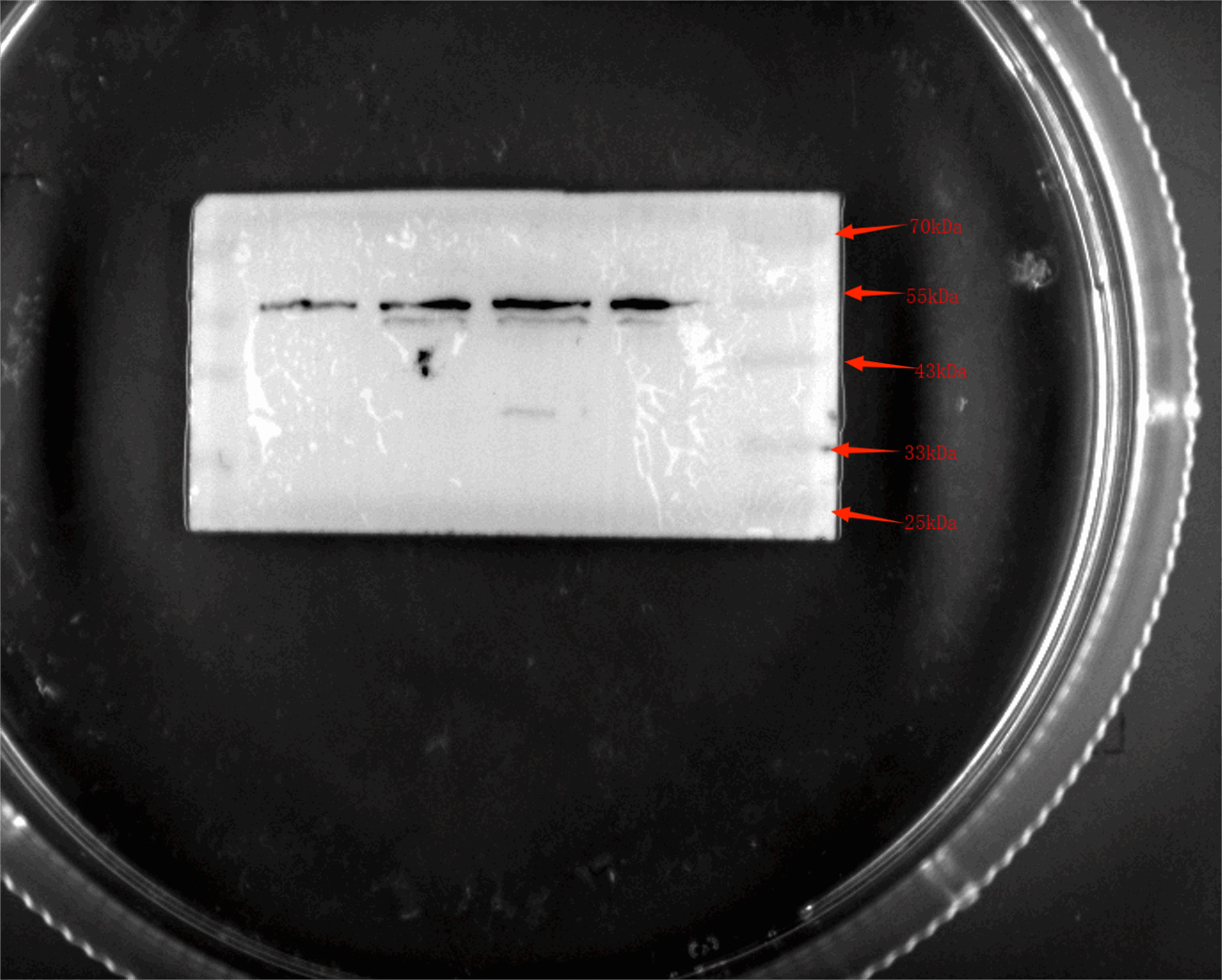

Supplement: Supplemental Information 3 [file peerj-13-19276-s003.zip › western blotting-marke/western blot-free tubulin/2-Free tubulin-M(1).png]

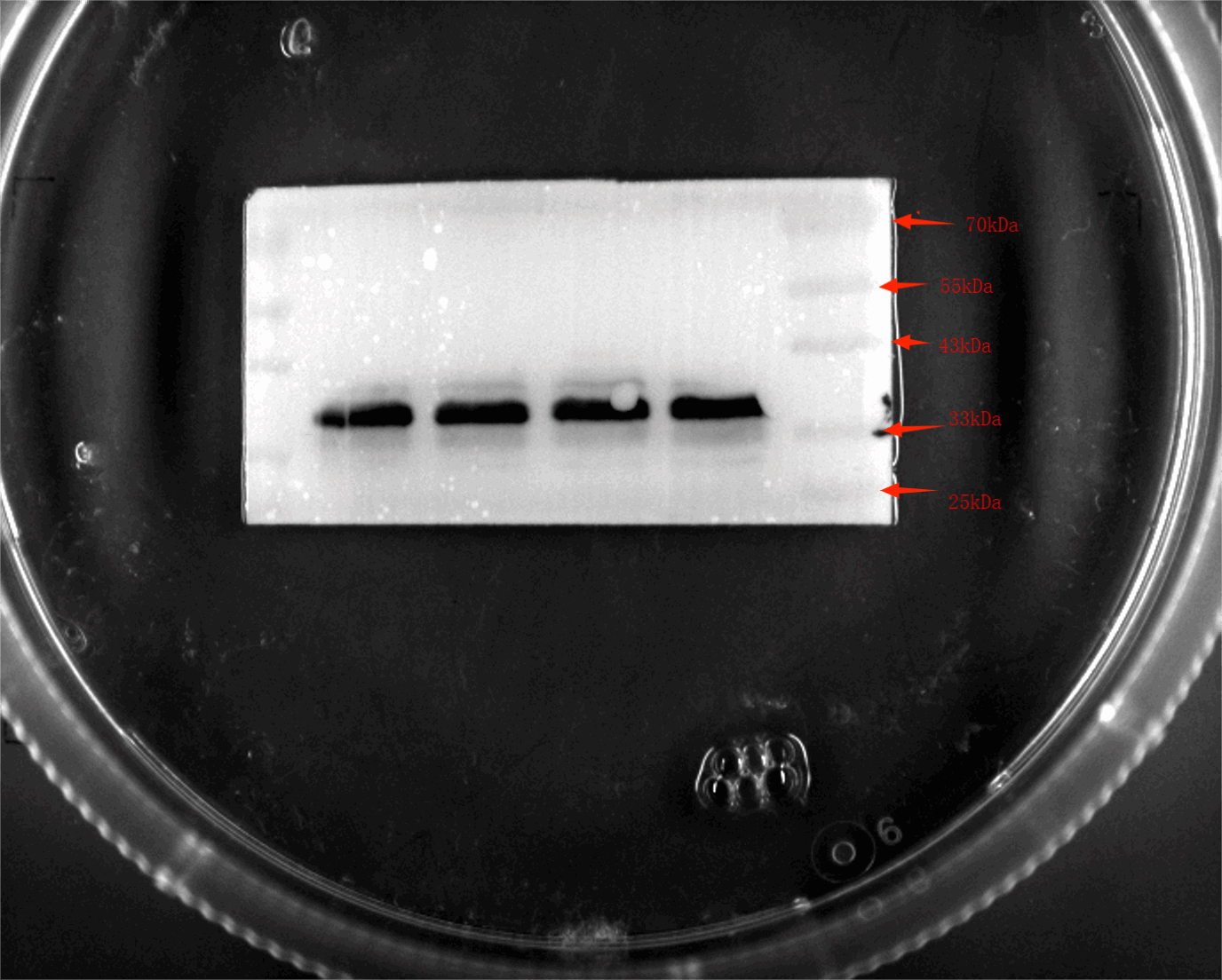

Supplement: Supplemental Information 3 [file peerj-13-19276-s003.zip › western blotting-marke/western blot-free tubulin/2-GAPDH-M(1).png]

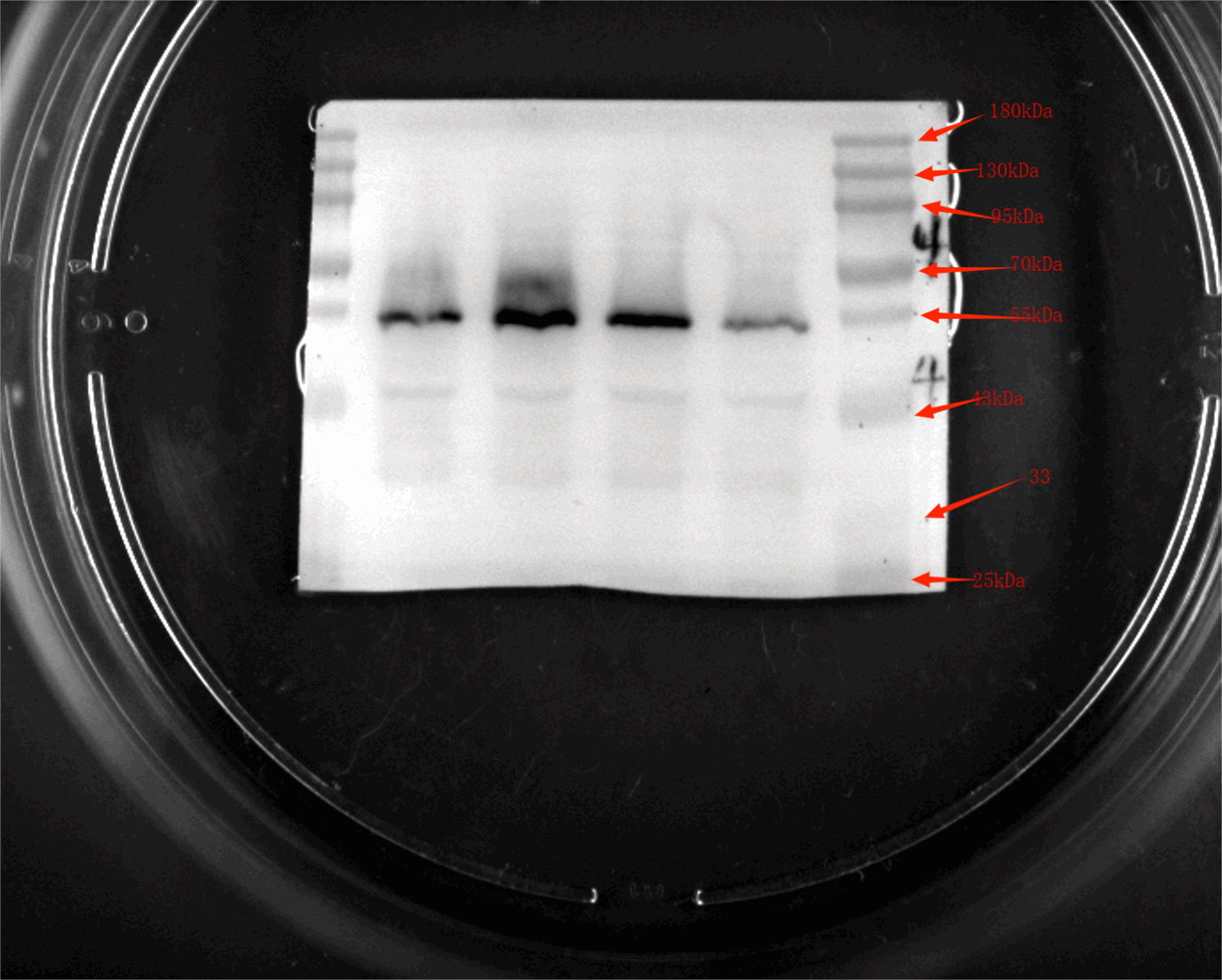

Supplement: Supplemental Information 3 [file peerj-13-19276-s003.zip › western blotting-marke/western blot-free tubulin/3-Free tubulin-M-used(1).png]

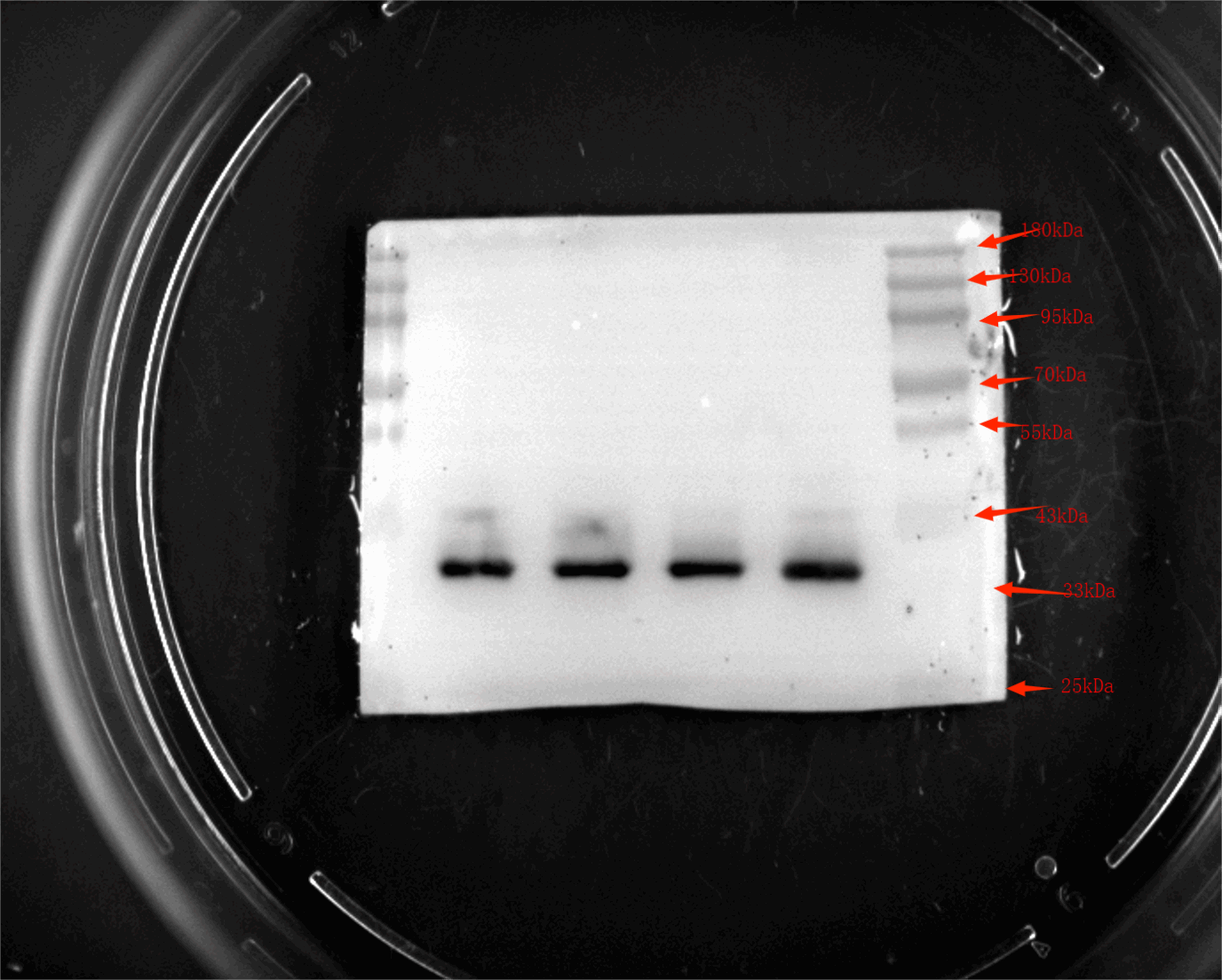

Supplement: Supplemental Information 3 [file peerj-13-19276-s003.zip › western blotting-marke/western blot-free tubulin/3-GAPDH-M-used(1).png]

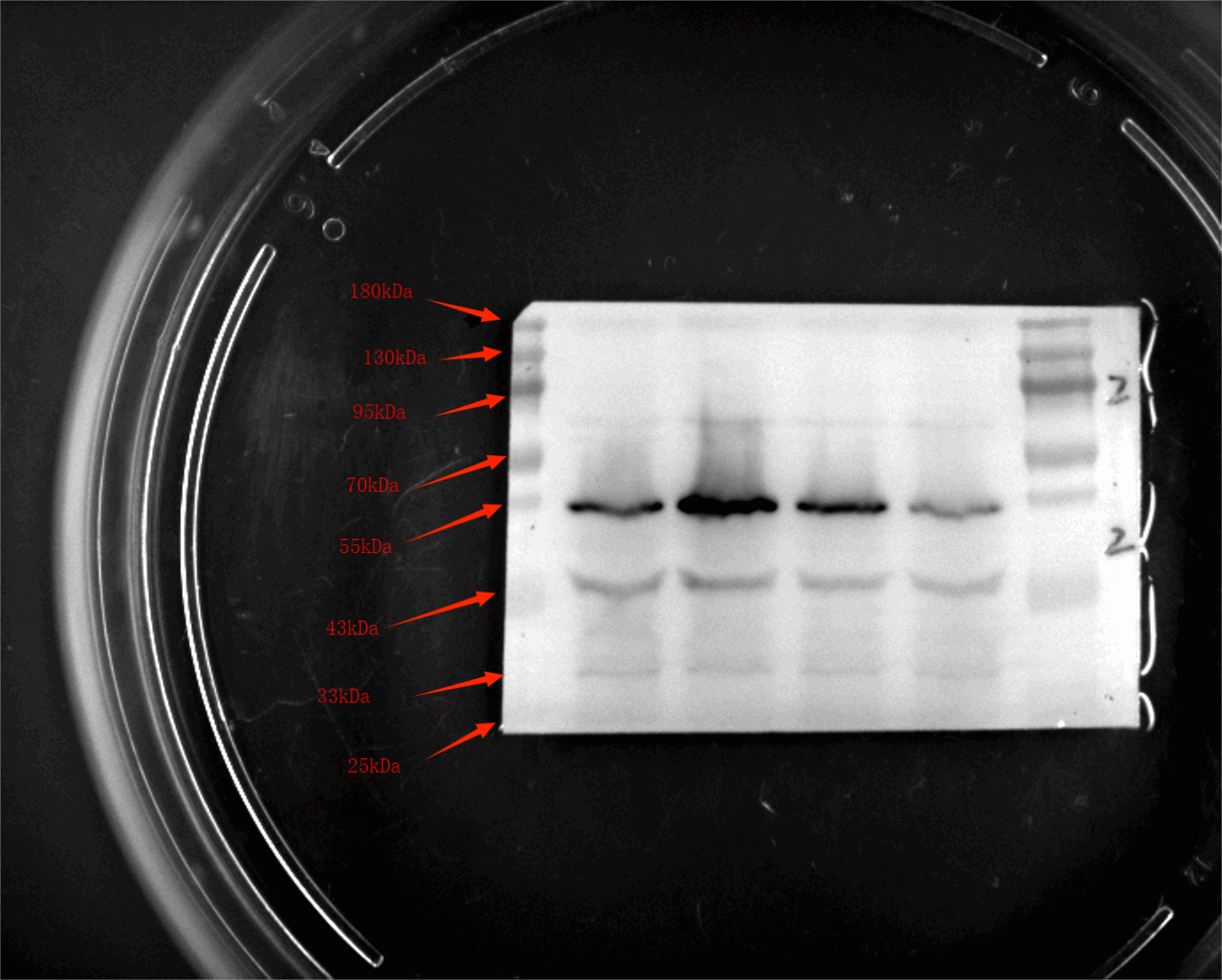

Supplement: Supplemental Information 3 [file peerj-13-19276-s003.zip › western blotting-marke/western blot-free tubulin/4-Free tubulin-M(1).png]

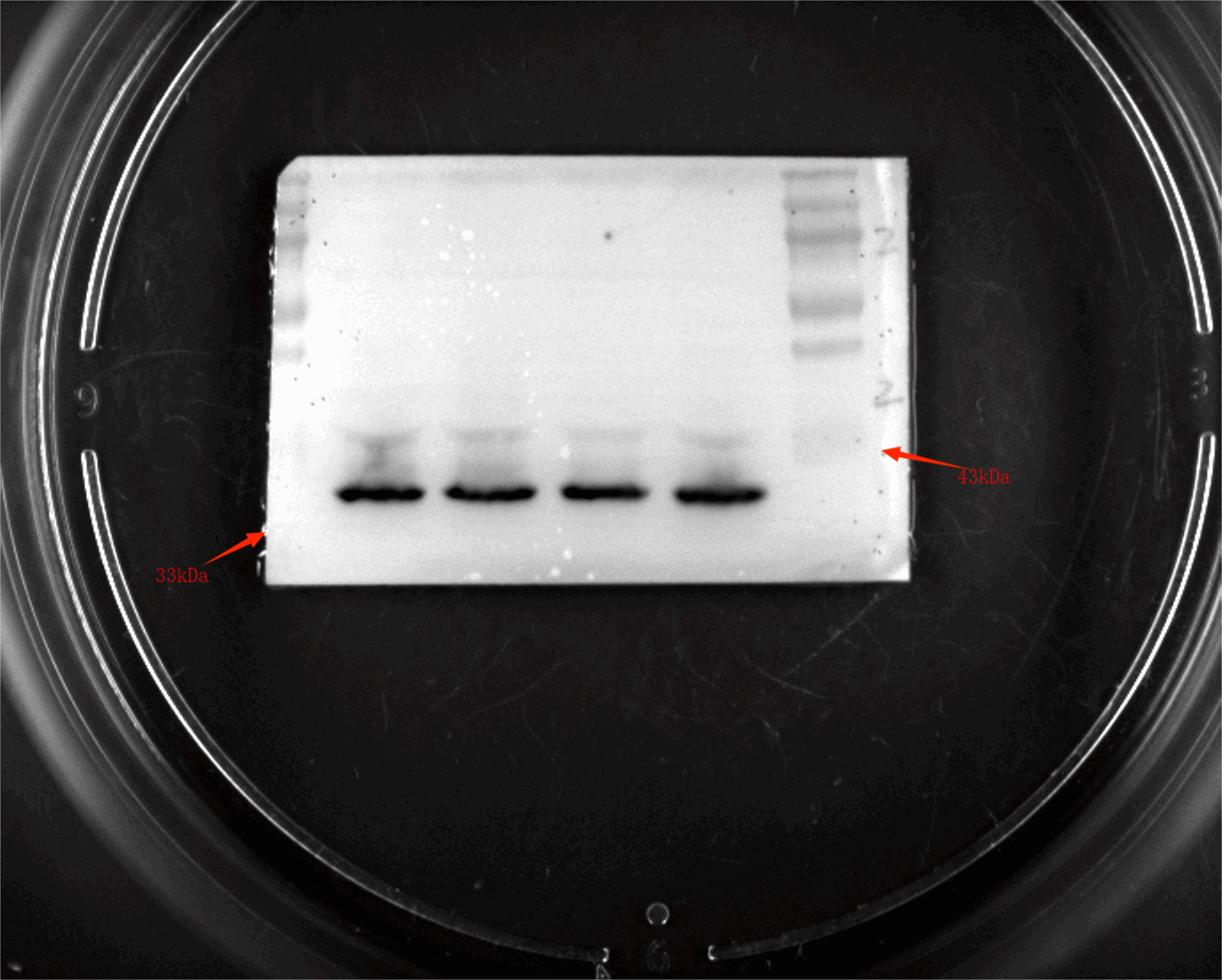

Supplement: Supplemental Information 3 [file peerj-13-19276-s003.zip › western blotting-marke/western blot-free tubulin/4-GAPDH-M(1).png]

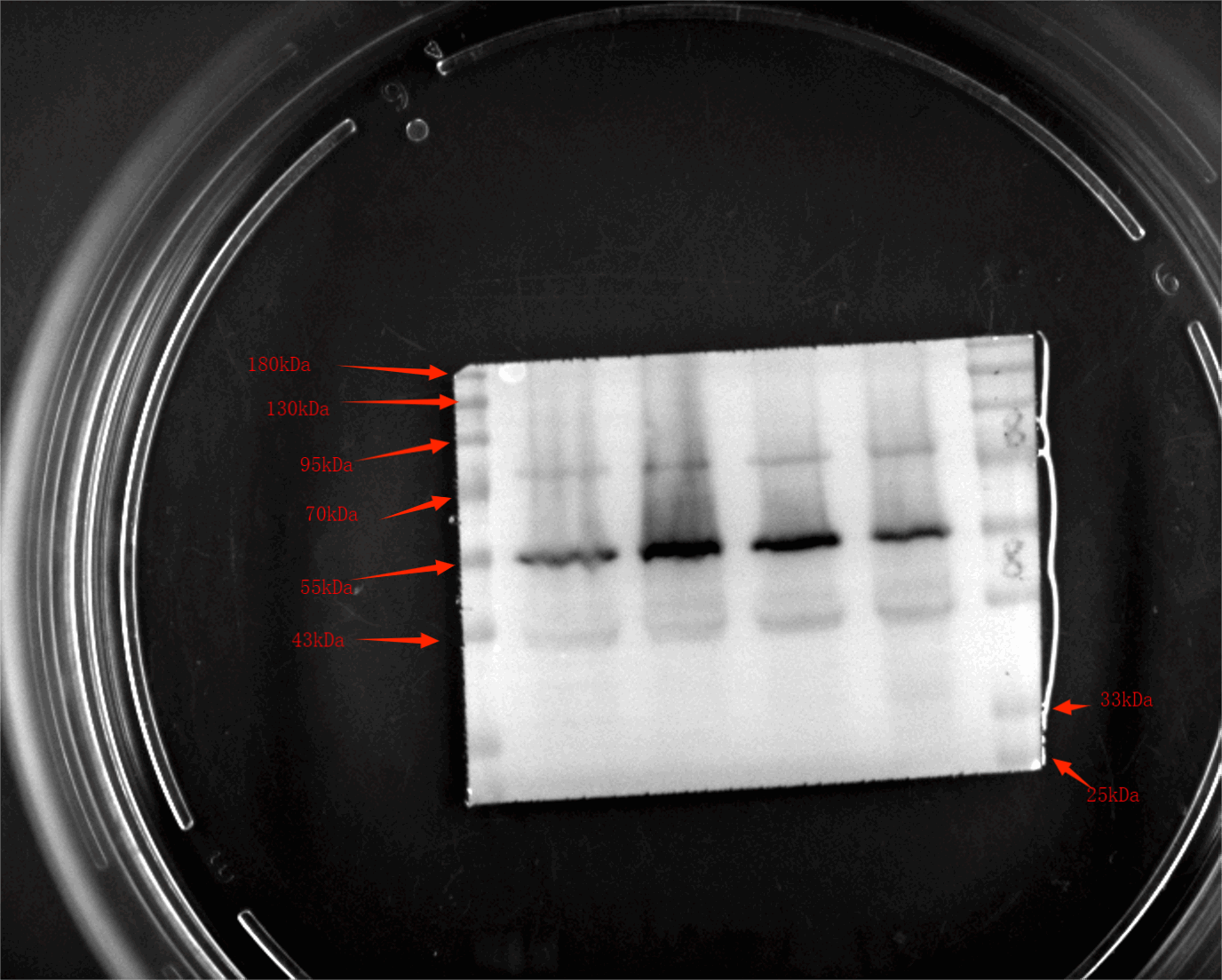

Supplement: Supplemental Information 3 [file peerj-13-19276-s003.zip › western blotting-marke/western blot-free tubulin/5-Free tubulin-M(1).png]

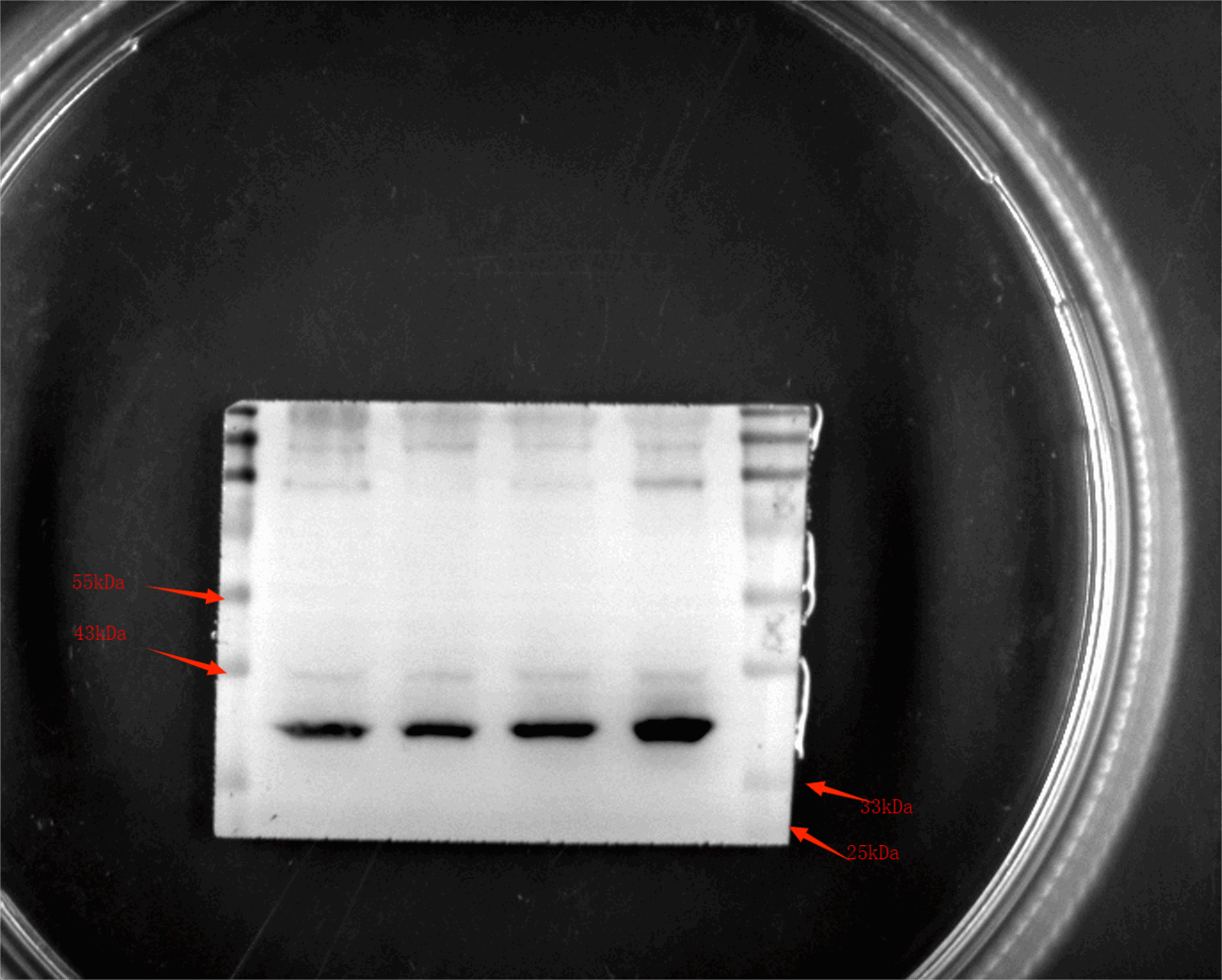

Supplement: Supplemental Information 3 [file peerj-13-19276-s003.zip › western blotting-marke/western blot-free tubulin/5-GAPDH-M(1).png]

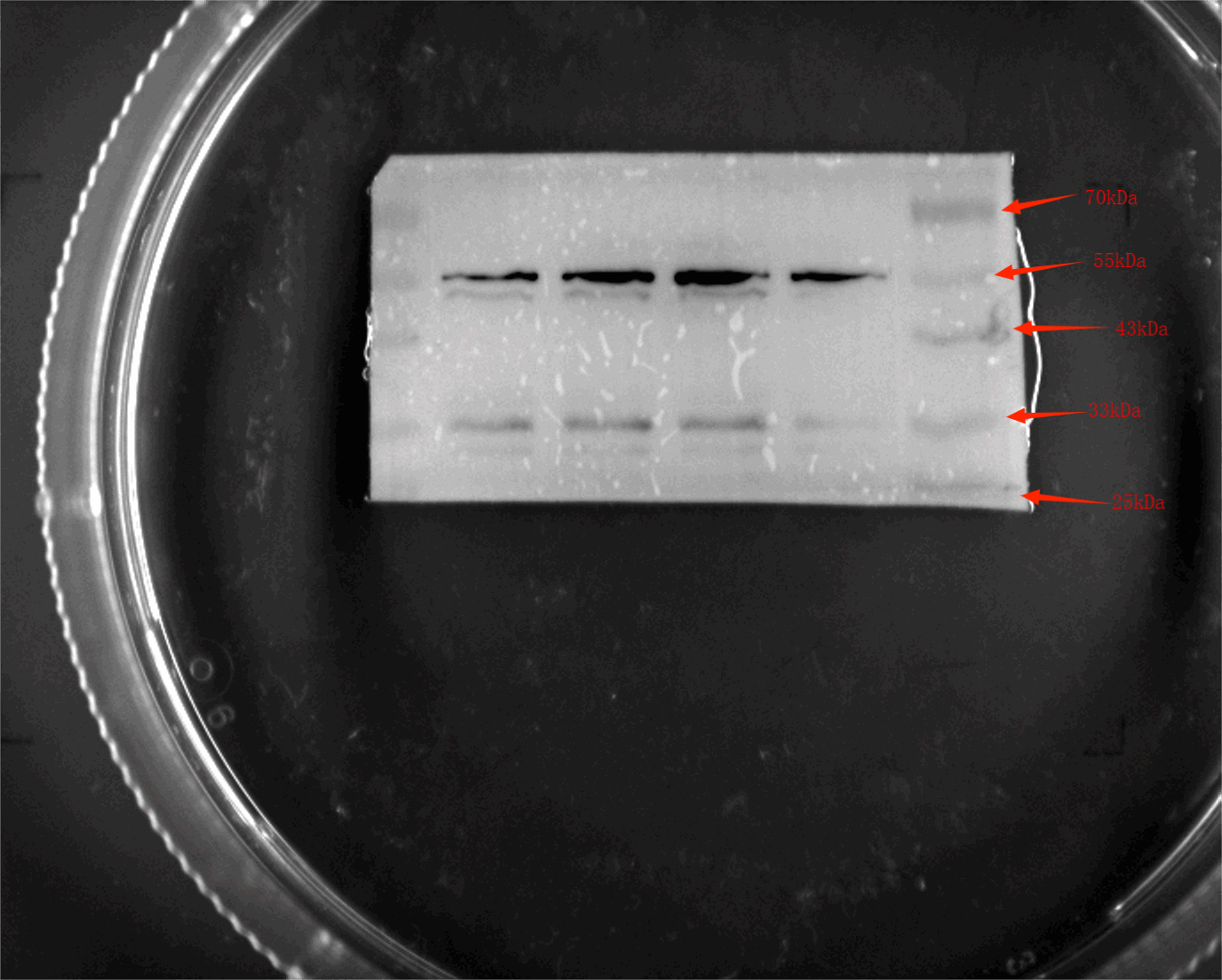

Supplement: Supplemental Information 3 [file peerj-13-19276-s003.zip › western blotting-marke/western blot-free tubulin/6-Free tubulin-M(1).png]

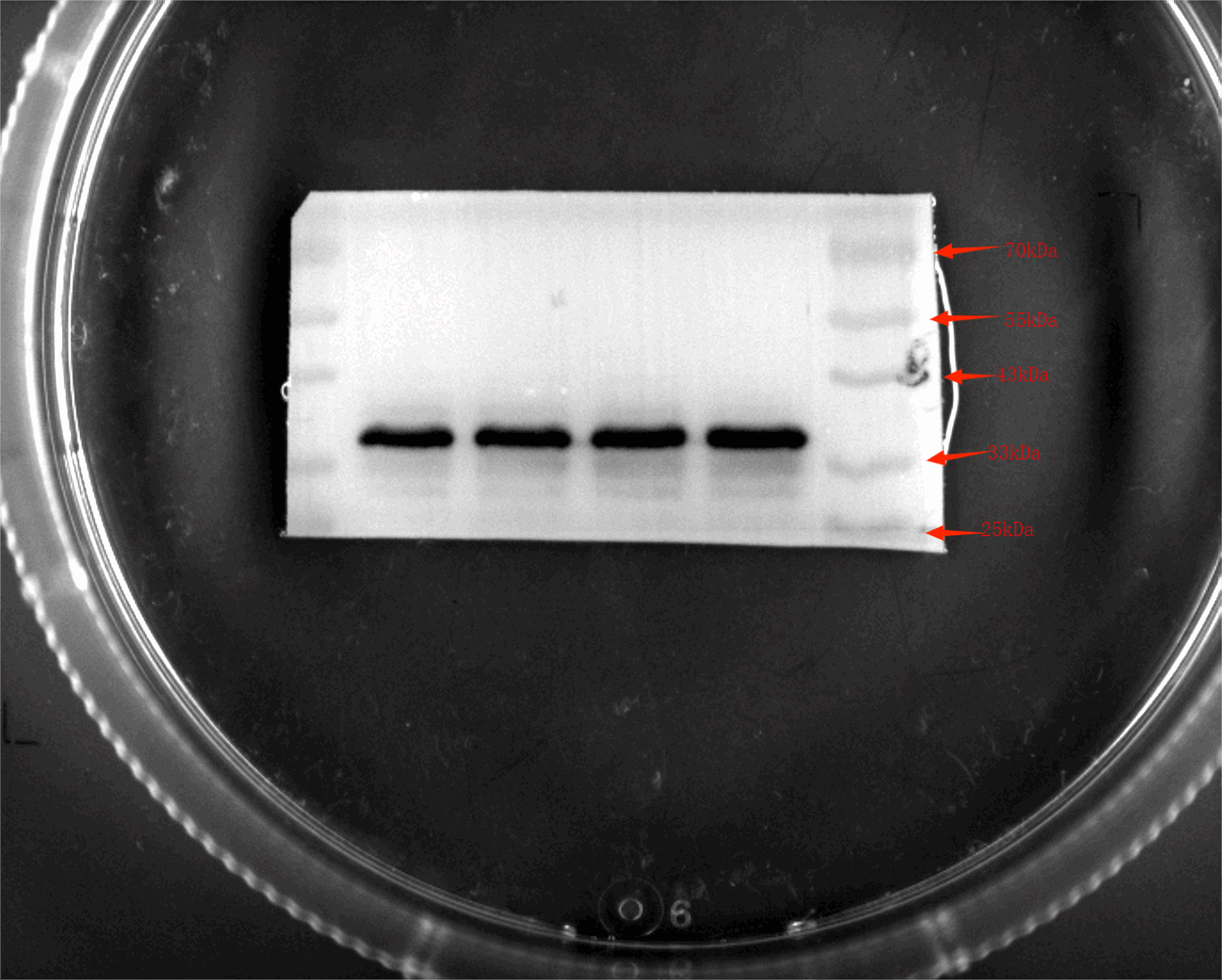

Supplement: Supplemental Information 3 [file peerj-13-19276-s003.zip › western blotting-marke/western blot-free tubulin/6-GAPDH-M(1).png]

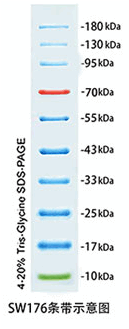

Supplement: Supplemental Information 3 [file peerj-13-19276-s003.zip › western blotting-marke/western blot-free tubulin/Marker reference diagram.png]

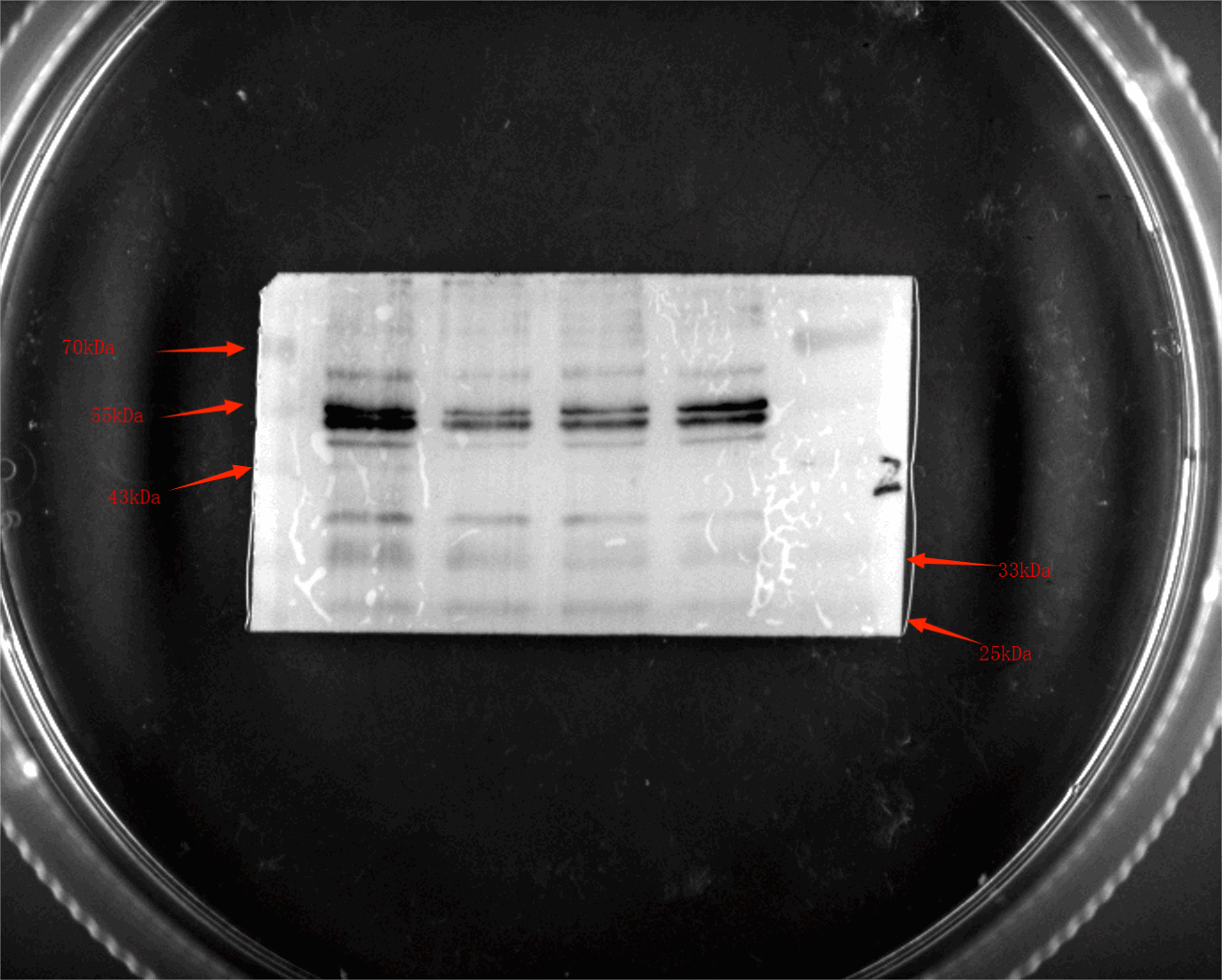

Supplement: Supplemental Information 3 [file peerj-13-19276-s003.zip › western blotting-marke/western blot-Polymeric tubulin/1-Polymeric tubulin-M(1).png]

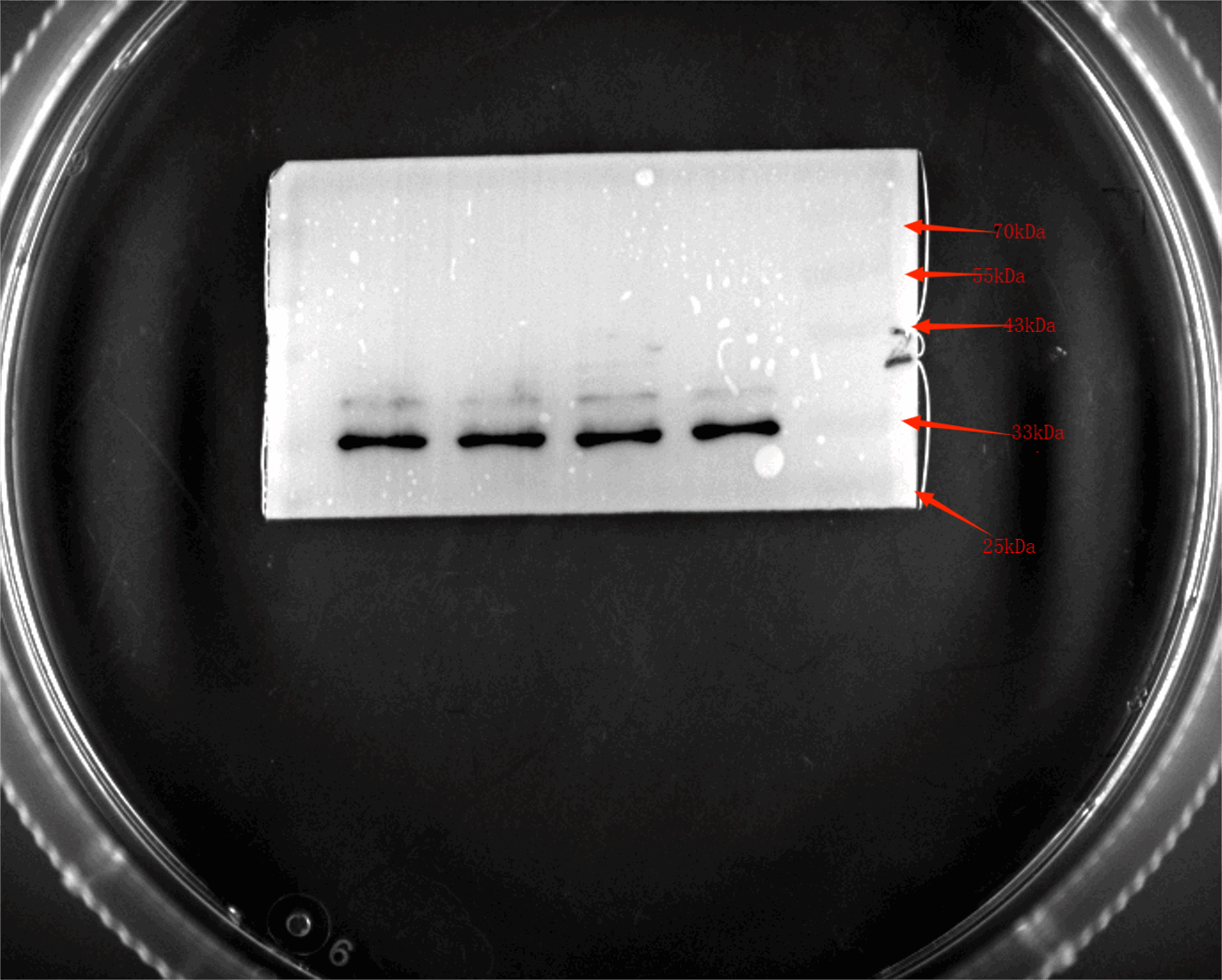

Supplement: Supplemental Information 3 [file peerj-13-19276-s003.zip › western blotting-marke/western blot-Polymeric tubulin/1-VDAC-M(1).png]

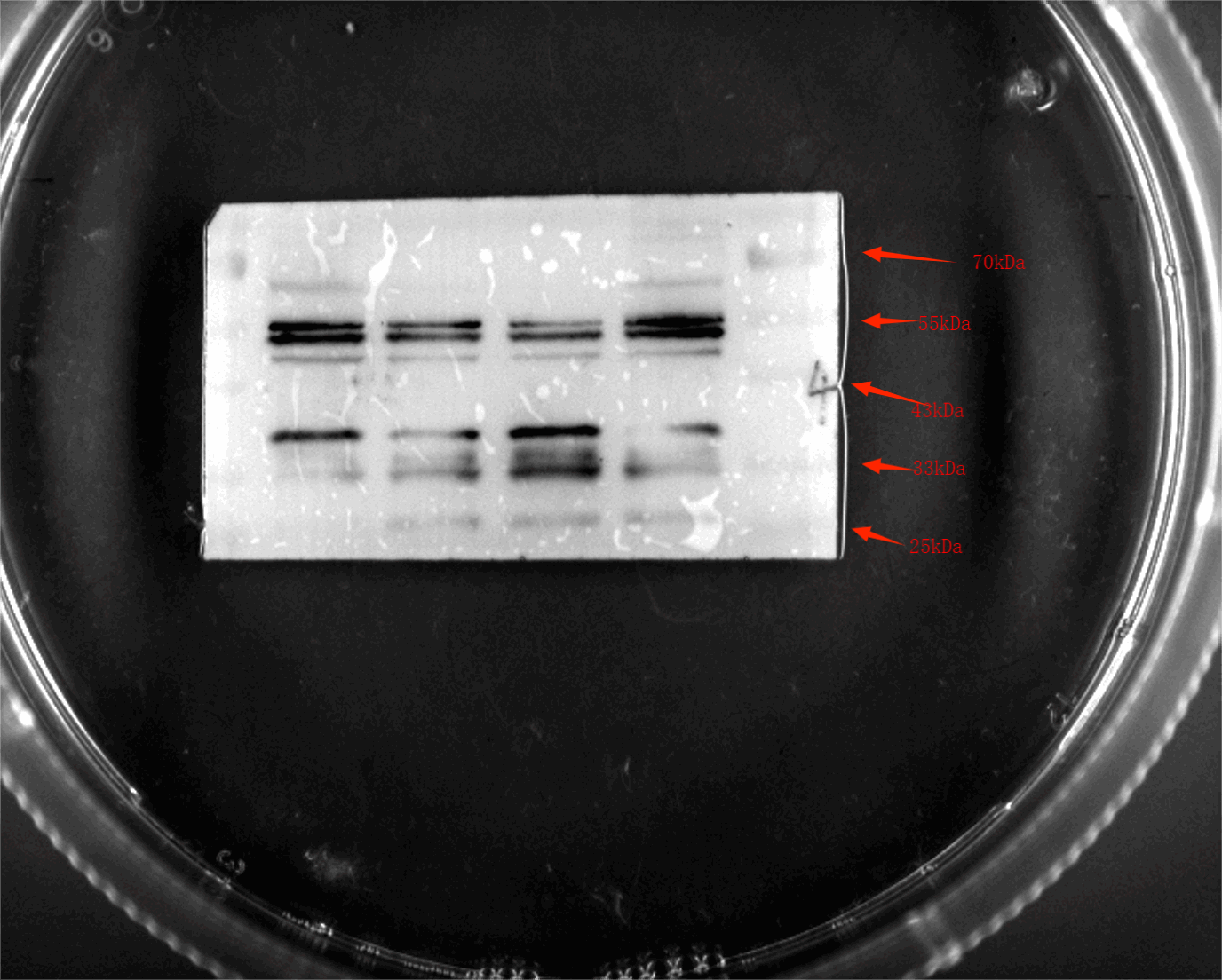

Supplement: Supplemental Information 3 [file peerj-13-19276-s003.zip › western blotting-marke/western blot-Polymeric tubulin/2-Polymeric tubulin-M(1).png]

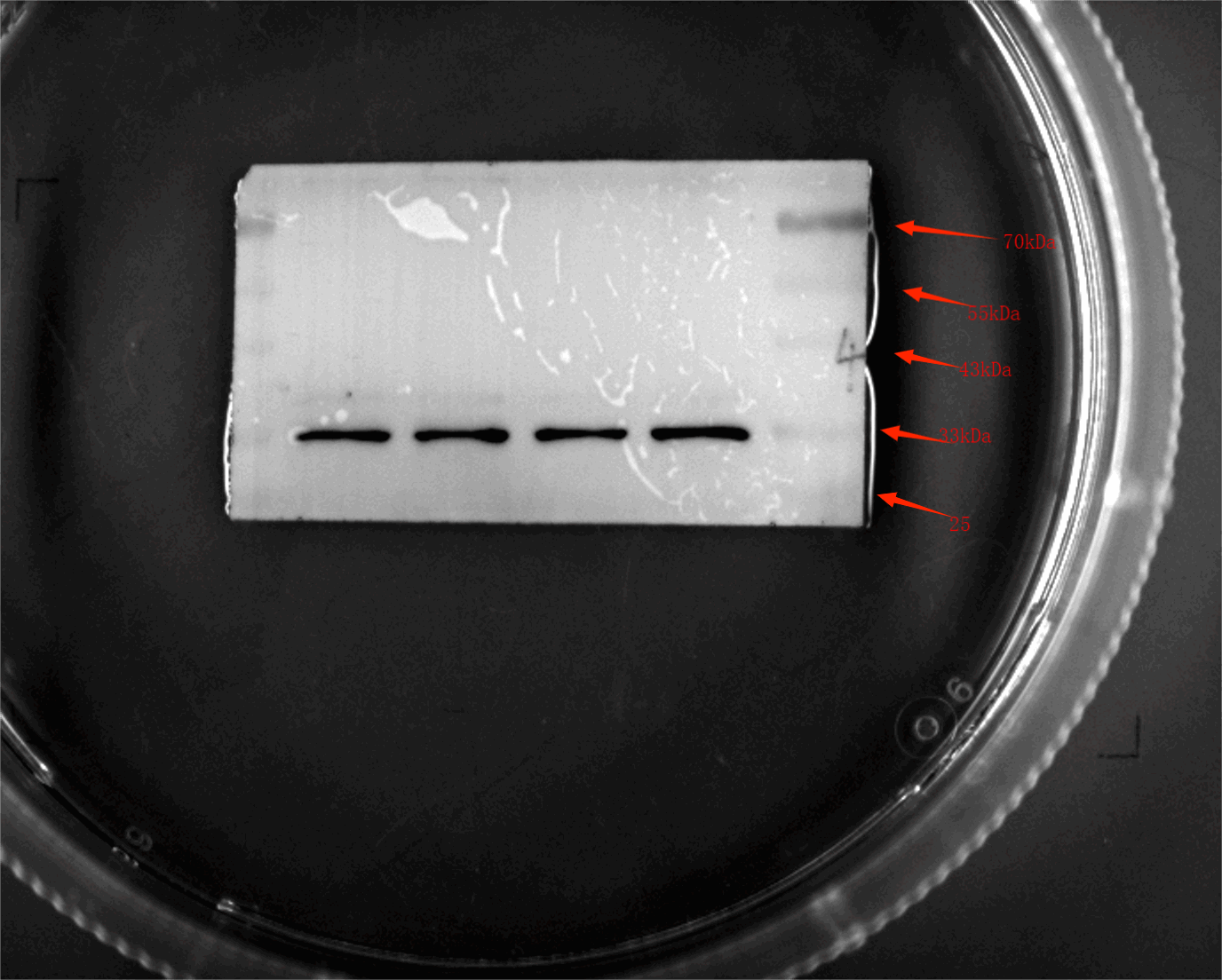

Supplement: Supplemental Information 3 [file peerj-13-19276-s003.zip › western blotting-marke/western blot-Polymeric tubulin/2-VDAC-M(1).png]

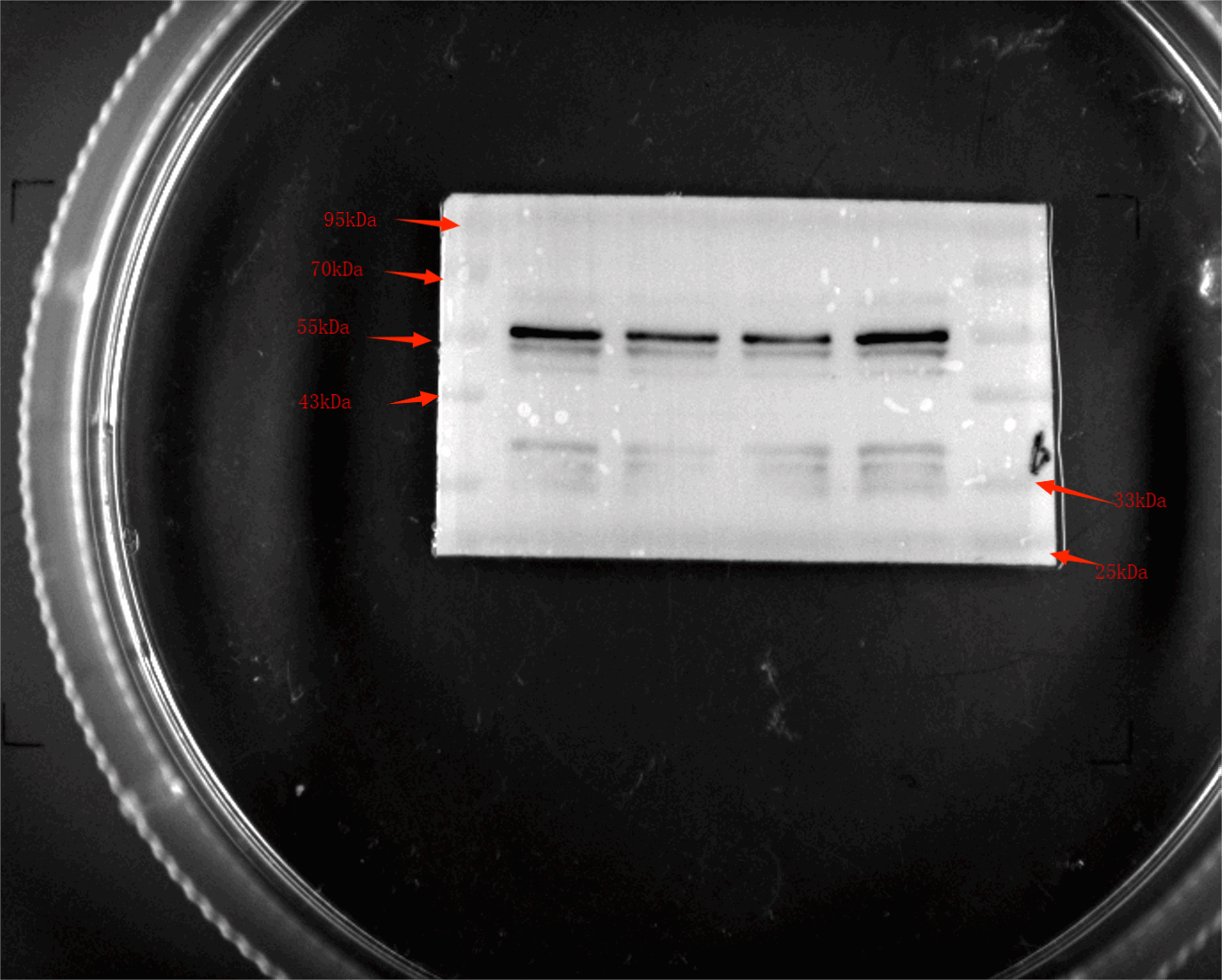

Supplement: Supplemental Information 3 [file peerj-13-19276-s003.zip › western blotting-marke/western blot-Polymeric tubulin/3-Polymeric tubulin-M-used(1).png]

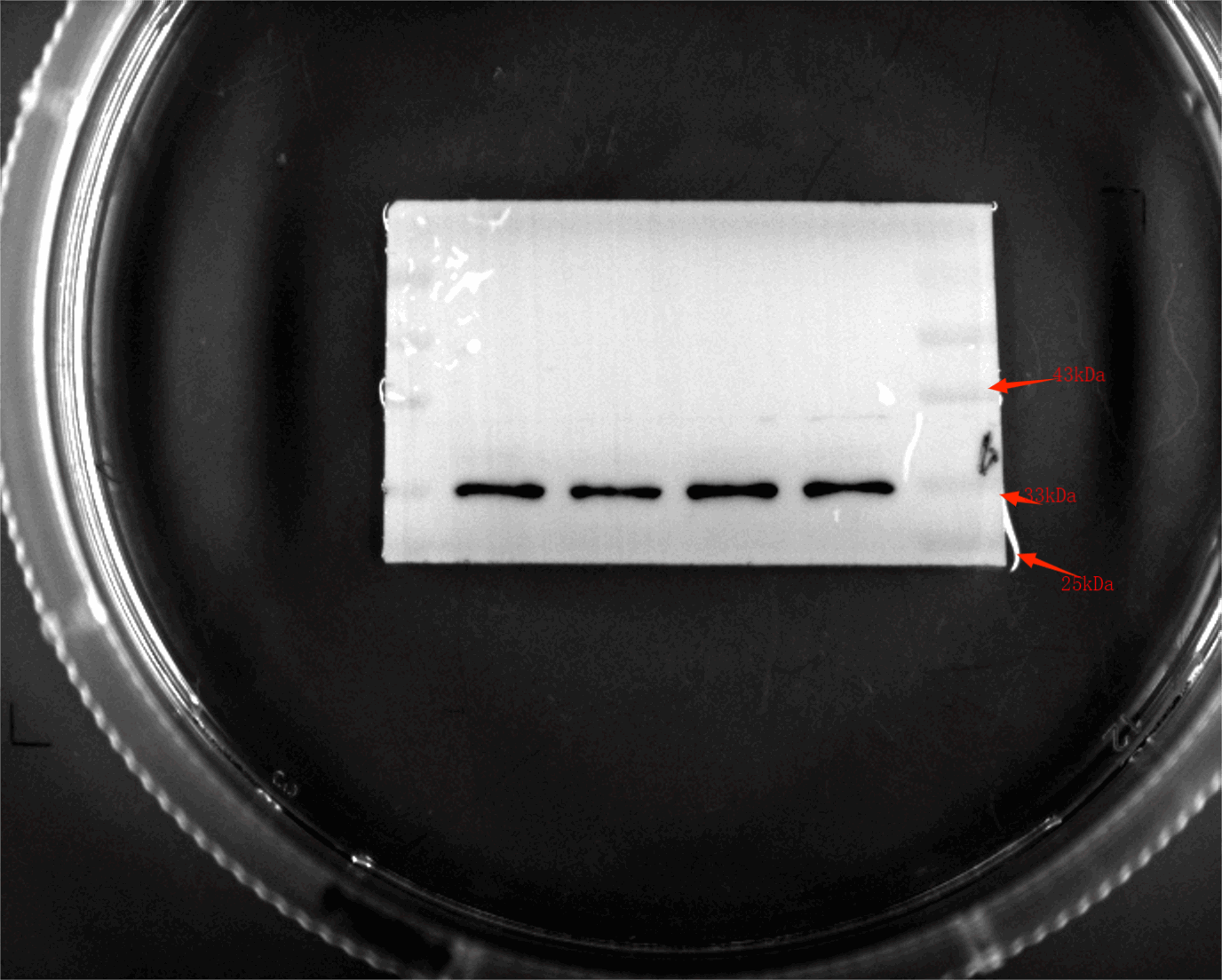

Supplement: Supplemental Information 3 [file peerj-13-19276-s003.zip › western blotting-marke/western blot-Polymeric tubulin/3-VDAC-M-used(1).png]

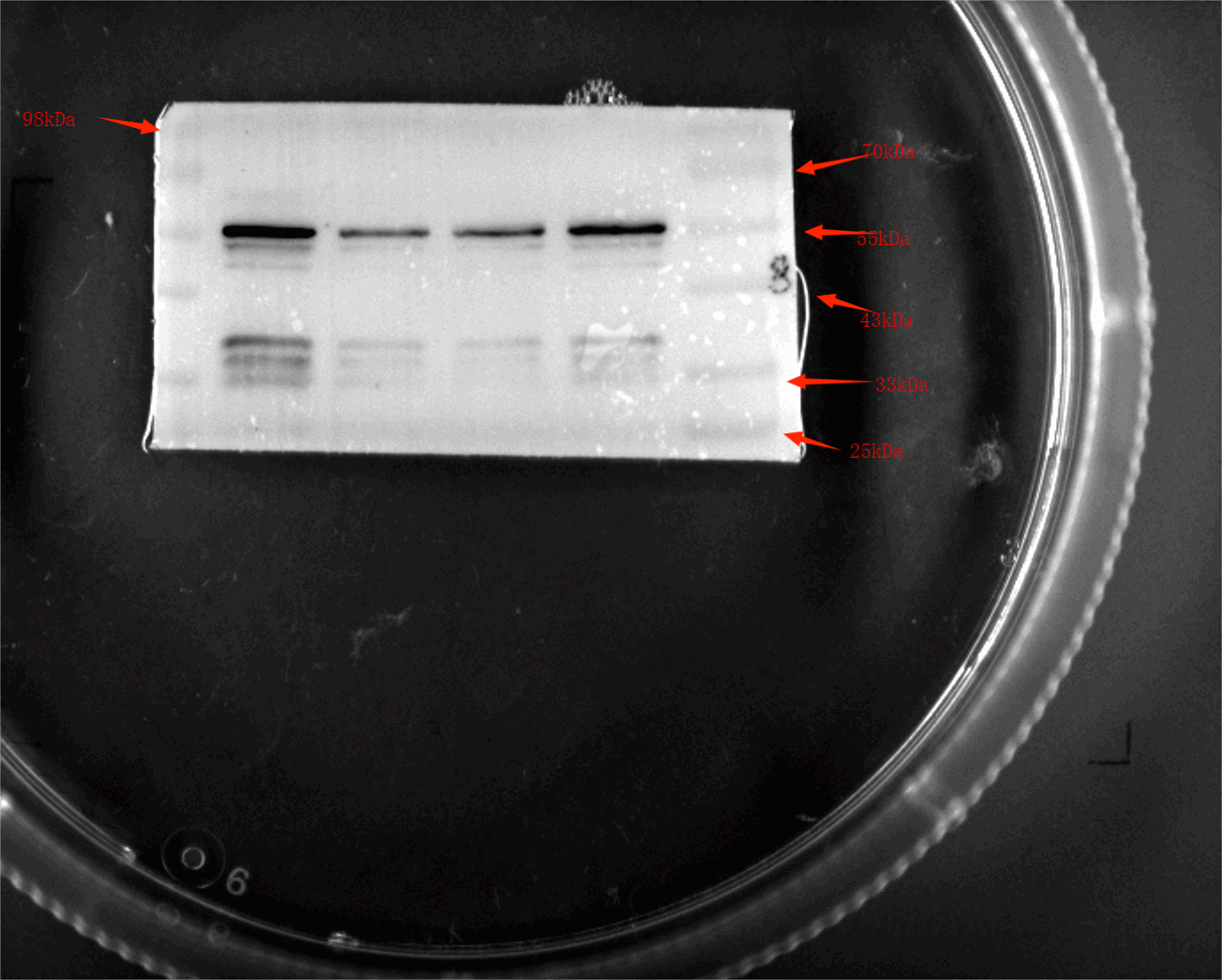

Supplement: Supplemental Information 3 [file peerj-13-19276-s003.zip › western blotting-marke/western blot-Polymeric tubulin/4-Polymeric tubulin-M(1).png]

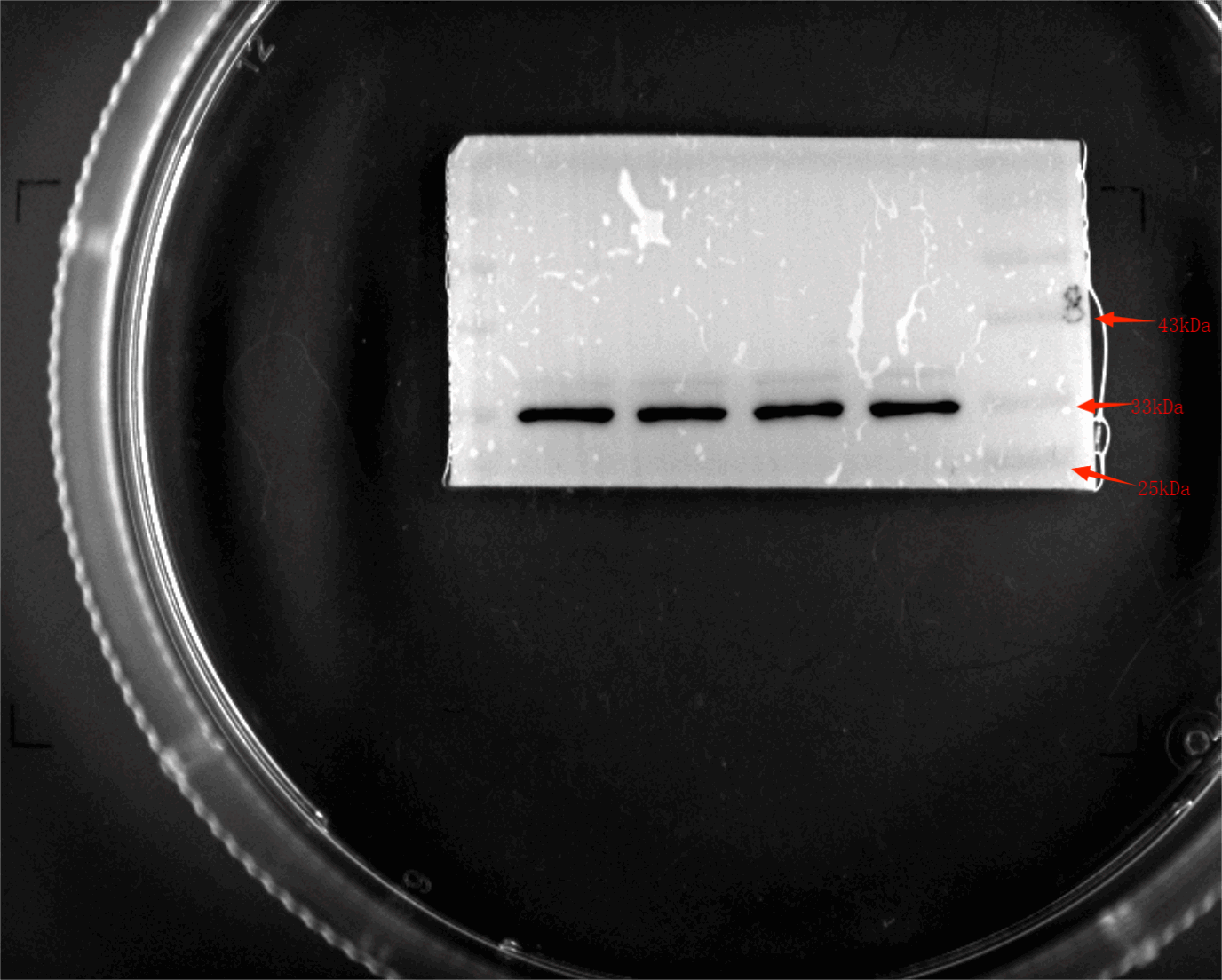

Supplement: Supplemental Information 3 [file peerj-13-19276-s003.zip › western blotting-marke/western blot-Polymeric tubulin/4-VDAC-M(1).png]

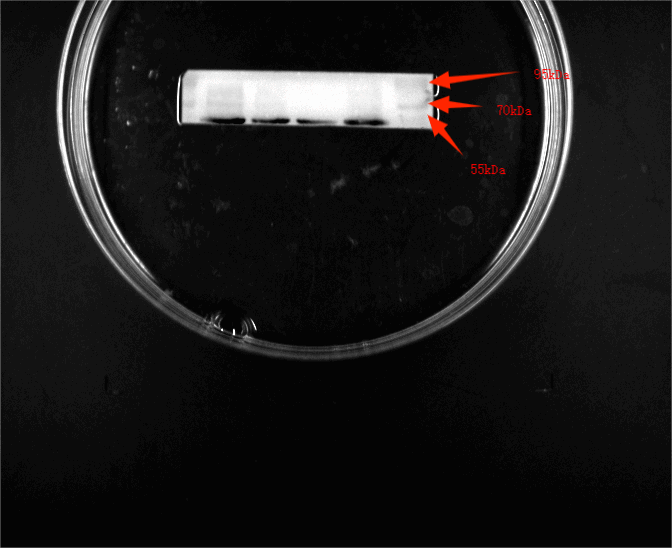

Supplement: Supplemental Information 3 [file peerj-13-19276-s003.zip › western blotting-marke/western blot-Polymeric tubulin/5-Polymeric tubulin-M(1).png]

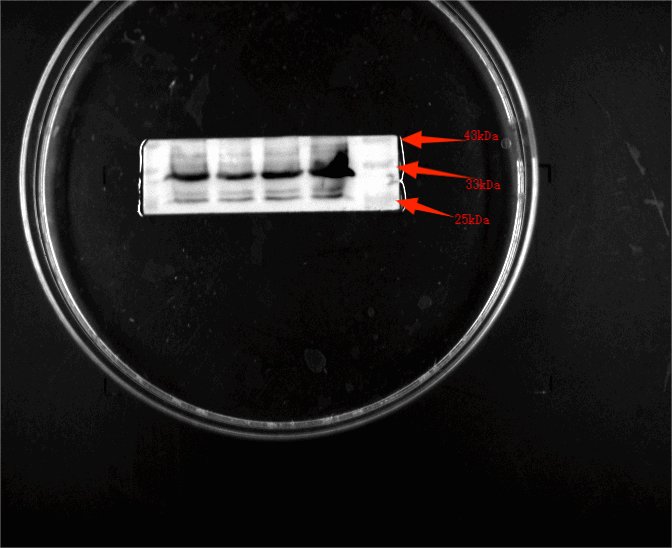

Supplement: Supplemental Information 3 [file peerj-13-19276-s003.zip › western blotting-marke/western blot-Polymeric tubulin/5-VDAC-M(1).png]

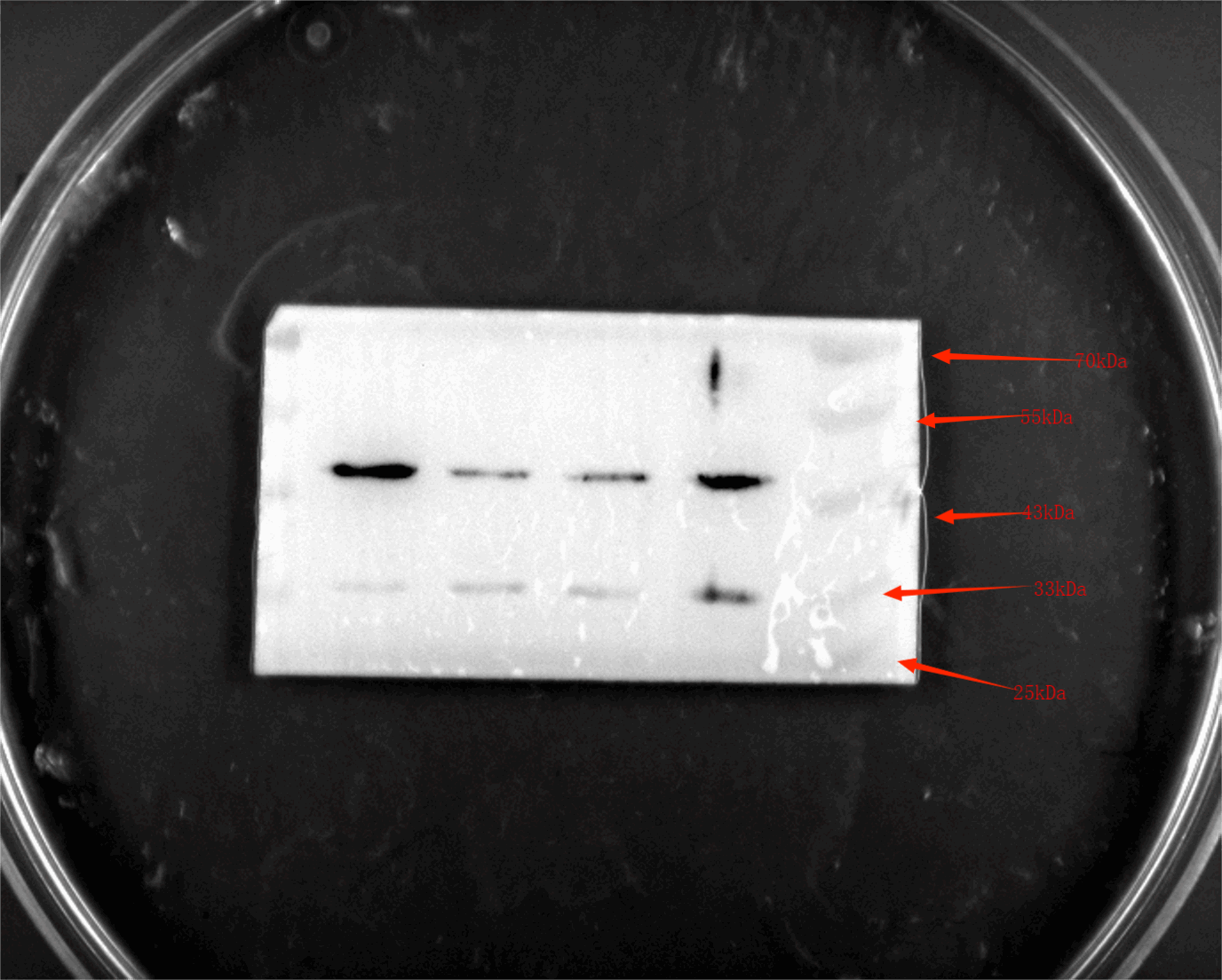

Supplement: Supplemental Information 3 [file peerj-13-19276-s003.zip › western blotting-marke/western blot-Polymeric tubulin/6-Polymeric tubulin-M(1).png]

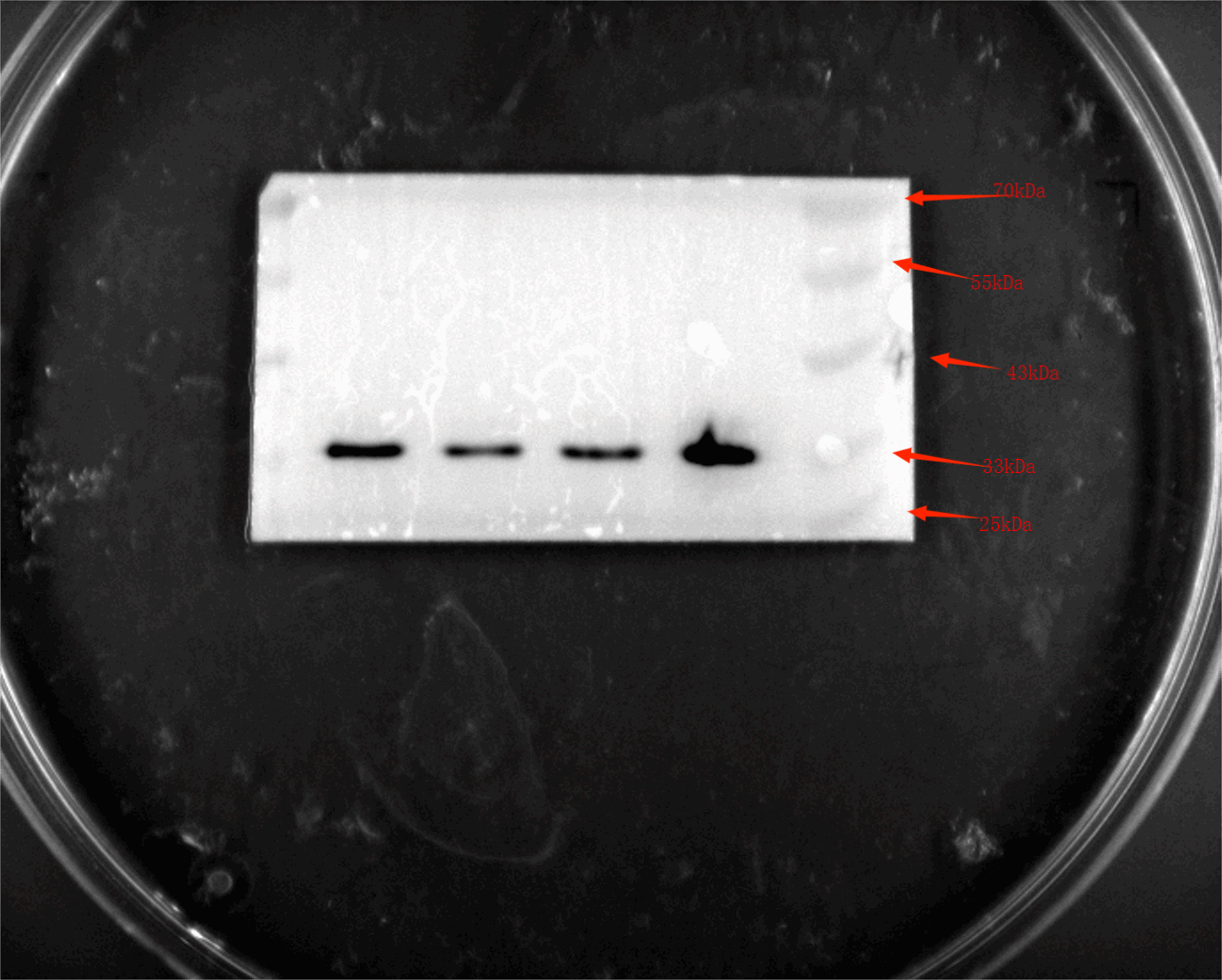

Supplement: Supplemental Information 3 [file peerj-13-19276-s003.zip › western blotting-marke/western blot-Polymeric tubulin/6-VDAC-M(1).png]

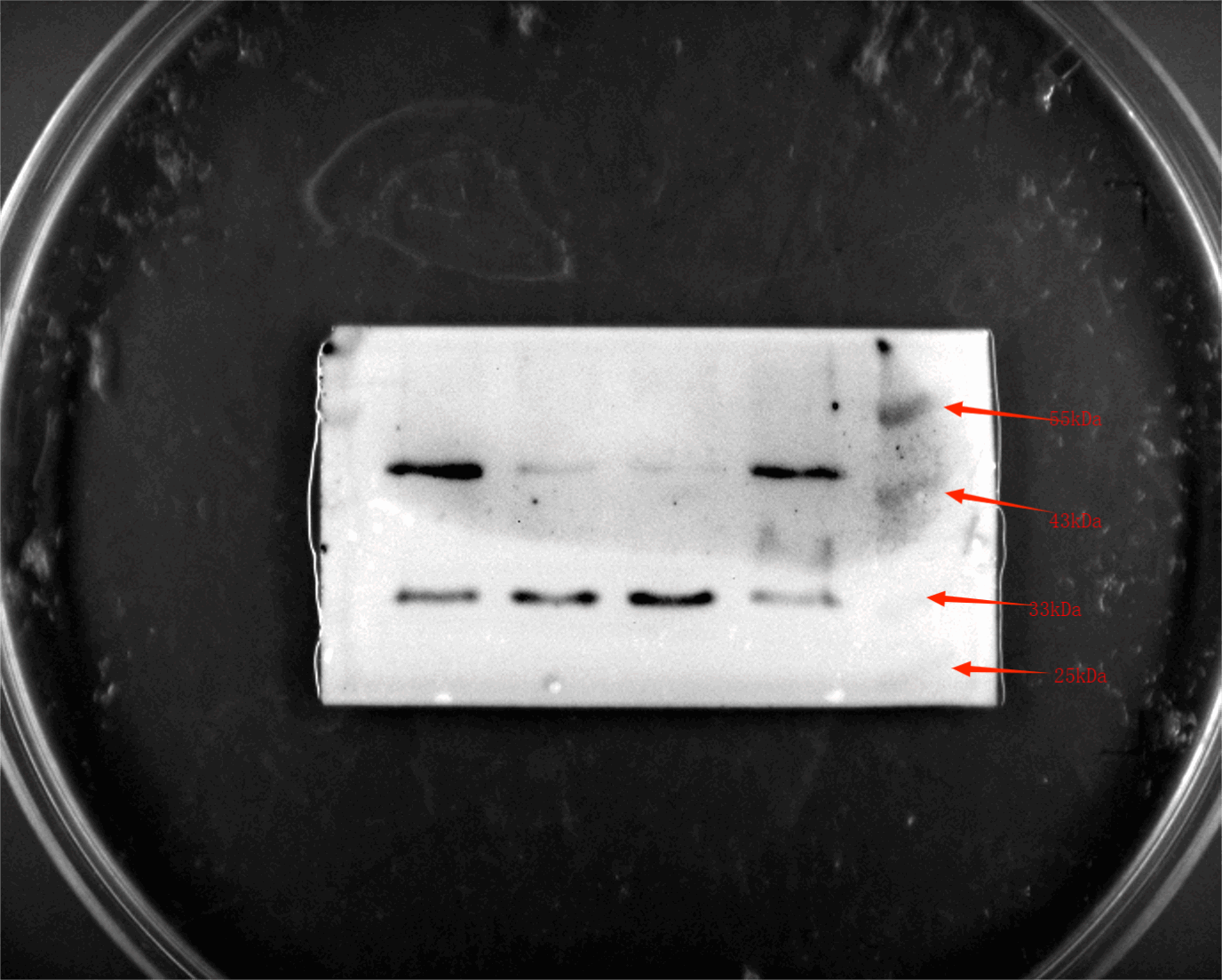

Supplement: Supplemental Information 3 [file peerj-13-19276-s003.zip › western blotting-marke/western blot-Polymeric tubulin/7-Polymeric tubulin-M(1).png]

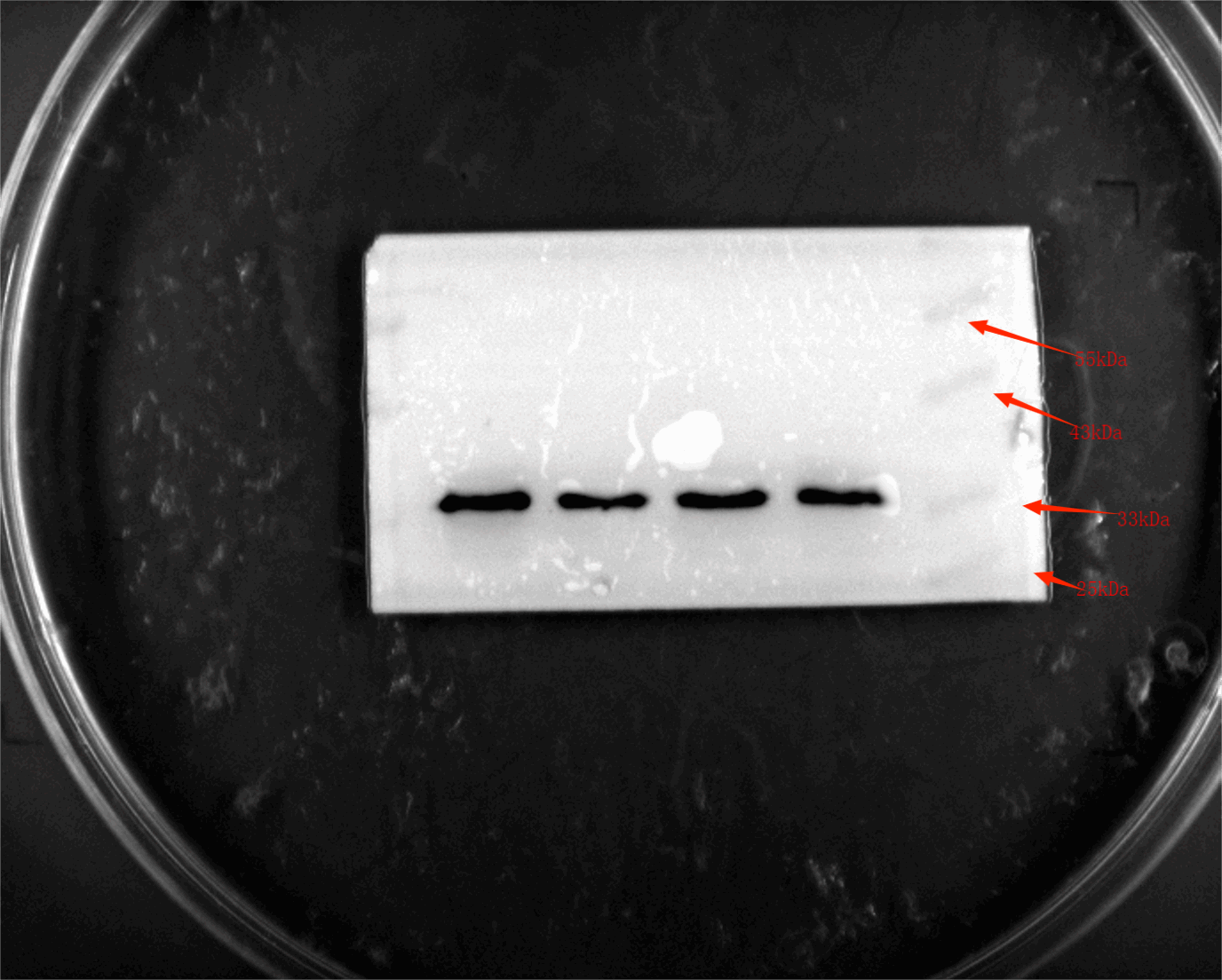

Supplement: Supplemental Information 3 [file peerj-13-19276-s003.zip › western blotting-marke/western blot-Polymeric tubulin/7-VDAC-M(1).png]

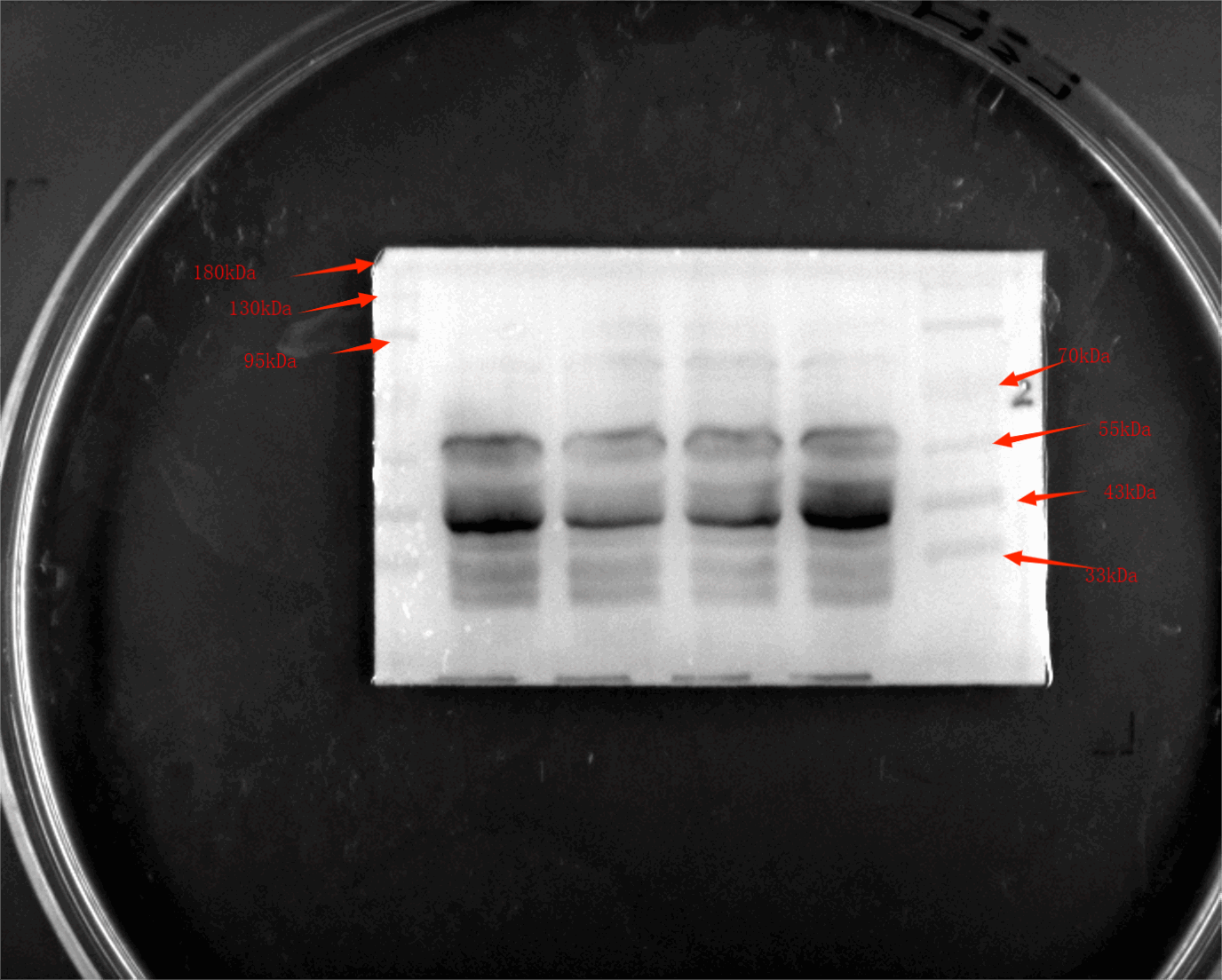

Supplement: Supplemental Information 3 [file peerj-13-19276-s003.zip › western blotting-marke/western blot-Total Cx43 EB1 N-cadherin/1-CX43-M(1).png]

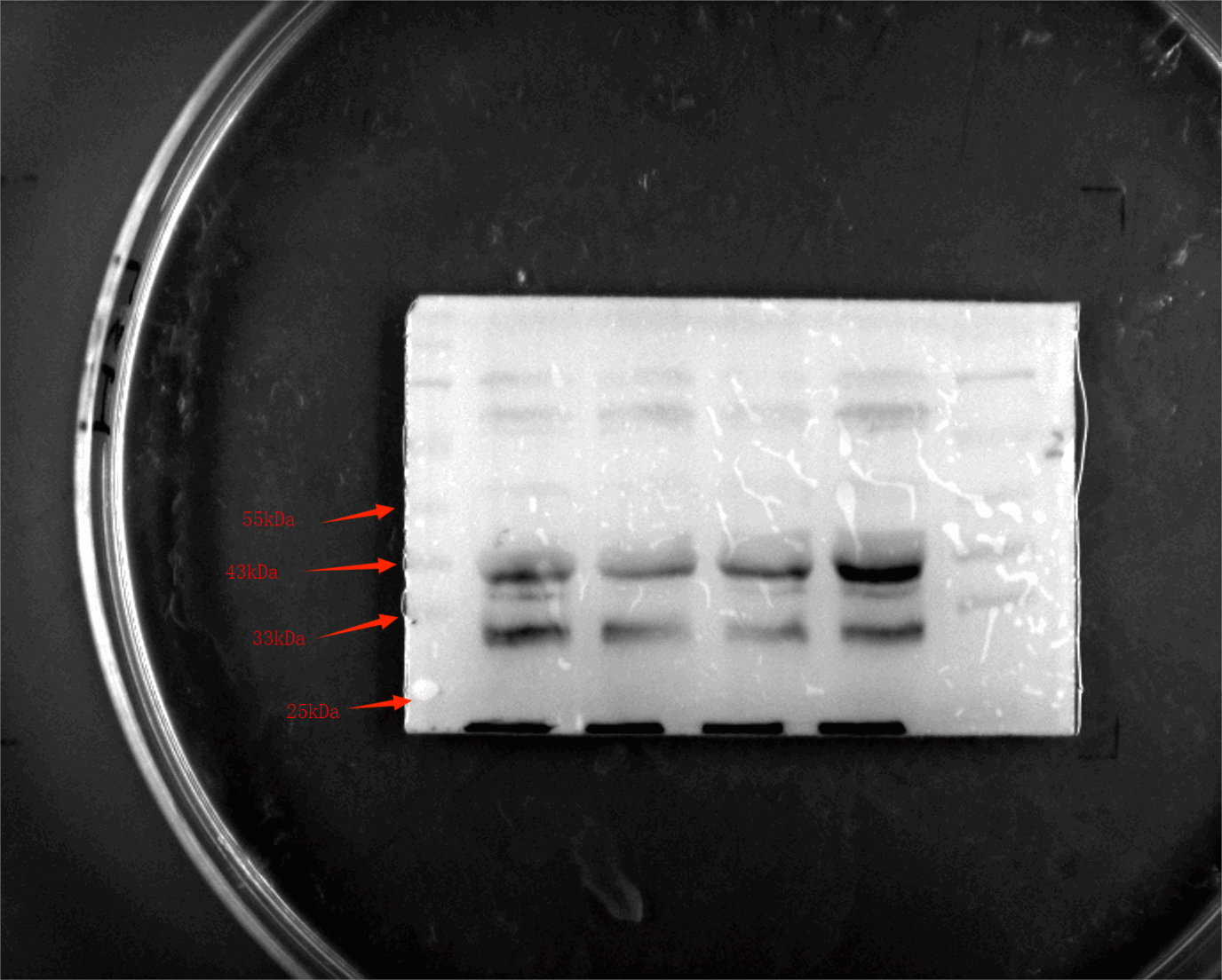

Supplement: Supplemental Information 3 [file peerj-13-19276-s003.zip › western blotting-marke/western blot-Total Cx43 EB1 N-cadherin/1-EB1-M(1).png]

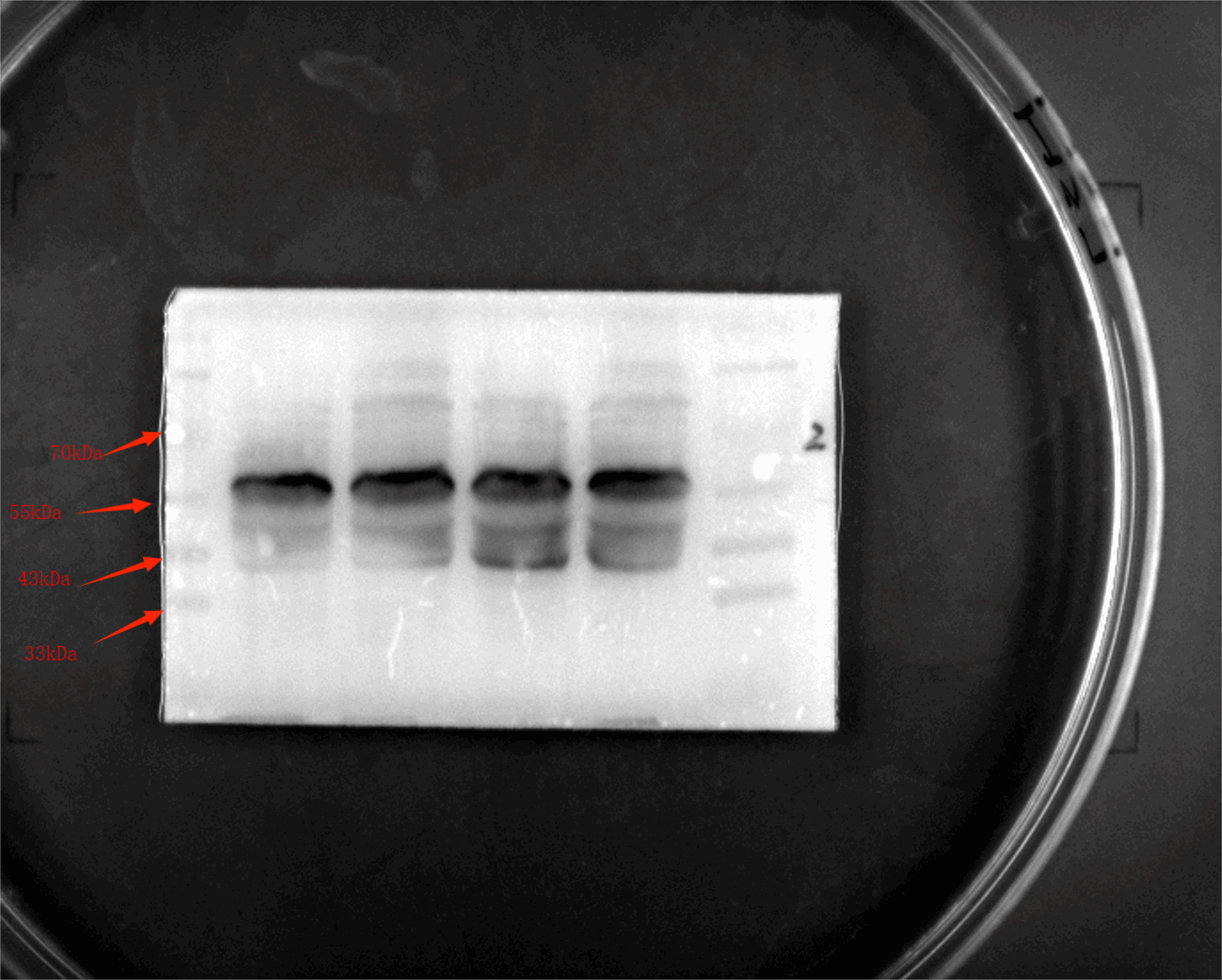

Supplement: Supplemental Information 3 [file peerj-13-19276-s003.zip › western blotting-marke/western blot-Total Cx43 EB1 N-cadherin/1-Tubulin-M(1).png]

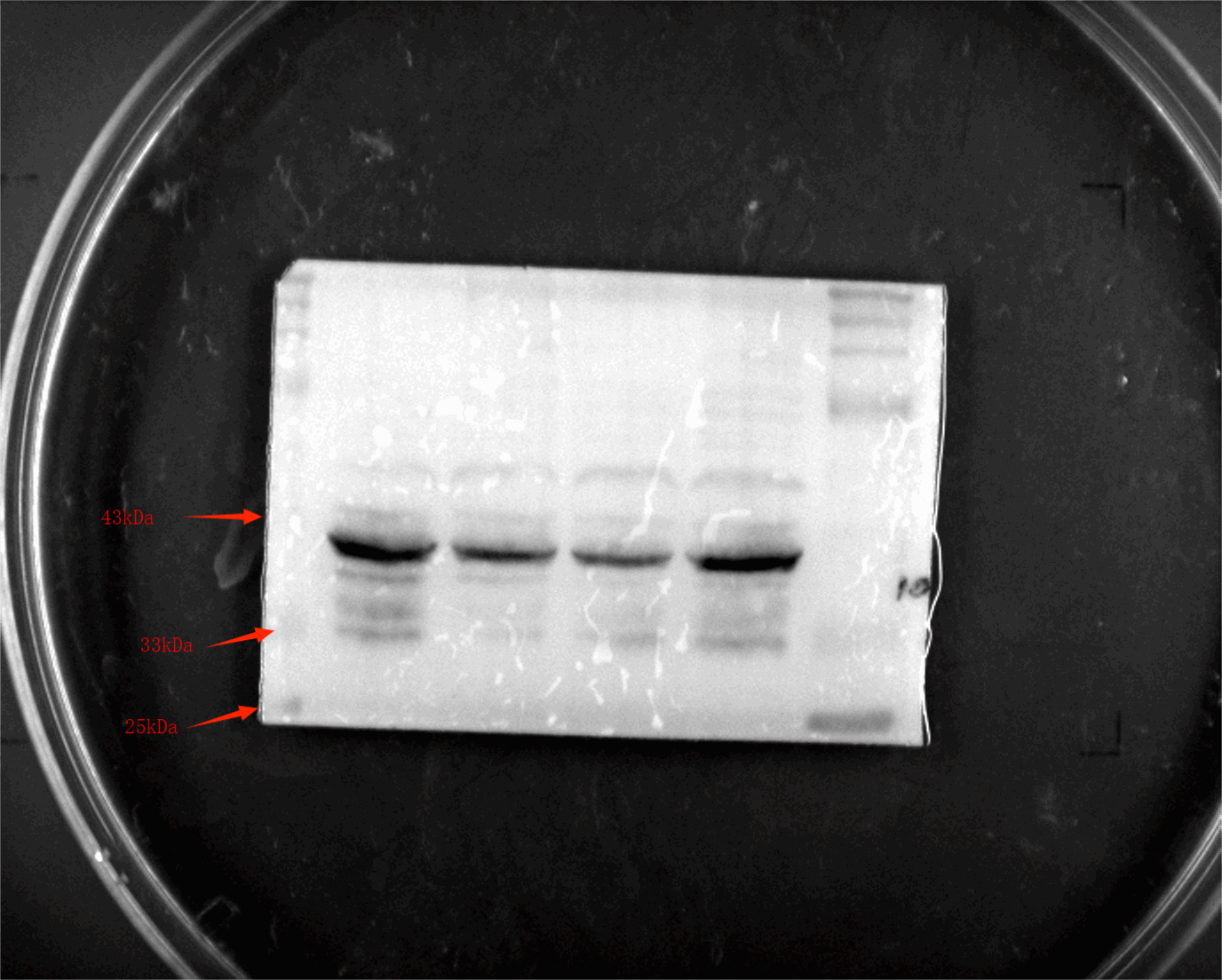

Supplement: Supplemental Information 3 [file peerj-13-19276-s003.zip › western blotting-marke/western blot-Total Cx43 EB1 N-cadherin/10-cx43-M-used(1).png]

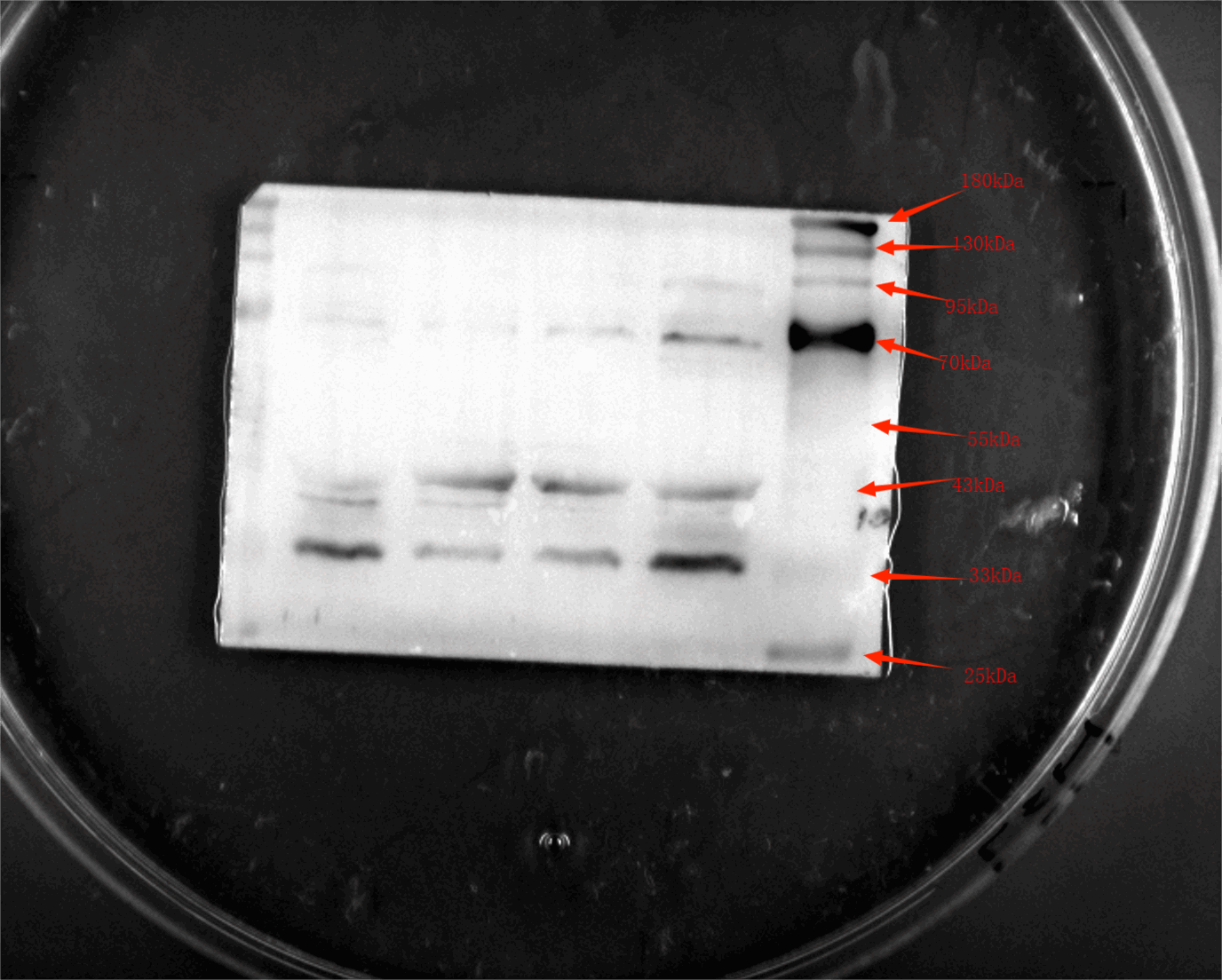

Supplement: Supplemental Information 3 [file peerj-13-19276-s003.zip › western blotting-marke/western blot-Total Cx43 EB1 N-cadherin/10-EB1-M-used(1).png]

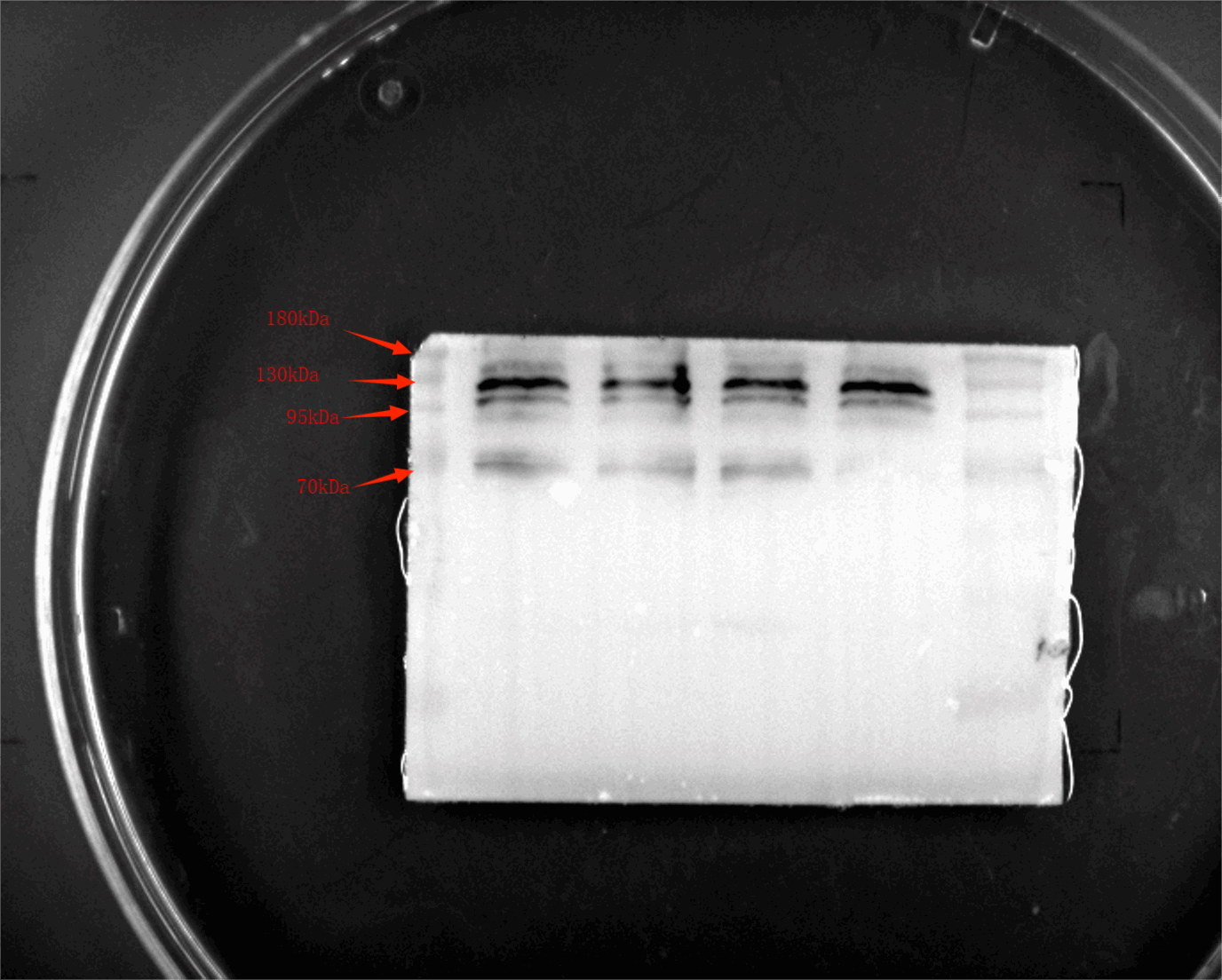

Supplement: Supplemental Information 3 [file peerj-13-19276-s003.zip › western blotting-marke/western blot-Total Cx43 EB1 N-cadherin/10-N-cadherin-M(1).png]

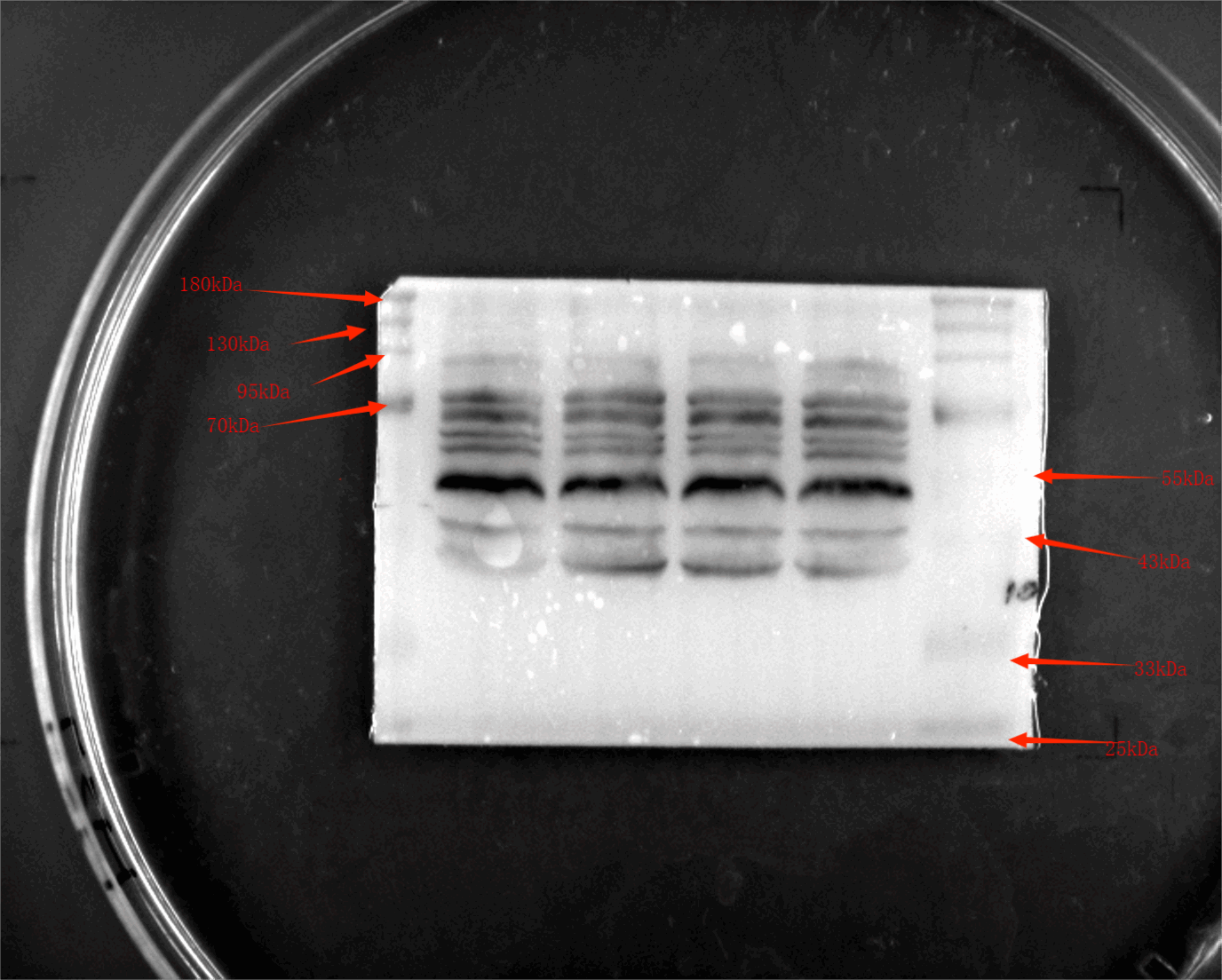

Supplement: Supplemental Information 3 [file peerj-13-19276-s003.zip › western blotting-marke/western blot-Total Cx43 EB1 N-cadherin/10-Tubulin-M-used(1).png]

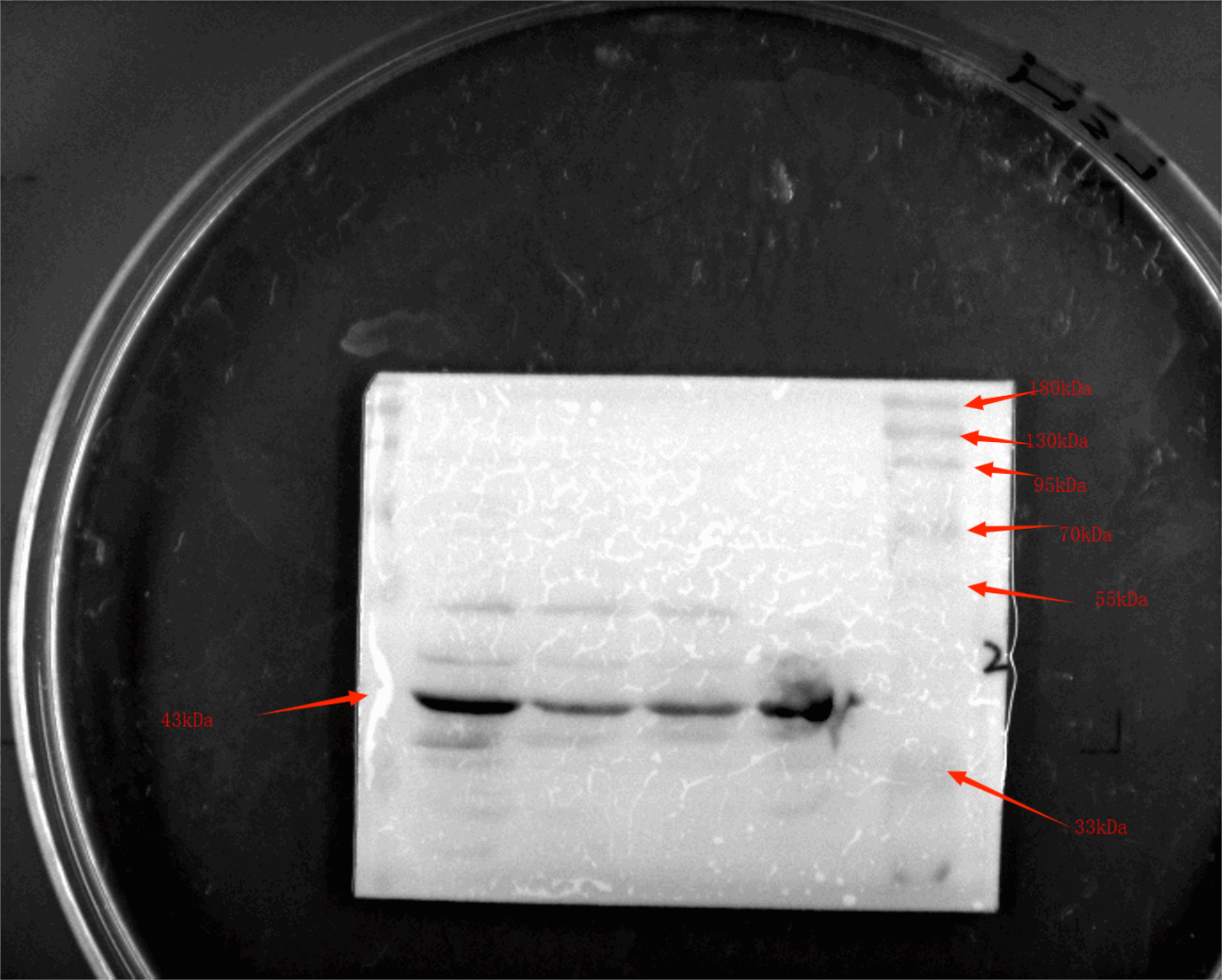

Supplement: Supplemental Information 3 [file peerj-13-19276-s003.zip › western blotting-marke/western blot-Total Cx43 EB1 N-cadherin/2-cx43-M(1).png]

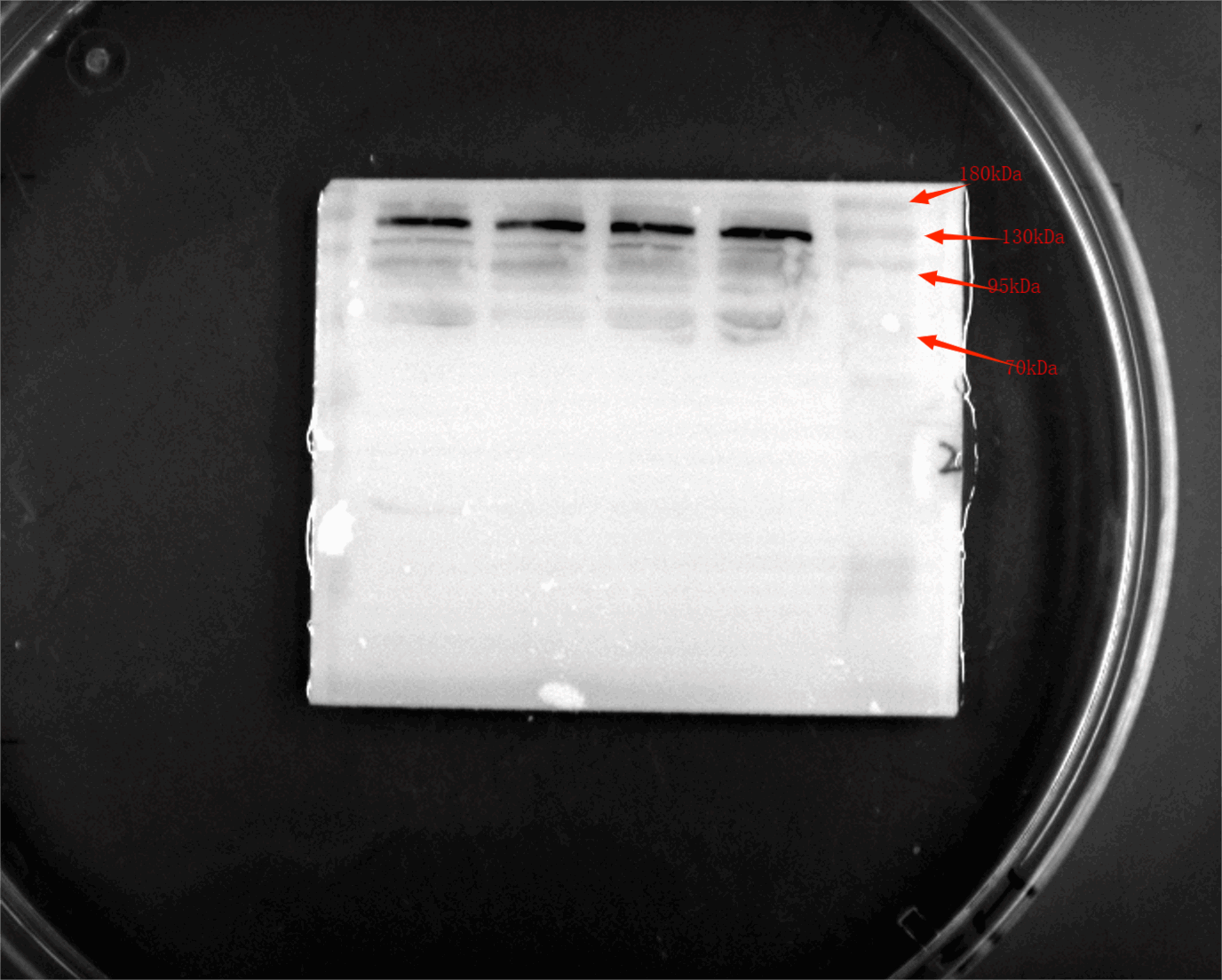

Supplement: Supplemental Information 3 [file peerj-13-19276-s003.zip › western blotting-marke/western blot-Total Cx43 EB1 N-cadherin/2-N-cadherin-M(1).png]

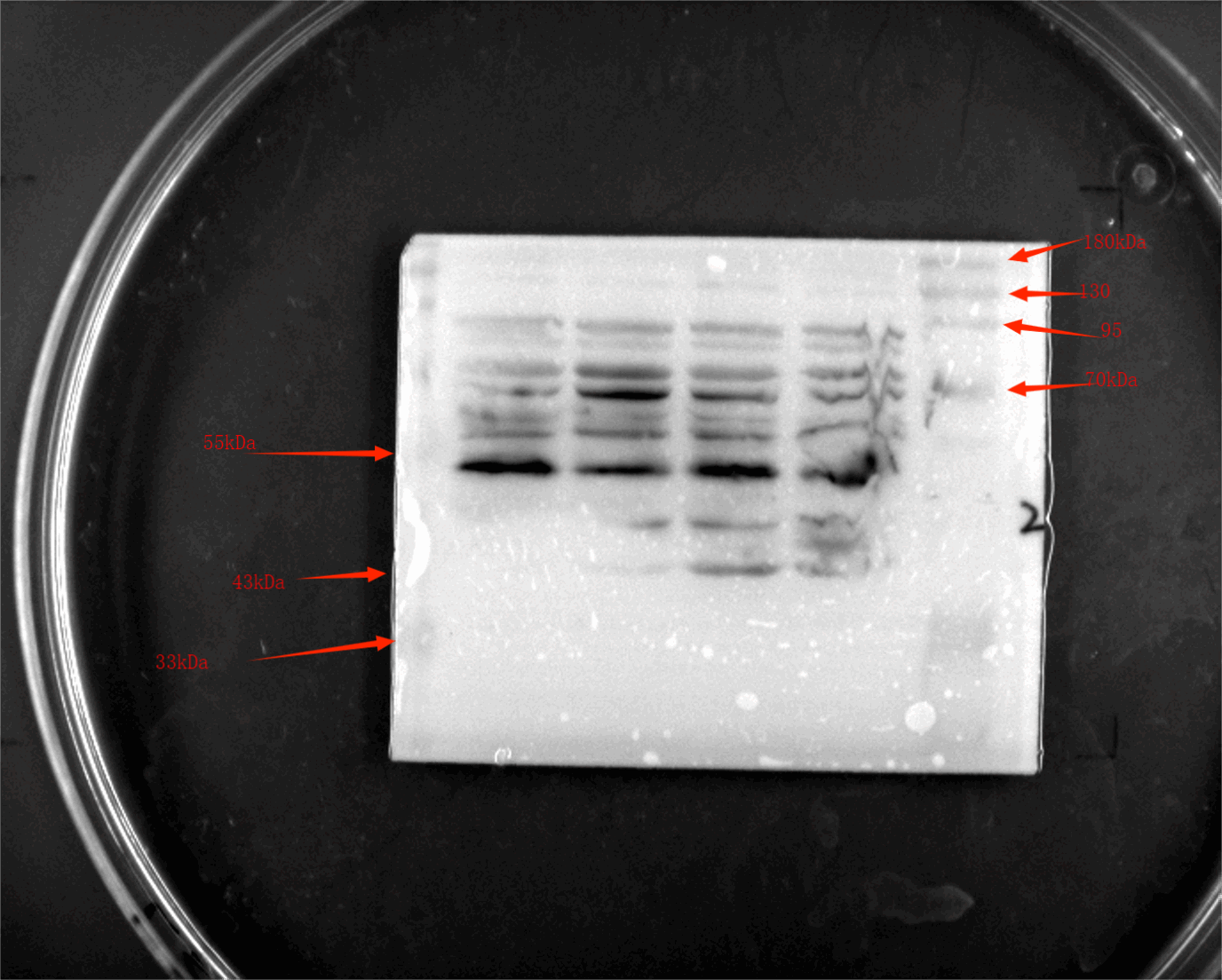

Supplement: Supplemental Information 3 [file peerj-13-19276-s003.zip › western blotting-marke/western blot-Total Cx43 EB1 N-cadherin/2-Tubulin-M(1).png]

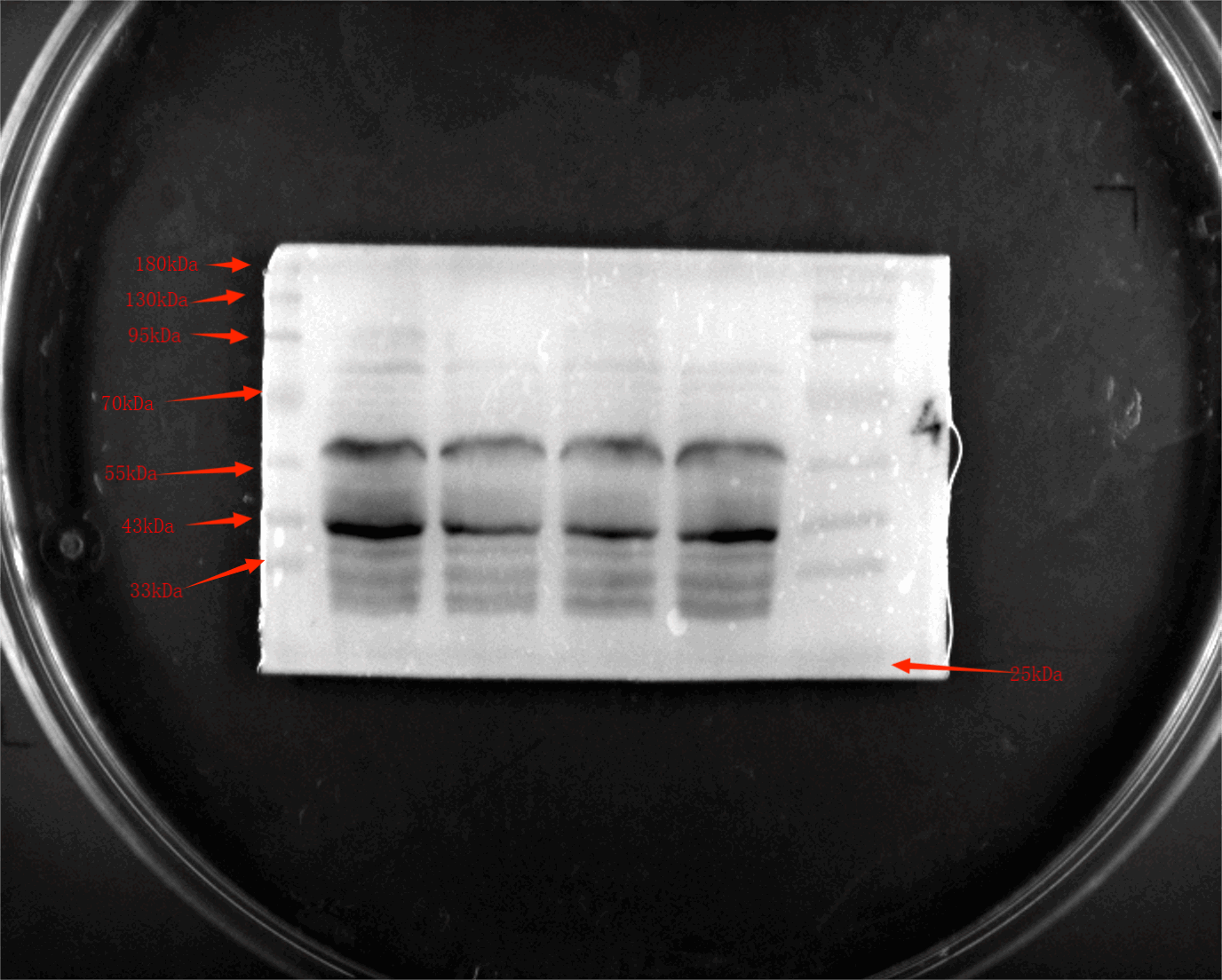

Supplement: Supplemental Information 3 [file peerj-13-19276-s003.zip › western blotting-marke/western blot-Total Cx43 EB1 N-cadherin/3-CX43-M(1).png]

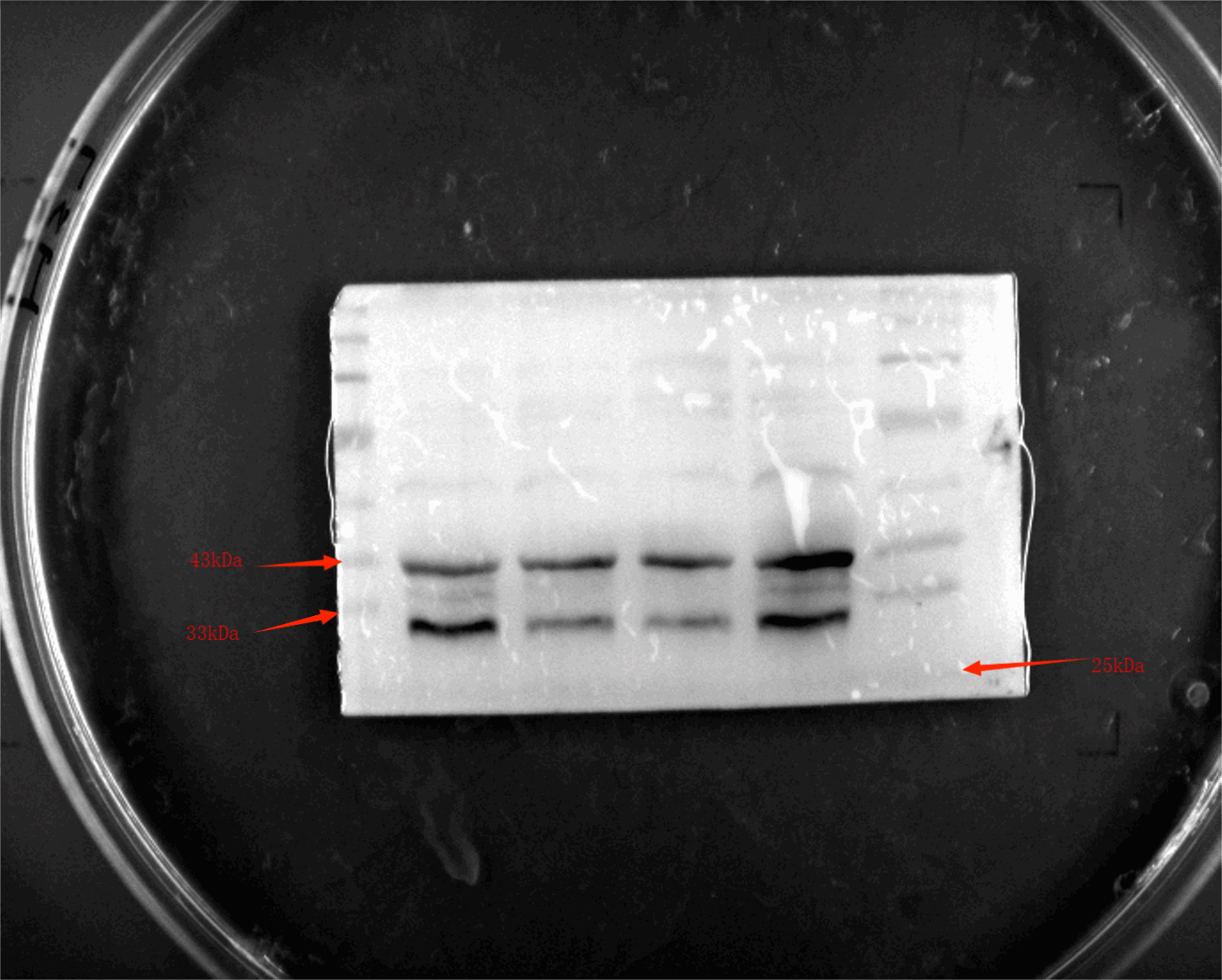

Supplement: Supplemental Information 3 [file peerj-13-19276-s003.zip › western blotting-marke/western blot-Total Cx43 EB1 N-cadherin/3-EB1-M(1).png]

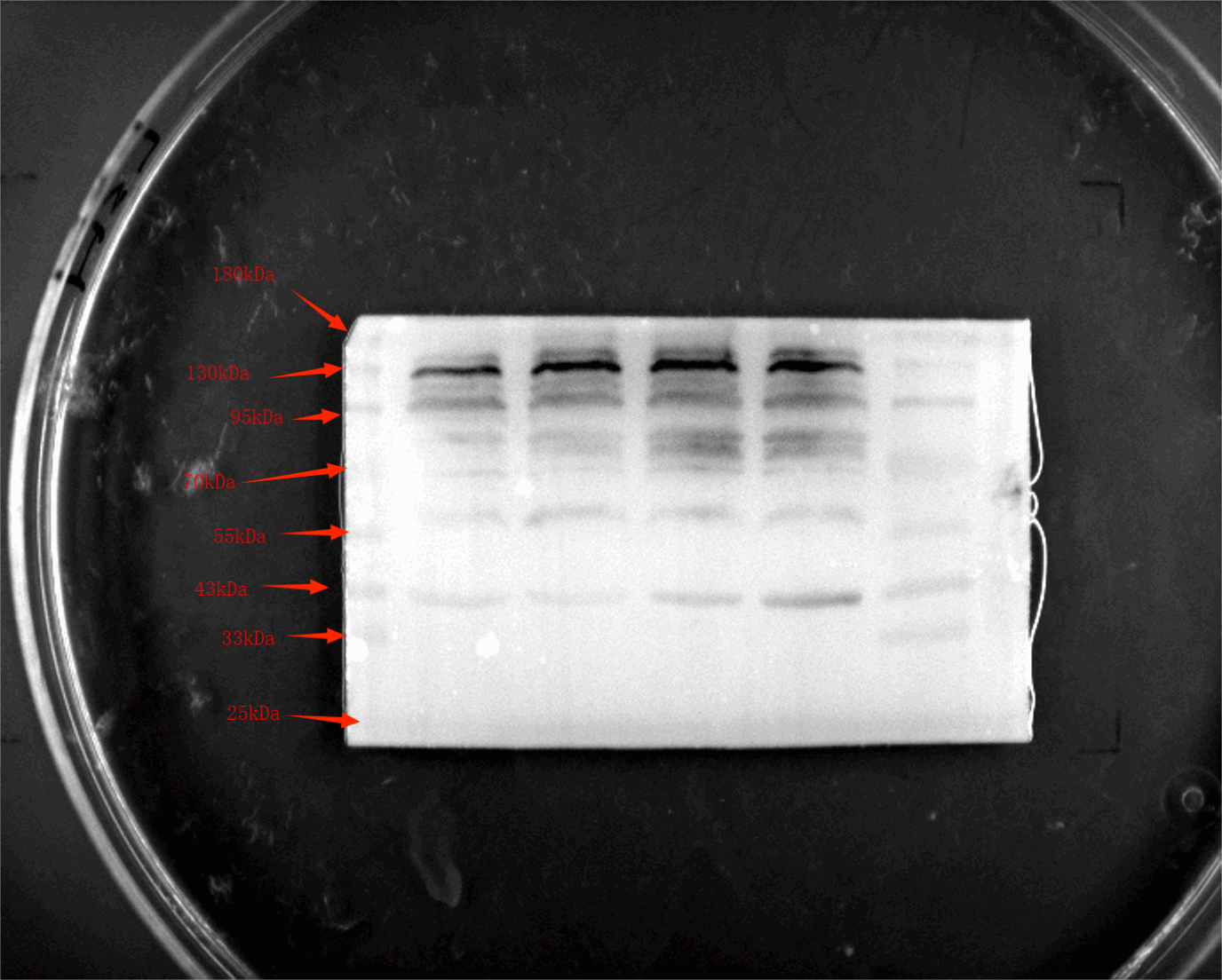

Supplement: Supplemental Information 3 [file peerj-13-19276-s003.zip › western blotting-marke/western blot-Total Cx43 EB1 N-cadherin/3-N-cadherin-M(1).png]

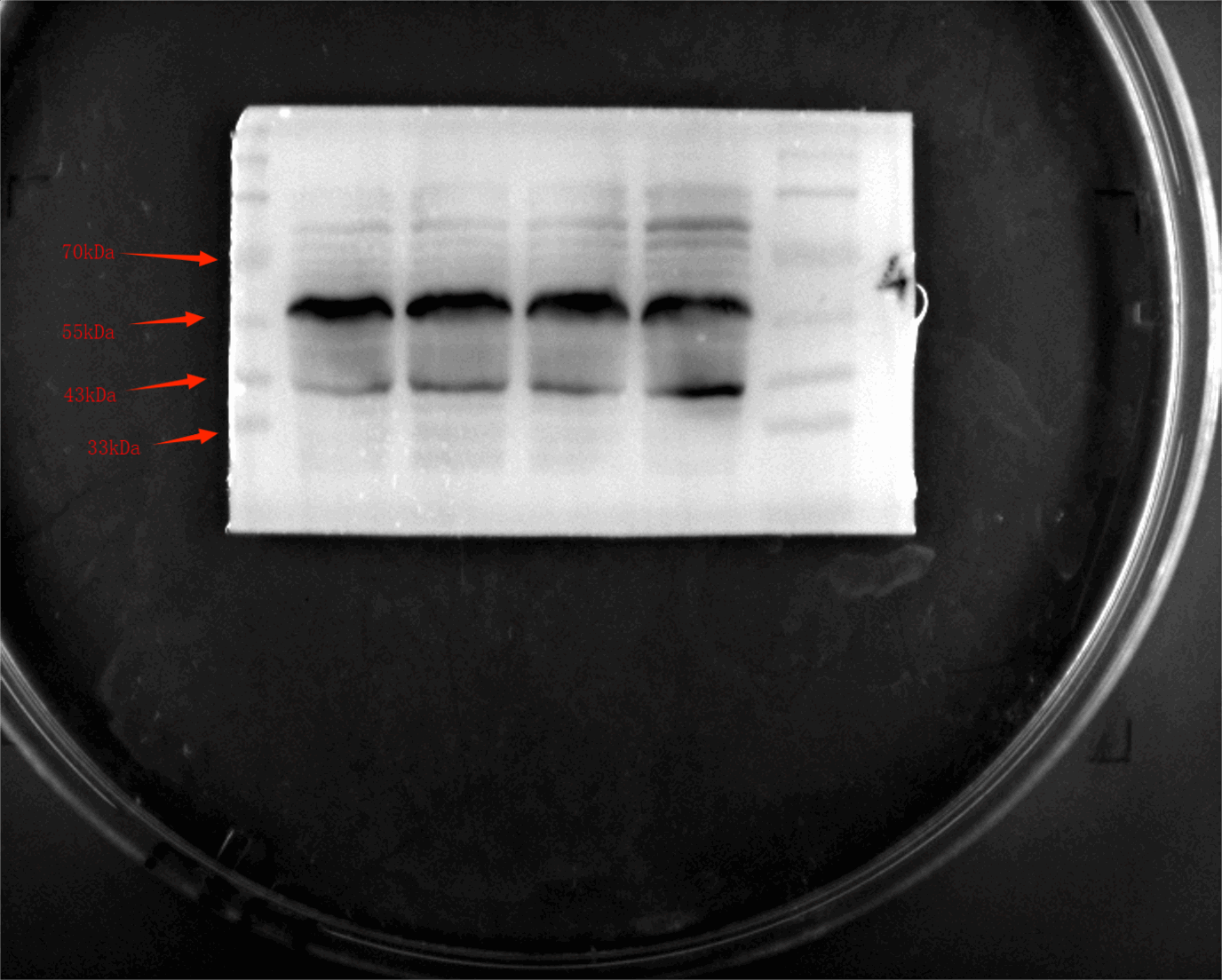

Supplement: Supplemental Information 3 [file peerj-13-19276-s003.zip › western blotting-marke/western blot-Total Cx43 EB1 N-cadherin/3-Tubulin-M(1).png]

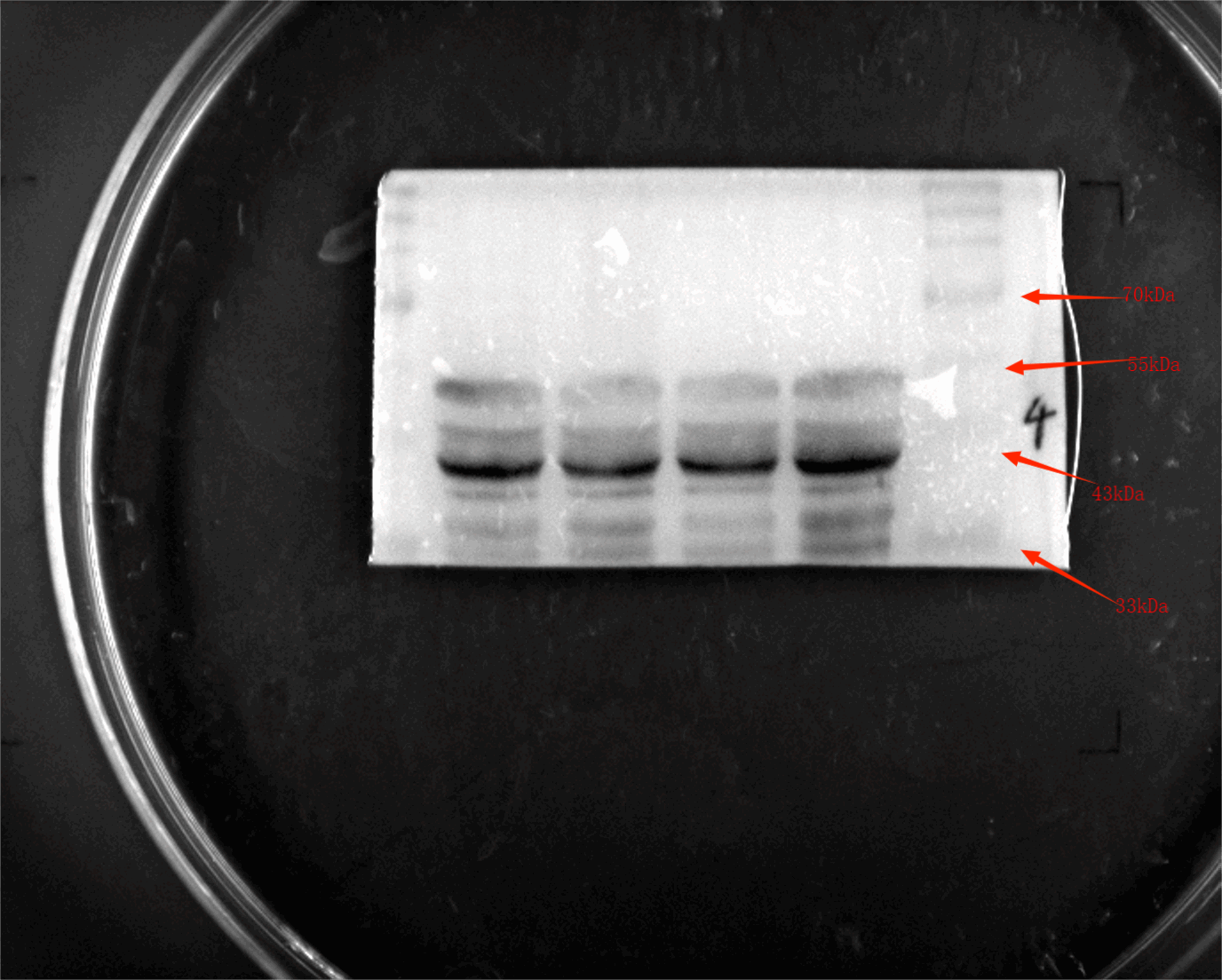

Supplement: Supplemental Information 3 [file peerj-13-19276-s003.zip › western blotting-marke/western blot-Total Cx43 EB1 N-cadherin/4-cx43-M(1).png]

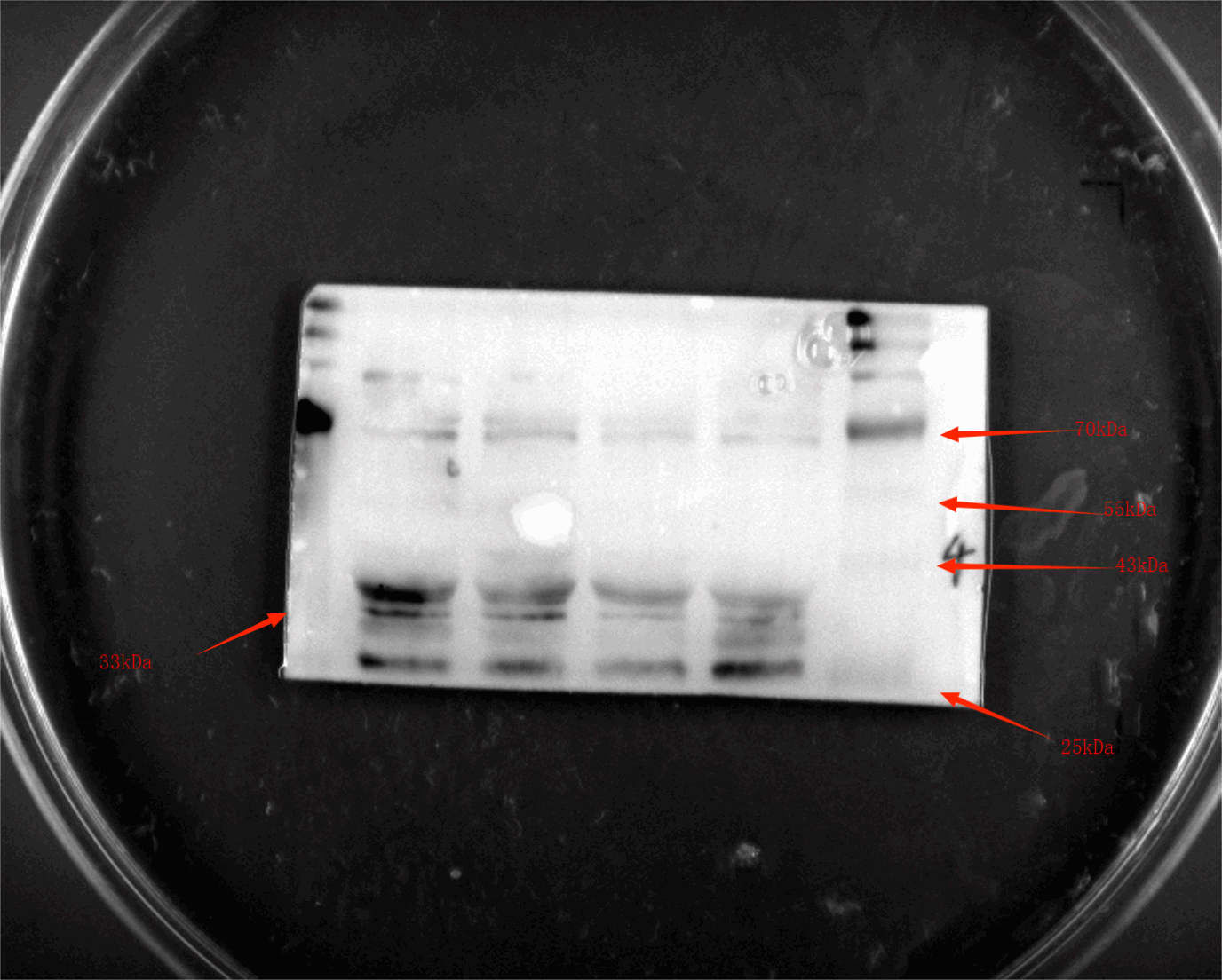

Supplement: Supplemental Information 3 [file peerj-13-19276-s003.zip › western blotting-marke/western blot-Total Cx43 EB1 N-cadherin/4-EB1-M(1).png]

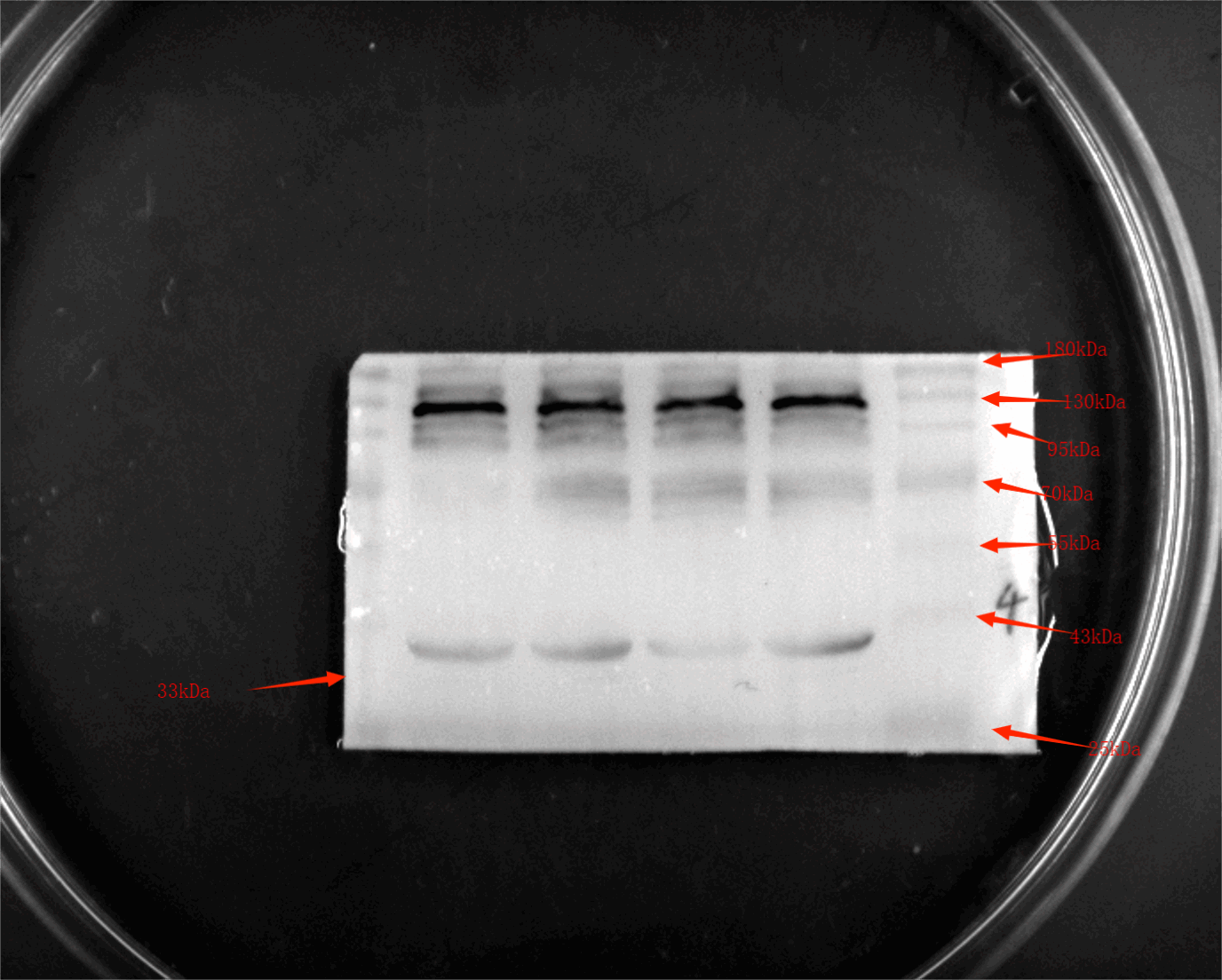

Supplement: Supplemental Information 3 [file peerj-13-19276-s003.zip › western blotting-marke/western blot-Total Cx43 EB1 N-cadherin/4-N-cadherin-M-used(1).png]

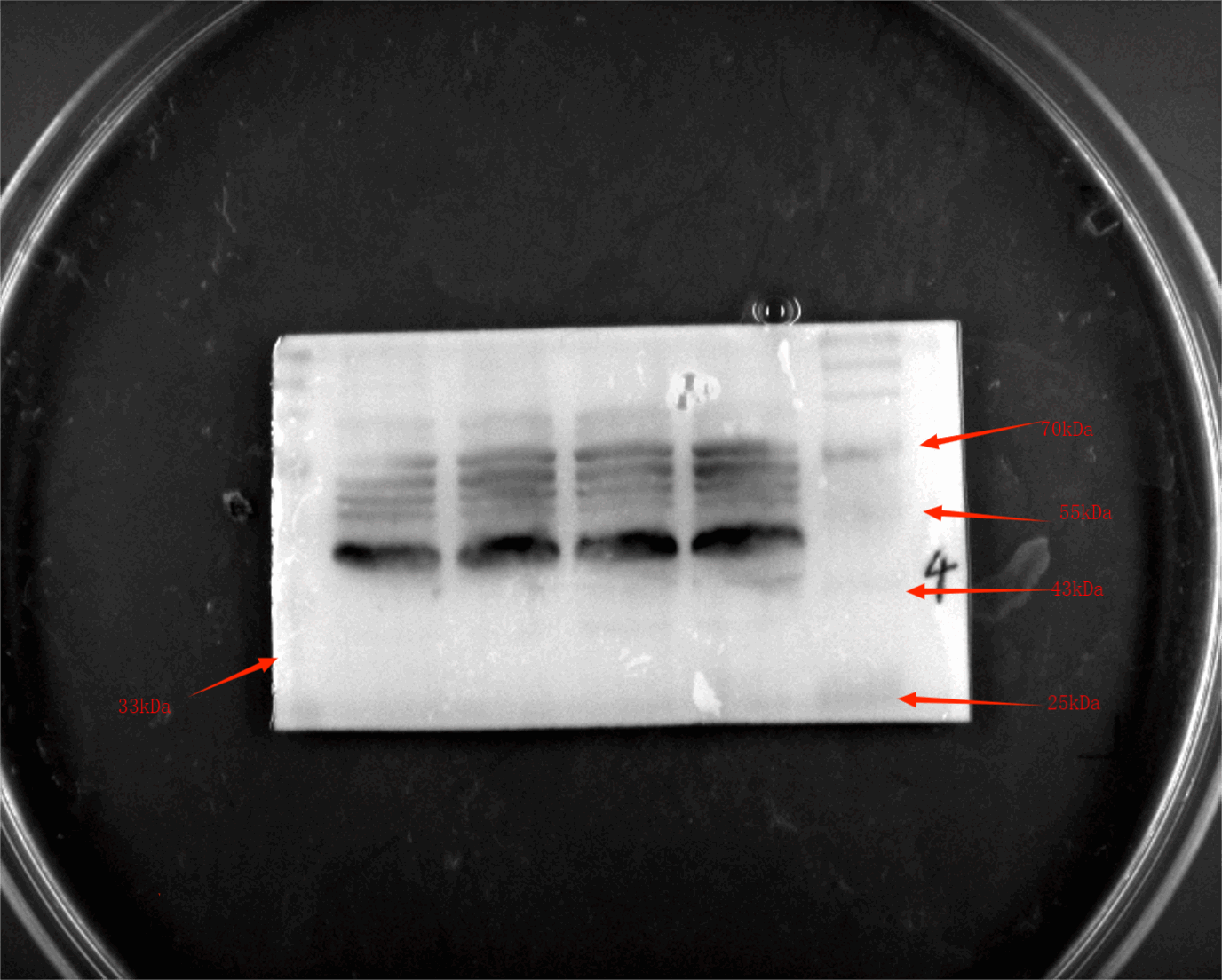

Supplement: Supplemental Information 3 [file peerj-13-19276-s003.zip › western blotting-marke/western blot-Total Cx43 EB1 N-cadherin/4-Tubulin-M(1).png]

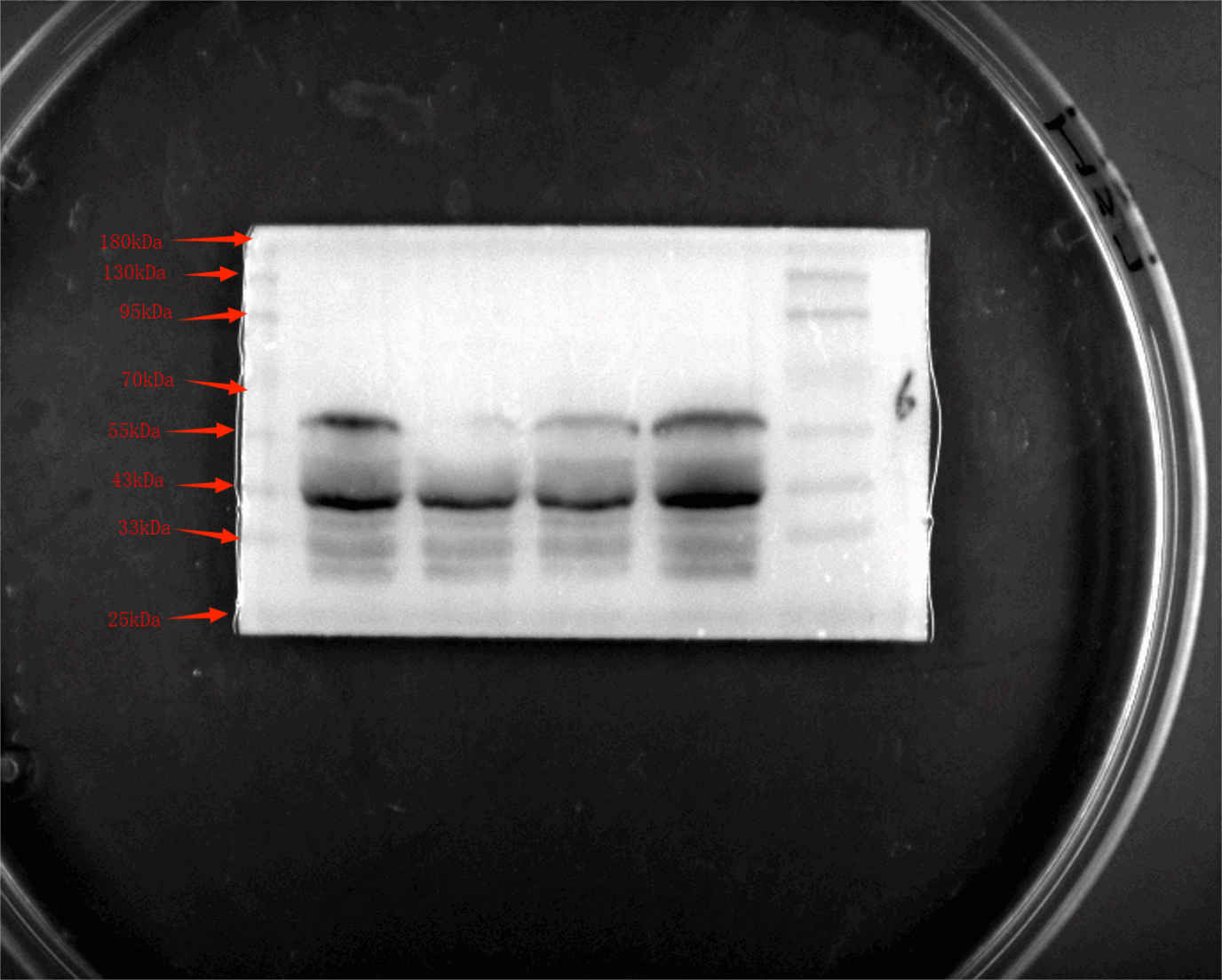

Supplement: Supplemental Information 3 [file peerj-13-19276-s003.zip › western blotting-marke/western blot-Total Cx43 EB1 N-cadherin/5-CX43-M(1).png]

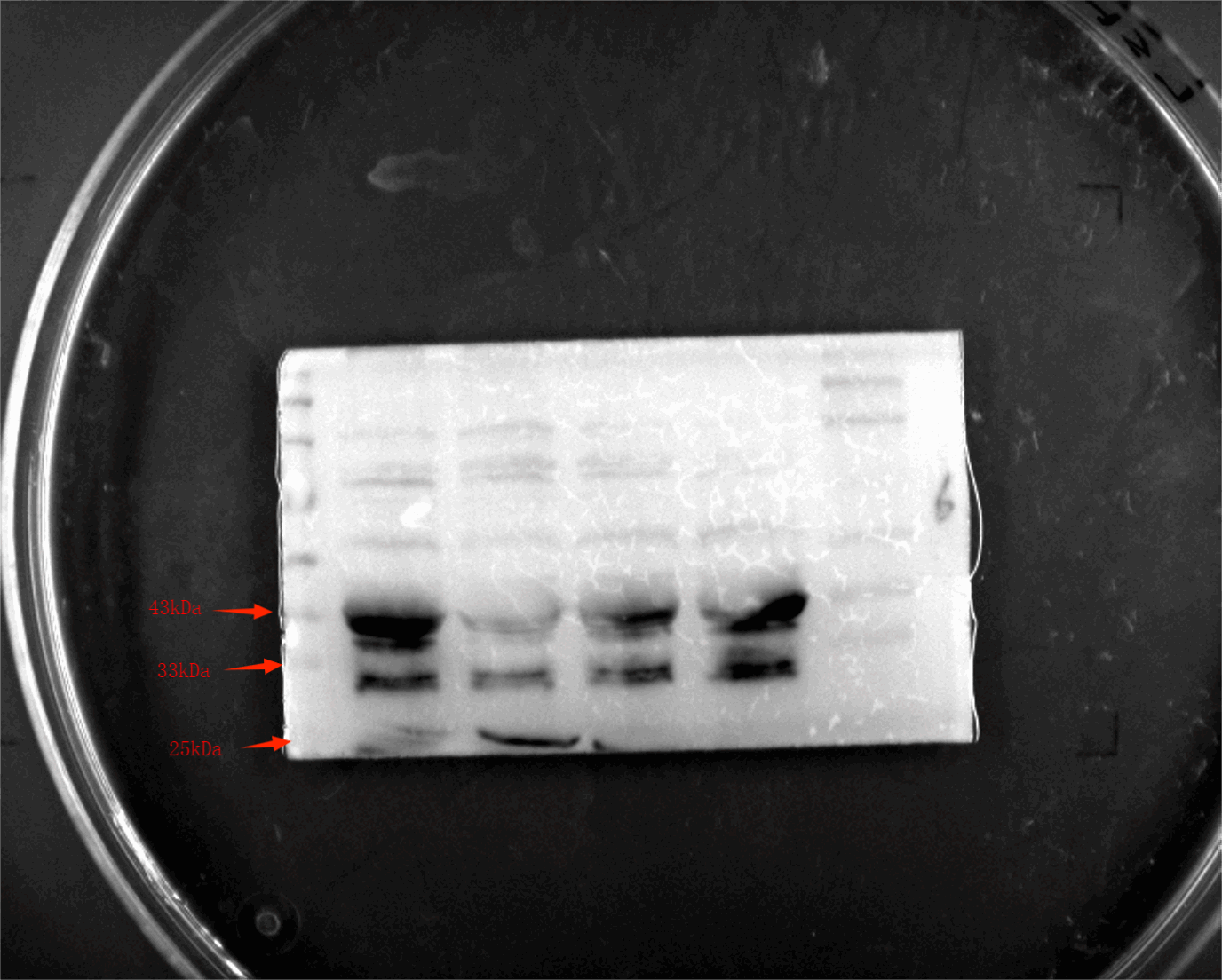

Supplement: Supplemental Information 3 [file peerj-13-19276-s003.zip › western blotting-marke/western blot-Total Cx43 EB1 N-cadherin/5-EB1-M(1).png]

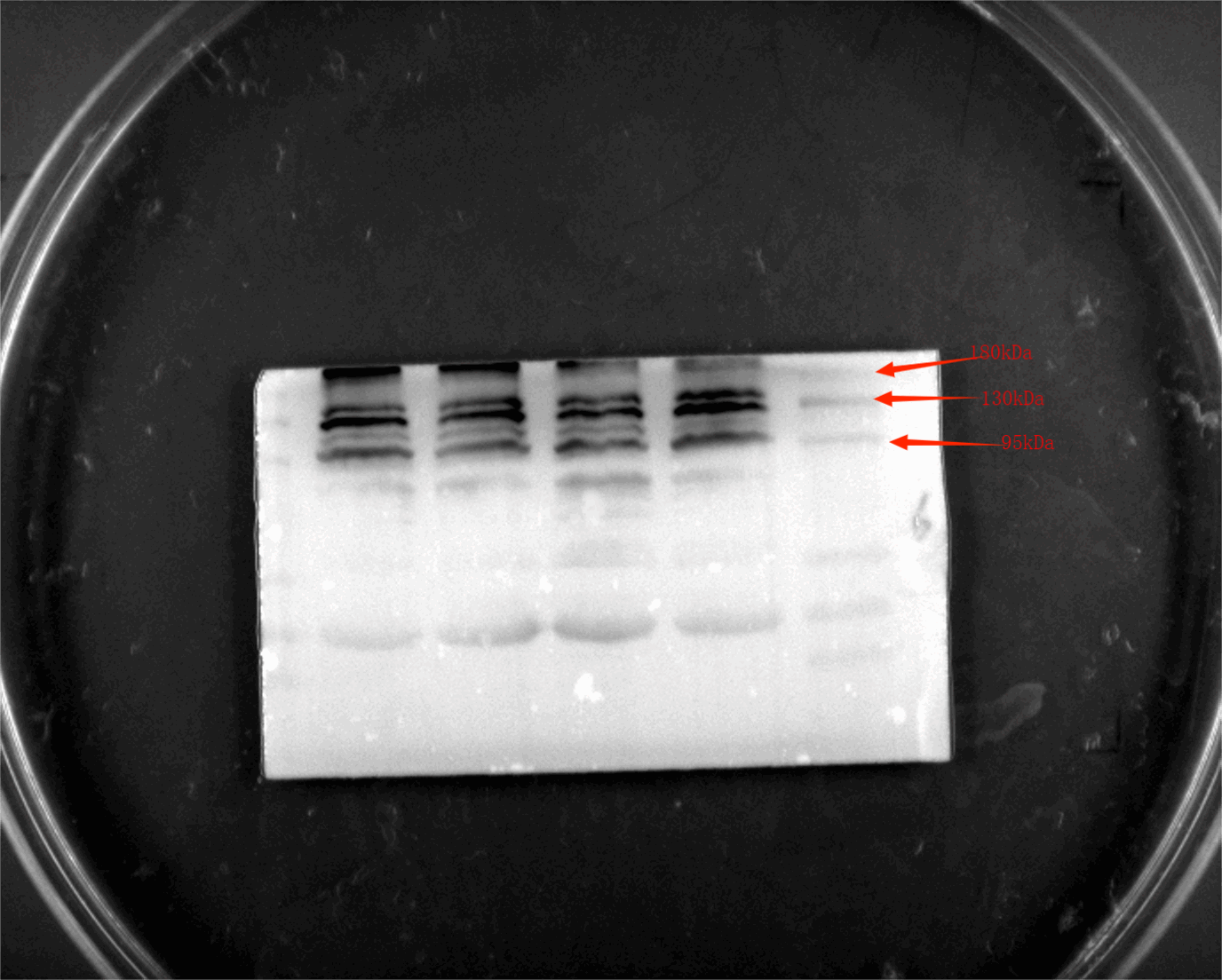

Supplement: Supplemental Information 3 [file peerj-13-19276-s003.zip › western blotting-marke/western blot-Total Cx43 EB1 N-cadherin/5-N-cadherin-M(1).png]

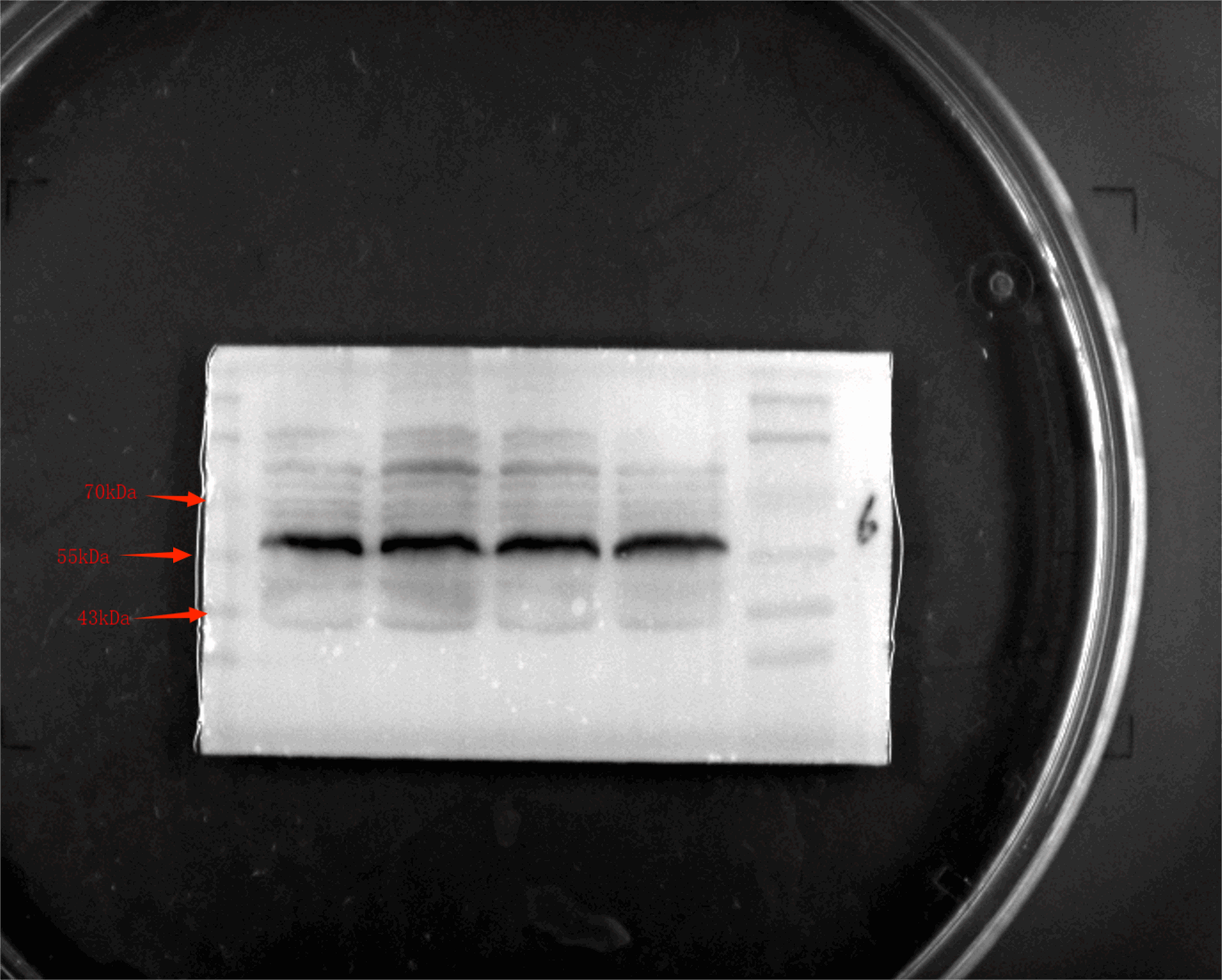

Supplement: Supplemental Information 3 [file peerj-13-19276-s003.zip › western blotting-marke/western blot-Total Cx43 EB1 N-cadherin/5-Tubulin-M(1).png]

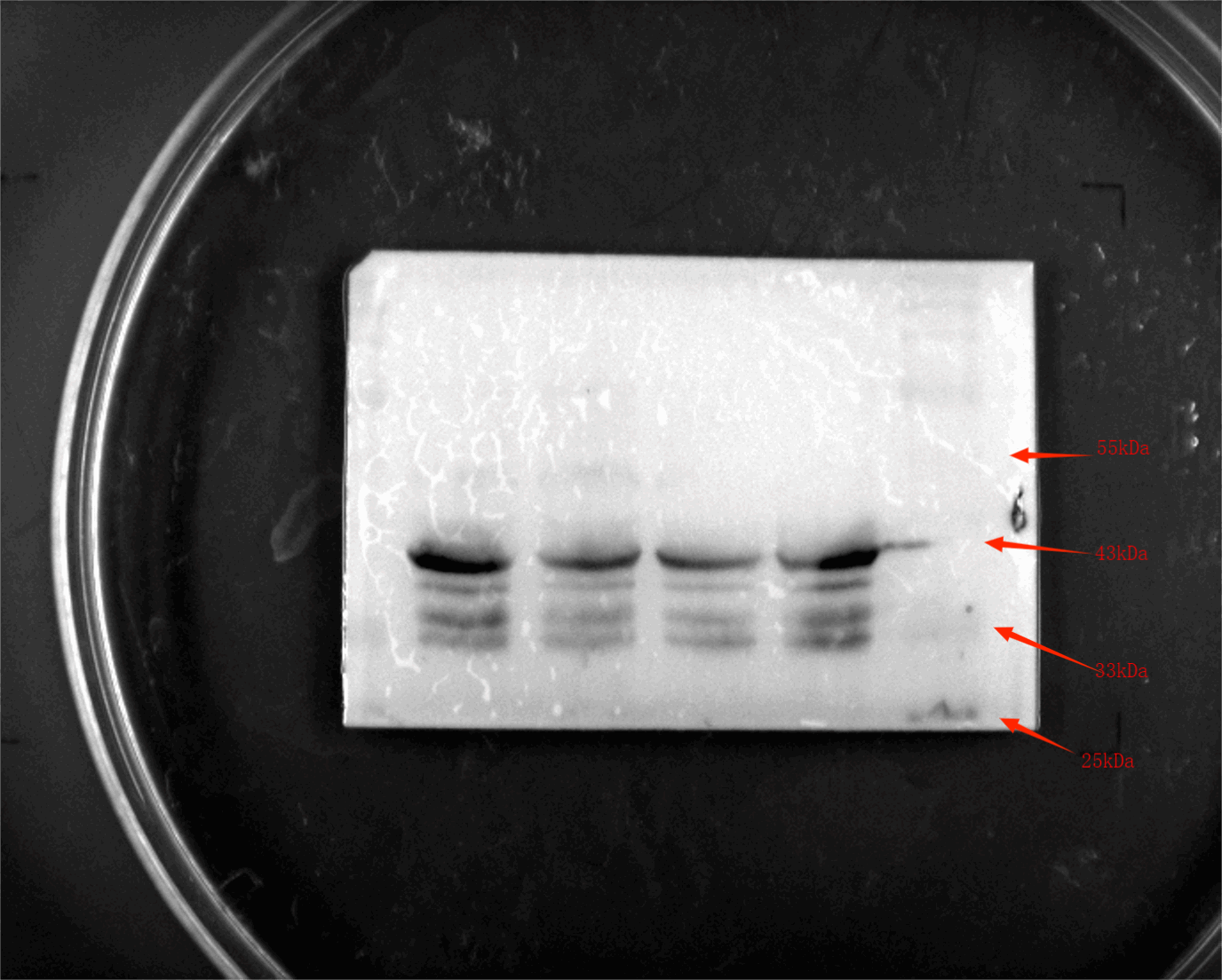

Supplement: Supplemental Information 3 [file peerj-13-19276-s003.zip › western blotting-marke/western blot-Total Cx43 EB1 N-cadherin/6-cx43-M(1).png]

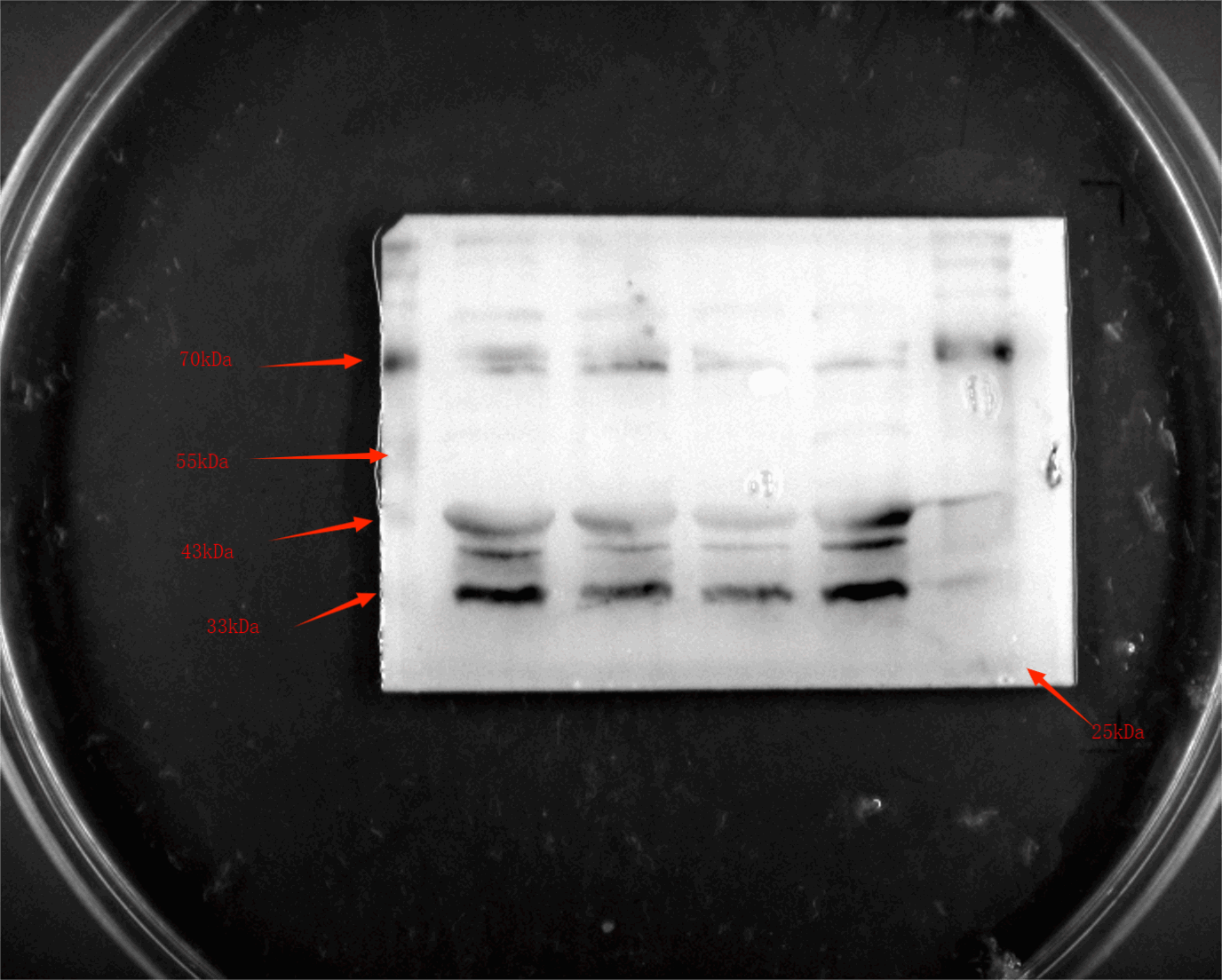

Supplement: Supplemental Information 3 [file peerj-13-19276-s003.zip › western blotting-marke/western blot-Total Cx43 EB1 N-cadherin/6-EB1-M(1).png]

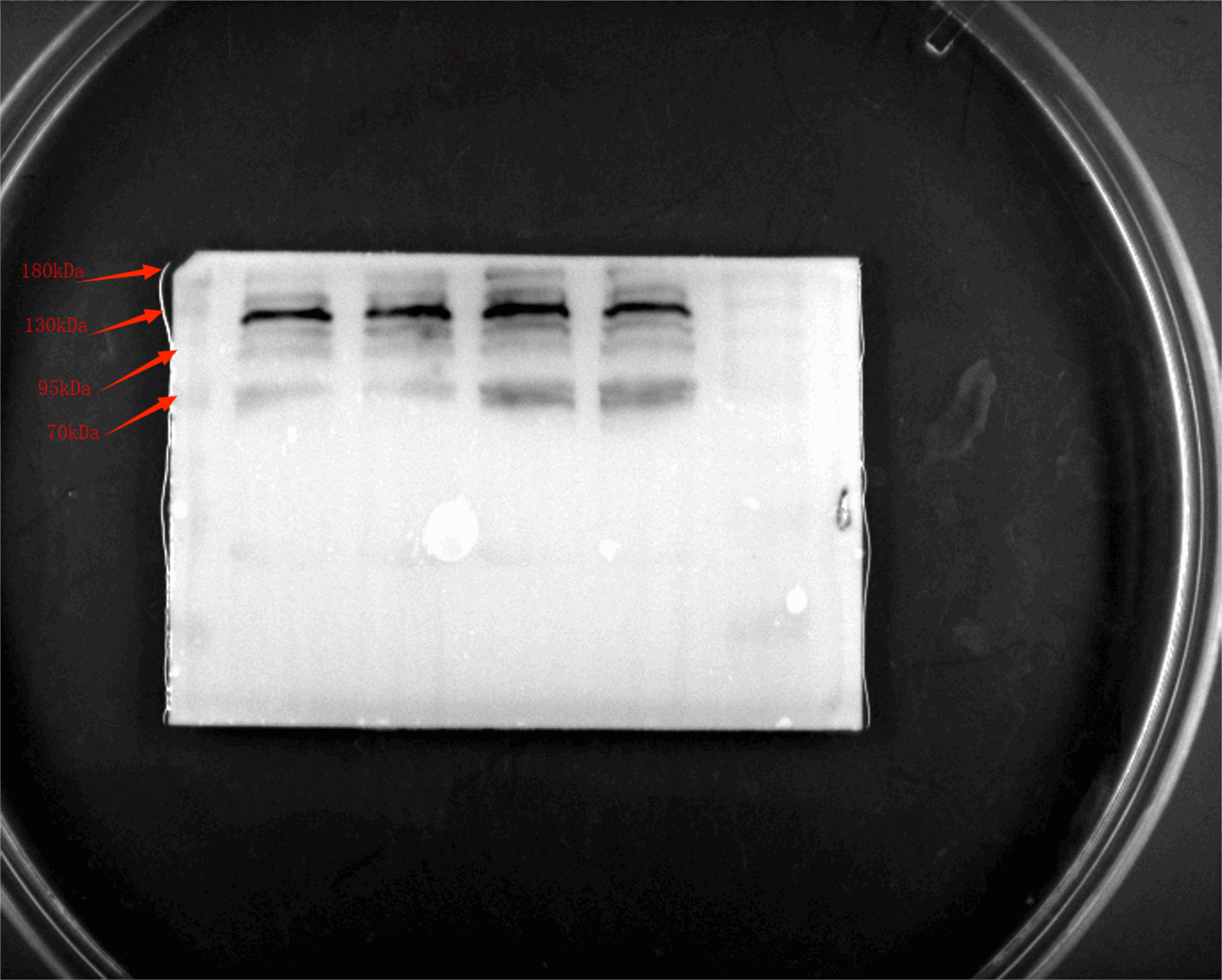

Supplement: Supplemental Information 3 [file peerj-13-19276-s003.zip › western blotting-marke/western blot-Total Cx43 EB1 N-cadherin/6-N-cadherin-M(1).png]

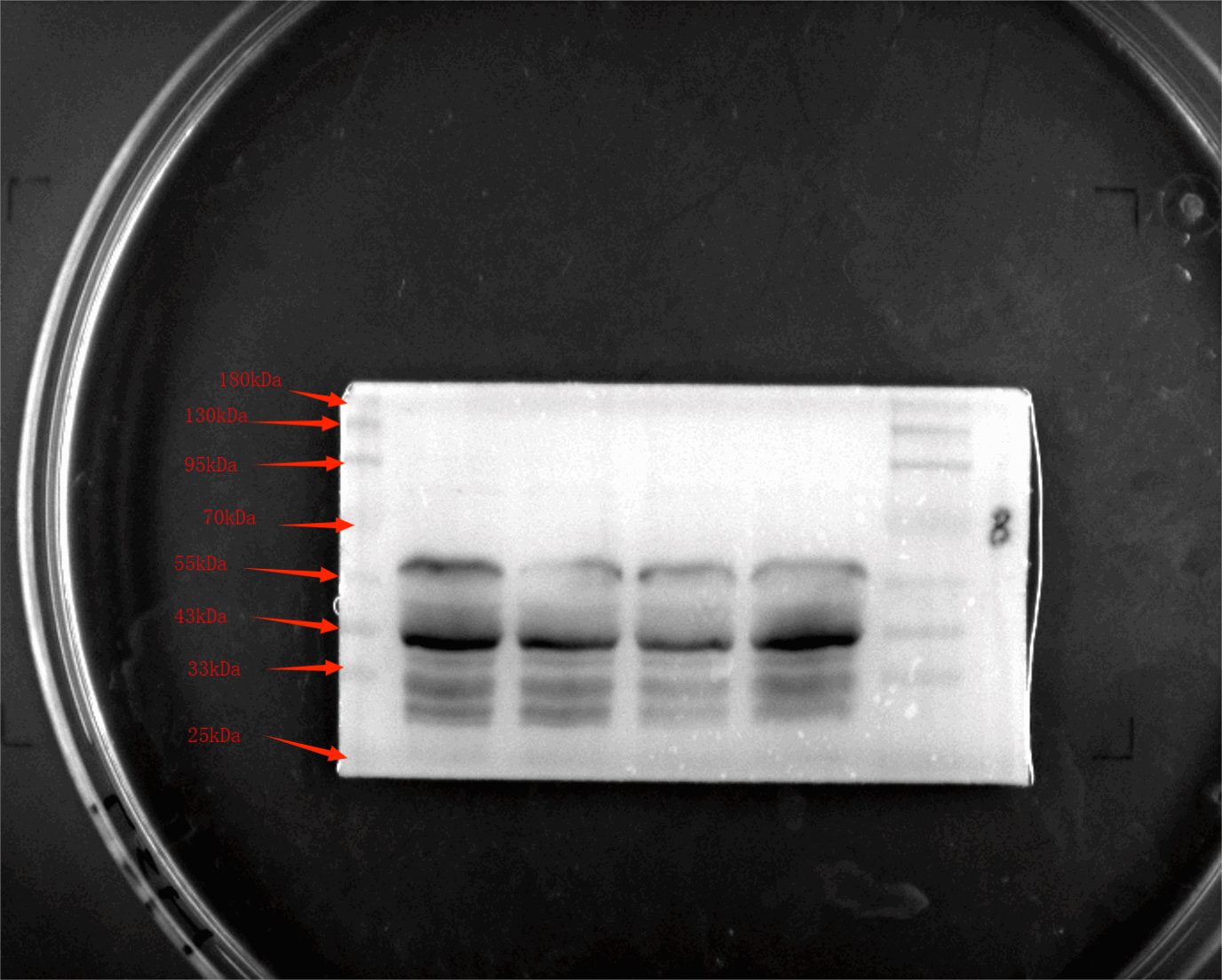

Supplement: Supplemental Information 3 [file peerj-13-19276-s003.zip › western blotting-marke/western blot-Total Cx43 EB1 N-cadherin/8-CX43-M(1).png]

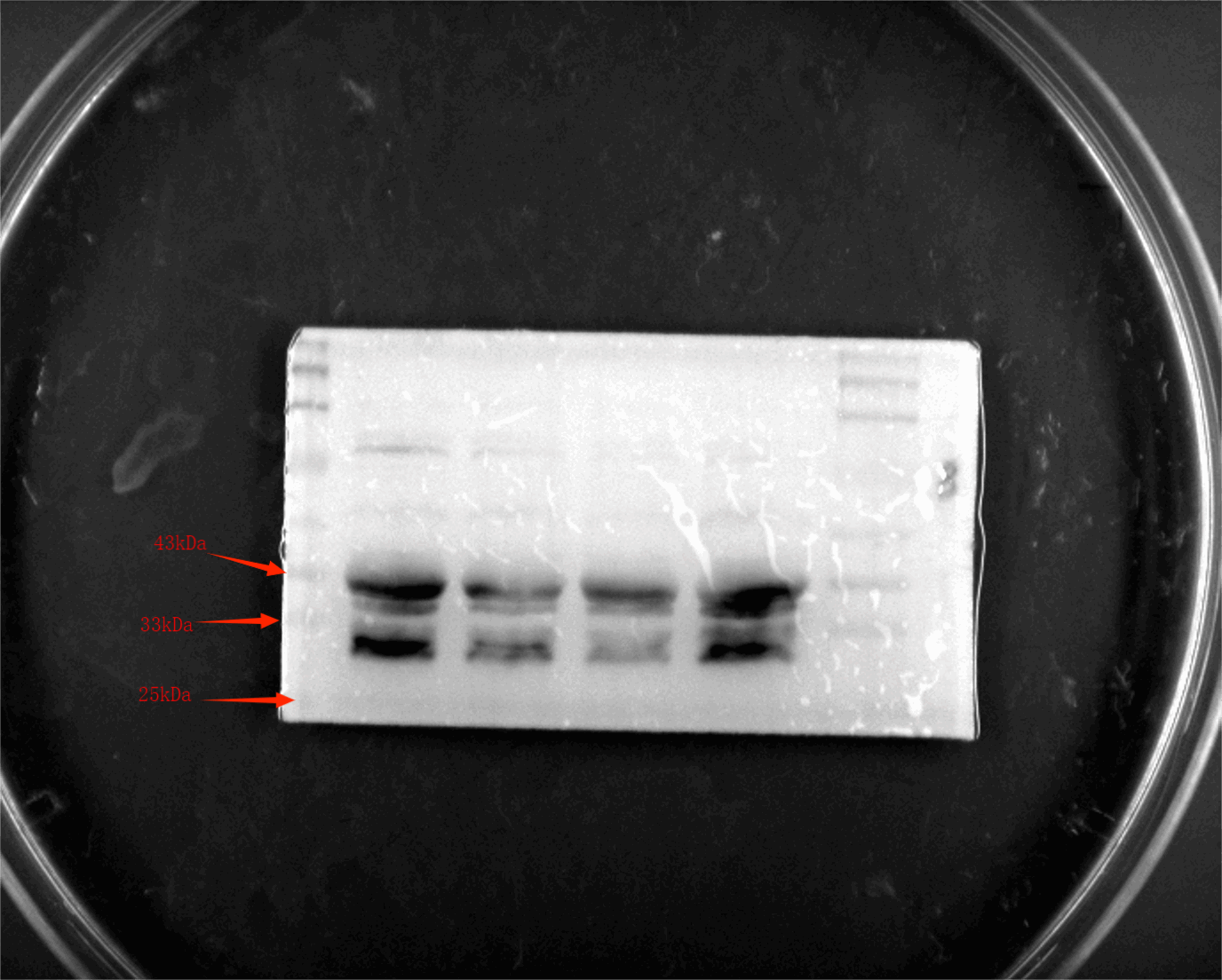

Supplement: Supplemental Information 3 [file peerj-13-19276-s003.zip › western blotting-marke/western blot-Total Cx43 EB1 N-cadherin/8-EB1-M(1).png]

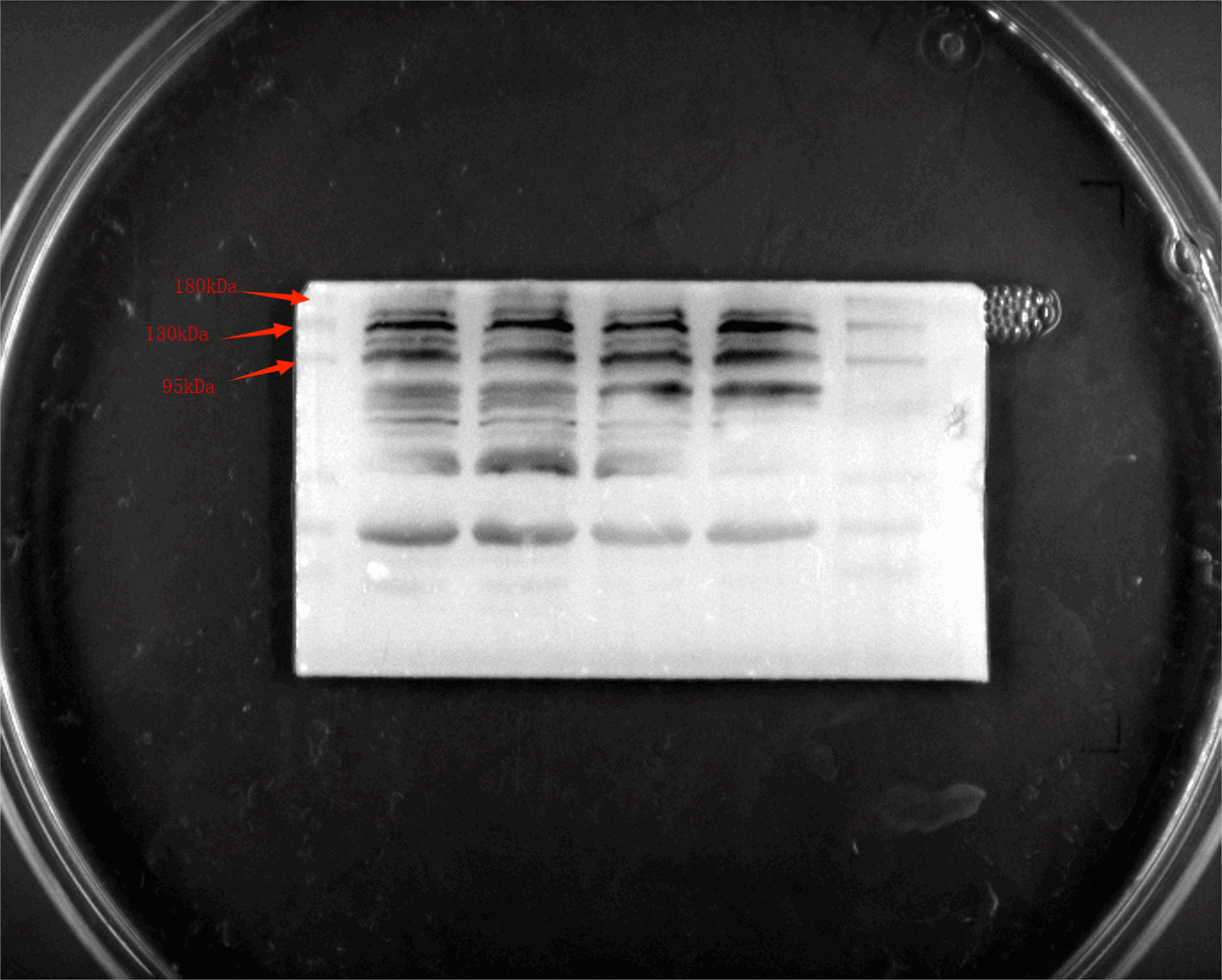

Supplement: Supplemental Information 3 [file peerj-13-19276-s003.zip › western blotting-marke/western blot-Total Cx43 EB1 N-cadherin/8-N-cadherin-M(1).png]

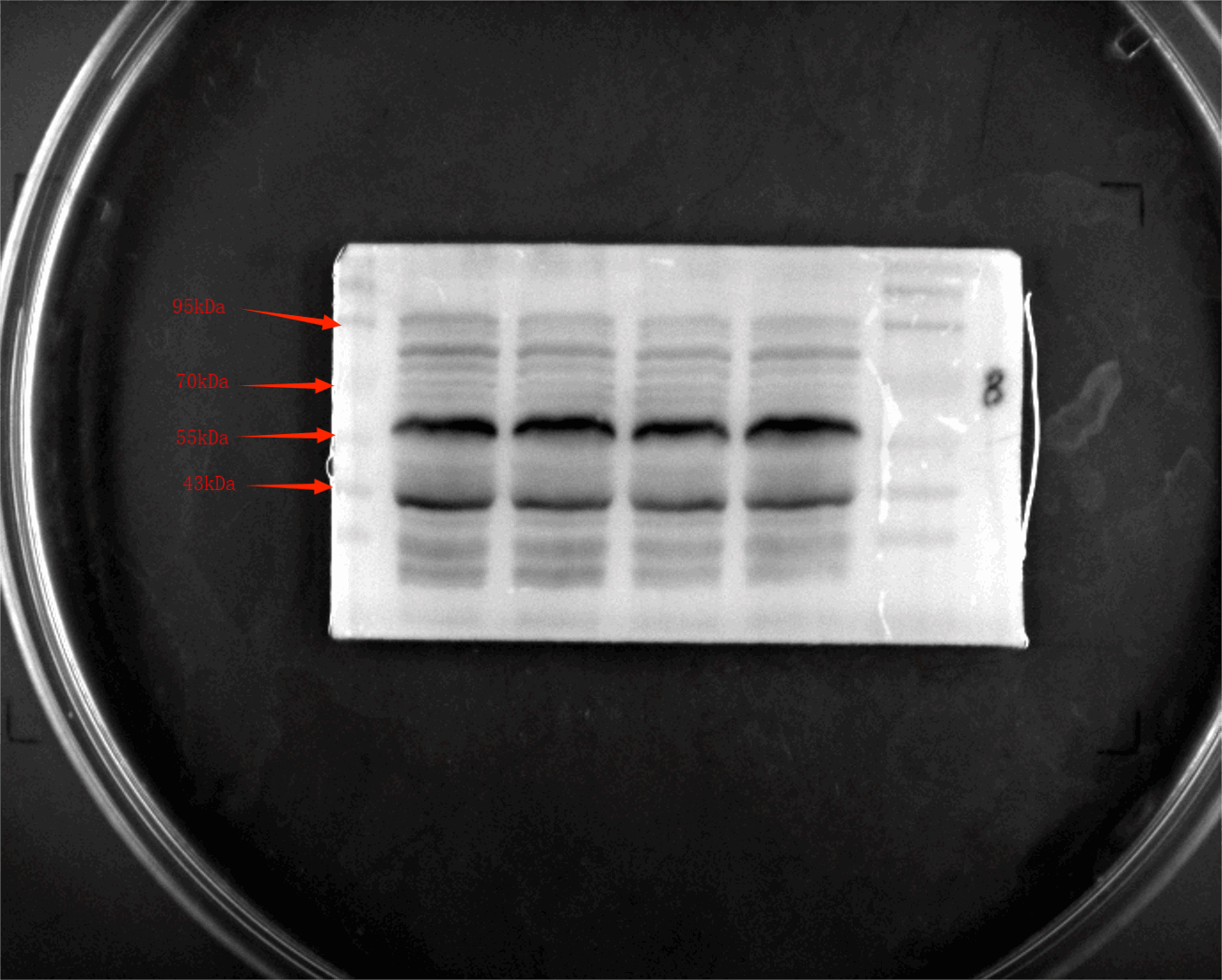

Supplement: Supplemental Information 3 [file peerj-13-19276-s003.zip › western blotting-marke/western blot-Total Cx43 EB1 N-cadherin/8-Tubulin-M(1).png]

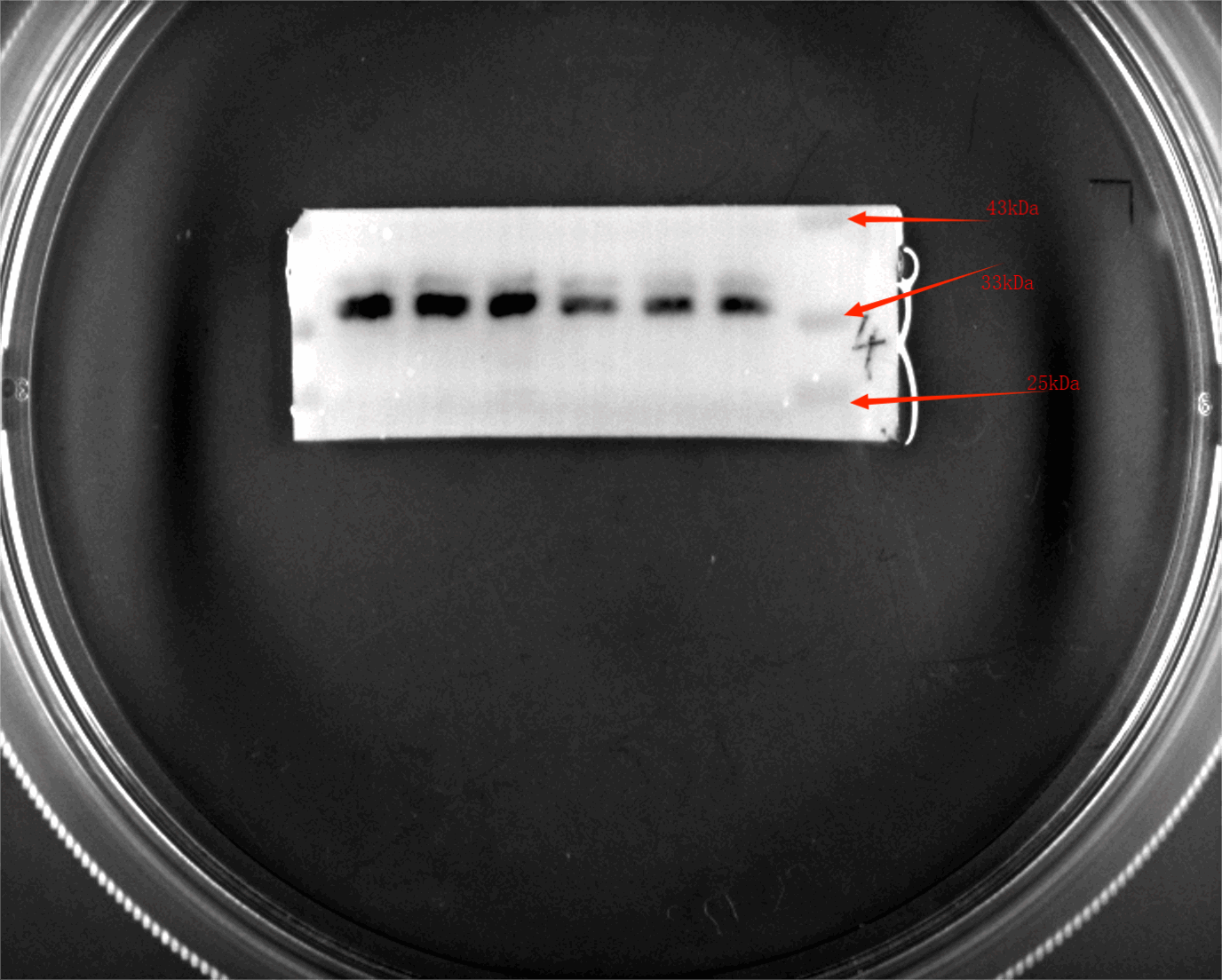

Supplement: Supplemental Information 3 [file peerj-13-19276-s003.zip › western blotting-marke/western blot-(CP IR group) EB1/1-EB1-M(1).png]

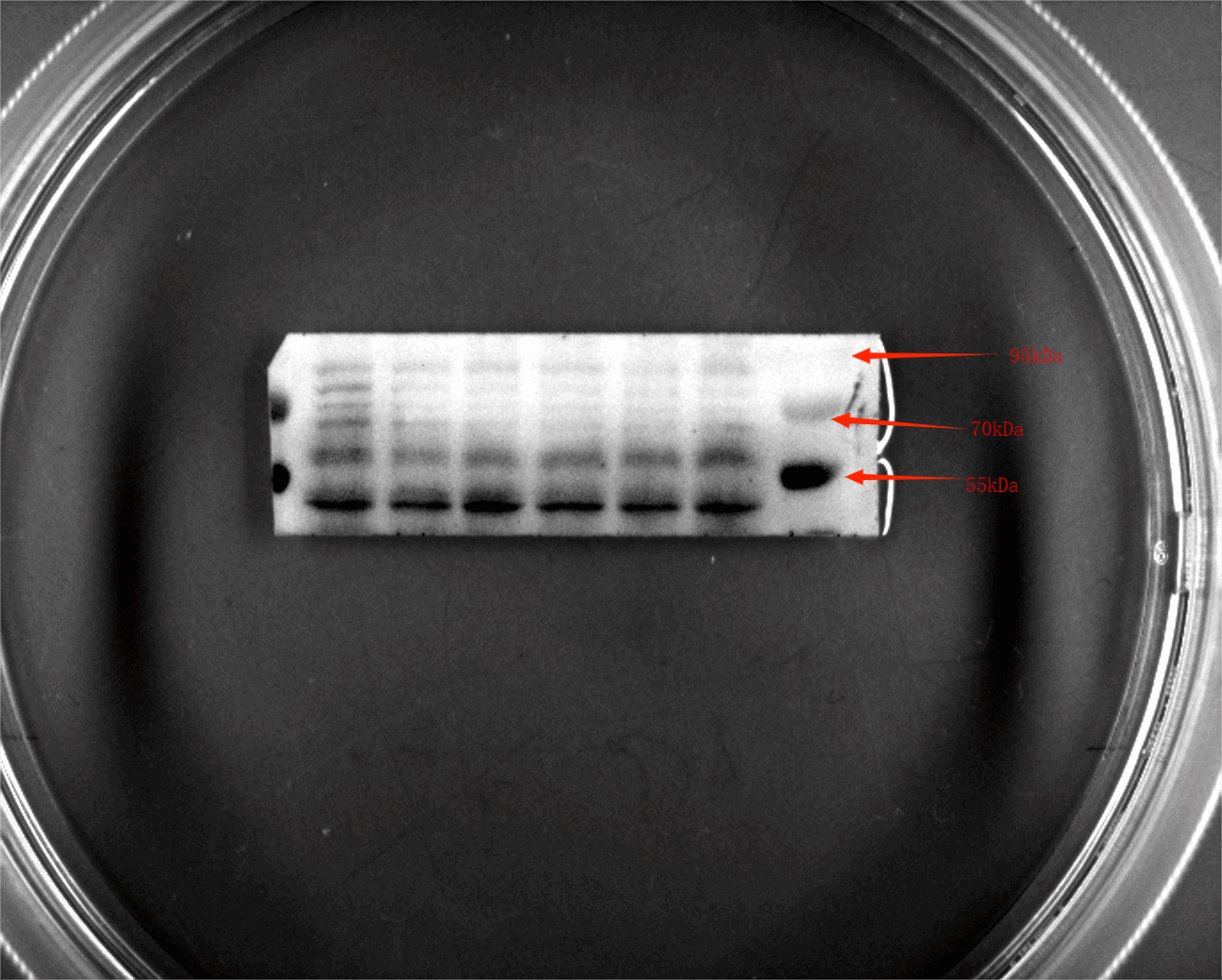

Supplement: Supplemental Information 3 [file peerj-13-19276-s003.zip › western blotting-marke/western blot-(CP IR group) EB1/1-Tubulin-M(1).png]

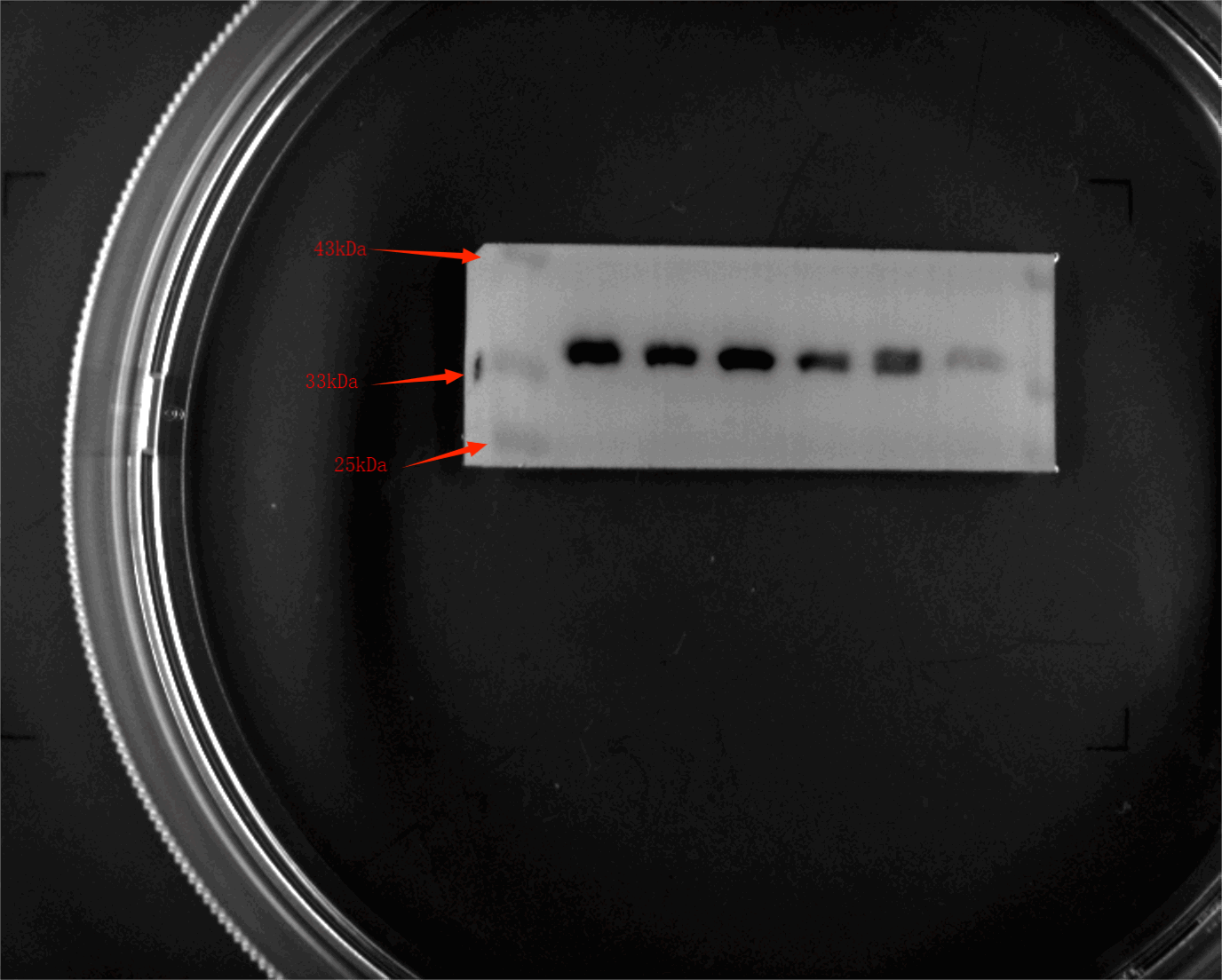

Supplement: Supplemental Information 3 [file peerj-13-19276-s003.zip › western blotting-marke/western blot-(CP IR group) EB1/2-EB1-M(1).png]

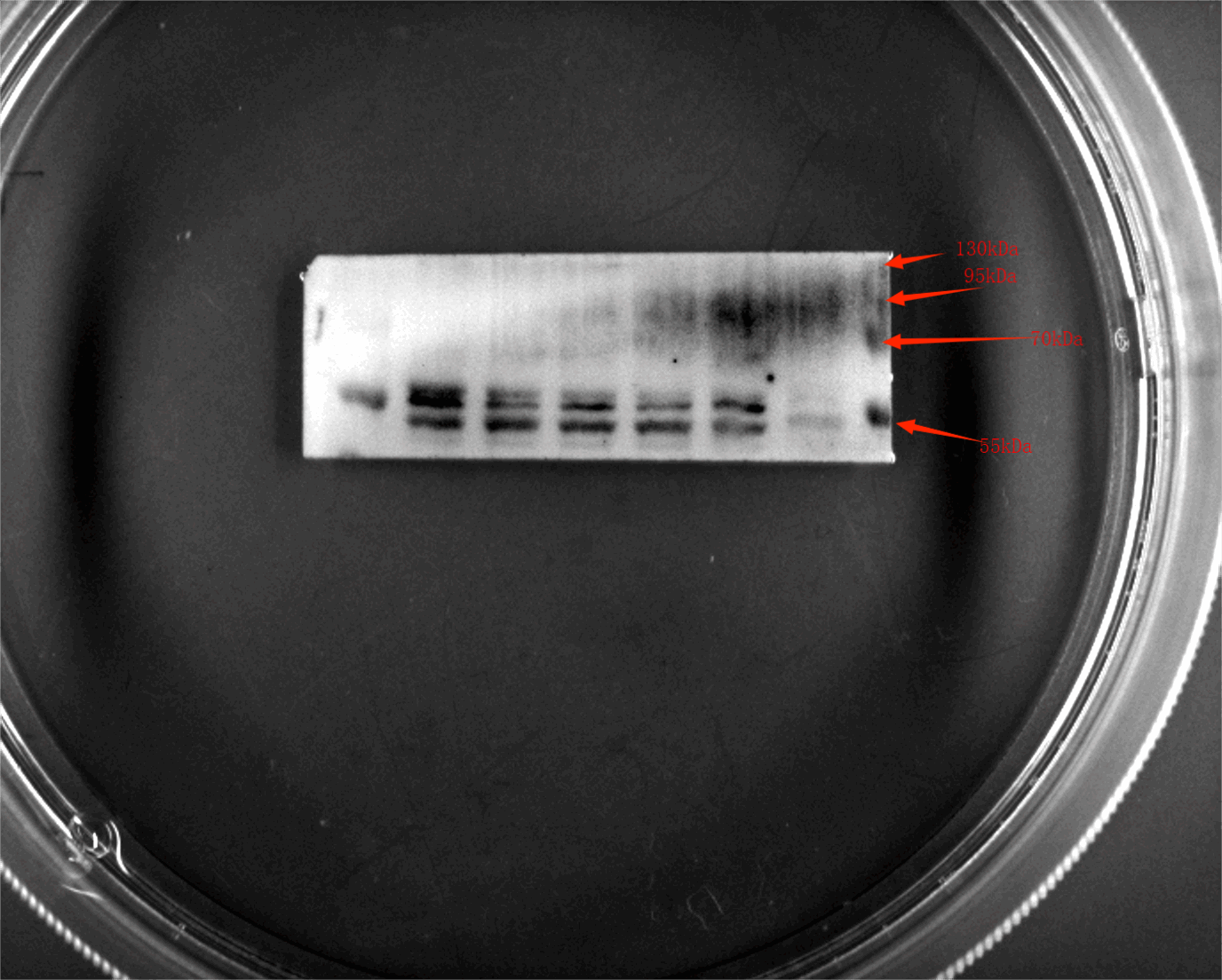

Supplement: Supplemental Information 3 [file peerj-13-19276-s003.zip › western blotting-marke/western blot-(CP IR group) EB1/2-Tubulin-M(1).png]

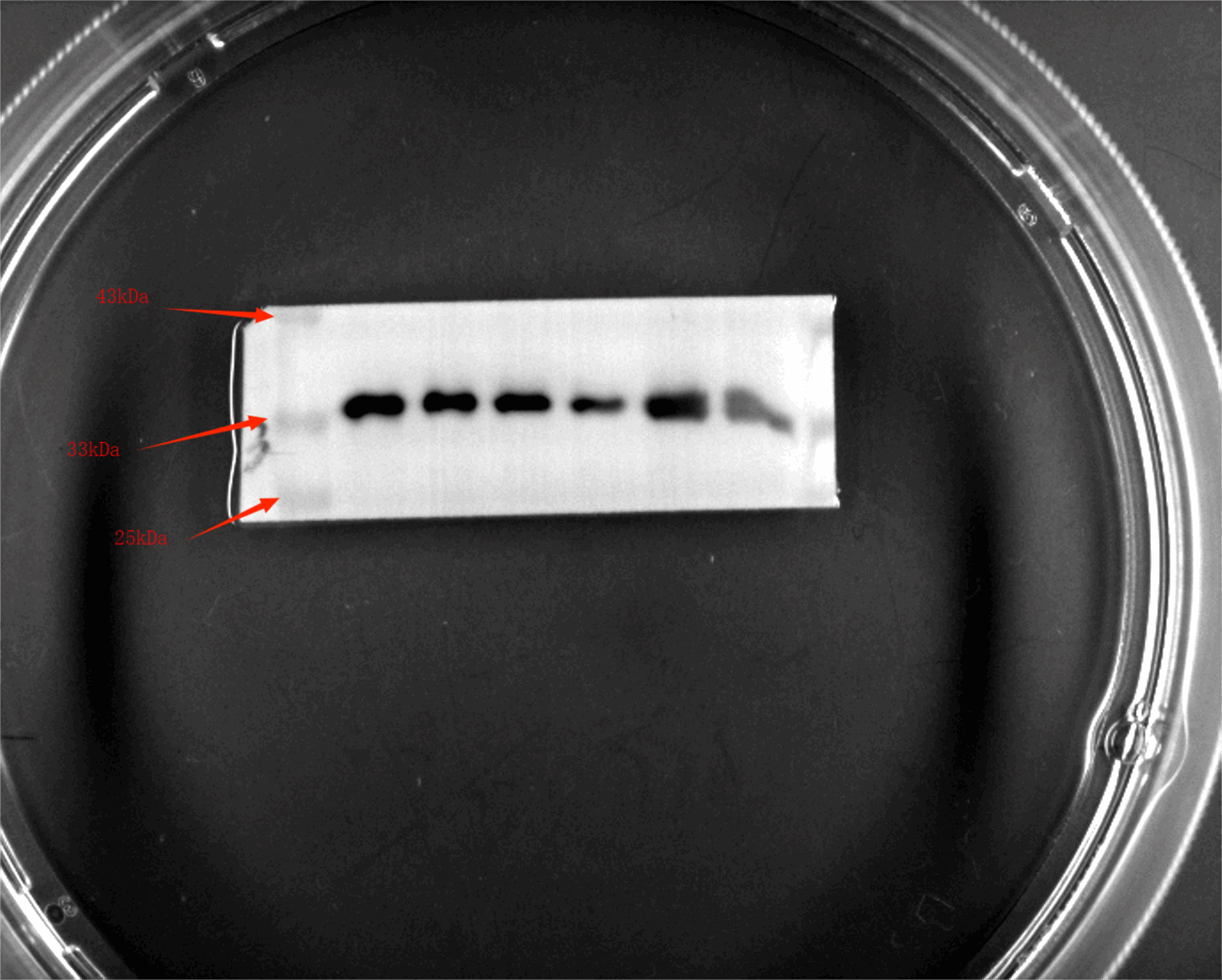

Supplement: Supplemental Information 3 [file peerj-13-19276-s003.zip › western blotting-marke/western blot-(CP IR group) EB1/3-EB1-M(1).png]

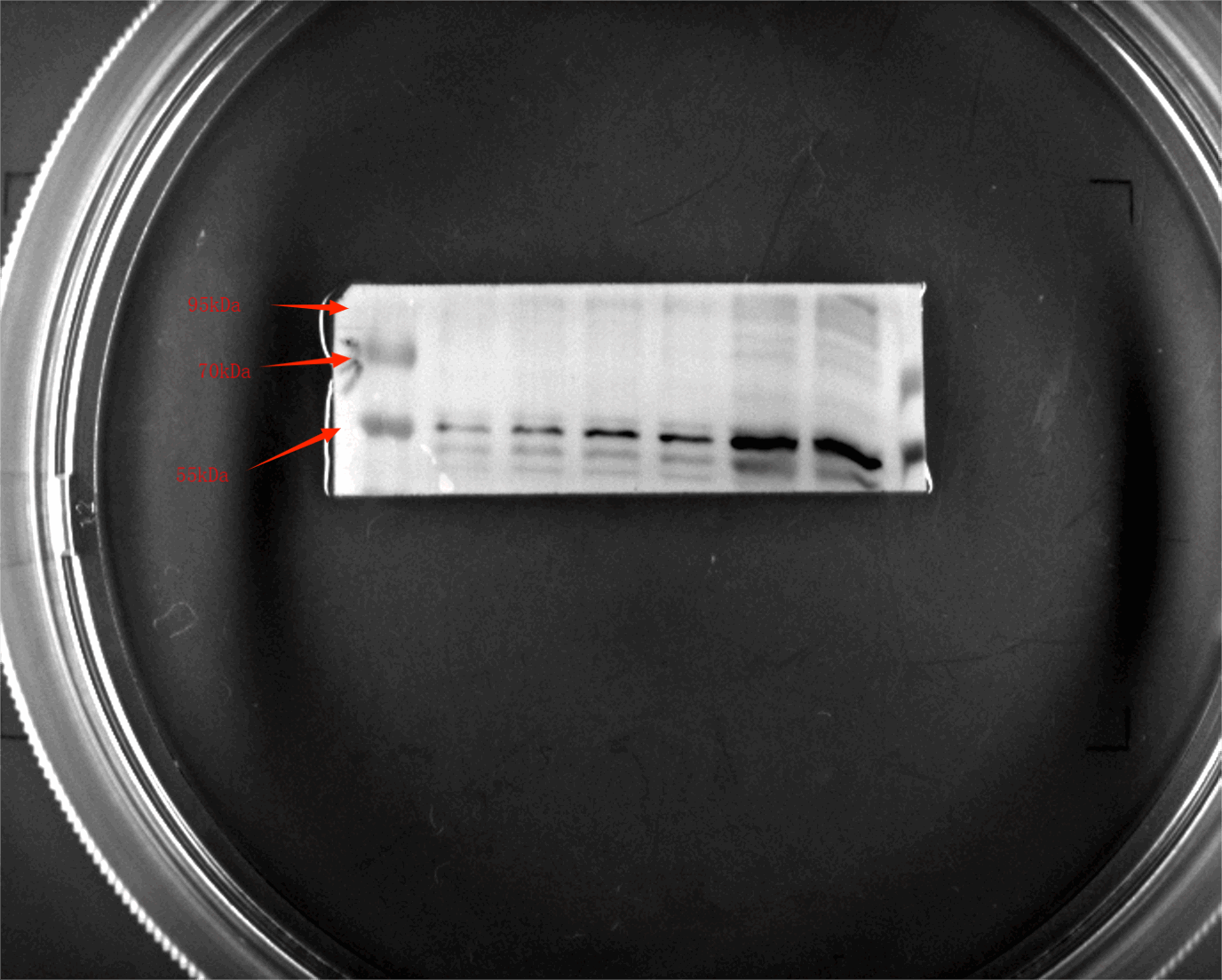

Supplement: Supplemental Information 3 [file peerj-13-19276-s003.zip › western blotting-marke/western blot-(CP IR group) EB1/3-Tubulin-M(1).png]

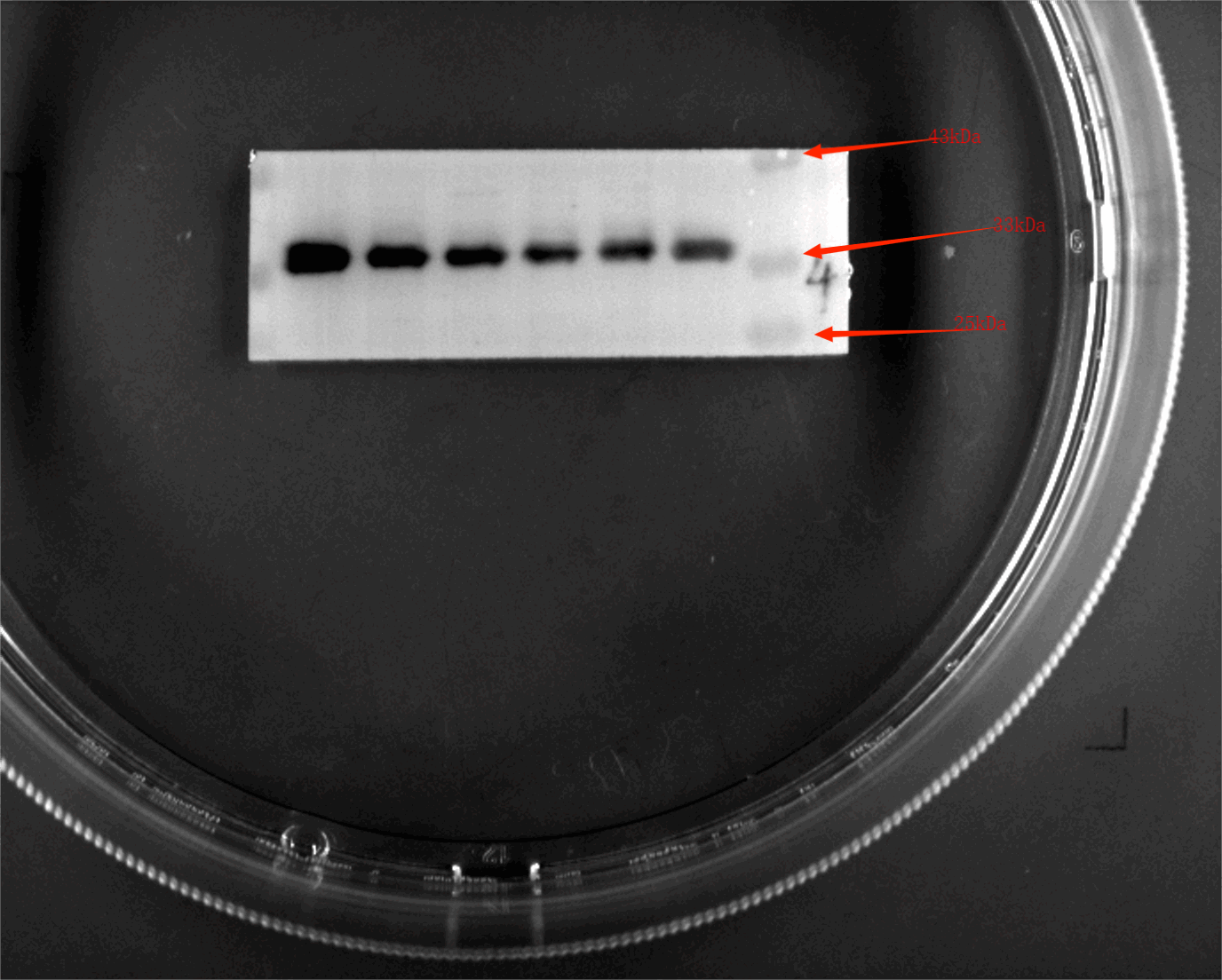

Supplement: Supplemental Information 3 [file peerj-13-19276-s003.zip › western blotting-marke/western blot-(CP IR group) EB1/4-EB1-M-used(1).png]

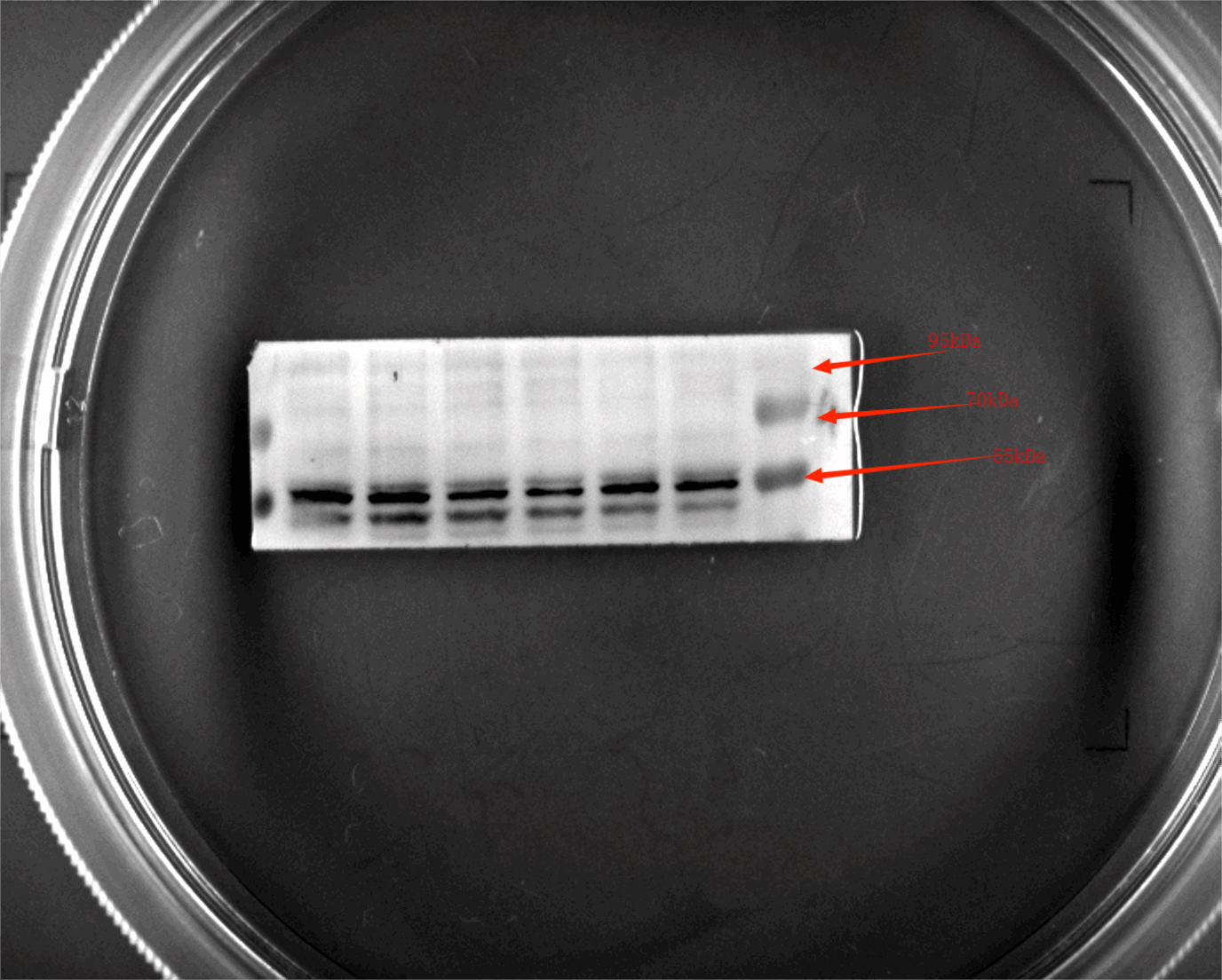

Supplement: Supplemental Information 3 [file peerj-13-19276-s003.zip › western blotting-marke/western blot-(CP IR group) EB1/4-Tubulin-M-used(1).png]

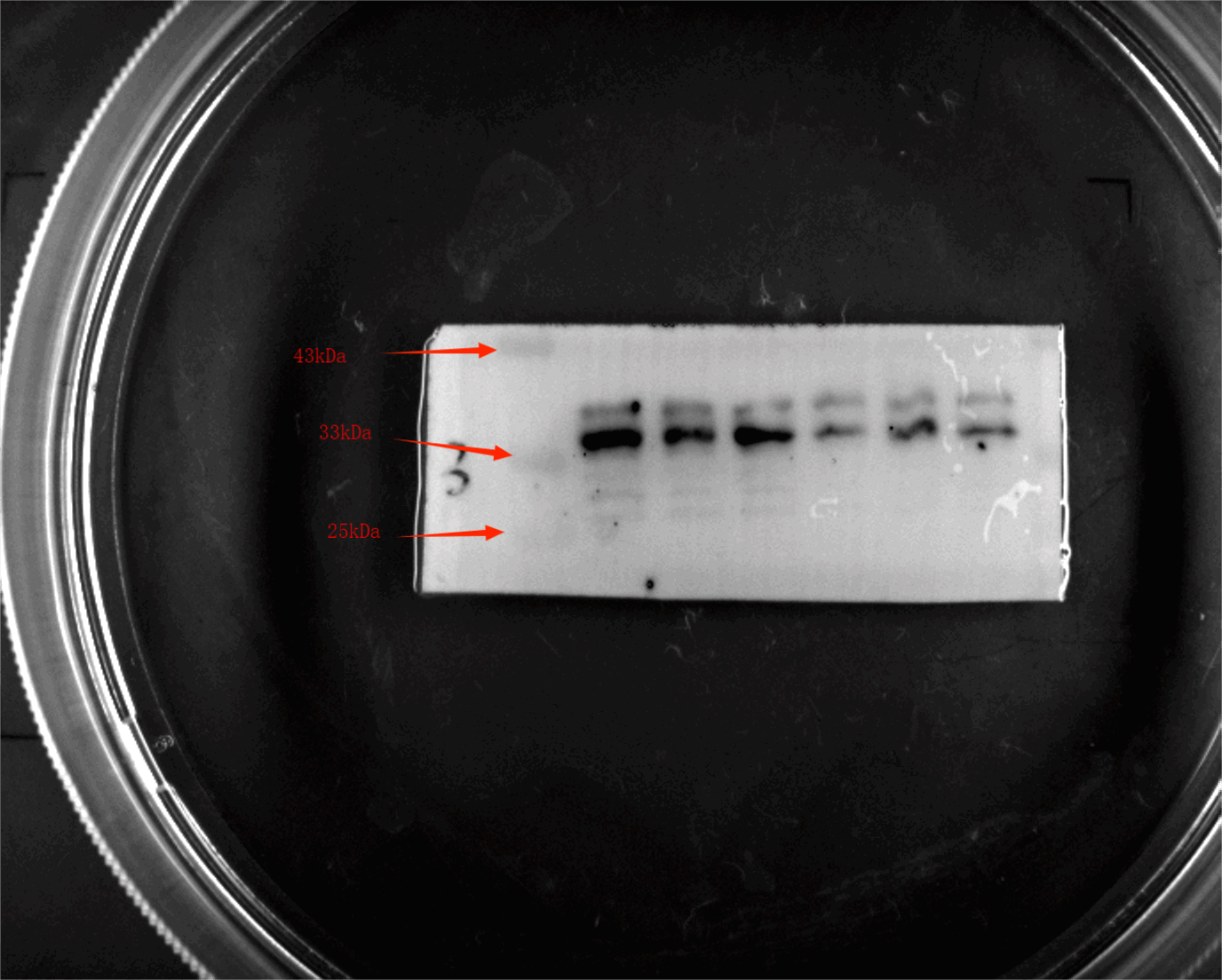

Supplement: Supplemental Information 3 [file peerj-13-19276-s003.zip › western blotting-marke/western blot-(CP IR group) EB1/5-EB1-M(1).png]

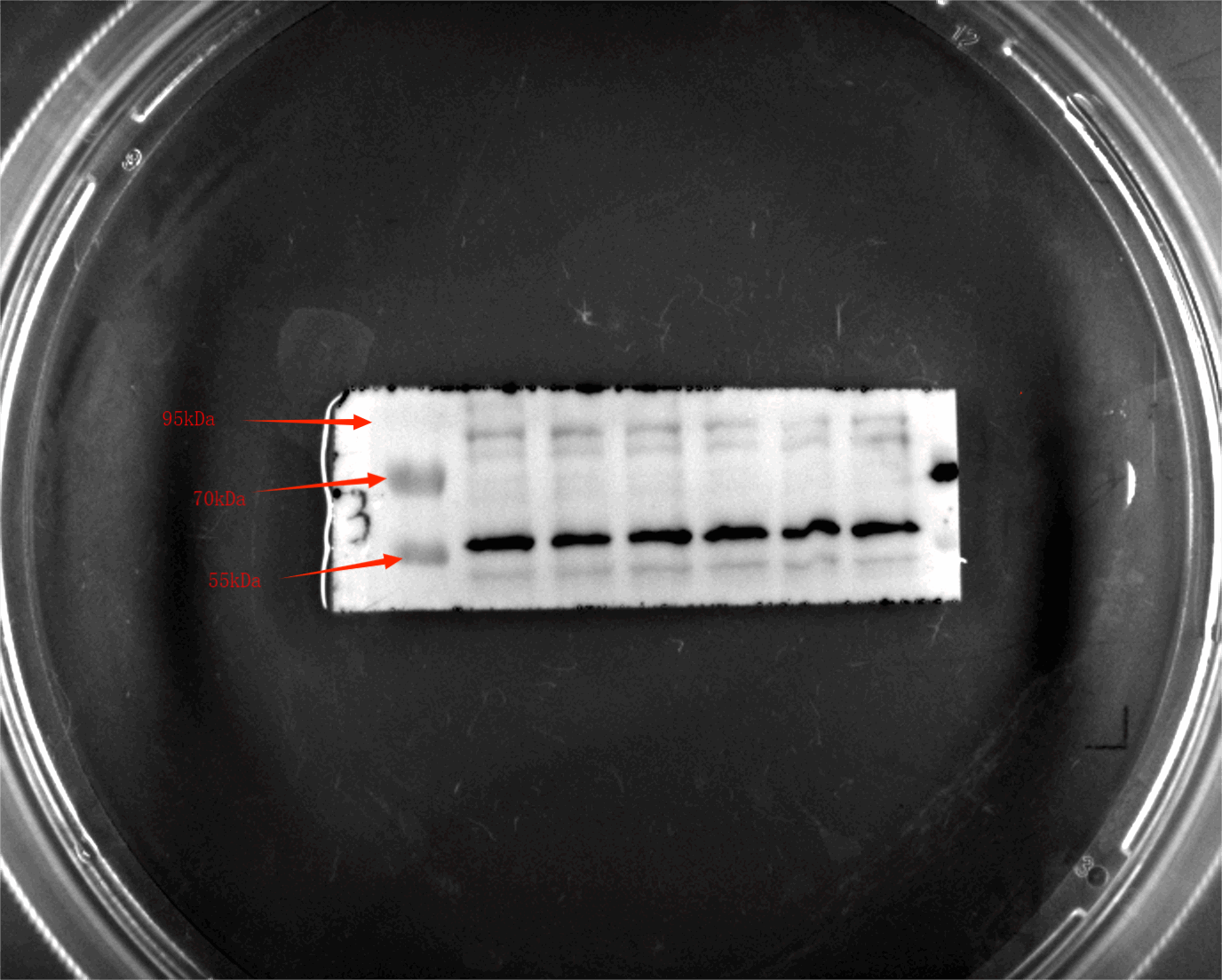

Supplement: Supplemental Information 3 [file peerj-13-19276-s003.zip › western blotting-marke/western blot-(CP IR group) EB1/5-Tubulin-M(1).png]

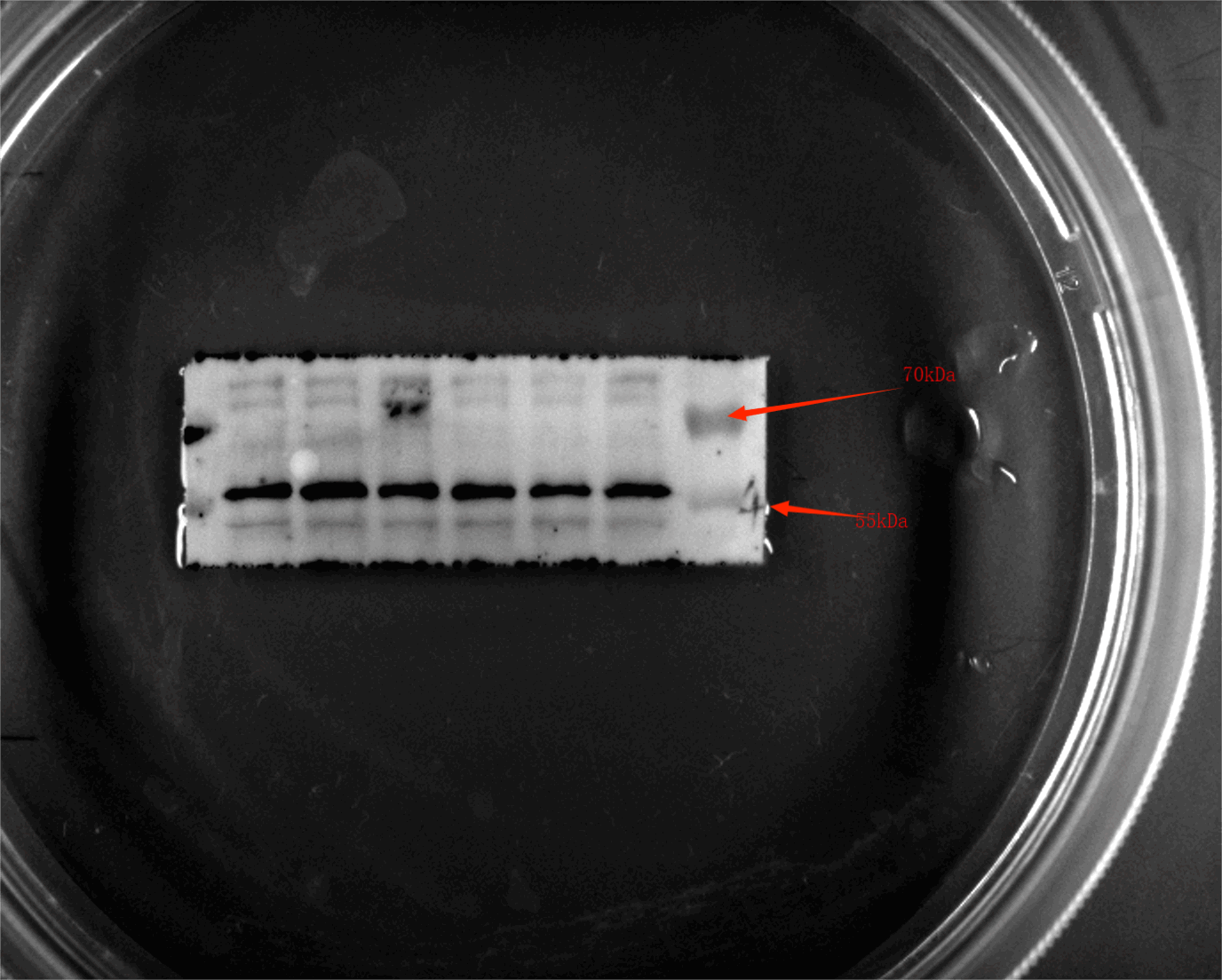

Supplement: Supplemental Information 3 [file peerj-13-19276-s003.zip › western blotting-marke/western blot-(CP IR group) EB1/6-Tubulin-M(1).png]

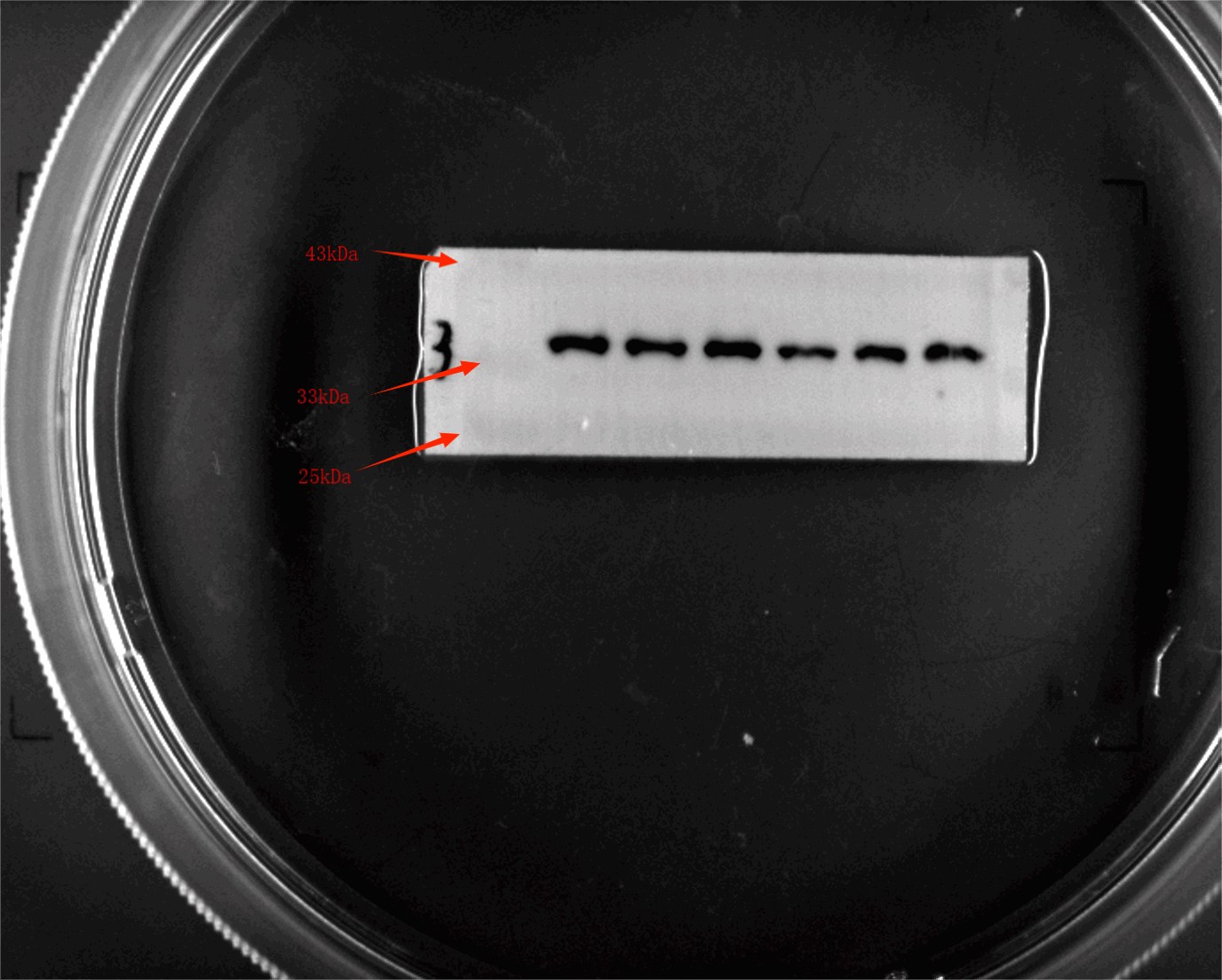

Supplement: Supplemental Information 3 [file peerj-13-19276-s003.zip › western blotting-marke/western blot-(CP IR group) EB1/7-EB1-M(1).png]

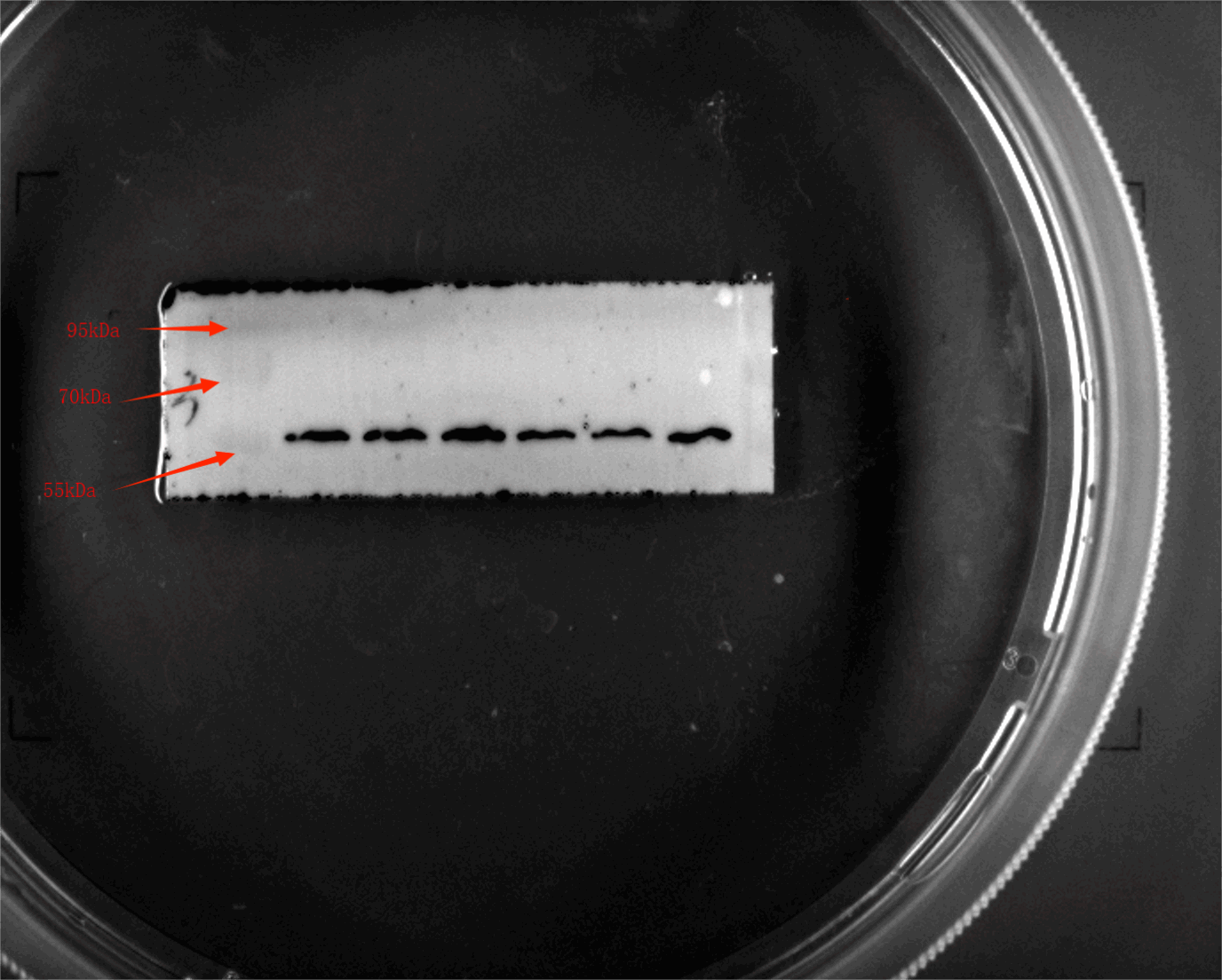

Supplement: Supplemental Information 3 [file peerj-13-19276-s003.zip › western blotting-marke/western blot-(CP IR group) EB1/7-Tubulin-M(1).png]

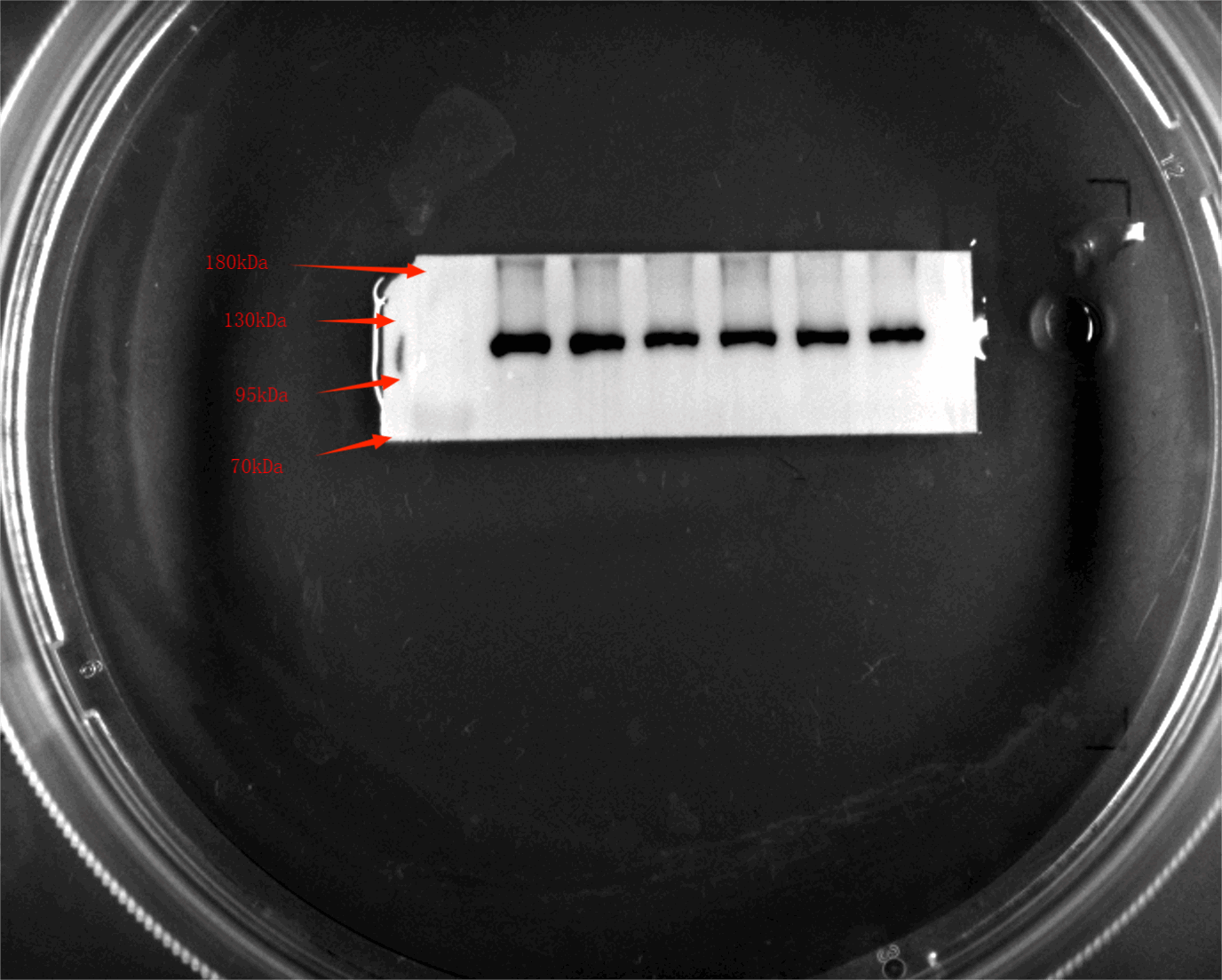

Supplement: Supplemental Information 3 [file peerj-13-19276-s003.zip › western blotting-marke/western blot-(CP IR group) membranal Cx43/1-ATPase-M(1).png]

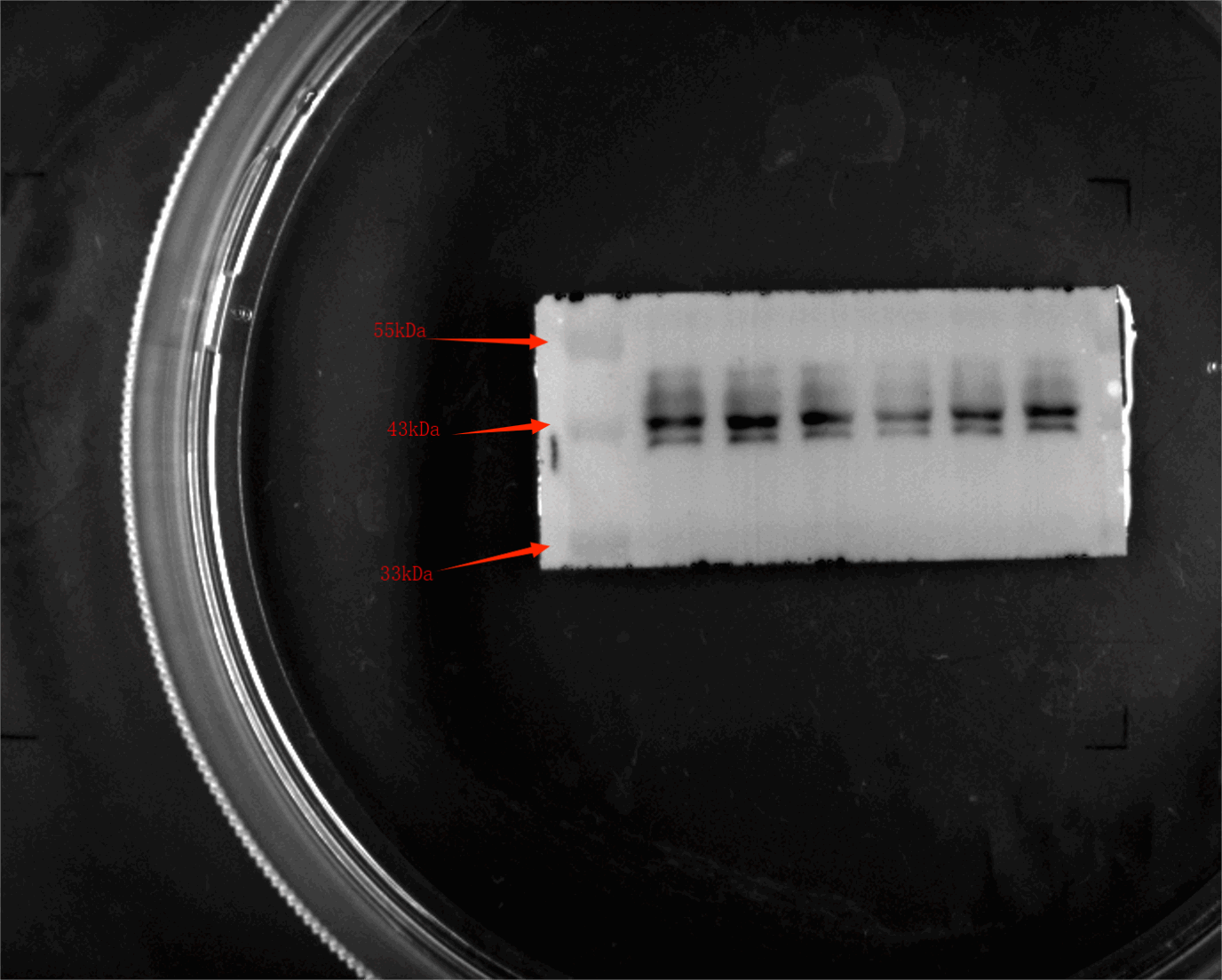

Supplement: Supplemental Information 3 [file peerj-13-19276-s003.zip › western blotting-marke/western blot-(CP IR group) membranal Cx43/1-CX43-M(1).png]

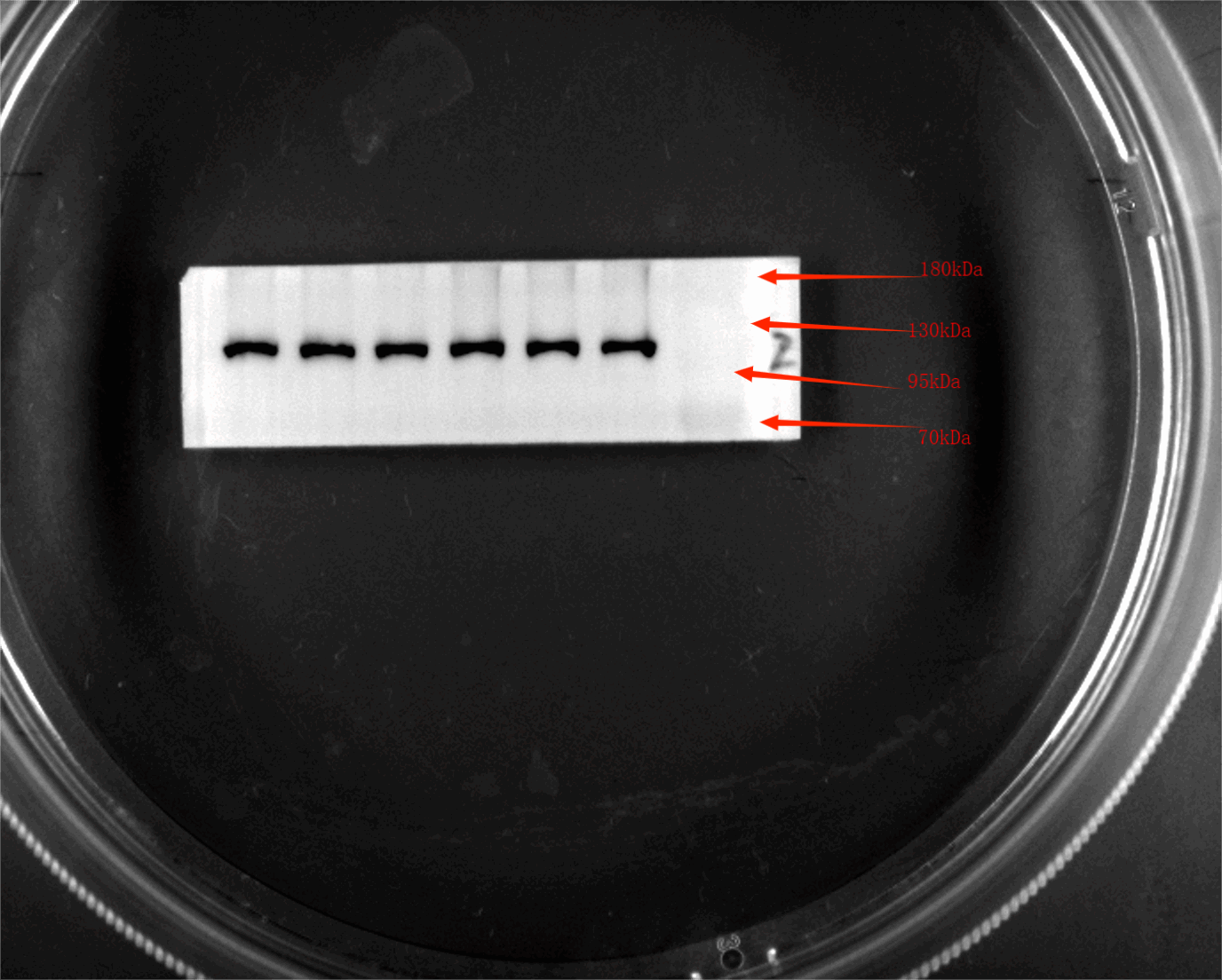

Supplement: Supplemental Information 3 [file peerj-13-19276-s003.zip › western blotting-marke/western blot-(CP IR group) membranal Cx43/2-ATPase-M(1).png]

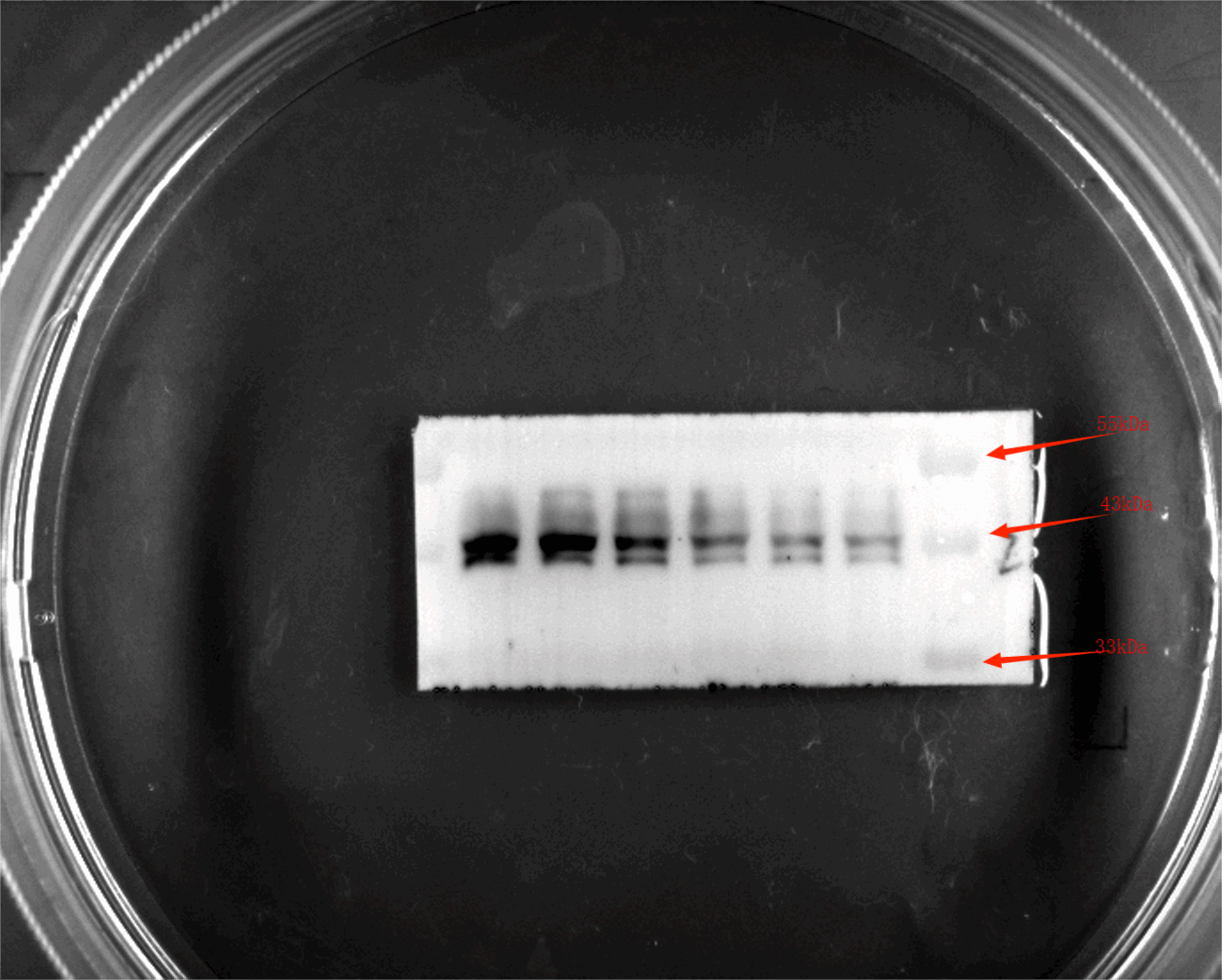

Supplement: Supplemental Information 3 [file peerj-13-19276-s003.zip › western blotting-marke/western blot-(CP IR group) membranal Cx43/2-CX43-M(1).png]

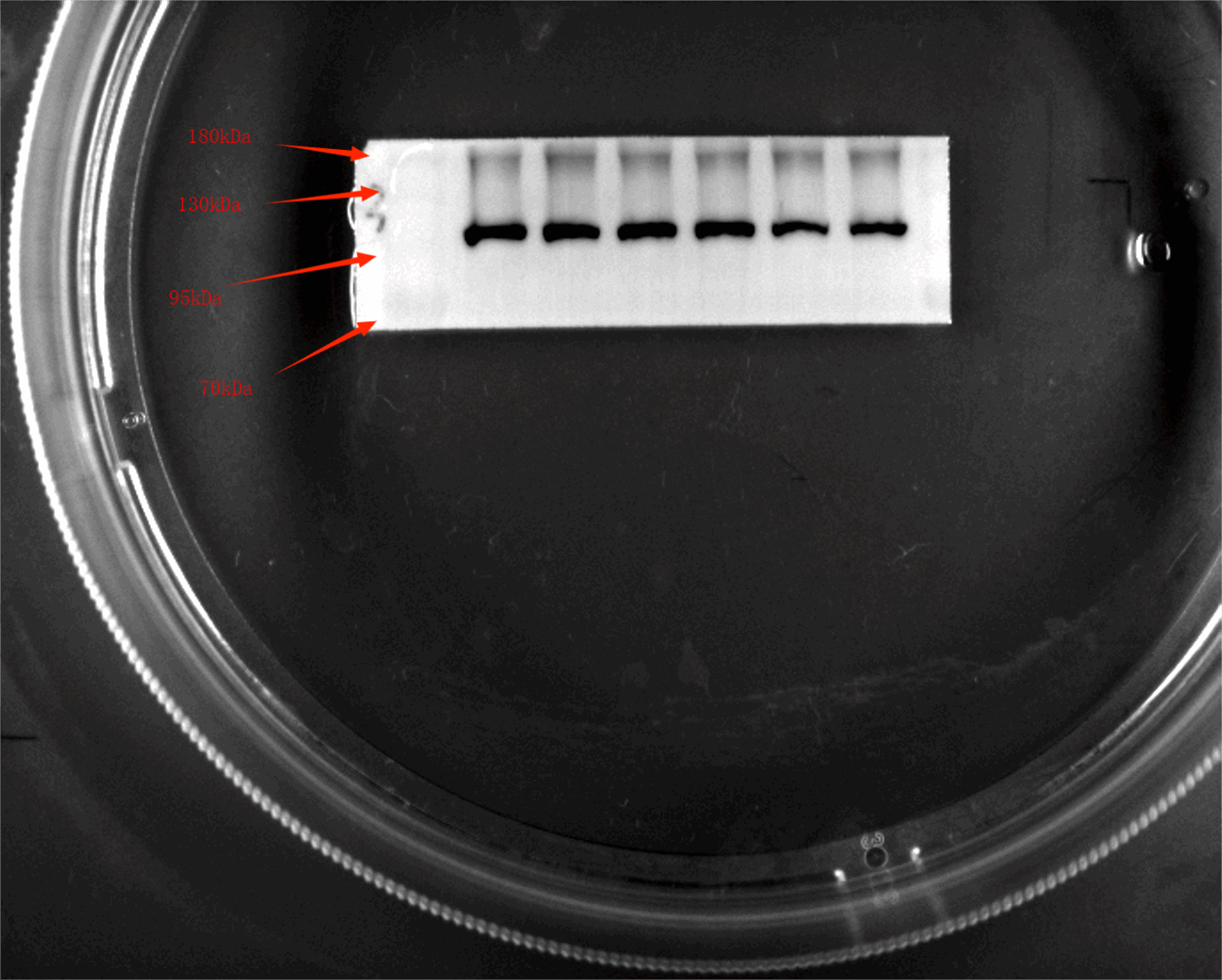

Supplement: Supplemental Information 3 [file peerj-13-19276-s003.zip › western blotting-marke/western blot-(CP IR group) membranal Cx43/3-ATPase-M(1).png]

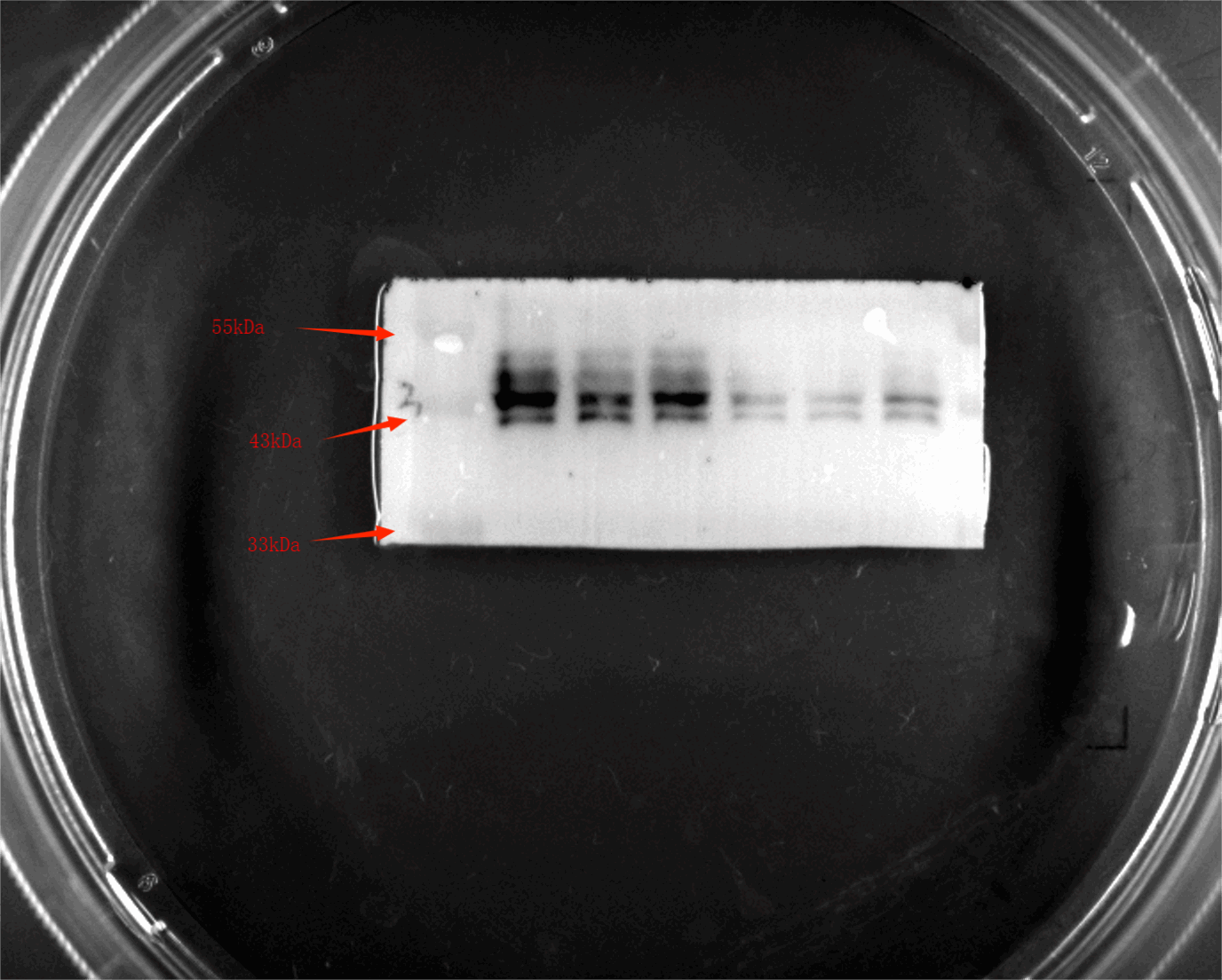

Supplement: Supplemental Information 3 [file peerj-13-19276-s003.zip › western blotting-marke/western blot-(CP IR group) membranal Cx43/3-CX43-M(1).png]

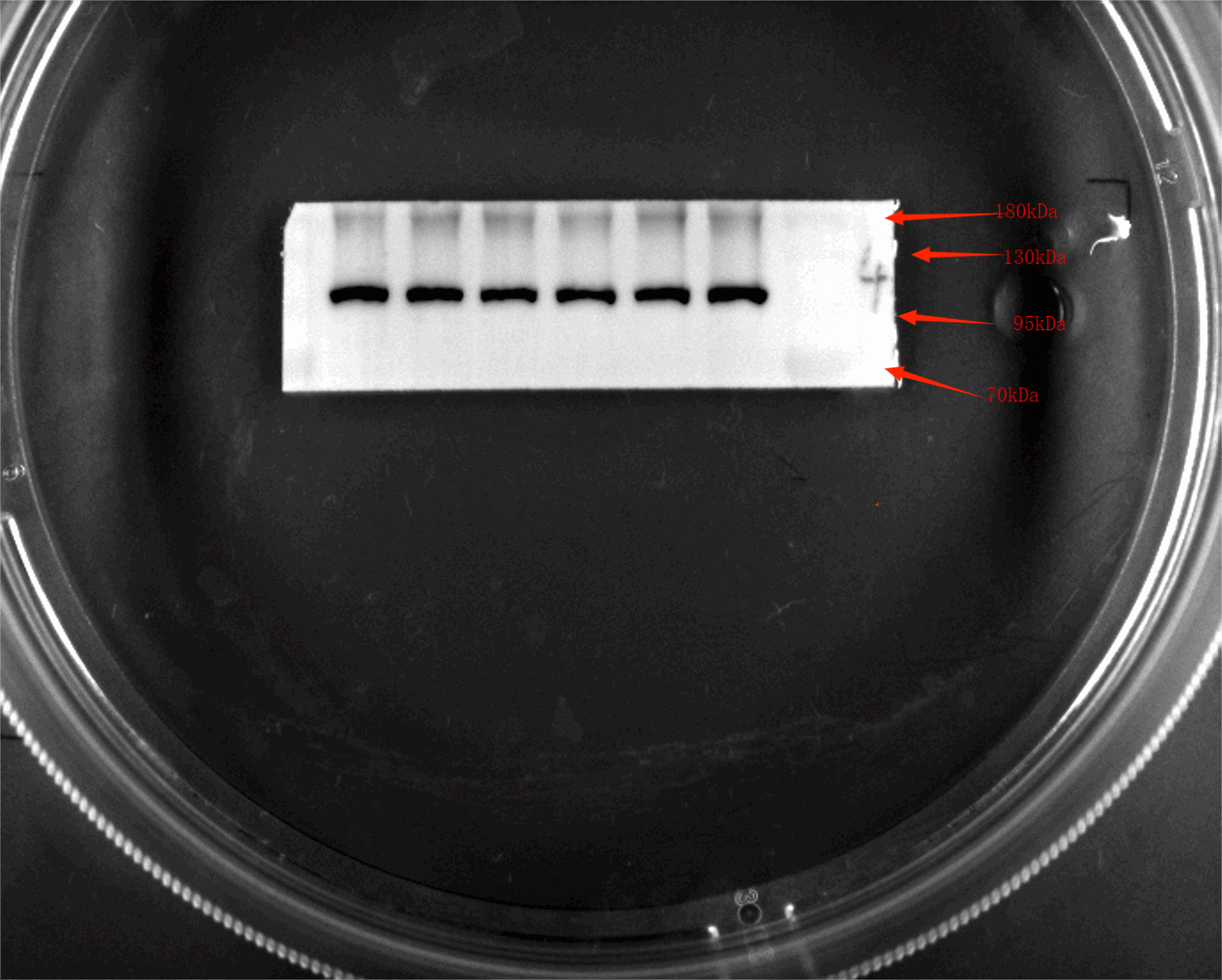

Supplement: Supplemental Information 3 [file peerj-13-19276-s003.zip › western blotting-marke/western blot-(CP IR group) membranal Cx43/4-ATPase-M-uded(1).png]

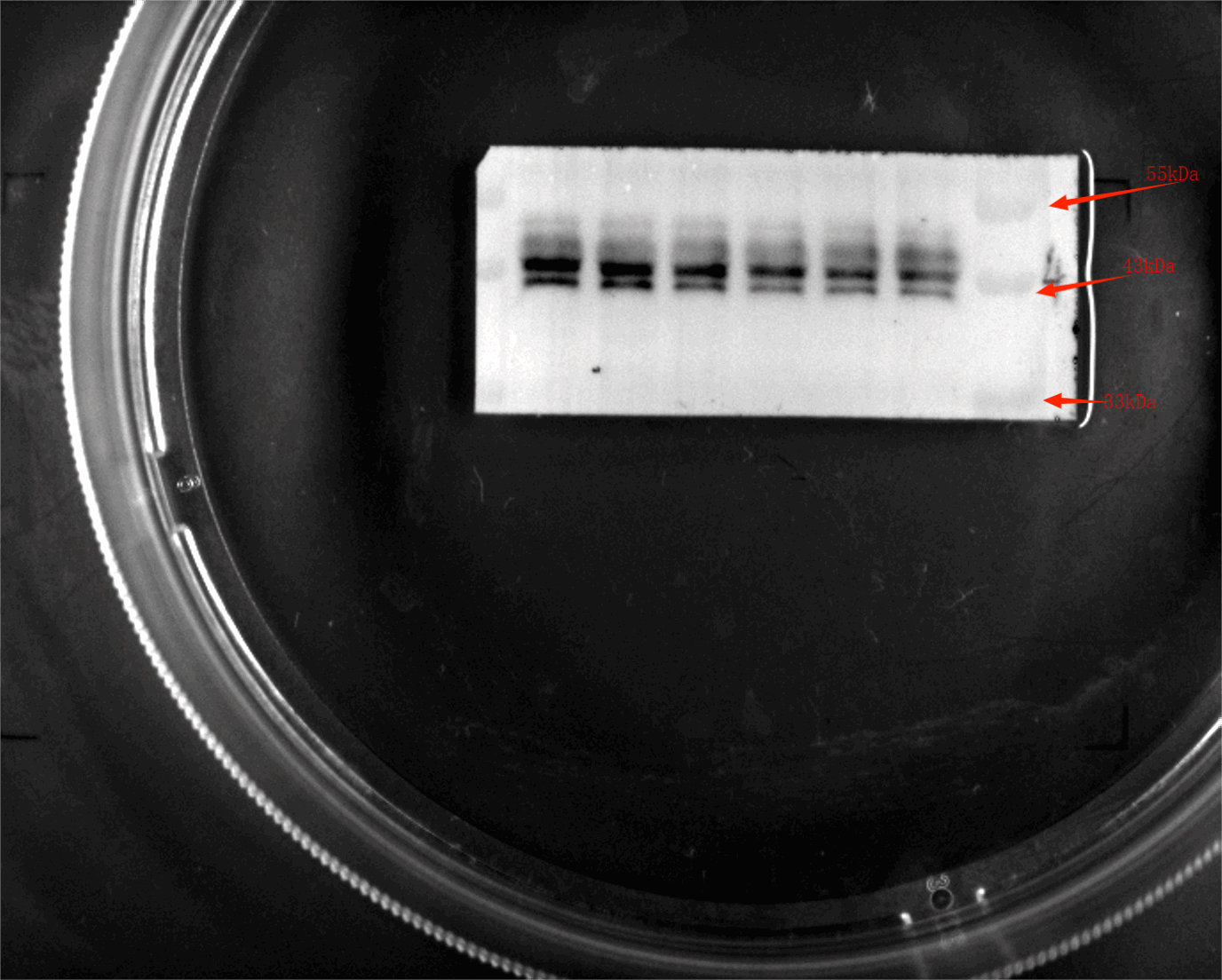

Supplement: Supplemental Information 3 [file peerj-13-19276-s003.zip › western blotting-marke/western blot-(CP IR group) membranal Cx43/4-CX43-M-used(1).png]

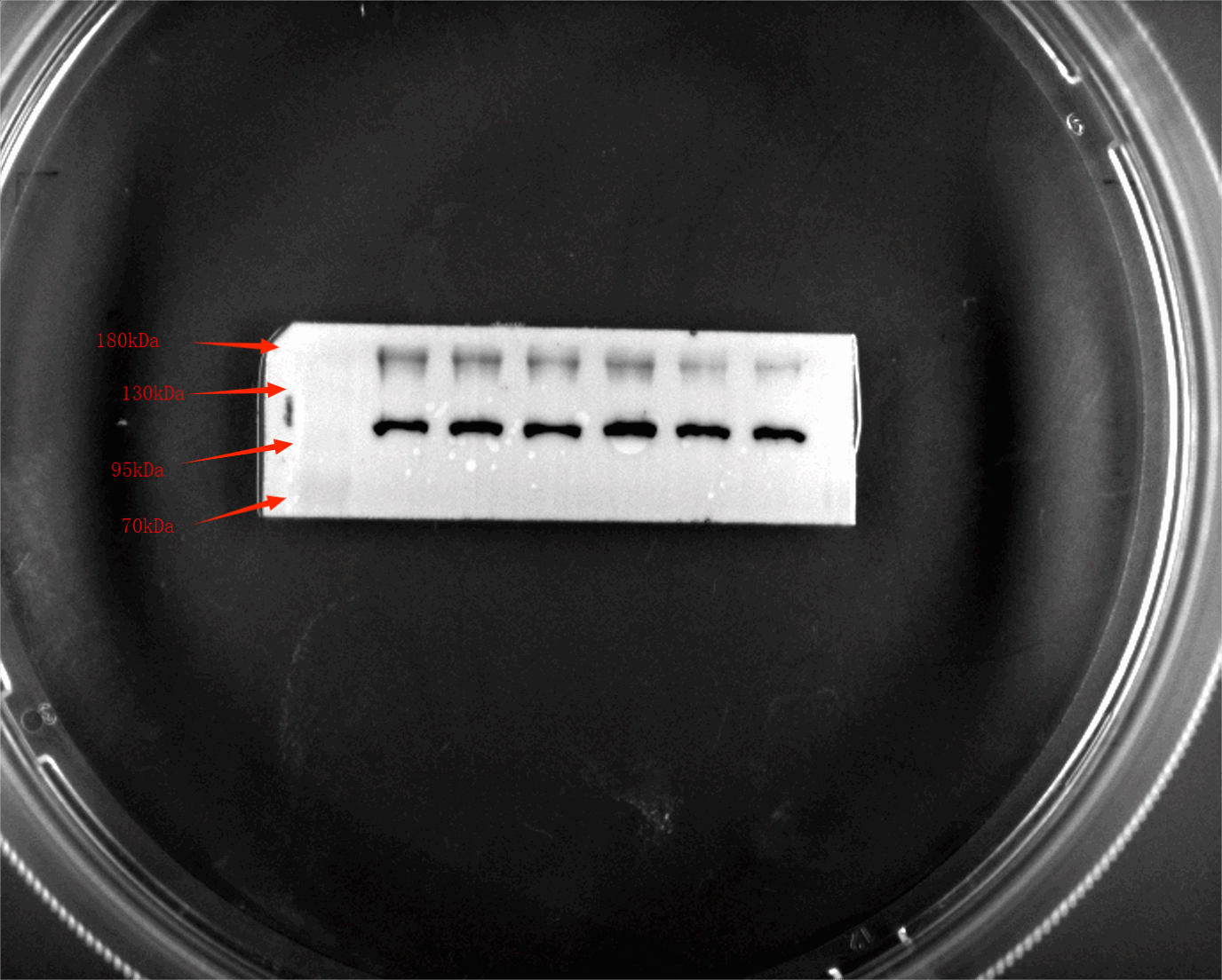

Supplement: Supplemental Information 3 [file peerj-13-19276-s003.zip › western blotting-marke/western blot-(CP IR group) membranal Cx43/5-ATPase-M(1).png]

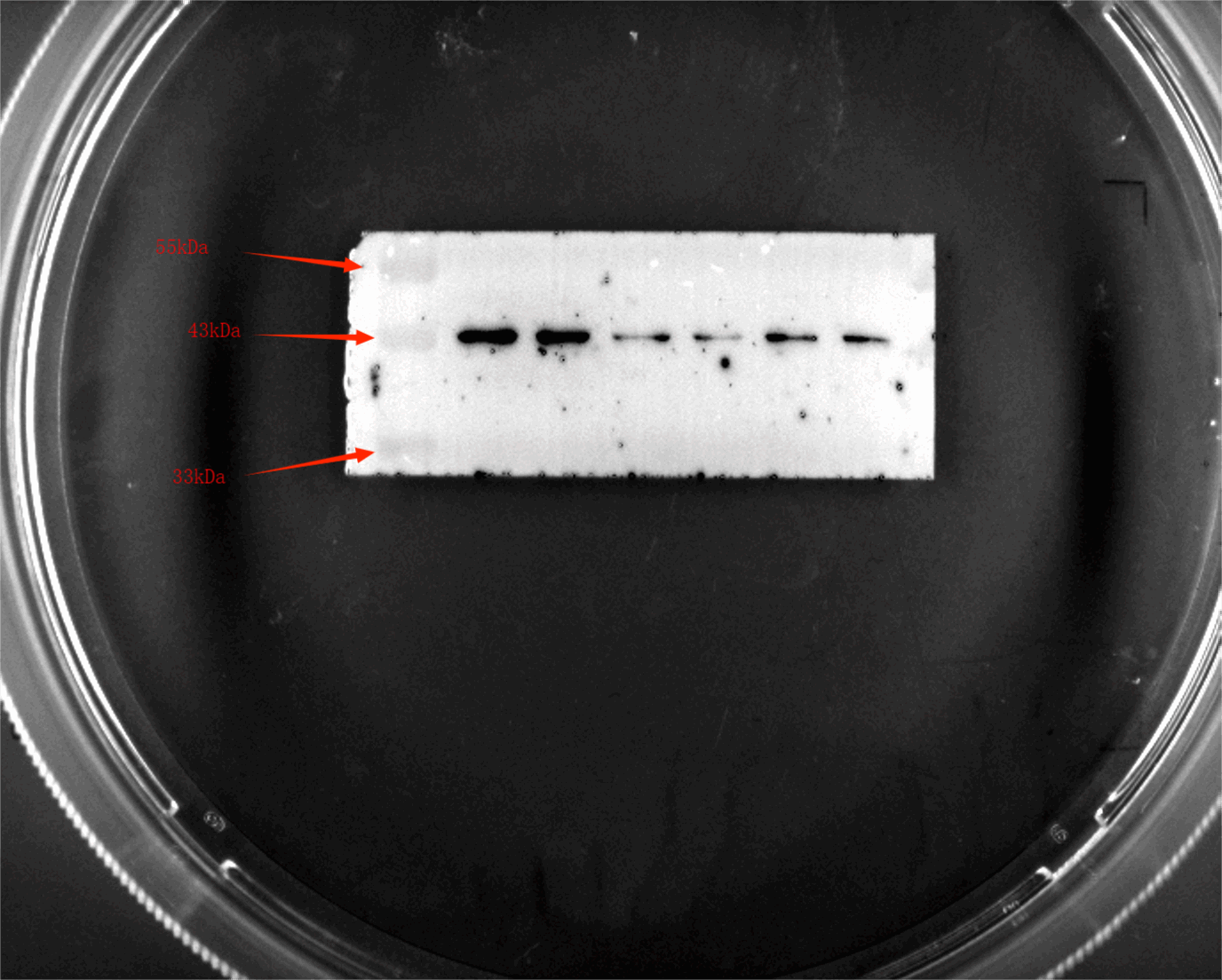

Supplement: Supplemental Information 3 [file peerj-13-19276-s003.zip › western blotting-marke/western blot-(CP IR group) membranal Cx43/5-CX43-M(1).png]

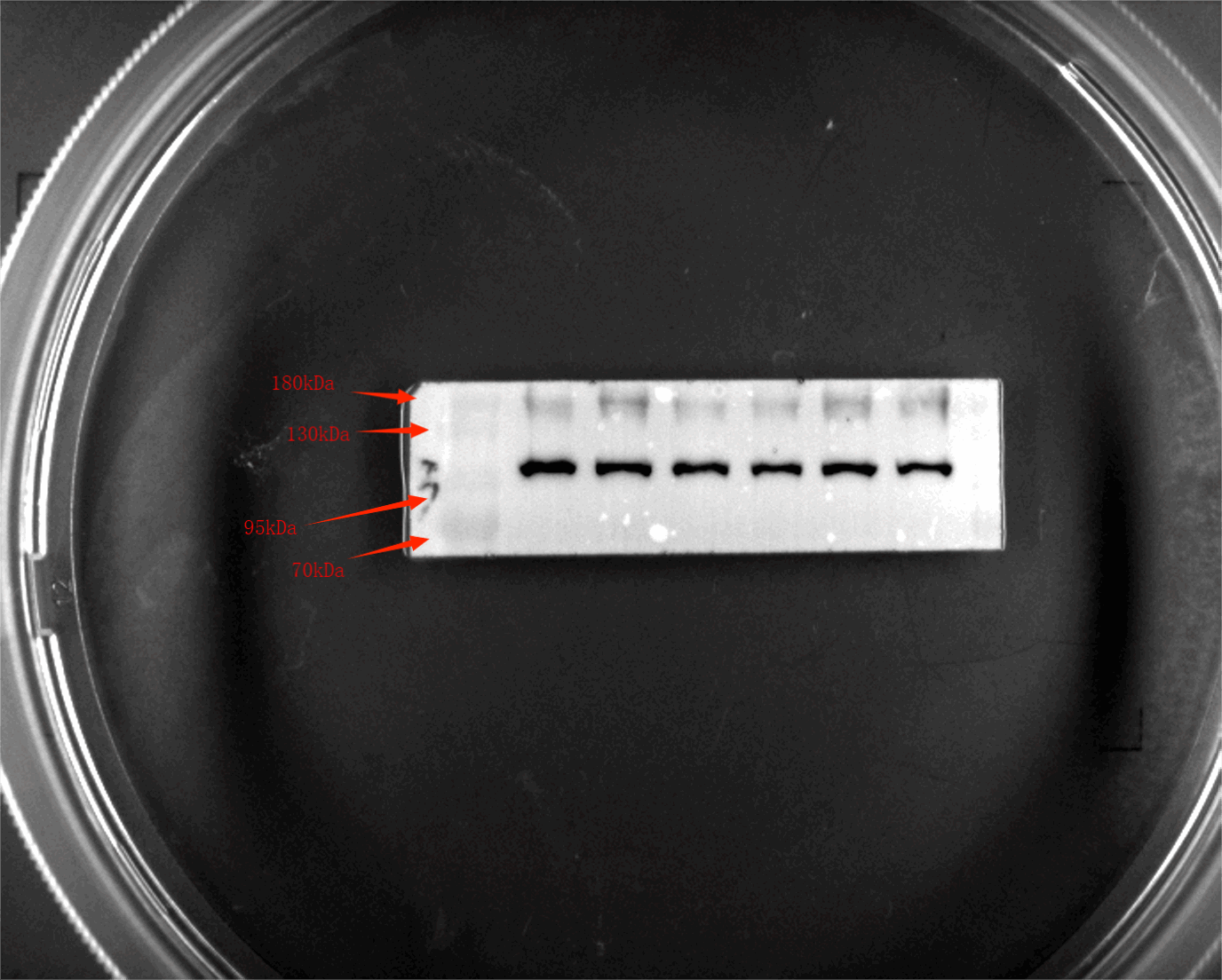

Supplement: Supplemental Information 3 [file peerj-13-19276-s003.zip › western blotting-marke/western blot-(CP IR group) membranal Cx43/6-ATPase-M(1).png]

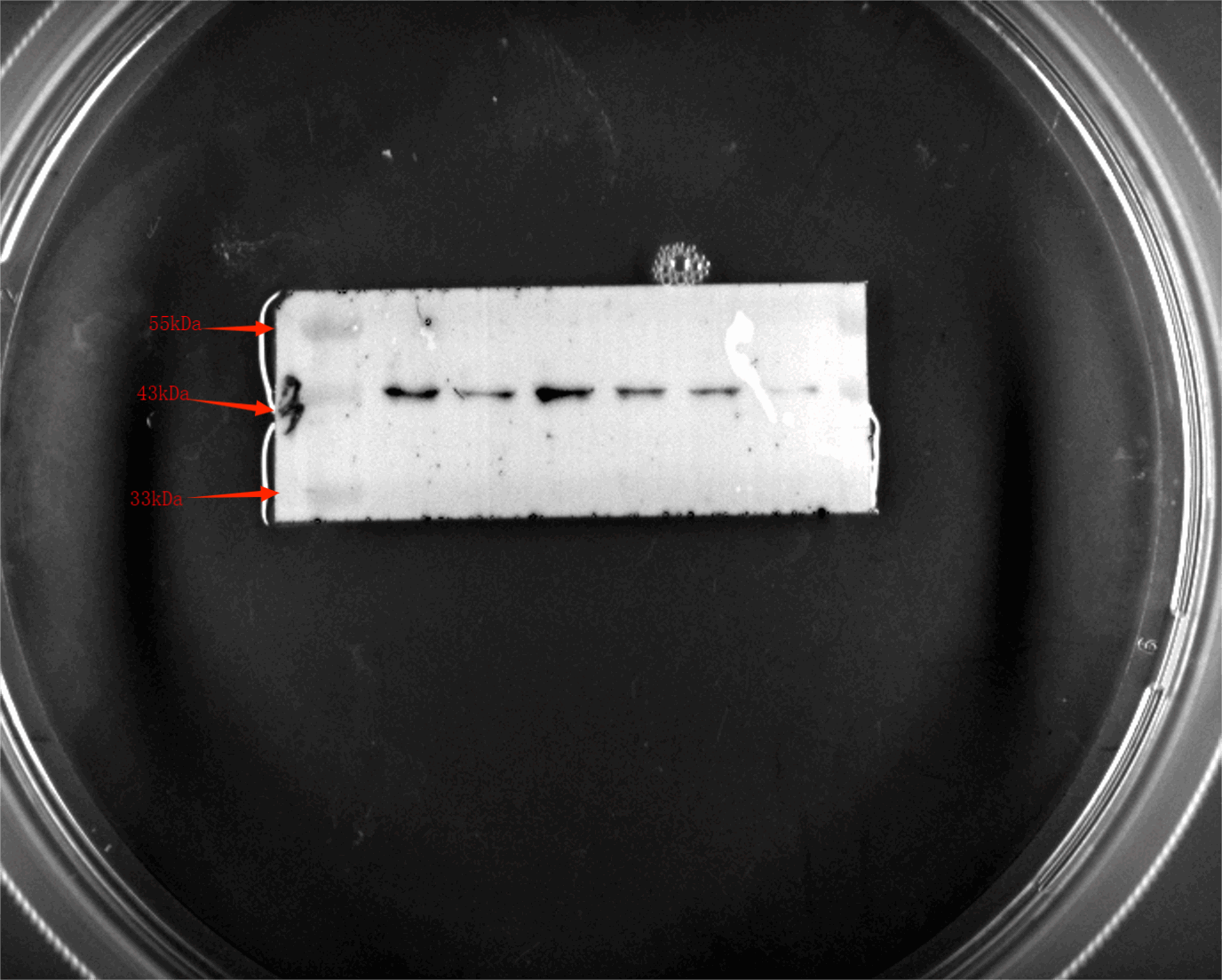

Supplement: Supplemental Information 3 [file peerj-13-19276-s003.zip › western blotting-marke/western blot-(CP IR group) membranal Cx43/6-CX43-M(1).png]

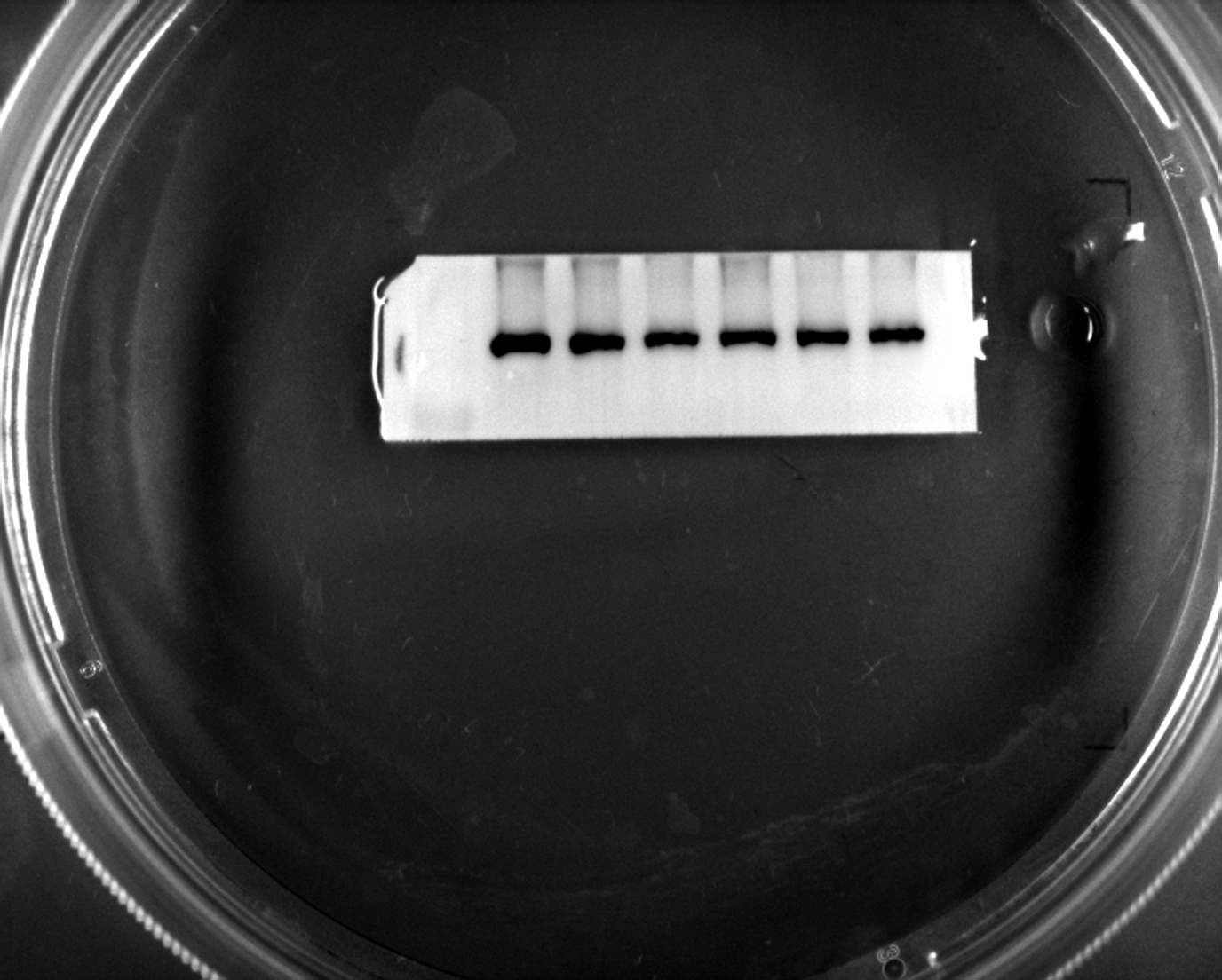

Supplement: Supplemental Information 4 [file peerj-13-19276-s004.zip › western blot-(CP IR group) Cx43membranal/western blot-(CP IR group)Cx43membranal -1/1-ATPase-M.Tif]

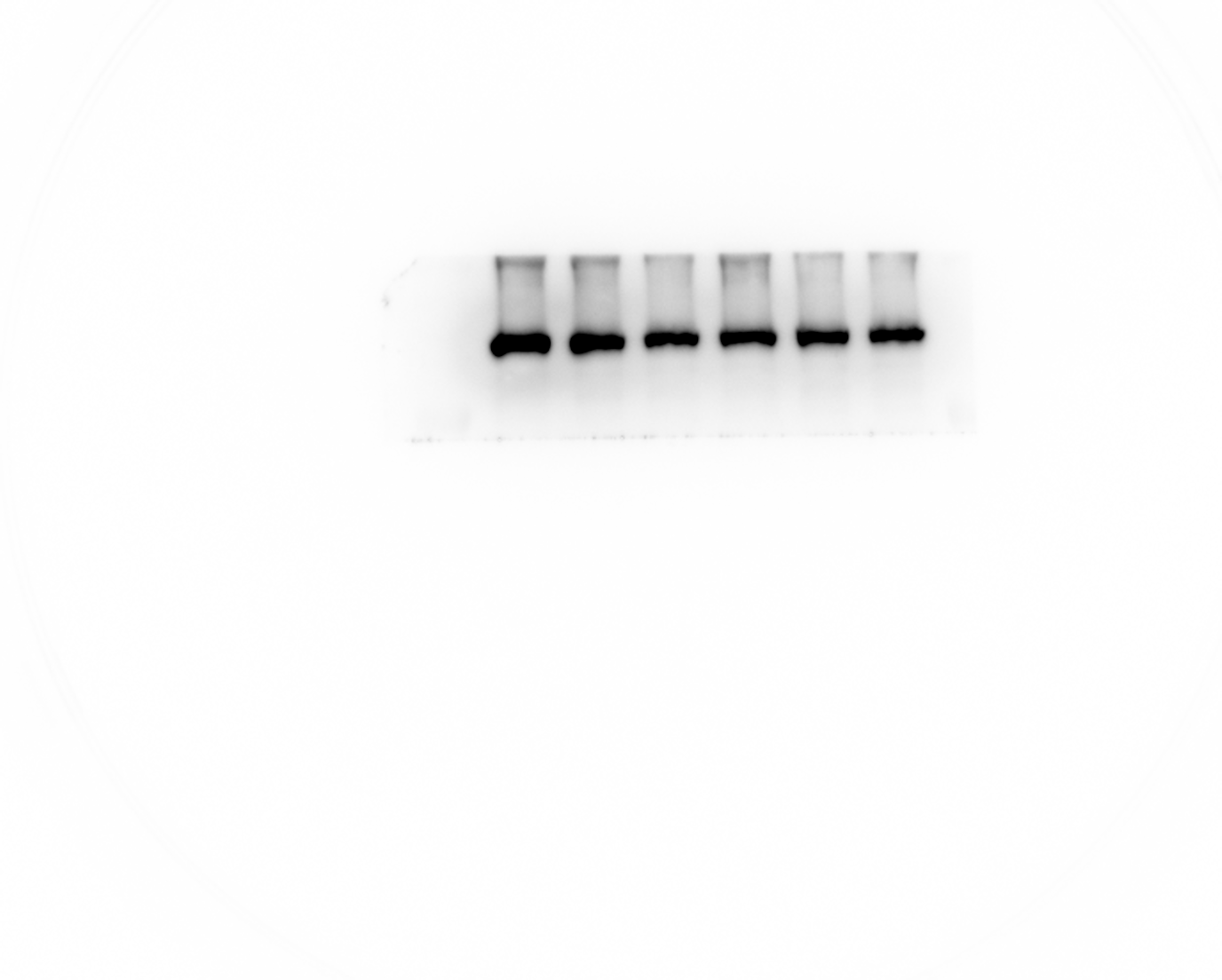

Supplement: Supplemental Information 4 [file peerj-13-19276-s004.zip › western blot-(CP IR group) Cx43membranal/western blot-(CP IR group)Cx43membranal -1/1-ATPase.Tif]
