# Supplementary figures and images for: Downregulation of EB1 impedes Cx43 localization and cardiac conduction after hypothermic ischemia-reperfusion in rats (part 2 of 5)
Source: PeerJ. 2025 Apr 14;13:e19276. doi: 10.7717/peerj.19276 (PMC12005192; doi:10.7717/peerj.19276)

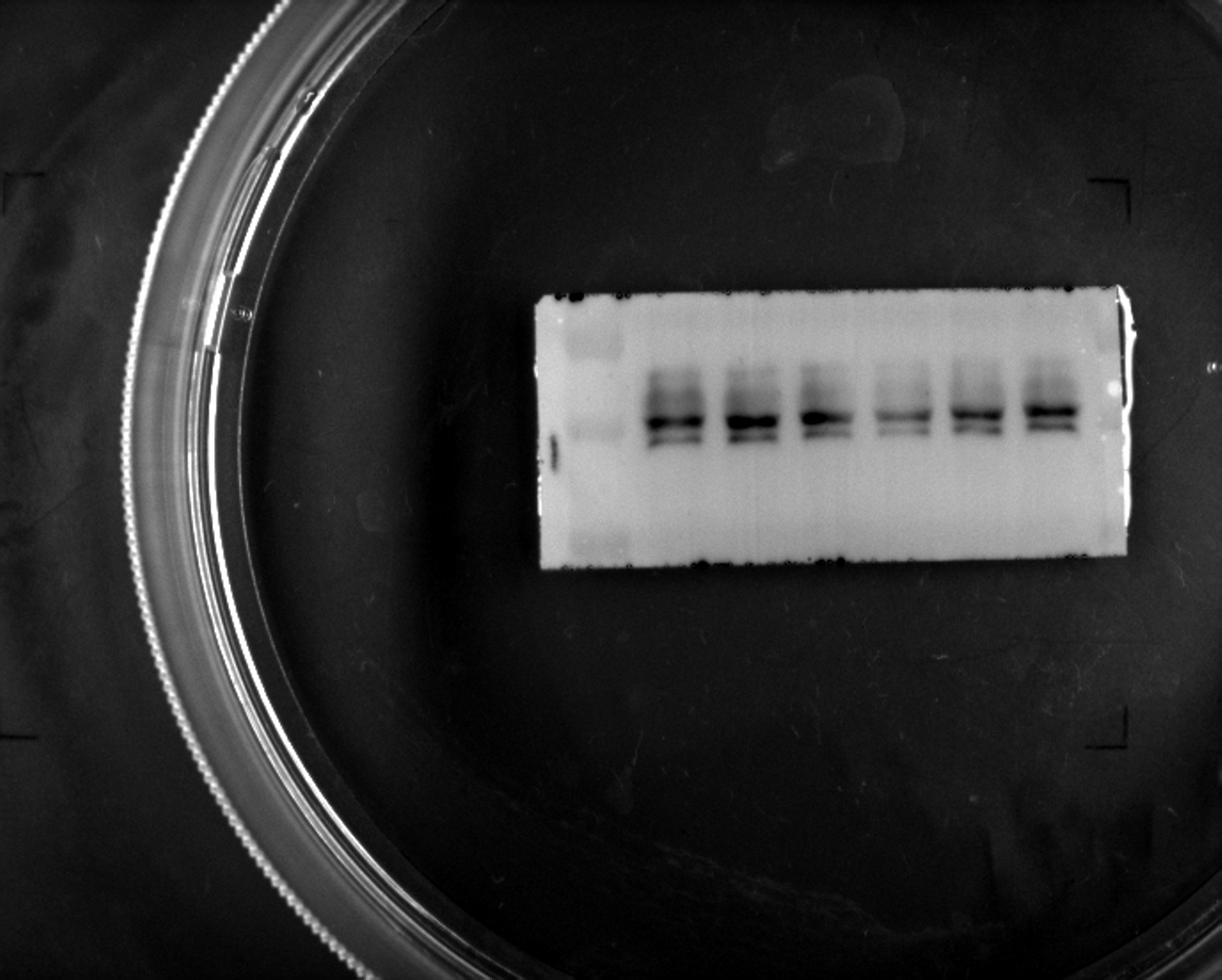

Supplement: Supplemental Information 4 [file peerj-13-19276-s004.zip › western blot-(CP IR group) Cx43membranal/western blot-(CP IR group)Cx43membranal -1/1-CX43-M.Tif]

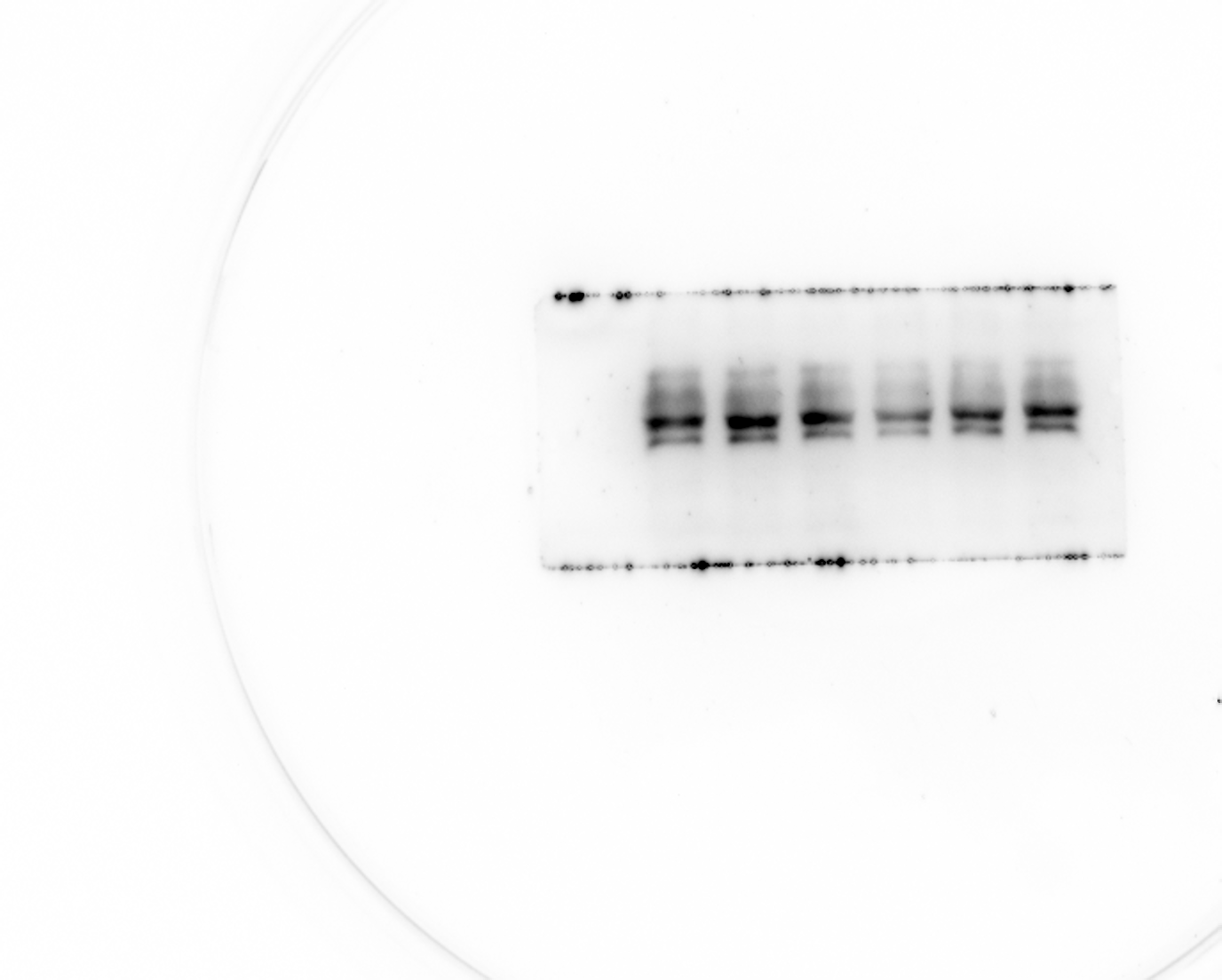

Supplement: Supplemental Information 4 [file peerj-13-19276-s004.zip › western blot-(CP IR group) Cx43membranal/western blot-(CP IR group)Cx43membranal -1/1-CX43.Tif]

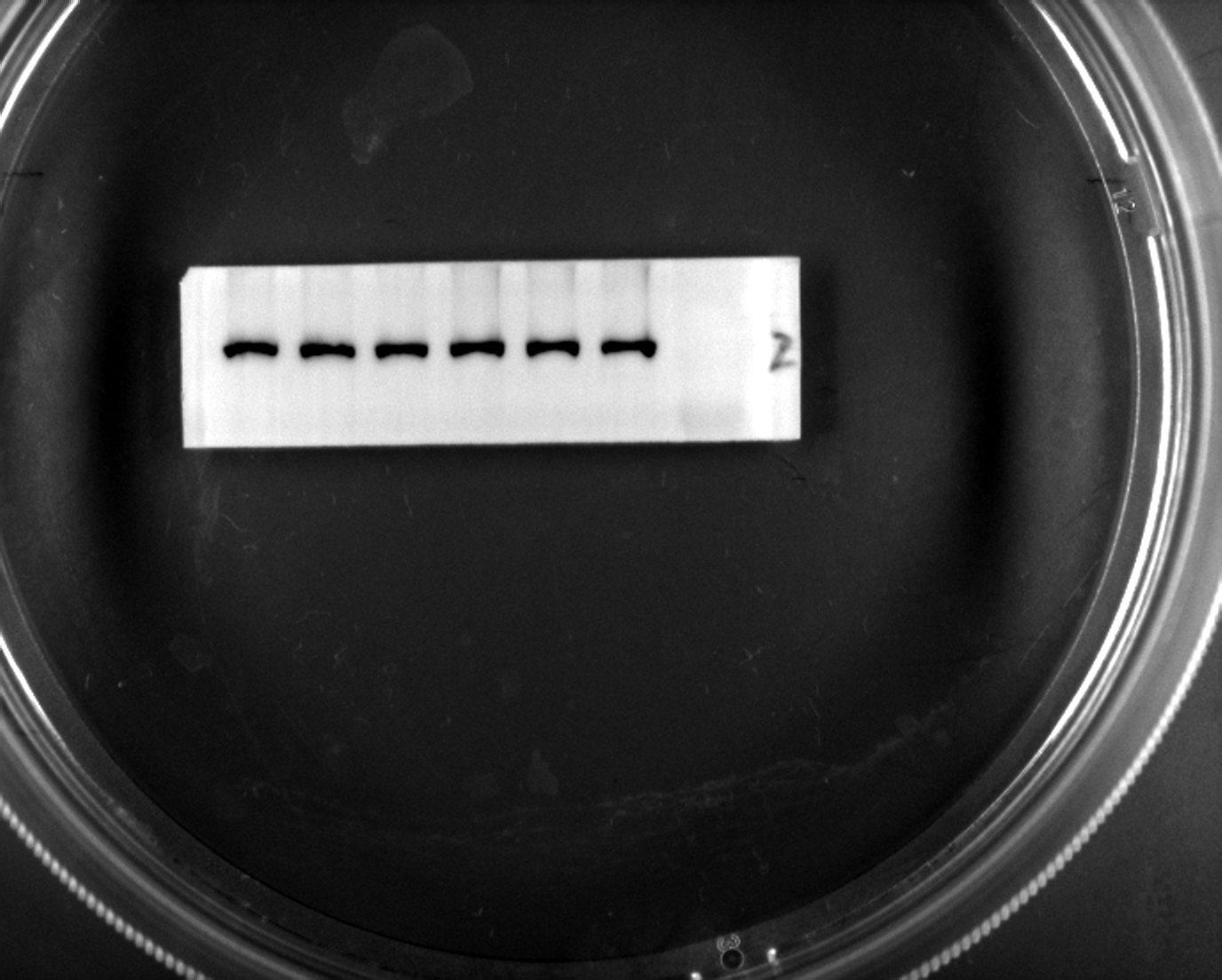

Supplement: Supplemental Information 4 [file peerj-13-19276-s004.zip › western blot-(CP IR group) Cx43membranal/western blot-(CP IR group)Cx43membranal -1/2-ATPase-M.Tif]

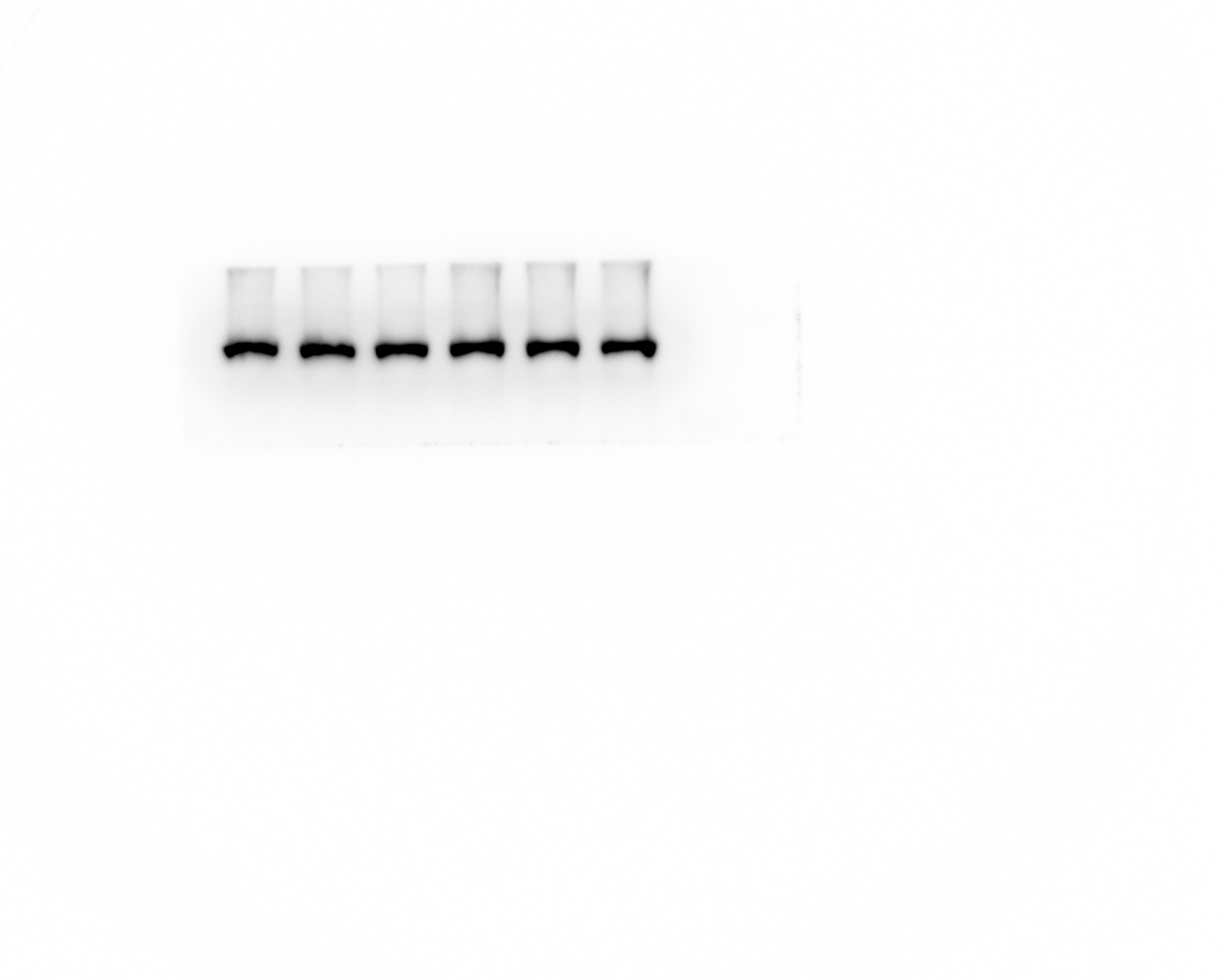

Supplement: Supplemental Information 4 [file peerj-13-19276-s004.zip › western blot-(CP IR group) Cx43membranal/western blot-(CP IR group)Cx43membranal -1/2-ATPase.Tif]

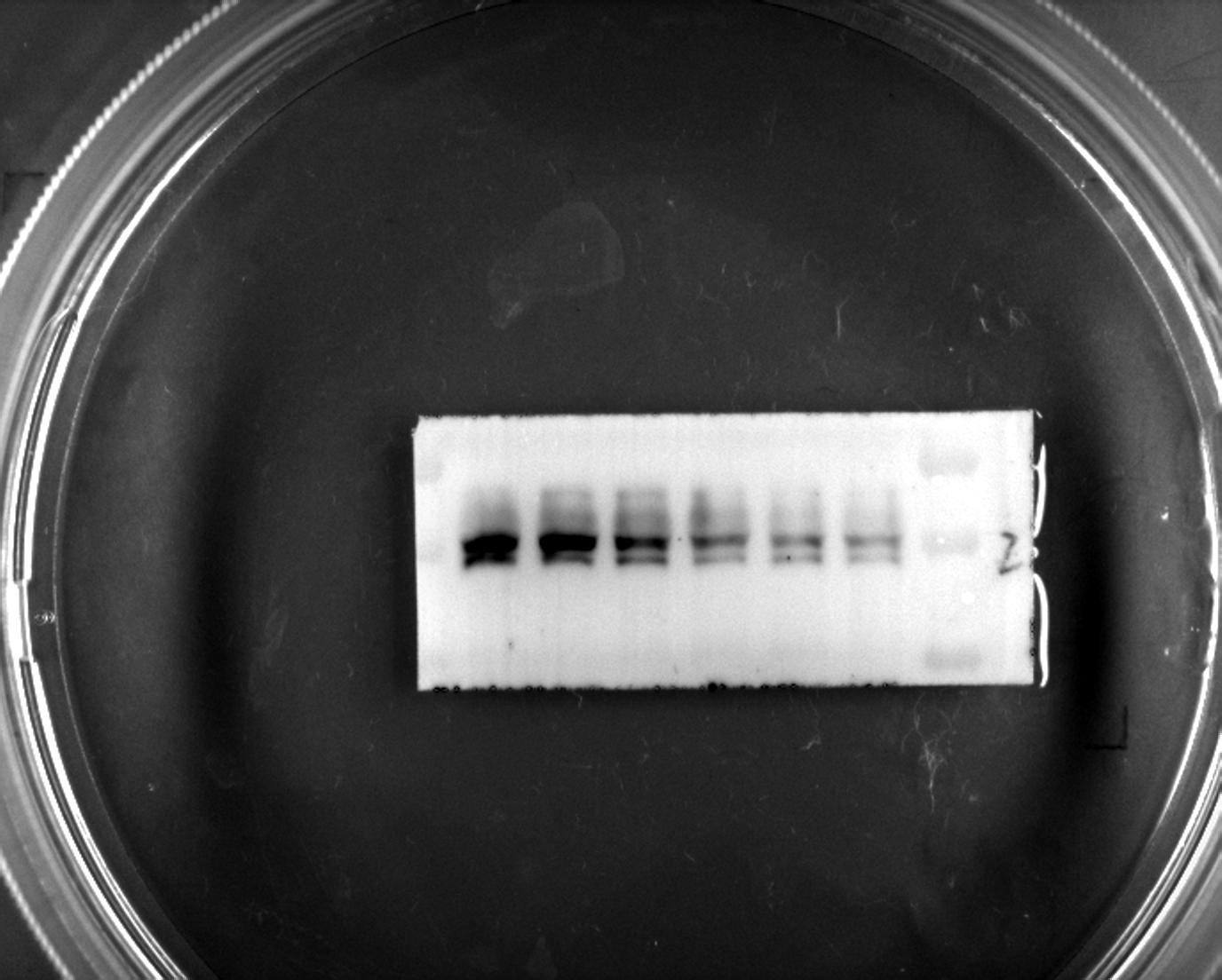

Supplement: Supplemental Information 4 [file peerj-13-19276-s004.zip › western blot-(CP IR group) Cx43membranal/western blot-(CP IR group)Cx43membranal -1/2-CX43-M.Tif]

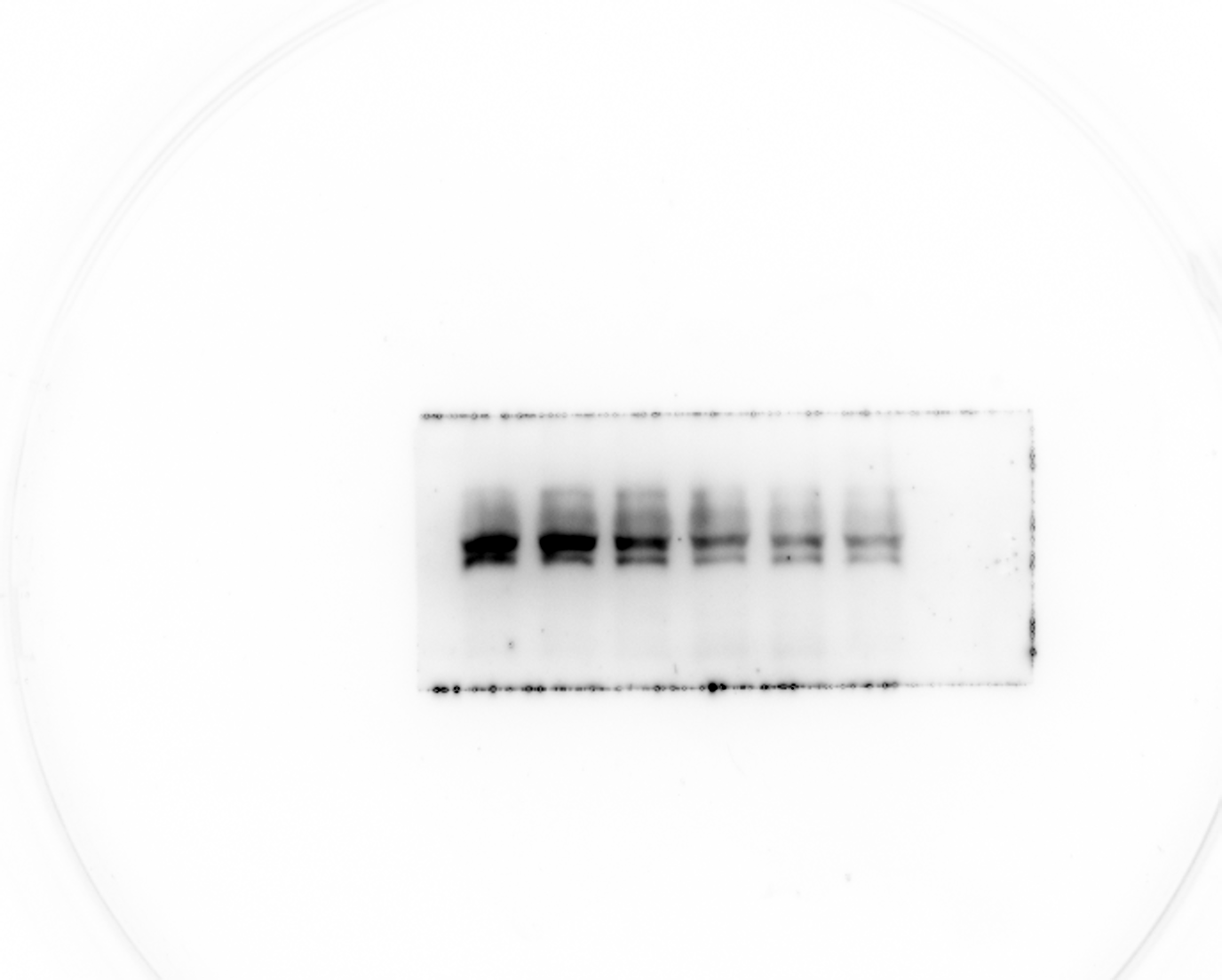

Supplement: Supplemental Information 4 [file peerj-13-19276-s004.zip › western blot-(CP IR group) Cx43membranal/western blot-(CP IR group)Cx43membranal -1/2-CX43.Tif]

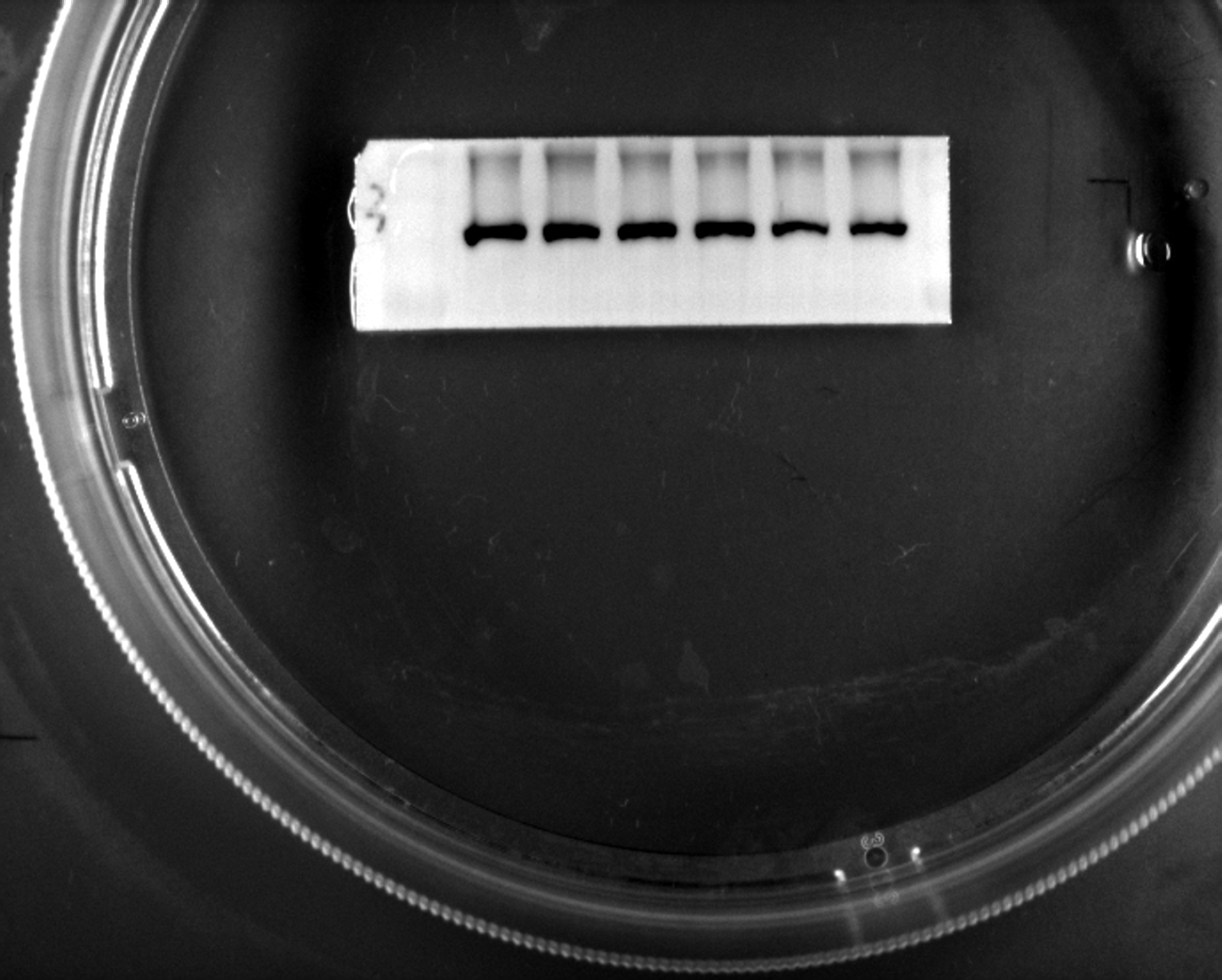

Supplement: Supplemental Information 4 [file peerj-13-19276-s004.zip › western blot-(CP IR group) Cx43membranal/western blot-(CP IR group)Cx43membranal -1/3-ATPase-M.Tif]

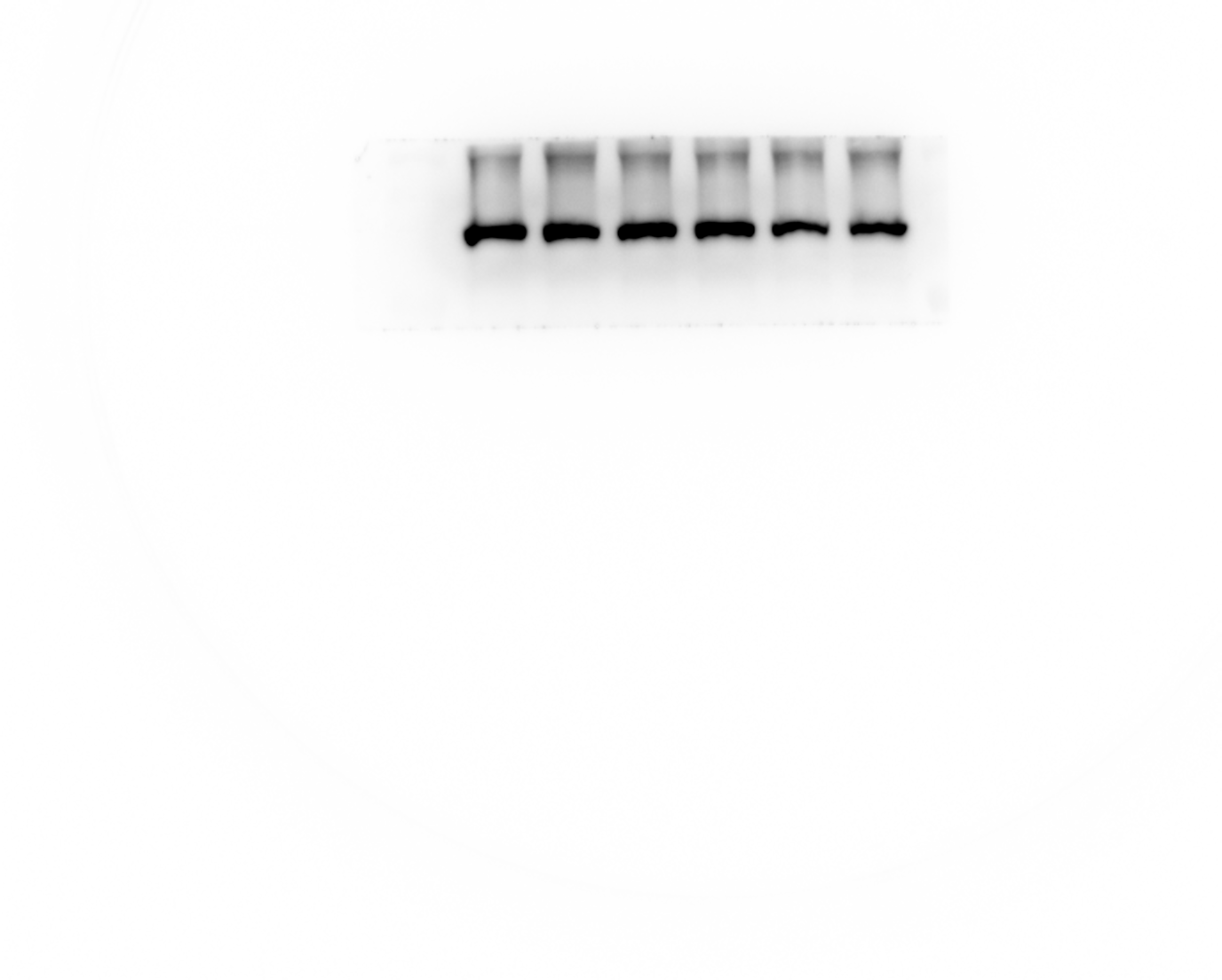

Supplement: Supplemental Information 4 [file peerj-13-19276-s004.zip › western blot-(CP IR group) Cx43membranal/western blot-(CP IR group)Cx43membranal -1/3-ATPase.Tif]

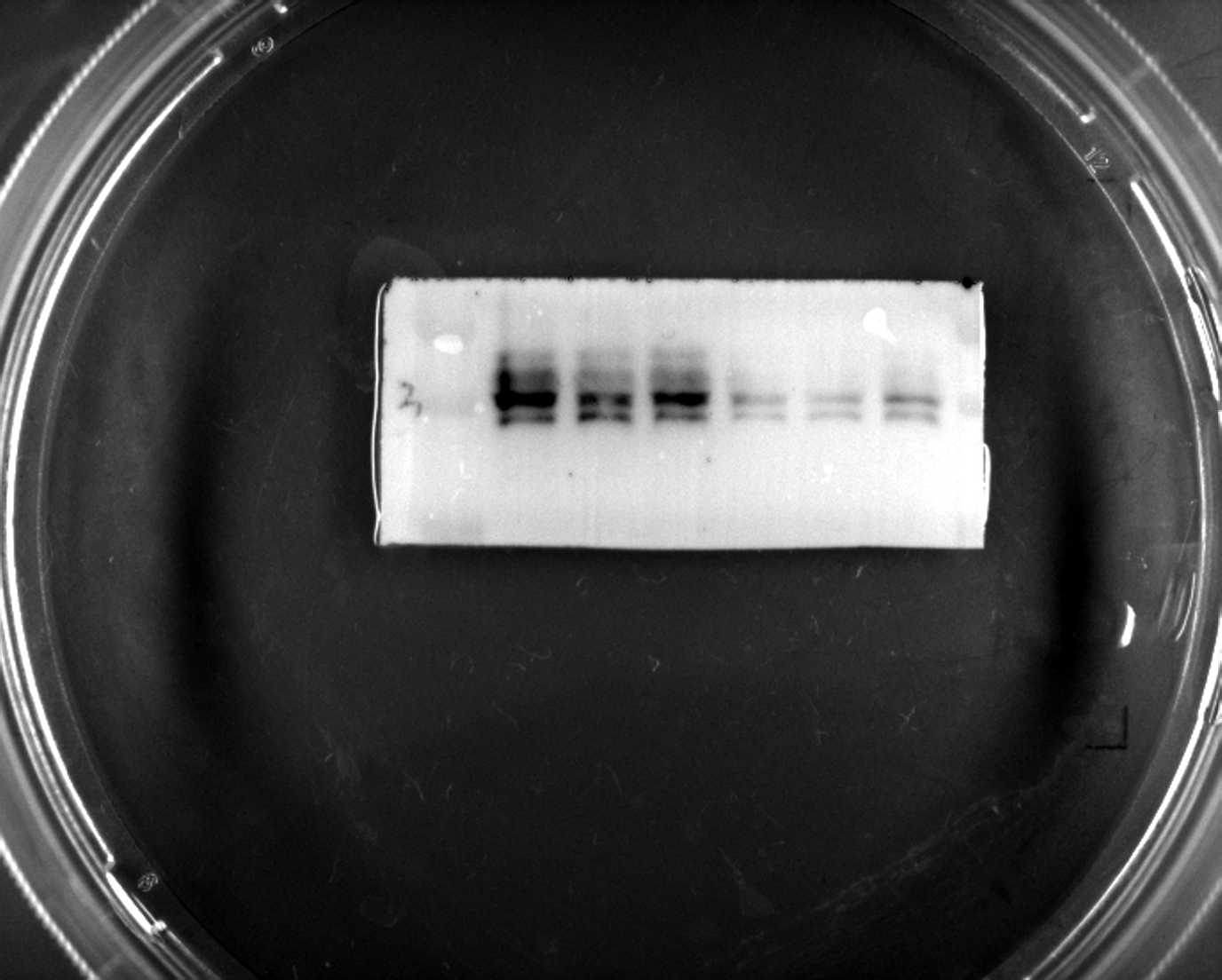

Supplement: Supplemental Information 4 [file peerj-13-19276-s004.zip › western blot-(CP IR group) Cx43membranal/western blot-(CP IR group)Cx43membranal -1/3-CX43-M.Tif]

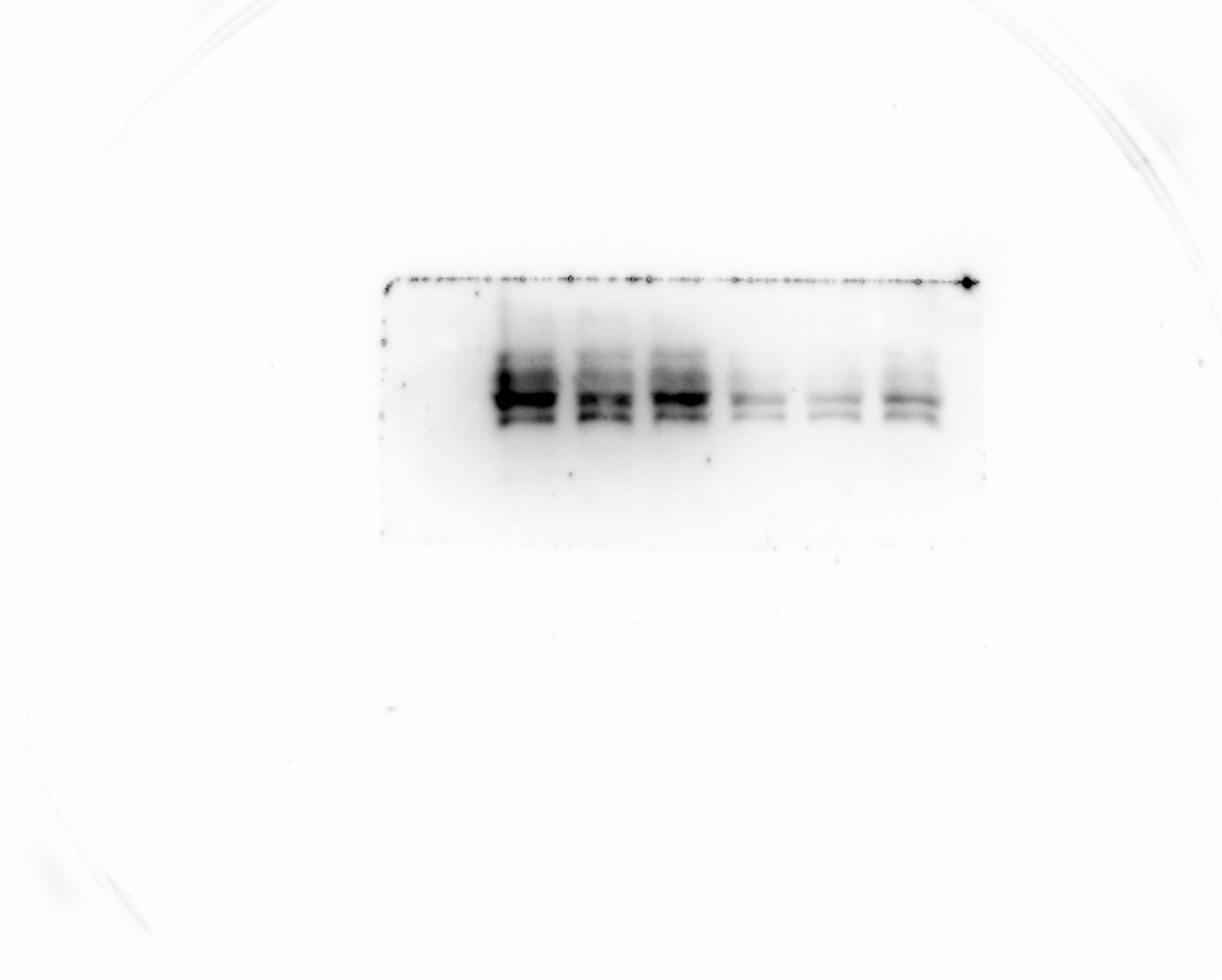

Supplement: Supplemental Information 4 [file peerj-13-19276-s004.zip › western blot-(CP IR group) Cx43membranal/western blot-(CP IR group)Cx43membranal -1/3-CX43.Tif]

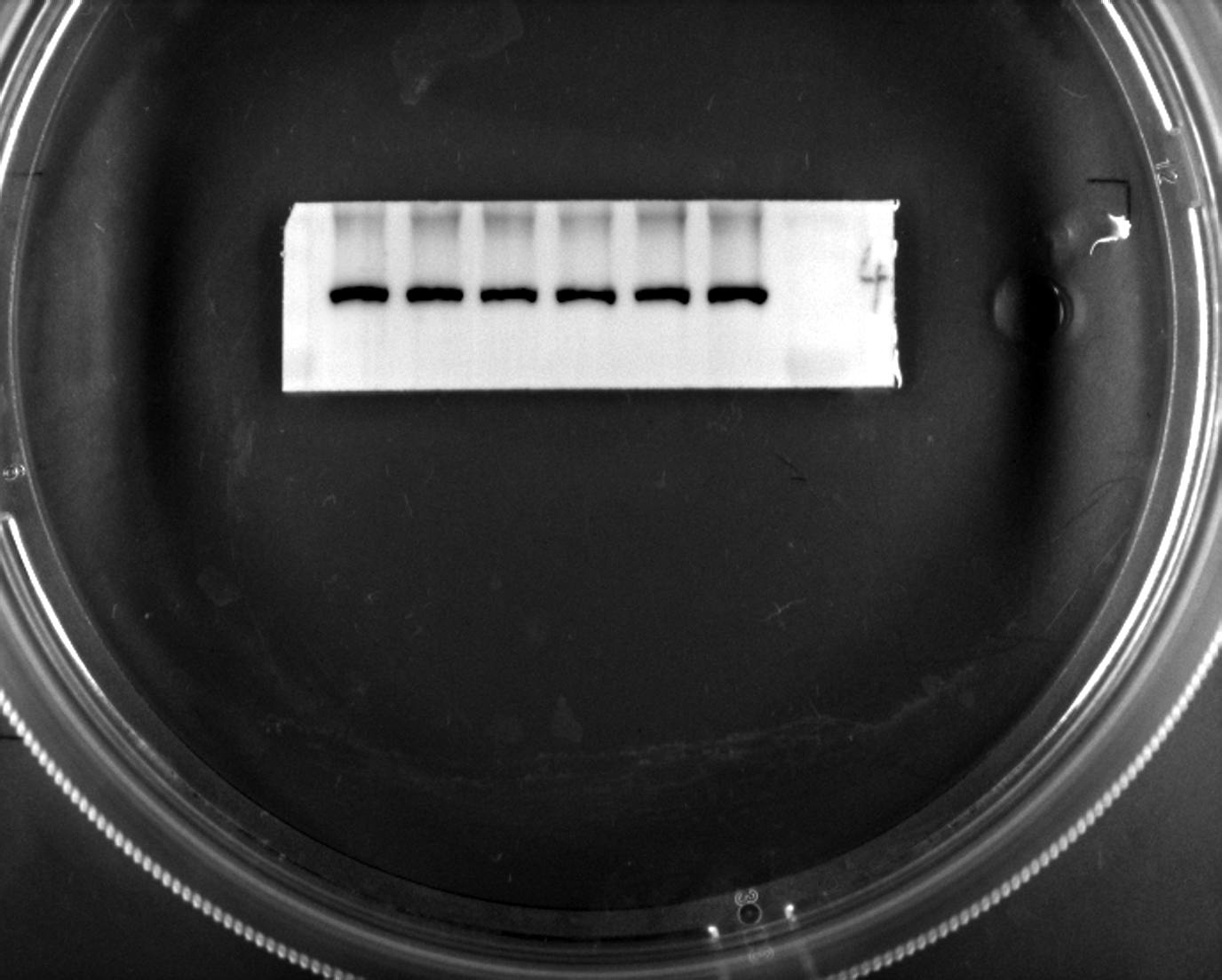

Supplement: Supplemental Information 4 [file peerj-13-19276-s004.zip › western blot-(CP IR group) Cx43membranal/western blot-(CP IR group)Cx43membranal -1/4-ATPase-M-uded.Tif]

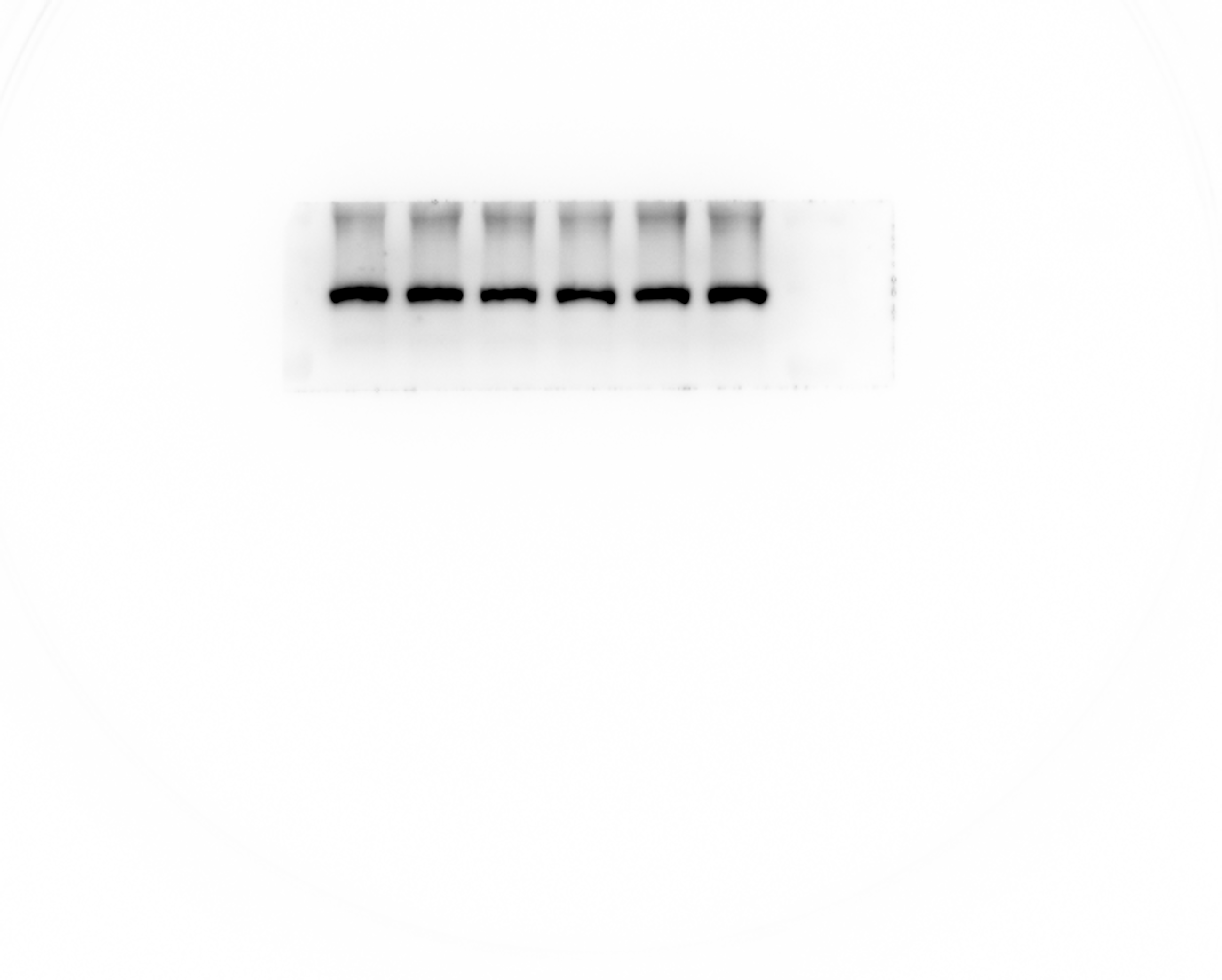

Supplement: Supplemental Information 4 [file peerj-13-19276-s004.zip › western blot-(CP IR group) Cx43membranal/western blot-(CP IR group)Cx43membranal -1/4-ATPase-used.Tif]

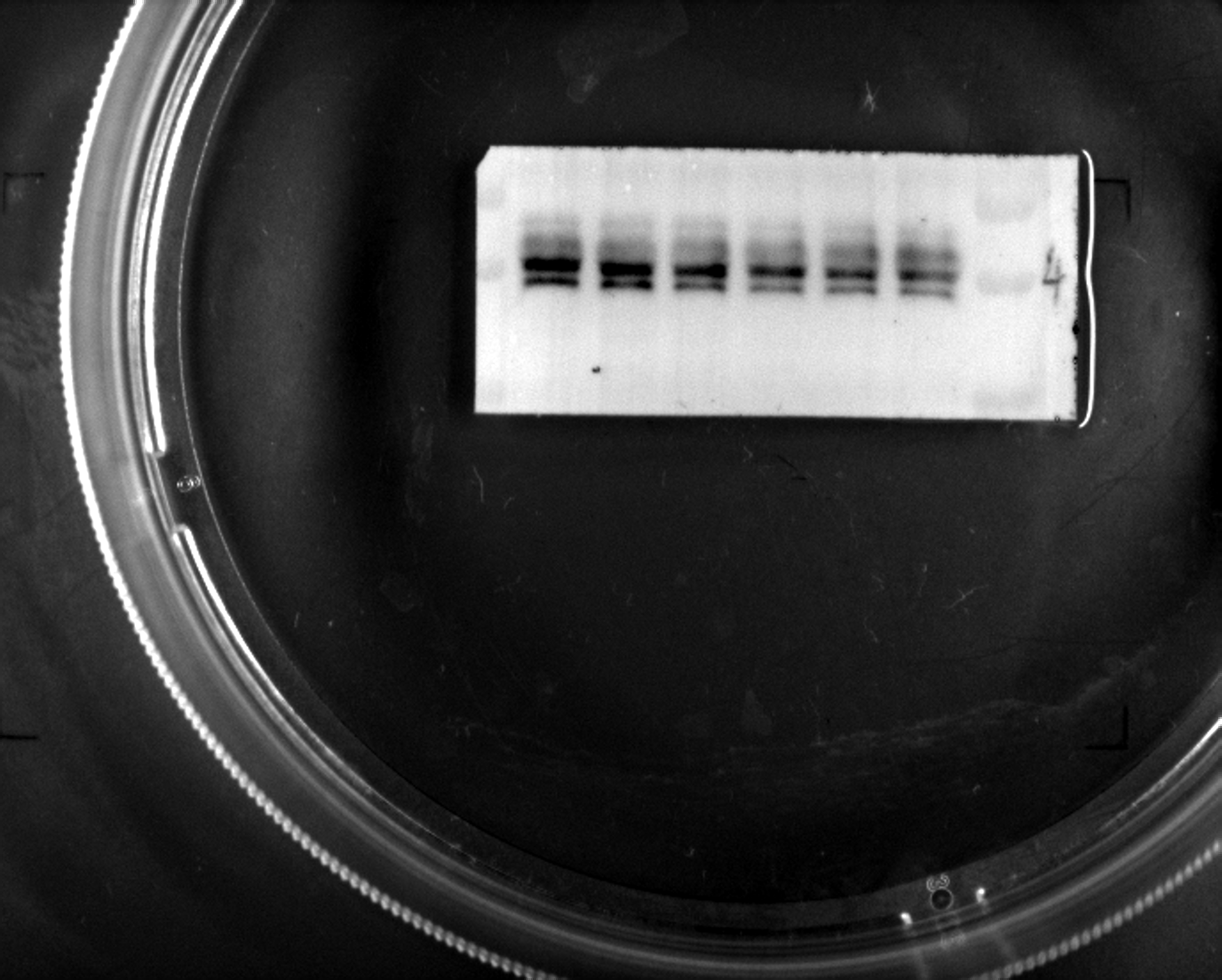

Supplement: Supplemental Information 4 [file peerj-13-19276-s004.zip › western blot-(CP IR group) Cx43membranal/western blot-(CP IR group)Cx43membranal -1/4-CX43-M-used.Tif]

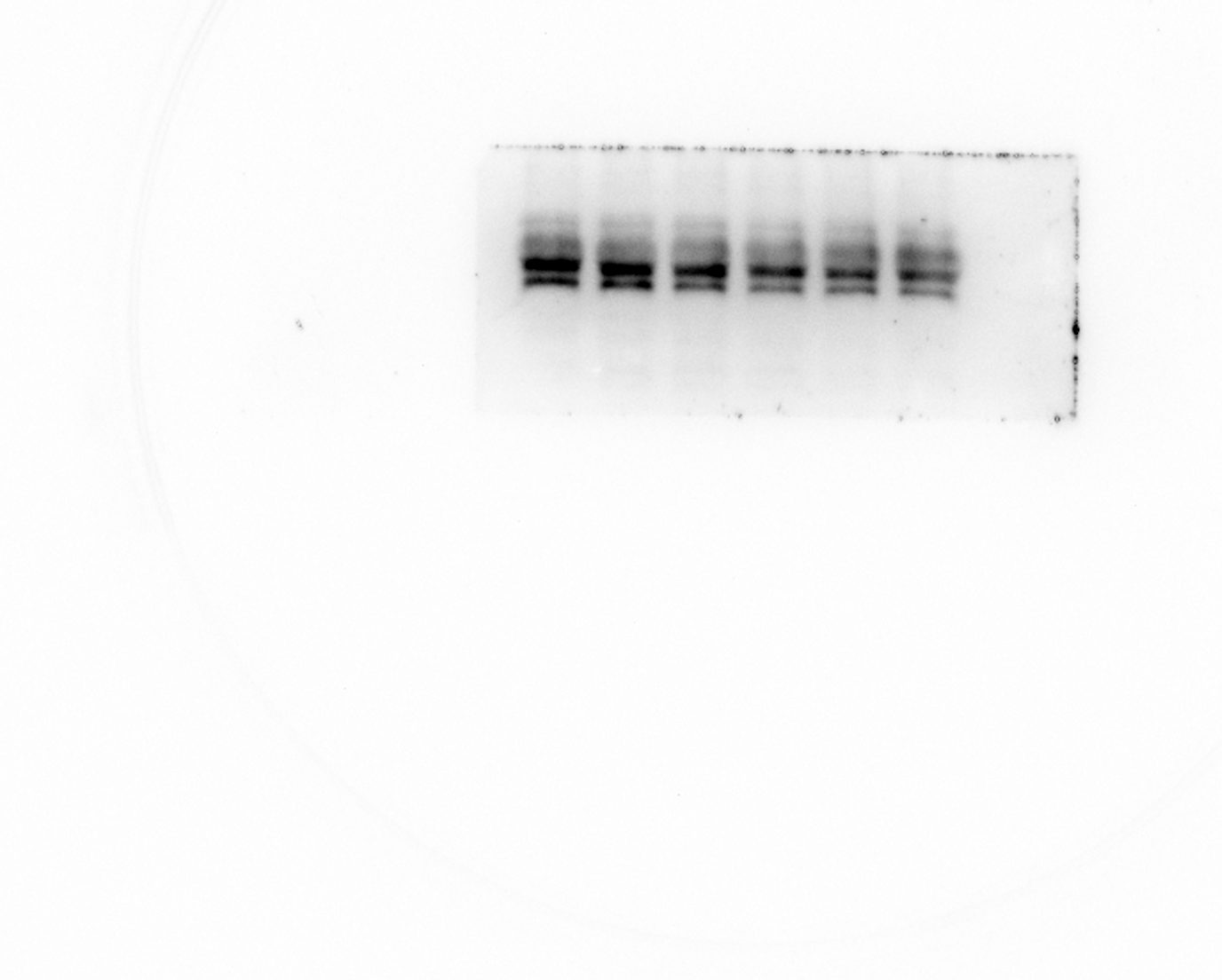

Supplement: Supplemental Information 4 [file peerj-13-19276-s004.zip › western blot-(CP IR group) Cx43membranal/western blot-(CP IR group)Cx43membranal -1/4-CX43-used.Tif]

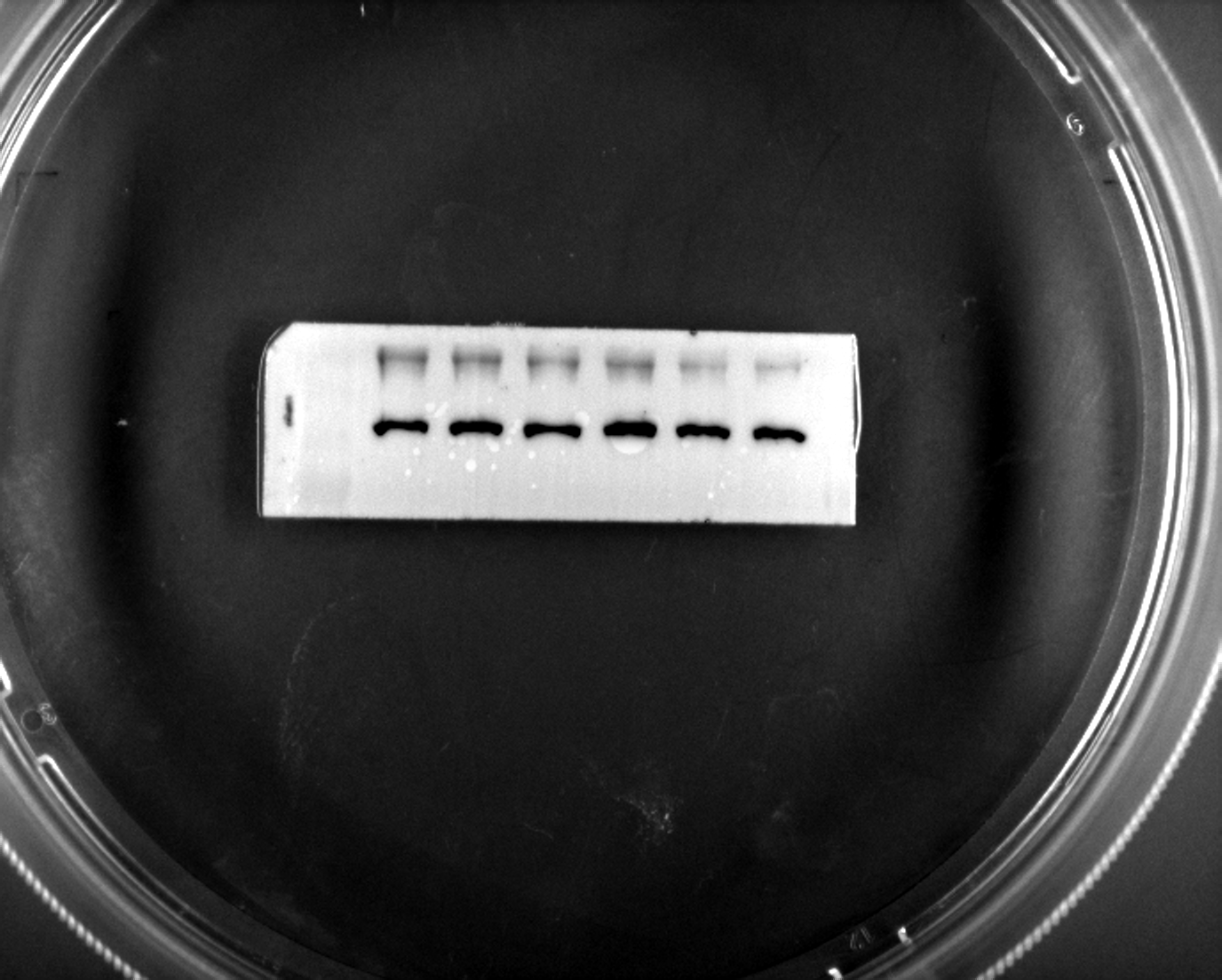

Supplement: Supplemental Information 4 [file peerj-13-19276-s004.zip › western blot-(CP IR group) Cx43membranal/western blot-(CP IR group)Cx43membranal -2/5-ATPase-M.Tif]

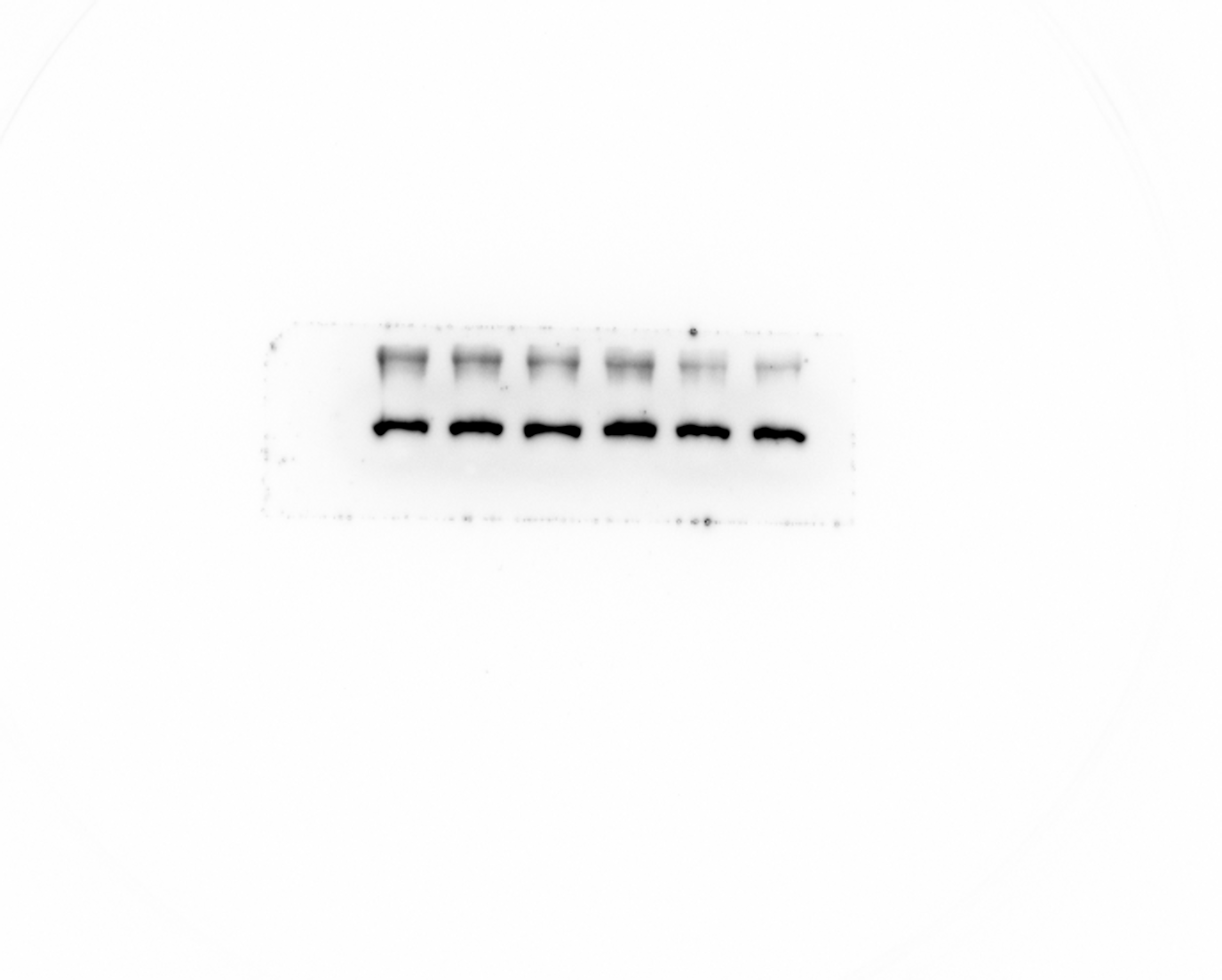

Supplement: Supplemental Information 4 [file peerj-13-19276-s004.zip › western blot-(CP IR group) Cx43membranal/western blot-(CP IR group)Cx43membranal -2/5-ATPase.Tif]

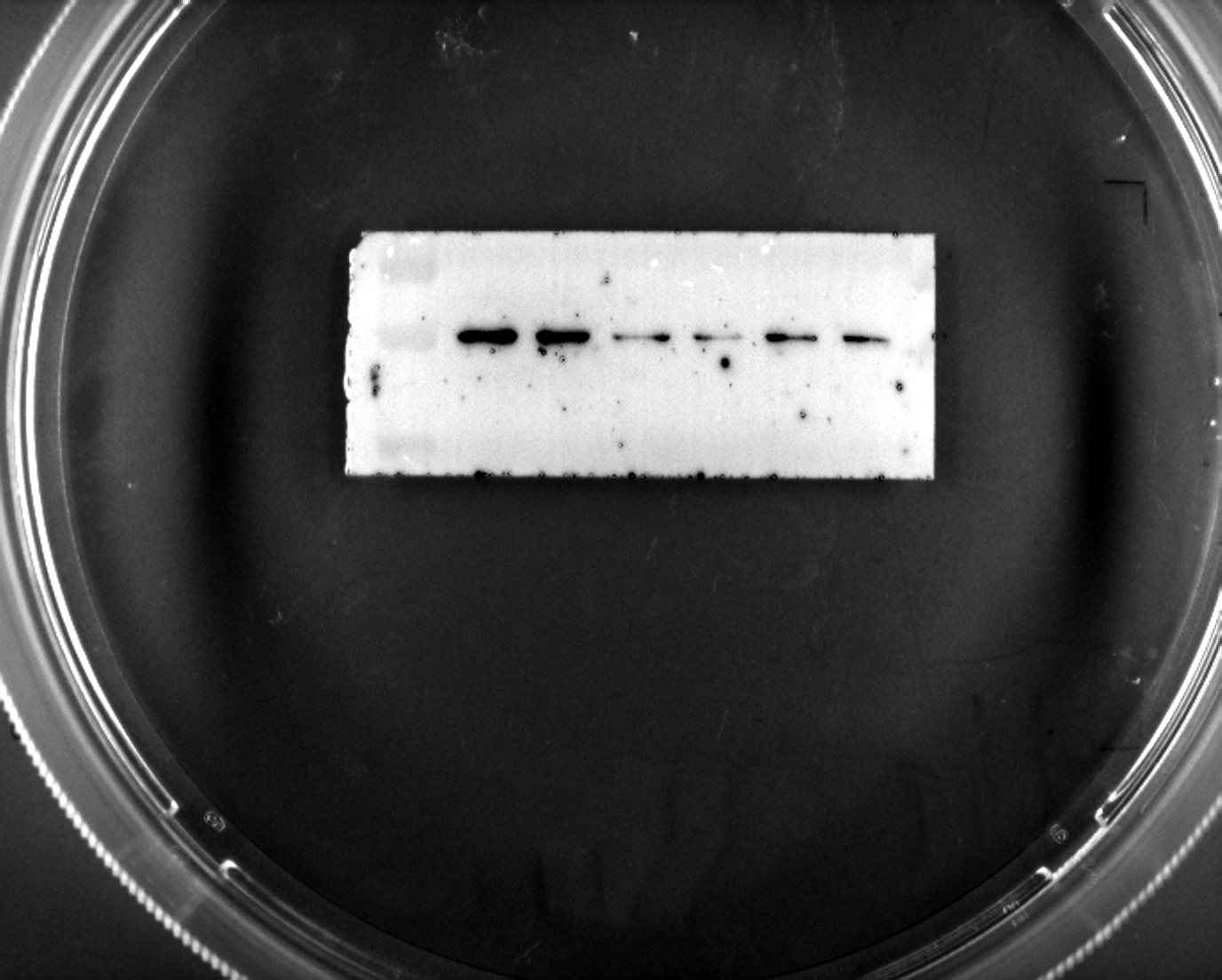

Supplement: Supplemental Information 4 [file peerj-13-19276-s004.zip › western blot-(CP IR group) Cx43membranal/western blot-(CP IR group)Cx43membranal -2/5-CX43-M.Tif]

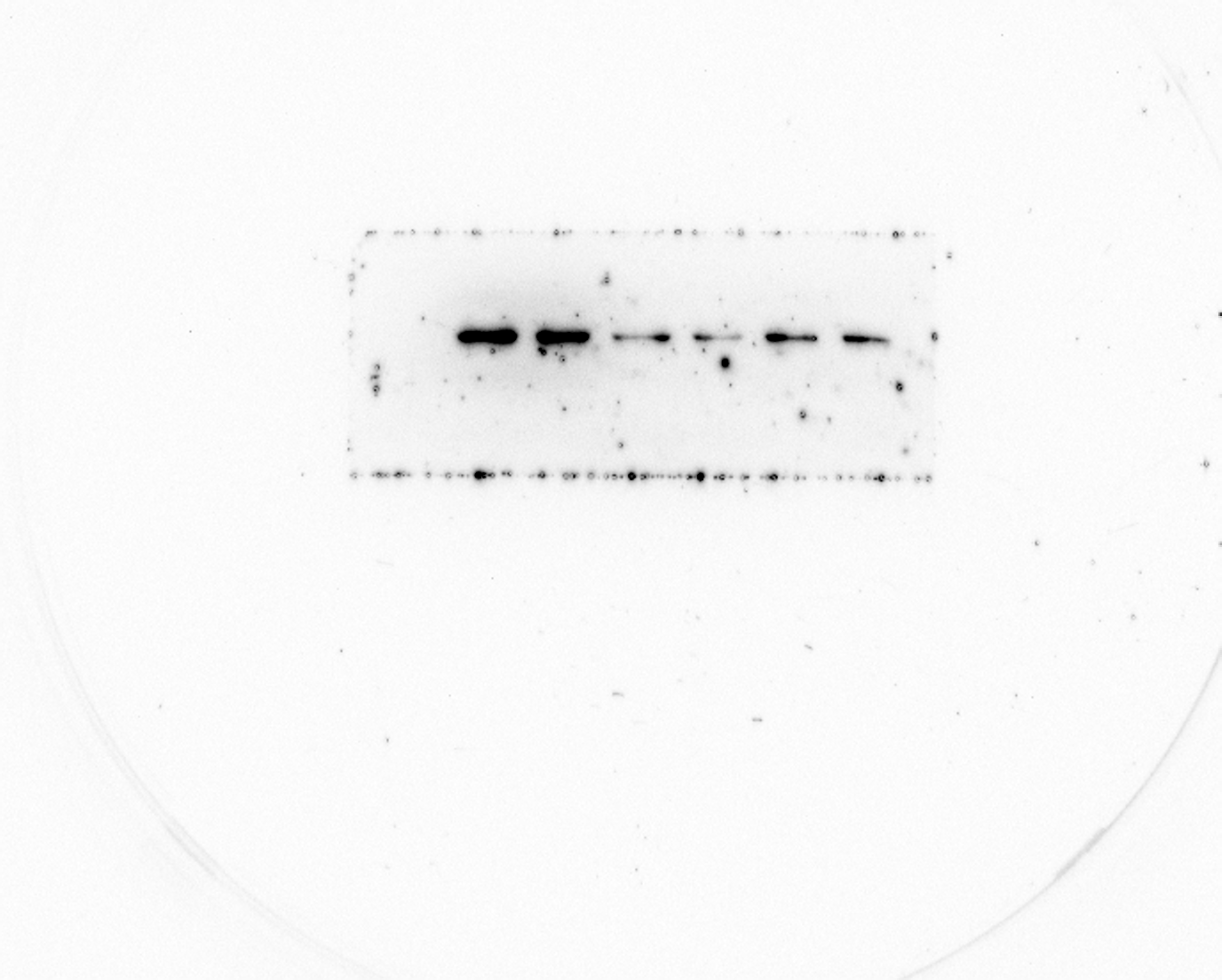

Supplement: Supplemental Information 4 [file peerj-13-19276-s004.zip › western blot-(CP IR group) Cx43membranal/western blot-(CP IR group)Cx43membranal -2/5-CX43.Tif]

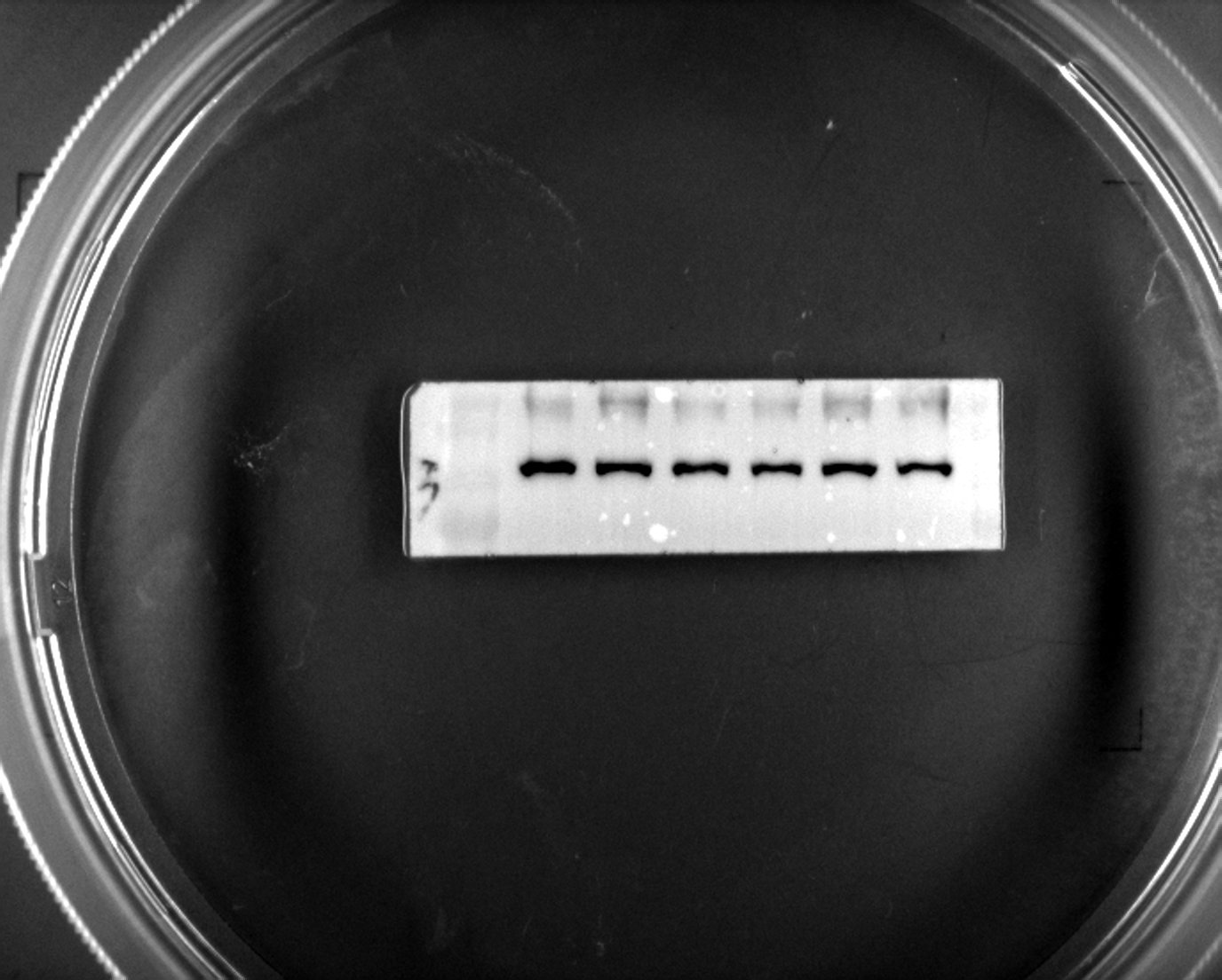

Supplement: Supplemental Information 4 [file peerj-13-19276-s004.zip › western blot-(CP IR group) Cx43membranal/western blot-(CP IR group)Cx43membranal -2/6-ATPase-M.Tif]

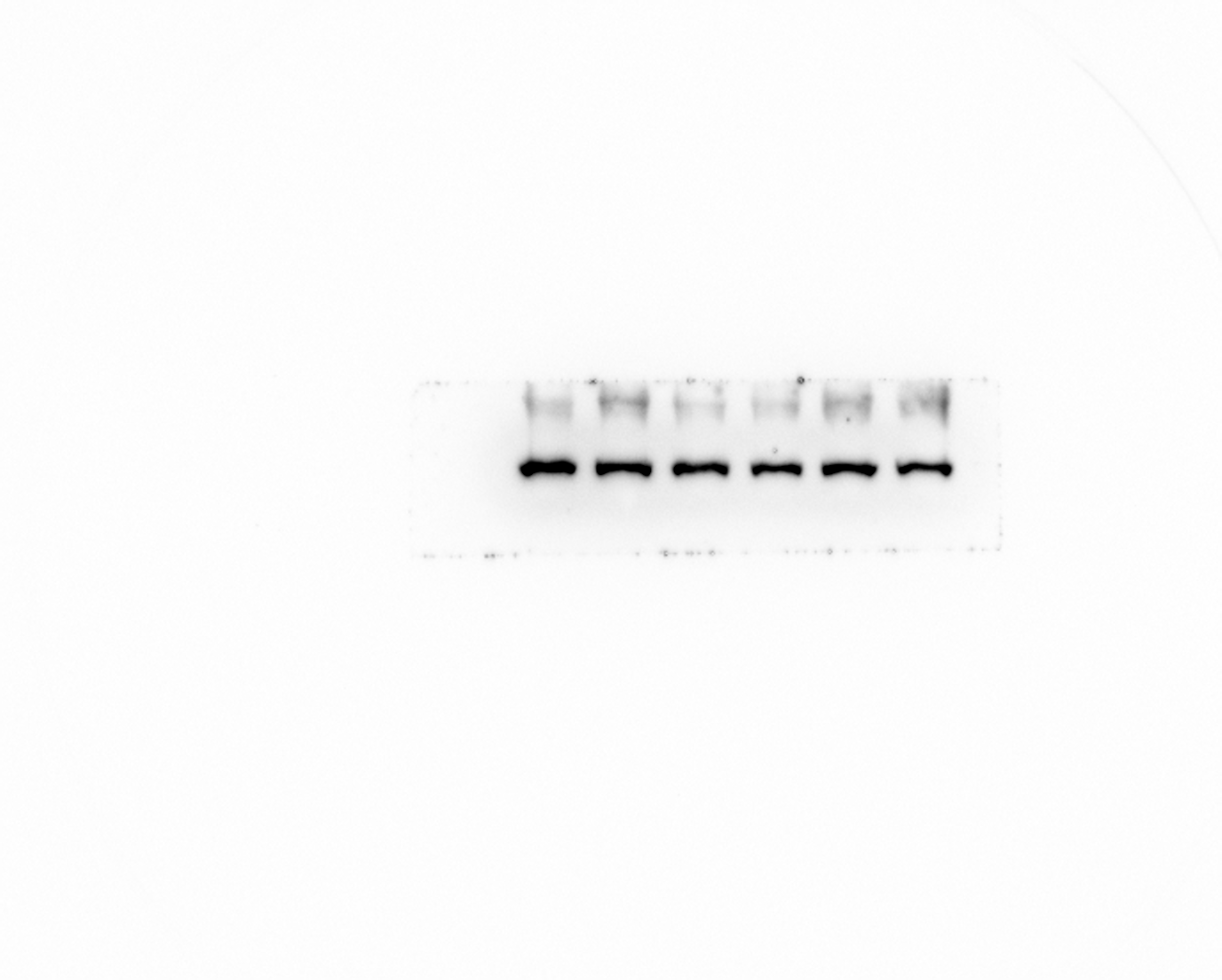

Supplement: Supplemental Information 4 [file peerj-13-19276-s004.zip › western blot-(CP IR group) Cx43membranal/western blot-(CP IR group)Cx43membranal -2/6-ATPase.Tif]

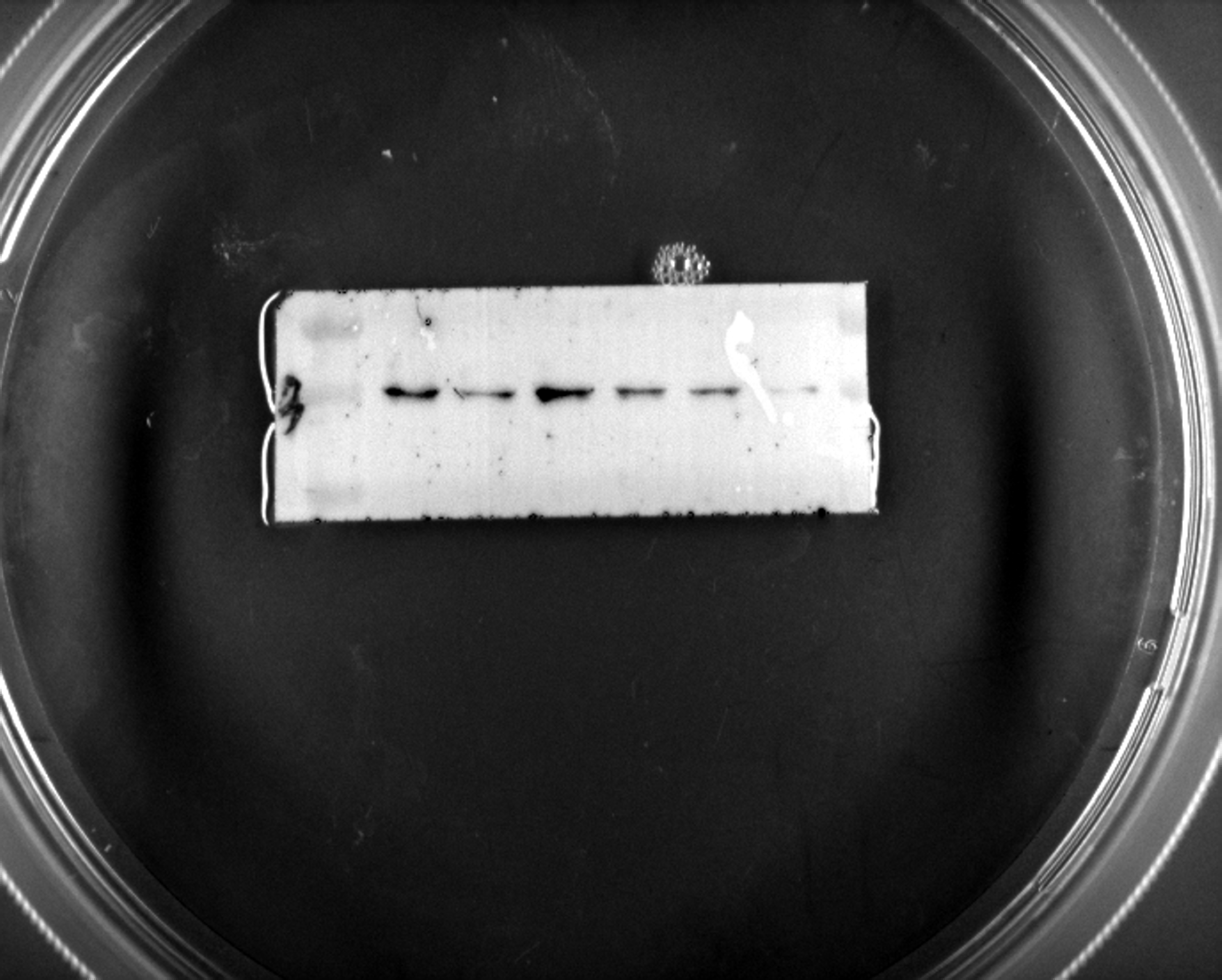

Supplement: Supplemental Information 4 [file peerj-13-19276-s004.zip › western blot-(CP IR group) Cx43membranal/western blot-(CP IR group)Cx43membranal -2/6-CX43-M.Tif]

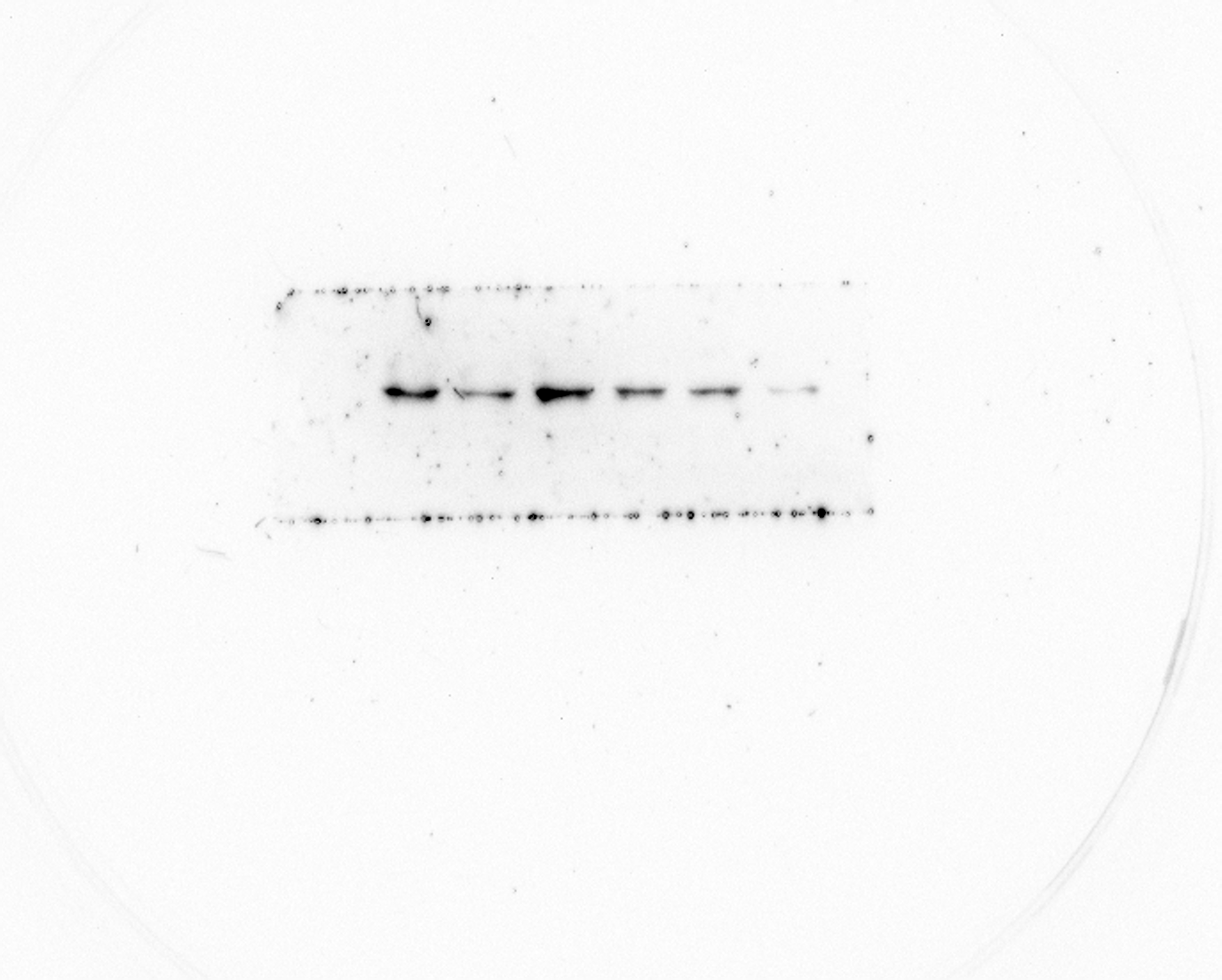

Supplement: Supplemental Information 4 [file peerj-13-19276-s004.zip › western blot-(CP IR group) Cx43membranal/western blot-(CP IR group)Cx43membranal -2/6-CX43.Tif]

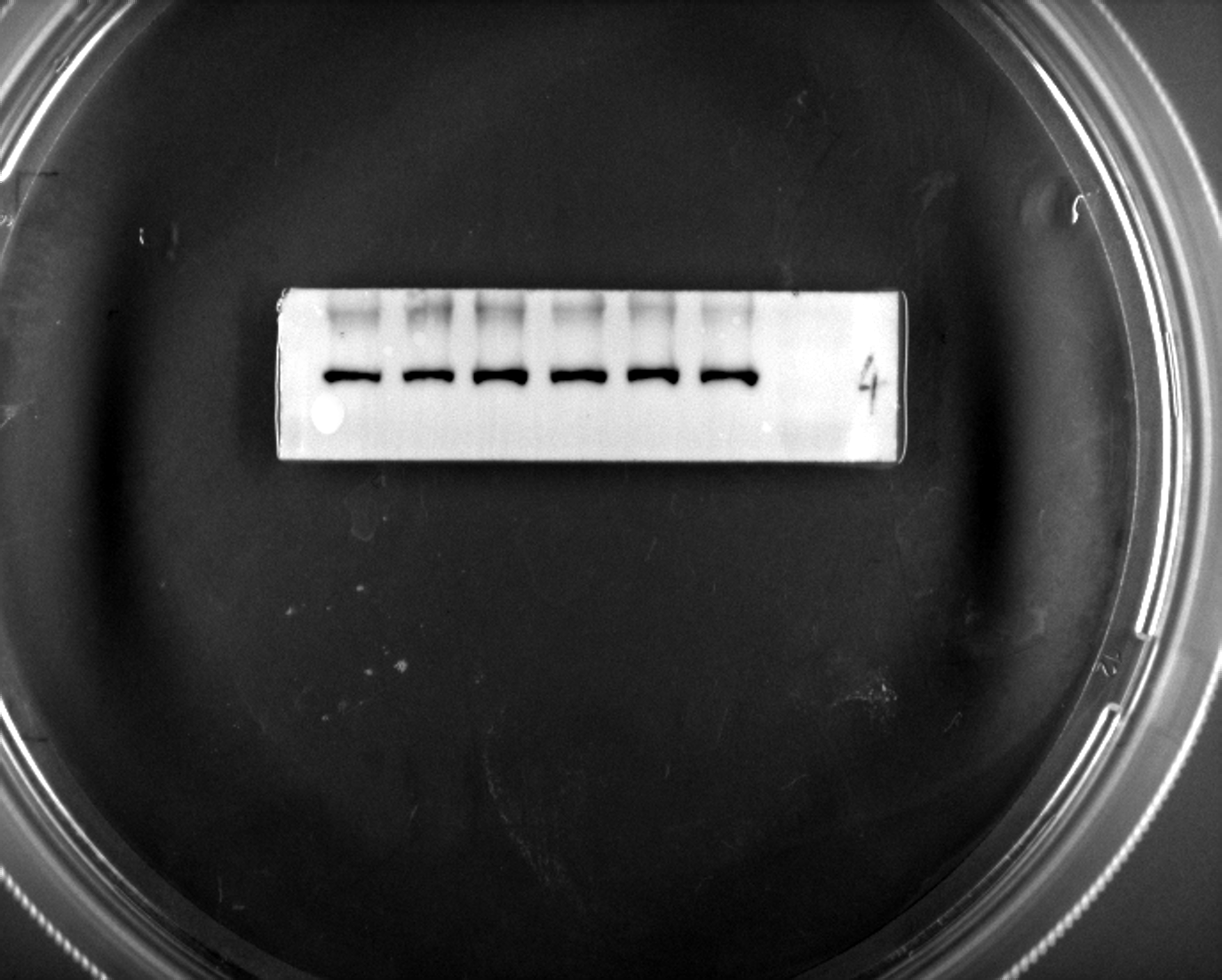

Supplement: Supplemental Information 4 [file peerj-13-19276-s004.zip › western blot-(CP IR group) Cx43membranal/western blot-(CP IR group)Cx43membranal -2/7-ATPase-M.Tif]

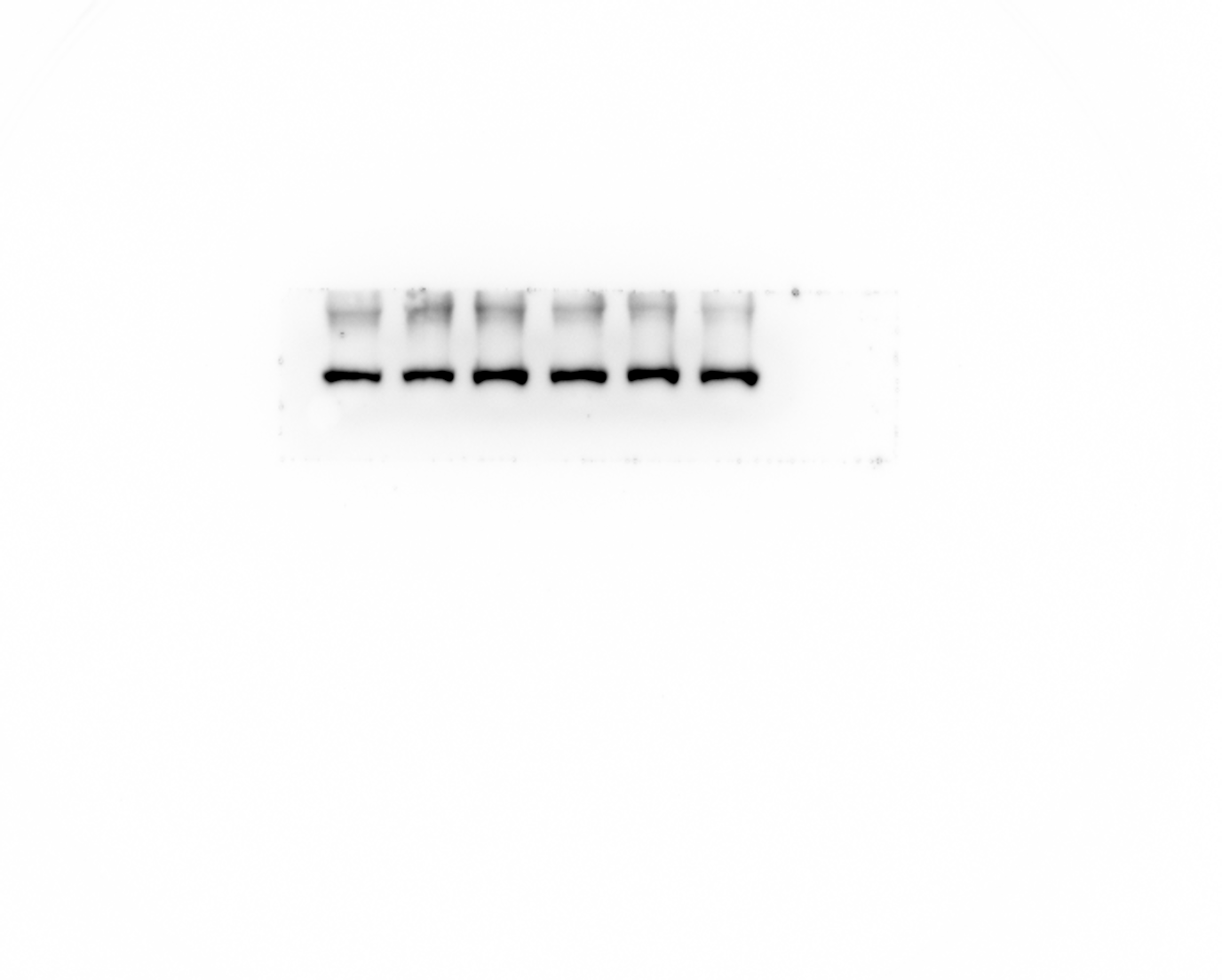

Supplement: Supplemental Information 4 [file peerj-13-19276-s004.zip › western blot-(CP IR group) Cx43membranal/western blot-(CP IR group)Cx43membranal -2/7-ATPase.Tif]

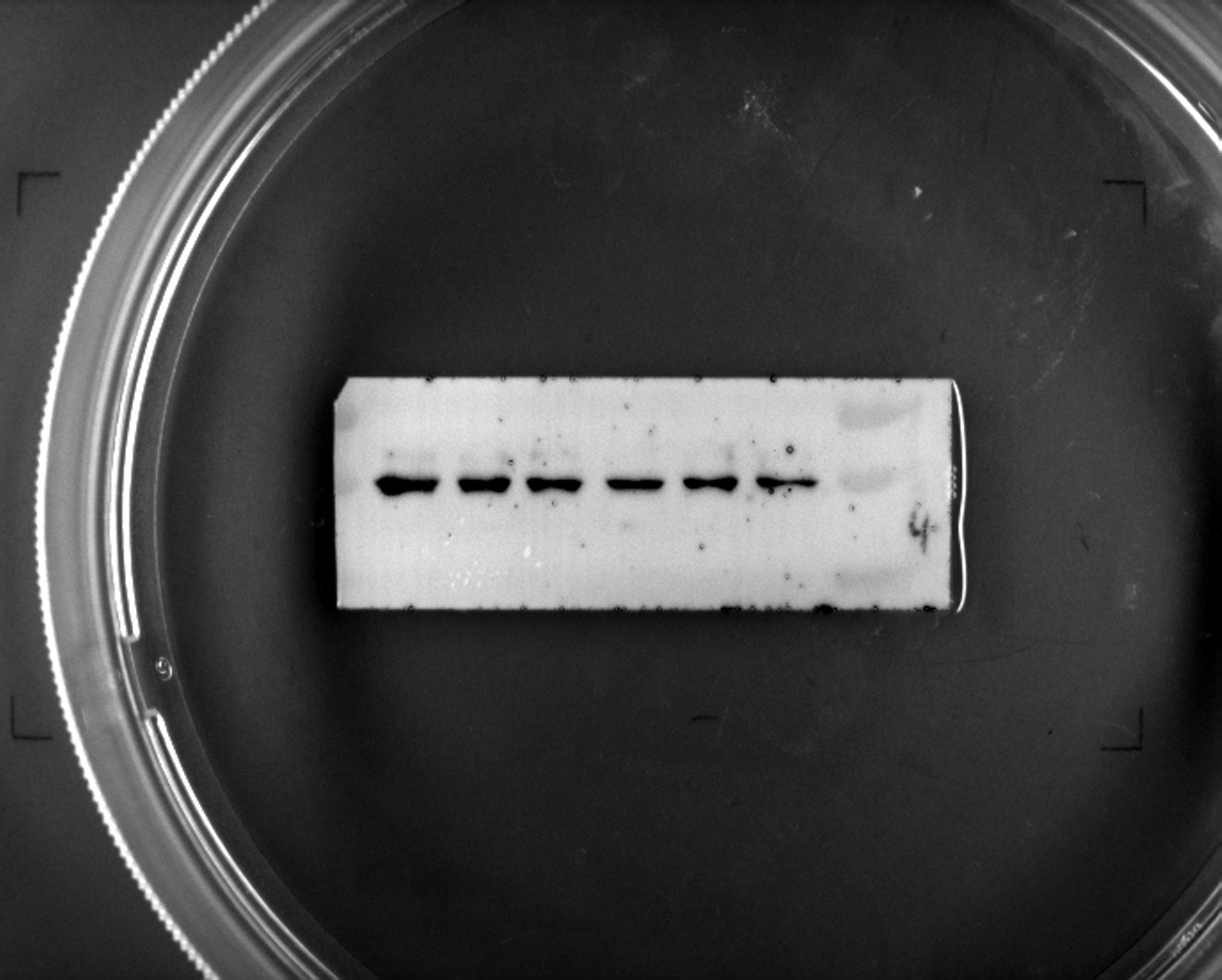

Supplement: Supplemental Information 4 [file peerj-13-19276-s004.zip › western blot-(CP IR group) Cx43membranal/western blot-(CP IR group)Cx43membranal -2/7-CX43-M.Tif]

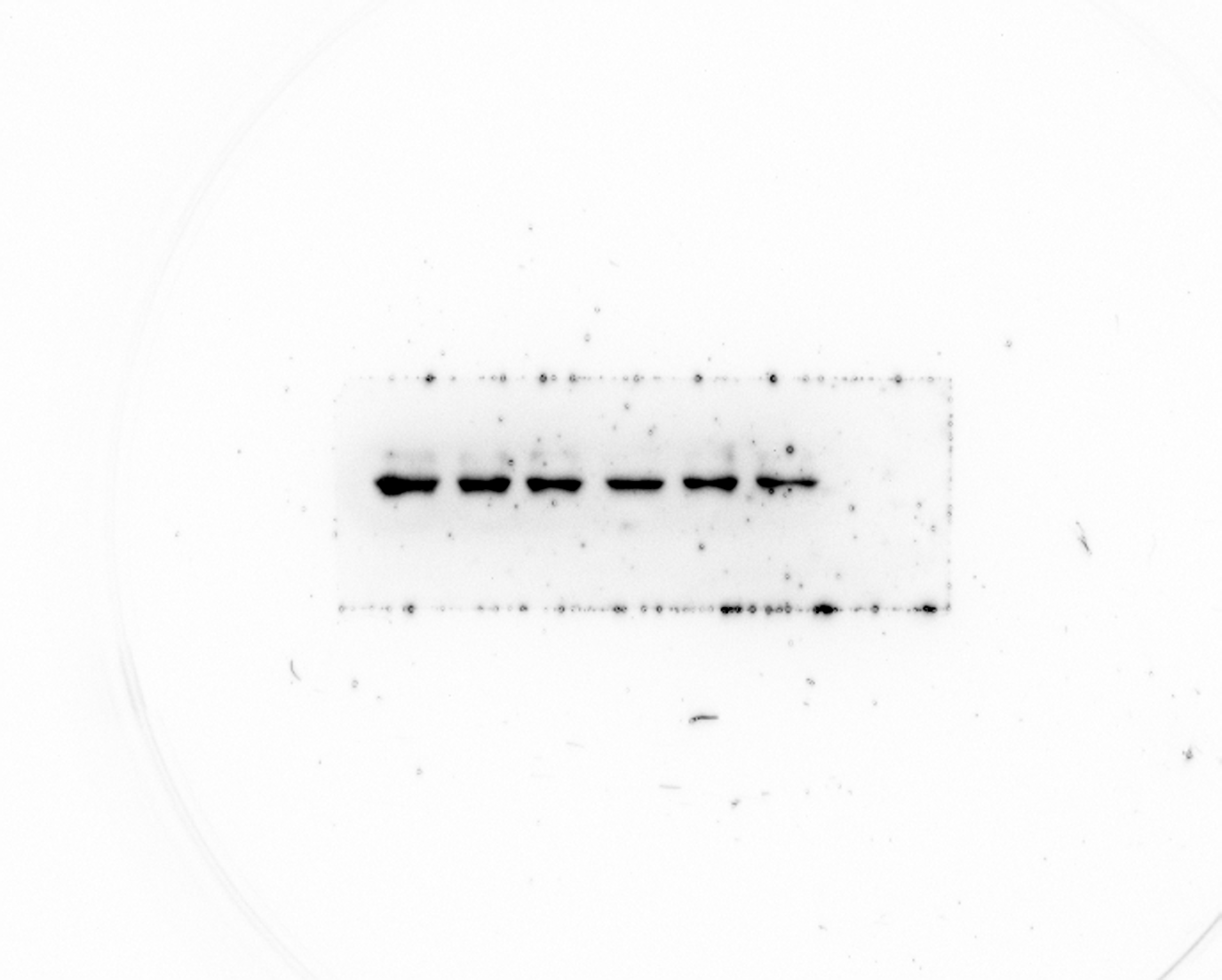

Supplement: Supplemental Information 4 [file peerj-13-19276-s004.zip › western blot-(CP IR group) Cx43membranal/western blot-(CP IR group)Cx43membranal -2/7-CX43.Tif]

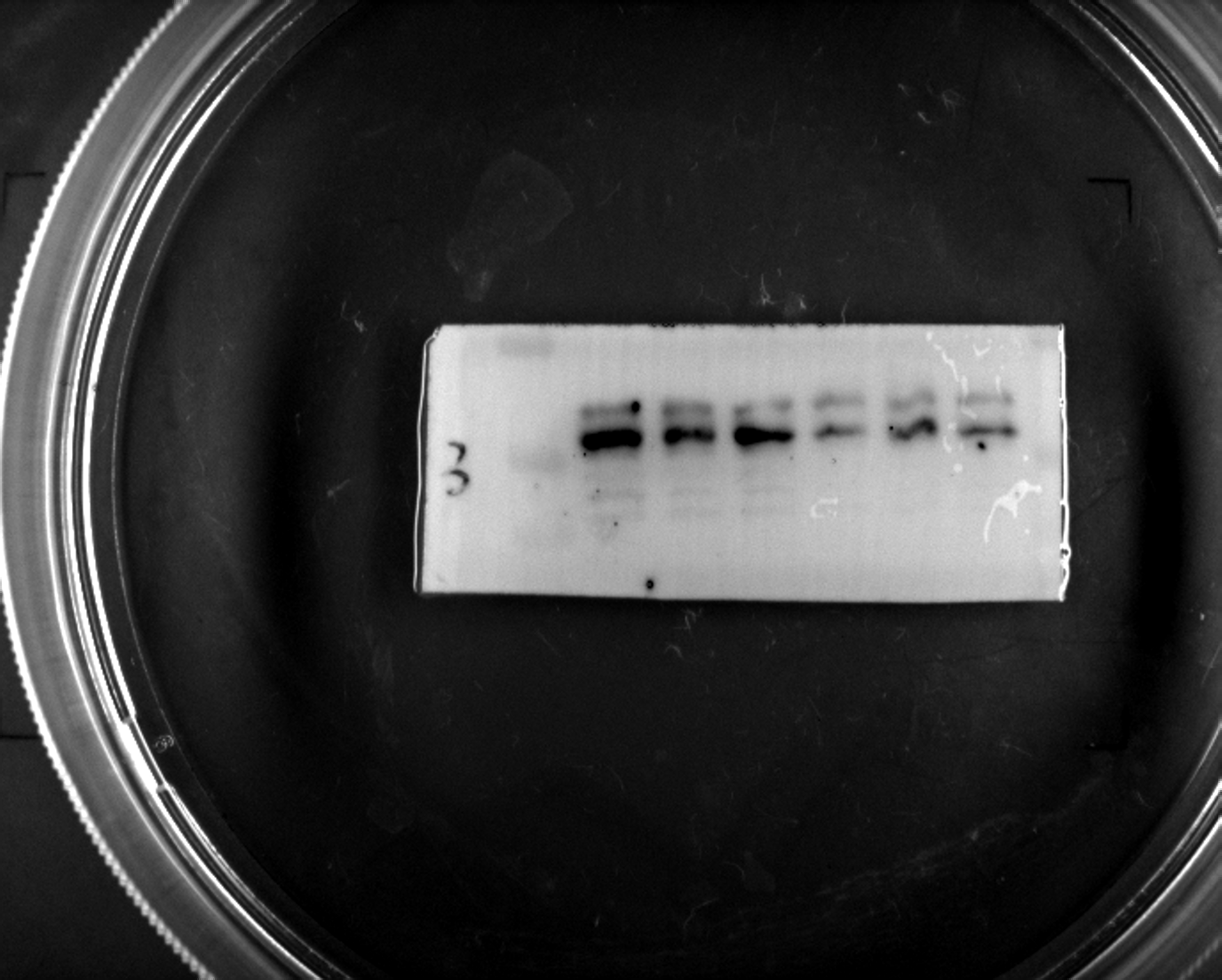

Supplement: Supplemental Information 5 [file peerj-13-19276-s005.zip › western blot-(CP IR group) EB1/western blot-(CP IR group) EB1-1/5-EB1-M.Tif]

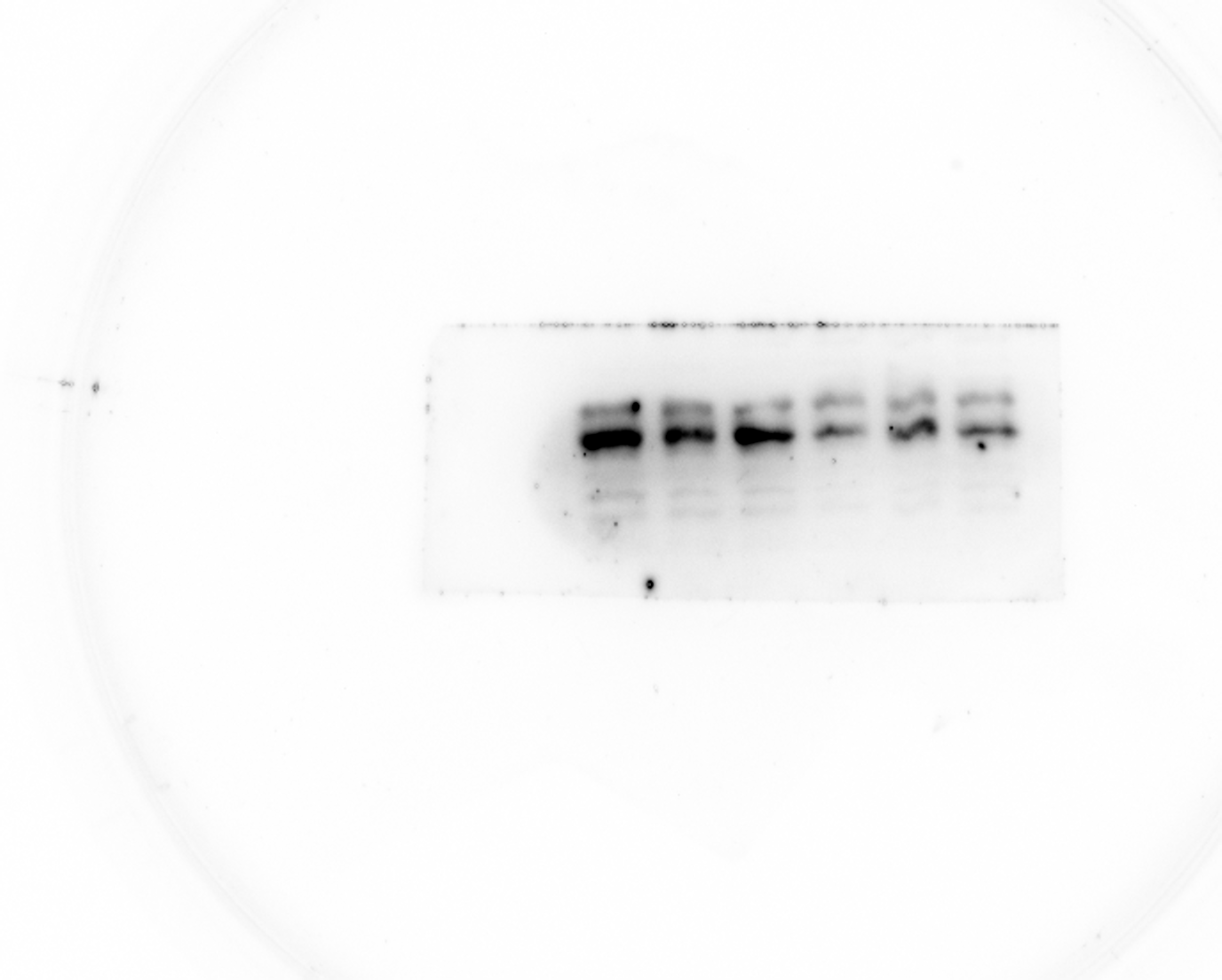

Supplement: Supplemental Information 5 [file peerj-13-19276-s005.zip › western blot-(CP IR group) EB1/western blot-(CP IR group) EB1-1/5-EB1.Tif]

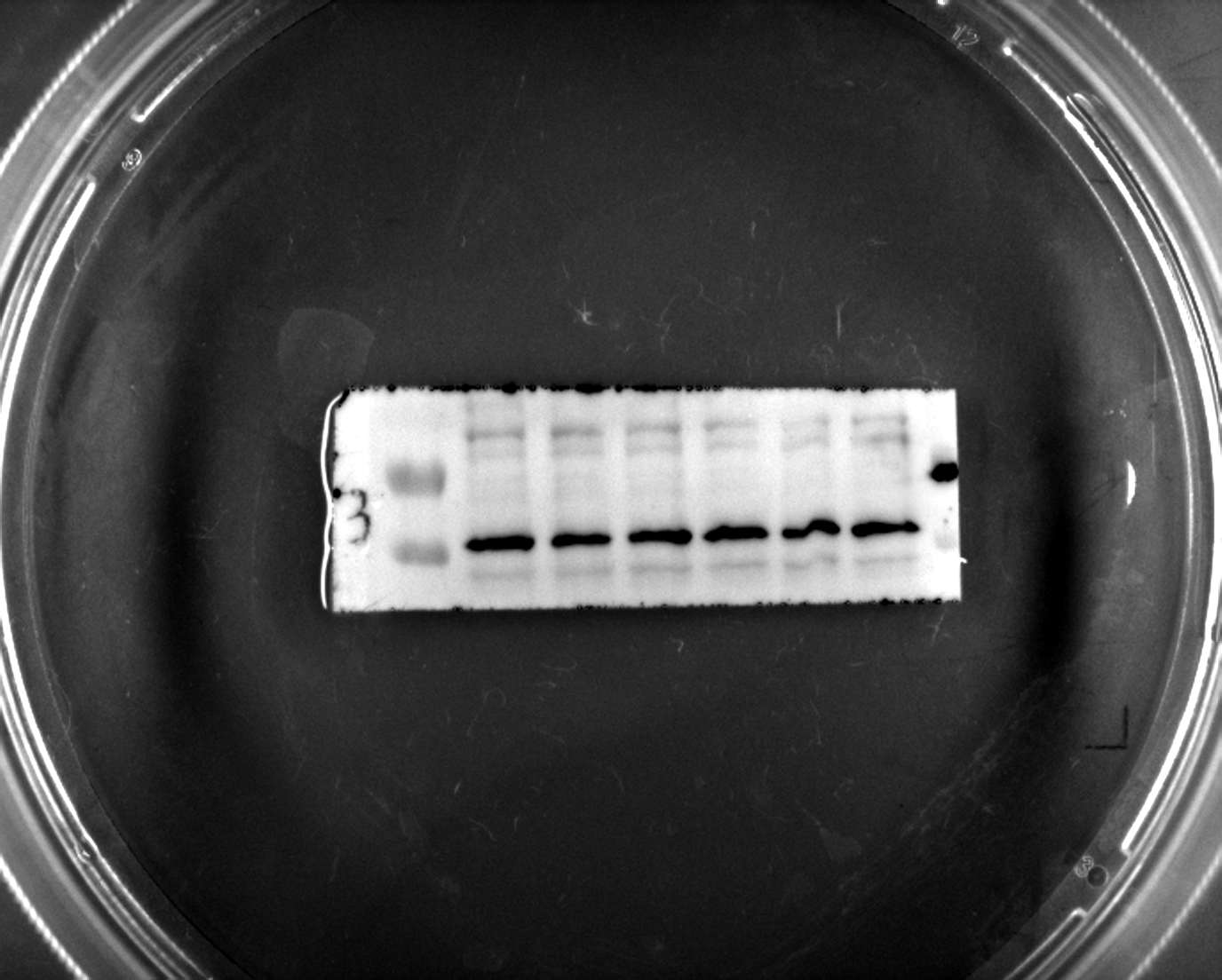

Supplement: Supplemental Information 5 [file peerj-13-19276-s005.zip › western blot-(CP IR group) EB1/western blot-(CP IR group) EB1-1/5-Tubulin-M.Tif]

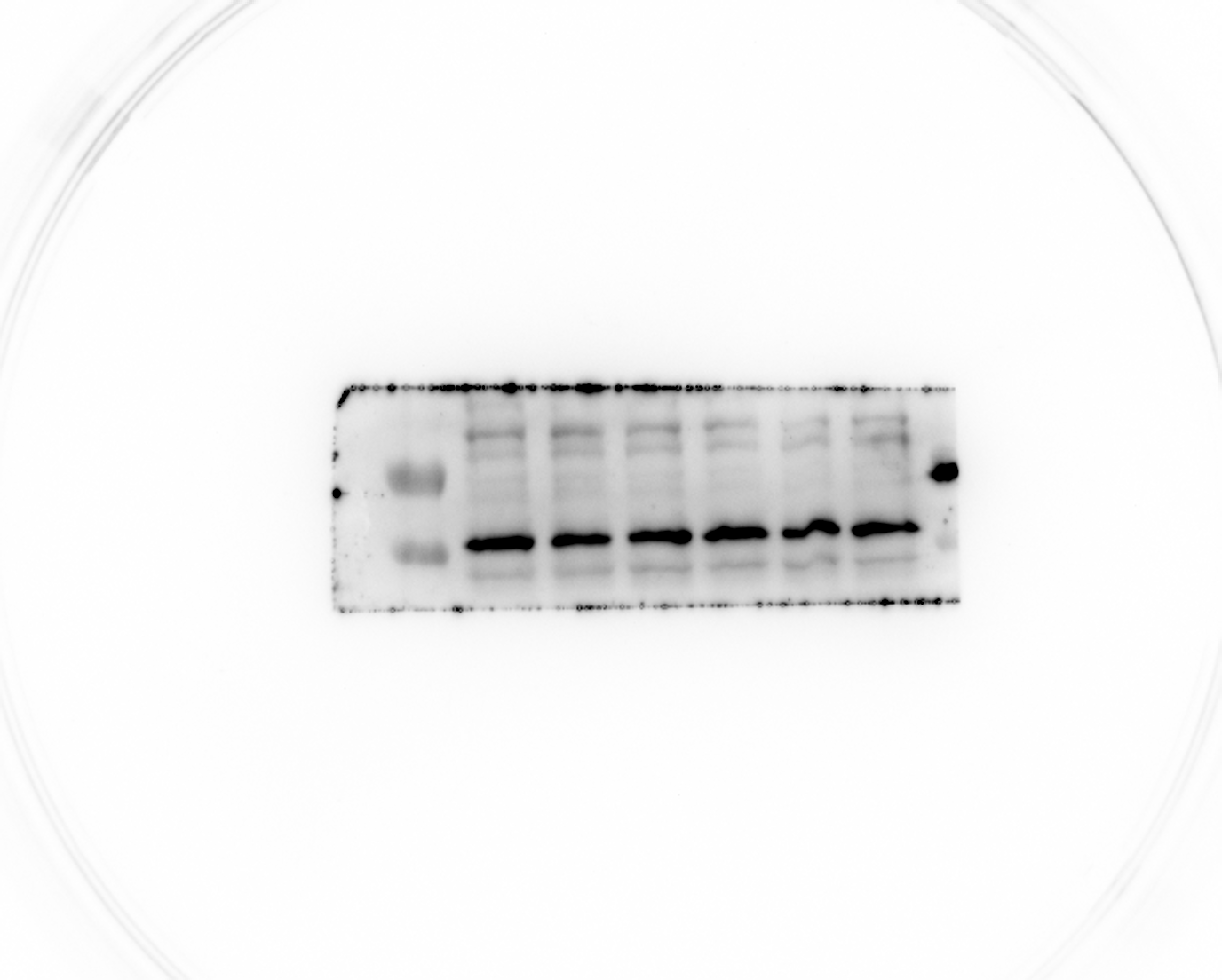

Supplement: Supplemental Information 5 [file peerj-13-19276-s005.zip › western blot-(CP IR group) EB1/western blot-(CP IR group) EB1-1/5-Tubulin.Tif]

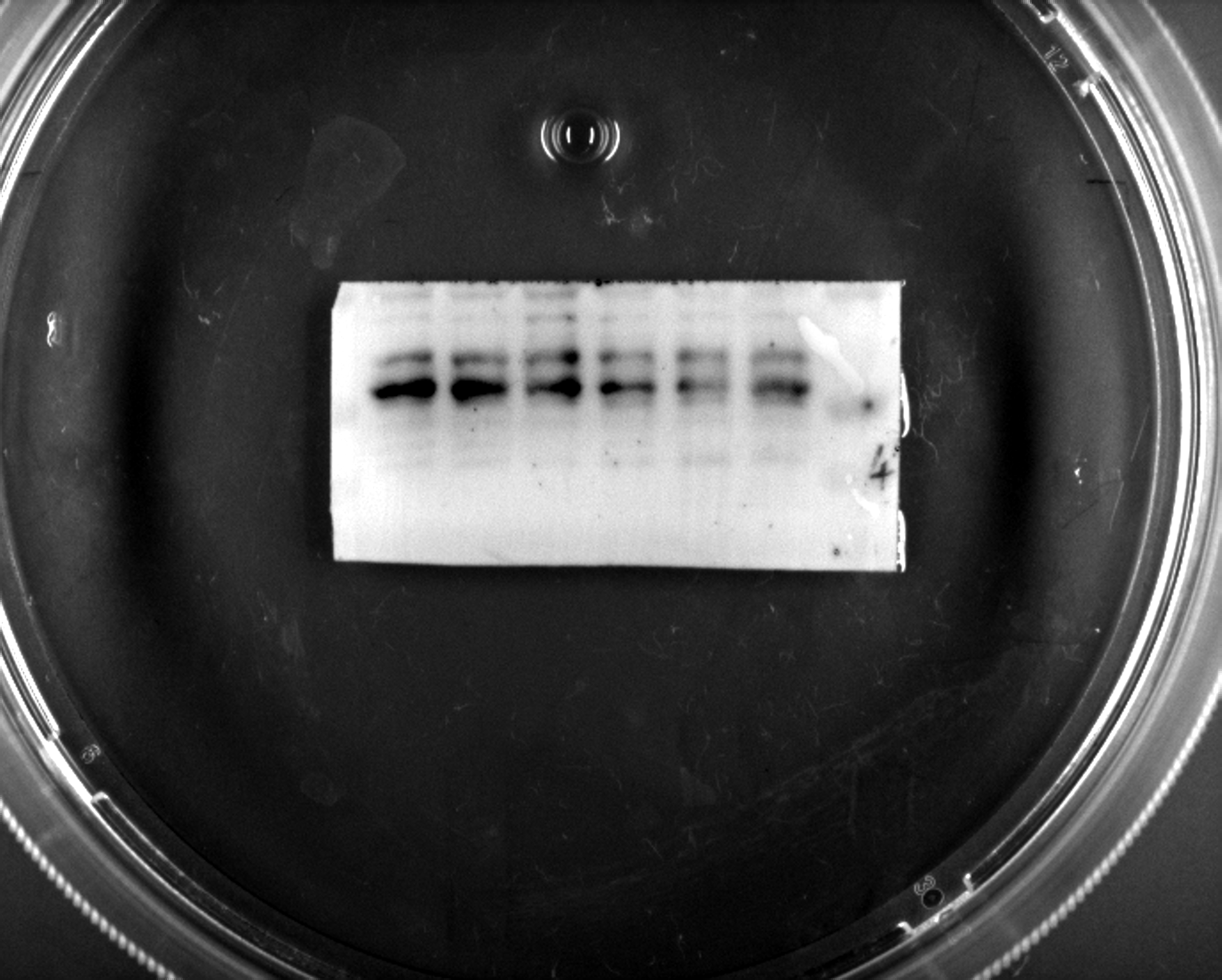

Supplement: Supplemental Information 5 [file peerj-13-19276-s005.zip › western blot-(CP IR group) EB1/western blot-(CP IR group) EB1-1/6-EB1-M.Tif]

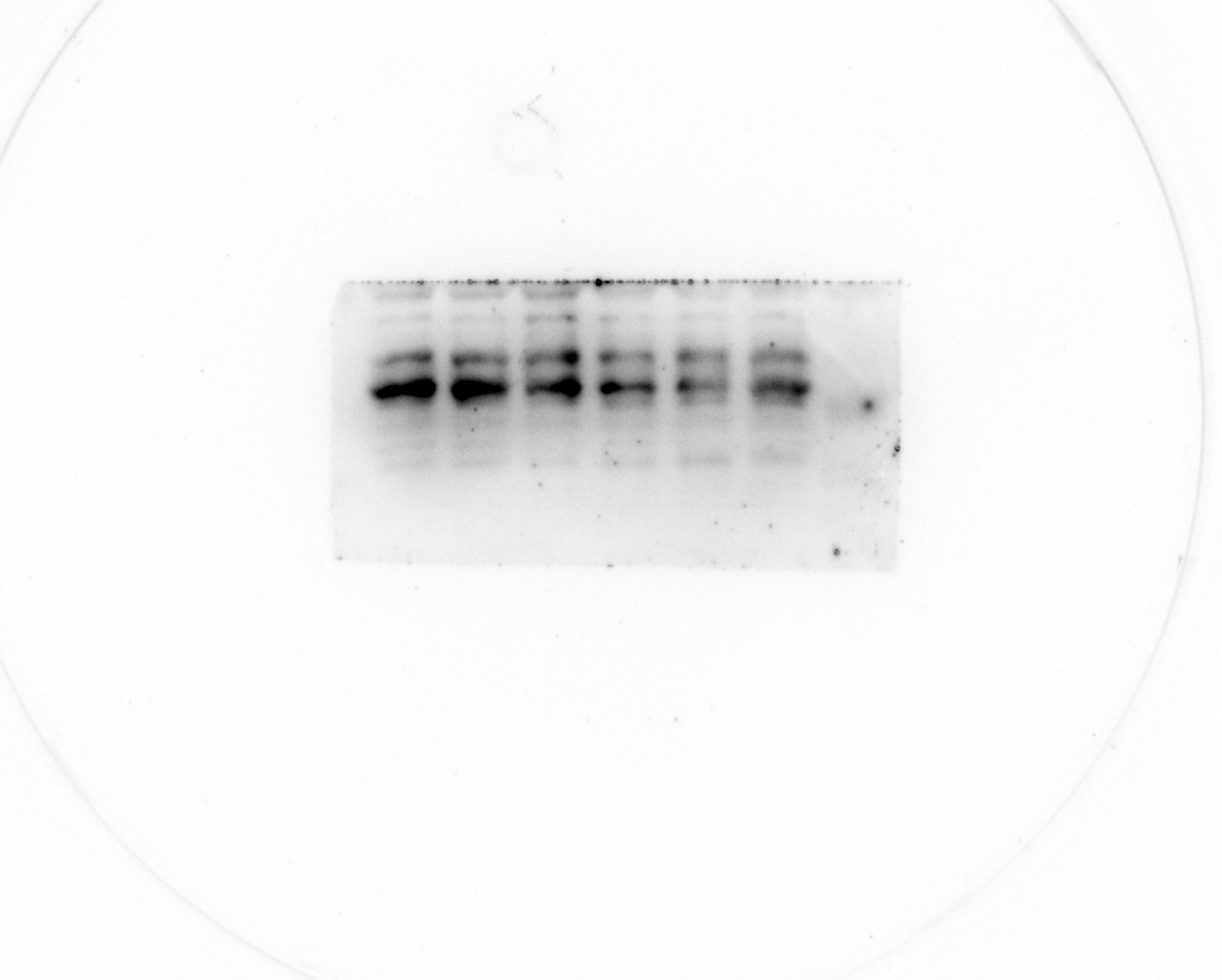

Supplement: Supplemental Information 5 [file peerj-13-19276-s005.zip › western blot-(CP IR group) EB1/western blot-(CP IR group) EB1-1/6-EB1.Tif]

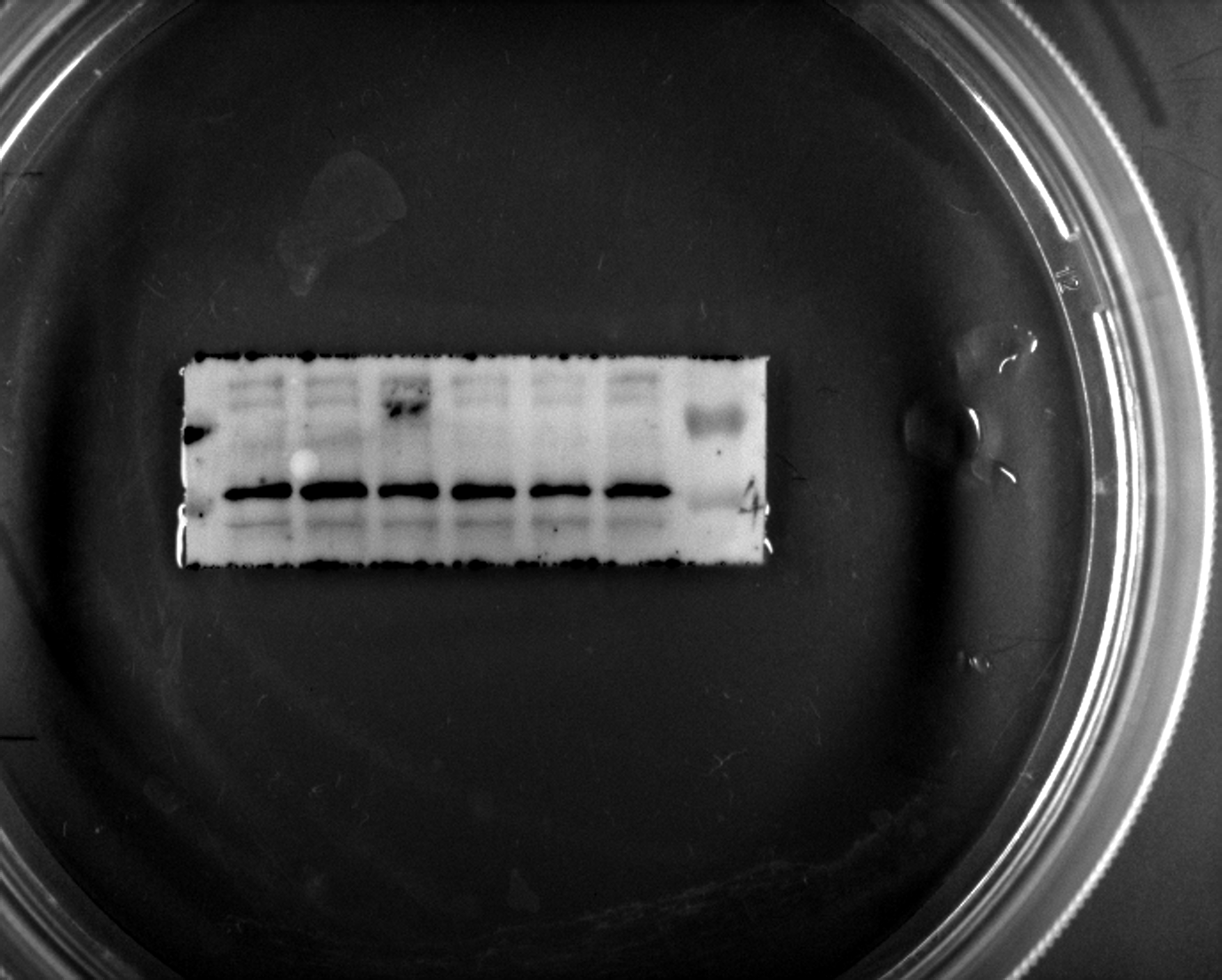

Supplement: Supplemental Information 5 [file peerj-13-19276-s005.zip › western blot-(CP IR group) EB1/western blot-(CP IR group) EB1-1/6-Tubulin-M.Tif]

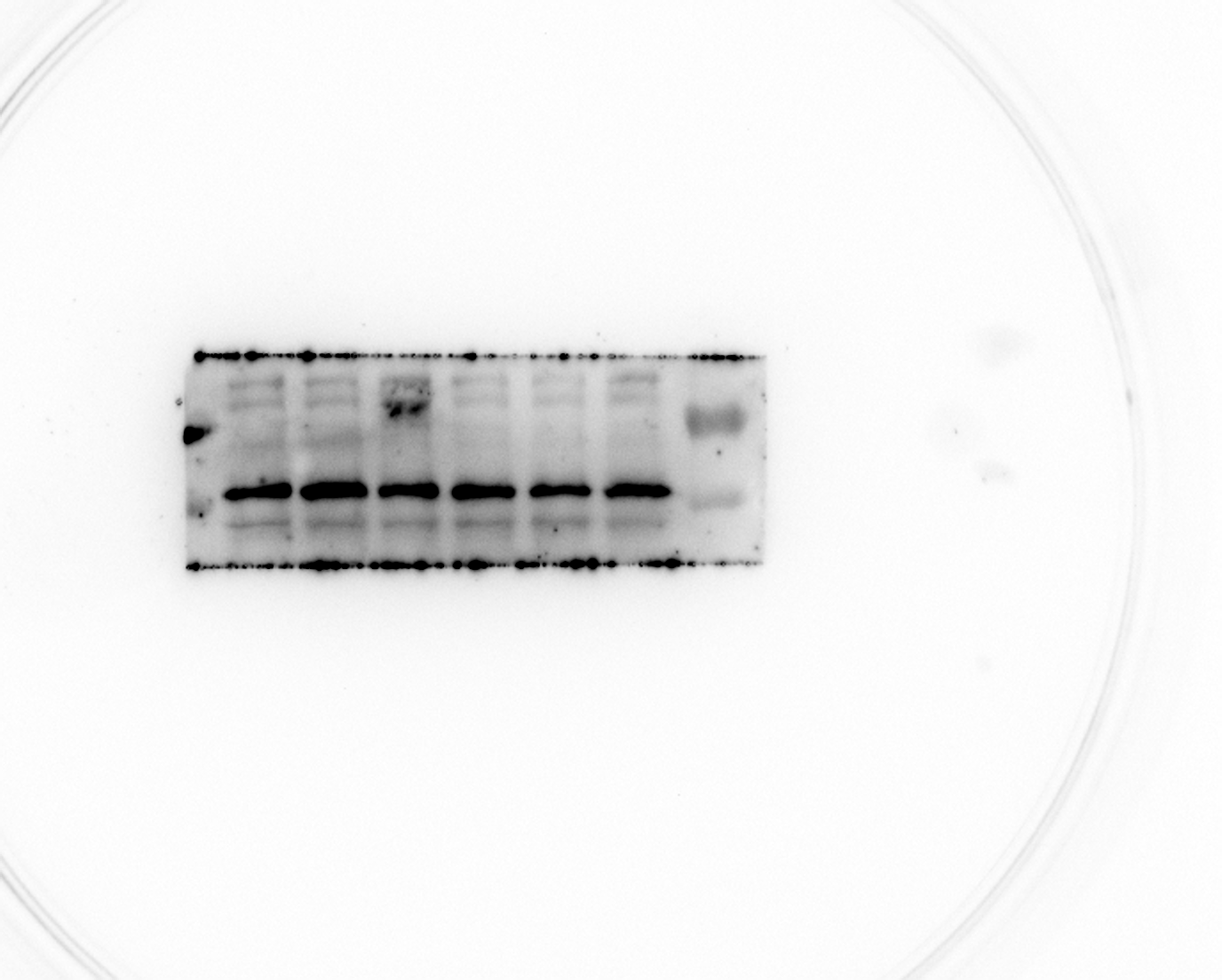

Supplement: Supplemental Information 5 [file peerj-13-19276-s005.zip › western blot-(CP IR group) EB1/western blot-(CP IR group) EB1-1/6-Tubulin.Tif]

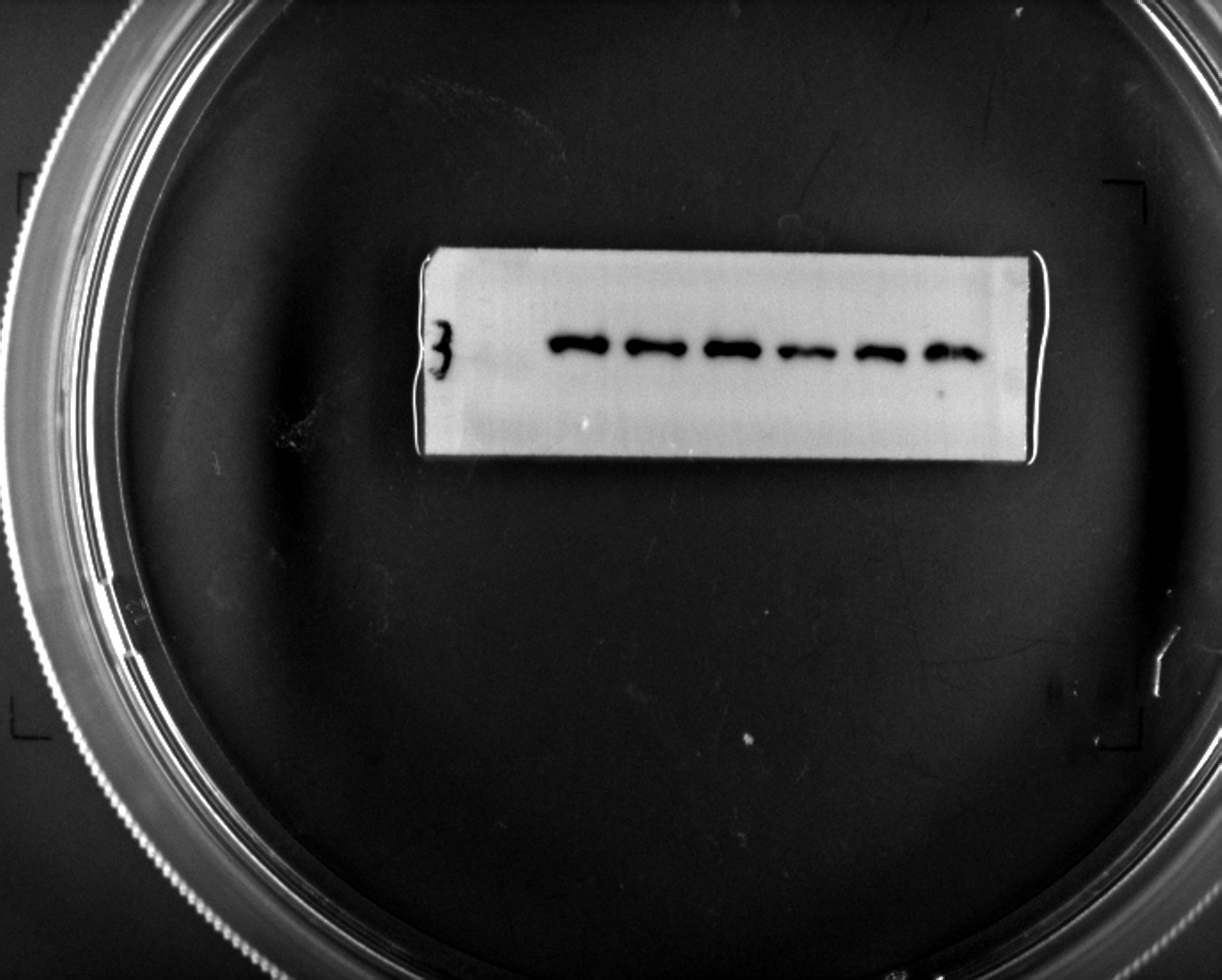

Supplement: Supplemental Information 5 [file peerj-13-19276-s005.zip › western blot-(CP IR group) EB1/western blot-(CP IR group) EB1-1/7-EB1-M.Tif]

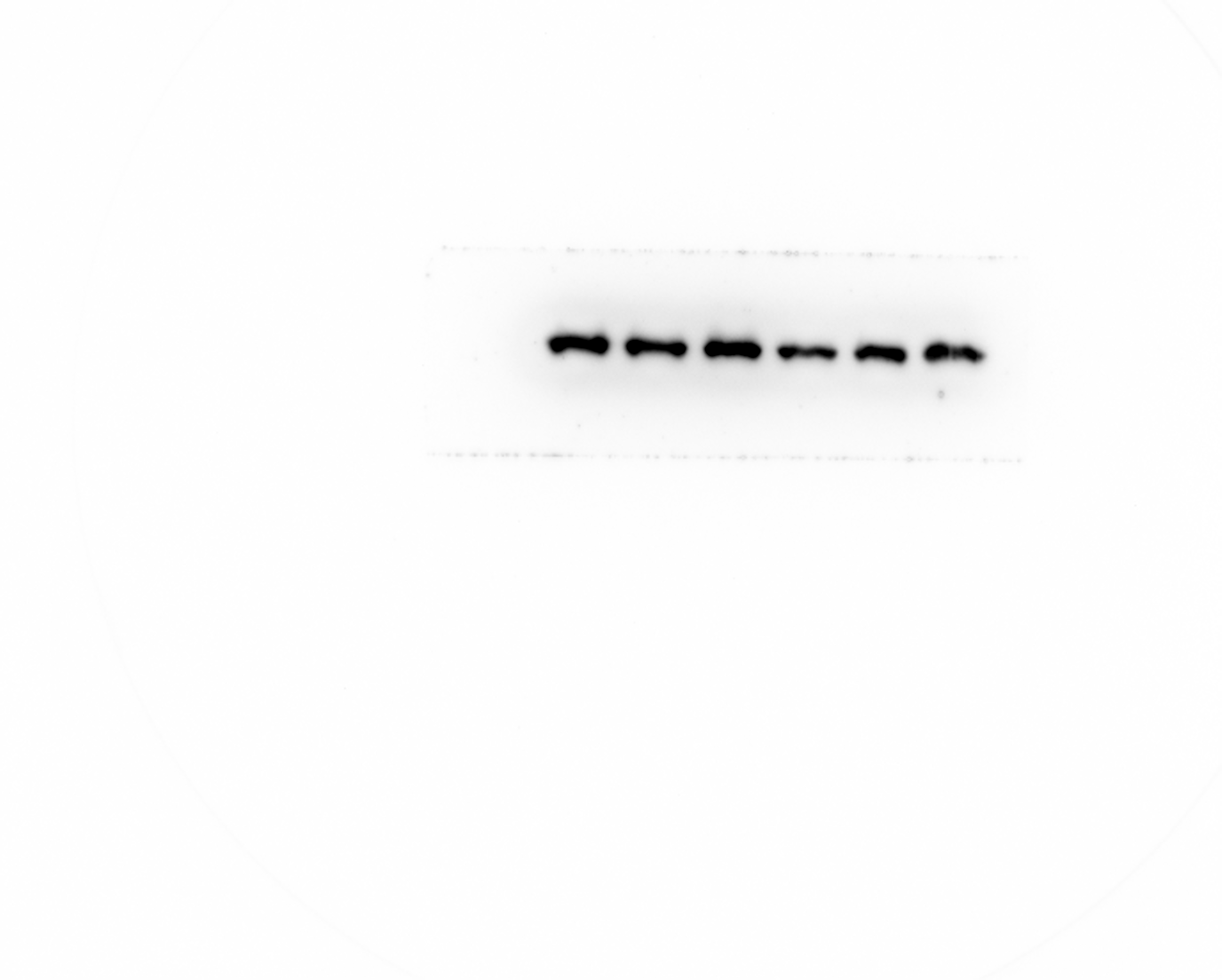

Supplement: Supplemental Information 5 [file peerj-13-19276-s005.zip › western blot-(CP IR group) EB1/western blot-(CP IR group) EB1-1/7-EB1.Tif]

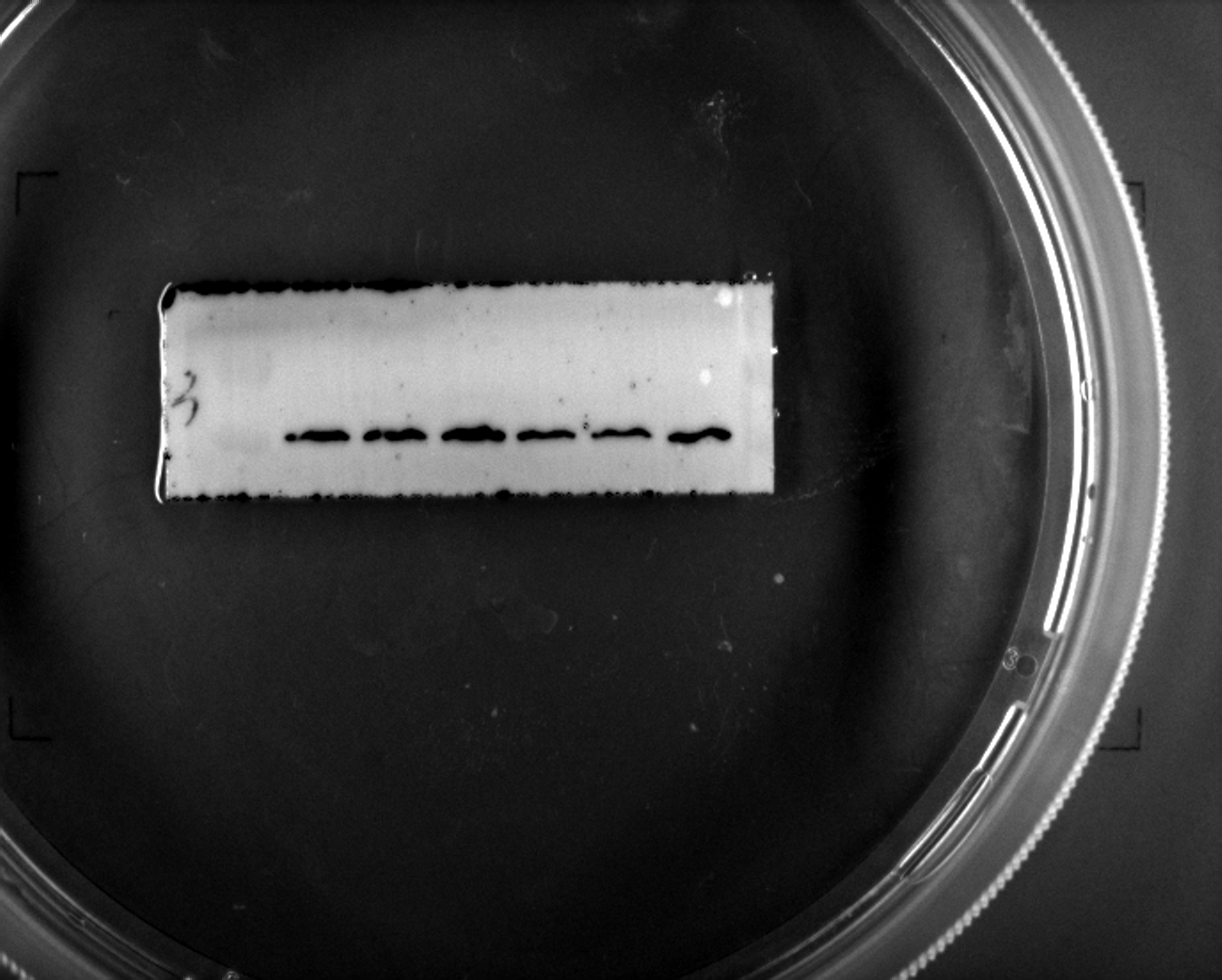

Supplement: Supplemental Information 5 [file peerj-13-19276-s005.zip › western blot-(CP IR group) EB1/western blot-(CP IR group) EB1-1/7-Tubulin-M.Tif]

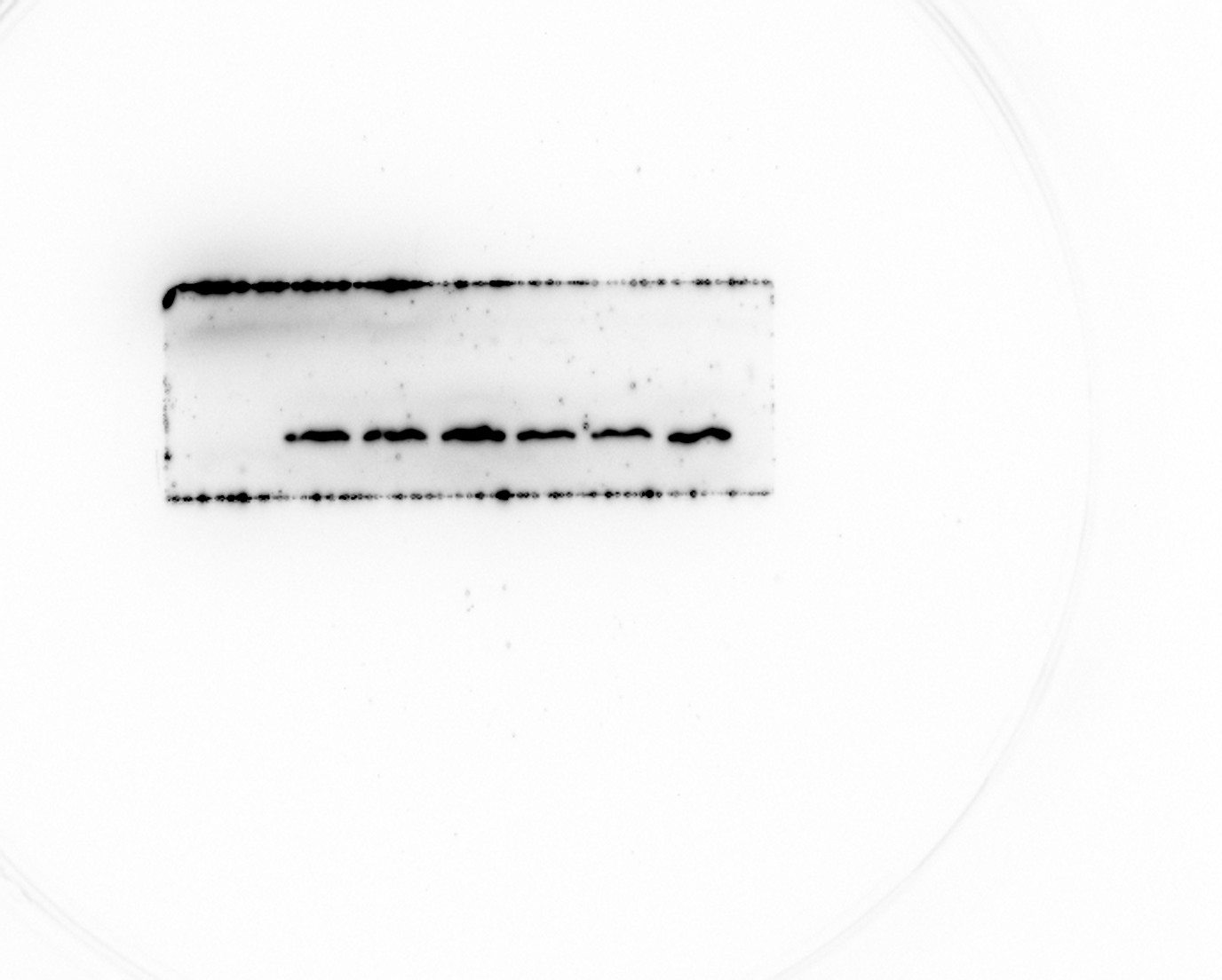

Supplement: Supplemental Information 5 [file peerj-13-19276-s005.zip › western blot-(CP IR group) EB1/western blot-(CP IR group) EB1-1/7-Tubulin.Tif]

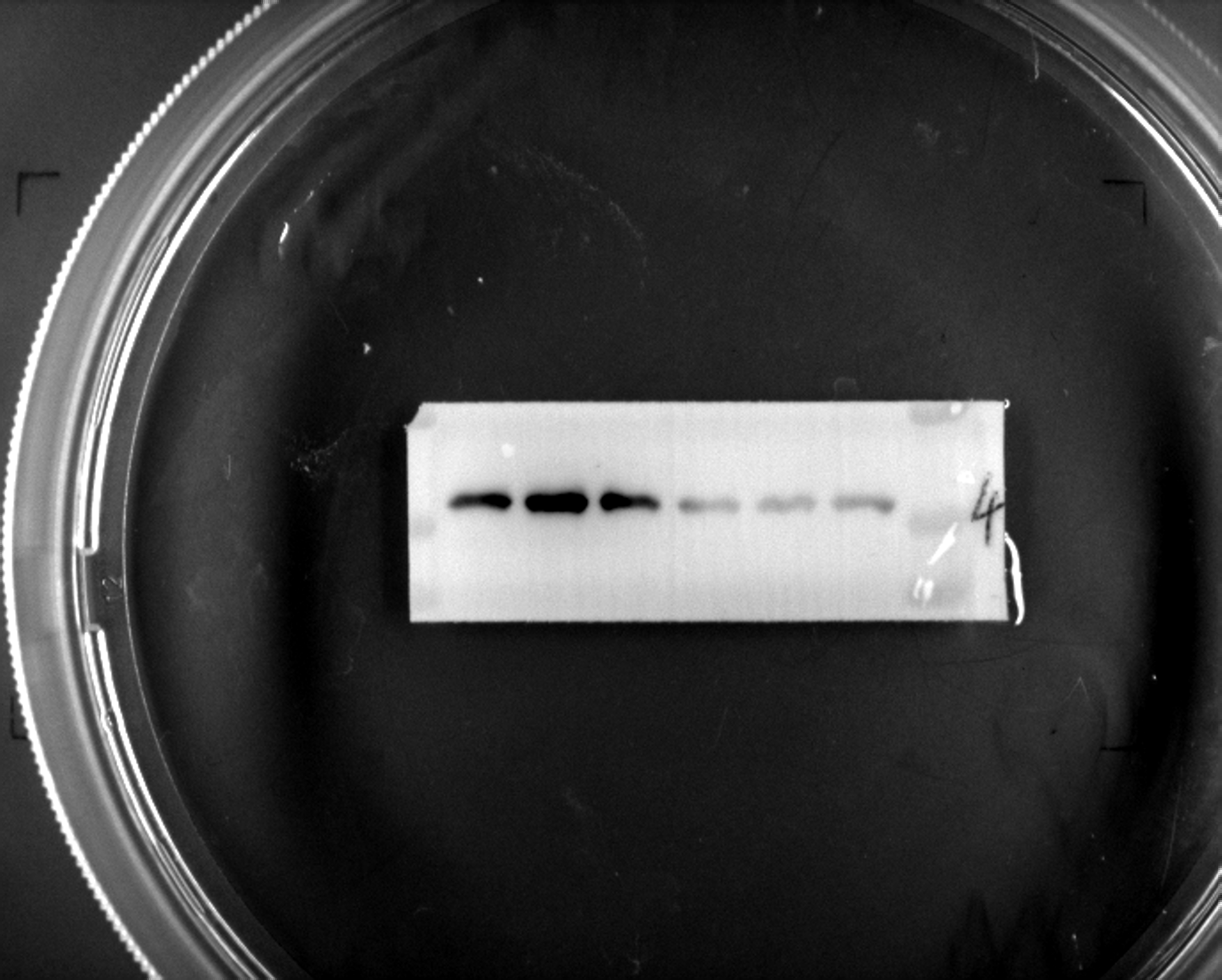

Supplement: Supplemental Information 5 [file peerj-13-19276-s005.zip › western blot-(CP IR group) EB1/western blot-(CP IR group) EB1-1/8-EB1-M.Tif]

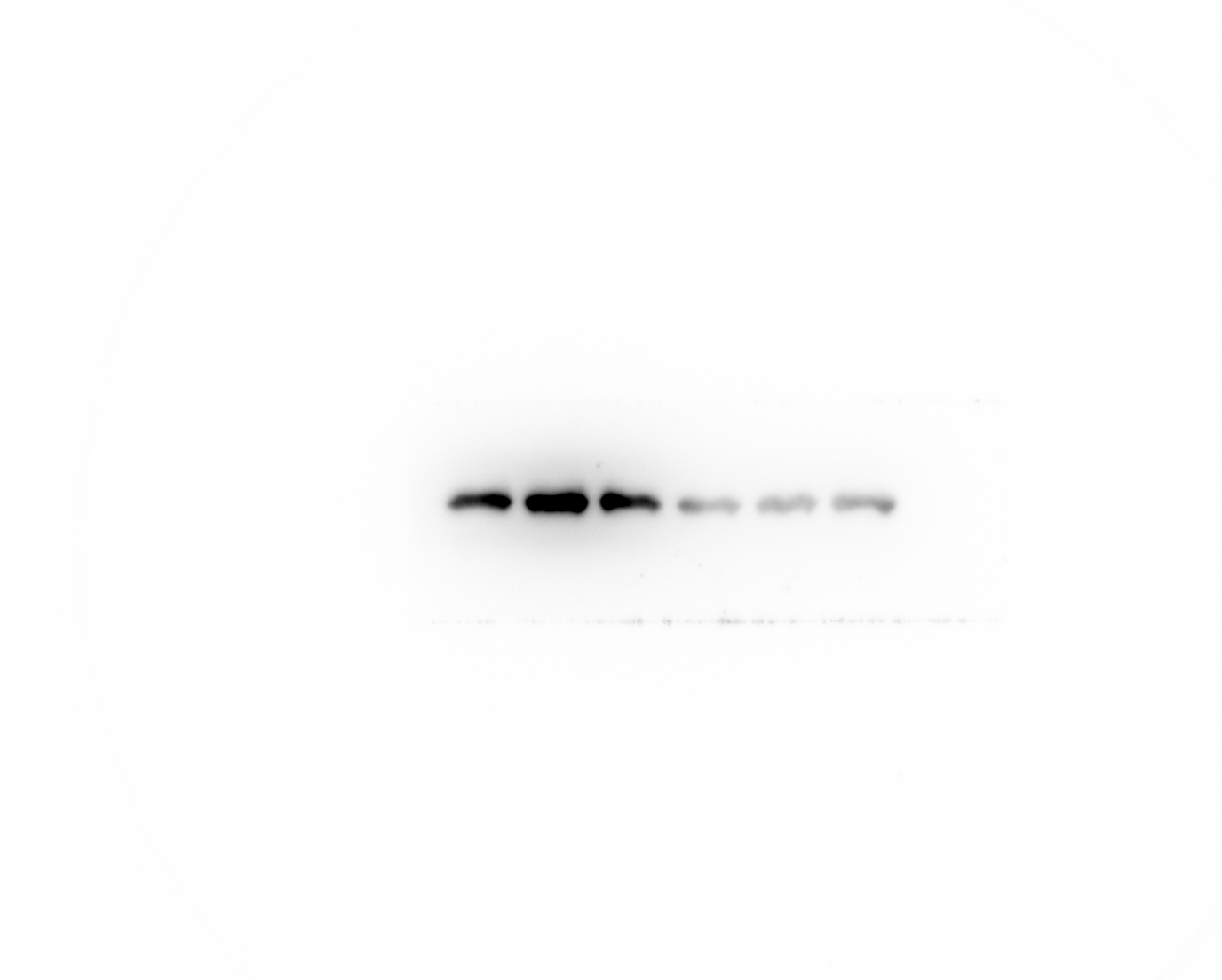

Supplement: Supplemental Information 5 [file peerj-13-19276-s005.zip › western blot-(CP IR group) EB1/western blot-(CP IR group) EB1-1/8-EB1.Tif]

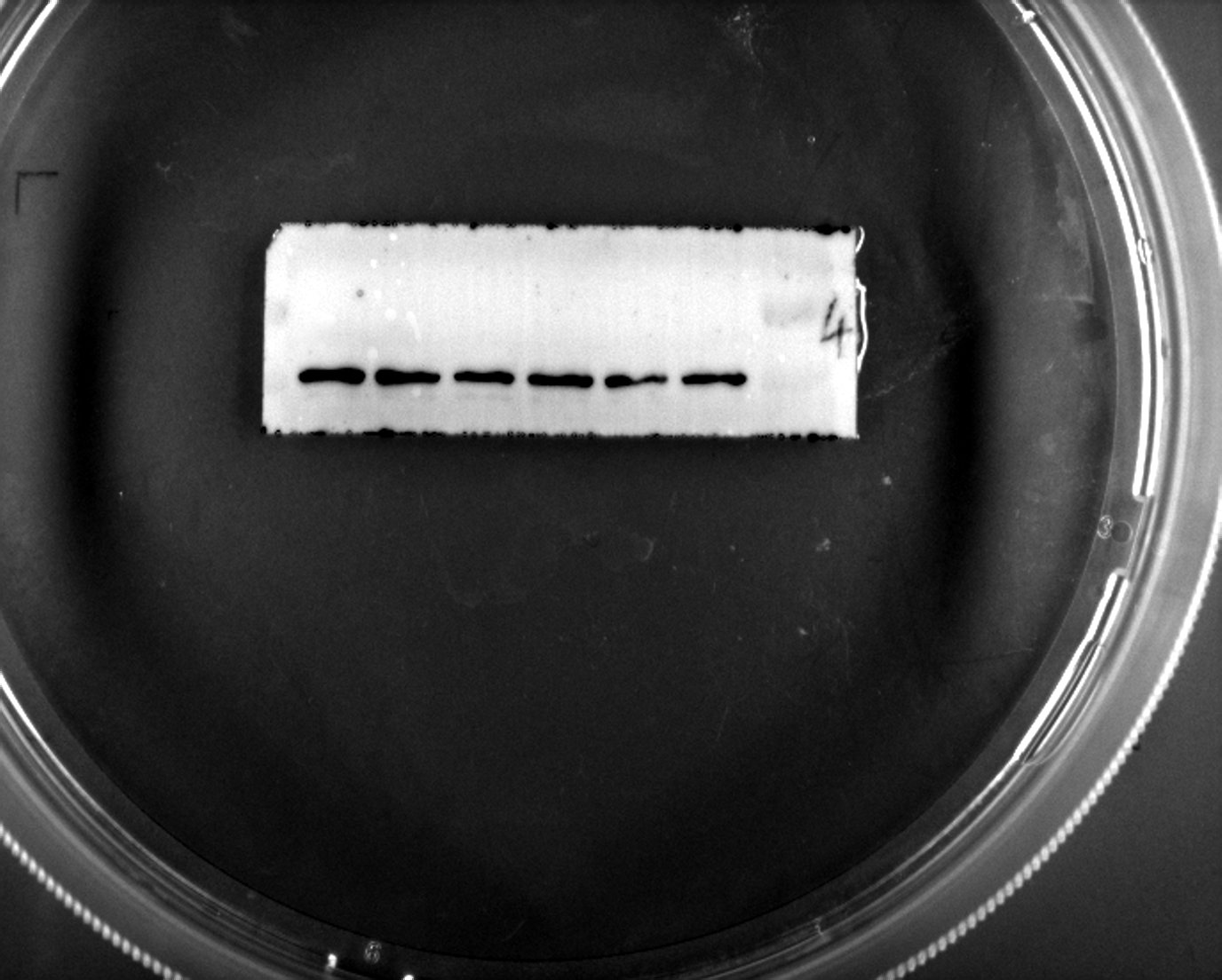

Supplement: Supplemental Information 5 [file peerj-13-19276-s005.zip › western blot-(CP IR group) EB1/western blot-(CP IR group) EB1-1/8-Tbulin-M.Tif]

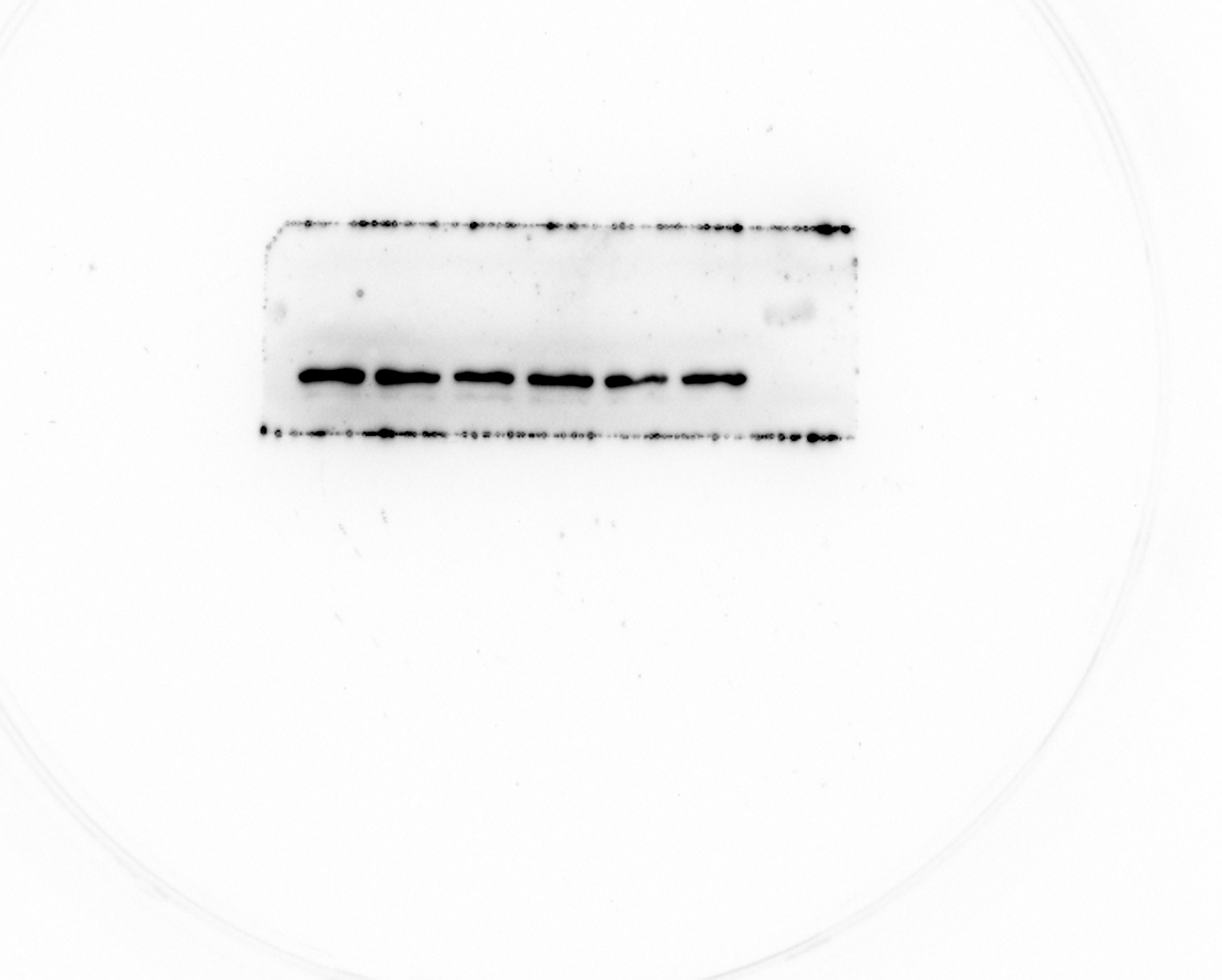

Supplement: Supplemental Information 5 [file peerj-13-19276-s005.zip › western blot-(CP IR group) EB1/western blot-(CP IR group) EB1-1/8-Tubulin.Tif]

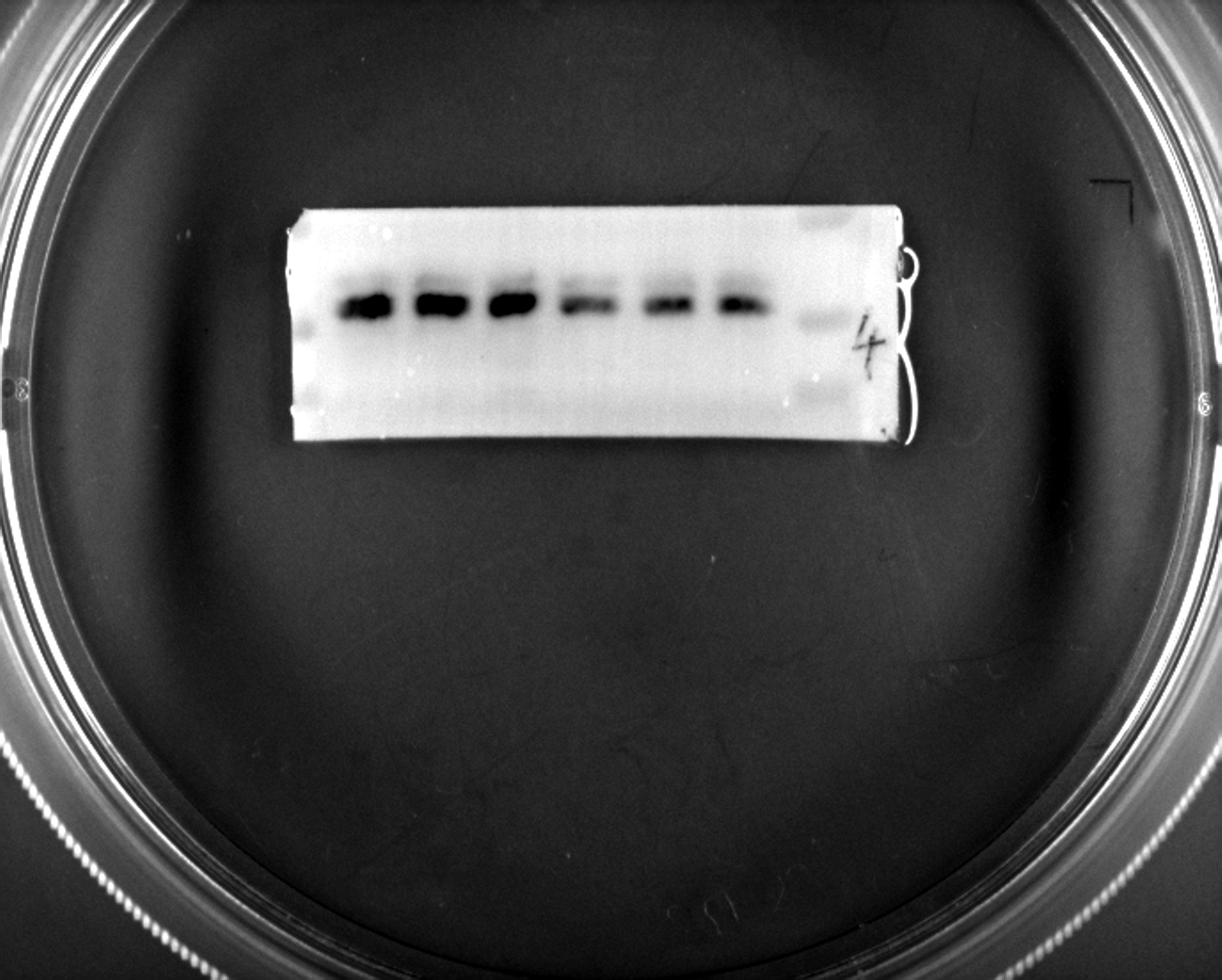

Supplement: Supplemental Information 5 [file peerj-13-19276-s005.zip › western blot-(CP IR group) EB1/western blot-(CP IR group) EB1-2/1-EB1-M.Tif]

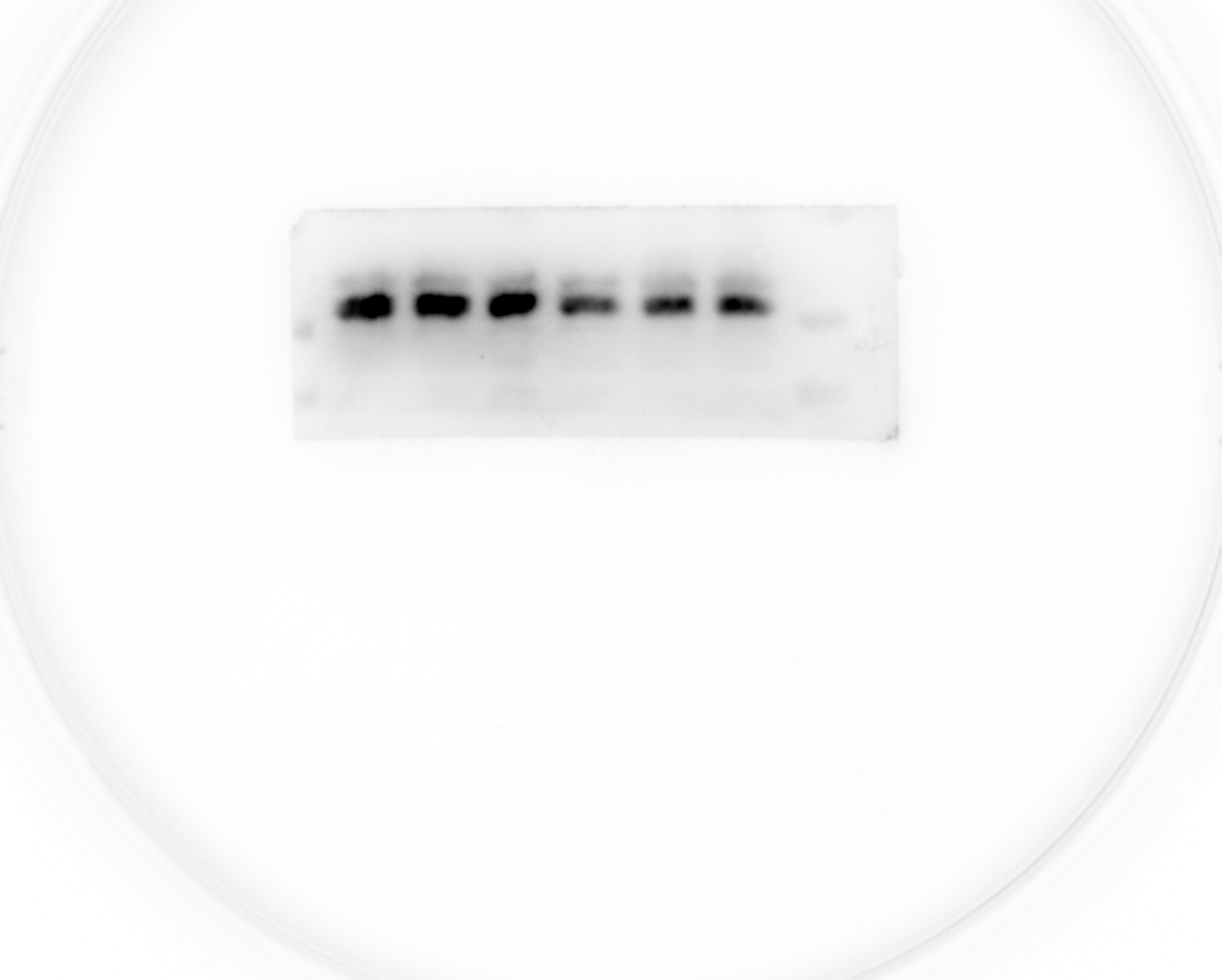

Supplement: Supplemental Information 5 [file peerj-13-19276-s005.zip › western blot-(CP IR group) EB1/western blot-(CP IR group) EB1-2/1-EB1.Tif]

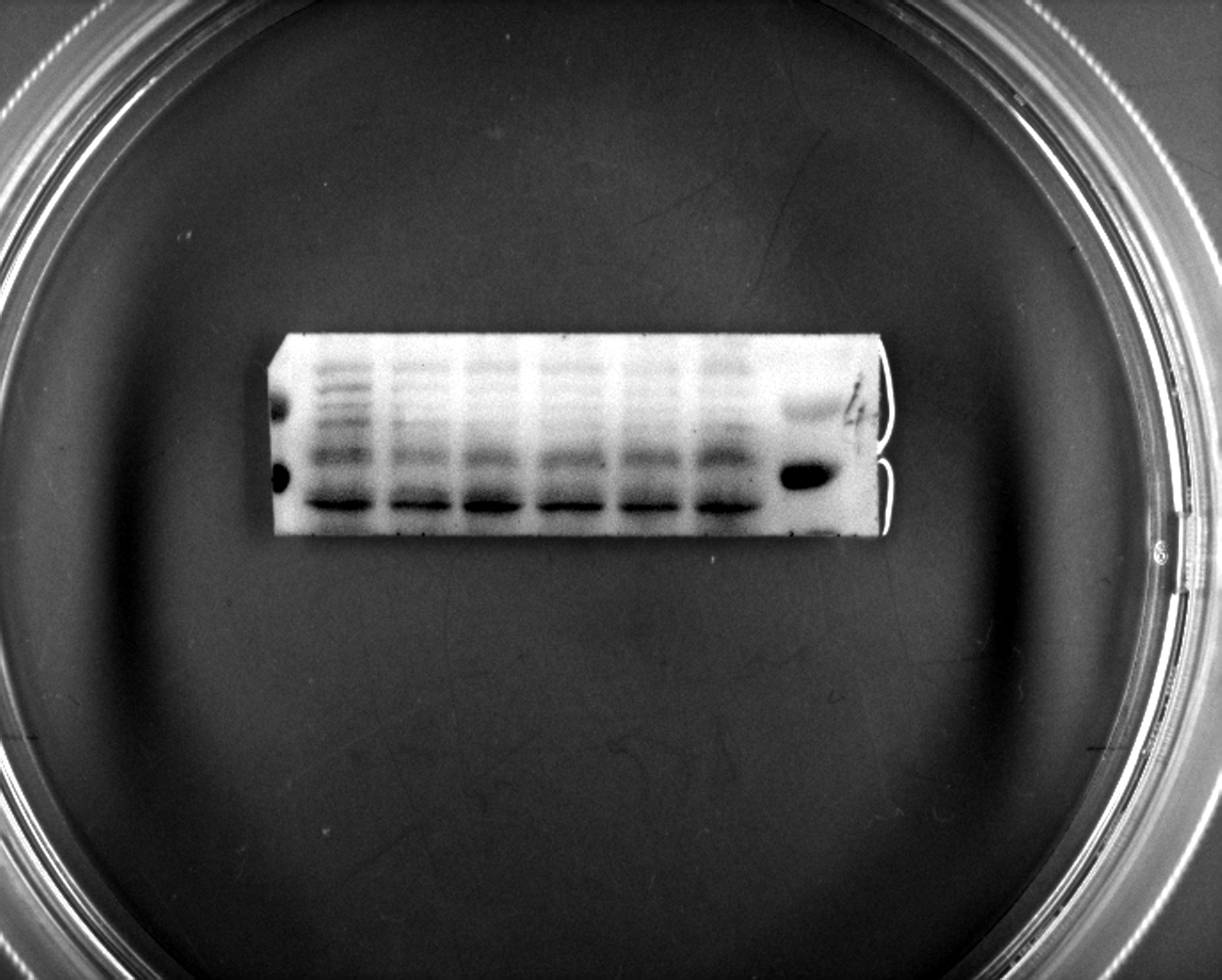

Supplement: Supplemental Information 5 [file peerj-13-19276-s005.zip › western blot-(CP IR group) EB1/western blot-(CP IR group) EB1-2/1-Tubulin-M.Tif]

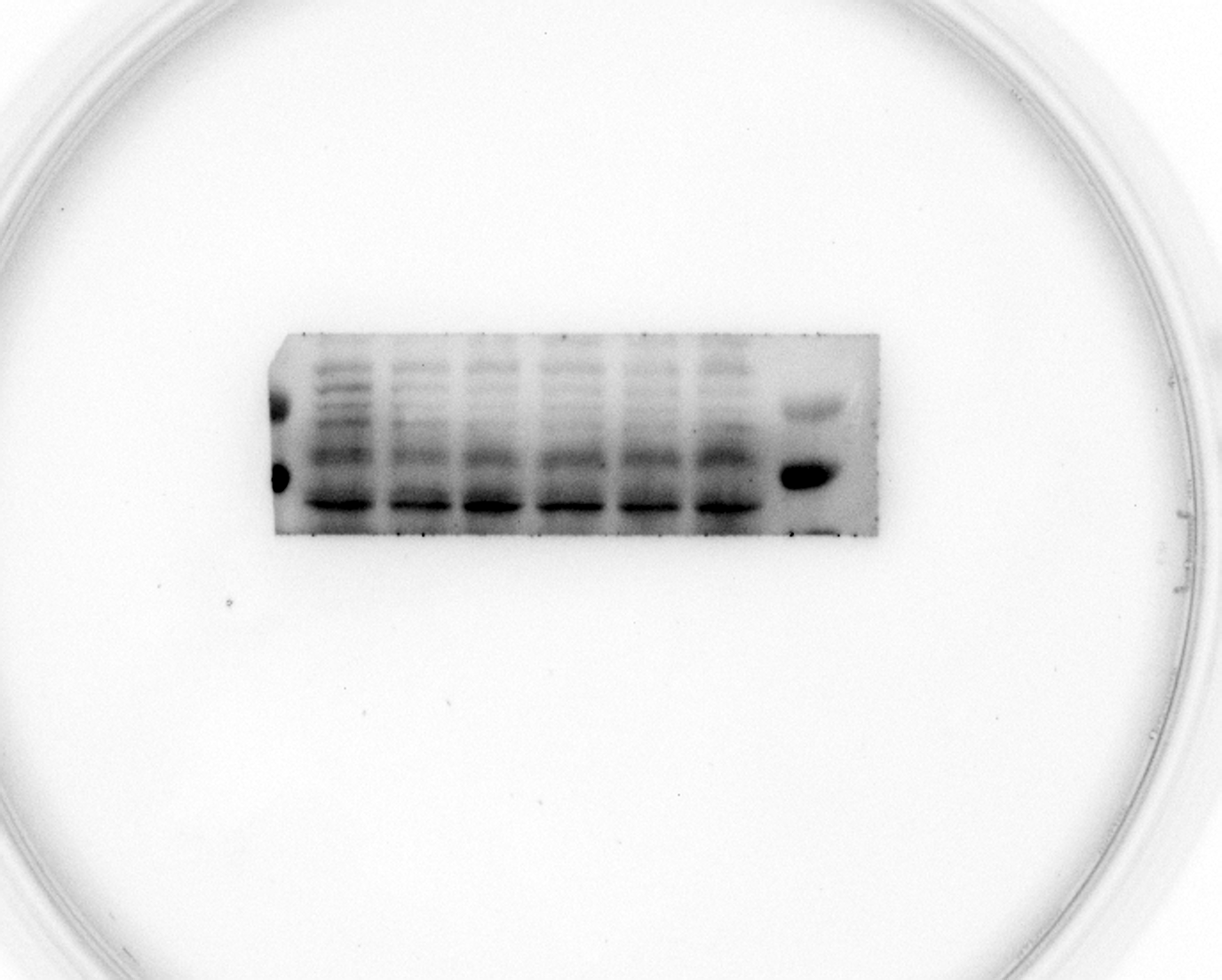

Supplement: Supplemental Information 5 [file peerj-13-19276-s005.zip › western blot-(CP IR group) EB1/western blot-(CP IR group) EB1-2/1-Tubulin.Tif]

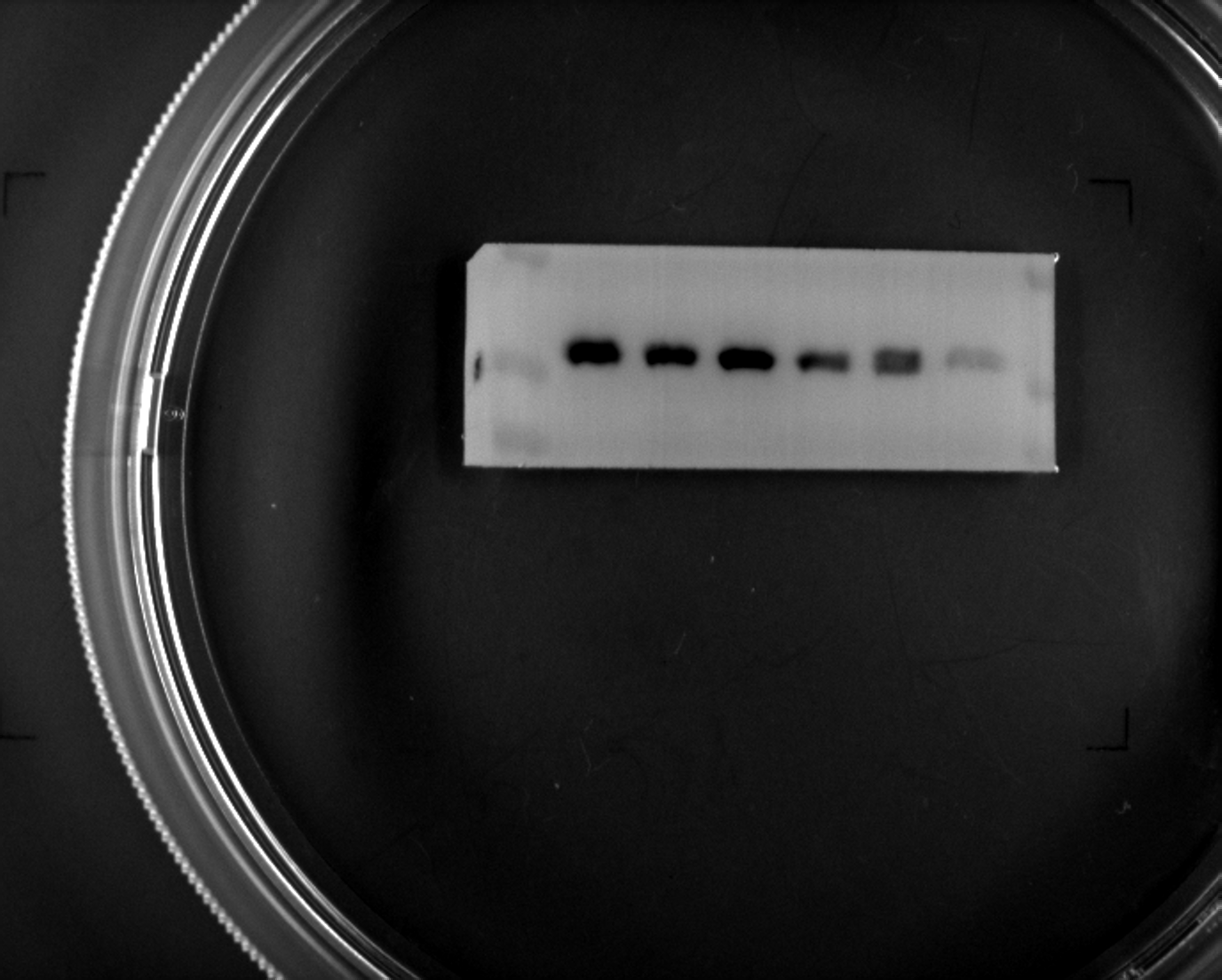

Supplement: Supplemental Information 5 [file peerj-13-19276-s005.zip › western blot-(CP IR group) EB1/western blot-(CP IR group) EB1-2/2-EB1-M.Tif]

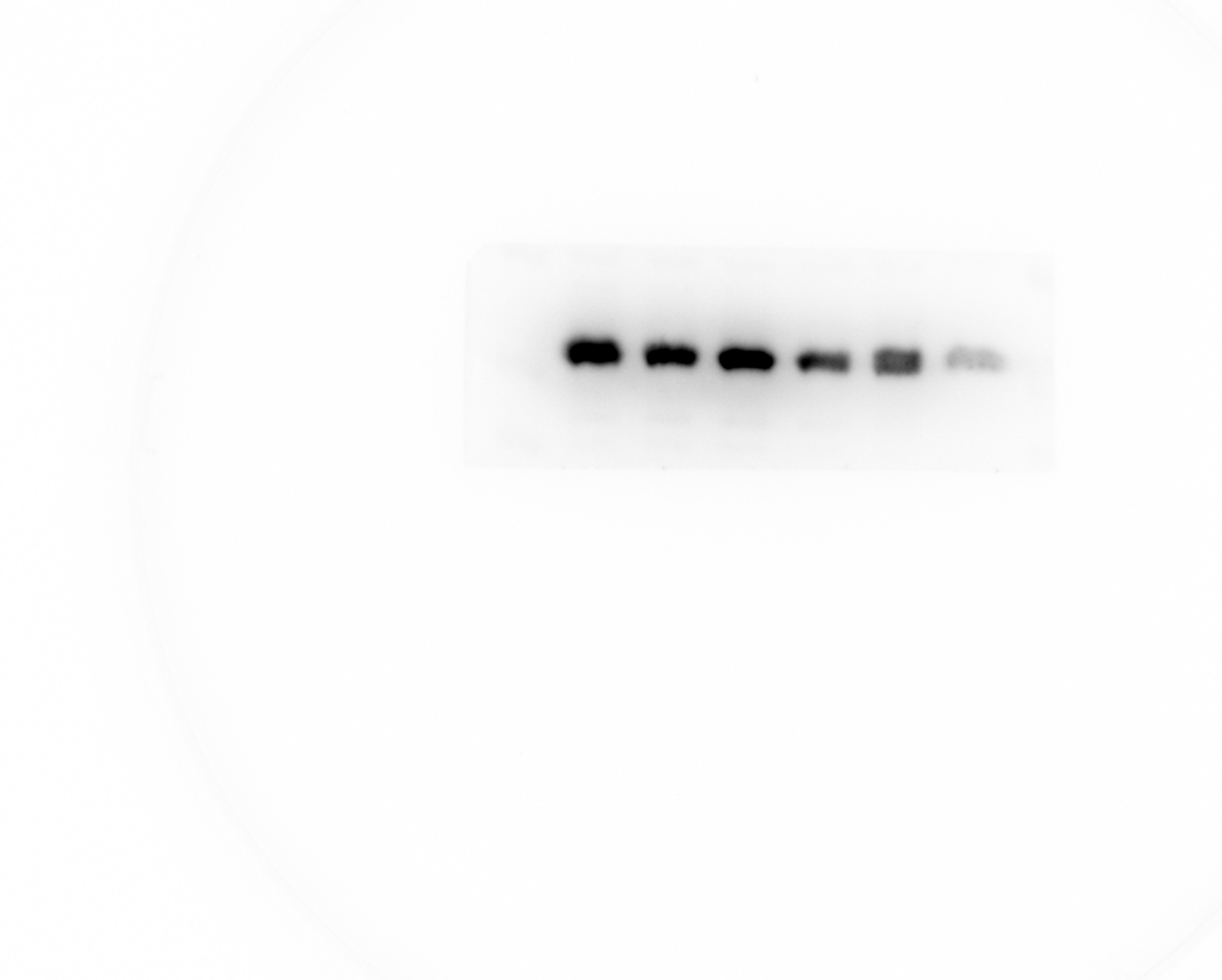

Supplement: Supplemental Information 5 [file peerj-13-19276-s005.zip › western blot-(CP IR group) EB1/western blot-(CP IR group) EB1-2/2-EB1.Tif]

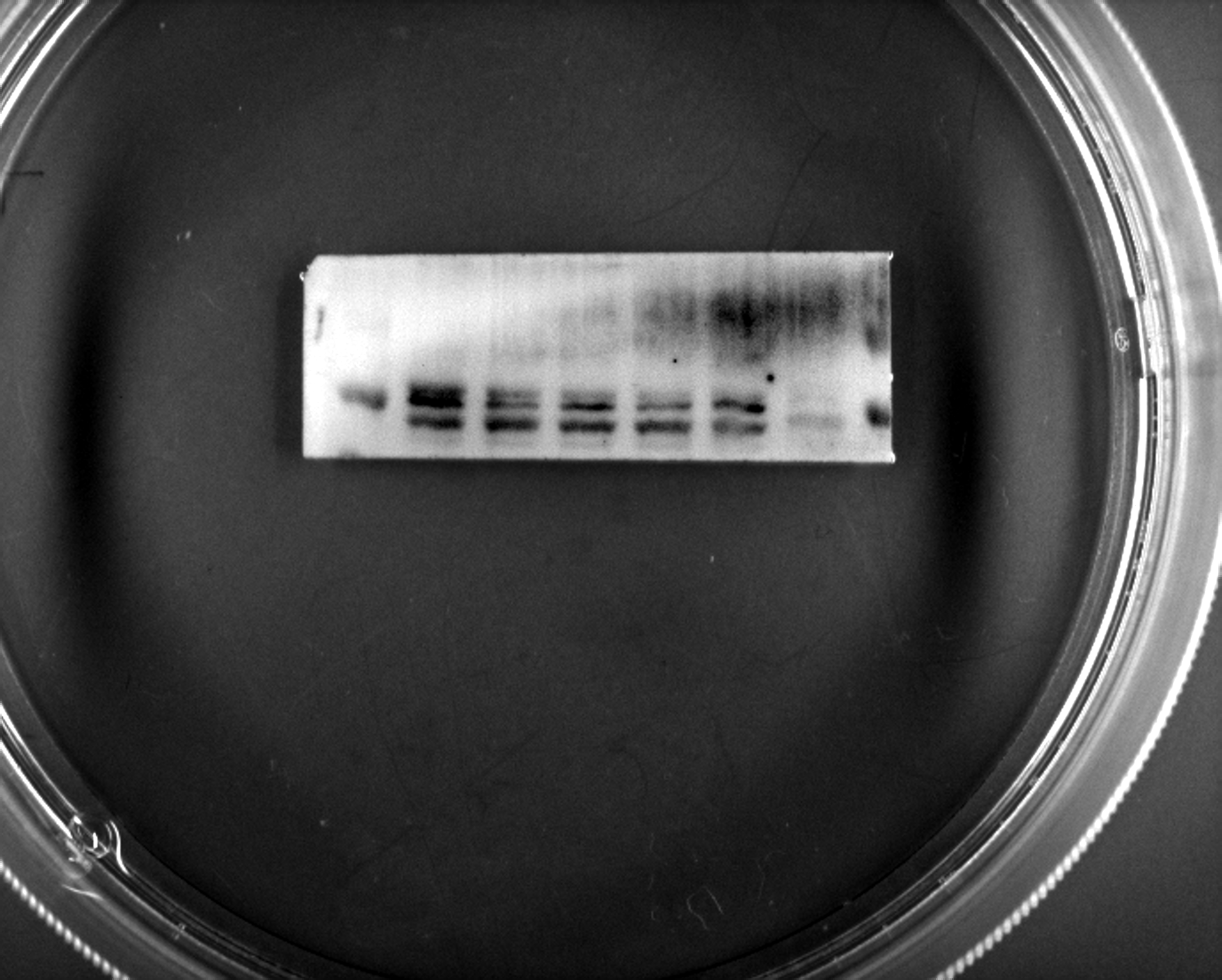

Supplement: Supplemental Information 5 [file peerj-13-19276-s005.zip › western blot-(CP IR group) EB1/western blot-(CP IR group) EB1-2/2-Tubulin-M.Tif]

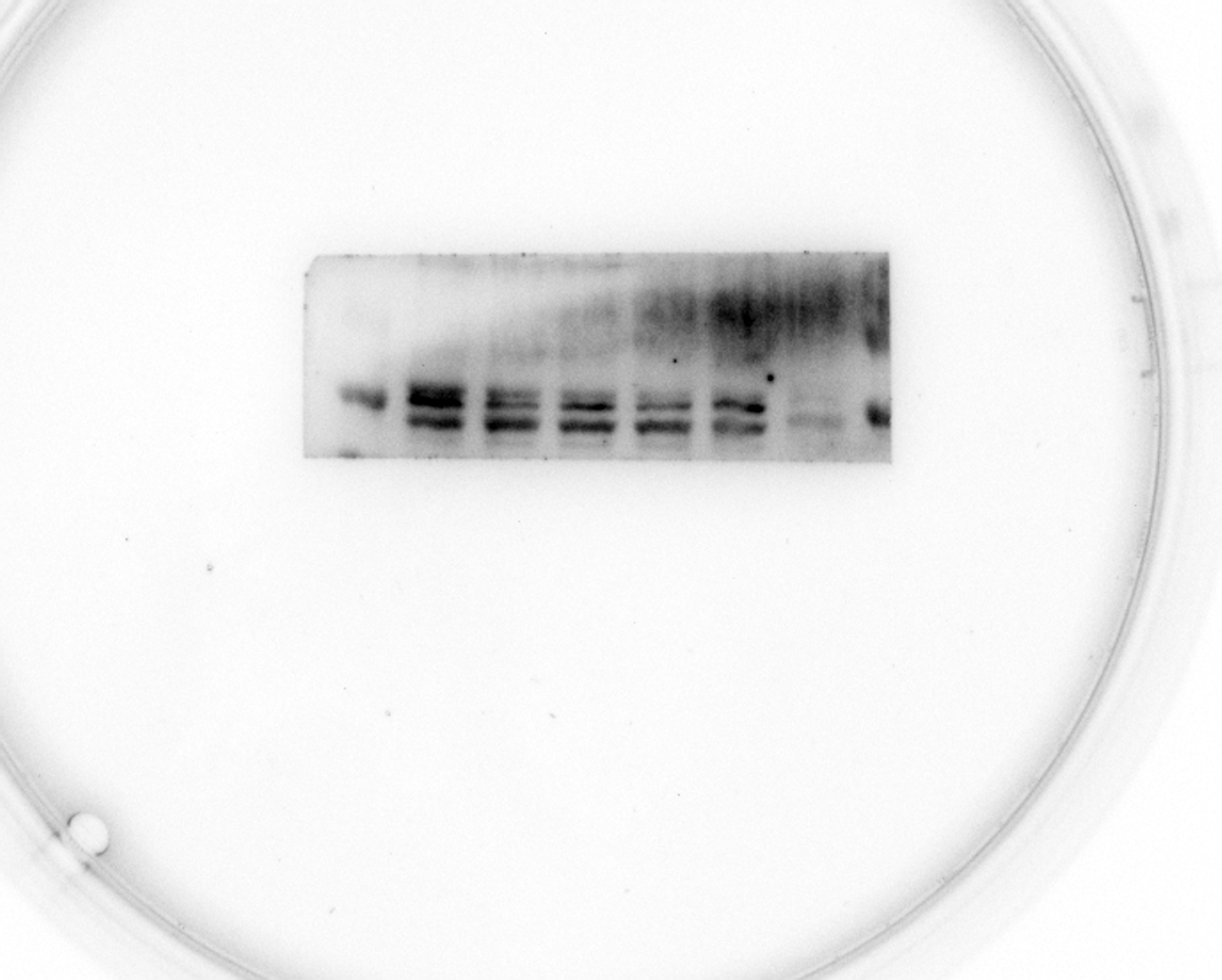

Supplement: Supplemental Information 5 [file peerj-13-19276-s005.zip › western blot-(CP IR group) EB1/western blot-(CP IR group) EB1-2/2-Tubulin.Tif]

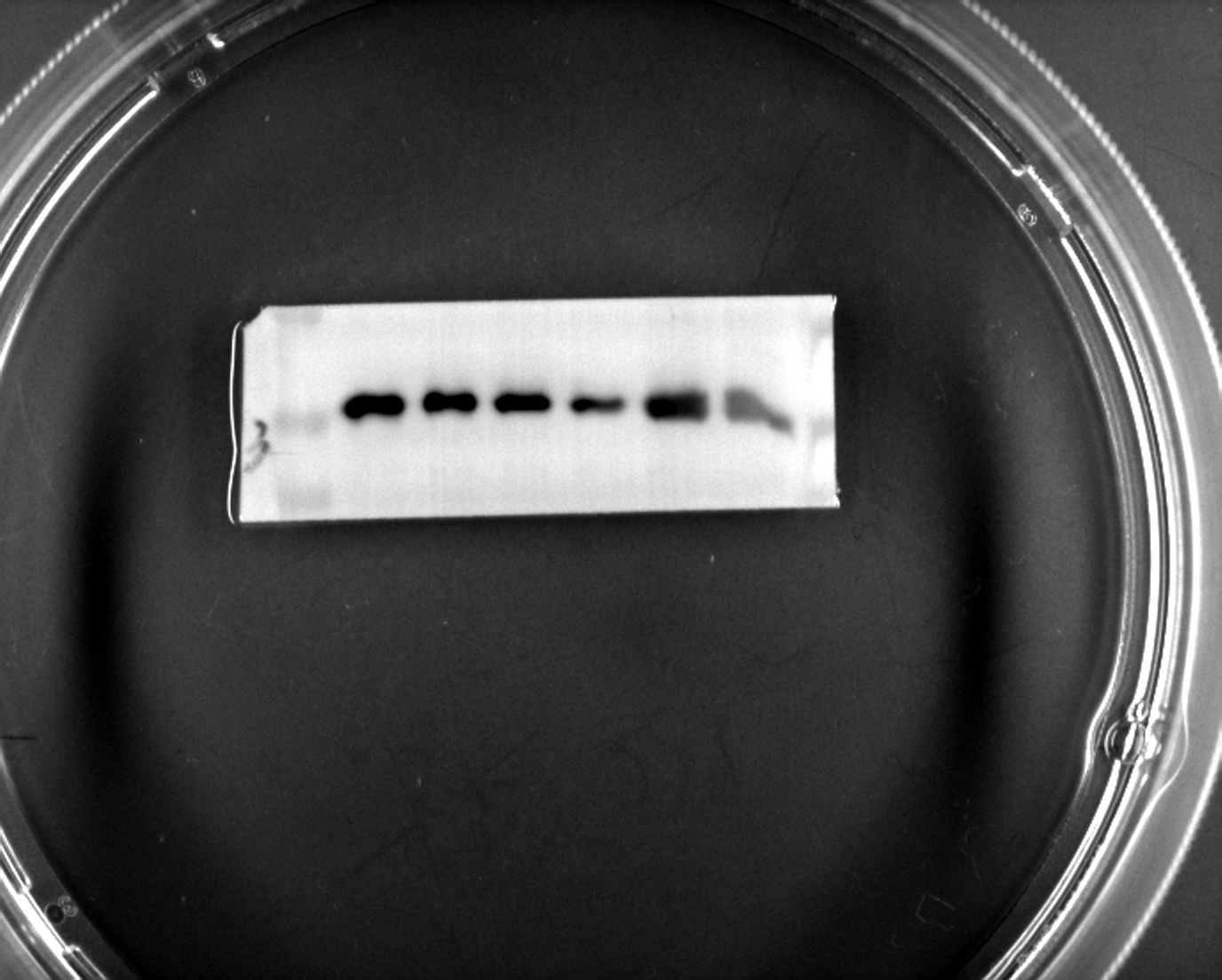

Supplement: Supplemental Information 5 [file peerj-13-19276-s005.zip › western blot-(CP IR group) EB1/western blot-(CP IR group) EB1-2/3-EB1-M.Tif]

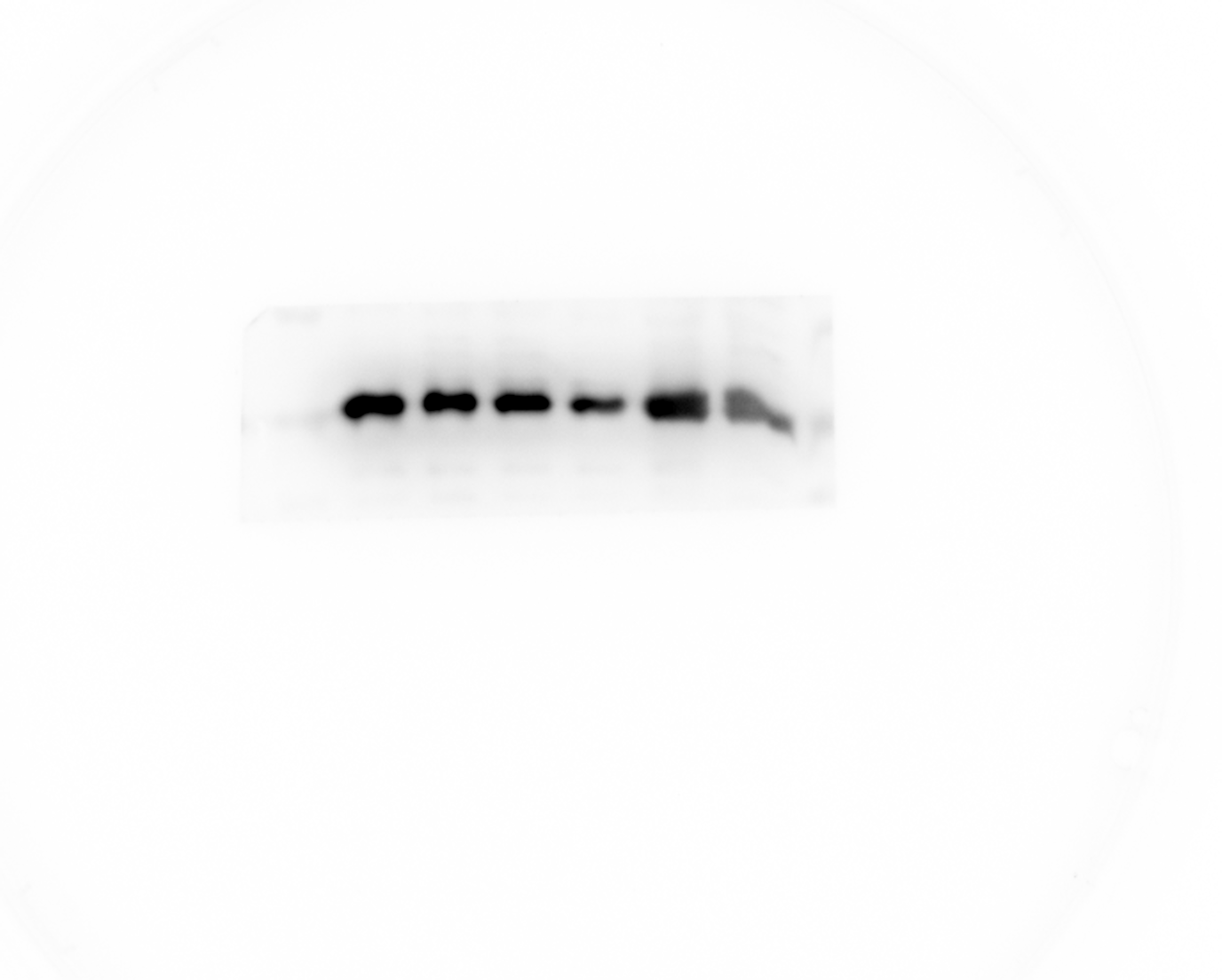

Supplement: Supplemental Information 5 [file peerj-13-19276-s005.zip › western blot-(CP IR group) EB1/western blot-(CP IR group) EB1-2/3-EB1.Tif]

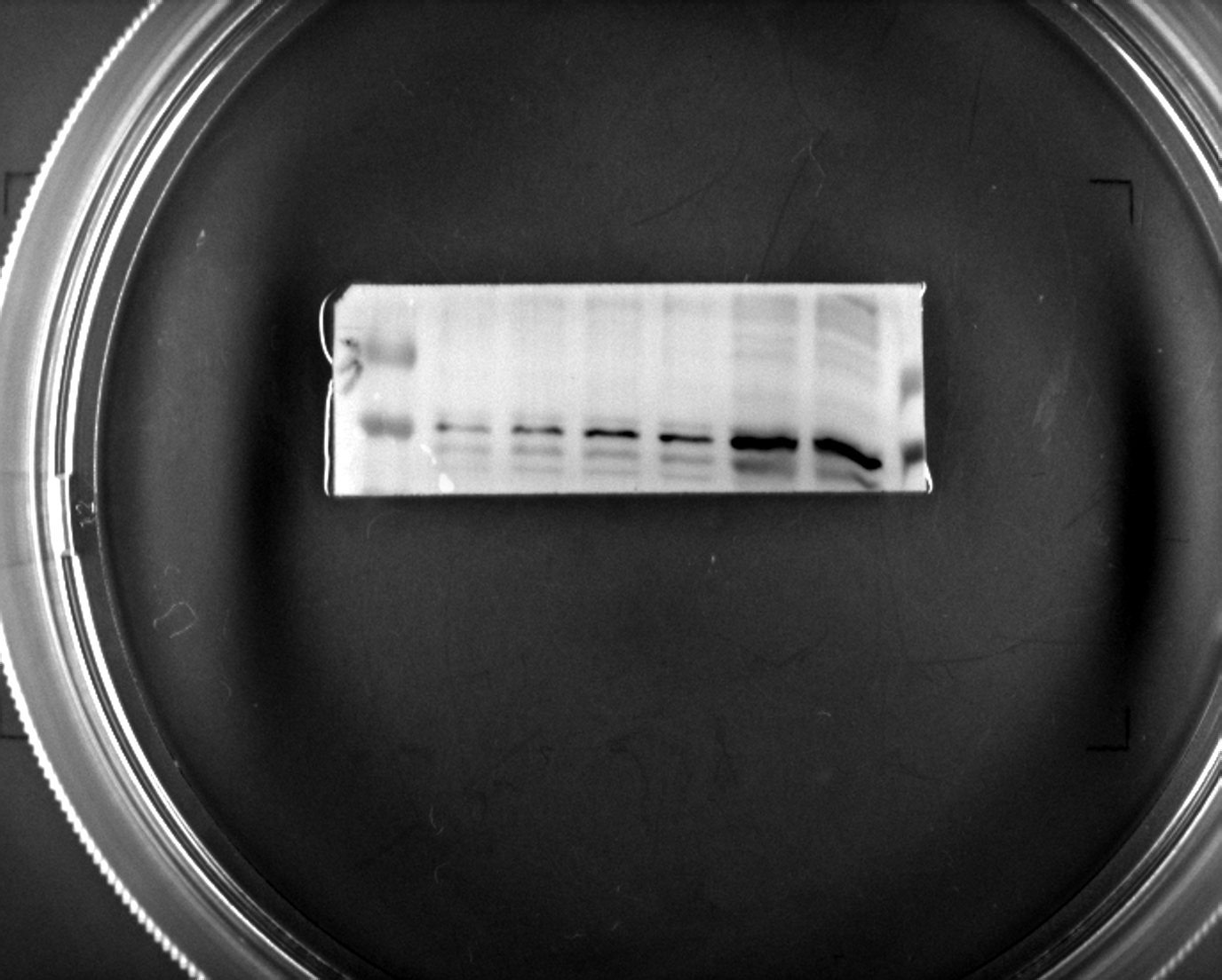

Supplement: Supplemental Information 5 [file peerj-13-19276-s005.zip › western blot-(CP IR group) EB1/western blot-(CP IR group) EB1-2/3-Tubulin-M.Tif]

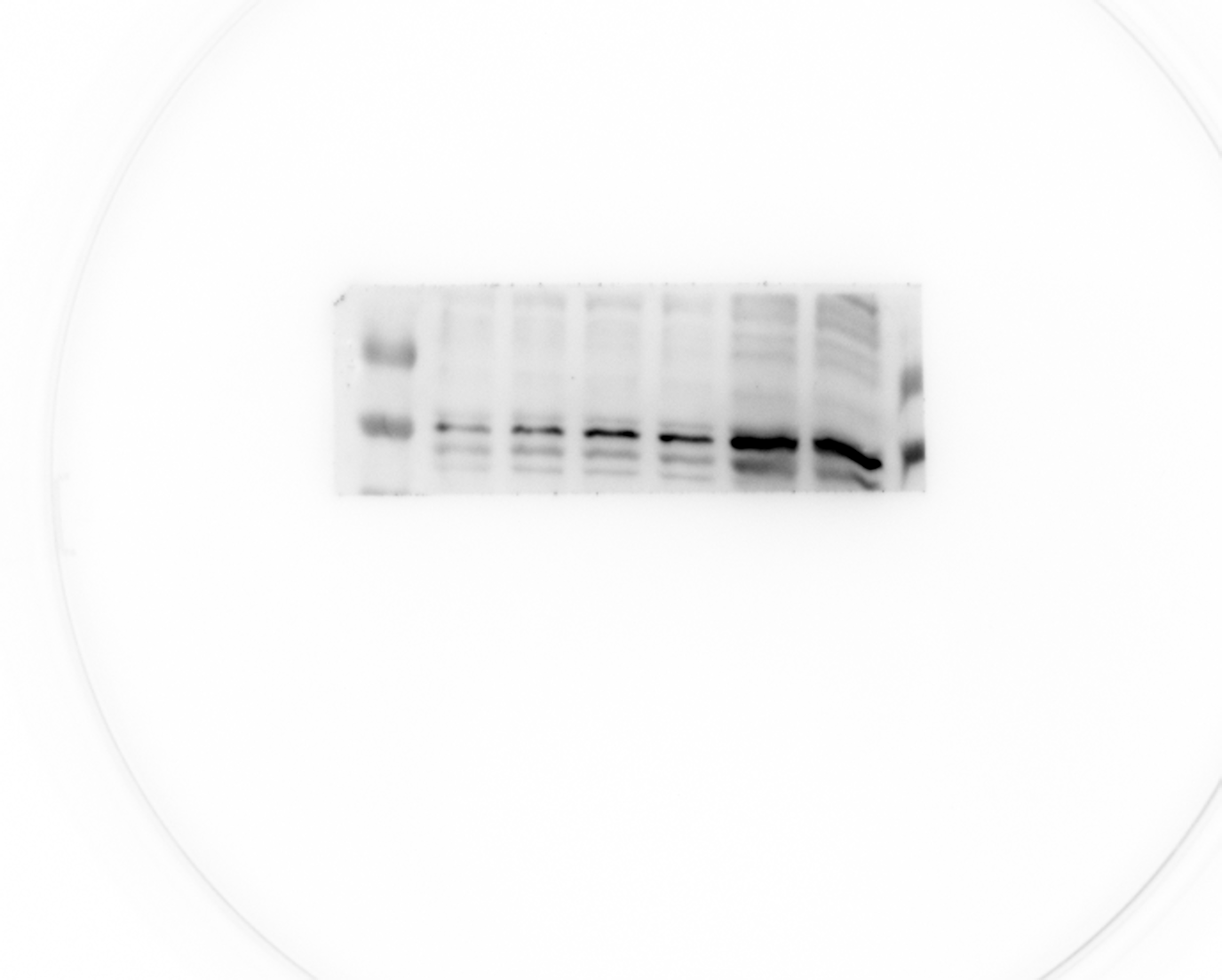

Supplement: Supplemental Information 5 [file peerj-13-19276-s005.zip › western blot-(CP IR group) EB1/western blot-(CP IR group) EB1-2/3-Tubulin.Tif]

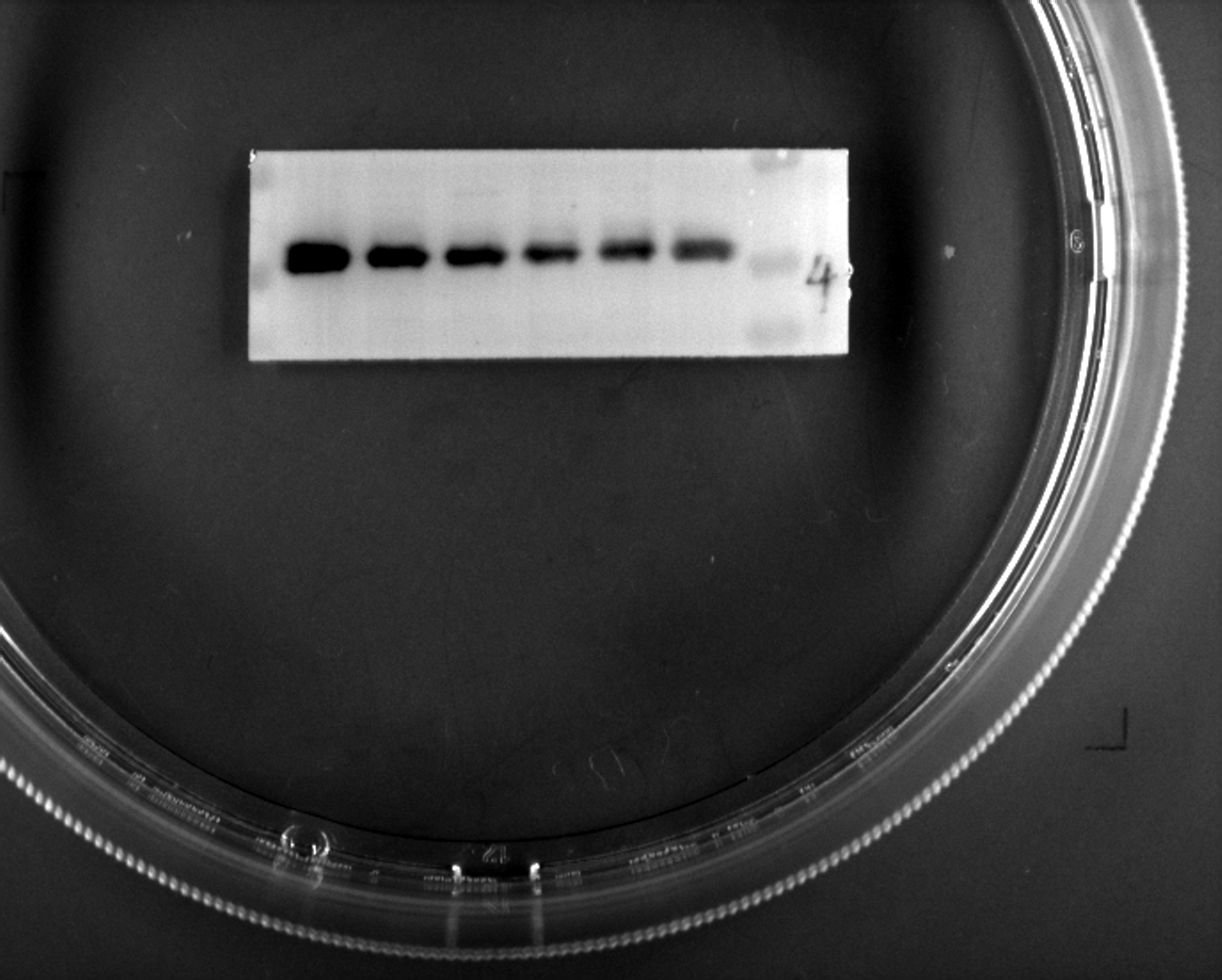

Supplement: Supplemental Information 5 [file peerj-13-19276-s005.zip › western blot-(CP IR group) EB1/western blot-(CP IR group) EB1-2/4-EB1-M-used.Tif]

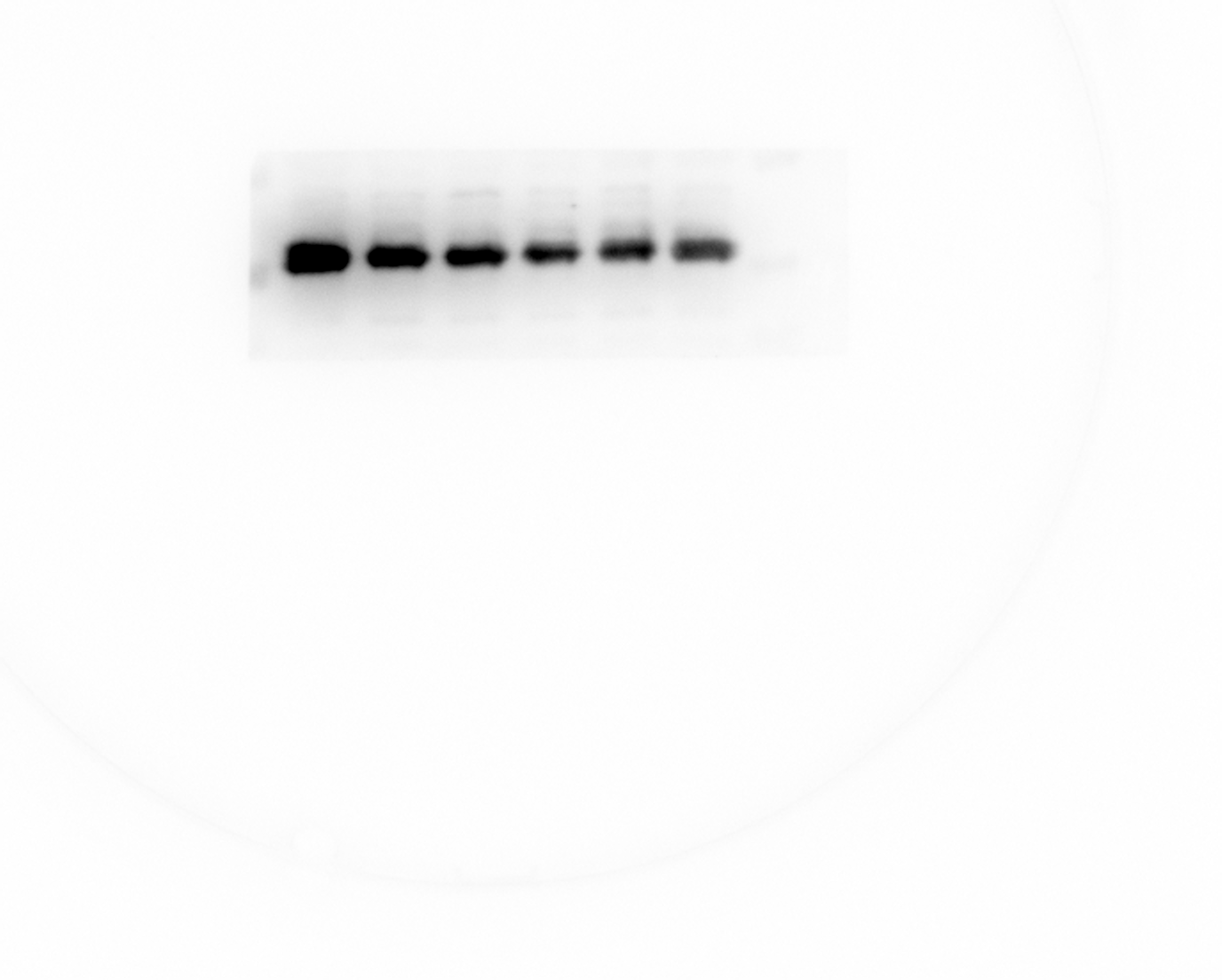

Supplement: Supplemental Information 5 [file peerj-13-19276-s005.zip › western blot-(CP IR group) EB1/western blot-(CP IR group) EB1-2/4-EB1-used.Tif]

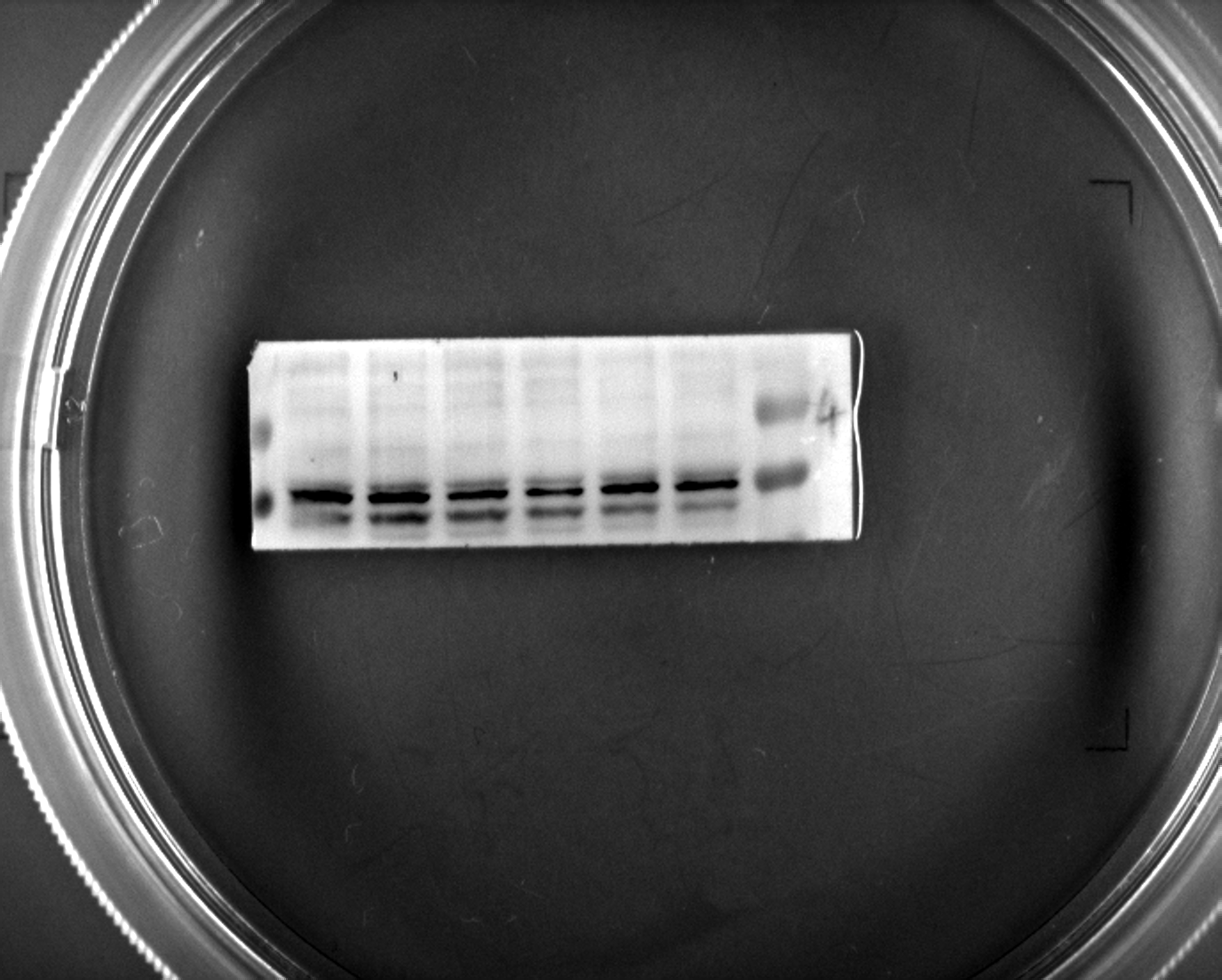

Supplement: Supplemental Information 5 [file peerj-13-19276-s005.zip › western blot-(CP IR group) EB1/western blot-(CP IR group) EB1-2/4-Tubulin-M-used.Tif]

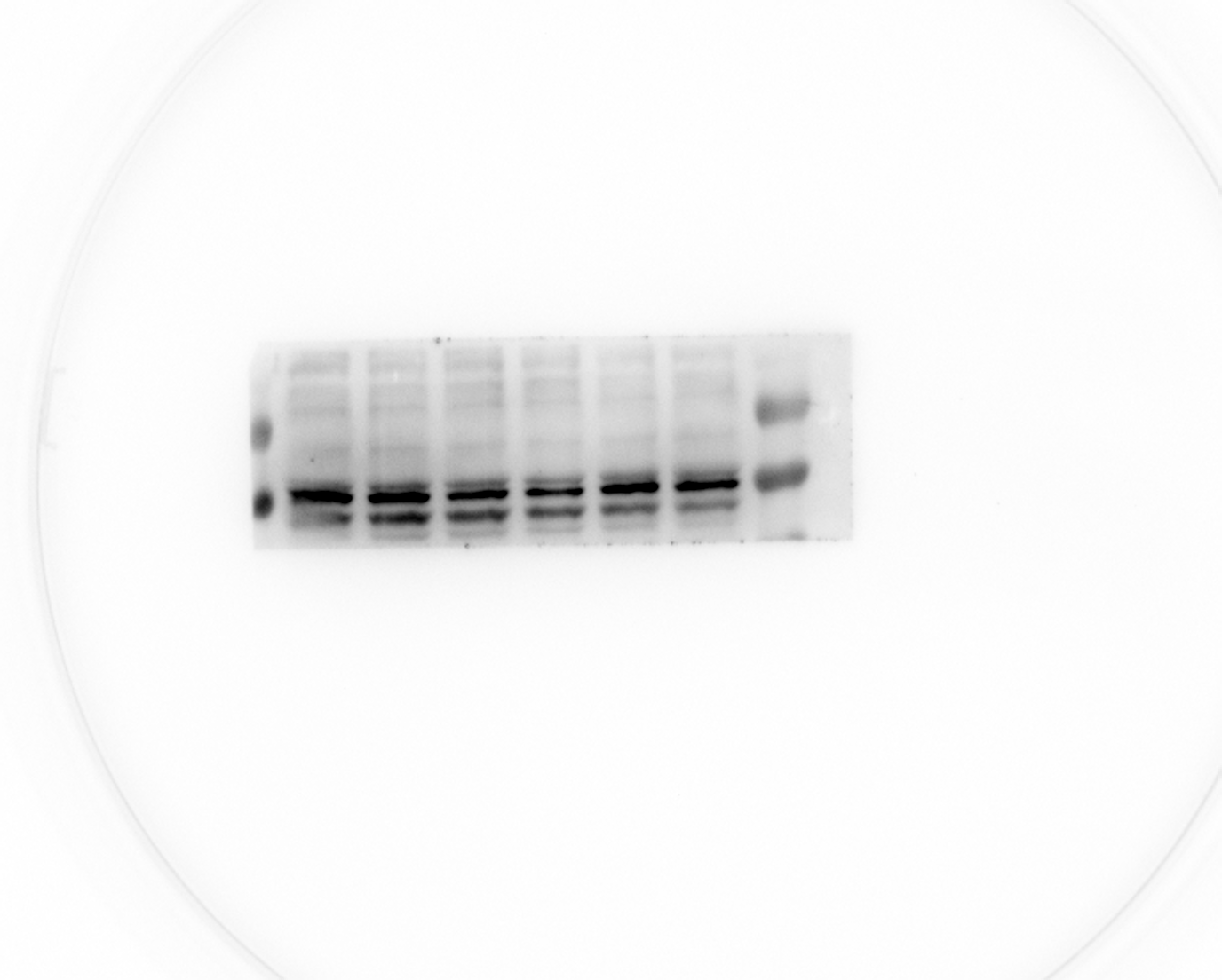

Supplement: Supplemental Information 5 [file peerj-13-19276-s005.zip › western blot-(CP IR group) EB1/western blot-(CP IR group) EB1-2/4-Tubulin-used.Tif]

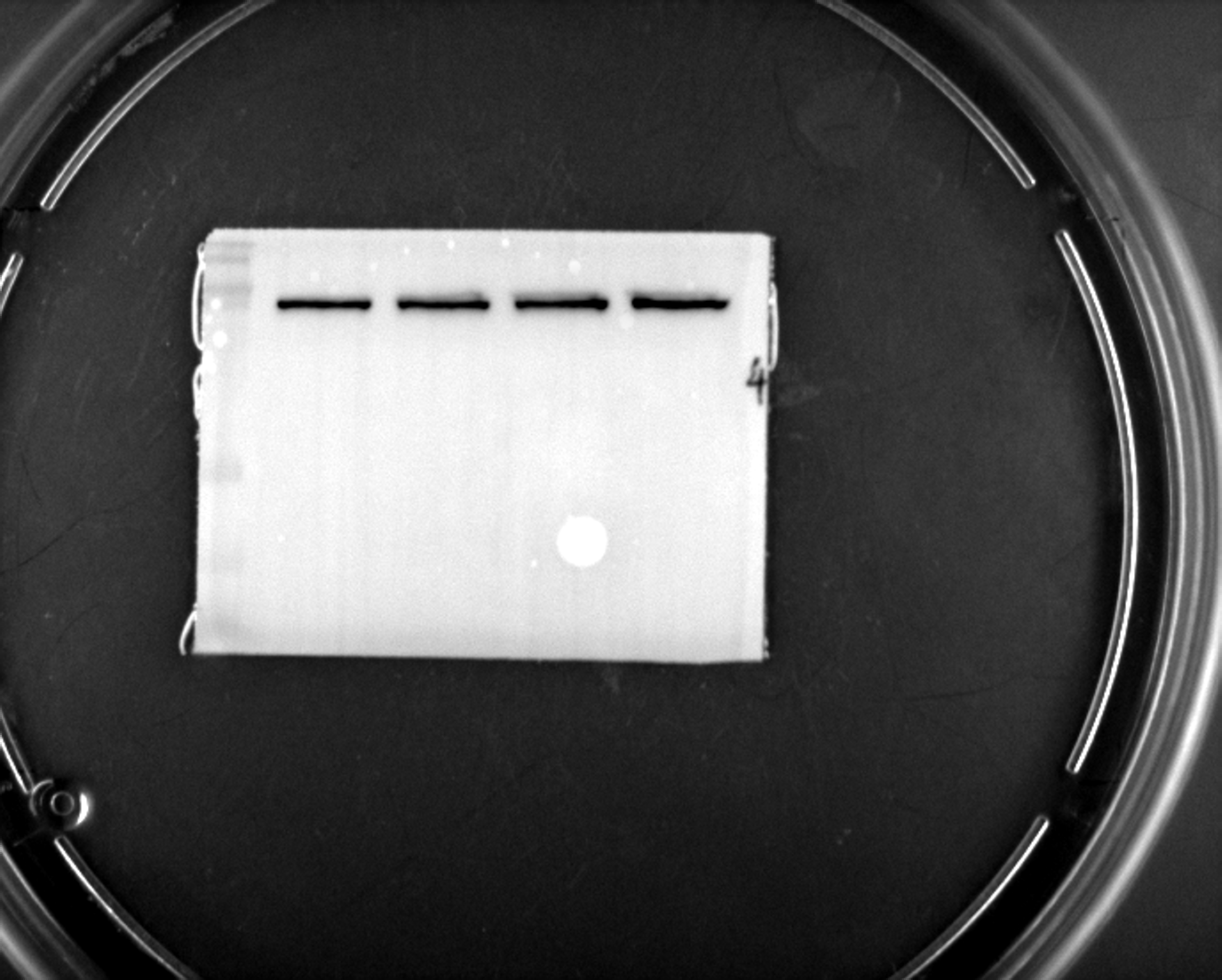

Supplement: Supplemental Information 6 [file peerj-13-19276-s006.zip › C I-R AAV9-CON AAV9-EB1 group western blot-membranal Cx43/1-ATPase-M-used.Tif]

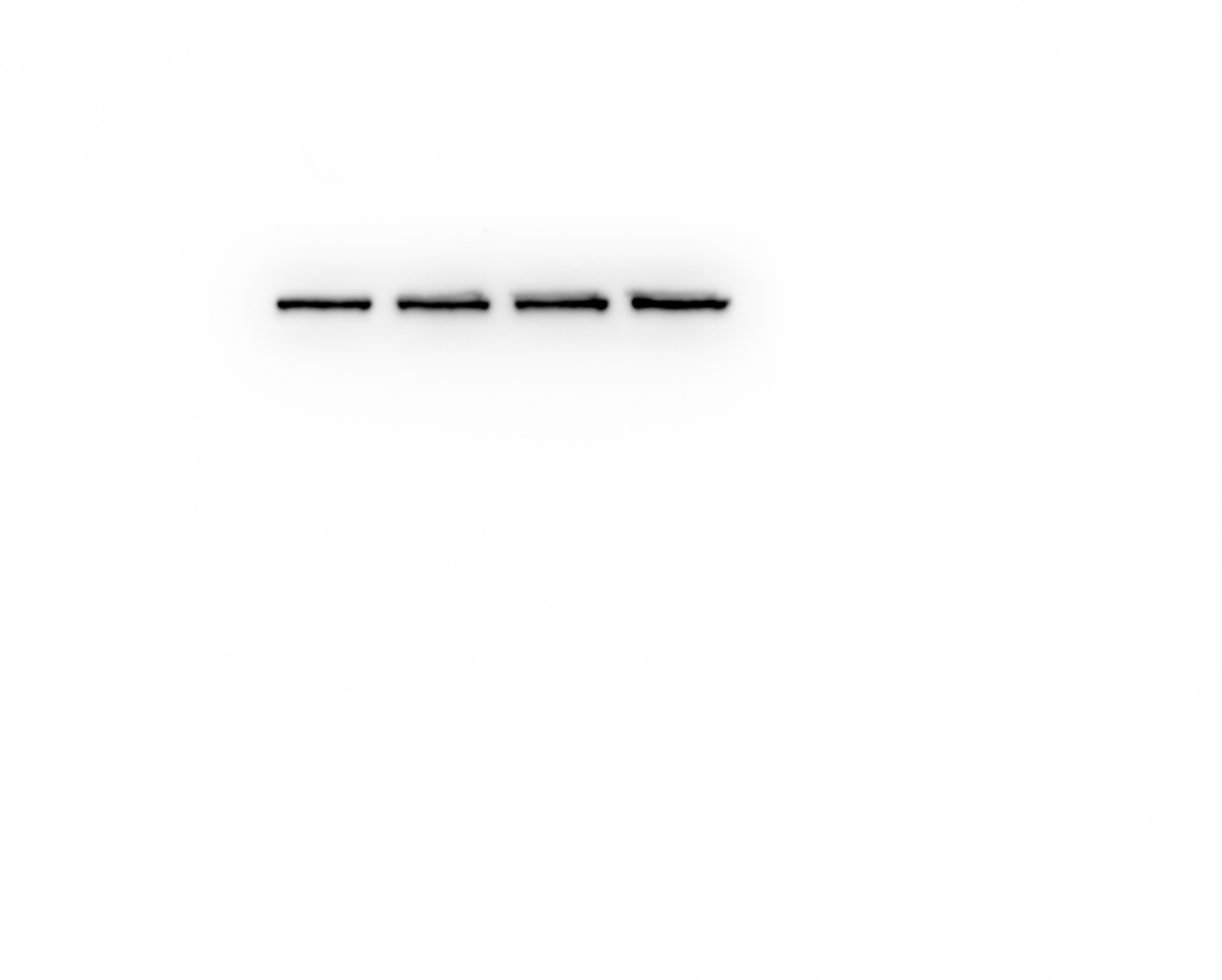

Supplement: Supplemental Information 6 [file peerj-13-19276-s006.zip › C I-R AAV9-CON AAV9-EB1 group western blot-membranal Cx43/1-ATPase-used.Tif]

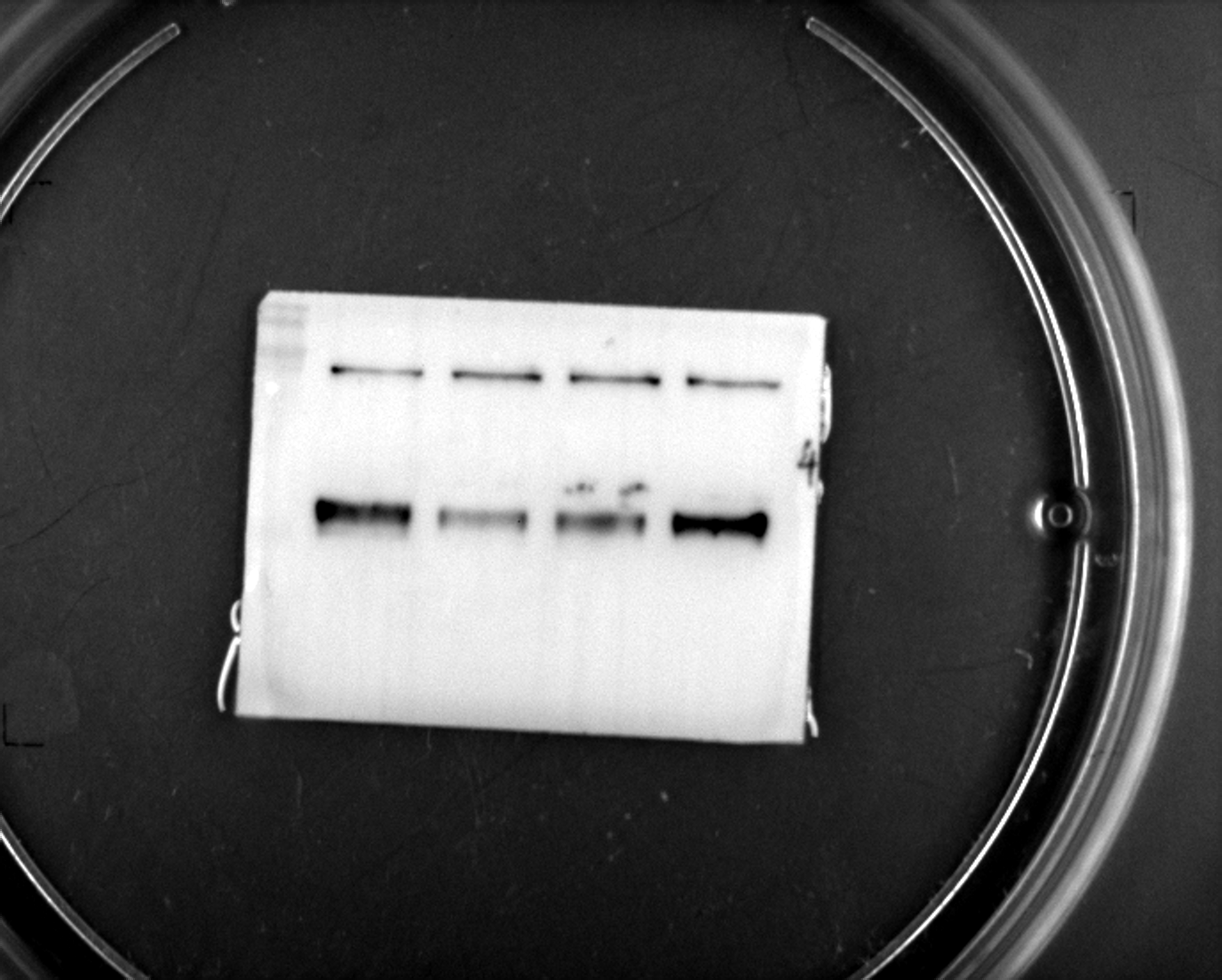

Supplement: Supplemental Information 6 [file peerj-13-19276-s006.zip › C I-R AAV9-CON AAV9-EB1 group western blot-membranal Cx43/1-Cx43-M-used.Tif]

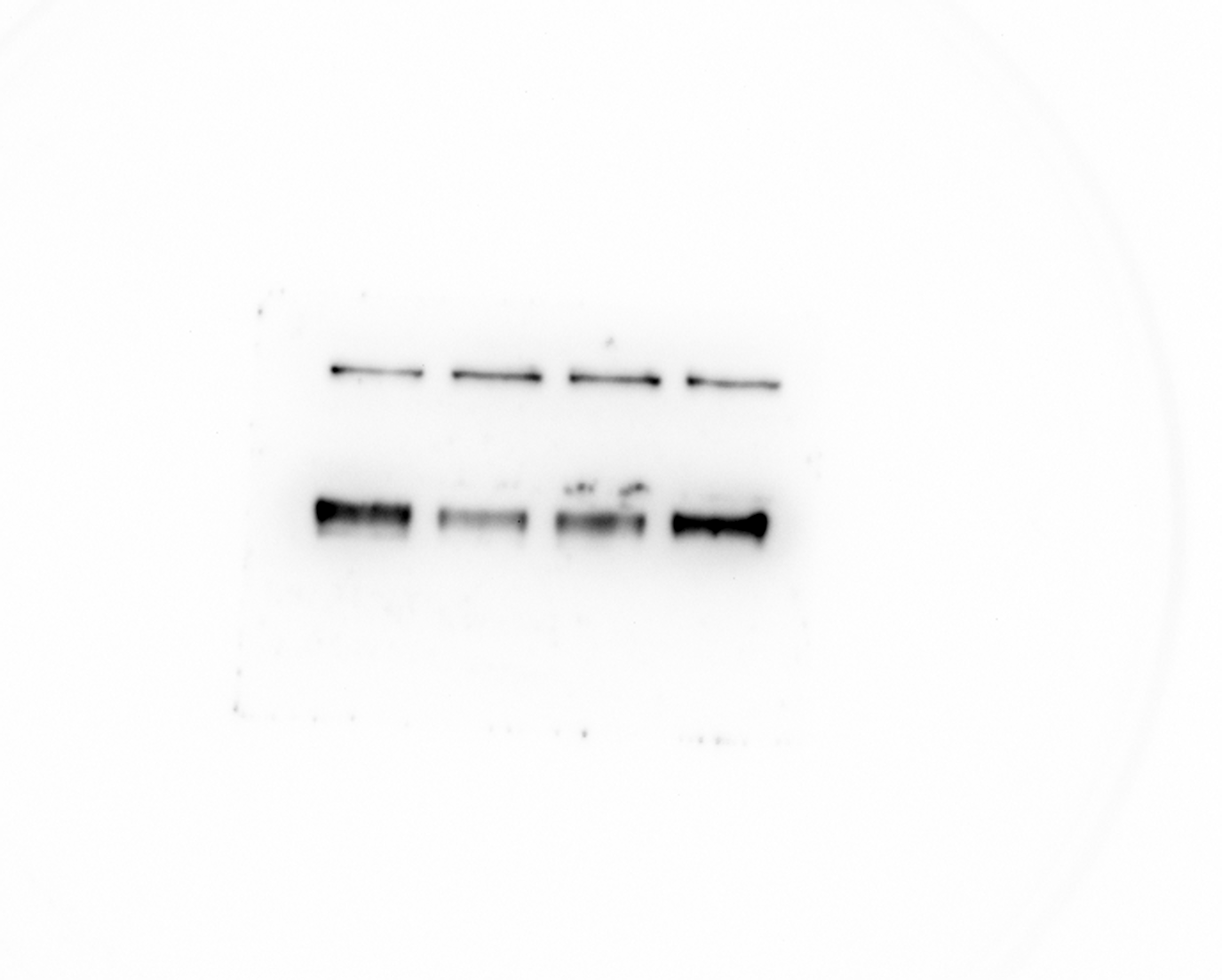

Supplement: Supplemental Information 6 [file peerj-13-19276-s006.zip › C I-R AAV9-CON AAV9-EB1 group western blot-membranal Cx43/1-Cx43-used.Tif]

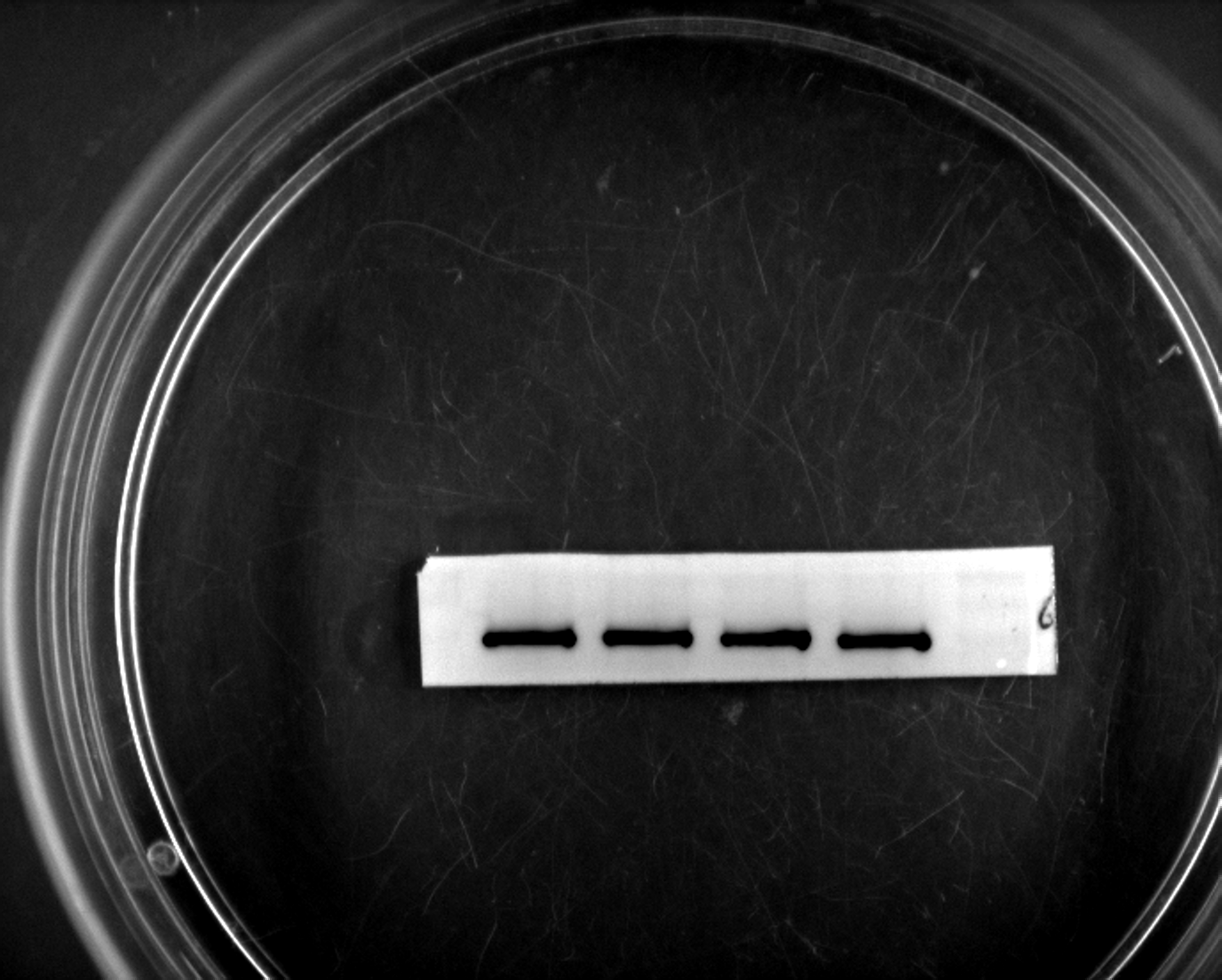

Supplement: Supplemental Information 6 [file peerj-13-19276-s006.zip › C I-R AAV9-CON AAV9-EB1 group western blot-membranal Cx43/2-ATPase-M.Tif]

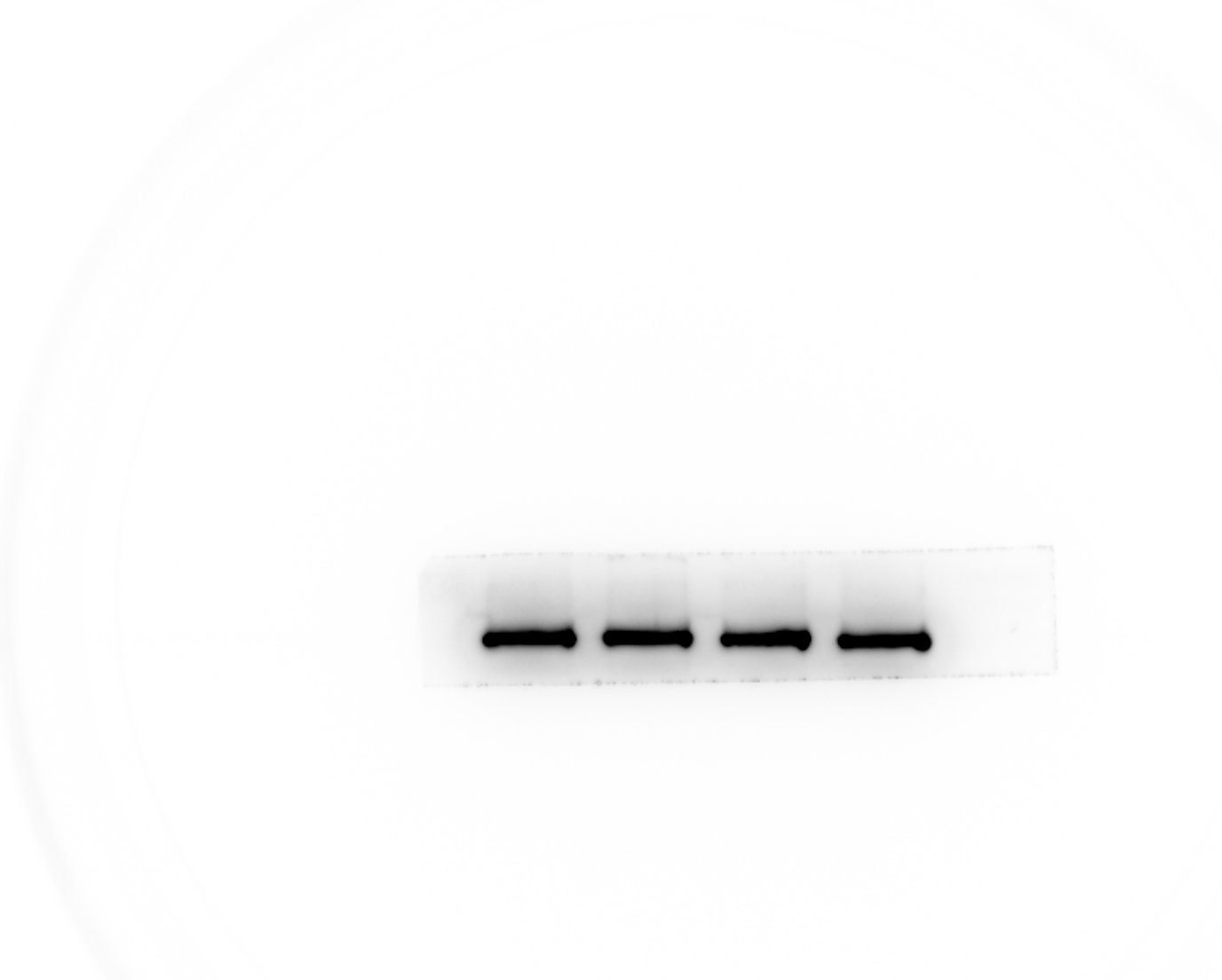

Supplement: Supplemental Information 6 [file peerj-13-19276-s006.zip › C I-R AAV9-CON AAV9-EB1 group western blot-membranal Cx43/2-ATPase.Tif]

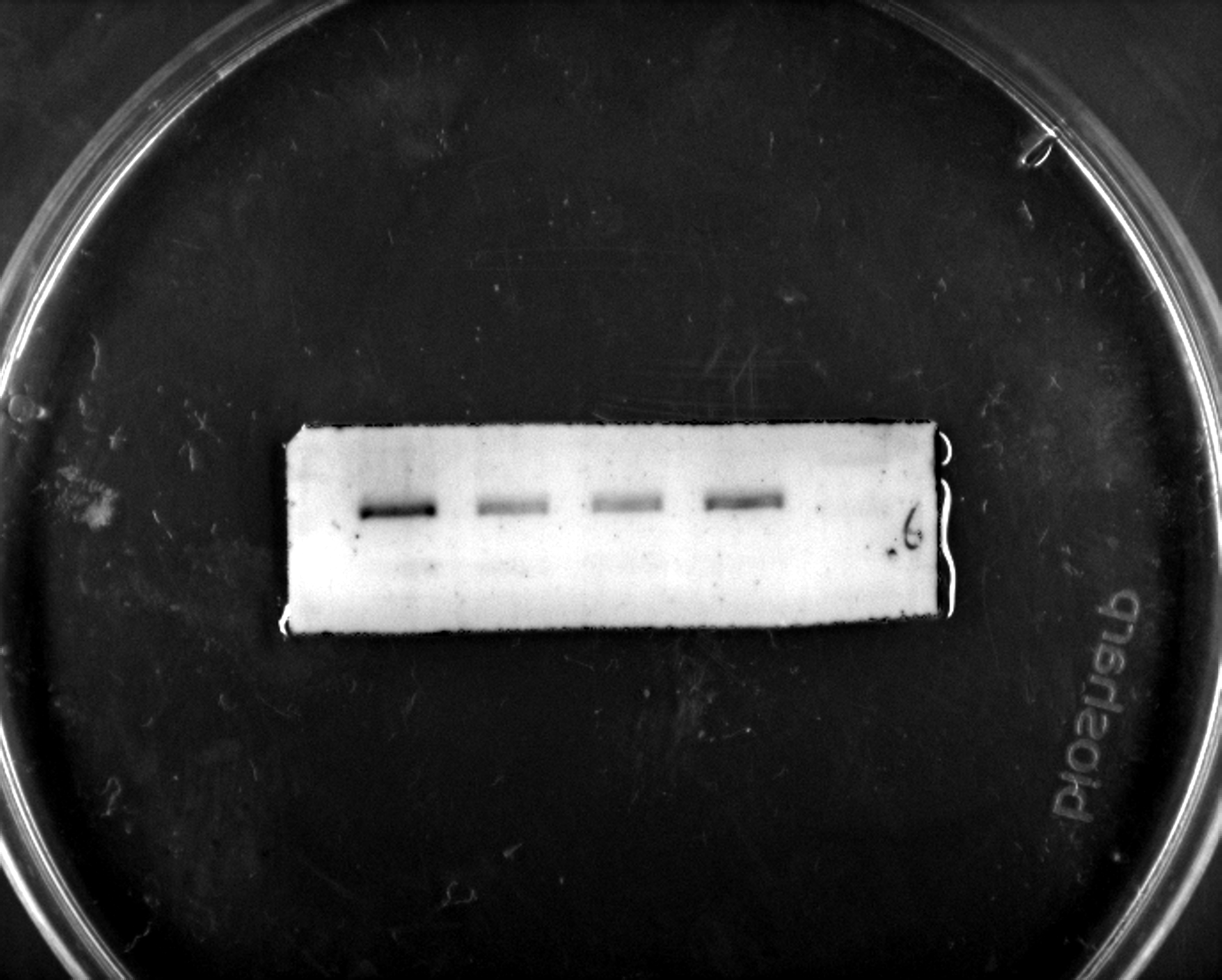

Supplement: Supplemental Information 6 [file peerj-13-19276-s006.zip › C I-R AAV9-CON AAV9-EB1 group western blot-membranal Cx43/2-Cx43-M.Tif]

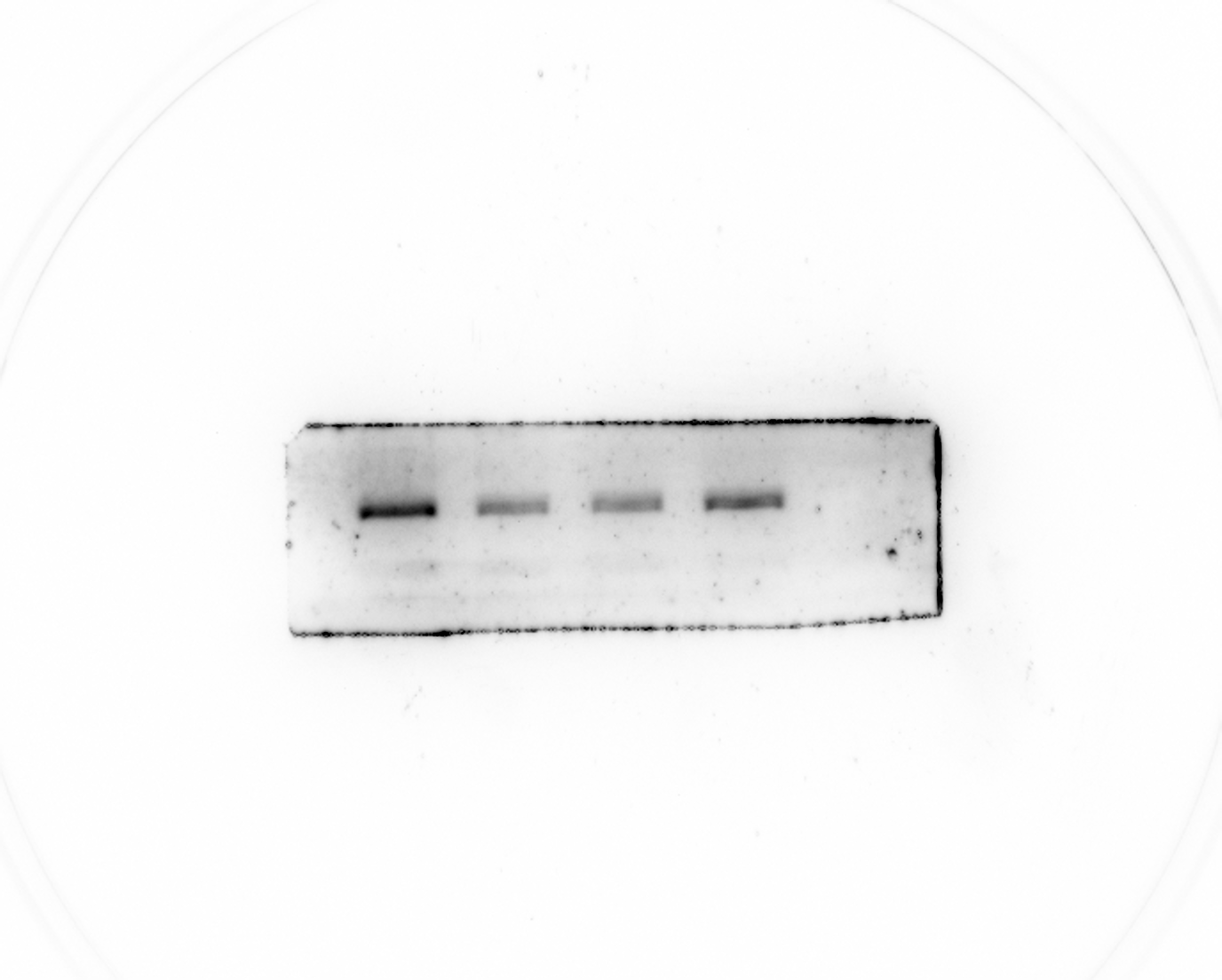

Supplement: Supplemental Information 6 [file peerj-13-19276-s006.zip › C I-R AAV9-CON AAV9-EB1 group western blot-membranal Cx43/2-Cx43.Tif]

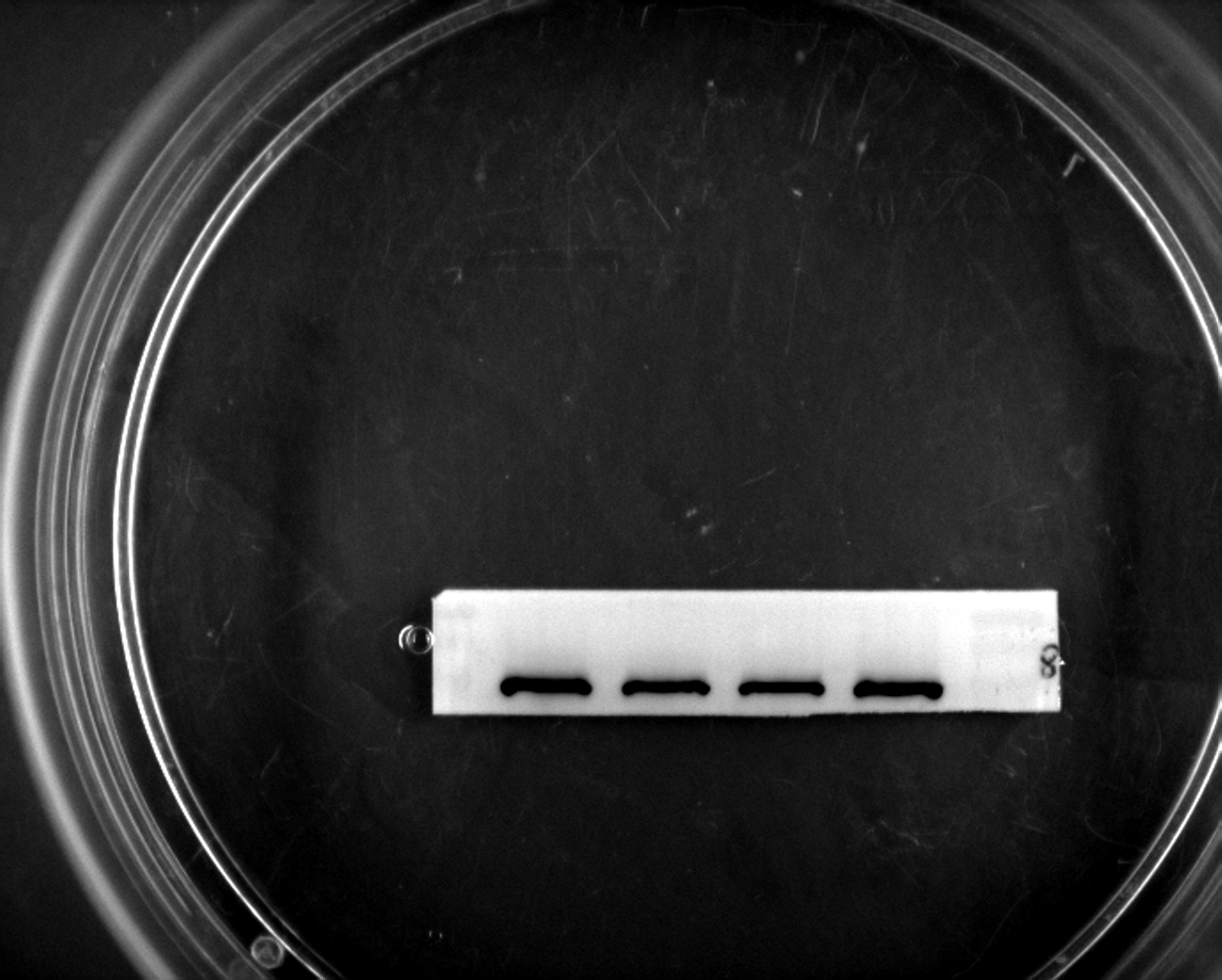

Supplement: Supplemental Information 6 [file peerj-13-19276-s006.zip › C I-R AAV9-CON AAV9-EB1 group western blot-membranal Cx43/3-ATPase-M.Tif]

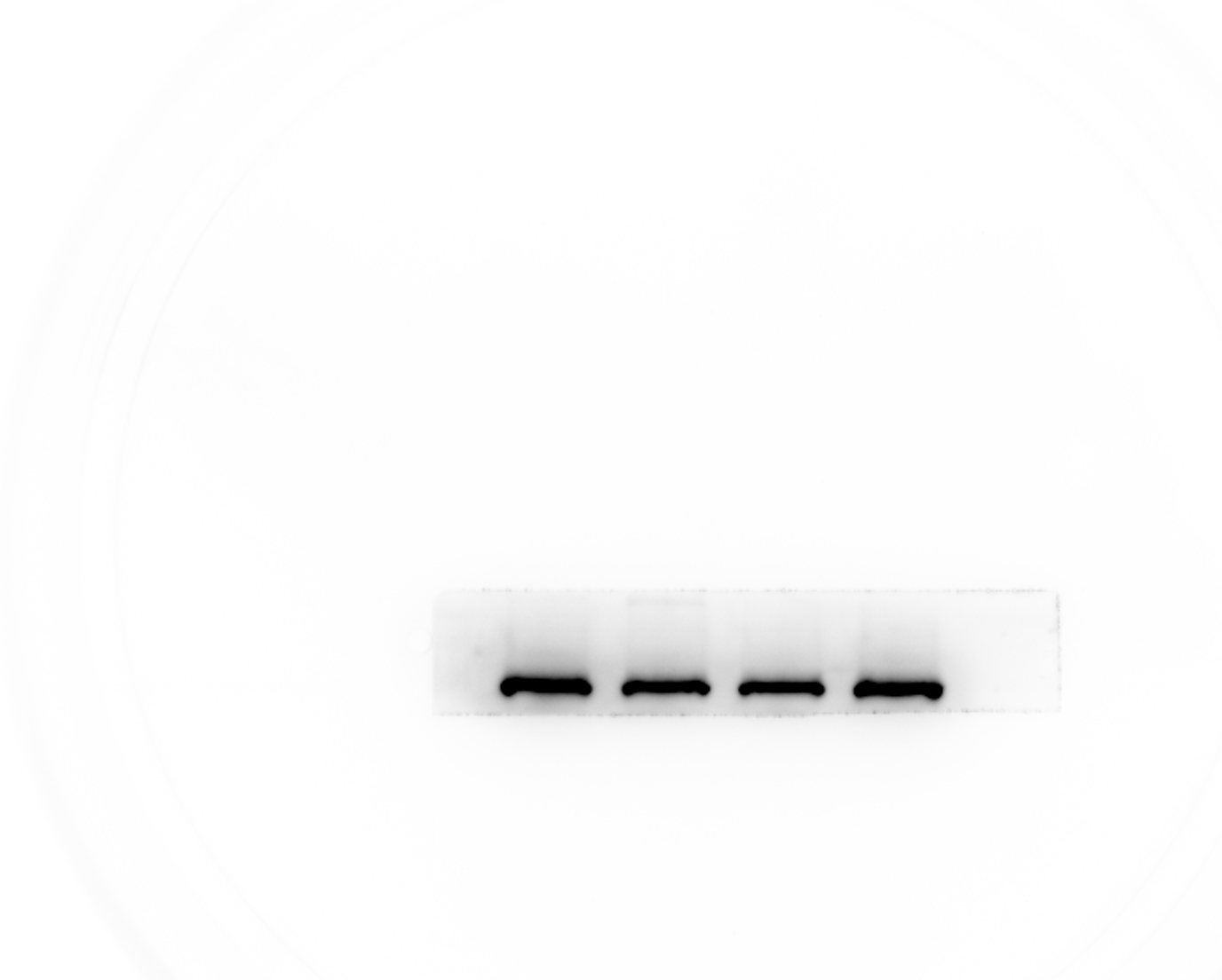

Supplement: Supplemental Information 6 [file peerj-13-19276-s006.zip › C I-R AAV9-CON AAV9-EB1 group western blot-membranal Cx43/3-ATPase.Tif]

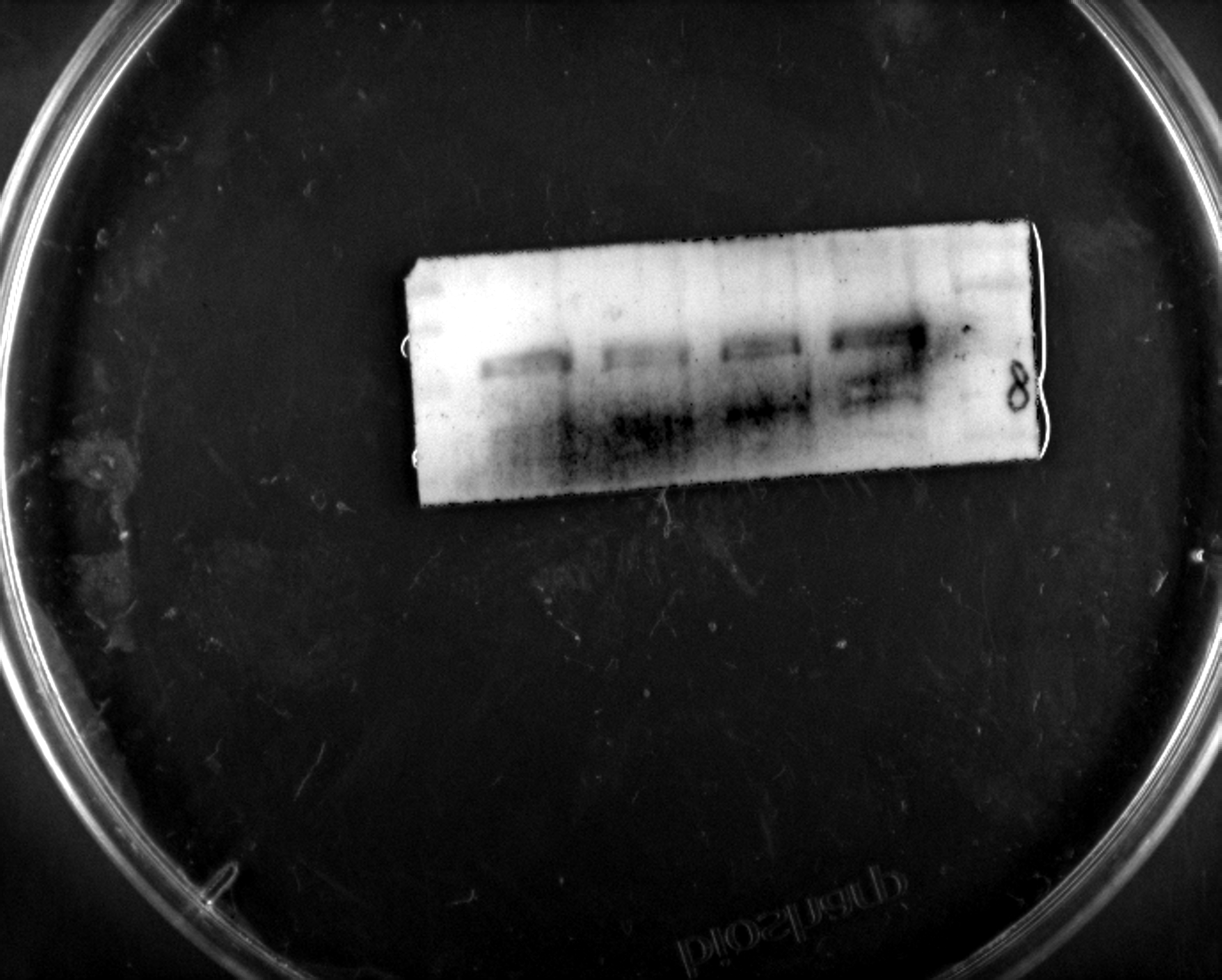

Supplement: Supplemental Information 6 [file peerj-13-19276-s006.zip › C I-R AAV9-CON AAV9-EB1 group western blot-membranal Cx43/3-Cx43-M.Tif]

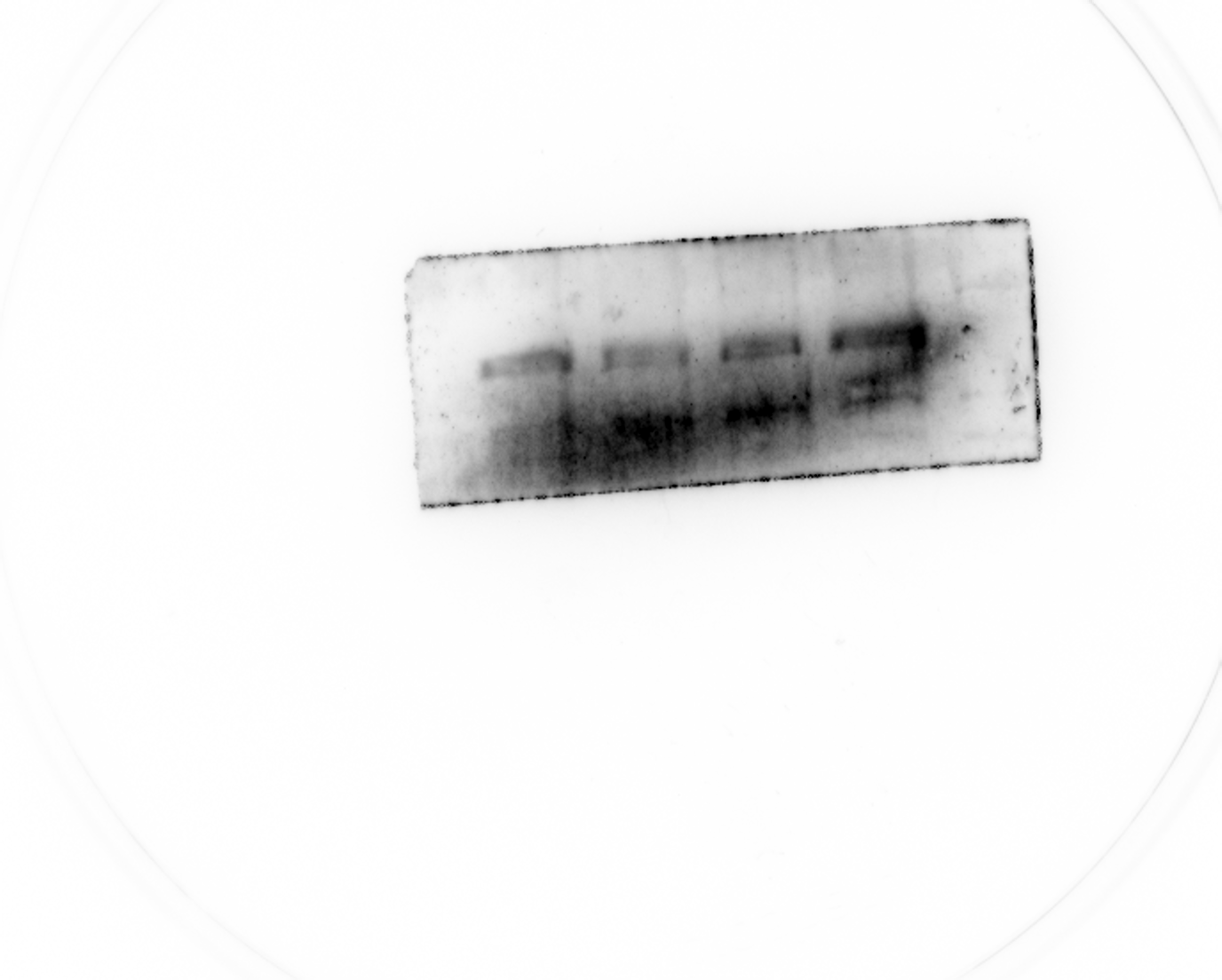

Supplement: Supplemental Information 6 [file peerj-13-19276-s006.zip › C I-R AAV9-CON AAV9-EB1 group western blot-membranal Cx43/3-Cx43.Tif]

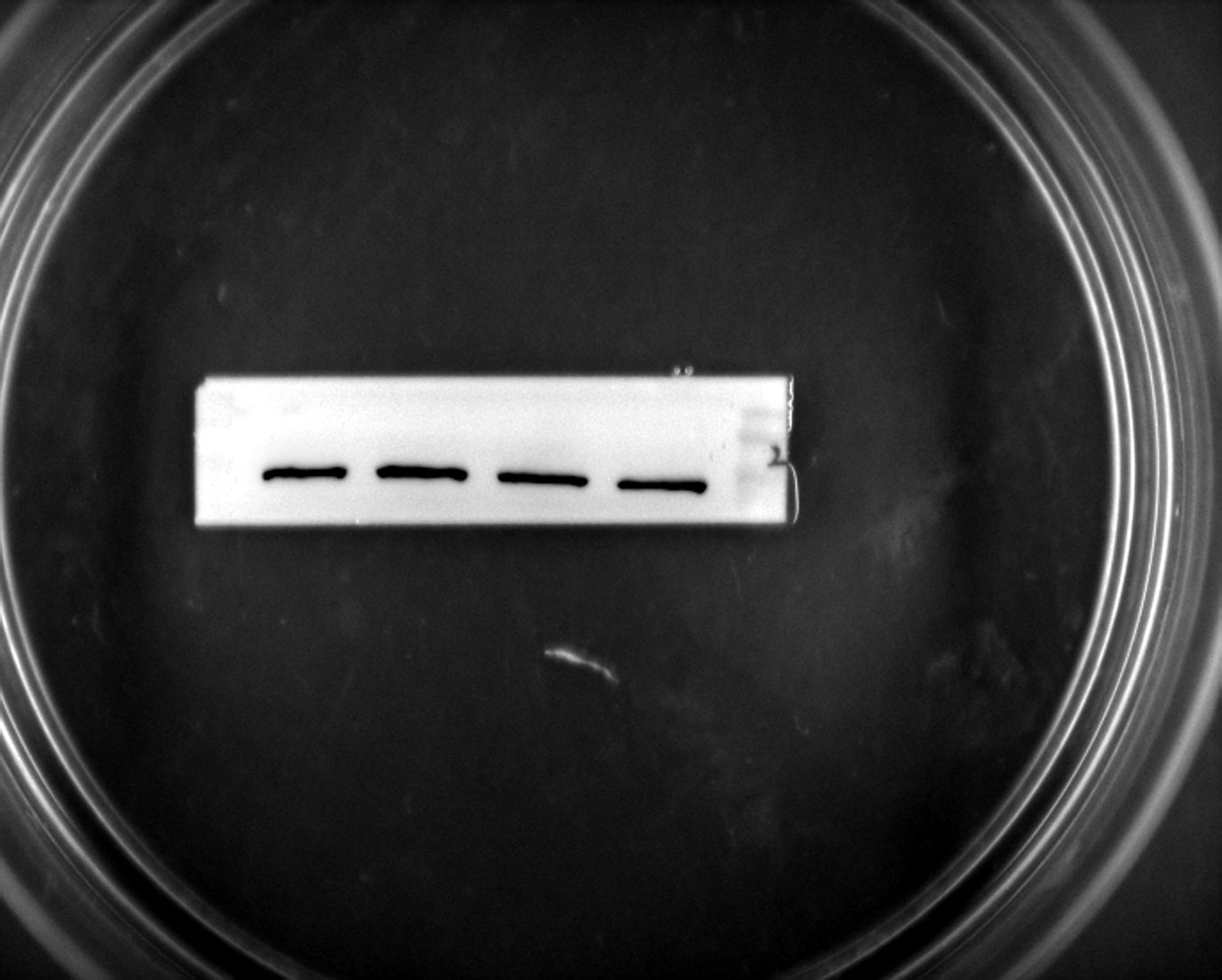

Supplement: Supplemental Information 6 [file peerj-13-19276-s006.zip › C I-R AAV9-CON AAV9-EB1 group western blot-membranal Cx43/4-ATPase-M.Tif]

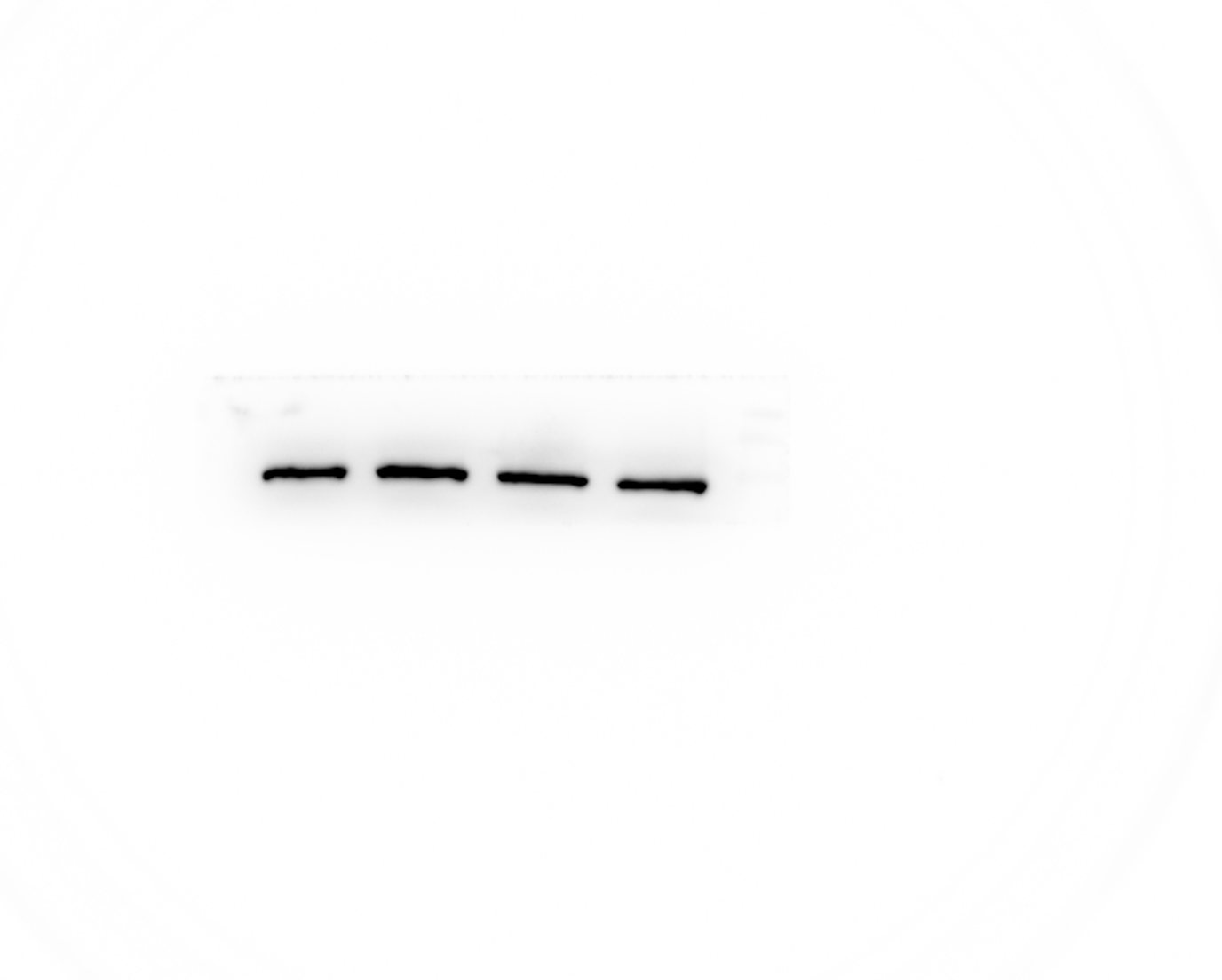

Supplement: Supplemental Information 6 [file peerj-13-19276-s006.zip › C I-R AAV9-CON AAV9-EB1 group western blot-membranal Cx43/4-ATPase.Tif]

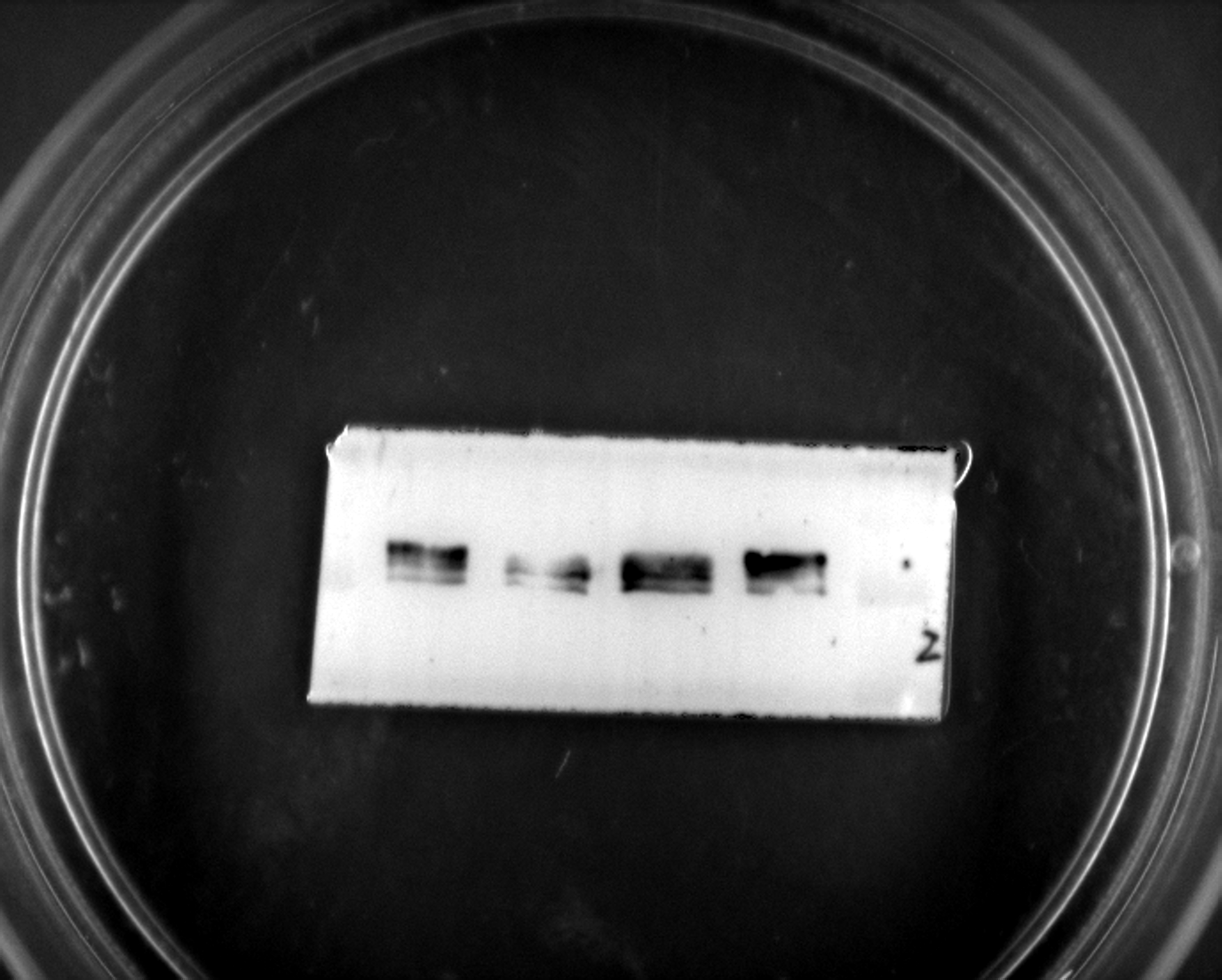

Supplement: Supplemental Information 6 [file peerj-13-19276-s006.zip › C I-R AAV9-CON AAV9-EB1 group western blot-membranal Cx43/4-Cx43-M.Tif]

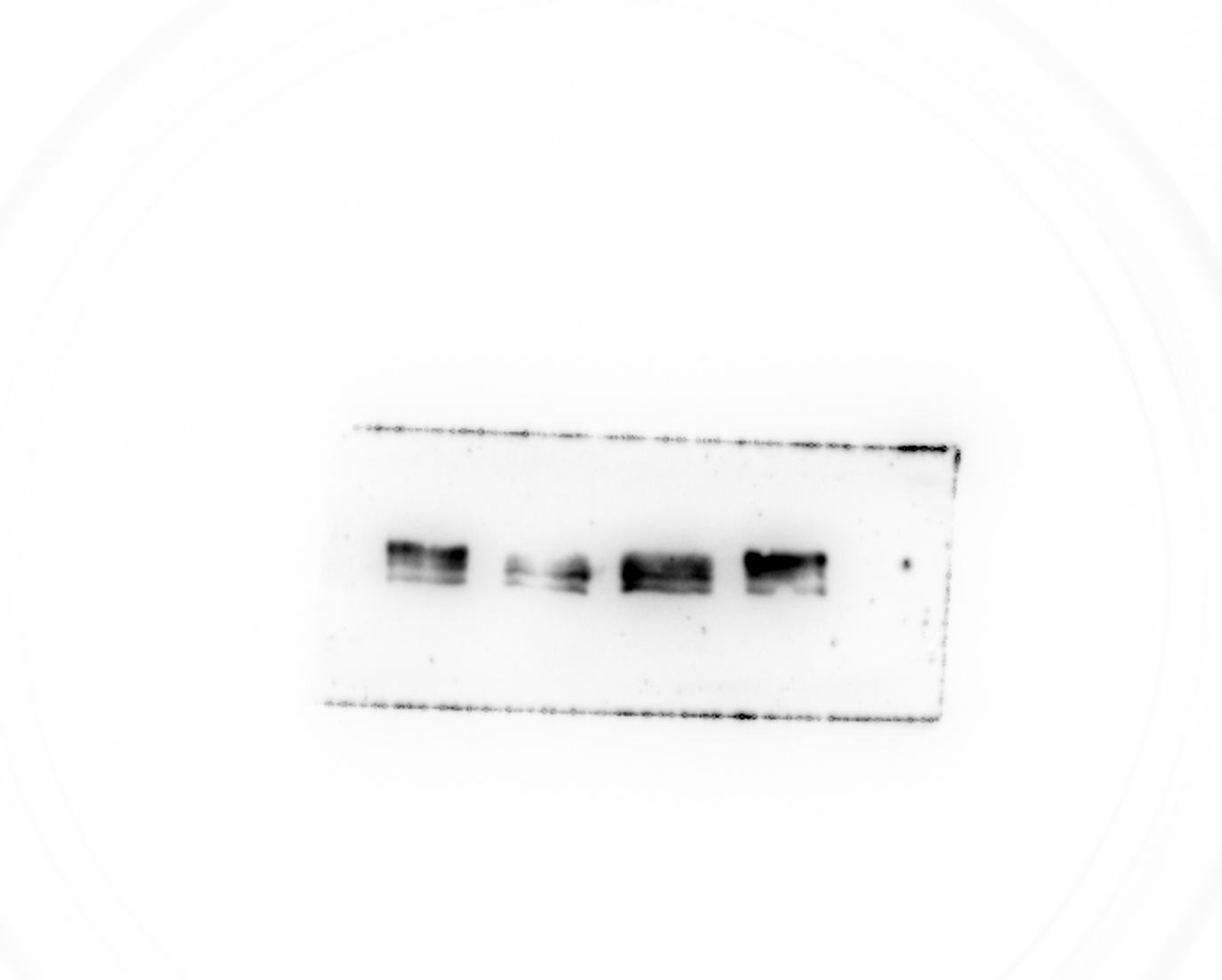

Supplement: Supplemental Information 6 [file peerj-13-19276-s006.zip › C I-R AAV9-CON AAV9-EB1 group western blot-membranal Cx43/4-Cx43.Tif]

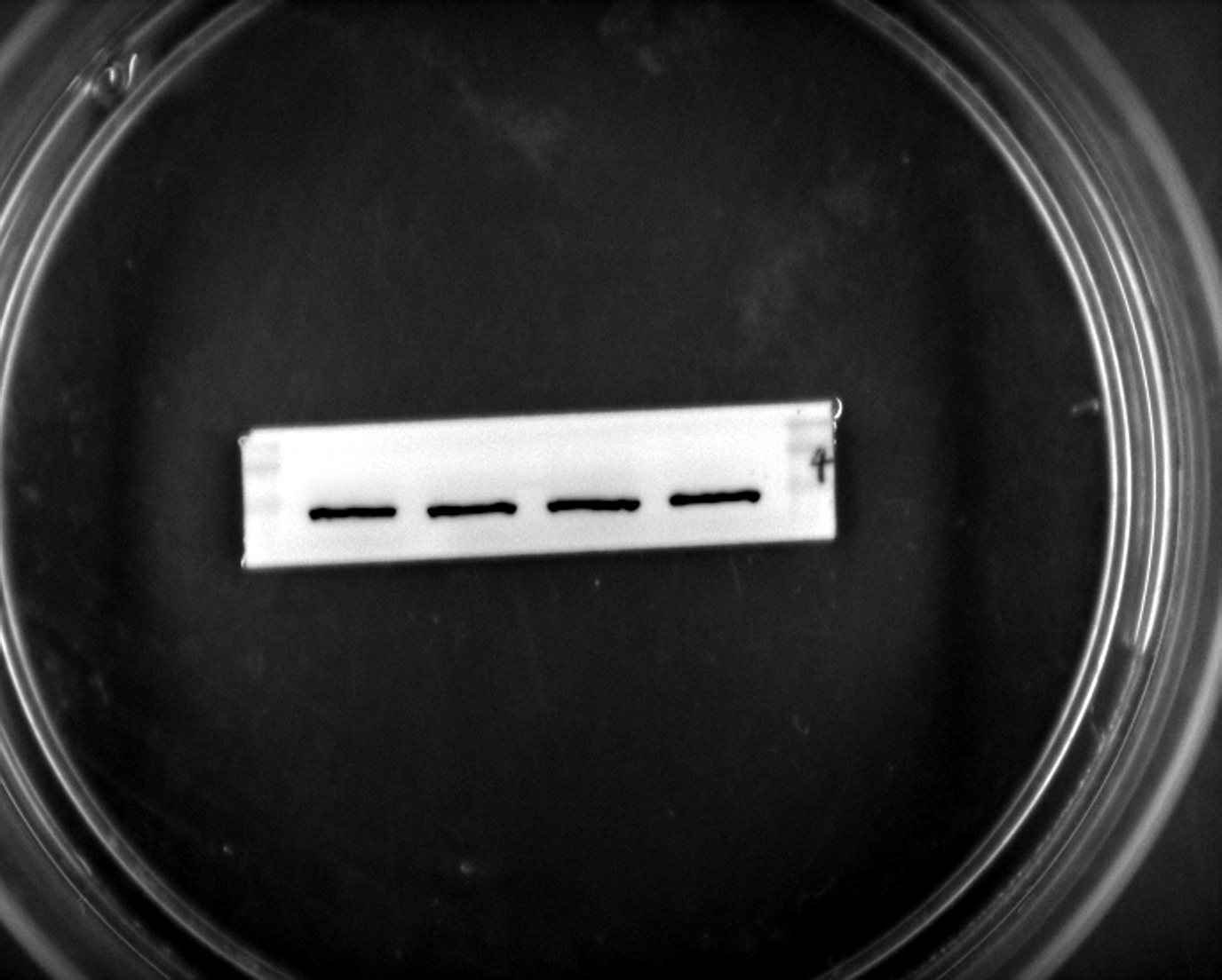

Supplement: Supplemental Information 6 [file peerj-13-19276-s006.zip › C I-R AAV9-CON AAV9-EB1 group western blot-membranal Cx43/5-ATPase-M.Tif]

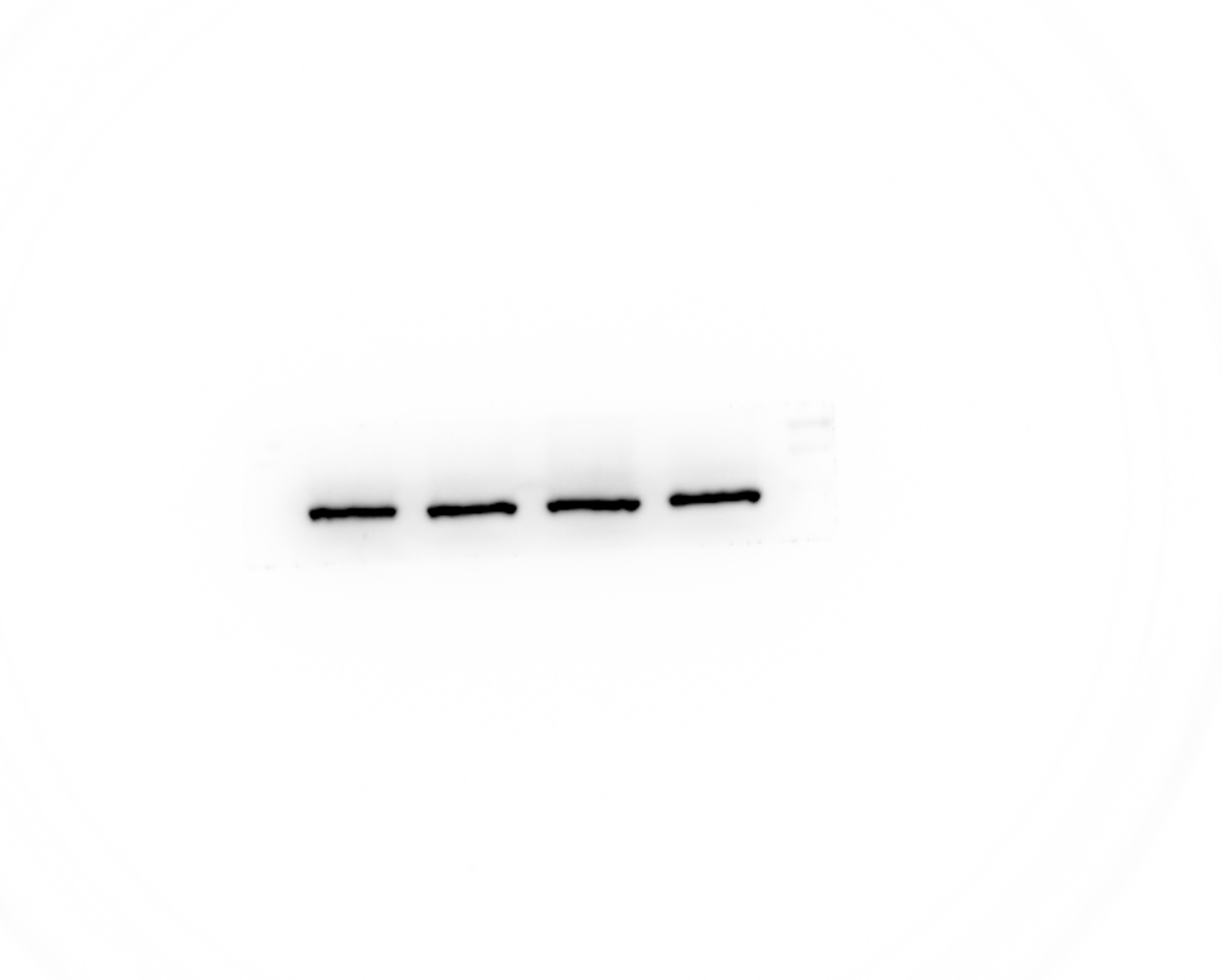

Supplement: Supplemental Information 6 [file peerj-13-19276-s006.zip › C I-R AAV9-CON AAV9-EB1 group western blot-membranal Cx43/5-ATPase.Tif]

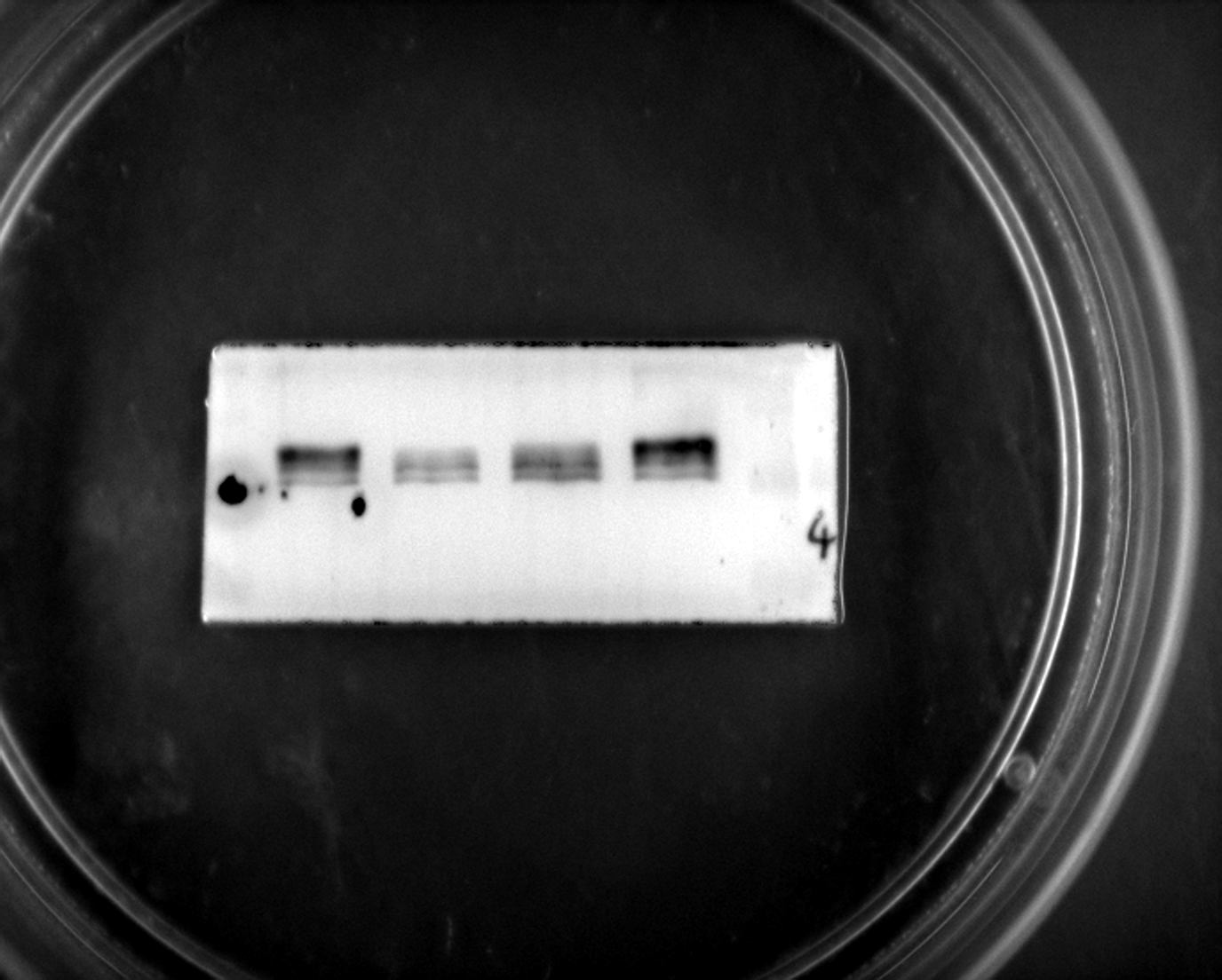

Supplement: Supplemental Information 6 [file peerj-13-19276-s006.zip › C I-R AAV9-CON AAV9-EB1 group western blot-membranal Cx43/5-Cx43-M.Tif]

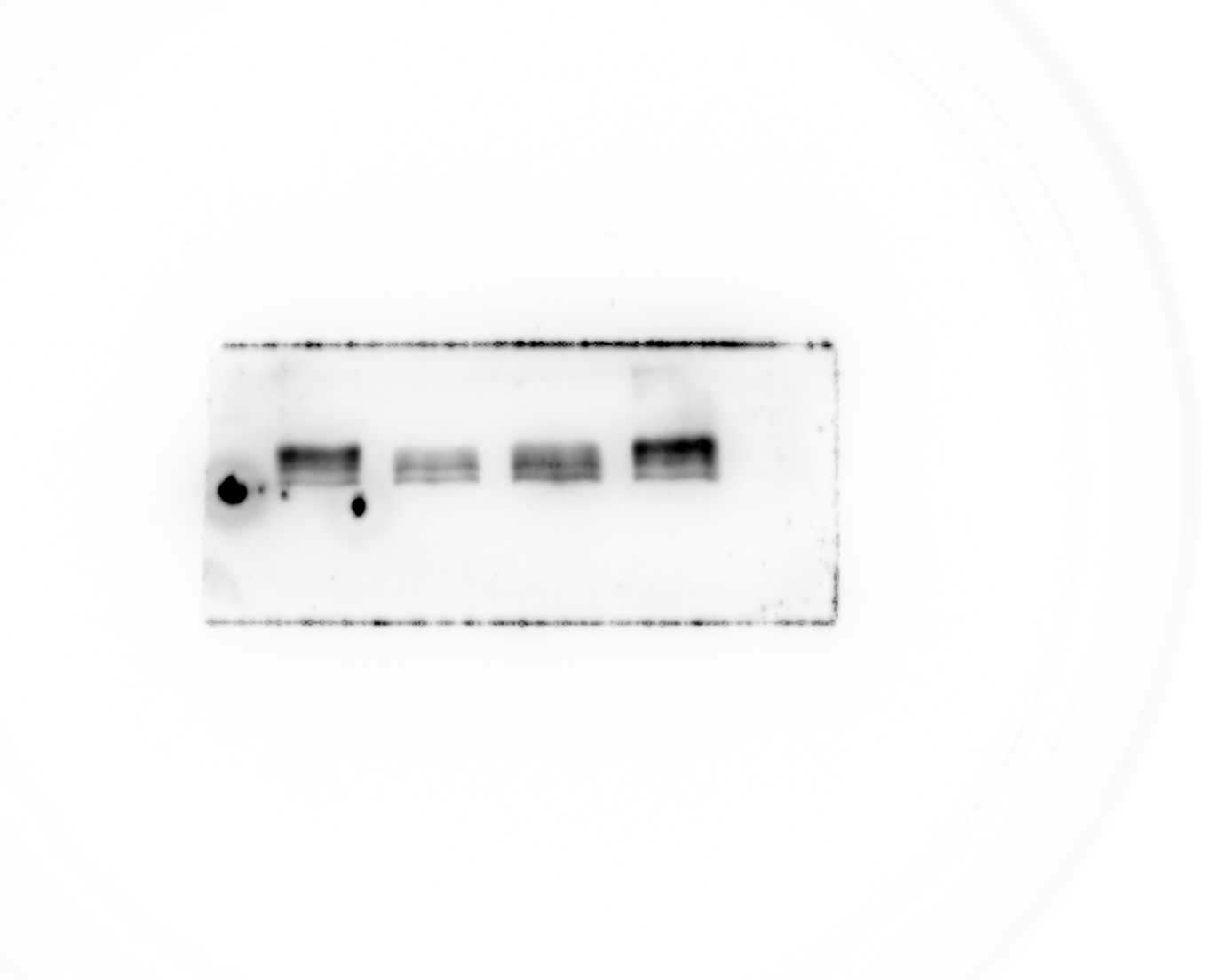

Supplement: Supplemental Information 6 [file peerj-13-19276-s006.zip › C I-R AAV9-CON AAV9-EB1 group western blot-membranal Cx43/5-Cx43.Tif]

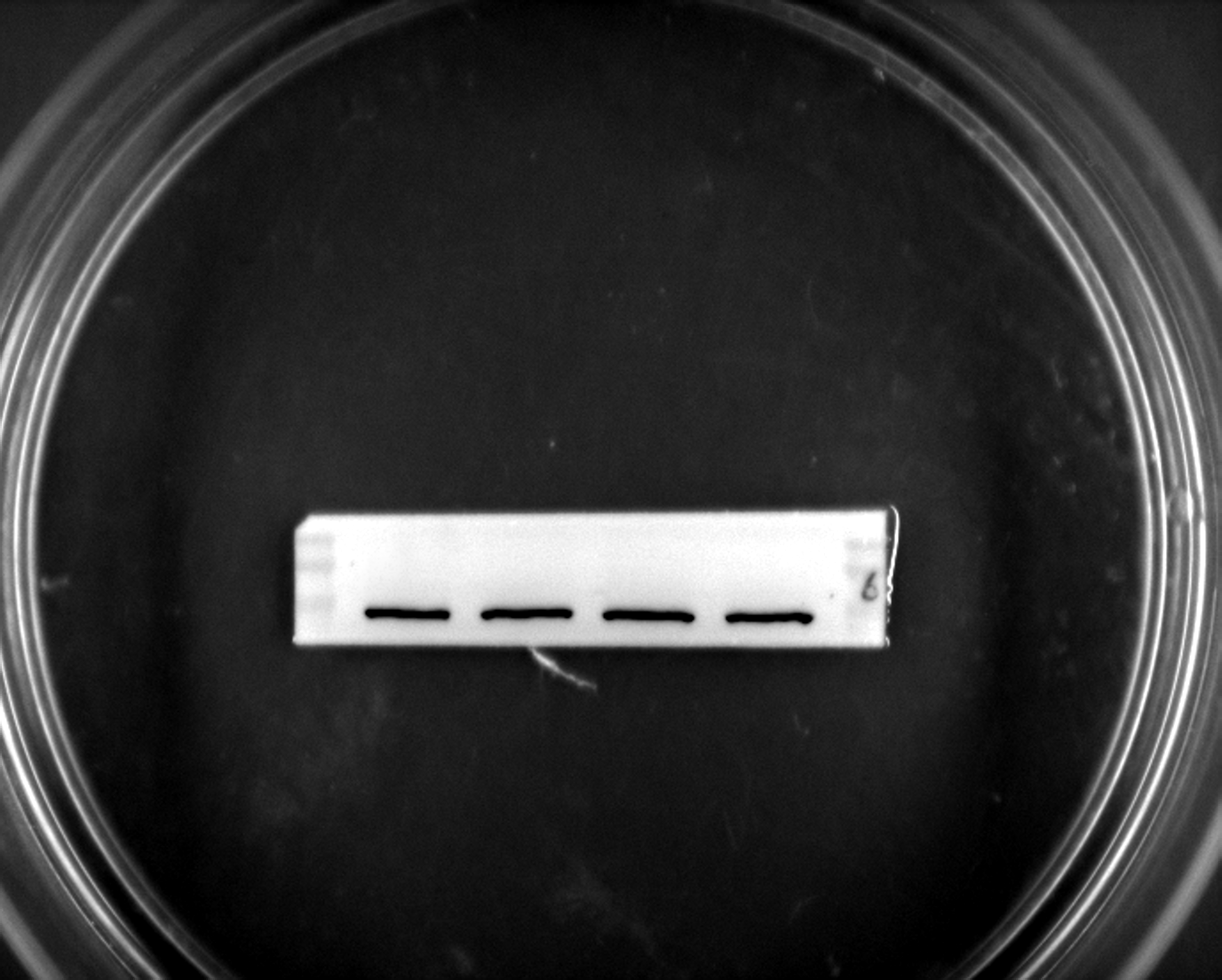

Supplement: Supplemental Information 6 [file peerj-13-19276-s006.zip › C I-R AAV9-CON AAV9-EB1 group western blot-membranal Cx43/6-ATPase-M.Tif]

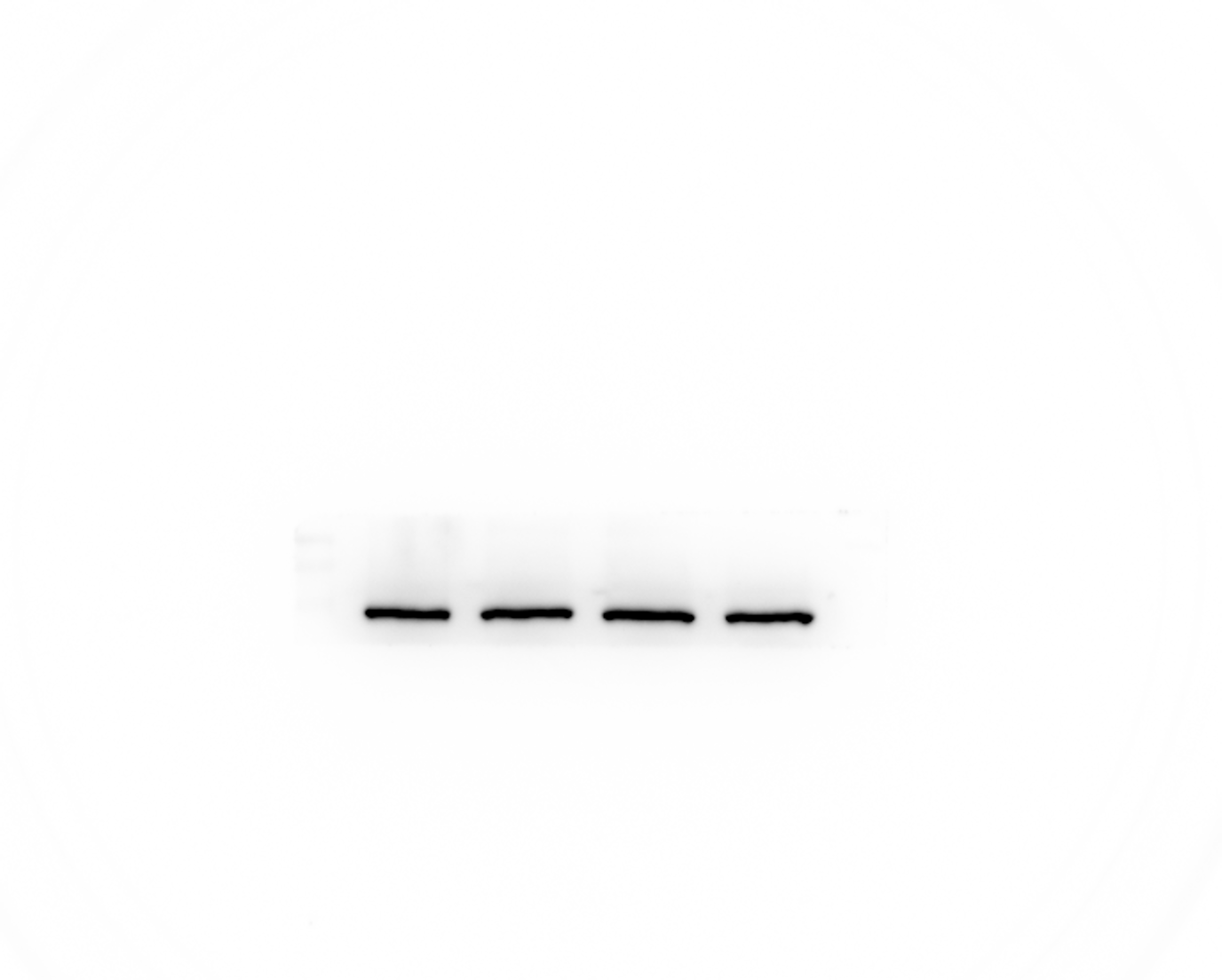

Supplement: Supplemental Information 6 [file peerj-13-19276-s006.zip › C I-R AAV9-CON AAV9-EB1 group western blot-membranal Cx43/6-ATPase.Tif]

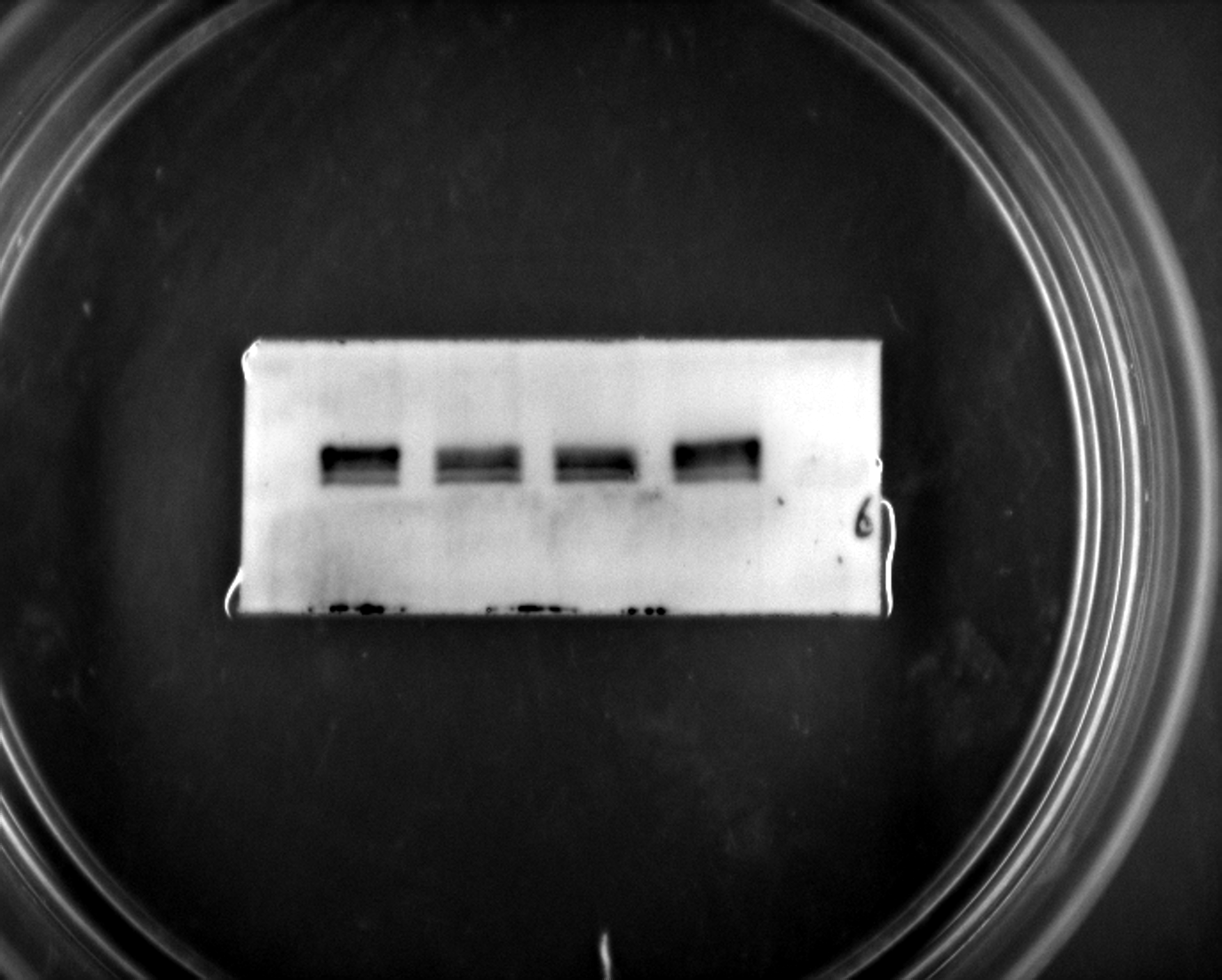

Supplement: Supplemental Information 6 [file peerj-13-19276-s006.zip › C I-R AAV9-CON AAV9-EB1 group western blot-membranal Cx43/6-Cx43-M.Tif]

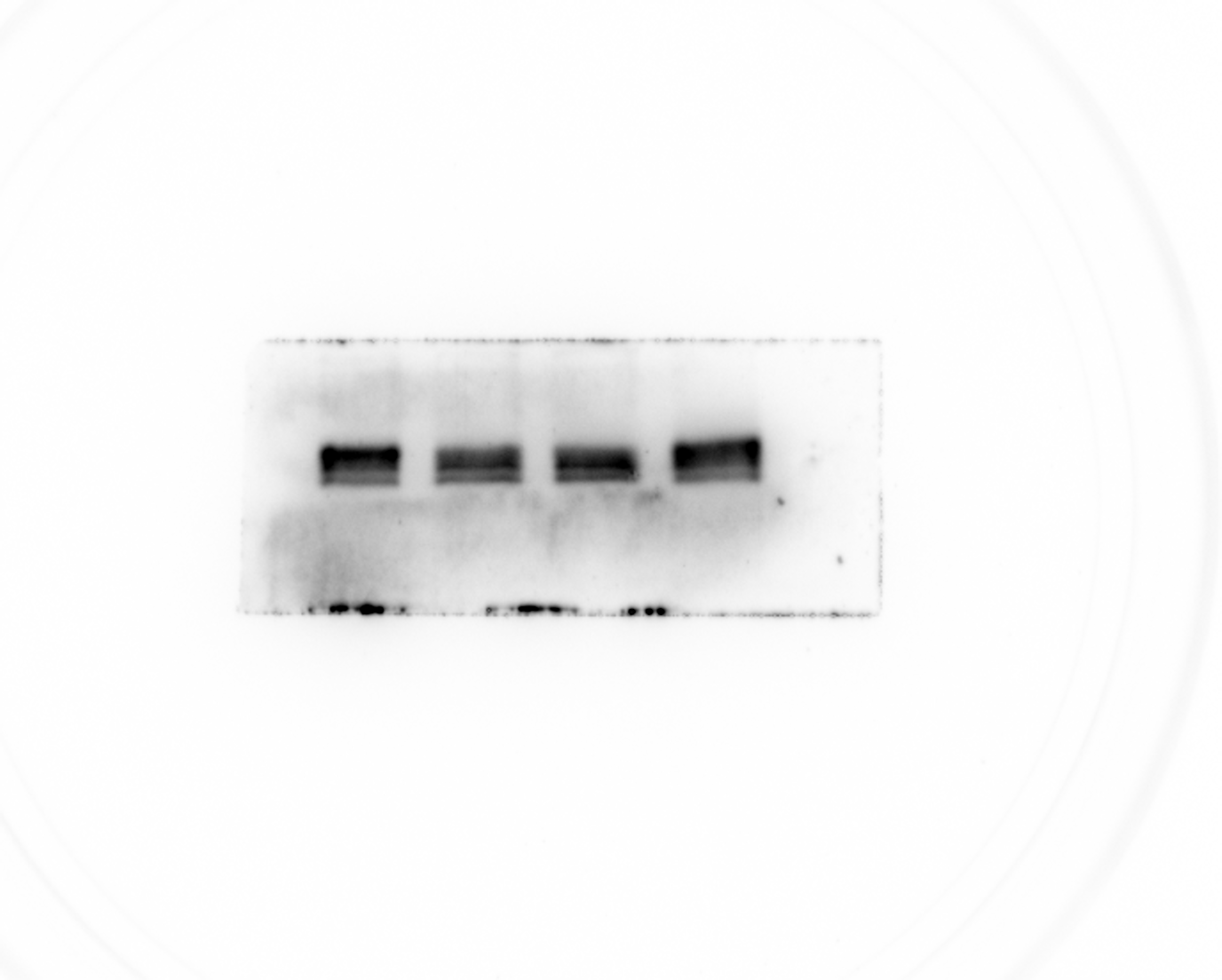

Supplement: Supplemental Information 6 [file peerj-13-19276-s006.zip › C I-R AAV9-CON AAV9-EB1 group western blot-membranal Cx43/6-Cx43.Tif]

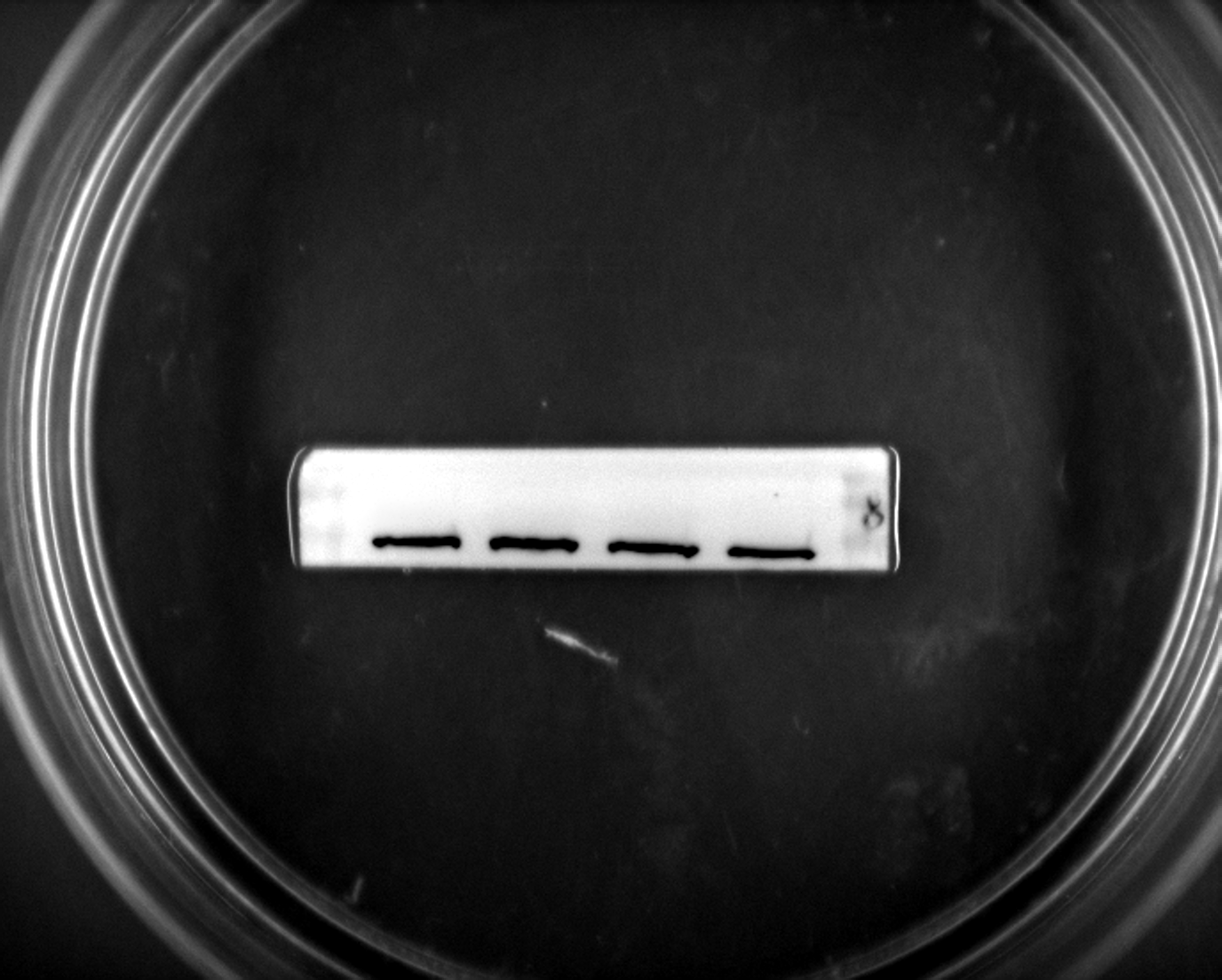

Supplement: Supplemental Information 6 [file peerj-13-19276-s006.zip › C I-R AAV9-CON AAV9-EB1 group western blot-membranal Cx43/7-ATPase-M.Tif]

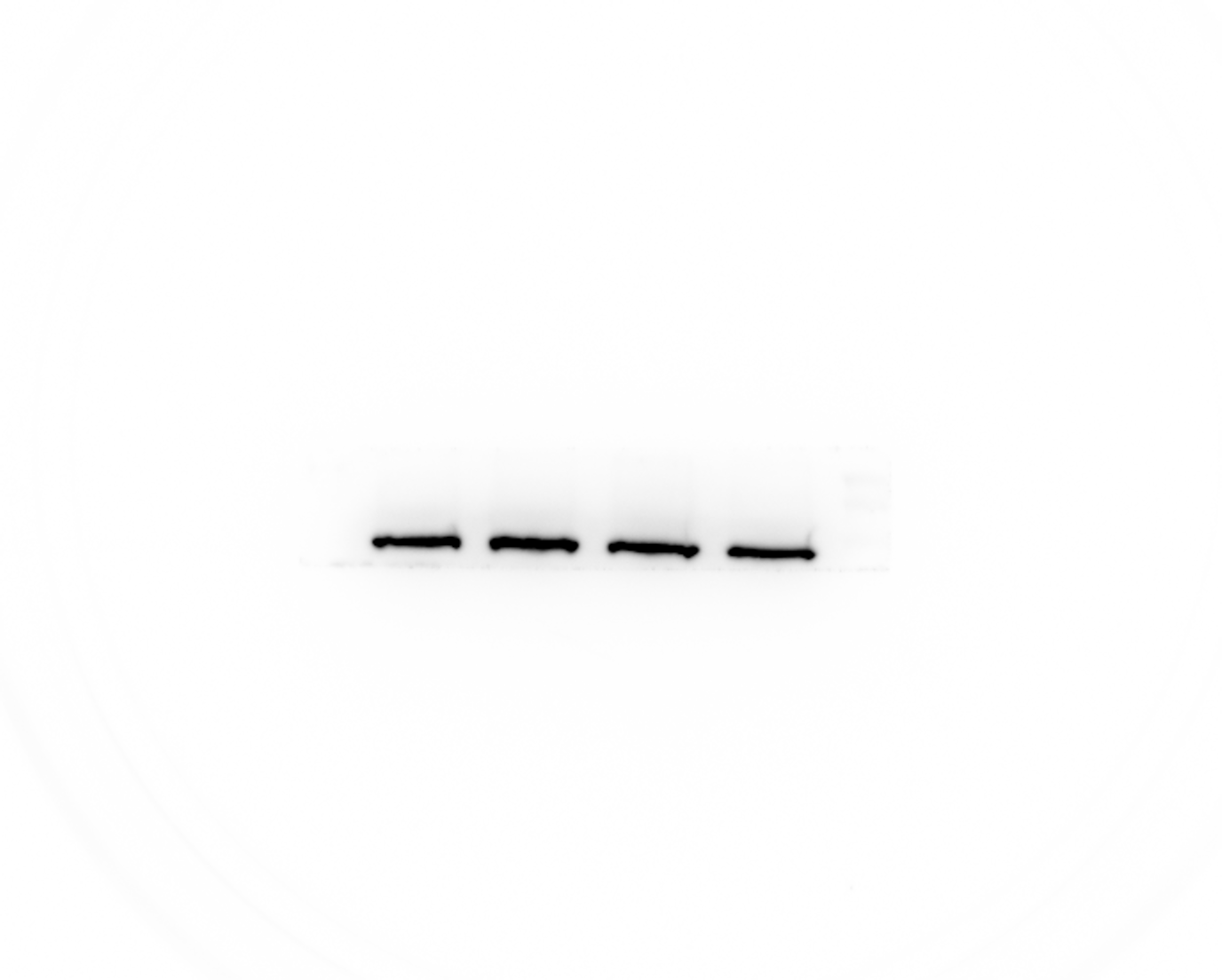

Supplement: Supplemental Information 6 [file peerj-13-19276-s006.zip › C I-R AAV9-CON AAV9-EB1 group western blot-membranal Cx43/7-ATPase.Tif]

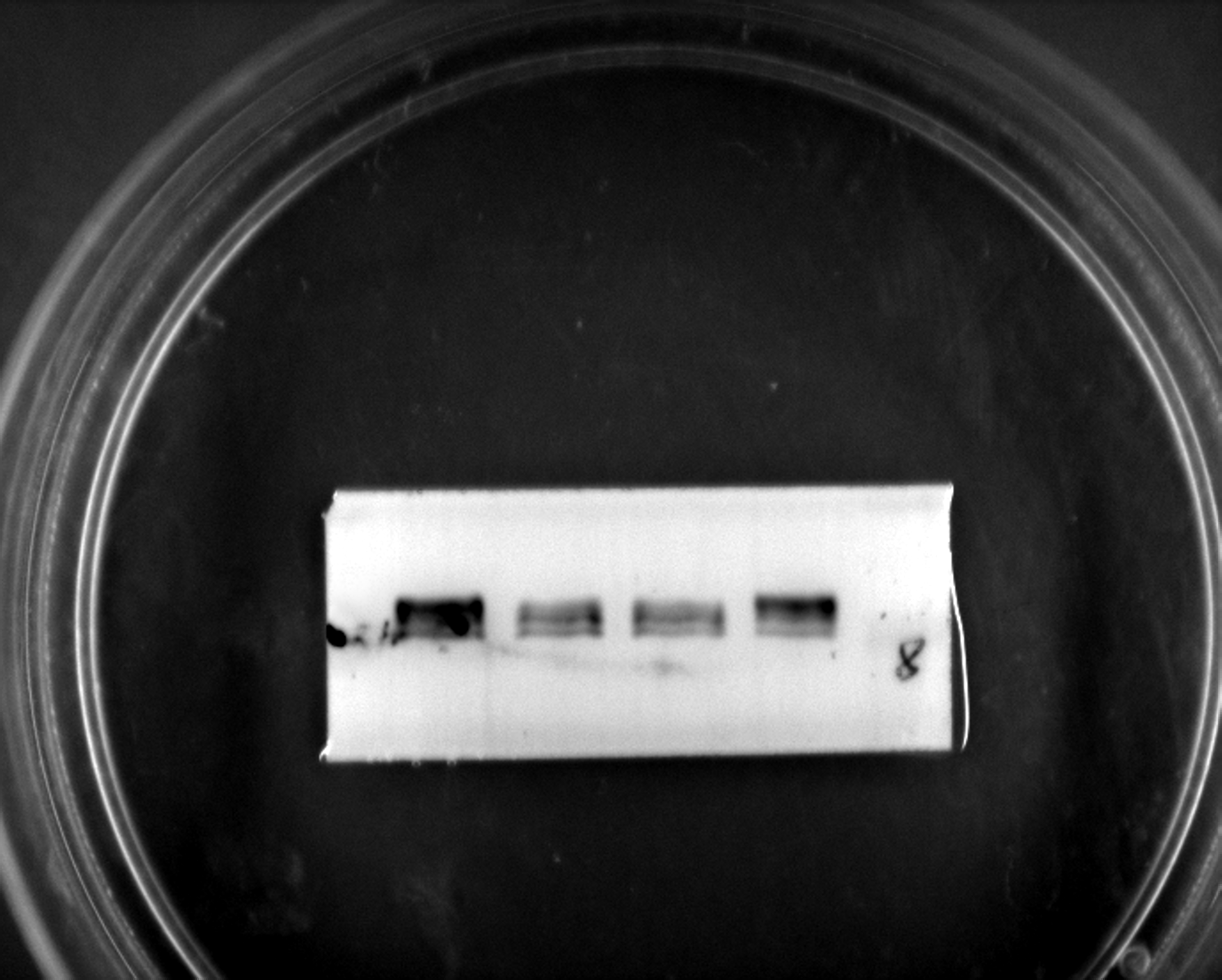

Supplement: Supplemental Information 6 [file peerj-13-19276-s006.zip › C I-R AAV9-CON AAV9-EB1 group western blot-membranal Cx43/7-Cx43-M.Tif]

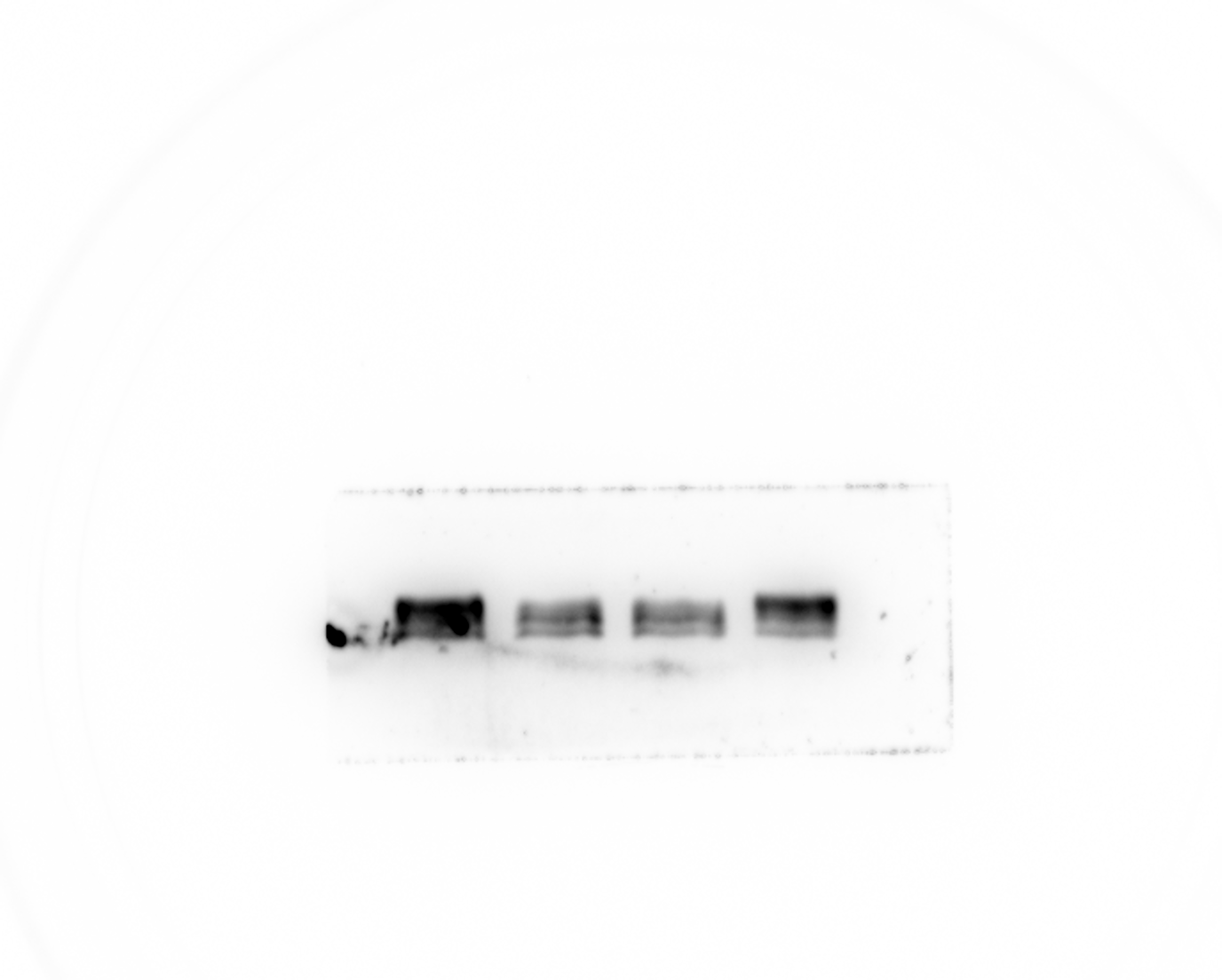

Supplement: Supplemental Information 6 [file peerj-13-19276-s006.zip › C I-R AAV9-CON AAV9-EB1 group western blot-membranal Cx43/7-Cx43.Tif]

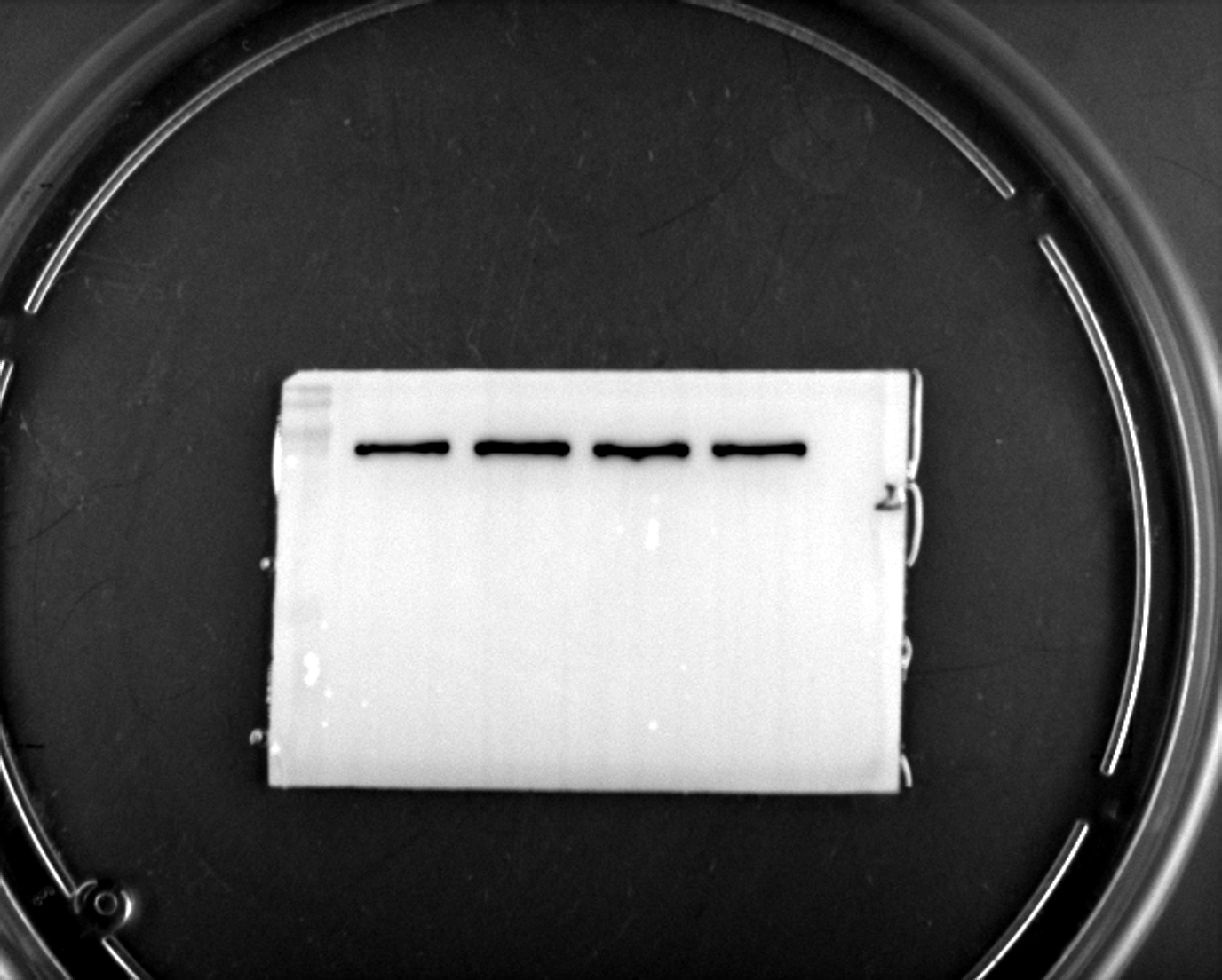

Supplement: Supplemental Information 6 [file peerj-13-19276-s006.zip › C I-R AAV9-CON AAV9-EB1 group western blot-membranal Cx43/8-ATPase-M.Tif]

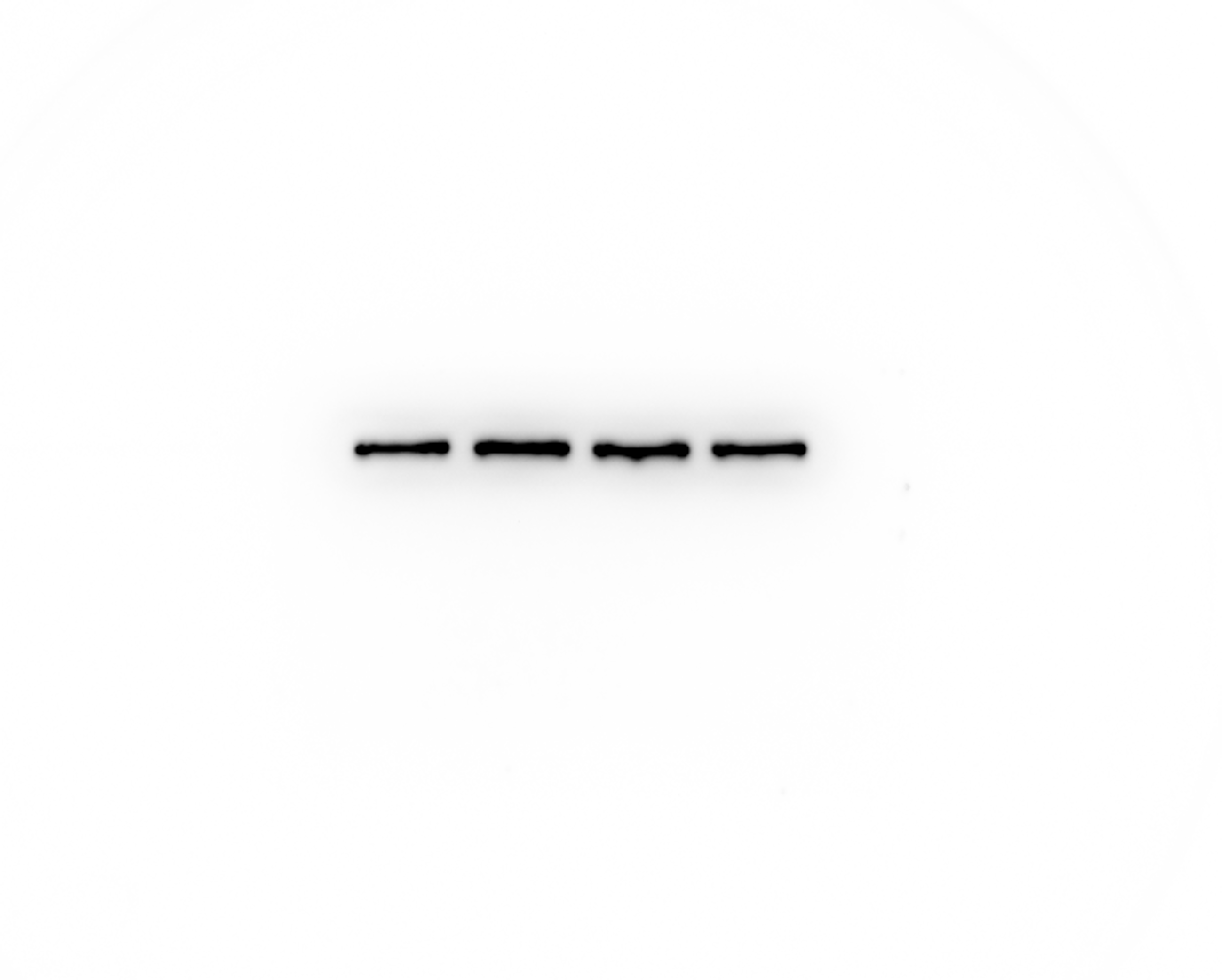

Supplement: Supplemental Information 6 [file peerj-13-19276-s006.zip › C I-R AAV9-CON AAV9-EB1 group western blot-membranal Cx43/8-ATPase.Tif]

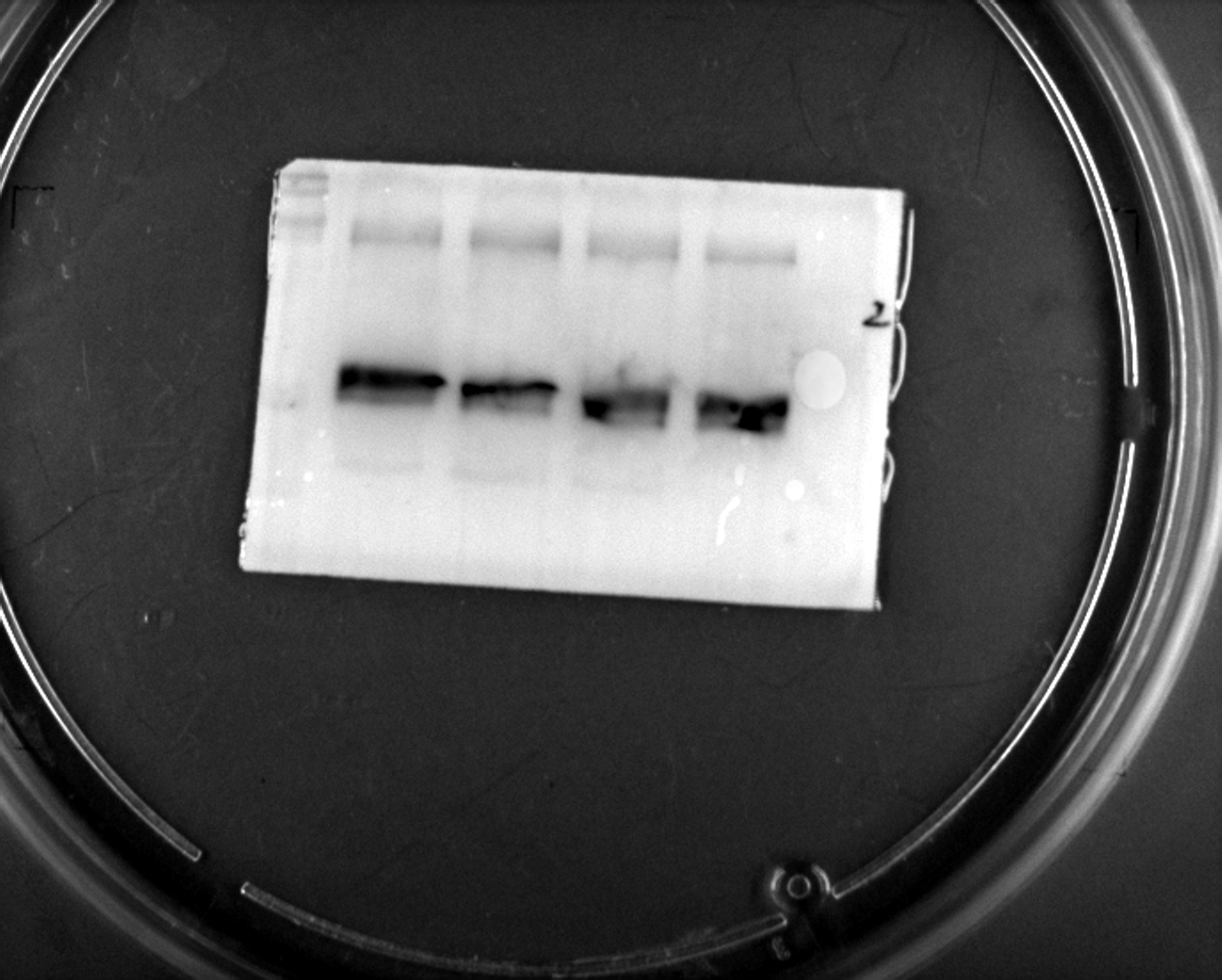

Supplement: Supplemental Information 6 [file peerj-13-19276-s006.zip › C I-R AAV9-CON AAV9-EB1 group western blot-membranal Cx43/8-Cx43-M.Tif]

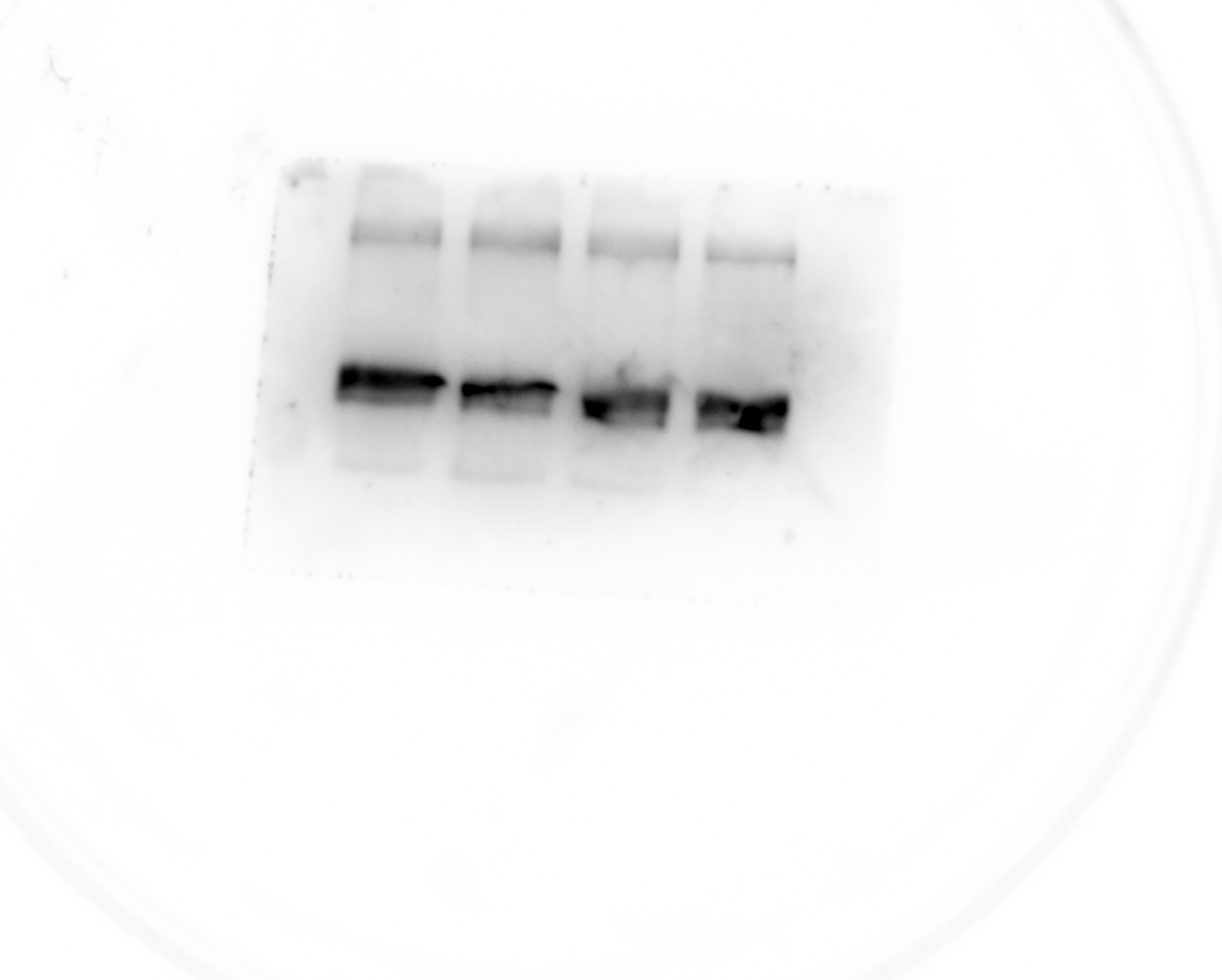

Supplement: Supplemental Information 6 [file peerj-13-19276-s006.zip › C I-R AAV9-CON AAV9-EB1 group western blot-membranal Cx43/8-Cx43.Tif]

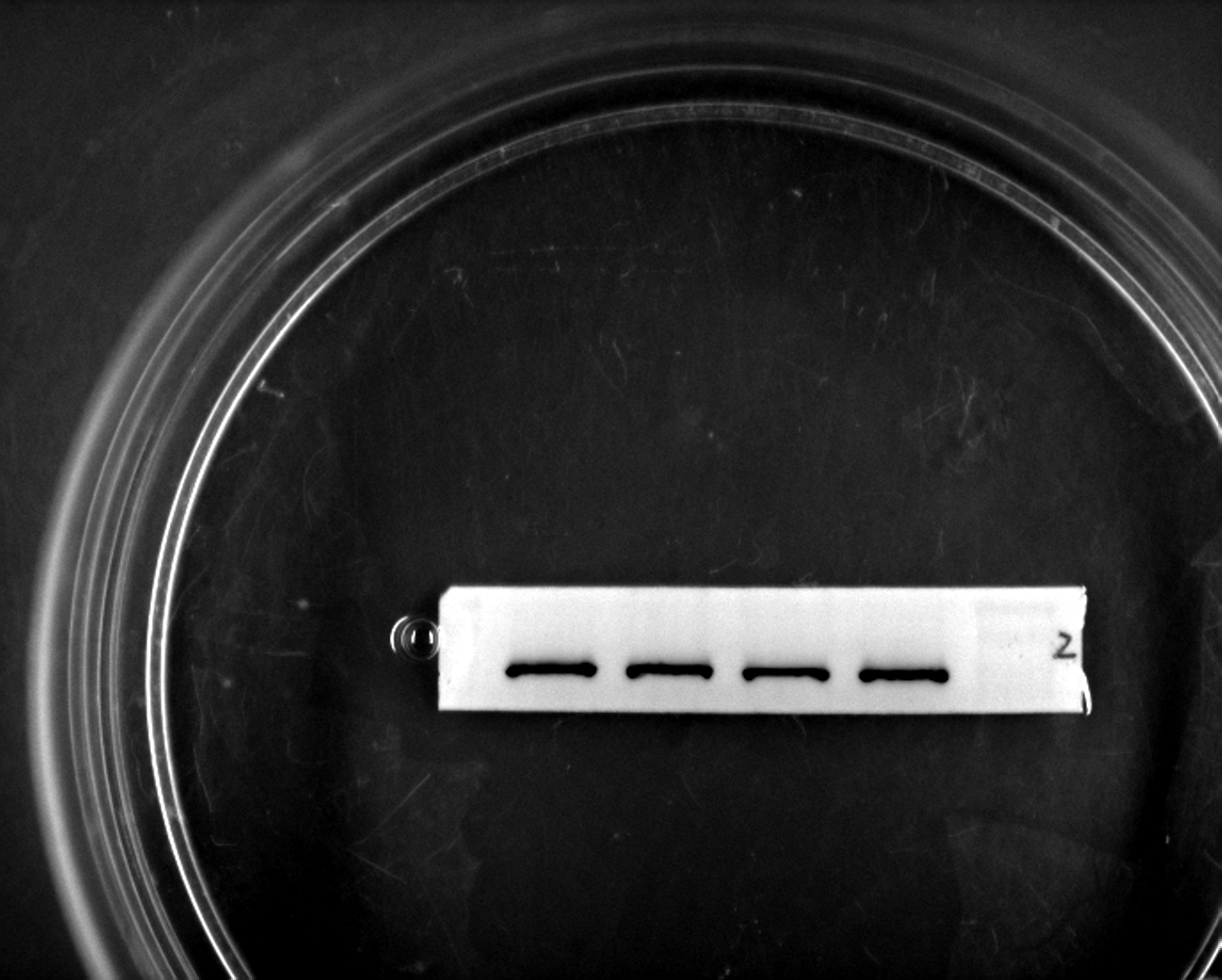

Supplement: Supplemental Information 6 [file peerj-13-19276-s006.zip › C I-R AAV9-CON AAV9-EB1 group western blot-membranal Cx43/9-ATPase-M.Tif]

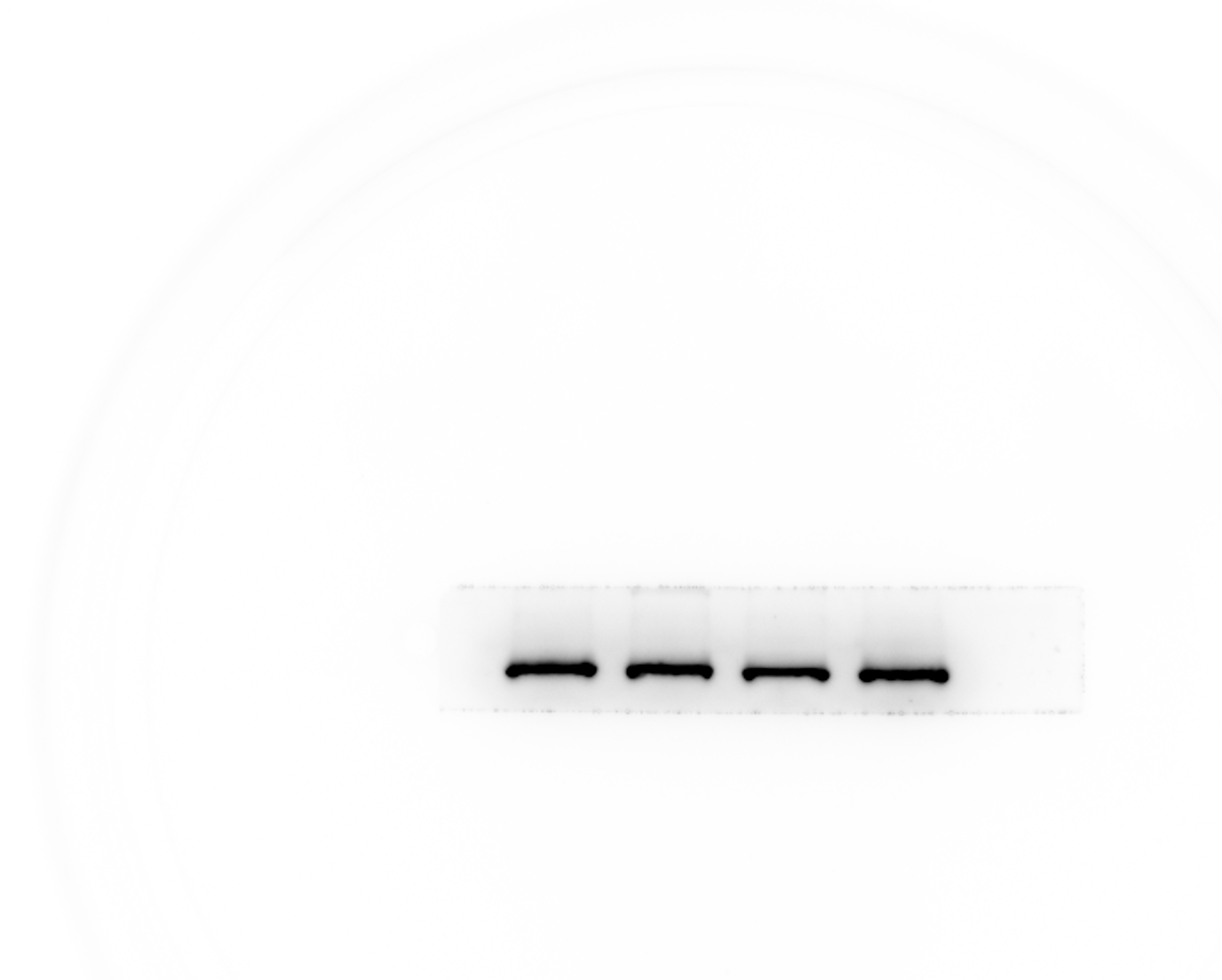

Supplement: Supplemental Information 6 [file peerj-13-19276-s006.zip › C I-R AAV9-CON AAV9-EB1 group western blot-membranal Cx43/9-ATPase.Tif]

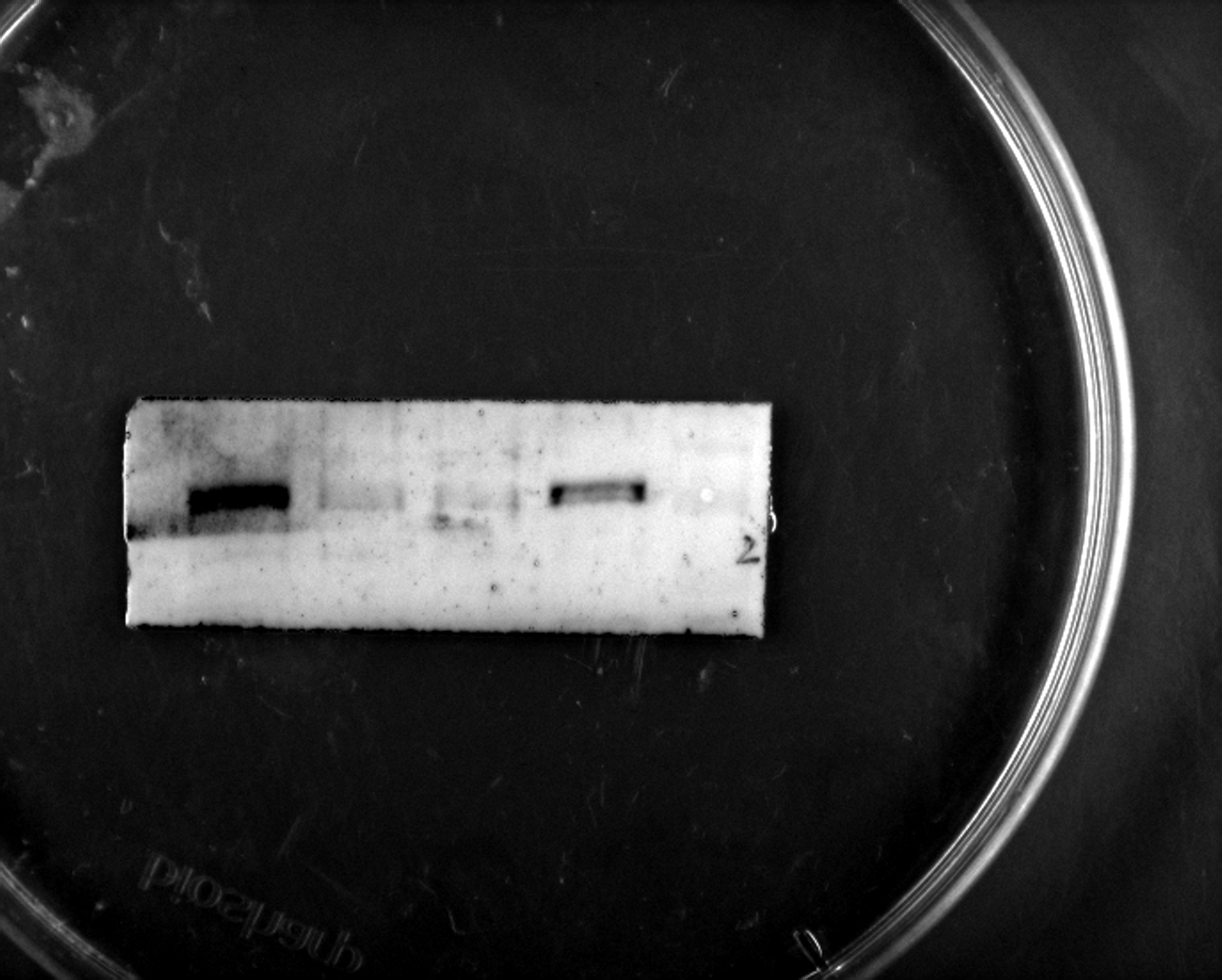

Supplement: Supplemental Information 6 [file peerj-13-19276-s006.zip › C I-R AAV9-CON AAV9-EB1 group western blot-membranal Cx43/9-Cx43-M.Tif]

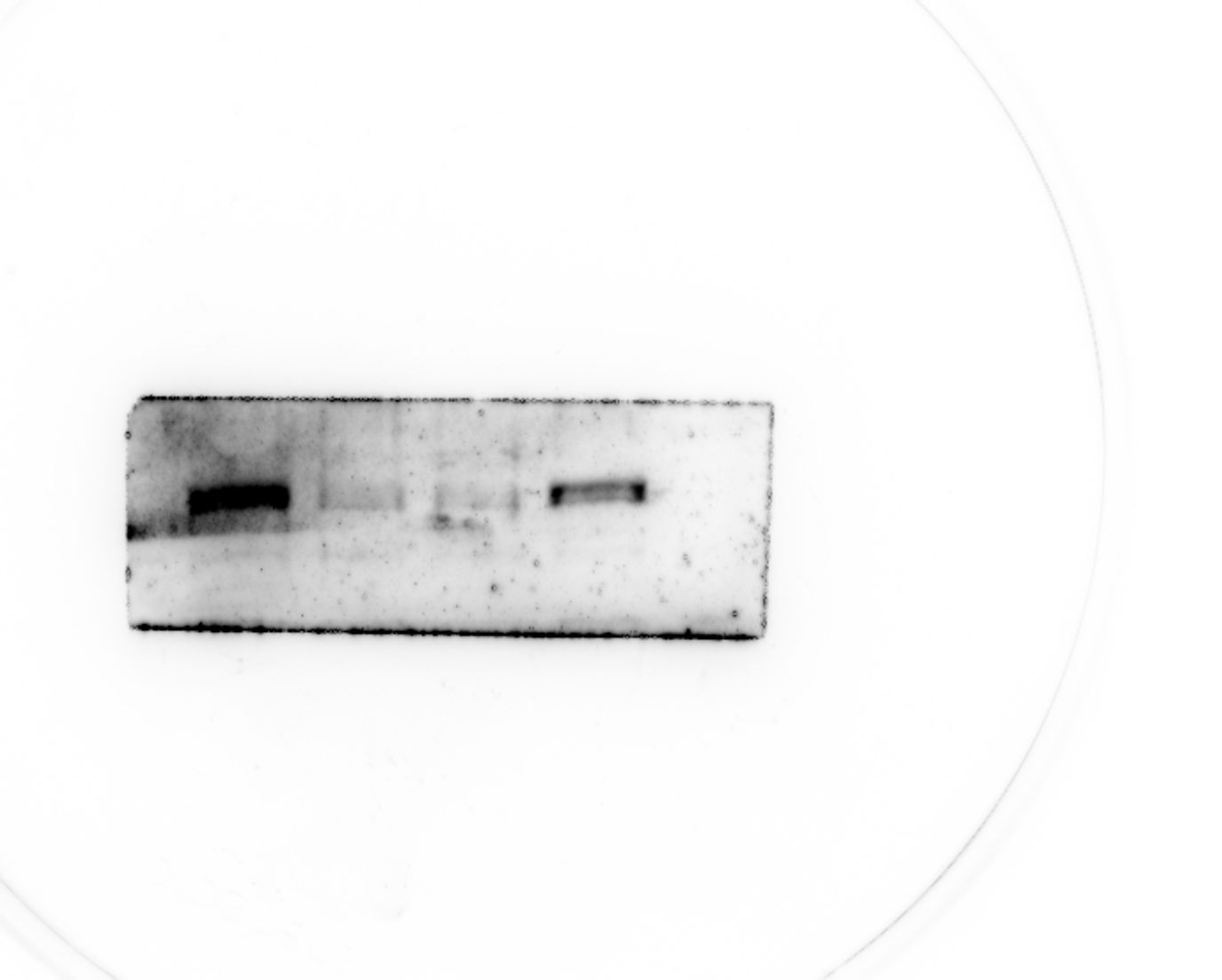

Supplement: Supplemental Information 6 [file peerj-13-19276-s006.zip › C I-R AAV9-CON AAV9-EB1 group western blot-membranal Cx43/9-Cx43.Tif]

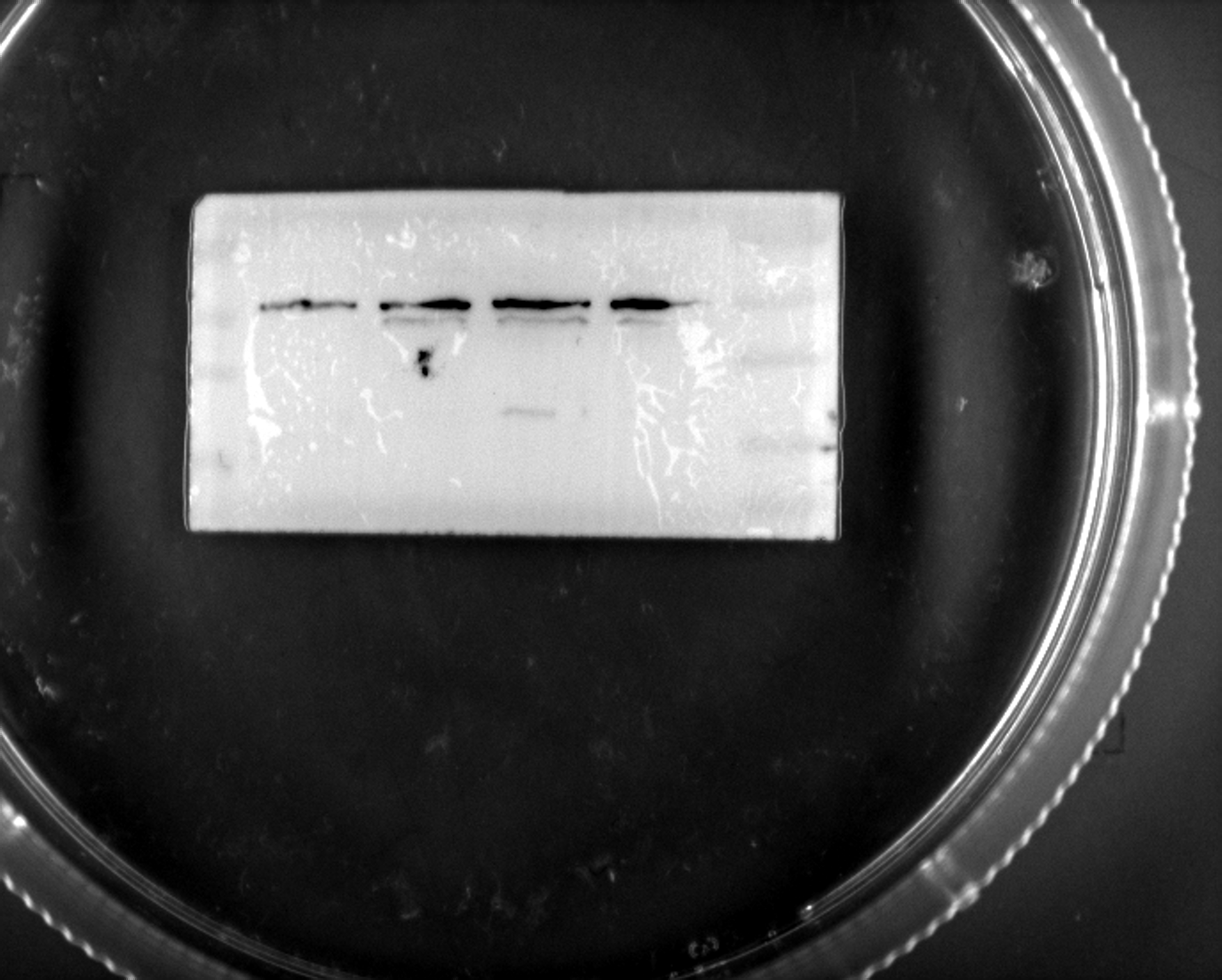

Supplement: Supplemental Information 7 [file peerj-13-19276-s007.zip › C I-R AAV9-CON AAV9-EB1 group western blot-free tubulin/1-Free tubulin-M.Tif]

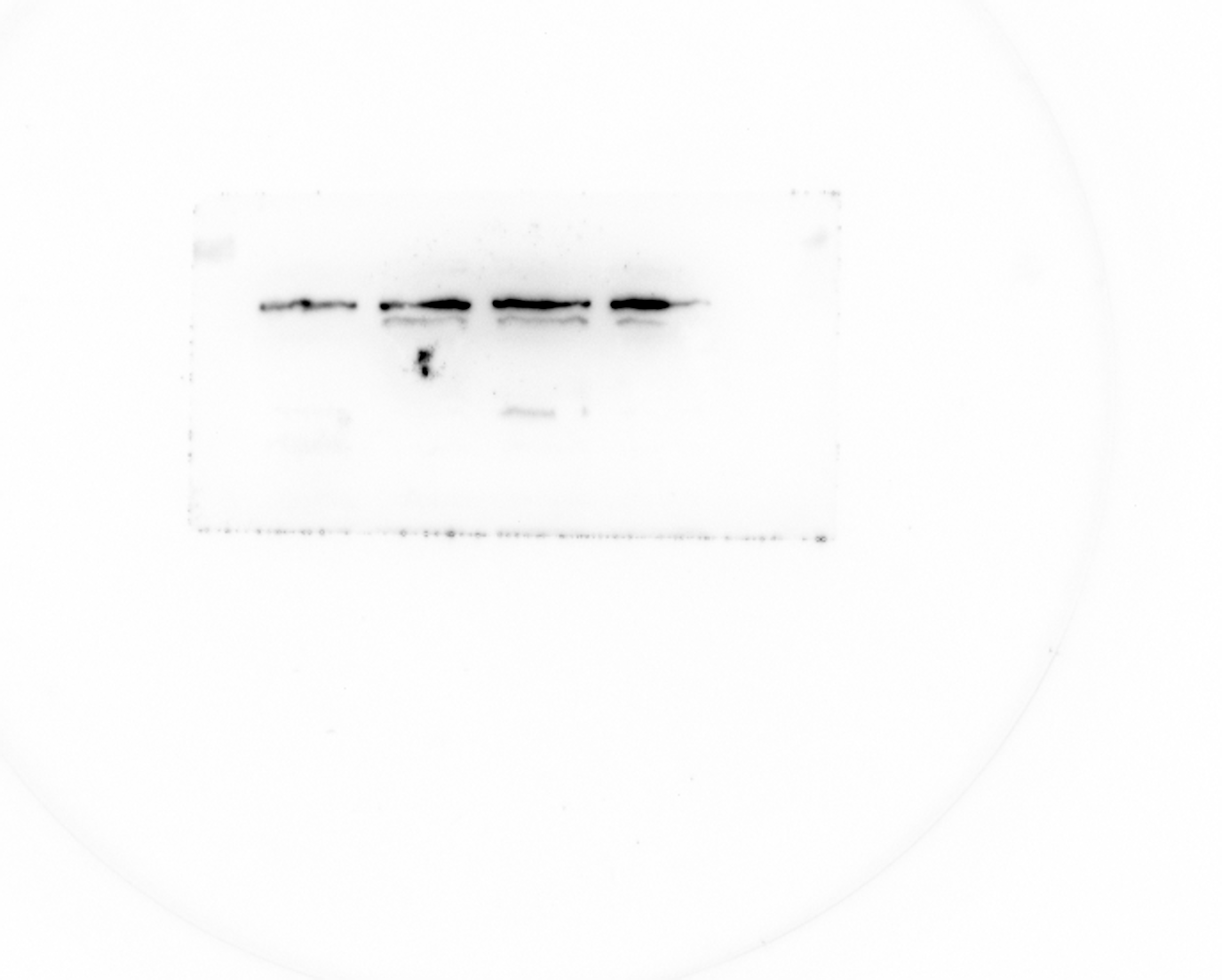

Supplement: Supplemental Information 7 [file peerj-13-19276-s007.zip › C I-R AAV9-CON AAV9-EB1 group western blot-free tubulin/1-Free tubulin.Tif]

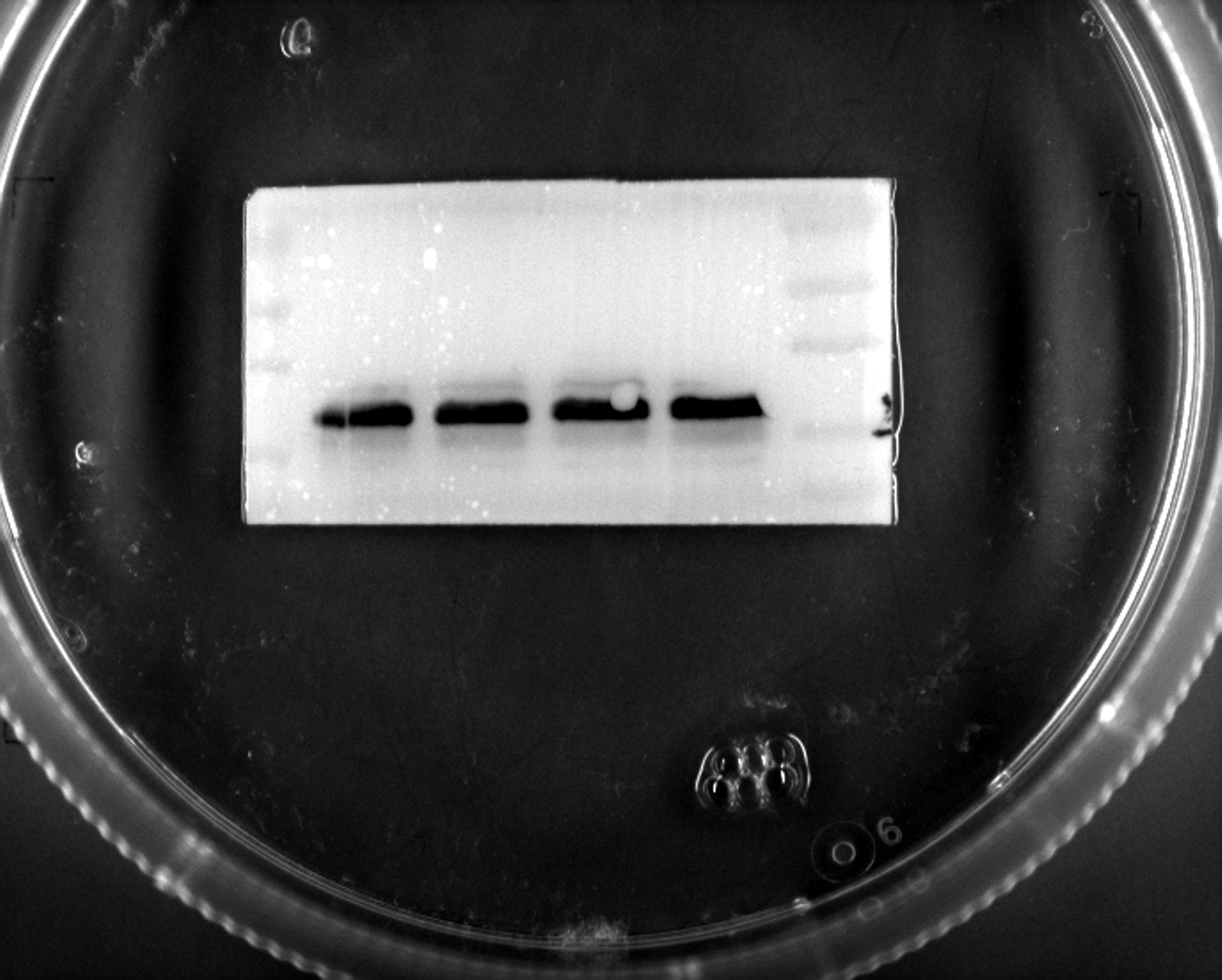

Supplement: Supplemental Information 7 [file peerj-13-19276-s007.zip › C I-R AAV9-CON AAV9-EB1 group western blot-free tubulin/1-GAPDH-M.Tif]

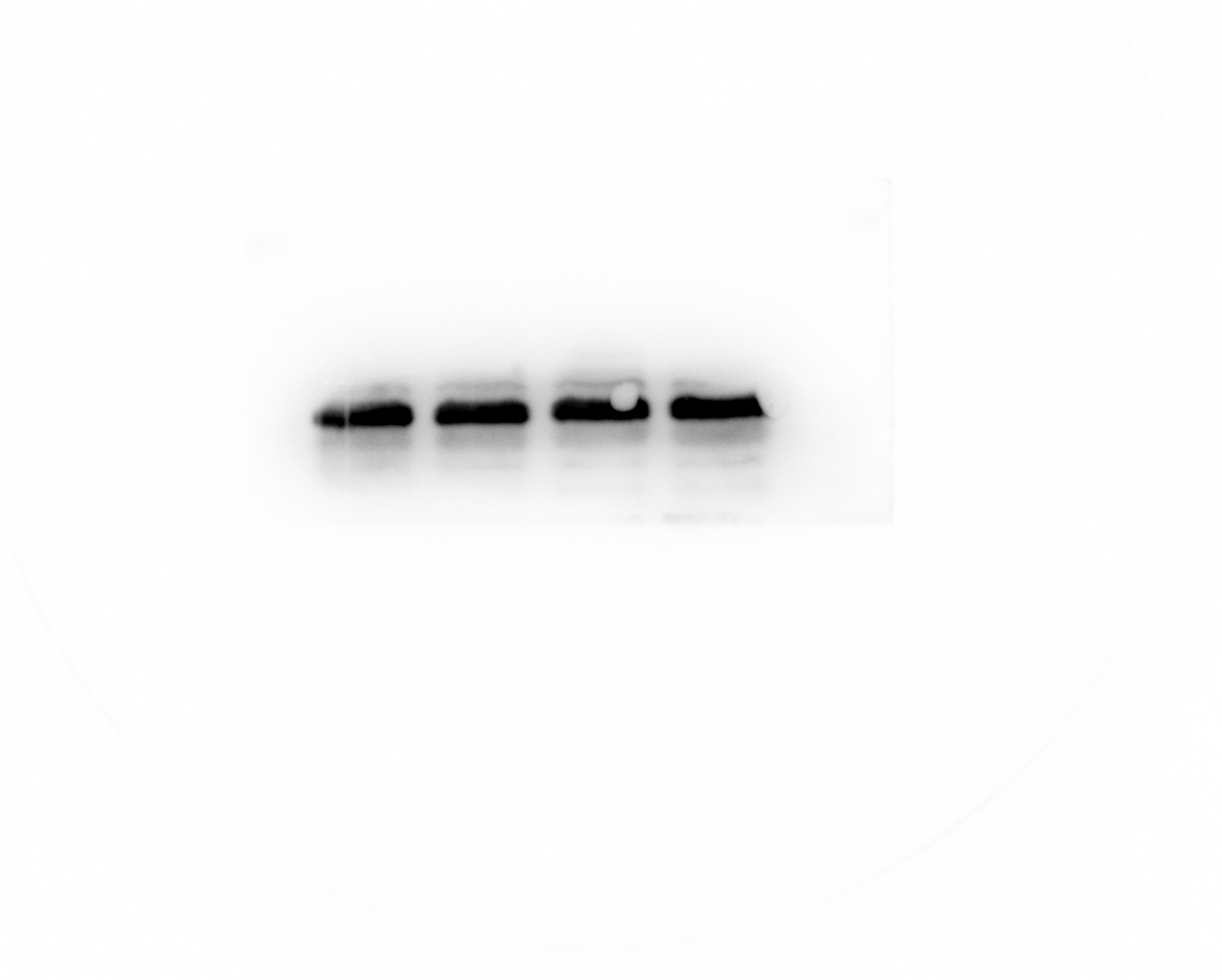

Supplement: Supplemental Information 7 [file peerj-13-19276-s007.zip › C I-R AAV9-CON AAV9-EB1 group western blot-free tubulin/1-GAPDH.Tif]

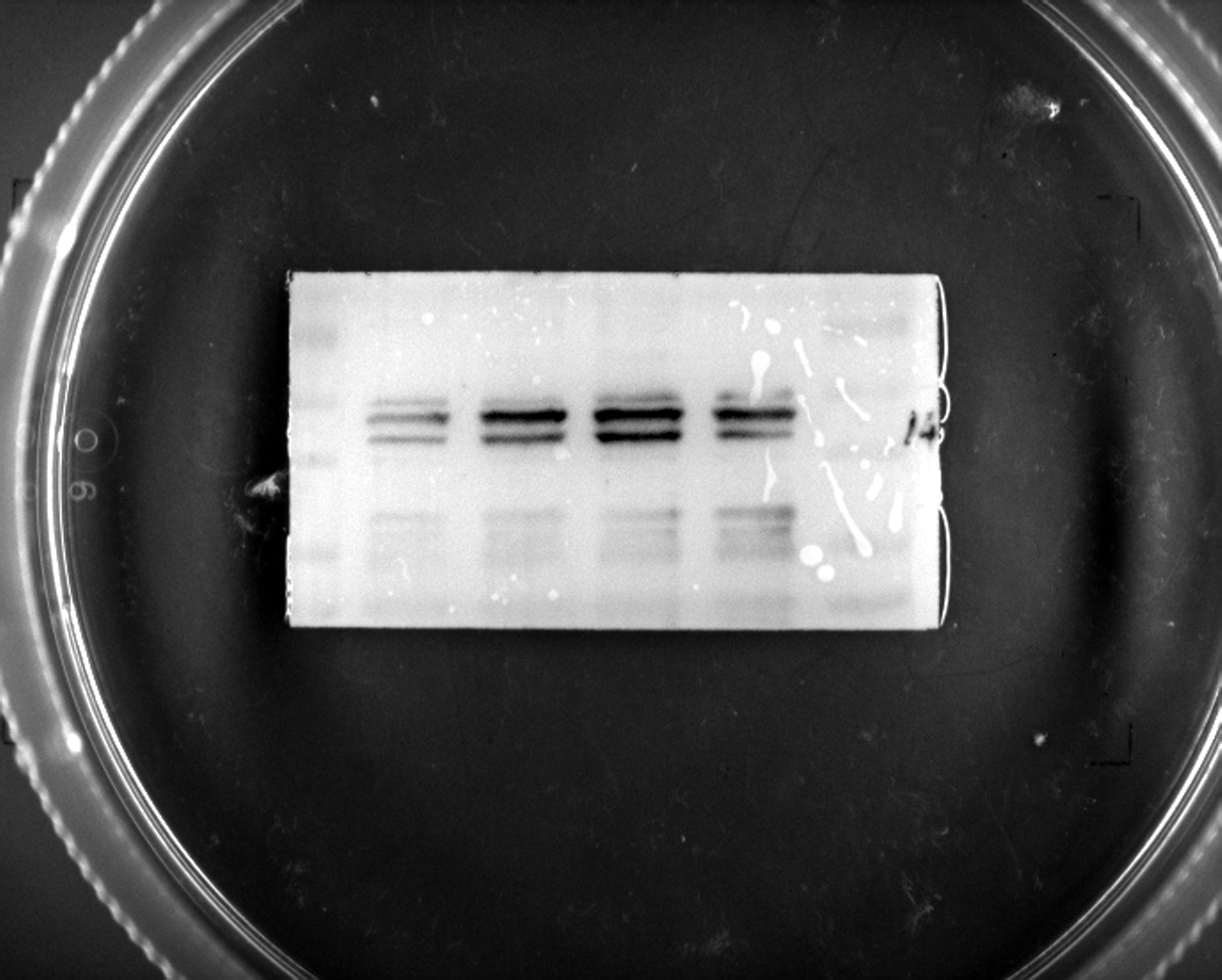

Supplement: Supplemental Information 7 [file peerj-13-19276-s007.zip › C I-R AAV9-CON AAV9-EB1 group western blot-free tubulin/2-Free tubulin-M.Tif]

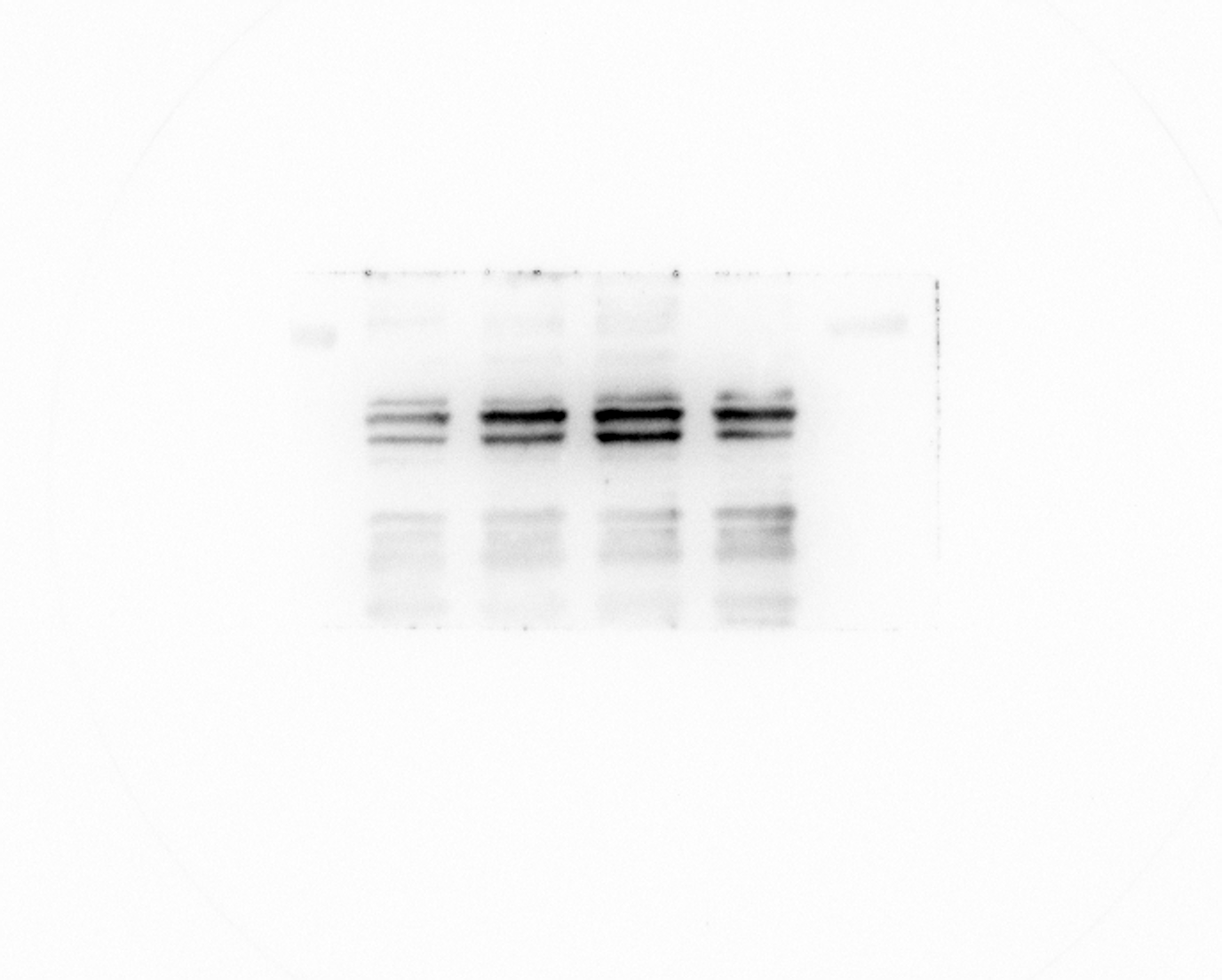

Supplement: Supplemental Information 7 [file peerj-13-19276-s007.zip › C I-R AAV9-CON AAV9-EB1 group western blot-free tubulin/2-Free tubulin.Tif]
