# Supplementary figures and images for: Downregulation of EB1 impedes Cx43 localization and cardiac conduction after hypothermic ischemia-reperfusion in rats (part 3 of 5)
Source: PeerJ. 2025 Apr 14;13:e19276. doi: 10.7717/peerj.19276 (PMC12005192; doi:10.7717/peerj.19276)

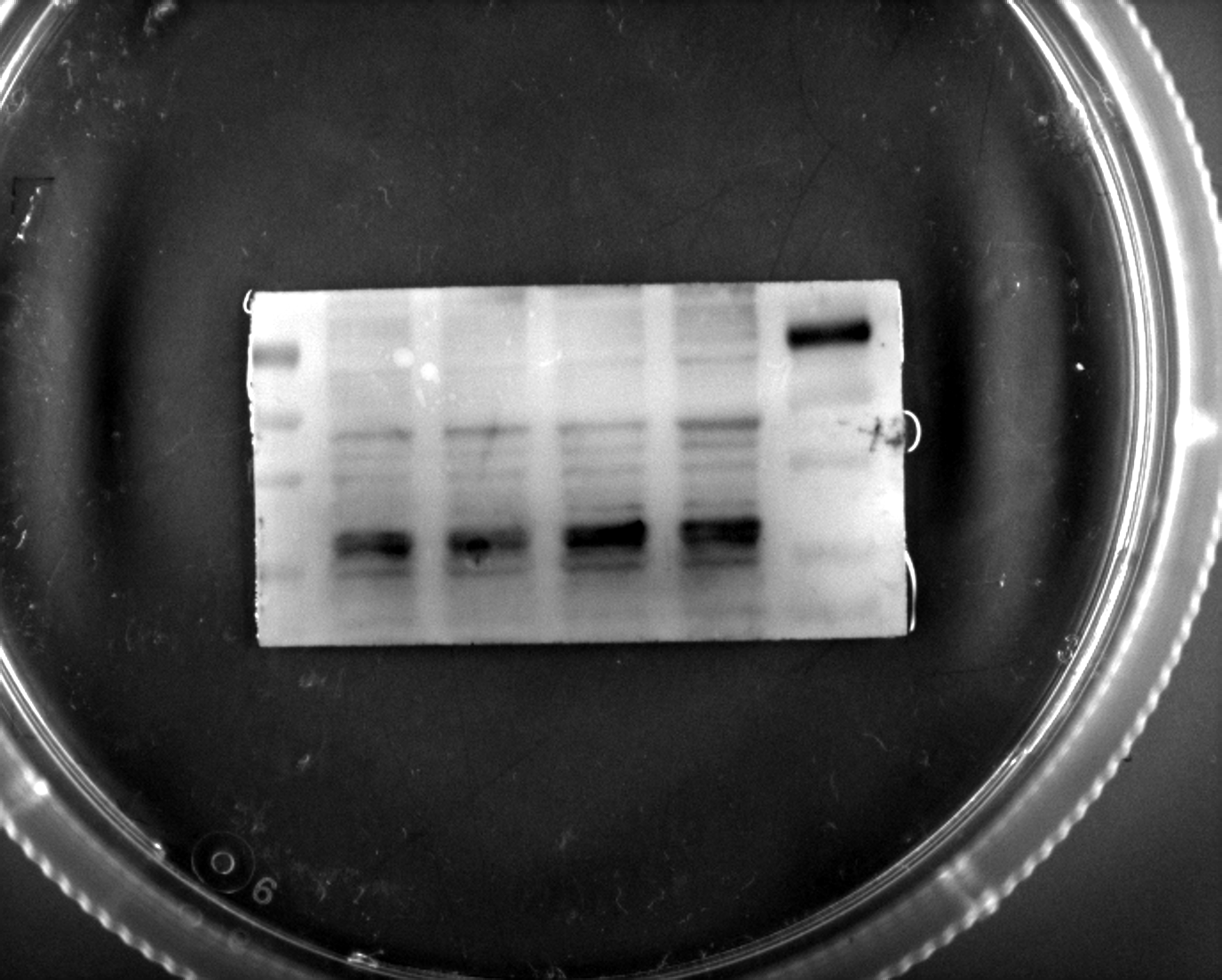

Supplement: Supplemental Information 7 [file peerj-13-19276-s007.zip › C I-R AAV9-CON AAV9-EB1 group western blot-free tubulin/2-GAPDH-M.Tif]

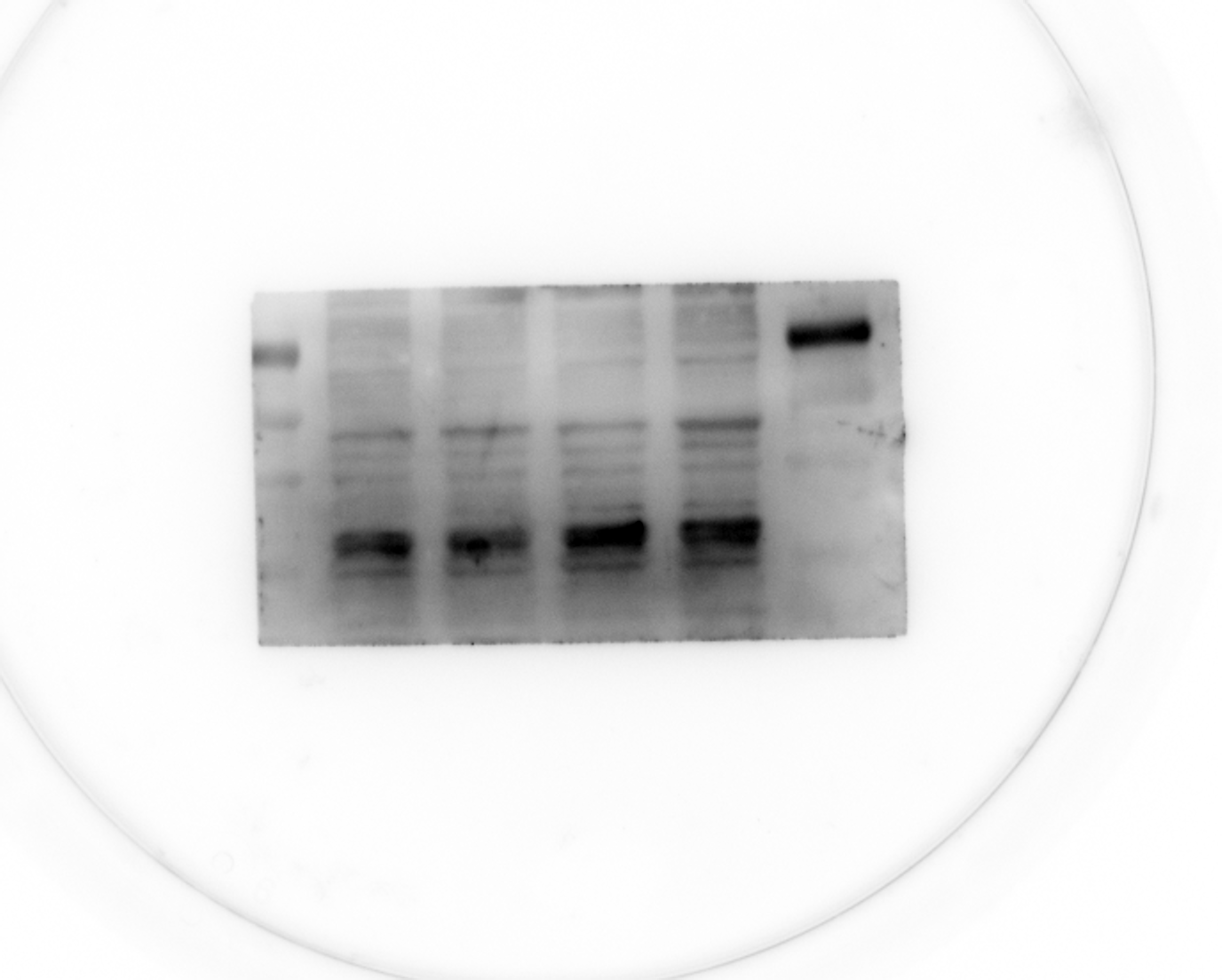

Supplement: Supplemental Information 7 [file peerj-13-19276-s007.zip › C I-R AAV9-CON AAV9-EB1 group western blot-free tubulin/2-GAPDH.Tif]

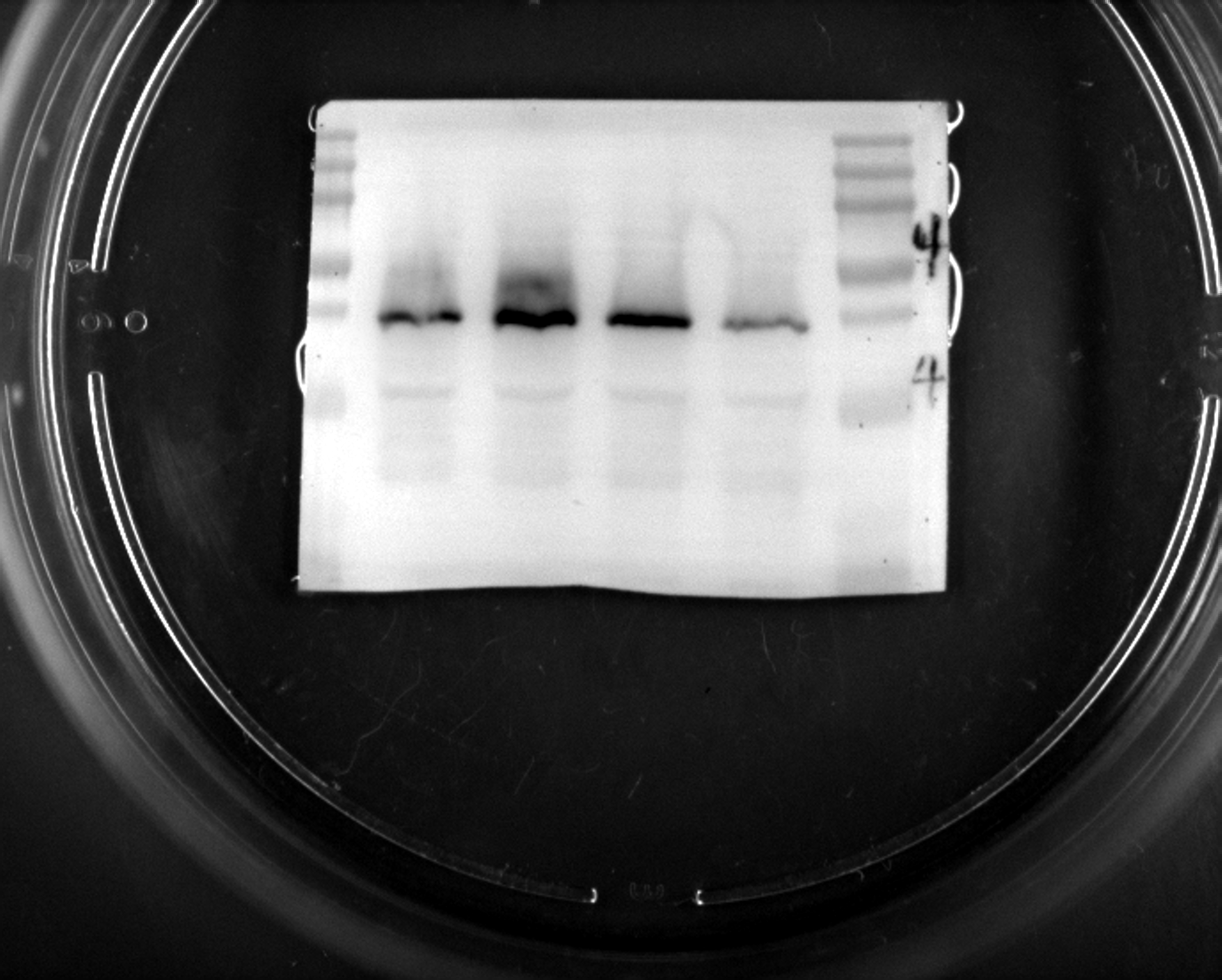

Supplement: Supplemental Information 7 [file peerj-13-19276-s007.zip › C I-R AAV9-CON AAV9-EB1 group western blot-free tubulin/3-Free tubulin-M-used.Tif]

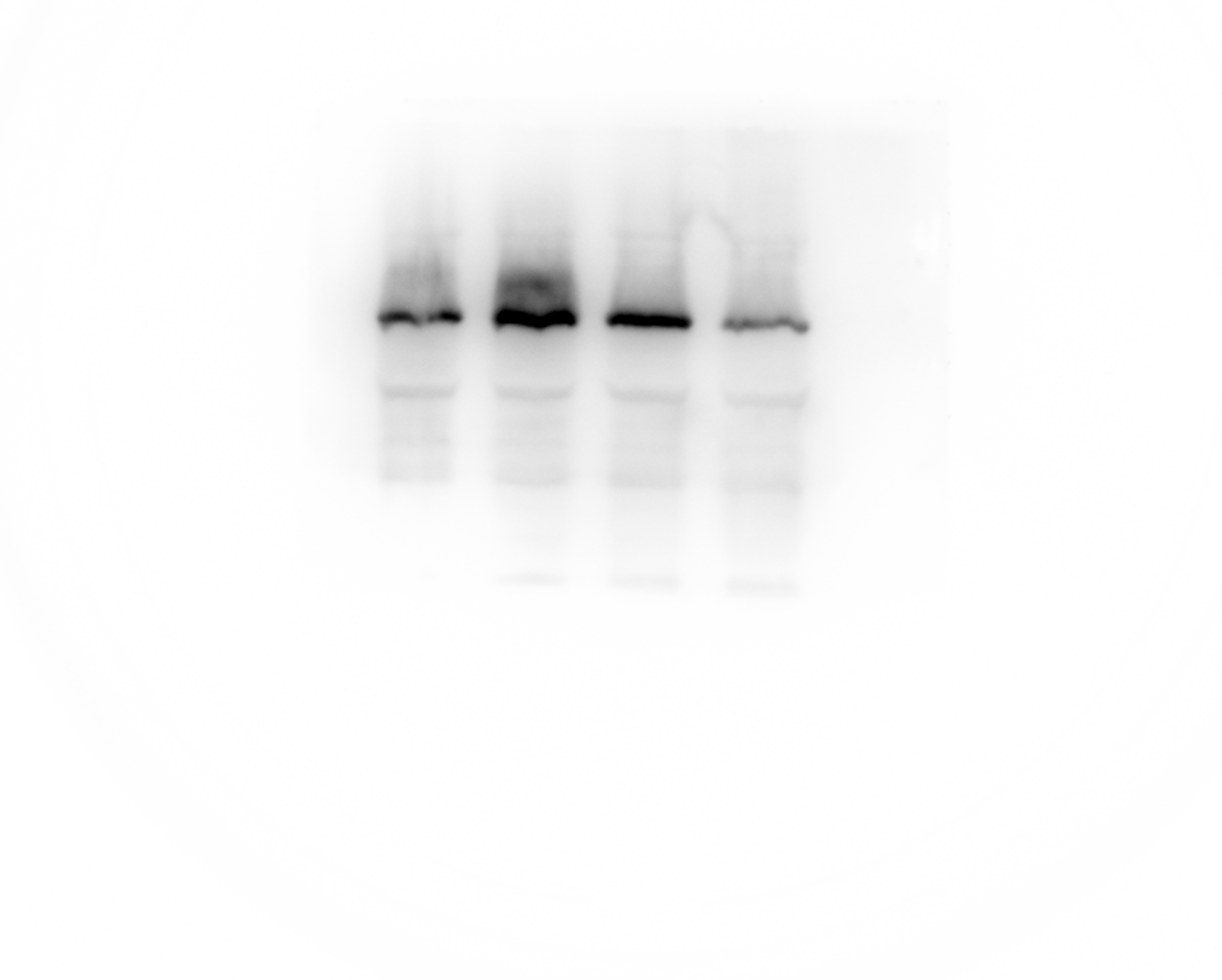

Supplement: Supplemental Information 7 [file peerj-13-19276-s007.zip › C I-R AAV9-CON AAV9-EB1 group western blot-free tubulin/3-Free tubulin-used.Tif]

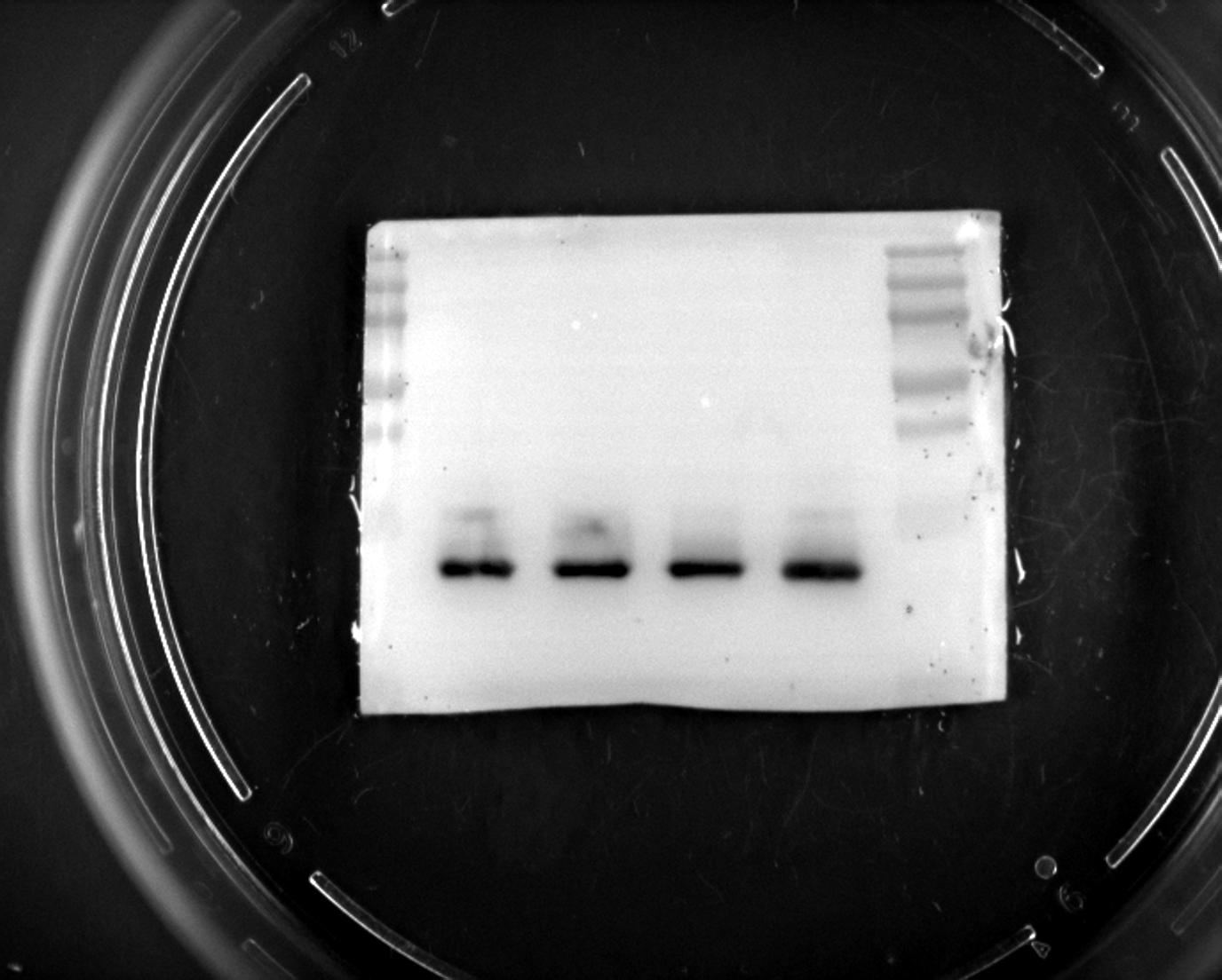

Supplement: Supplemental Information 7 [file peerj-13-19276-s007.zip › C I-R AAV9-CON AAV9-EB1 group western blot-free tubulin/3-GAPDH-M-used.Tif]

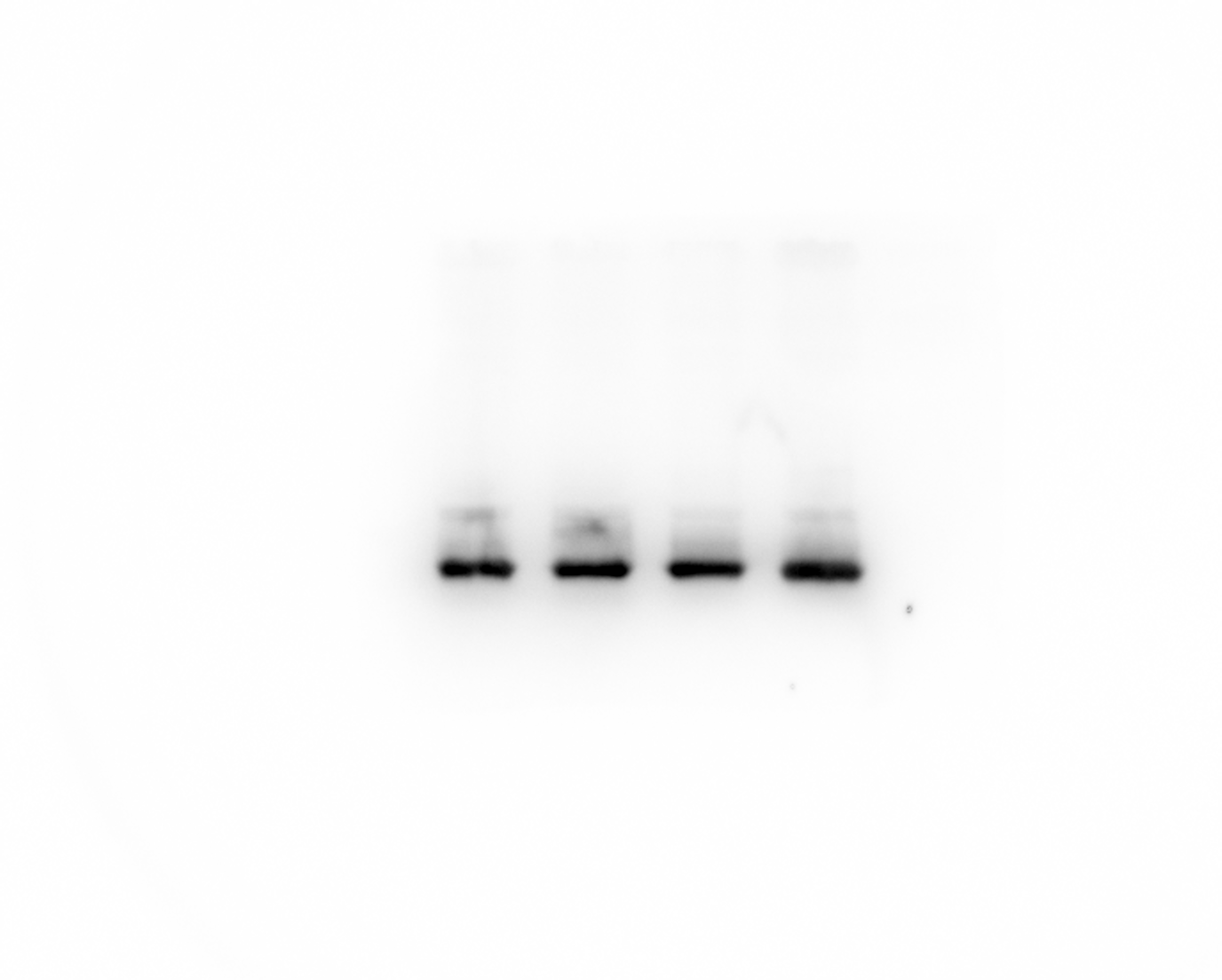

Supplement: Supplemental Information 7 [file peerj-13-19276-s007.zip › C I-R AAV9-CON AAV9-EB1 group western blot-free tubulin/3-GAPDH-used.Tif]

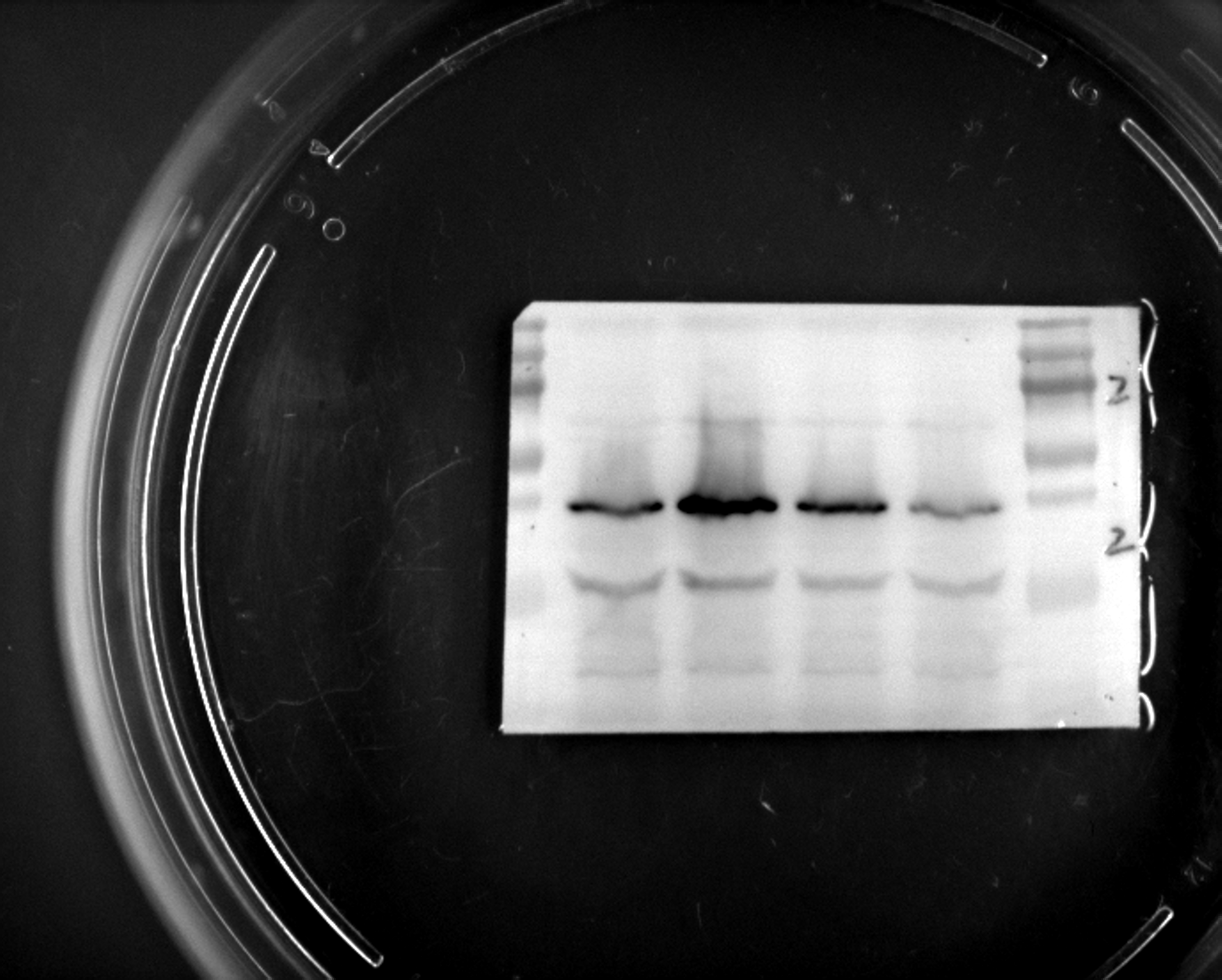

Supplement: Supplemental Information 7 [file peerj-13-19276-s007.zip › C I-R AAV9-CON AAV9-EB1 group western blot-free tubulin/4-Free tubulin-M.Tif]

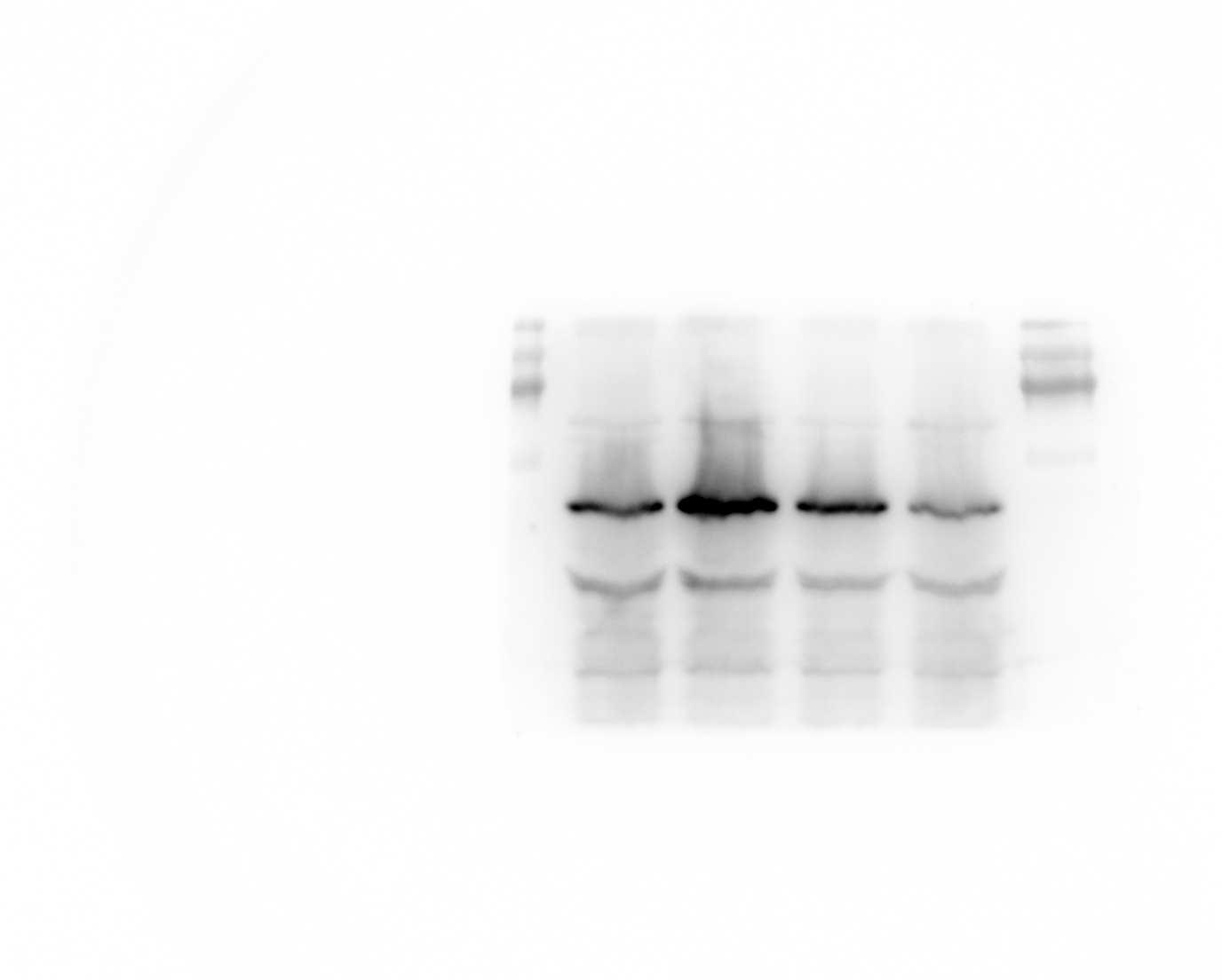

Supplement: Supplemental Information 7 [file peerj-13-19276-s007.zip › C I-R AAV9-CON AAV9-EB1 group western blot-free tubulin/4-Free tubulin.Tif]

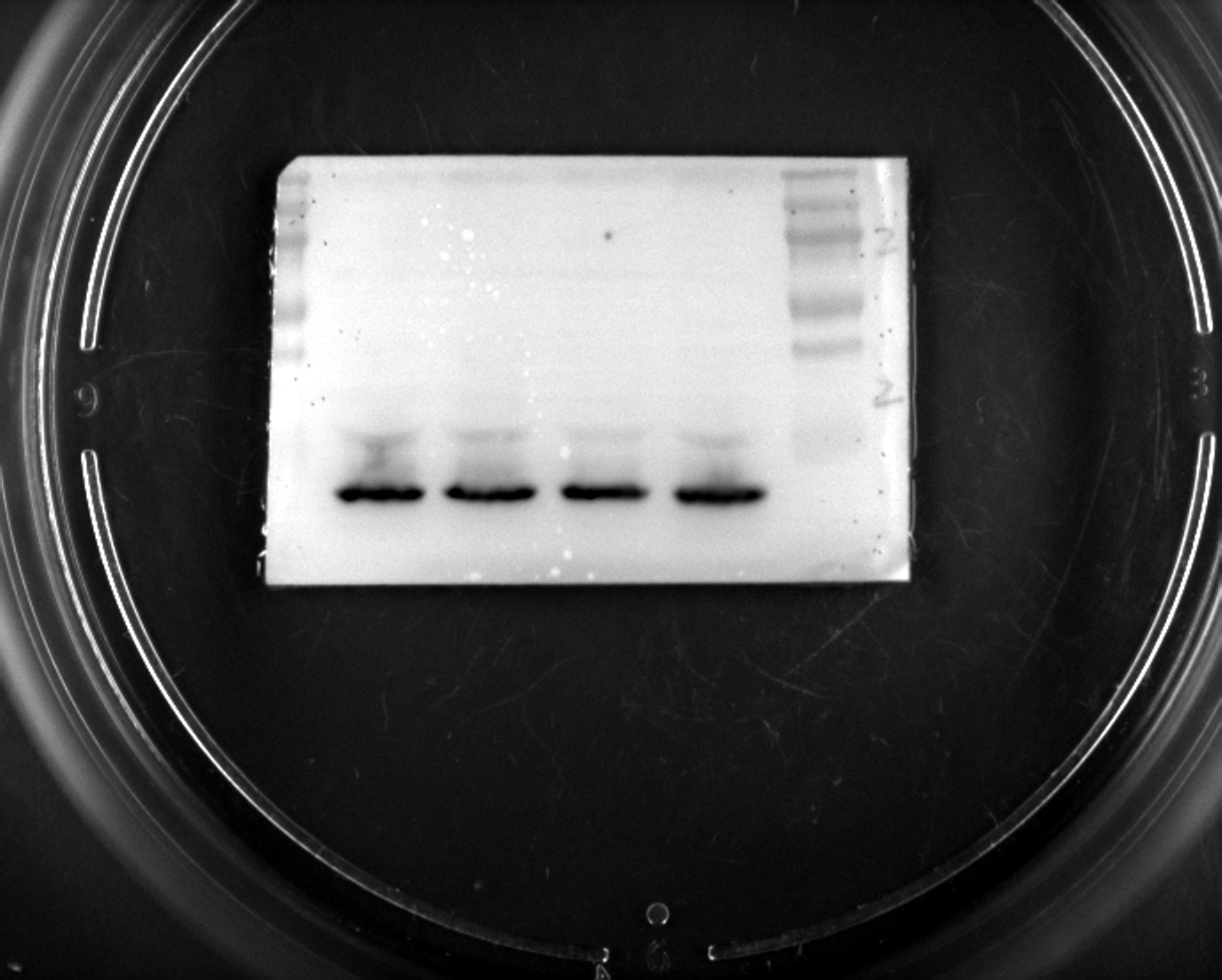

Supplement: Supplemental Information 7 [file peerj-13-19276-s007.zip › C I-R AAV9-CON AAV9-EB1 group western blot-free tubulin/4-GAPDH-M.Tif]

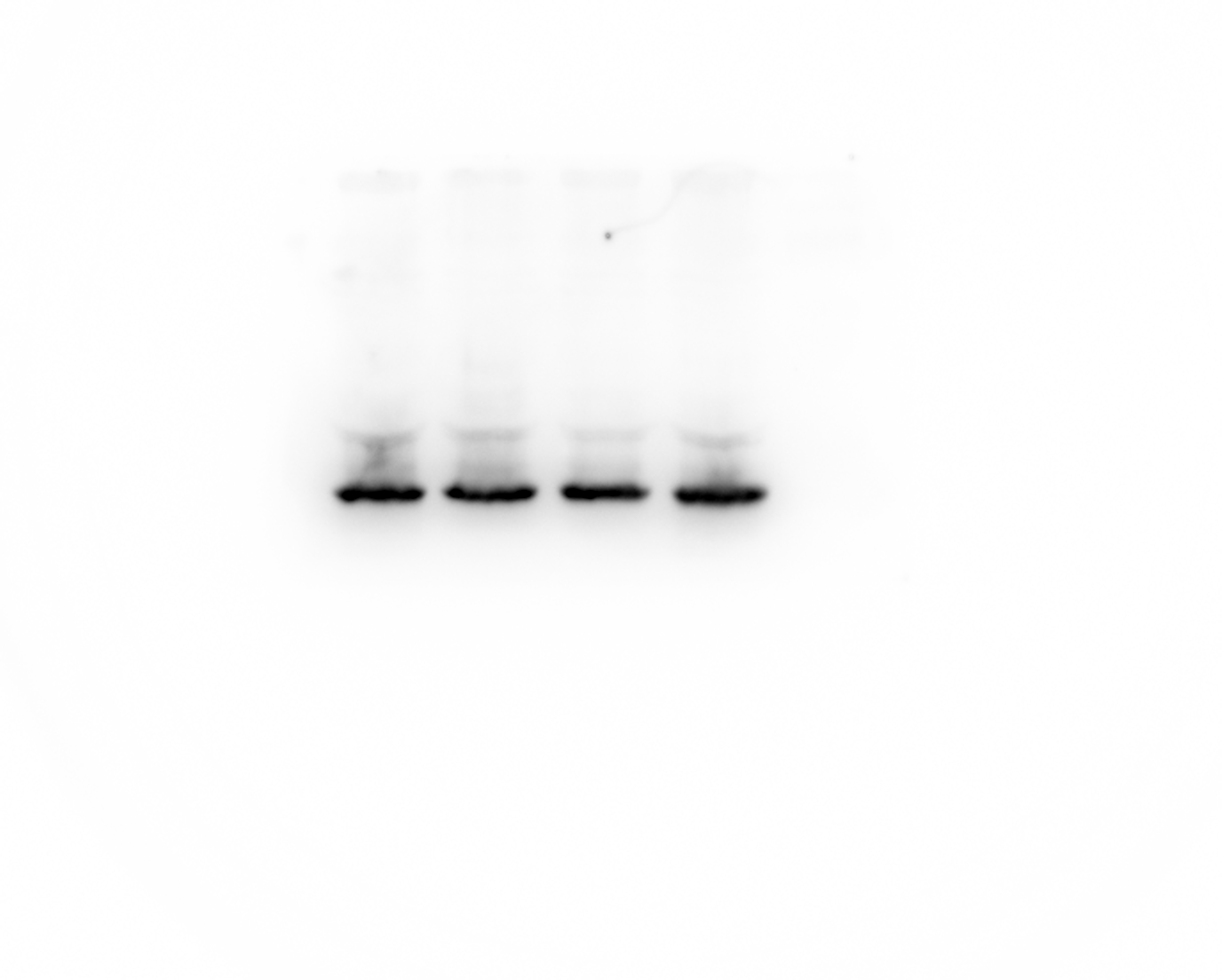

Supplement: Supplemental Information 7 [file peerj-13-19276-s007.zip › C I-R AAV9-CON AAV9-EB1 group western blot-free tubulin/4-GAPDH.Tif]

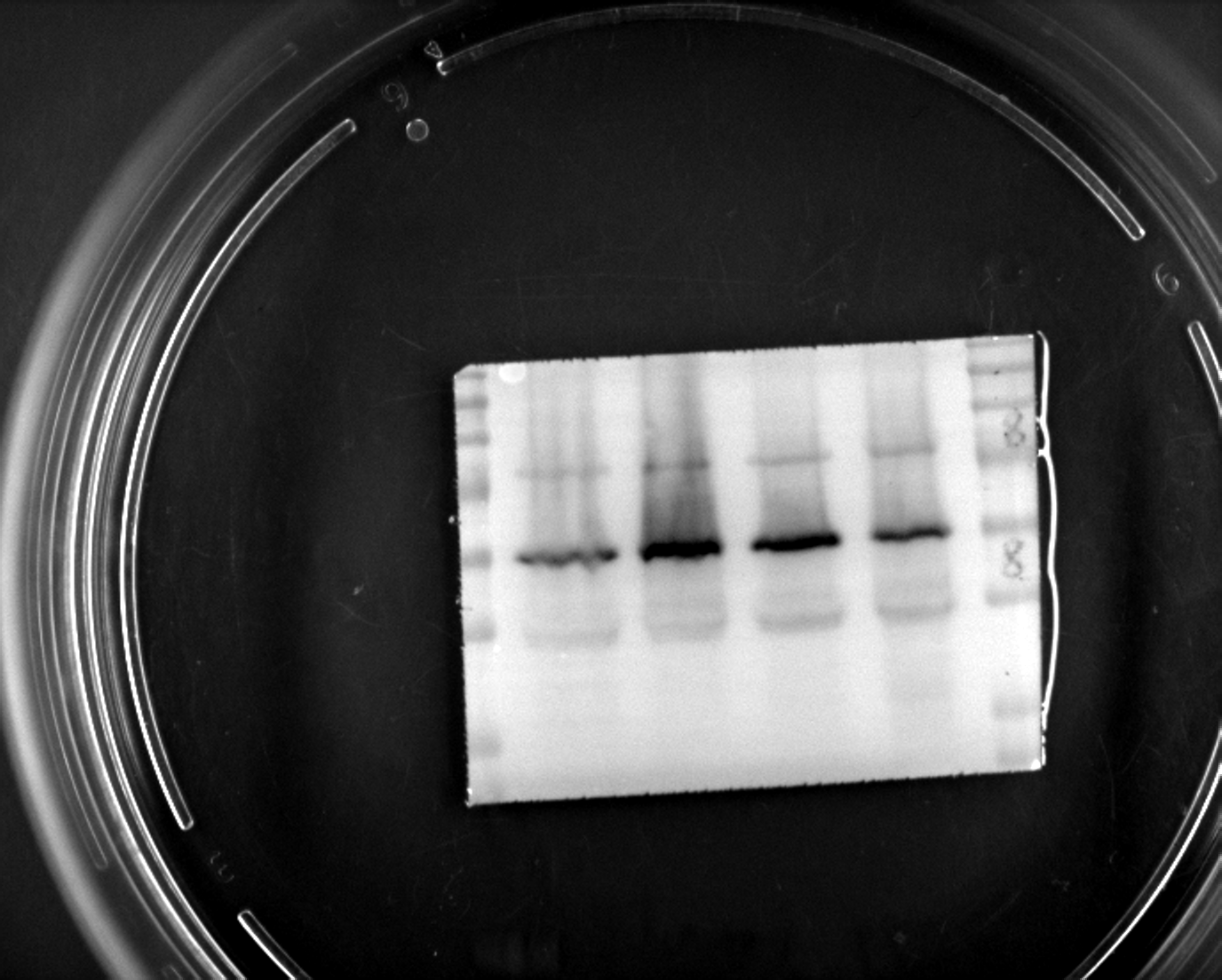

Supplement: Supplemental Information 7 [file peerj-13-19276-s007.zip › C I-R AAV9-CON AAV9-EB1 group western blot-free tubulin/5-Free tubulin-M.Tif]

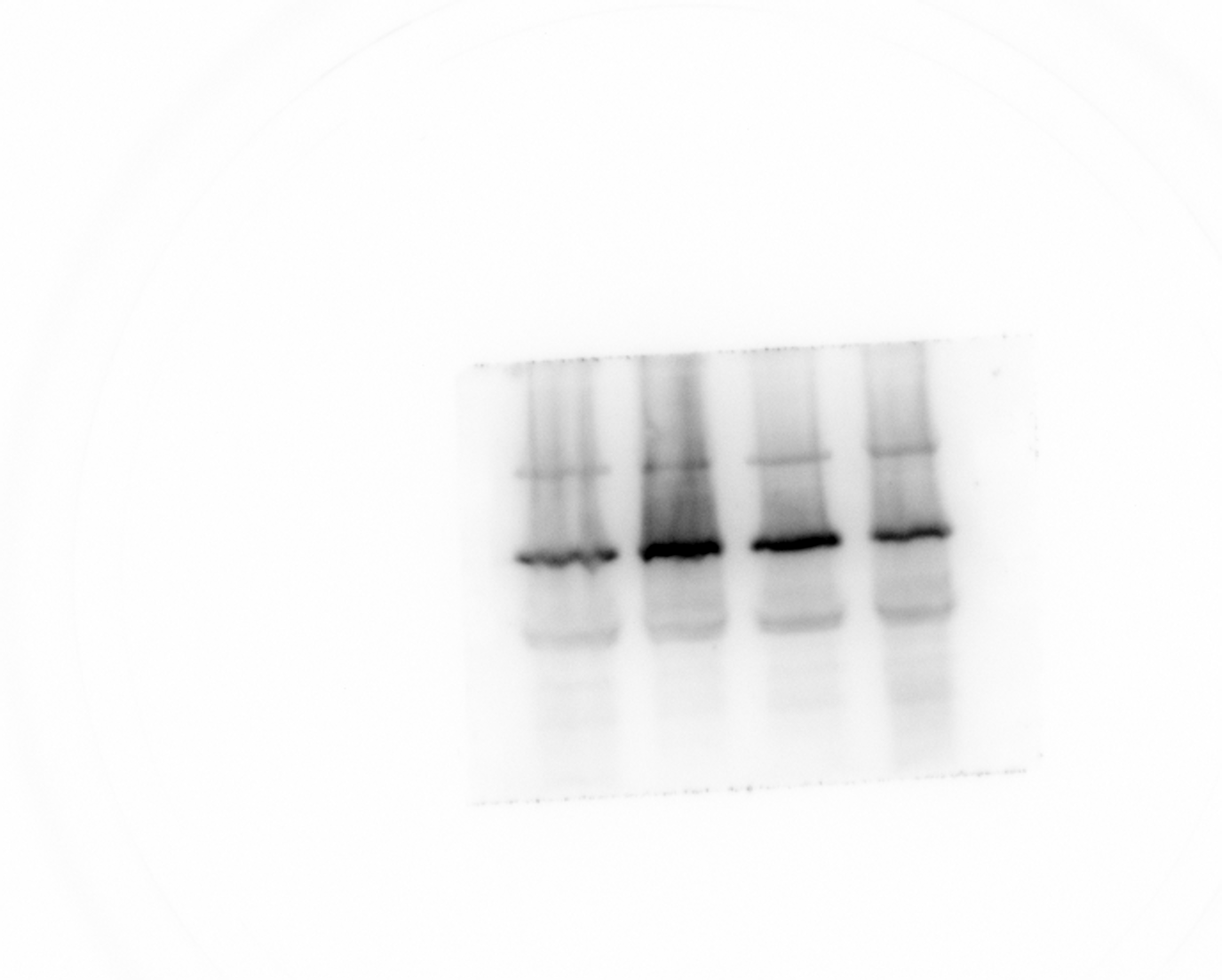

Supplement: Supplemental Information 7 [file peerj-13-19276-s007.zip › C I-R AAV9-CON AAV9-EB1 group western blot-free tubulin/5-Free tubulin.Tif]

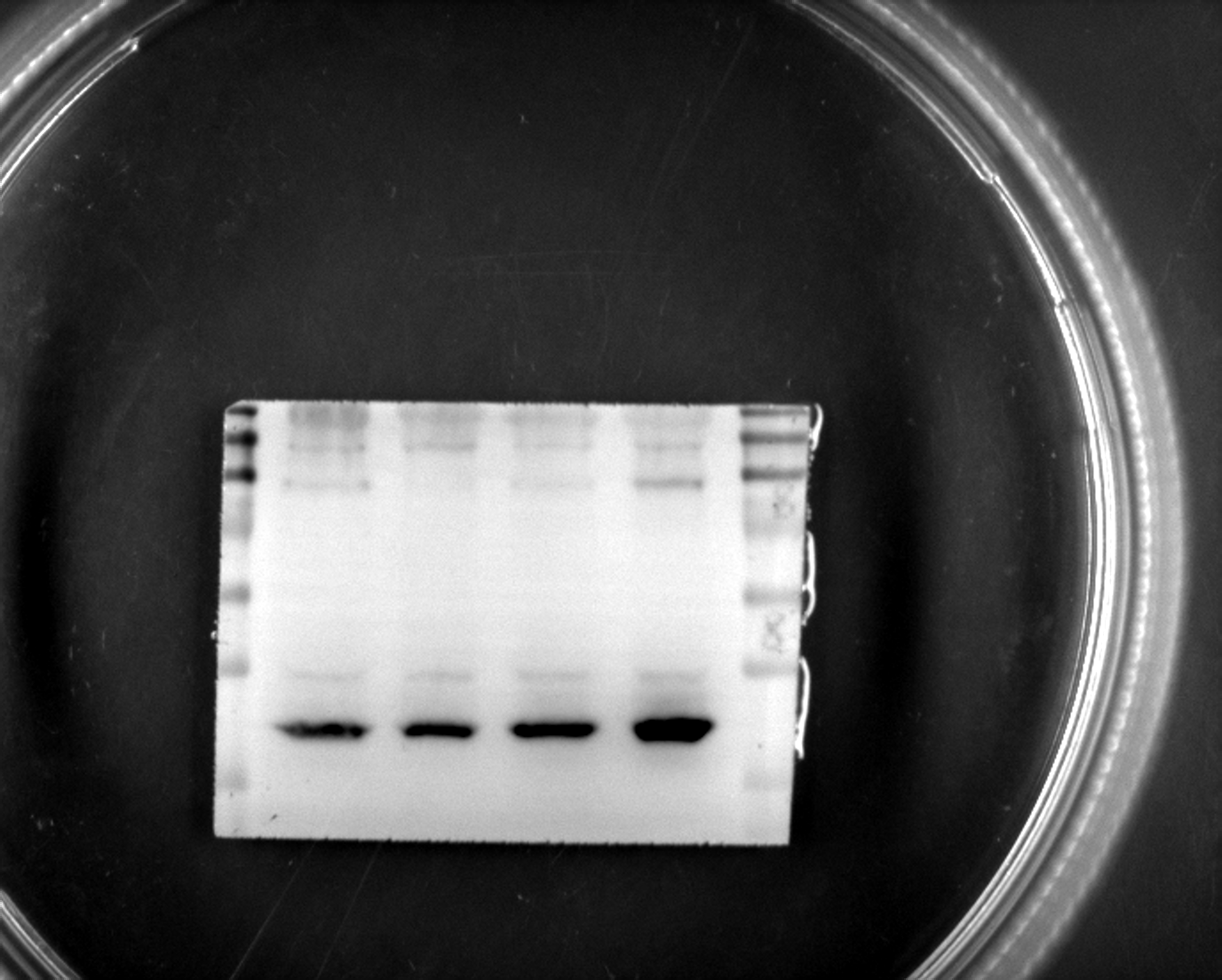

Supplement: Supplemental Information 7 [file peerj-13-19276-s007.zip › C I-R AAV9-CON AAV9-EB1 group western blot-free tubulin/5-GAPDH-M.Tif]

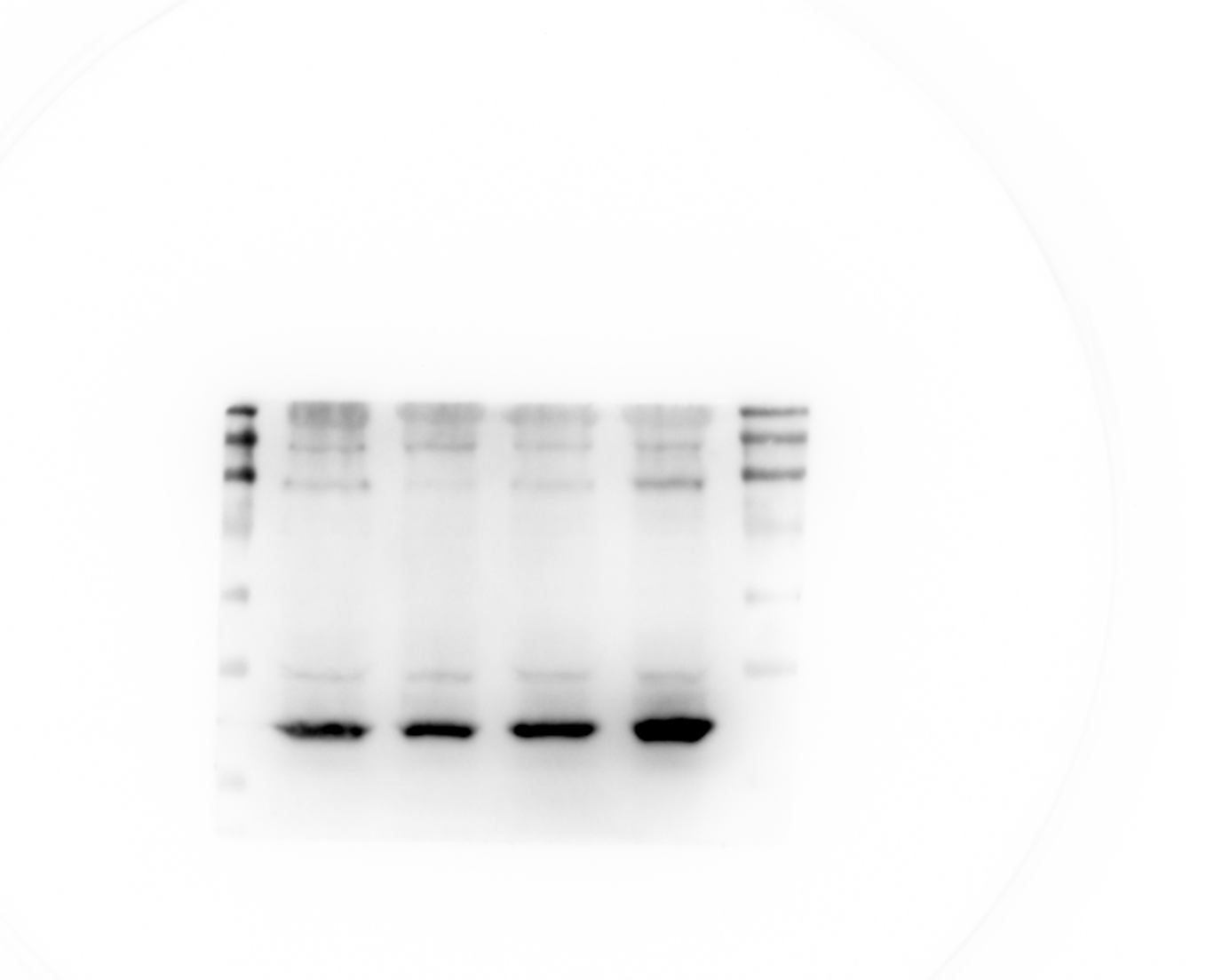

Supplement: Supplemental Information 7 [file peerj-13-19276-s007.zip › C I-R AAV9-CON AAV9-EB1 group western blot-free tubulin/5-GAPDH.Tif]

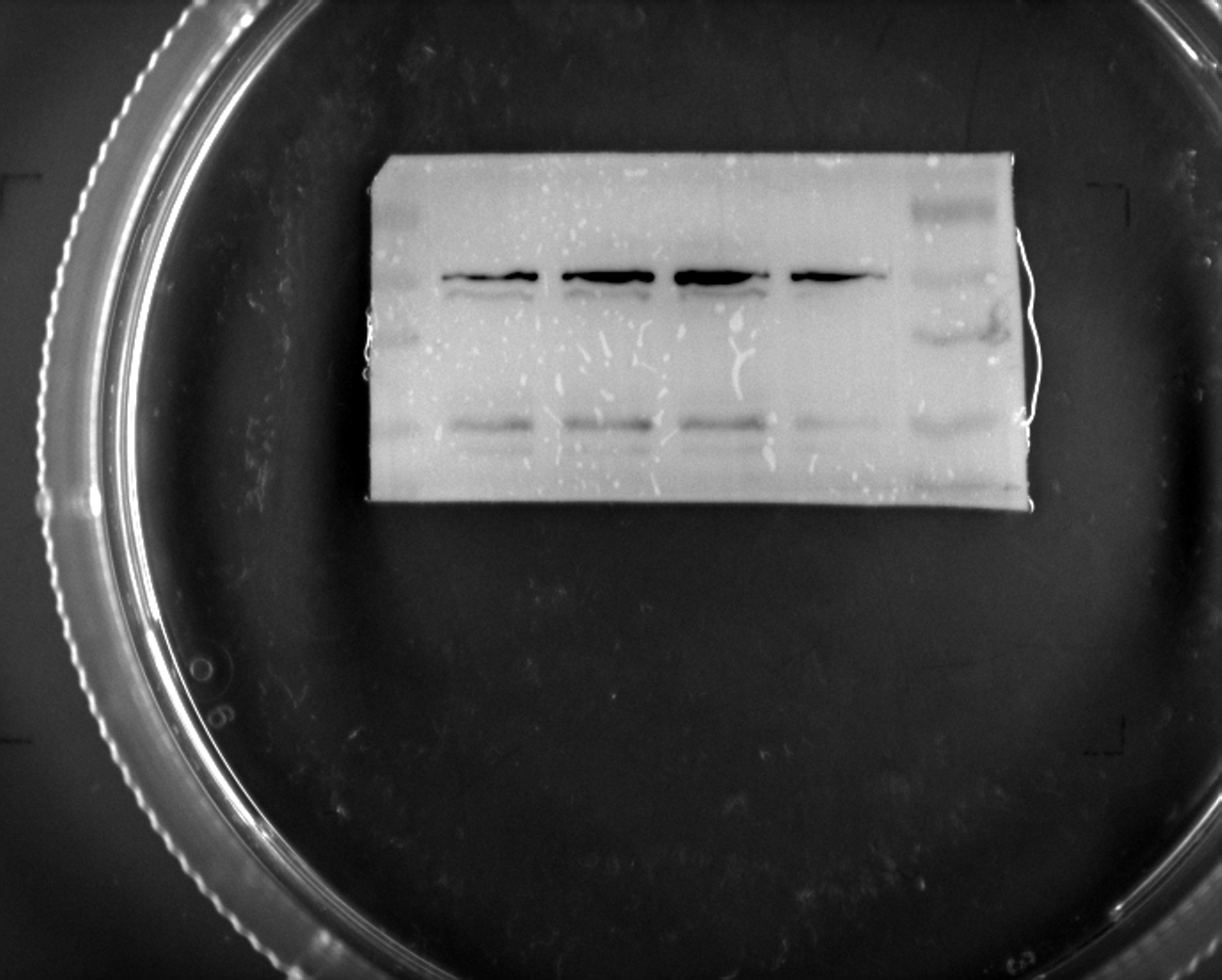

Supplement: Supplemental Information 7 [file peerj-13-19276-s007.zip › C I-R AAV9-CON AAV9-EB1 group western blot-free tubulin/6-Free tubulin-M.Tif]

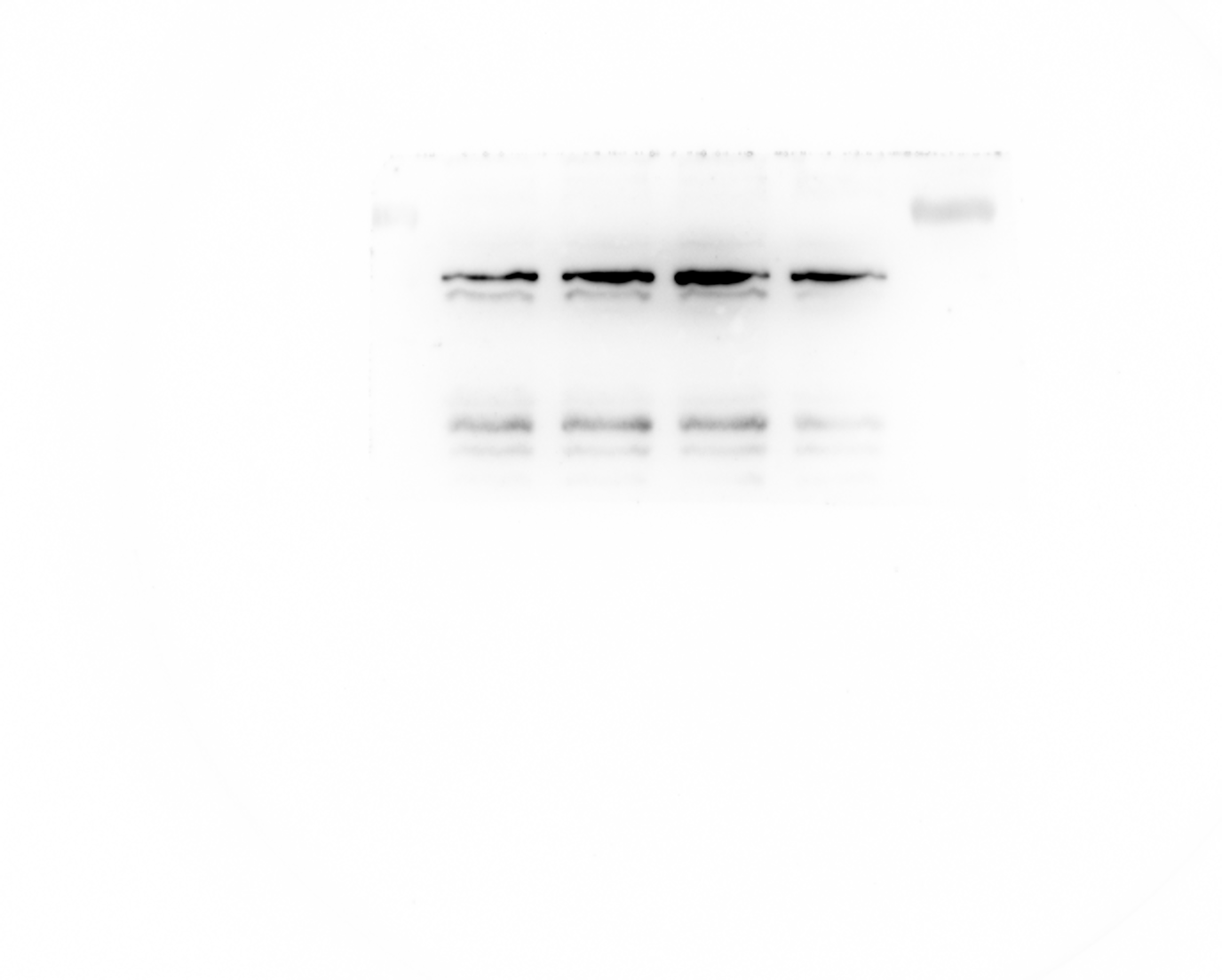

Supplement: Supplemental Information 7 [file peerj-13-19276-s007.zip › C I-R AAV9-CON AAV9-EB1 group western blot-free tubulin/6-Free tubulin.Tif]

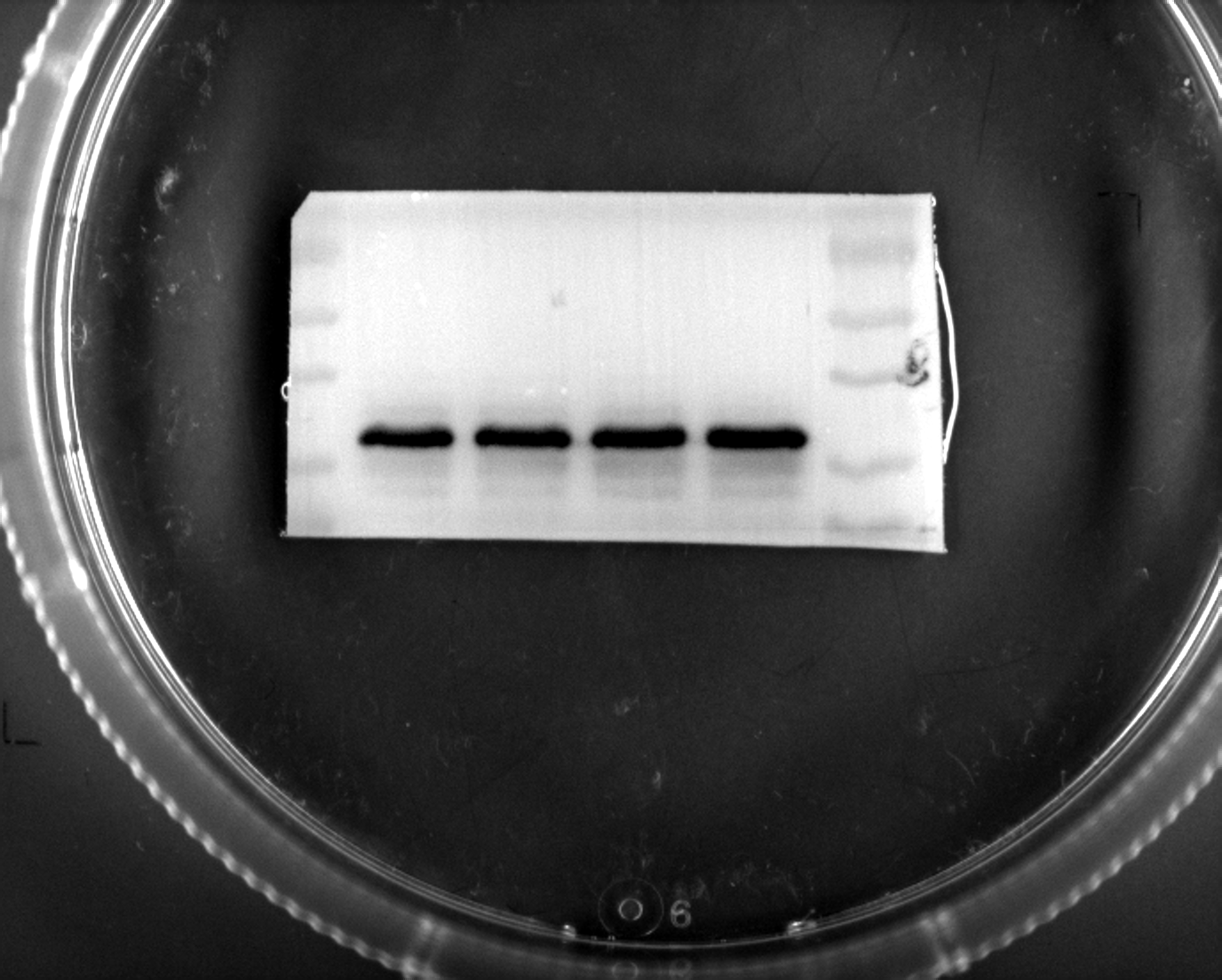

Supplement: Supplemental Information 7 [file peerj-13-19276-s007.zip › C I-R AAV9-CON AAV9-EB1 group western blot-free tubulin/6-GAPDH-M.Tif]

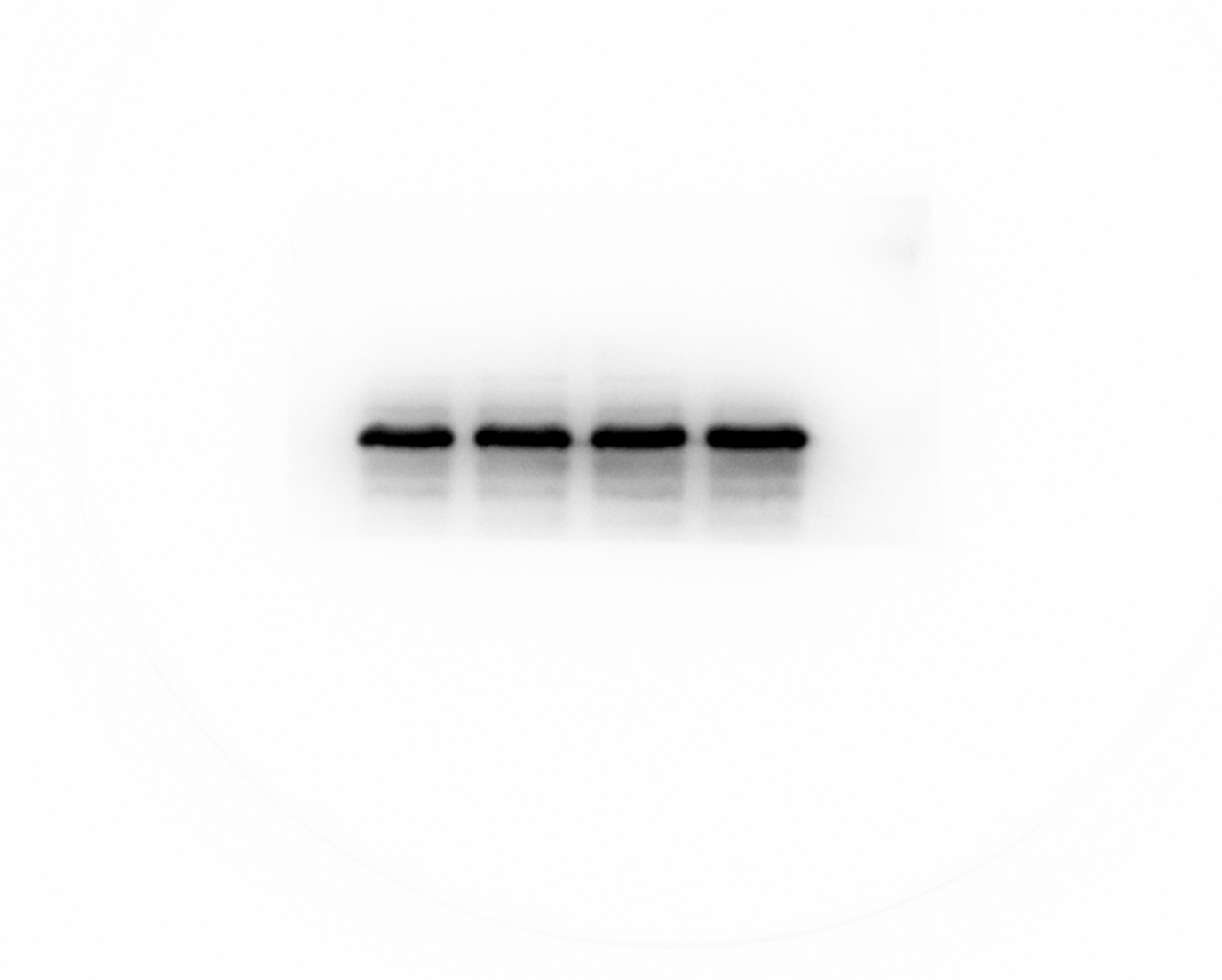

Supplement: Supplemental Information 7 [file peerj-13-19276-s007.zip › C I-R AAV9-CON AAV9-EB1 group western blot-free tubulin/6-GAPDH.Tif]

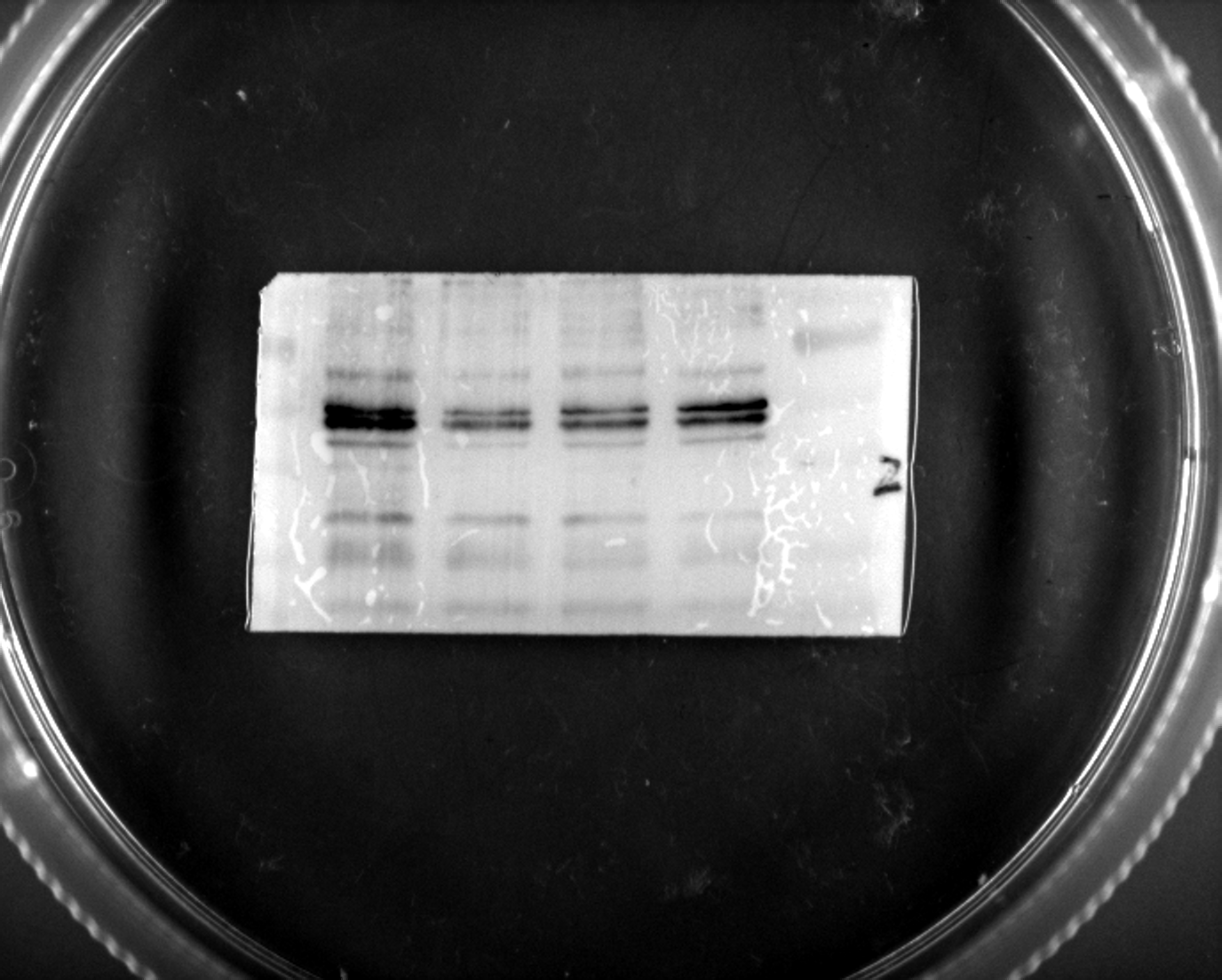

Supplement: Supplemental Information 8 [file peerj-13-19276-s008.zip › C I-R AAV9-CON AAV9-EB1 group western blot-Polymeric tubulin/1-Polymeric tubulin-M.Tif]

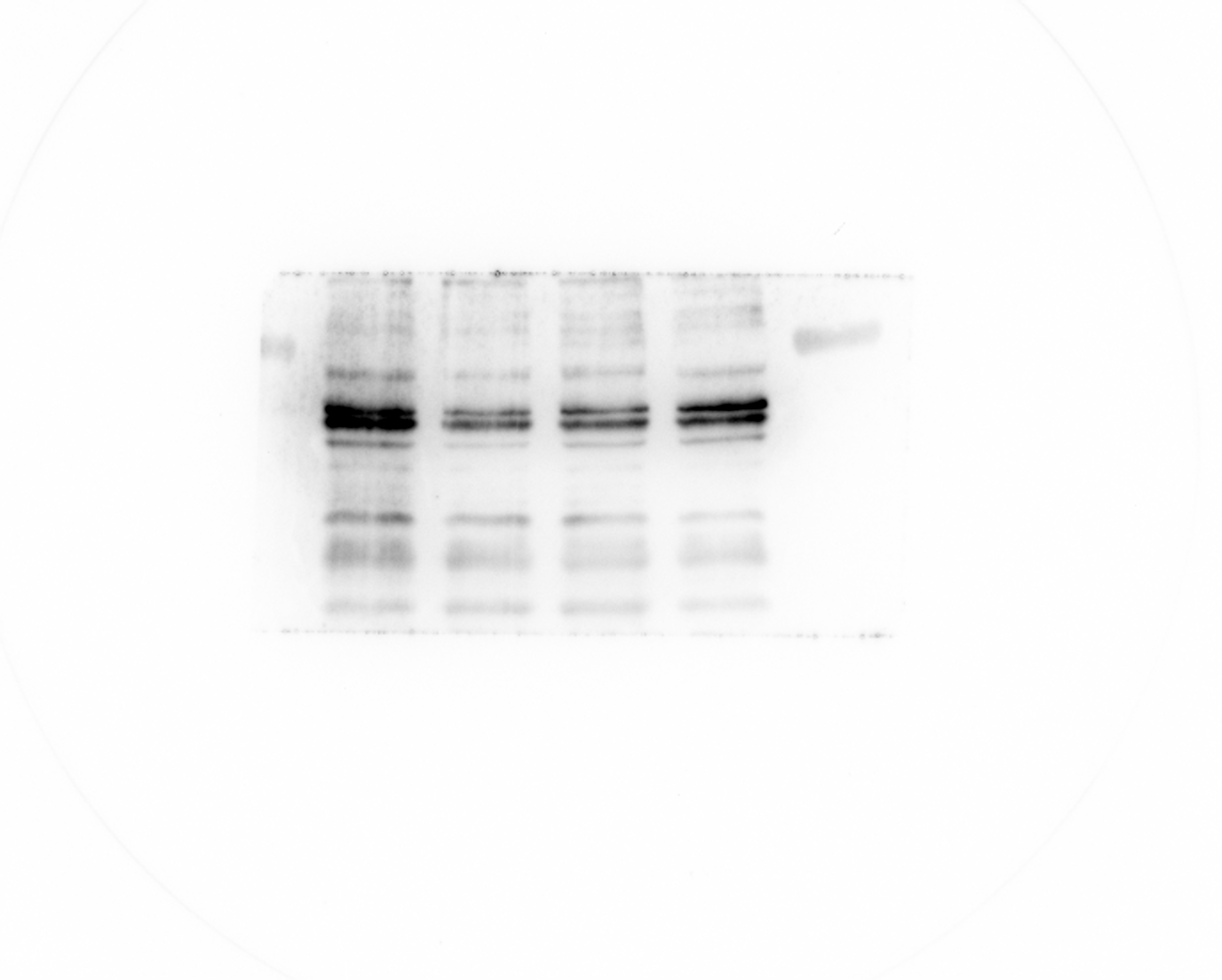

Supplement: Supplemental Information 8 [file peerj-13-19276-s008.zip › C I-R AAV9-CON AAV9-EB1 group western blot-Polymeric tubulin/1-Polymeric tubulin.Tif]

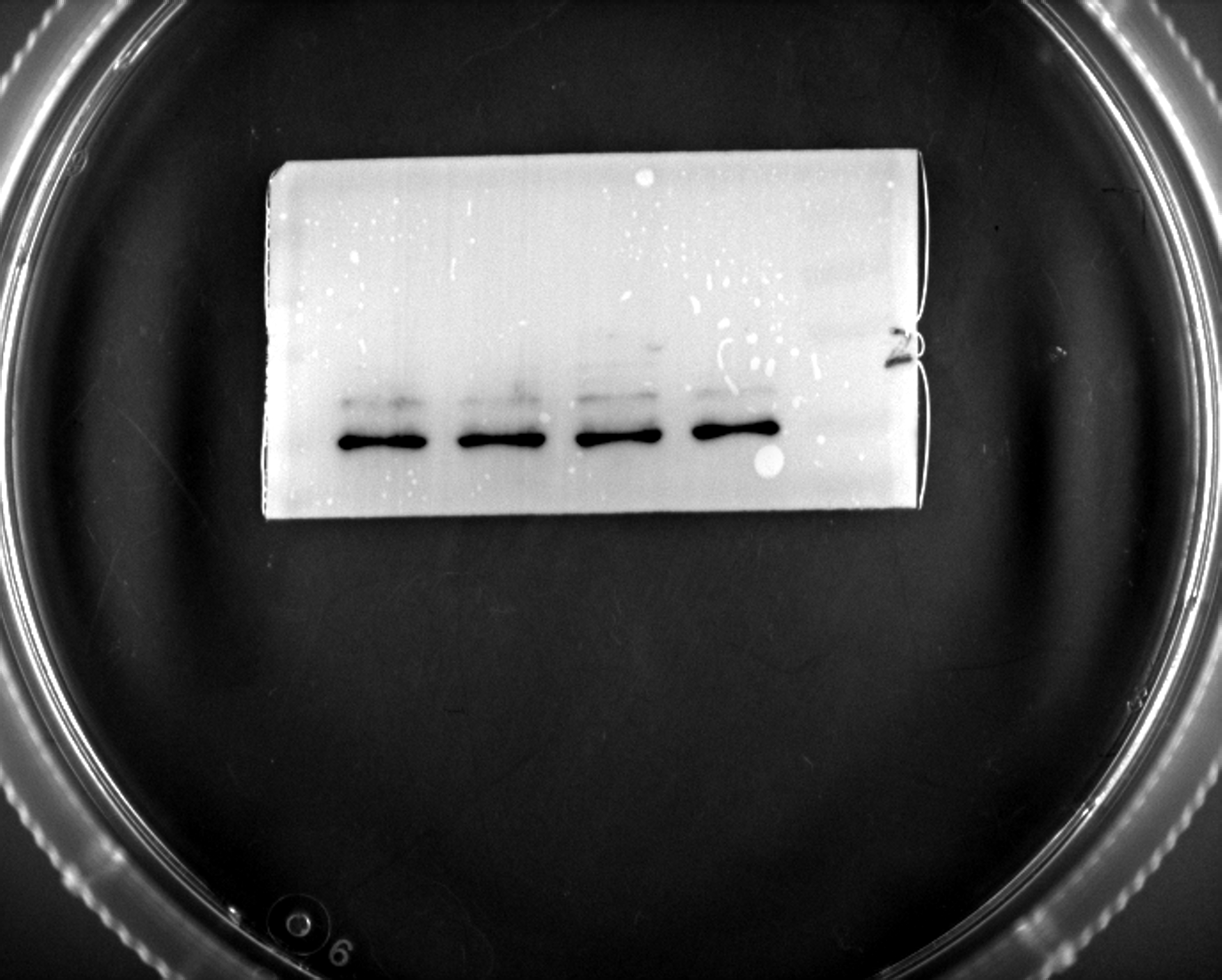

Supplement: Supplemental Information 8 [file peerj-13-19276-s008.zip › C I-R AAV9-CON AAV9-EB1 group western blot-Polymeric tubulin/1-VDAC-M.Tif]

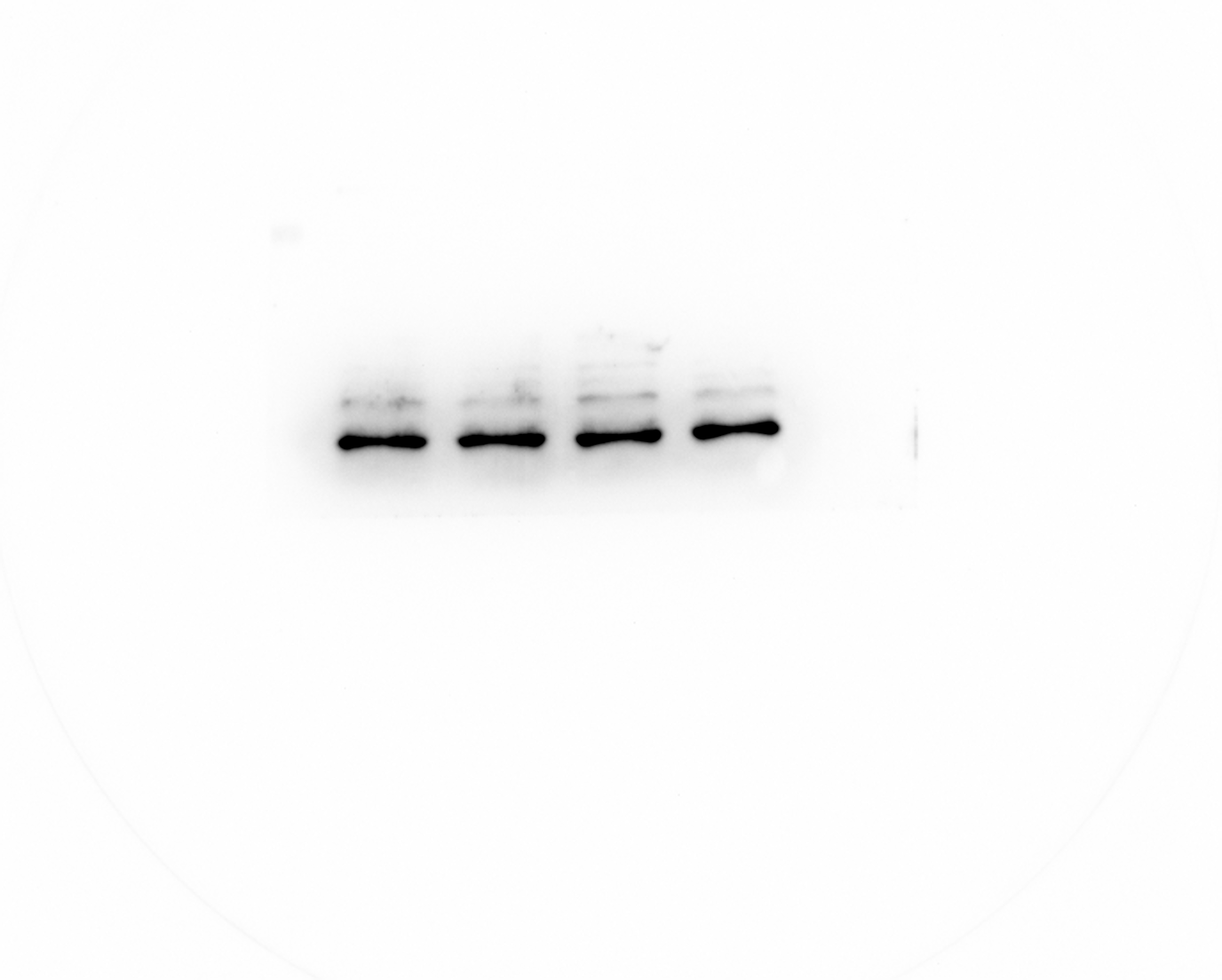

Supplement: Supplemental Information 8 [file peerj-13-19276-s008.zip › C I-R AAV9-CON AAV9-EB1 group western blot-Polymeric tubulin/1-VDAC.Tif]

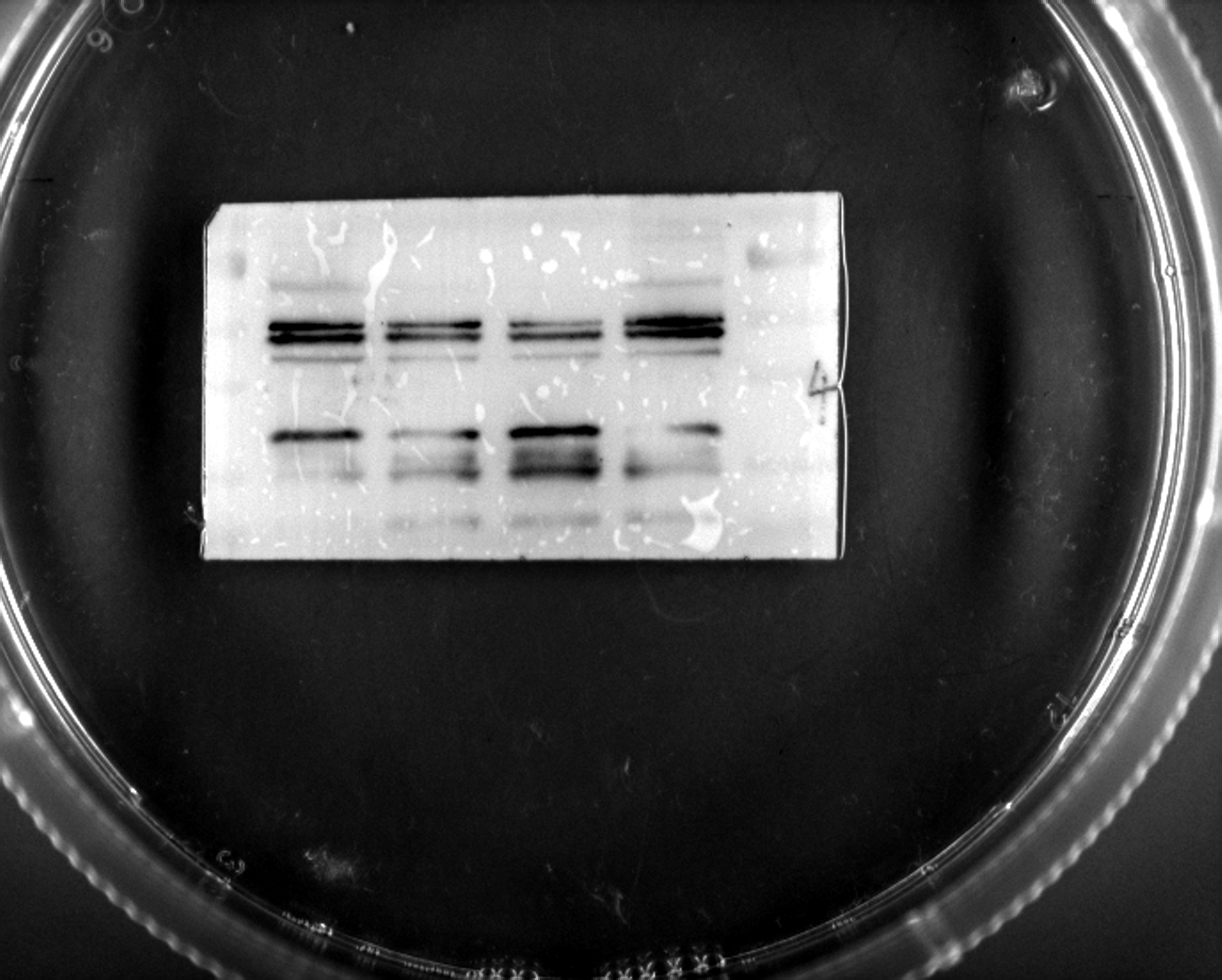

Supplement: Supplemental Information 8 [file peerj-13-19276-s008.zip › C I-R AAV9-CON AAV9-EB1 group western blot-Polymeric tubulin/2-Polymeric tubulin-M.Tif]

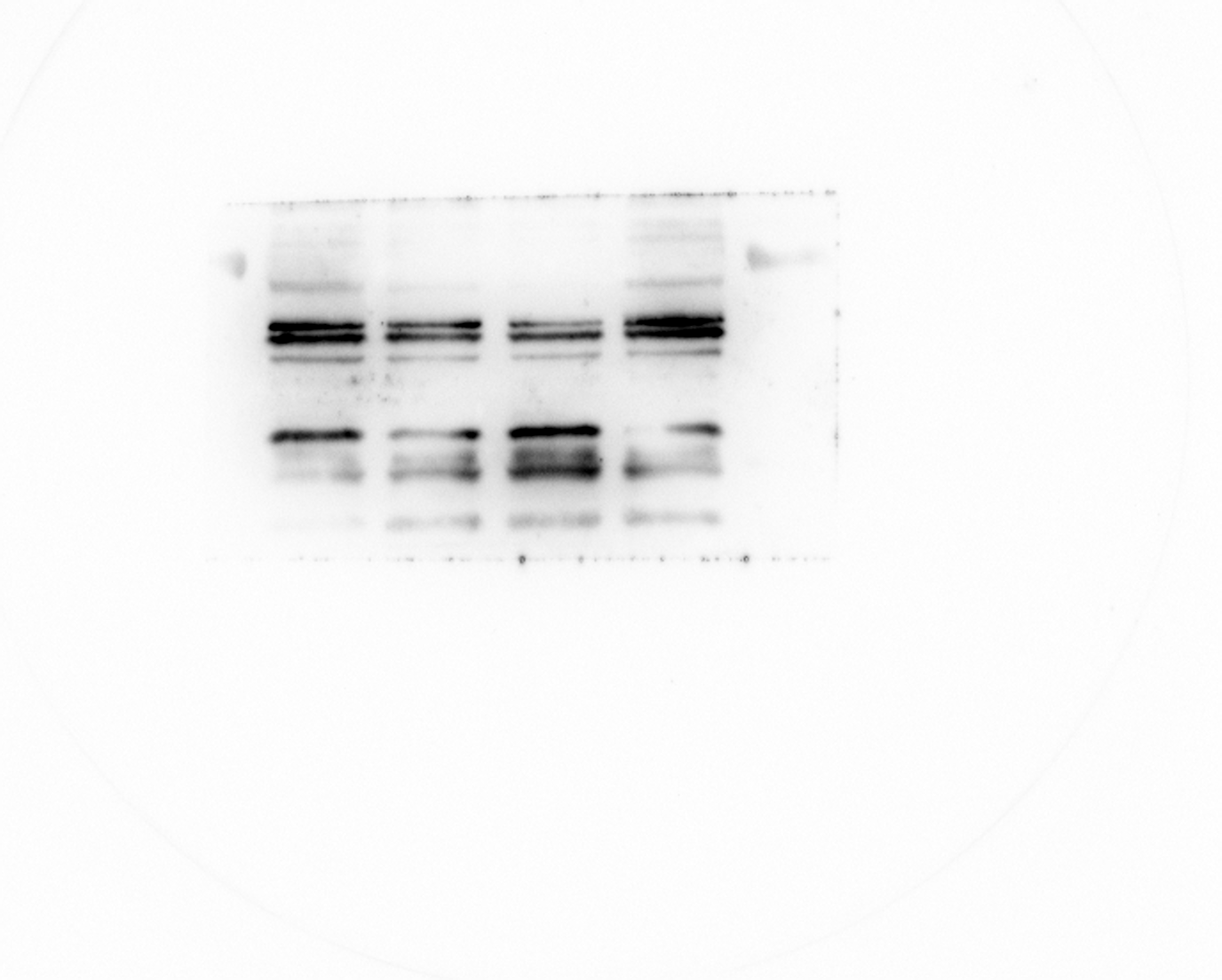

Supplement: Supplemental Information 8 [file peerj-13-19276-s008.zip › C I-R AAV9-CON AAV9-EB1 group western blot-Polymeric tubulin/2-Polymeric tubulin.Tif]

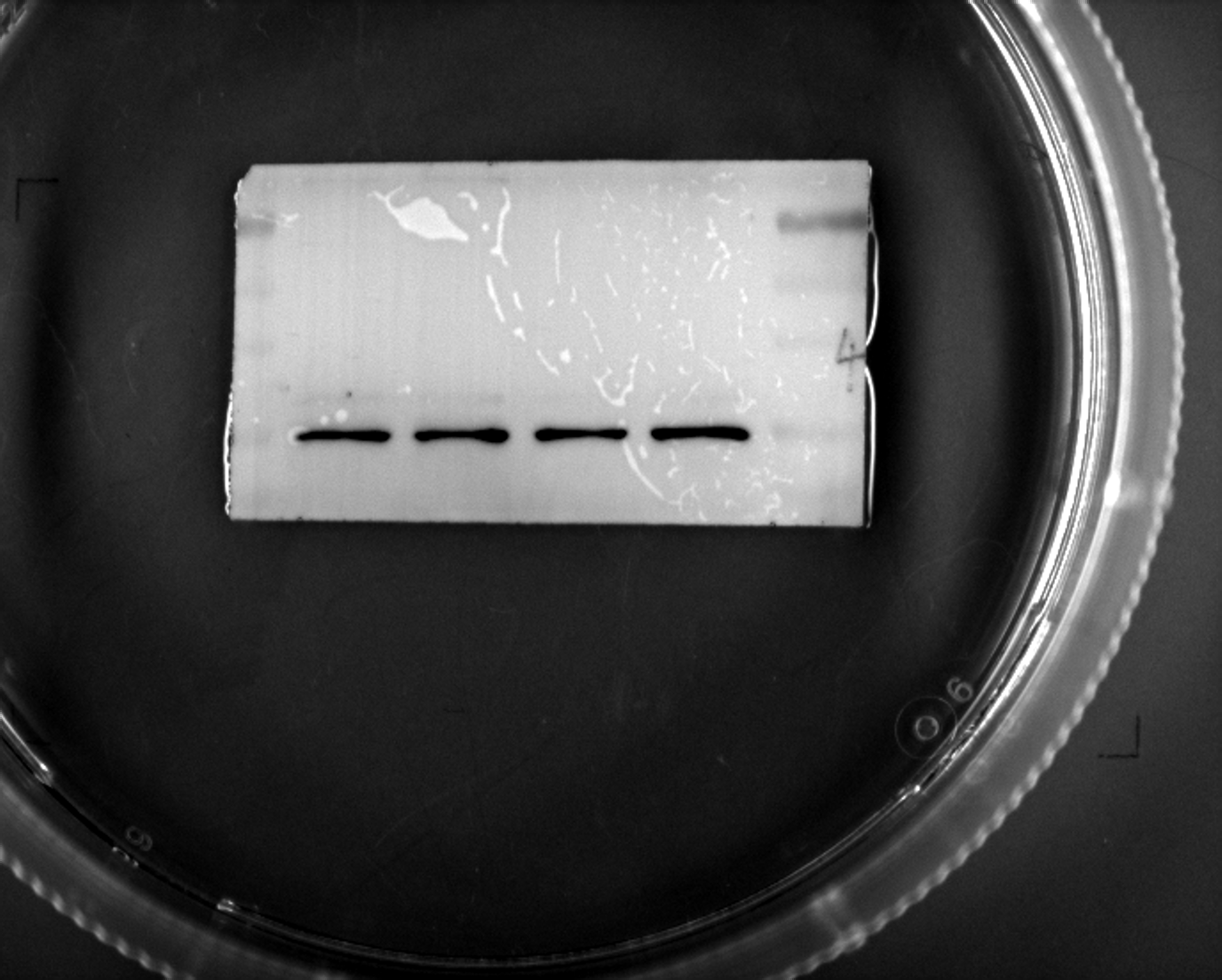

Supplement: Supplemental Information 8 [file peerj-13-19276-s008.zip › C I-R AAV9-CON AAV9-EB1 group western blot-Polymeric tubulin/2-VDAC-M.Tif]

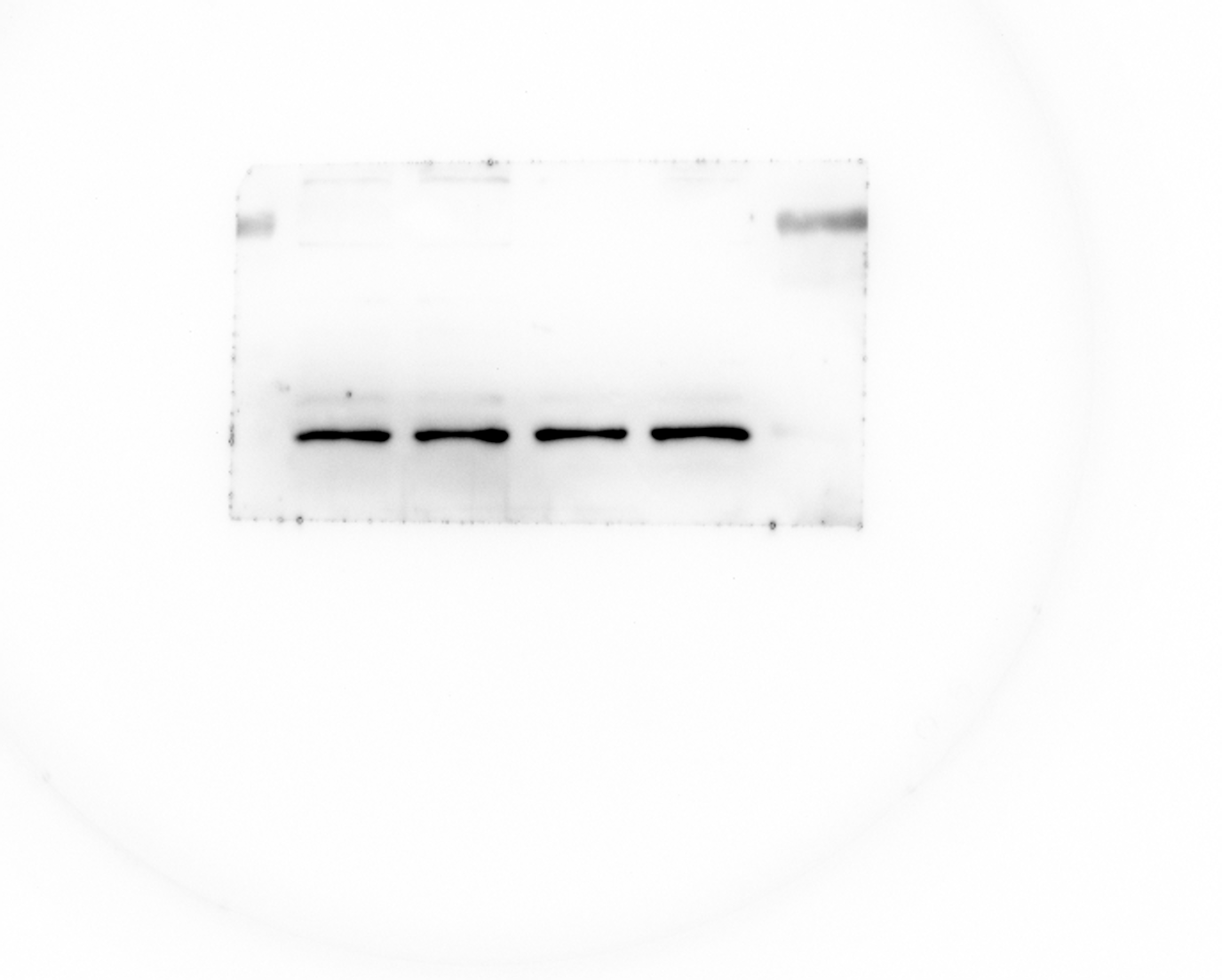

Supplement: Supplemental Information 8 [file peerj-13-19276-s008.zip › C I-R AAV9-CON AAV9-EB1 group western blot-Polymeric tubulin/2-VDAC.Tif]

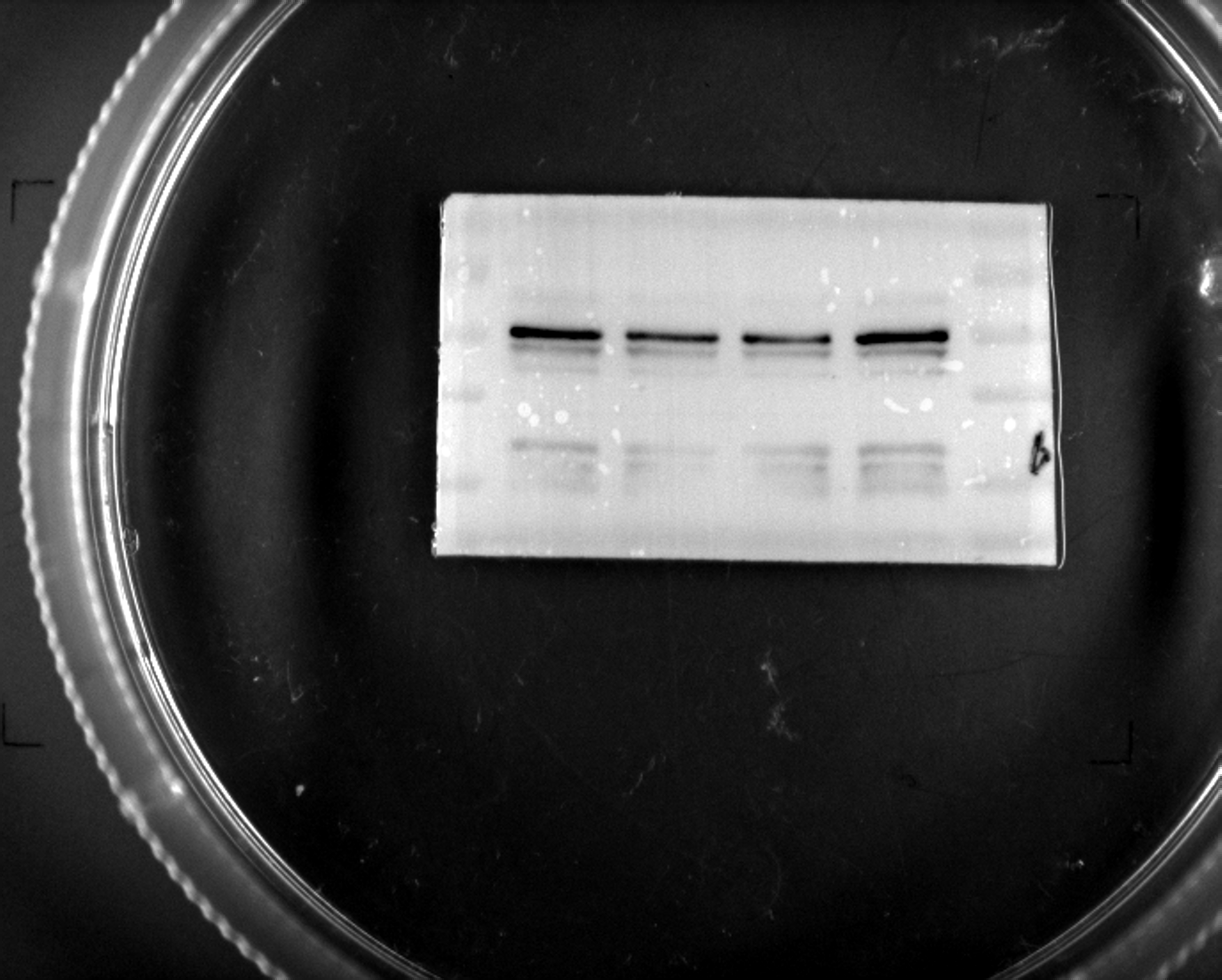

Supplement: Supplemental Information 8 [file peerj-13-19276-s008.zip › C I-R AAV9-CON AAV9-EB1 group western blot-Polymeric tubulin/3-Polymeric tubulin-M-used.Tif]

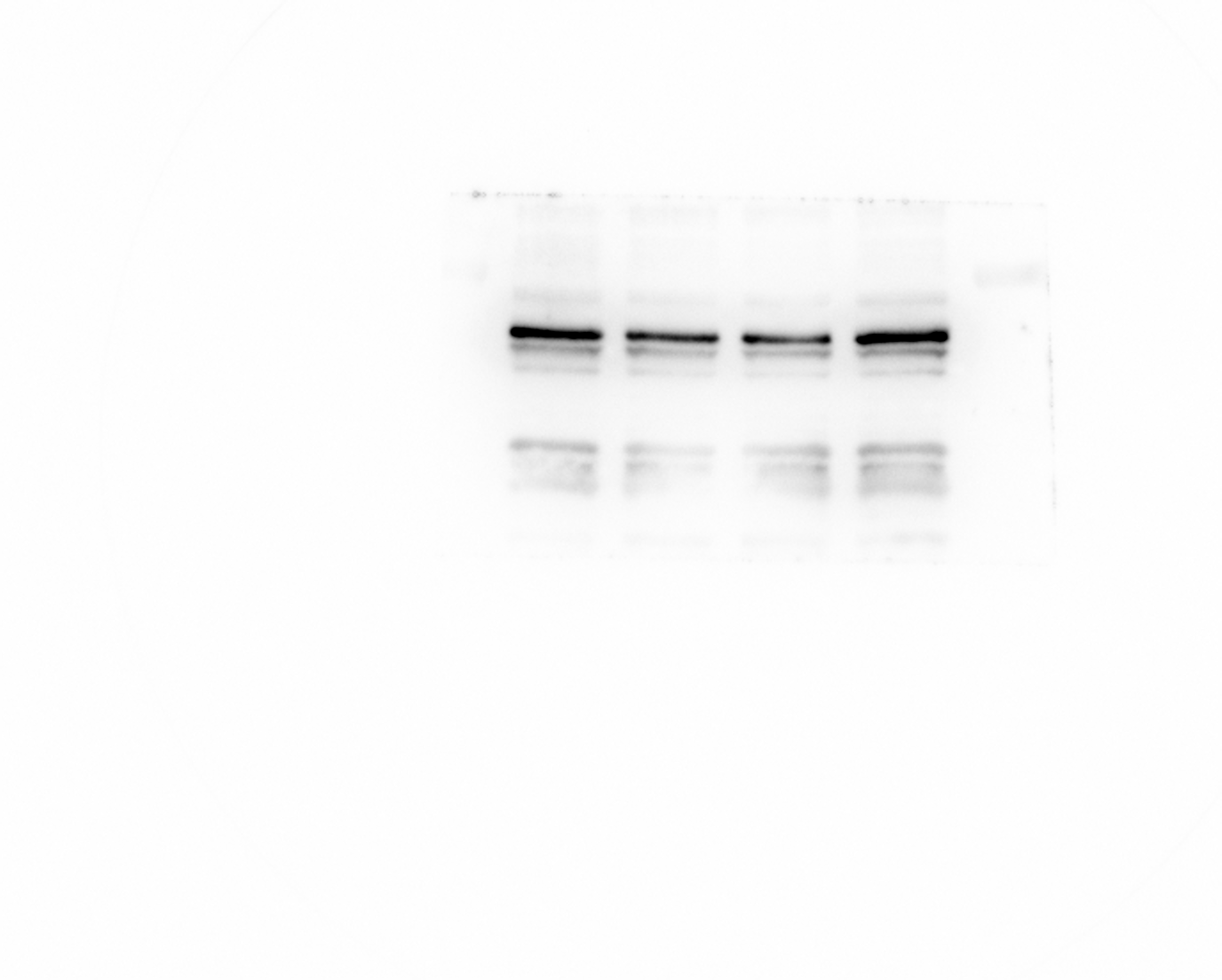

Supplement: Supplemental Information 8 [file peerj-13-19276-s008.zip › C I-R AAV9-CON AAV9-EB1 group western blot-Polymeric tubulin/3-Polymeric tubulin-used.Tif]

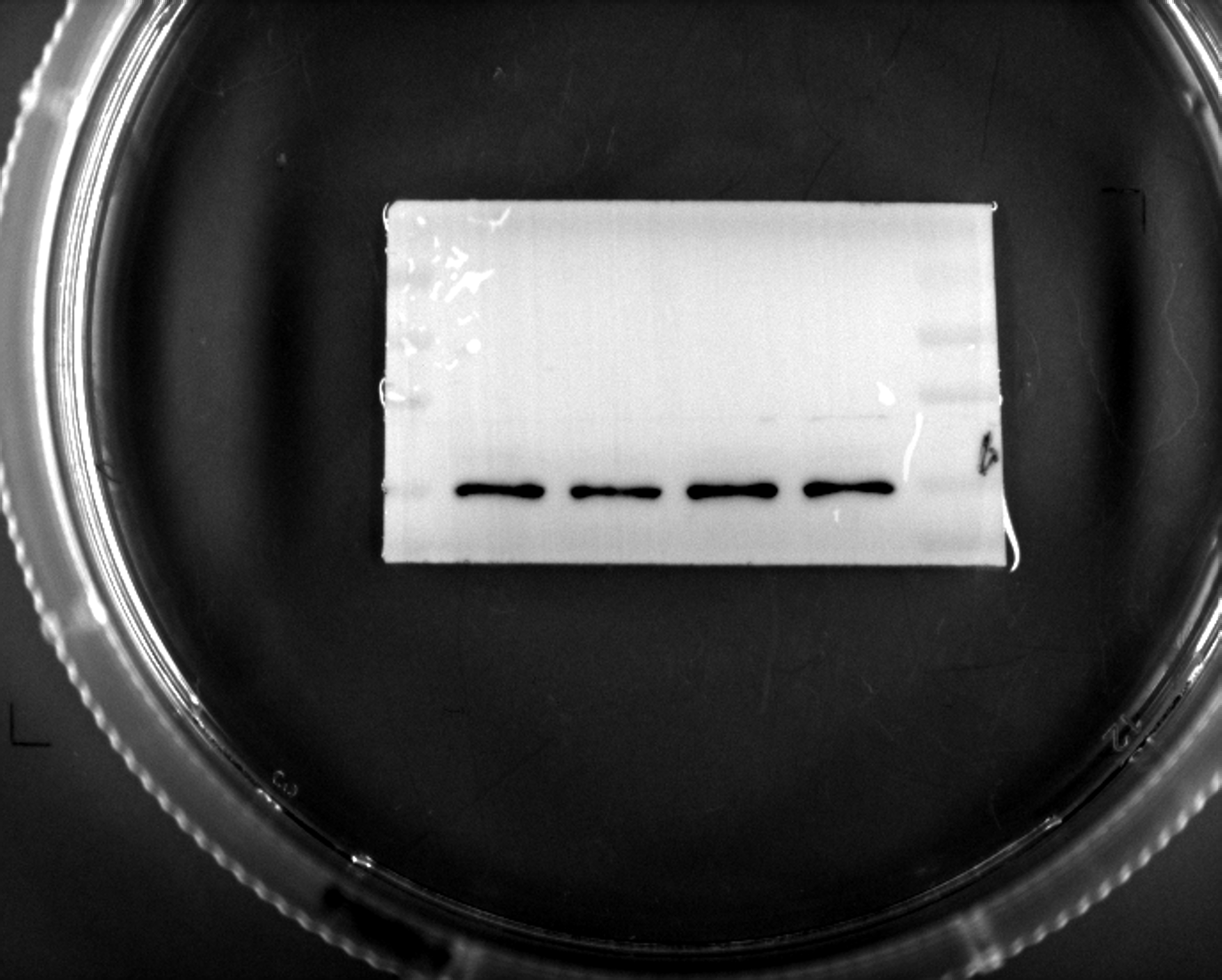

Supplement: Supplemental Information 8 [file peerj-13-19276-s008.zip › C I-R AAV9-CON AAV9-EB1 group western blot-Polymeric tubulin/3-VDAC-M-used.Tif]

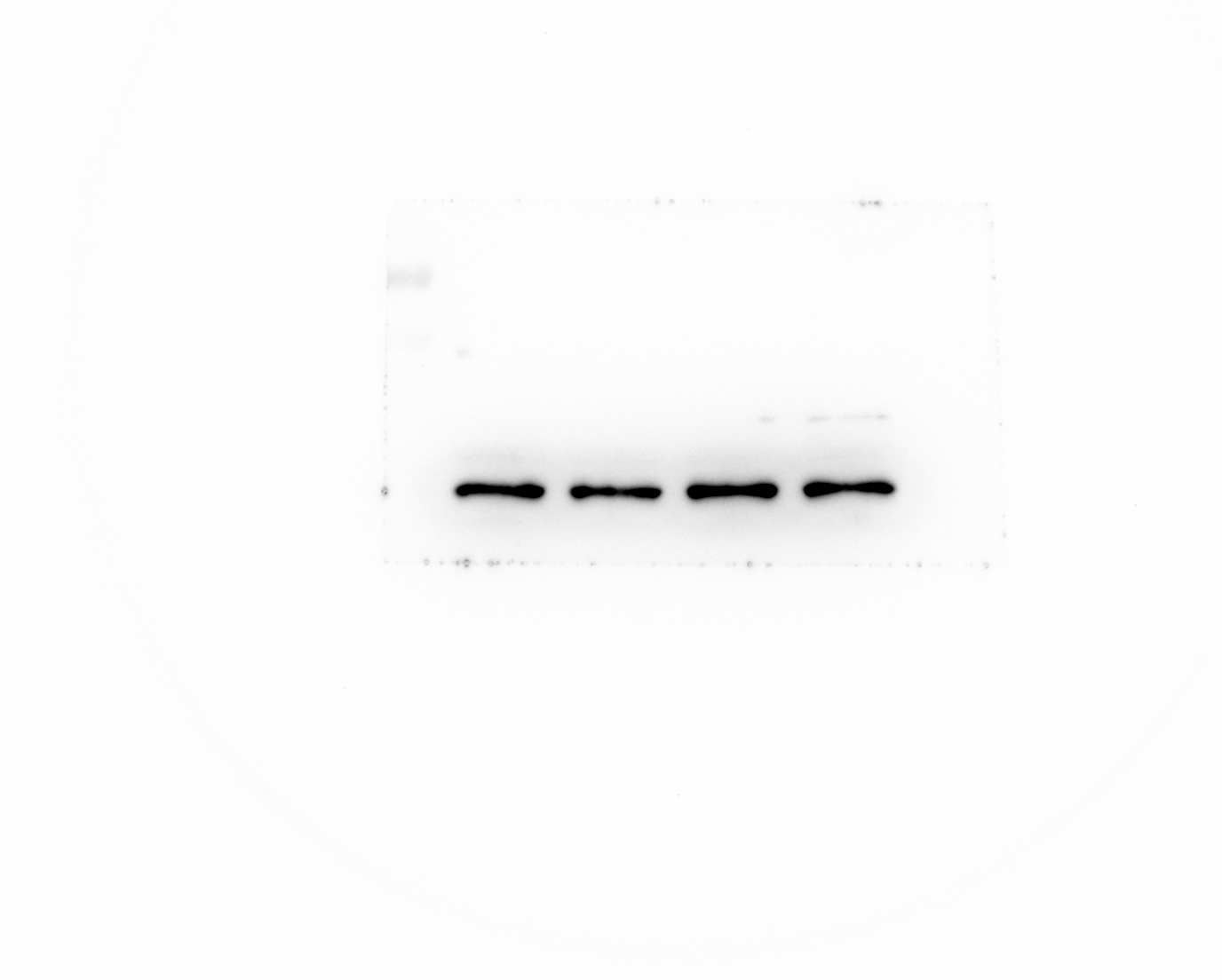

Supplement: Supplemental Information 8 [file peerj-13-19276-s008.zip › C I-R AAV9-CON AAV9-EB1 group western blot-Polymeric tubulin/3-VDAC-used.Tif]

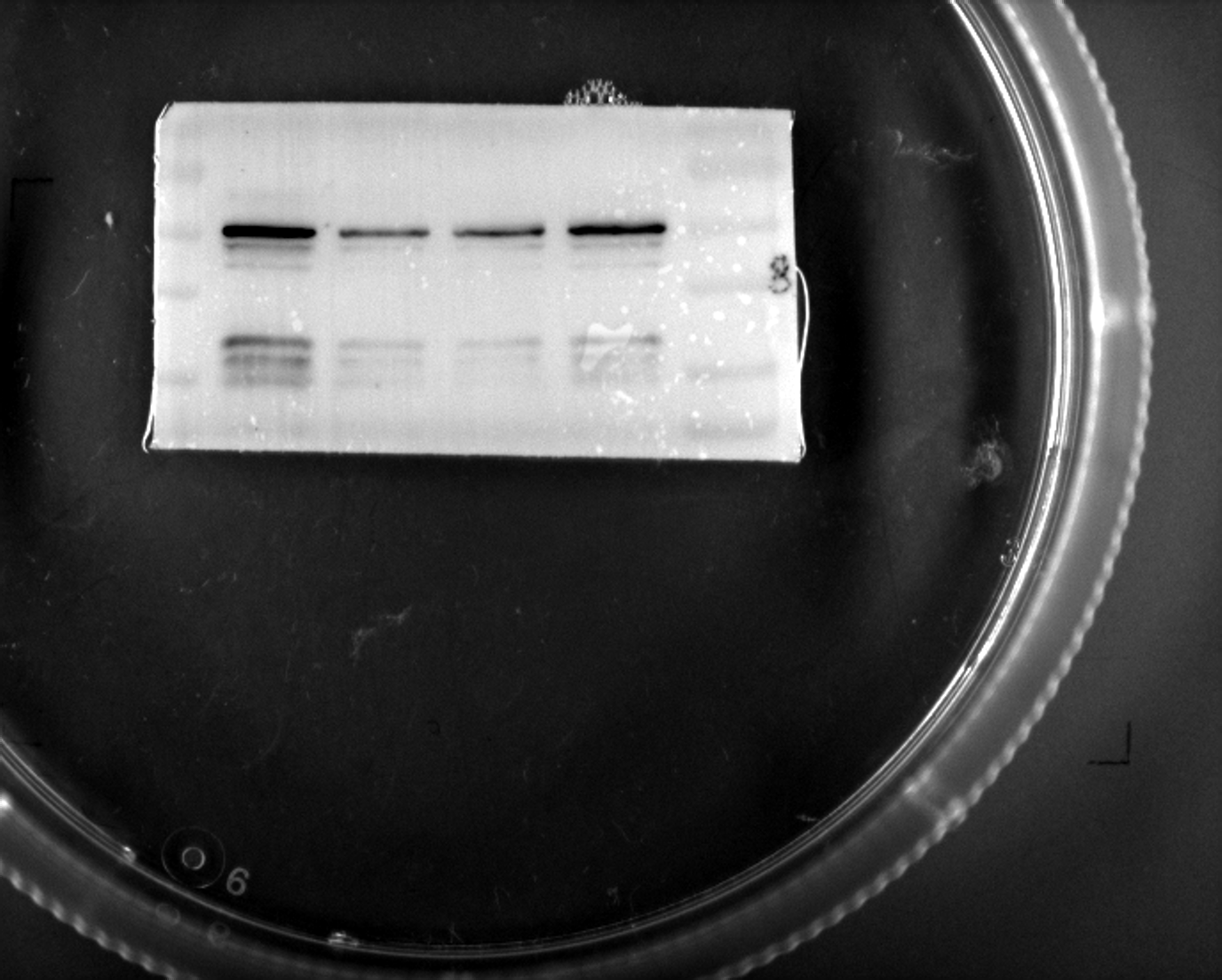

Supplement: Supplemental Information 8 [file peerj-13-19276-s008.zip › C I-R AAV9-CON AAV9-EB1 group western blot-Polymeric tubulin/4-Polymeric tubulin-M.Tif]

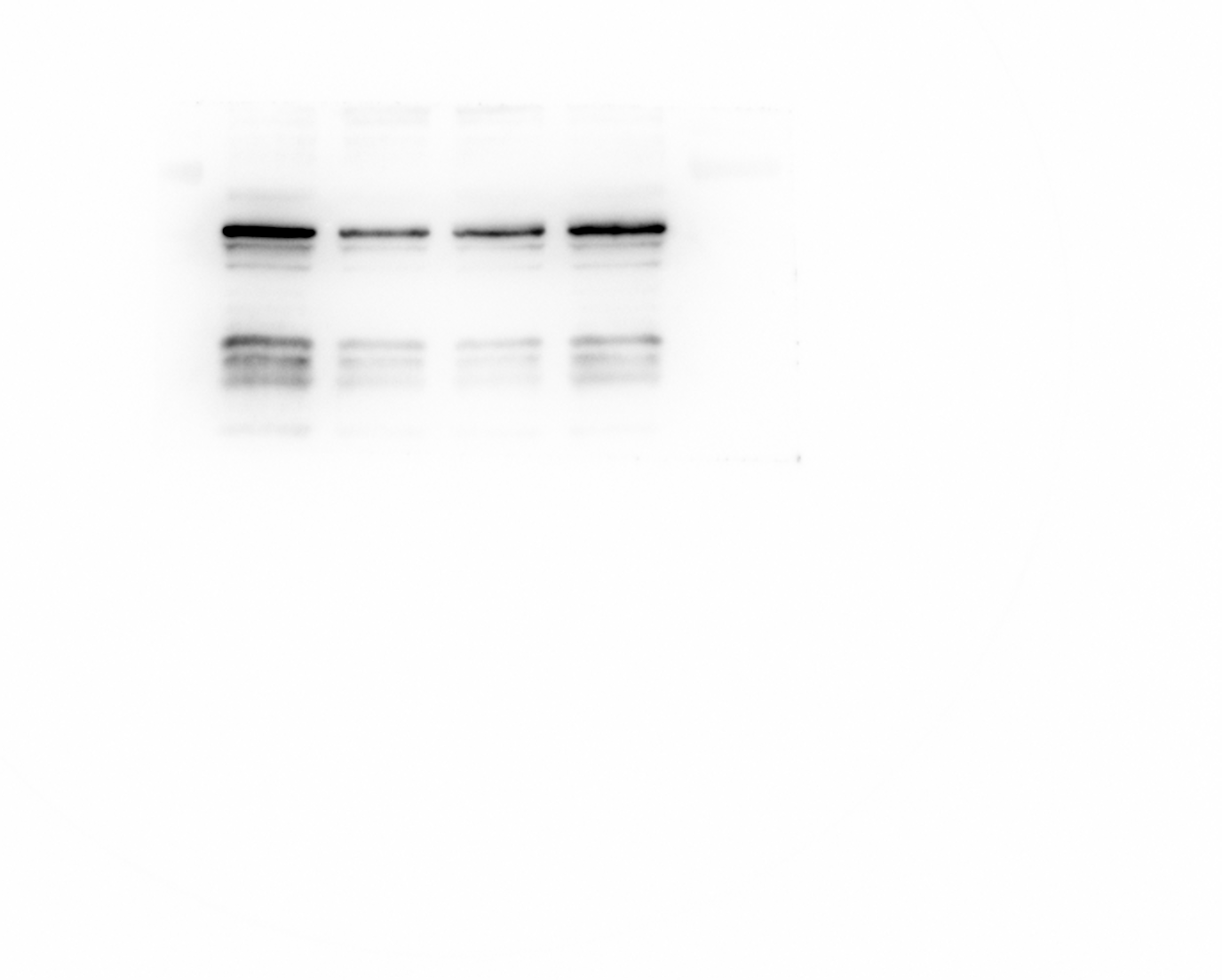

Supplement: Supplemental Information 8 [file peerj-13-19276-s008.zip › C I-R AAV9-CON AAV9-EB1 group western blot-Polymeric tubulin/4-Polymeric tubulin.Tif]

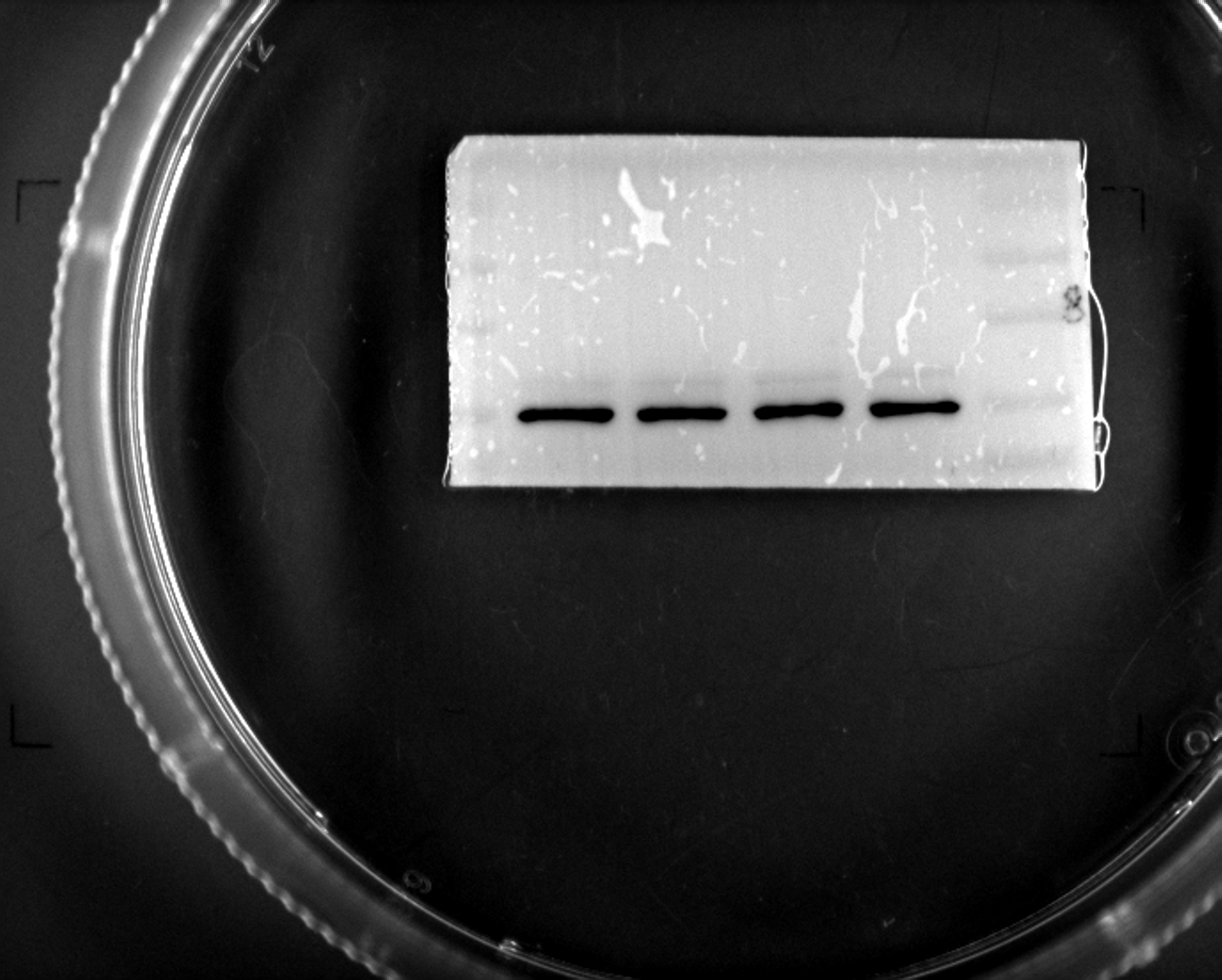

Supplement: Supplemental Information 8 [file peerj-13-19276-s008.zip › C I-R AAV9-CON AAV9-EB1 group western blot-Polymeric tubulin/4-VDAC-M.Tif]

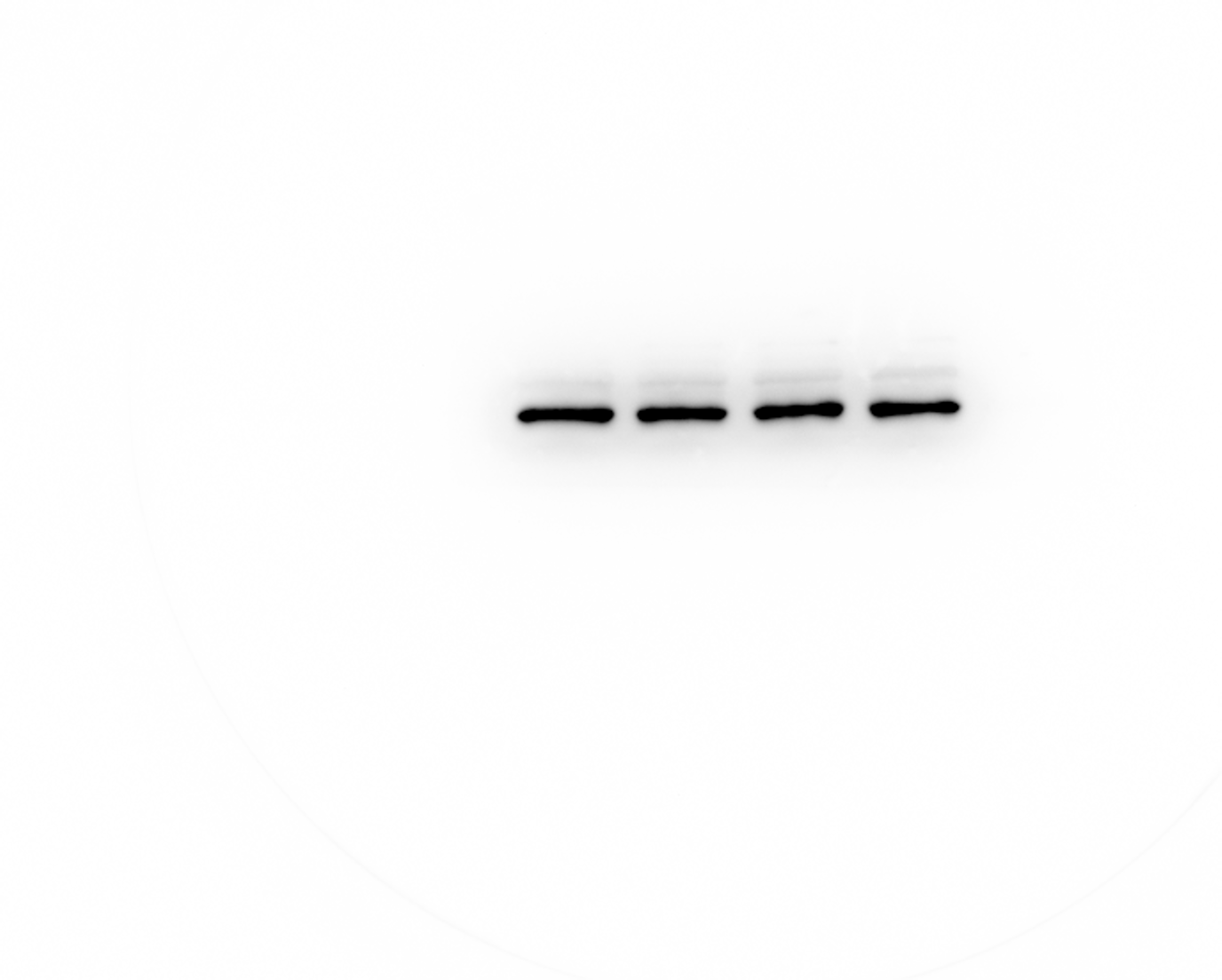

Supplement: Supplemental Information 8 [file peerj-13-19276-s008.zip › C I-R AAV9-CON AAV9-EB1 group western blot-Polymeric tubulin/4-VDAC.Tif]

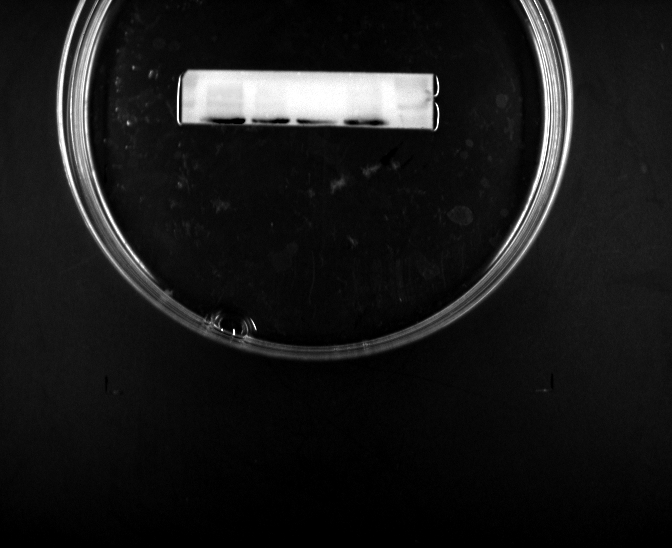

Supplement: Supplemental Information 8 [file peerj-13-19276-s008.zip › C I-R AAV9-CON AAV9-EB1 group western blot-Polymeric tubulin/5-Polymeric tubulin-M.Tif]

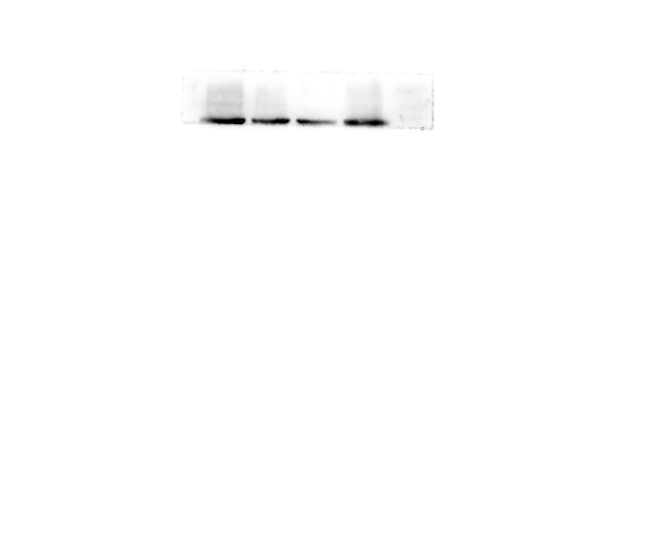

Supplement: Supplemental Information 8 [file peerj-13-19276-s008.zip › C I-R AAV9-CON AAV9-EB1 group western blot-Polymeric tubulin/5-Polymeric tubulin.Tif]

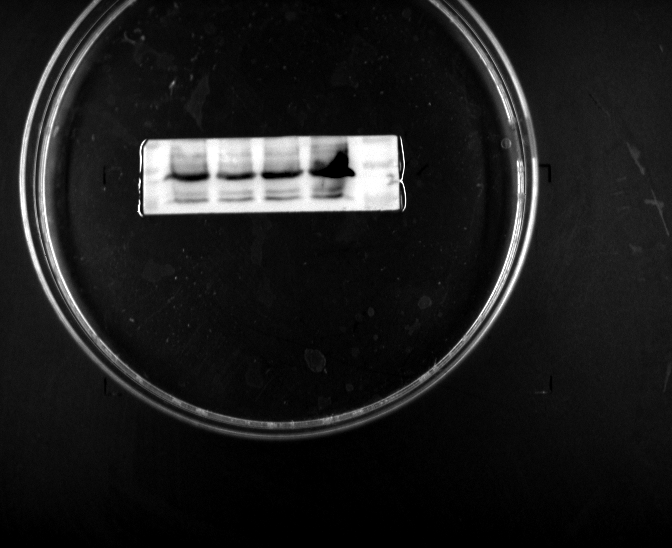

Supplement: Supplemental Information 8 [file peerj-13-19276-s008.zip › C I-R AAV9-CON AAV9-EB1 group western blot-Polymeric tubulin/5-VDAC-M.Tif]

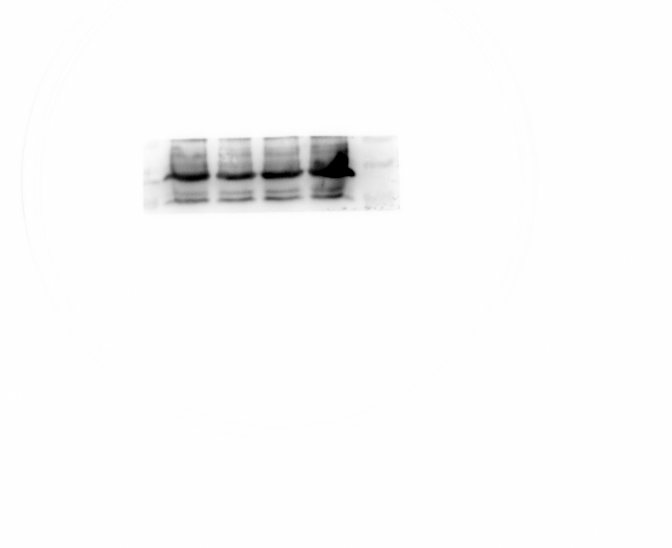

Supplement: Supplemental Information 8 [file peerj-13-19276-s008.zip › C I-R AAV9-CON AAV9-EB1 group western blot-Polymeric tubulin/5-VDAC.Tif]

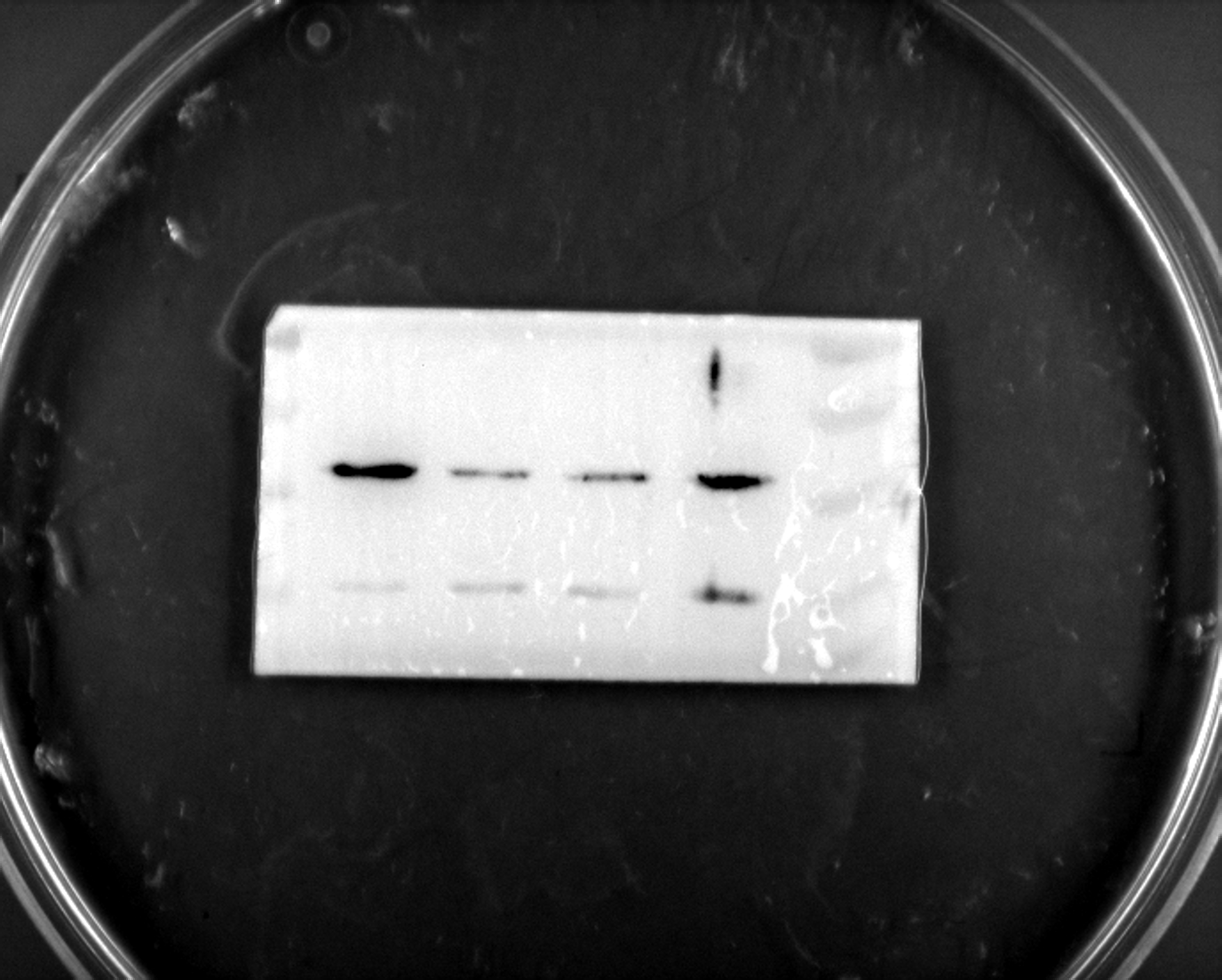

Supplement: Supplemental Information 8 [file peerj-13-19276-s008.zip › C I-R AAV9-CON AAV9-EB1 group western blot-Polymeric tubulin/6-Polymeric tubulin-M.Tif]

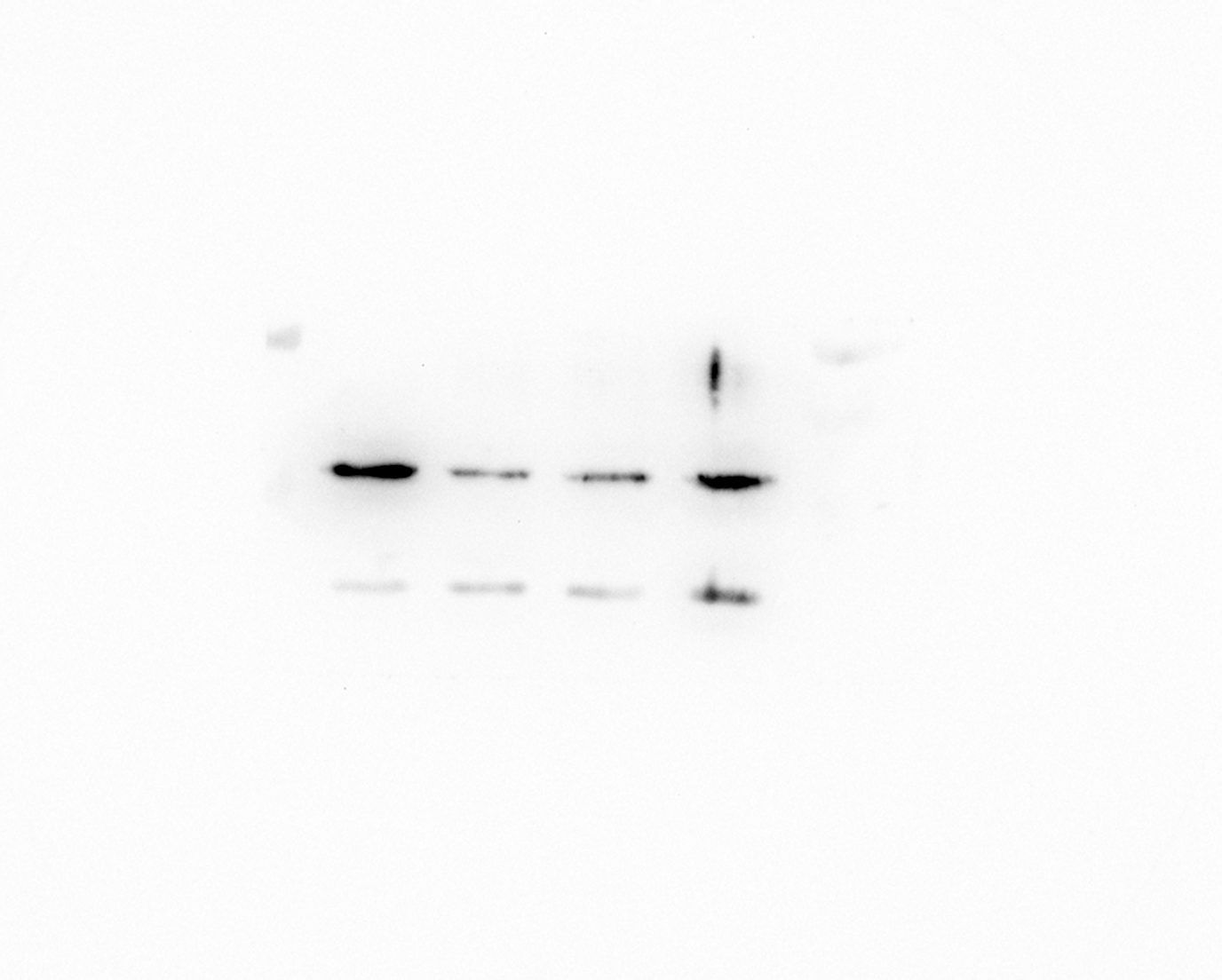

Supplement: Supplemental Information 8 [file peerj-13-19276-s008.zip › C I-R AAV9-CON AAV9-EB1 group western blot-Polymeric tubulin/6-Polymeric tubulin.Tif]

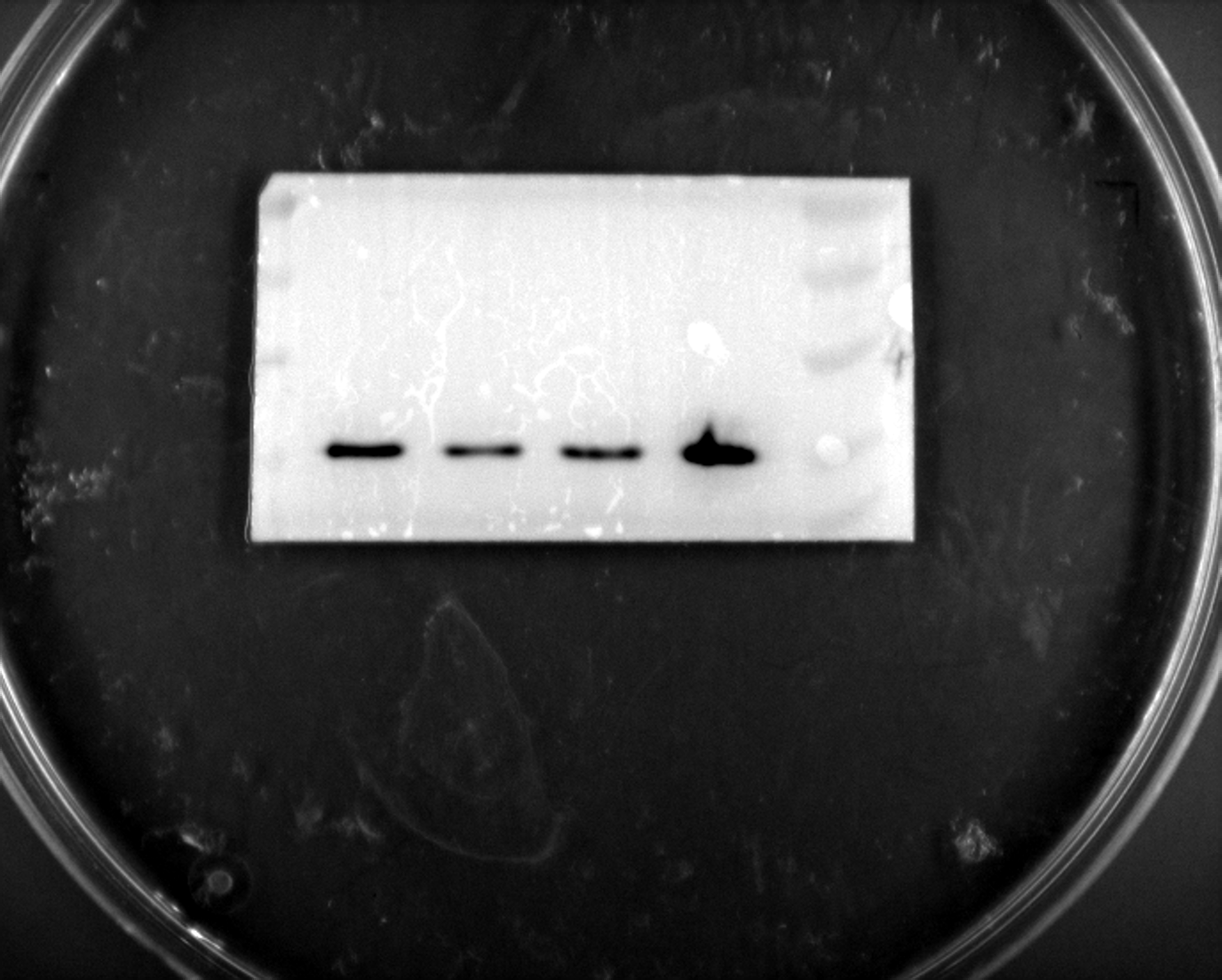

Supplement: Supplemental Information 8 [file peerj-13-19276-s008.zip › C I-R AAV9-CON AAV9-EB1 group western blot-Polymeric tubulin/6-VDAC-M.Tif]

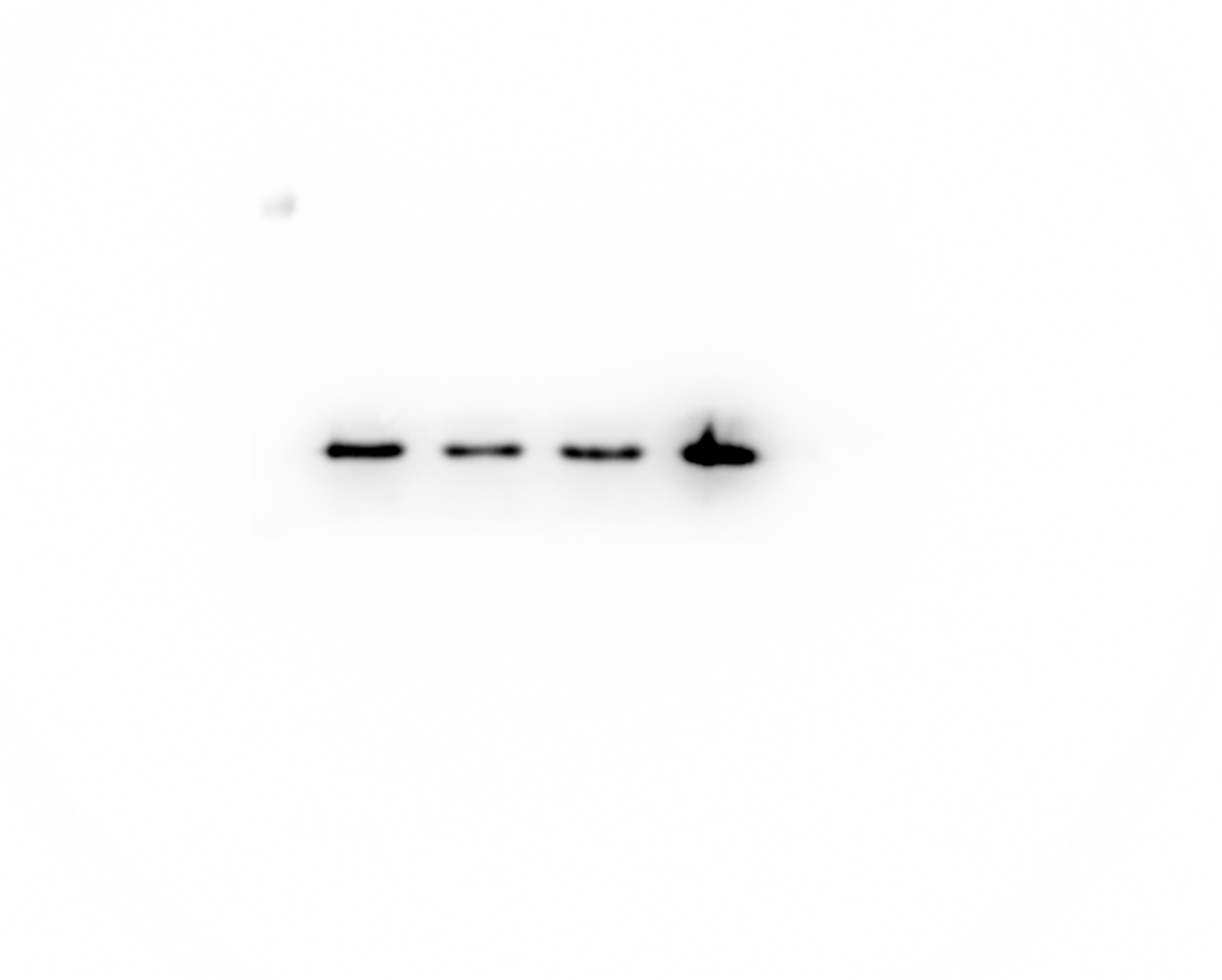

Supplement: Supplemental Information 8 [file peerj-13-19276-s008.zip › C I-R AAV9-CON AAV9-EB1 group western blot-Polymeric tubulin/6-VDAC.Tif]

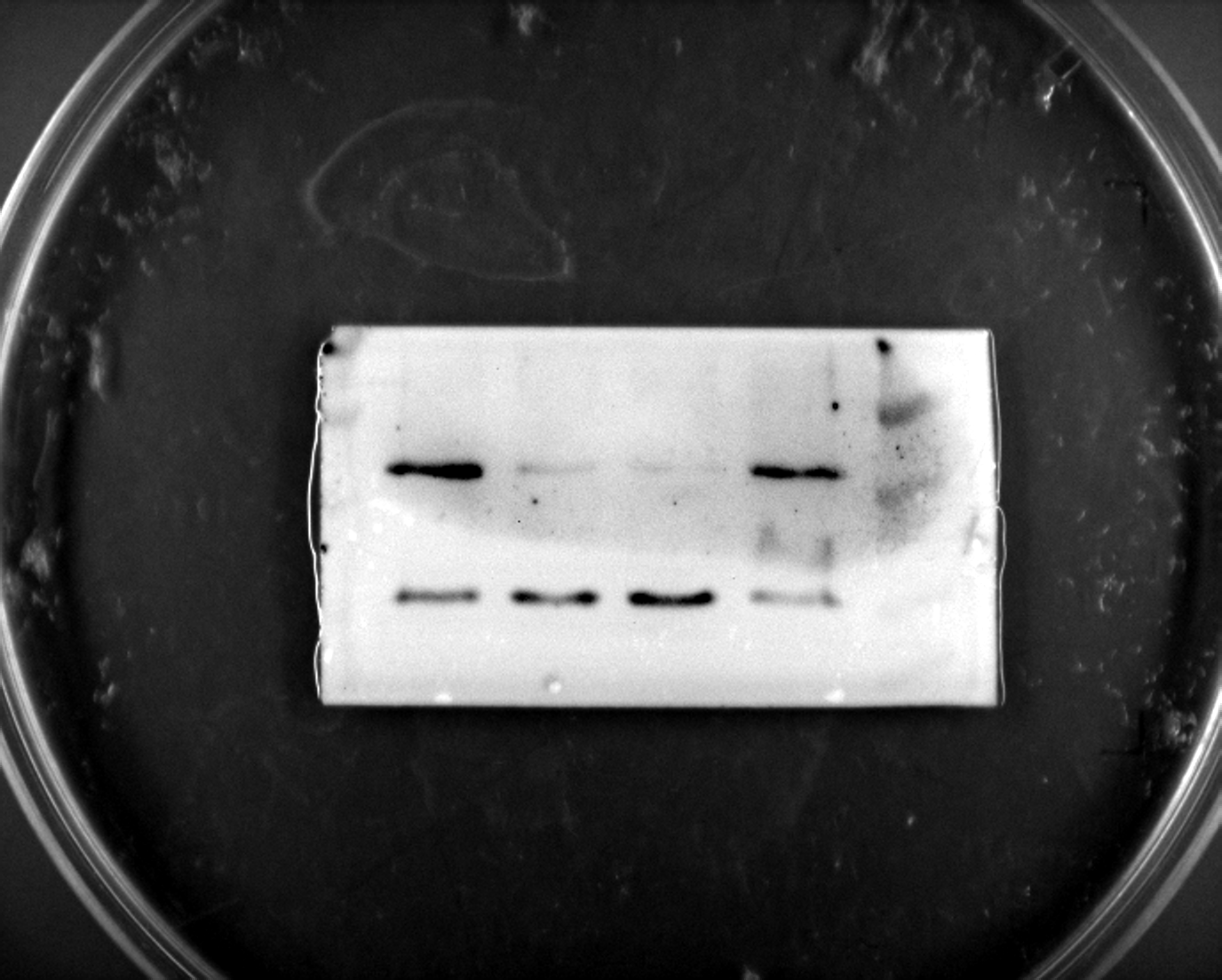

Supplement: Supplemental Information 8 [file peerj-13-19276-s008.zip › C I-R AAV9-CON AAV9-EB1 group western blot-Polymeric tubulin/7-Polymeric tubulin-M.Tif]

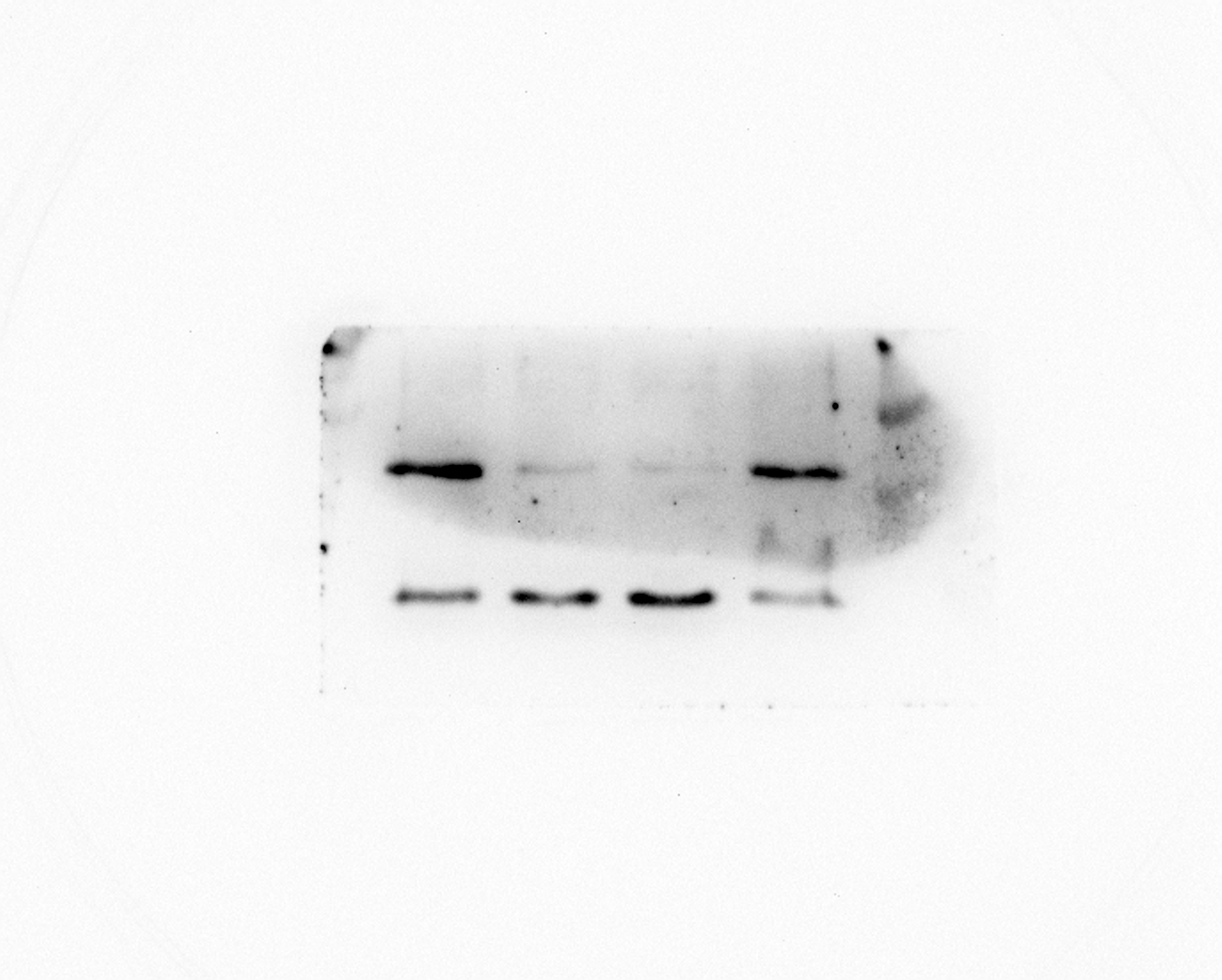

Supplement: Supplemental Information 8 [file peerj-13-19276-s008.zip › C I-R AAV9-CON AAV9-EB1 group western blot-Polymeric tubulin/7-Polymeric tubulin.Tif]

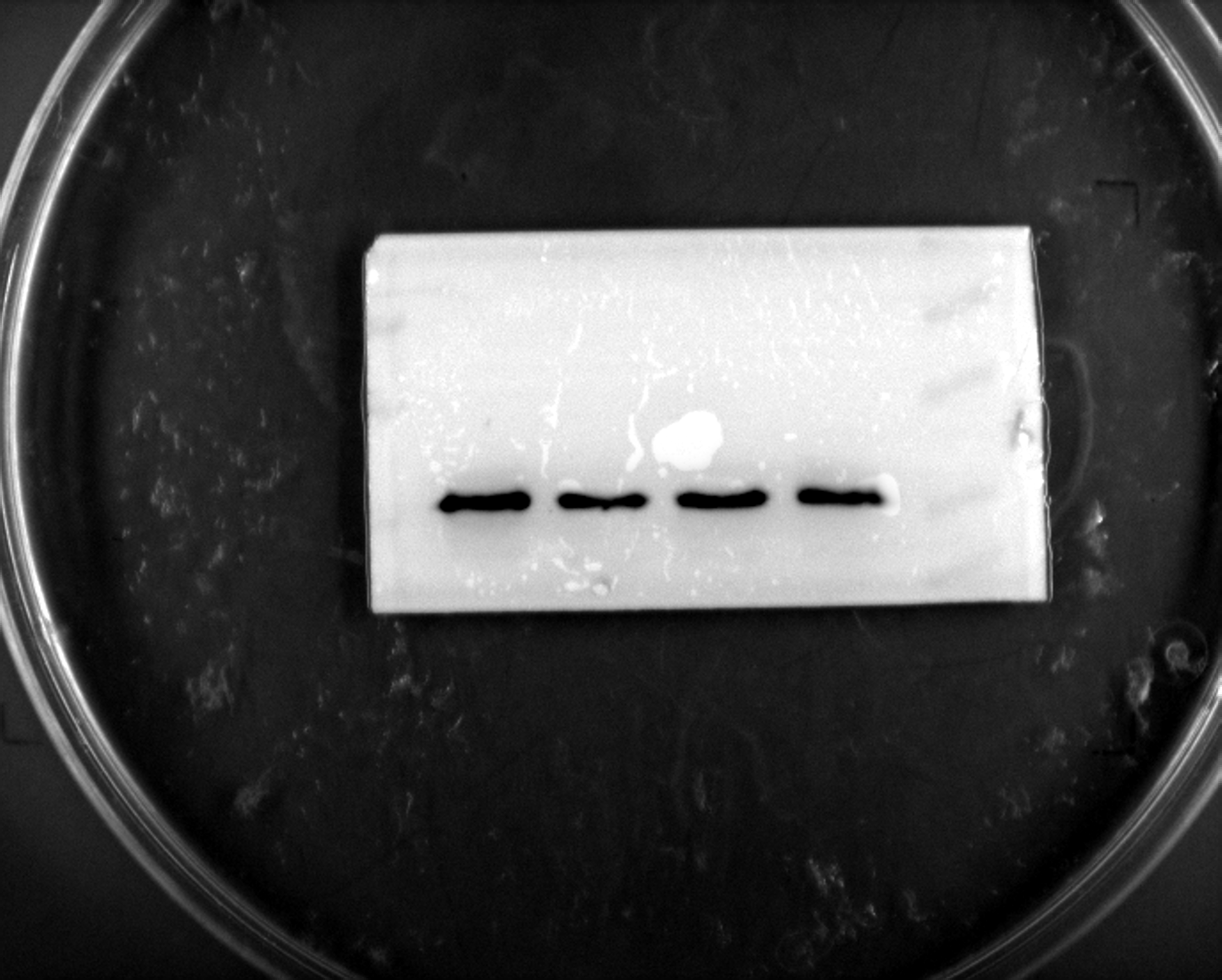

Supplement: Supplemental Information 8 [file peerj-13-19276-s008.zip › C I-R AAV9-CON AAV9-EB1 group western blot-Polymeric tubulin/7-VDAC-M.Tif]

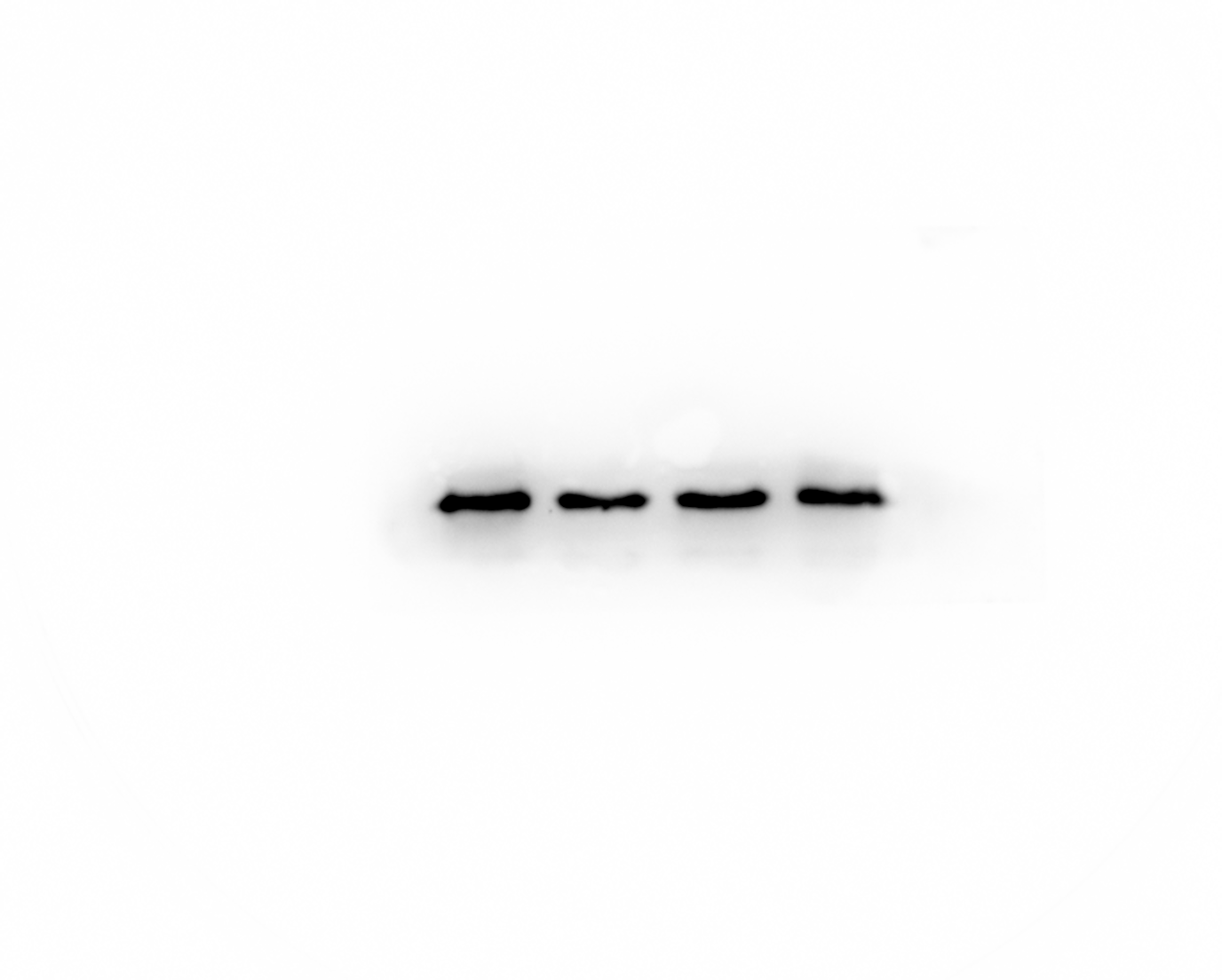

Supplement: Supplemental Information 8 [file peerj-13-19276-s008.zip › C I-R AAV9-CON AAV9-EB1 group western blot-Polymeric tubulin/7-VDAC.Tif]

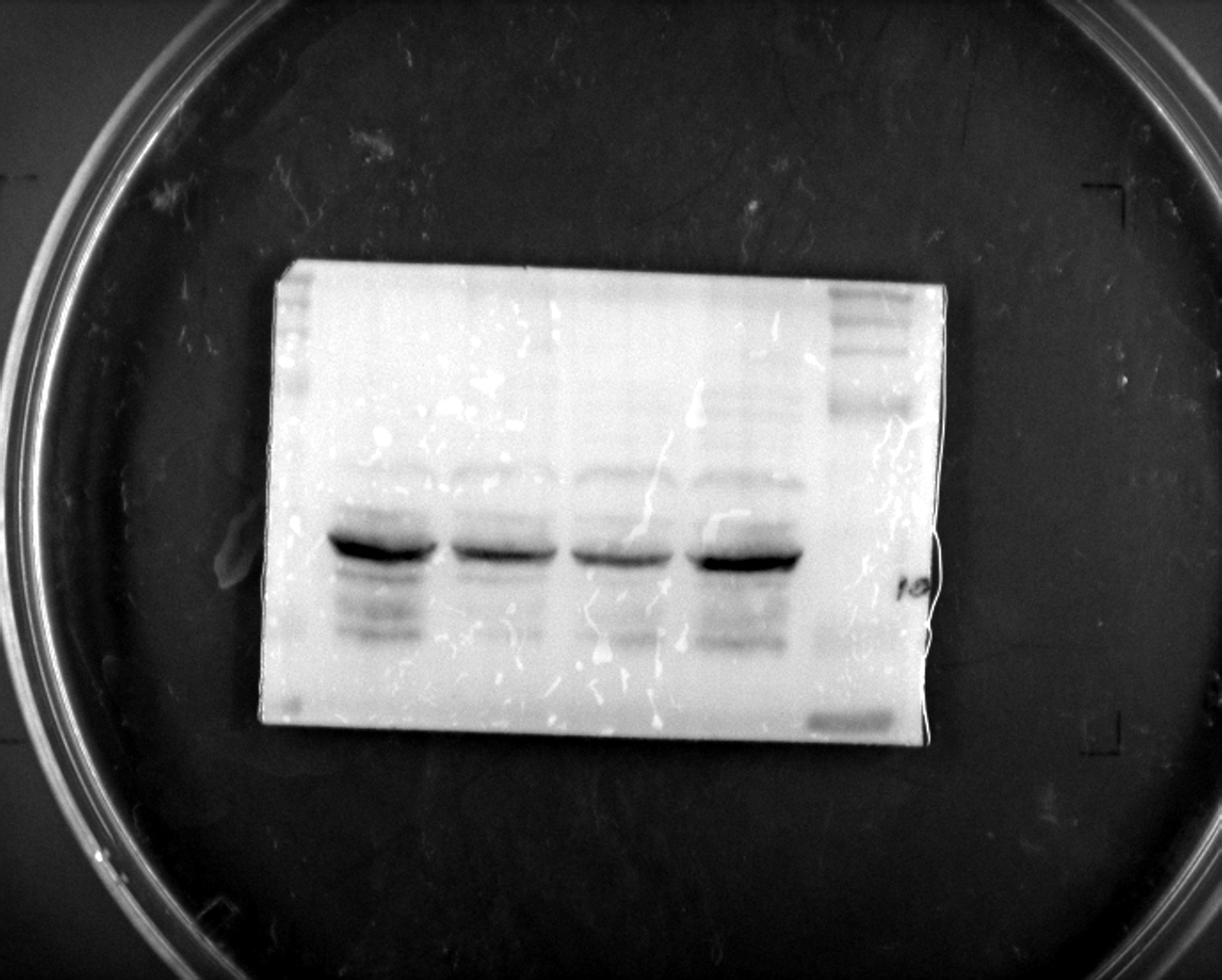

Supplement: Supplemental Information 9 [file peerj-13-19276-s009.zip › western blot-Total Cx43 EB1 N-cadherin 1/10-cx43-M-used.Tif]

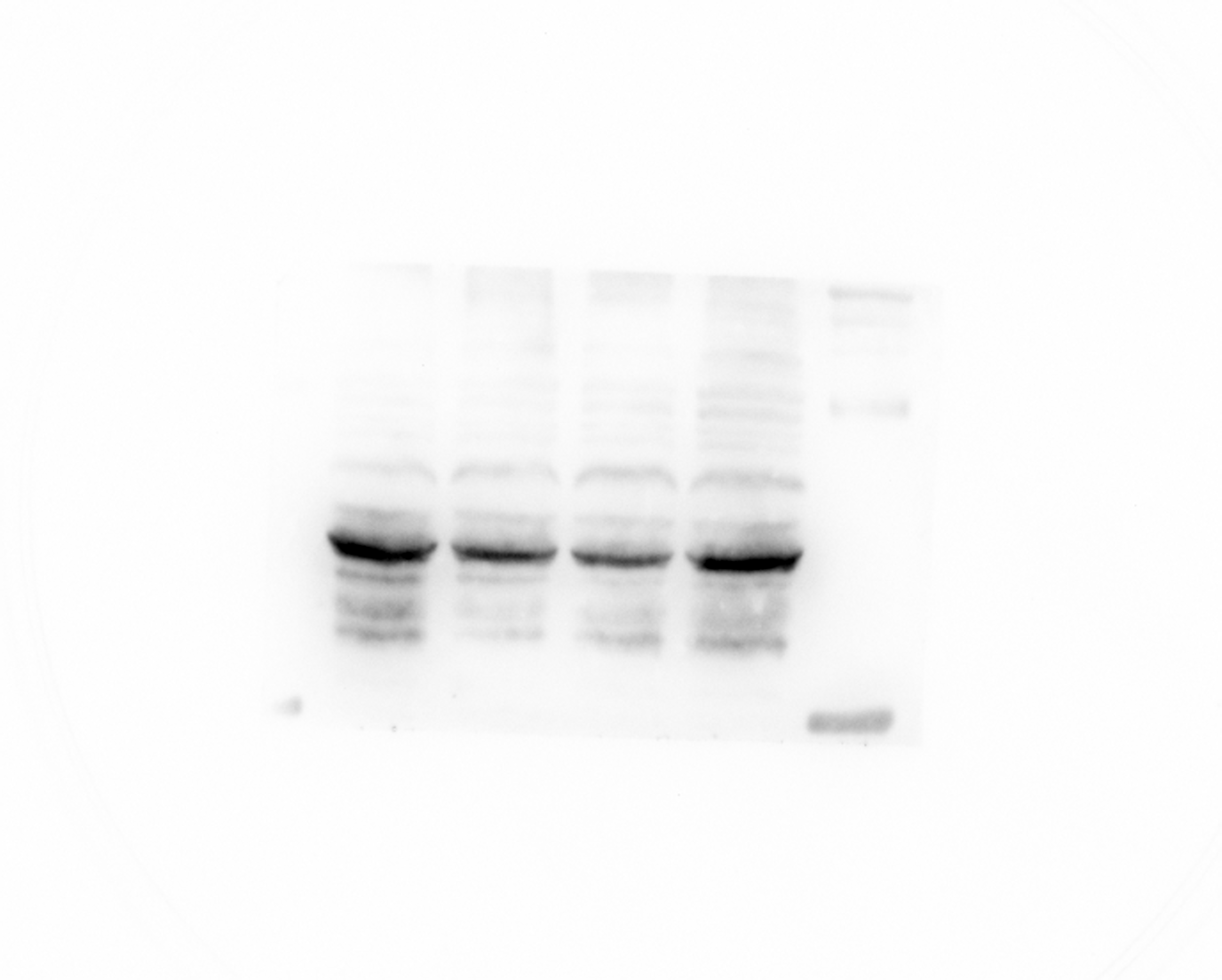

Supplement: Supplemental Information 9 [file peerj-13-19276-s009.zip › western blot-Total Cx43 EB1 N-cadherin 1/10-cx43-used.Tif]

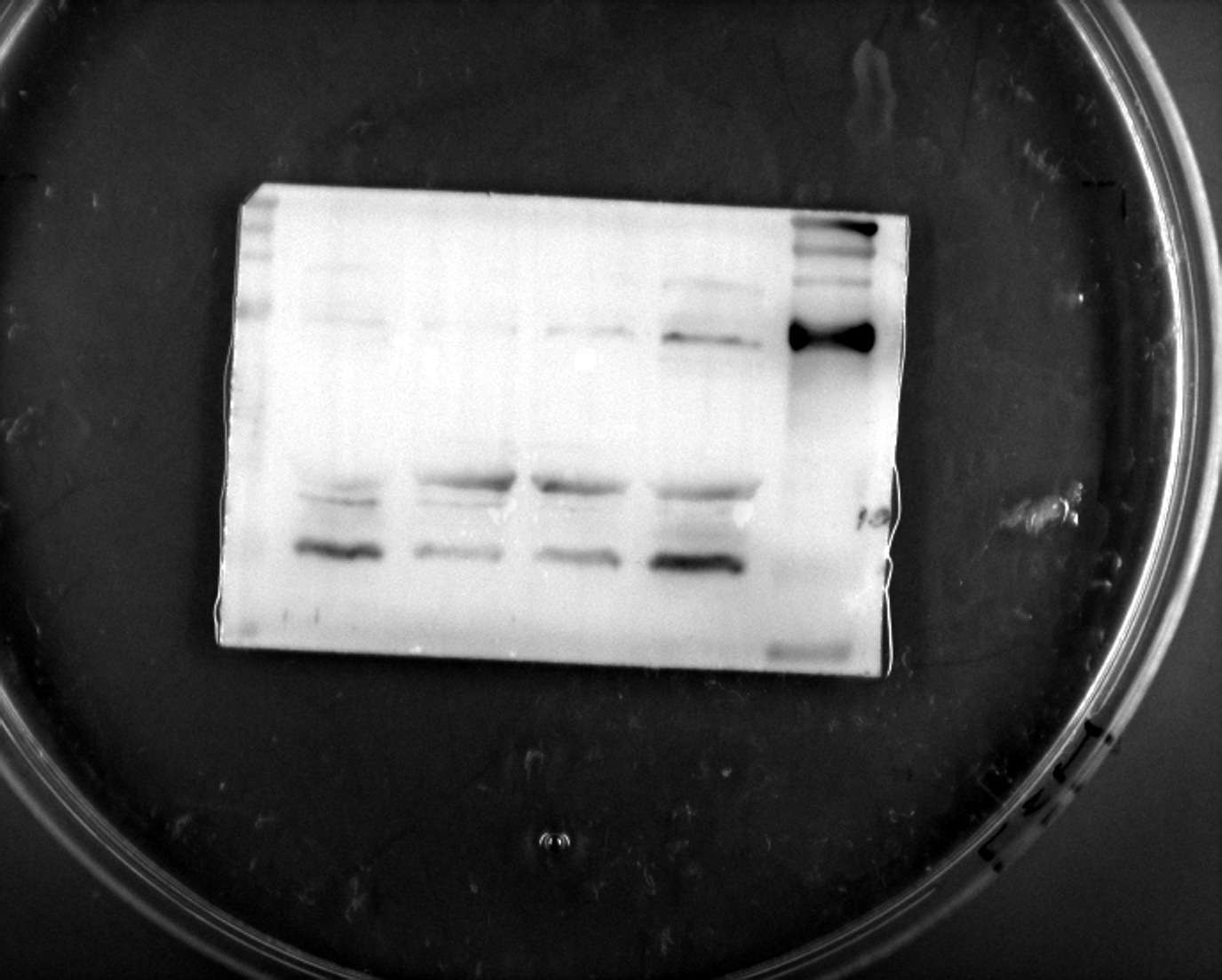

Supplement: Supplemental Information 9 [file peerj-13-19276-s009.zip › western blot-Total Cx43 EB1 N-cadherin 1/10-EB1-M-used.Tif]

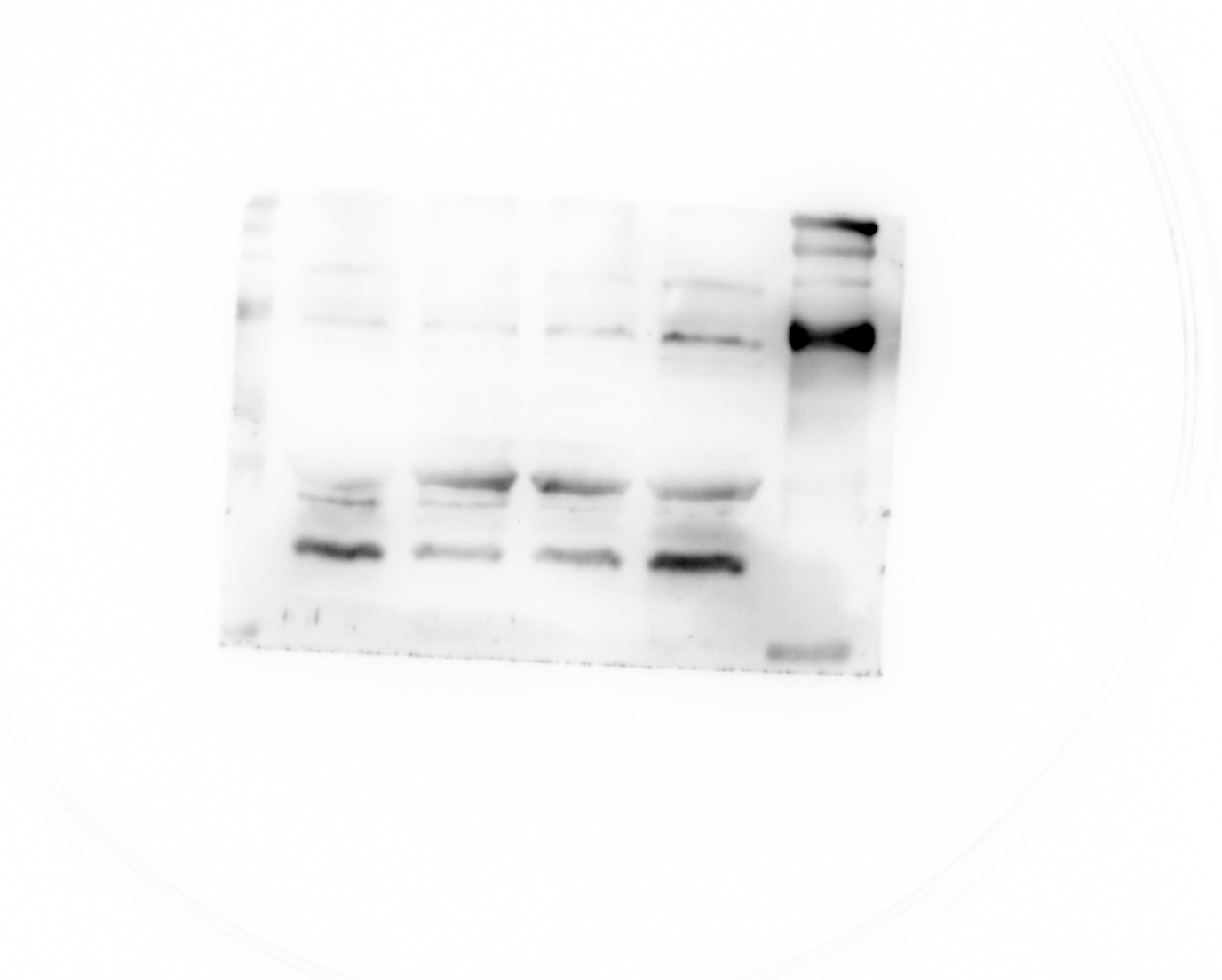

Supplement: Supplemental Information 9 [file peerj-13-19276-s009.zip › western blot-Total Cx43 EB1 N-cadherin 1/10-EB1-used.Tif]

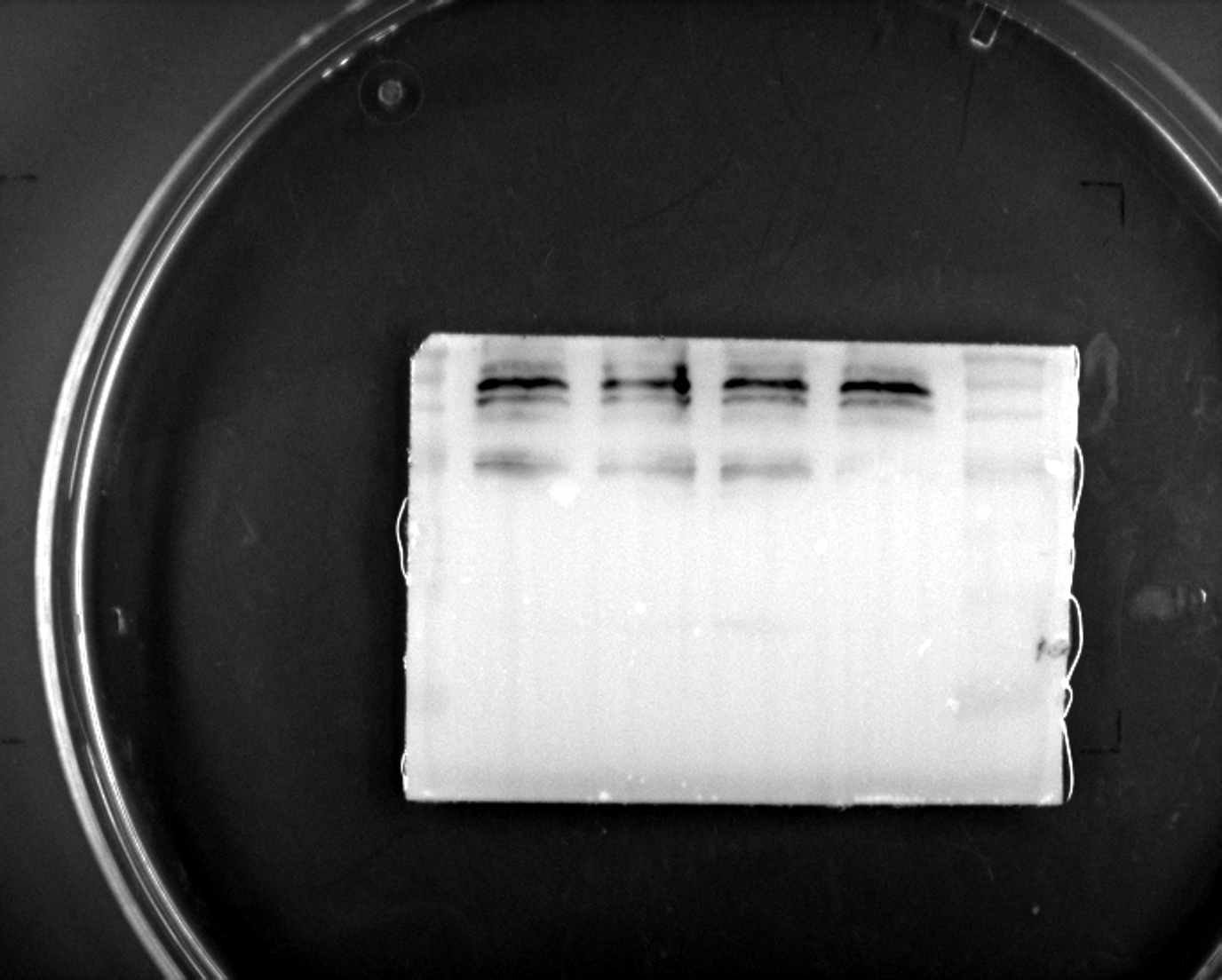

Supplement: Supplemental Information 9 [file peerj-13-19276-s009.zip › western blot-Total Cx43 EB1 N-cadherin 1/10-N-cadherin-M.Tif]

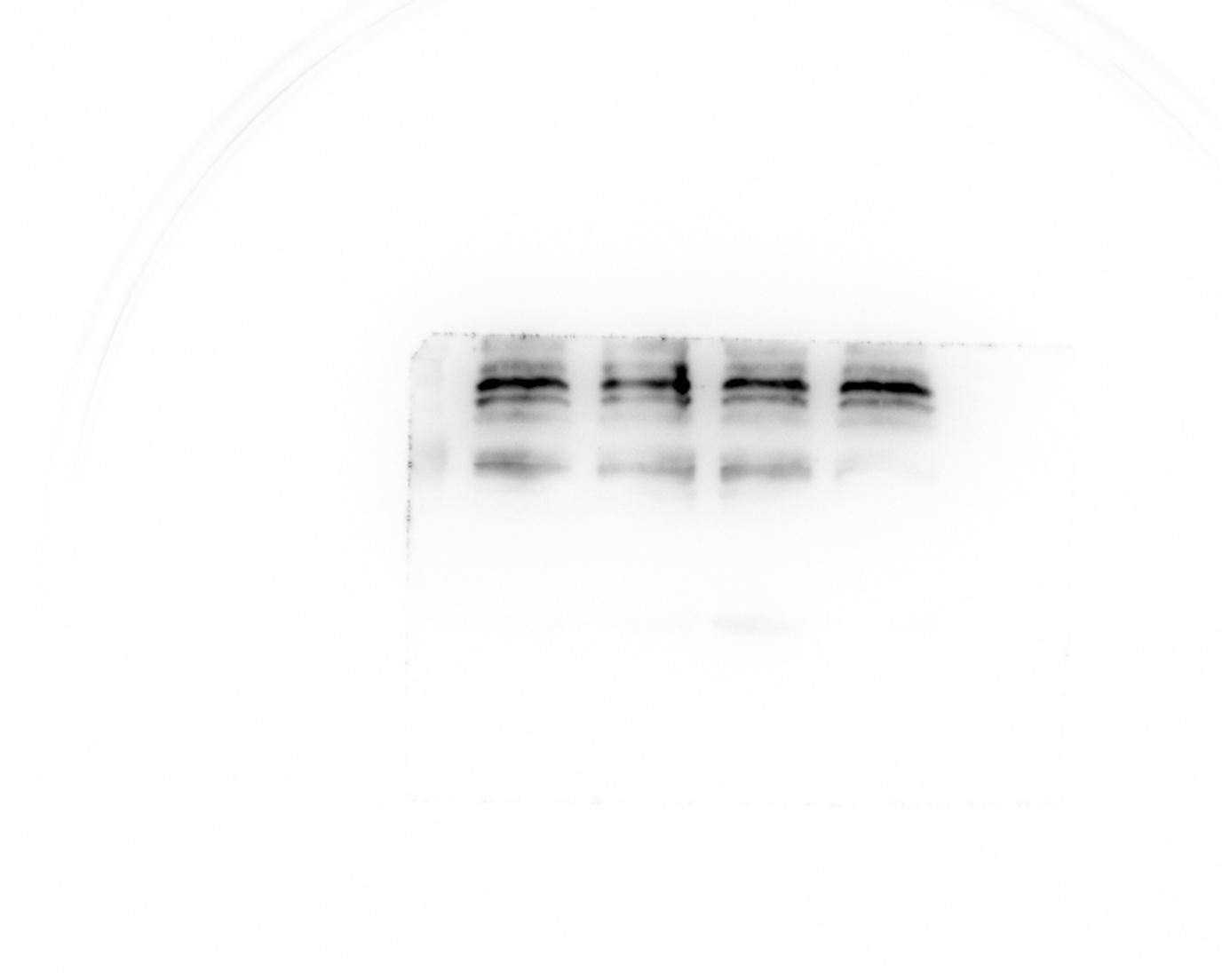

Supplement: Supplemental Information 9 [file peerj-13-19276-s009.zip › western blot-Total Cx43 EB1 N-cadherin 1/10-N-cadherin.Tif]

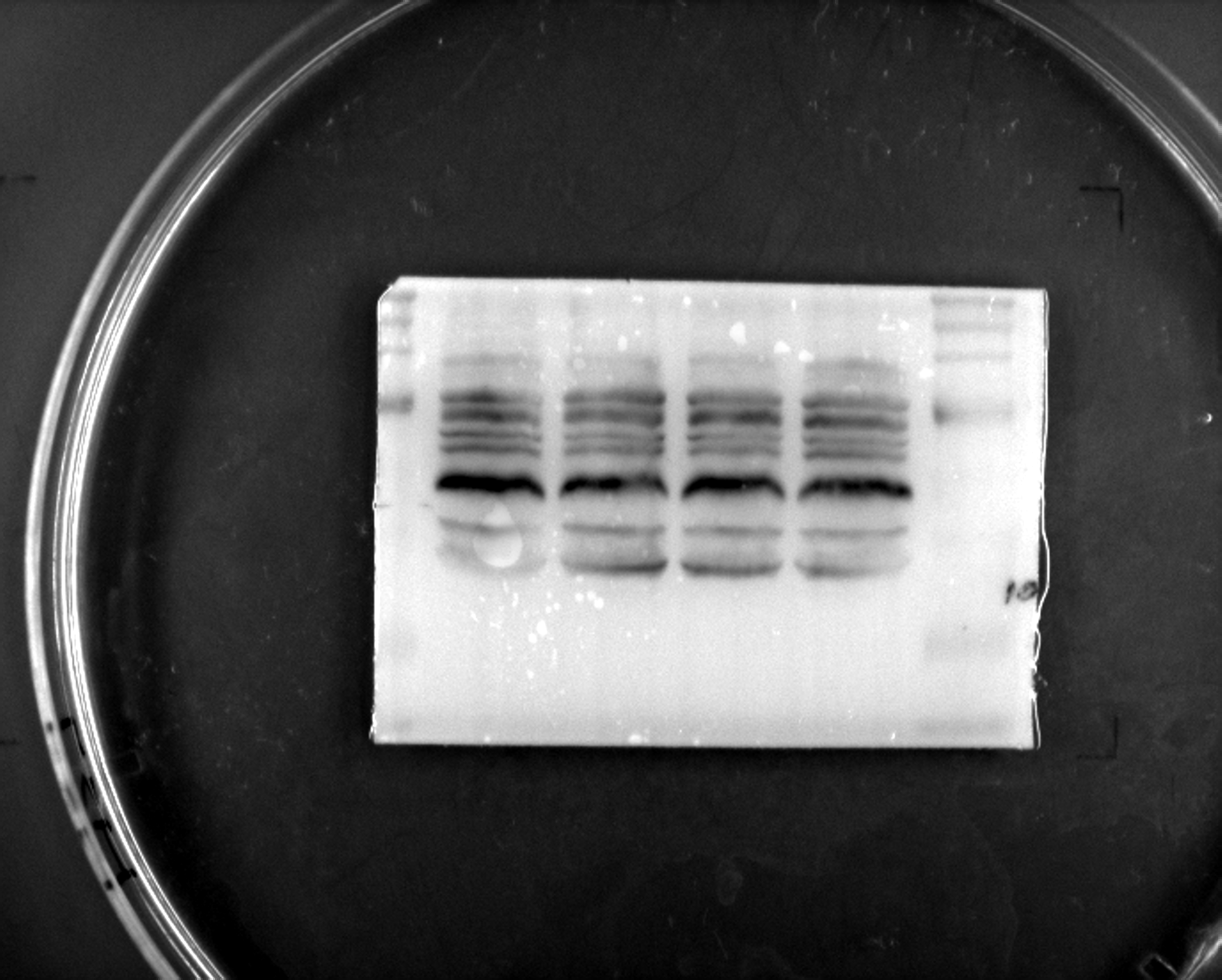

Supplement: Supplemental Information 9 [file peerj-13-19276-s009.zip › western blot-Total Cx43 EB1 N-cadherin 1/10-Tubulin-M-used.Tif]

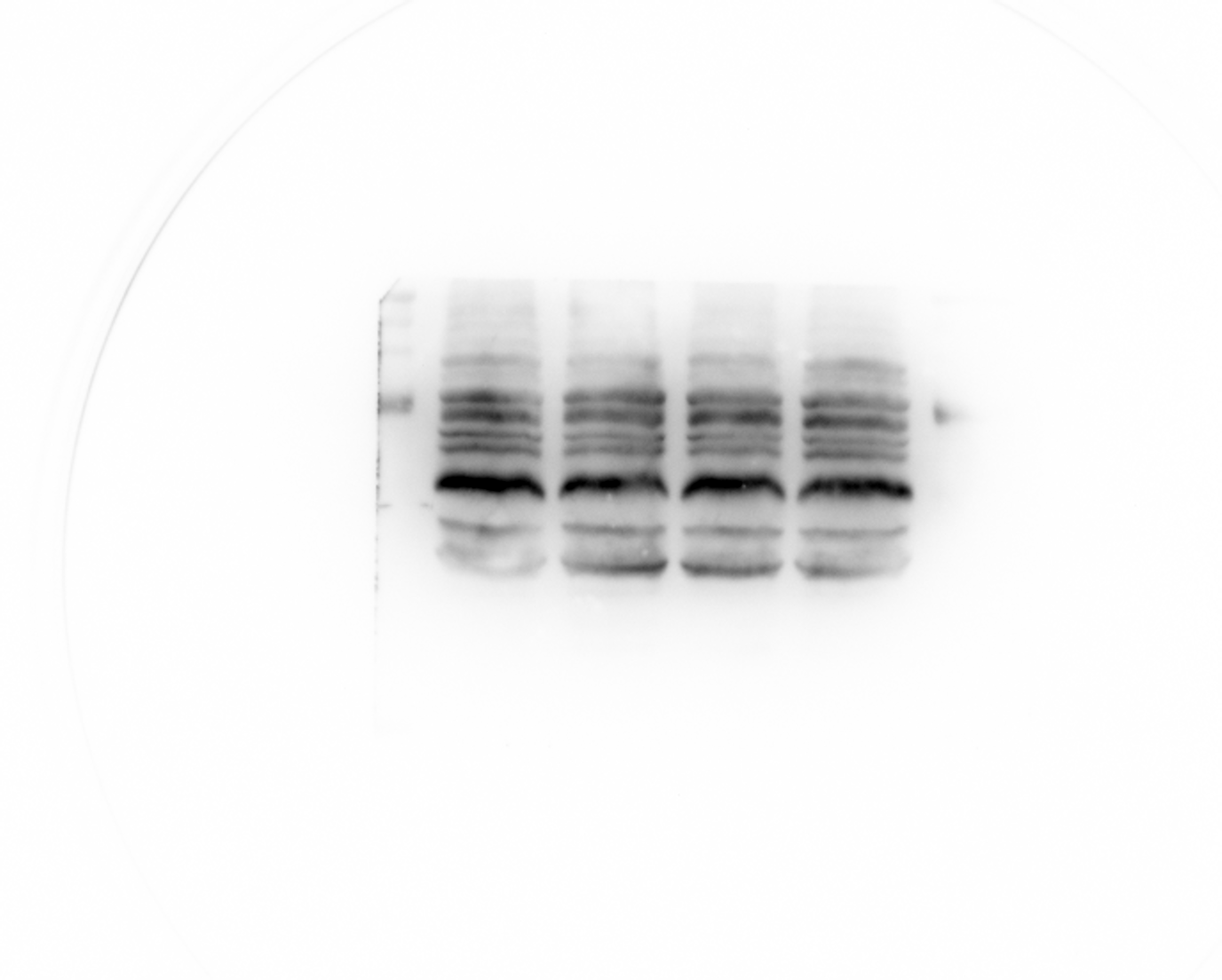

Supplement: Supplemental Information 9 [file peerj-13-19276-s009.zip › western blot-Total Cx43 EB1 N-cadherin 1/10-Tubulin-used.Tif]

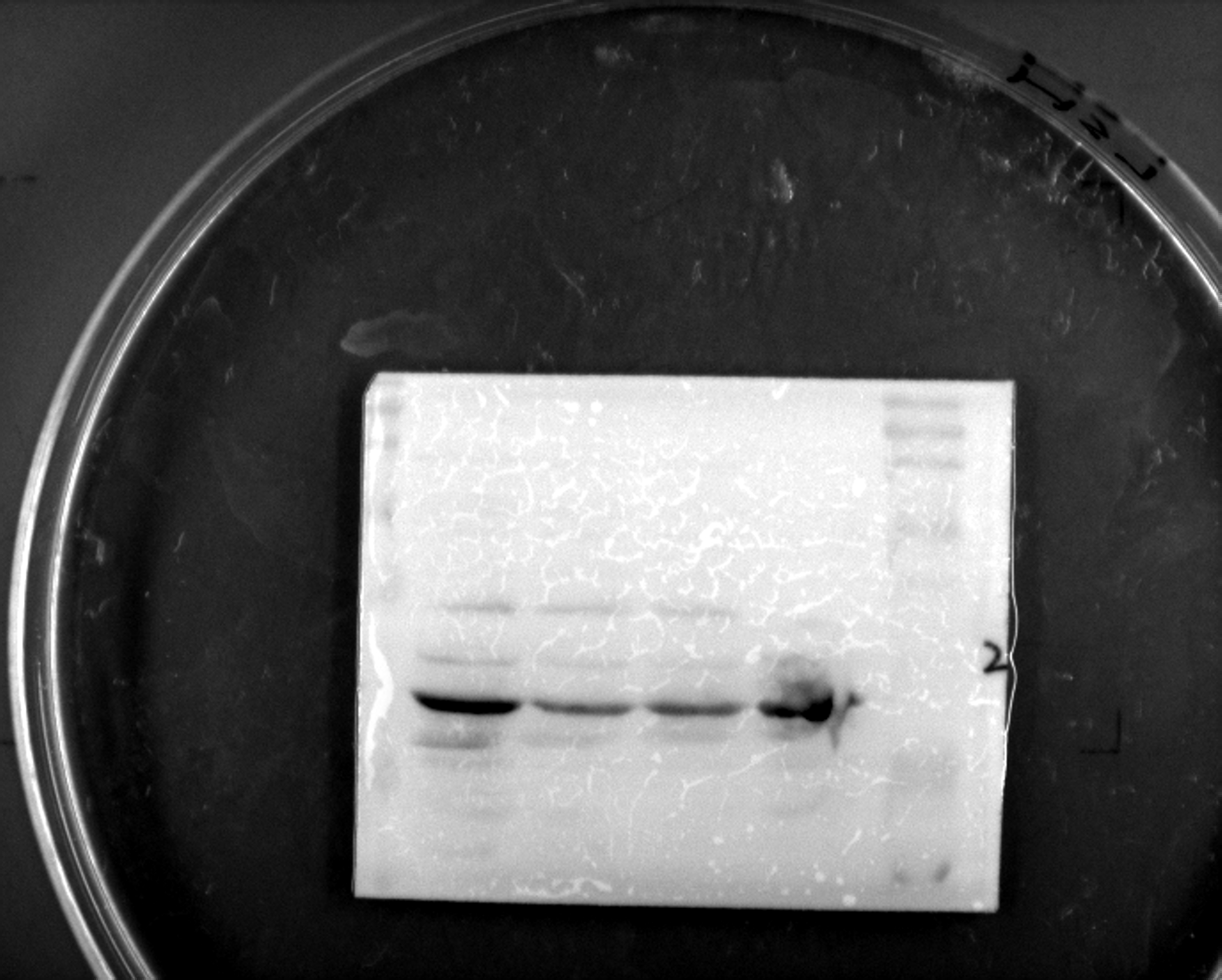

Supplement: Supplemental Information 9 [file peerj-13-19276-s009.zip › western blot-Total Cx43 EB1 N-cadherin 1/2-cx43-M.Tif]

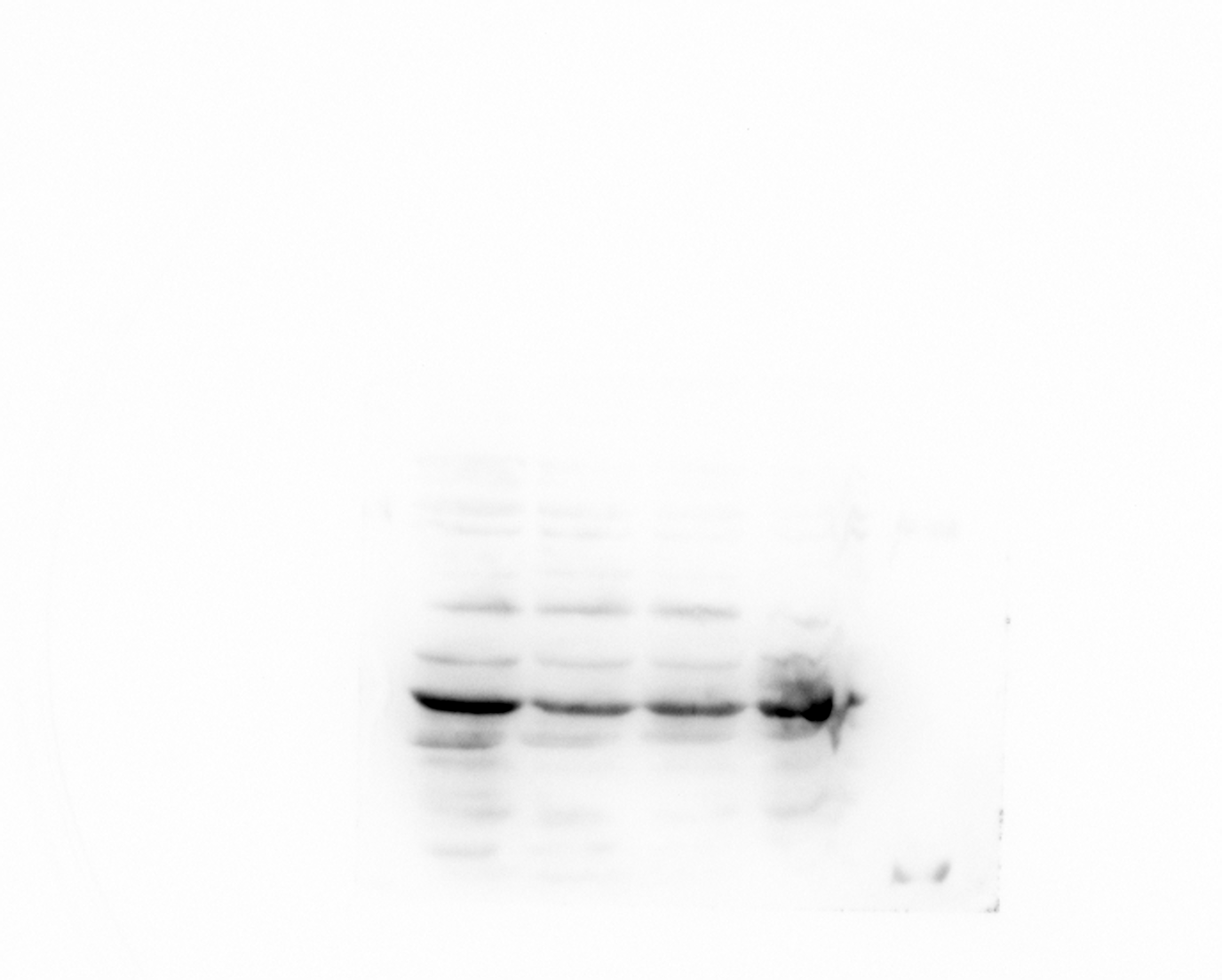

Supplement: Supplemental Information 9 [file peerj-13-19276-s009.zip › western blot-Total Cx43 EB1 N-cadherin 1/2-cx43.Tif]

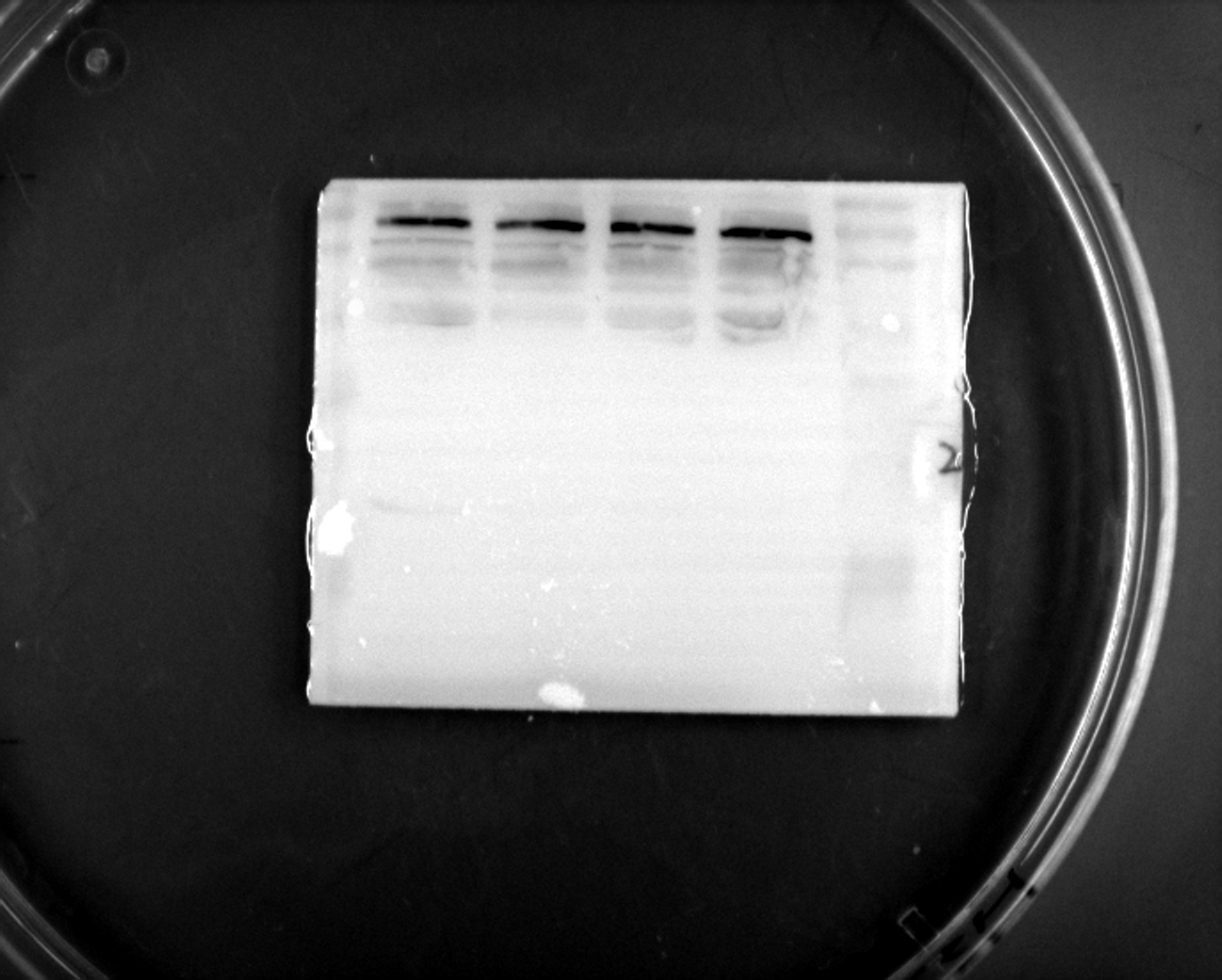

Supplement: Supplemental Information 9 [file peerj-13-19276-s009.zip › western blot-Total Cx43 EB1 N-cadherin 1/2-N-cadherin-M.Tif]

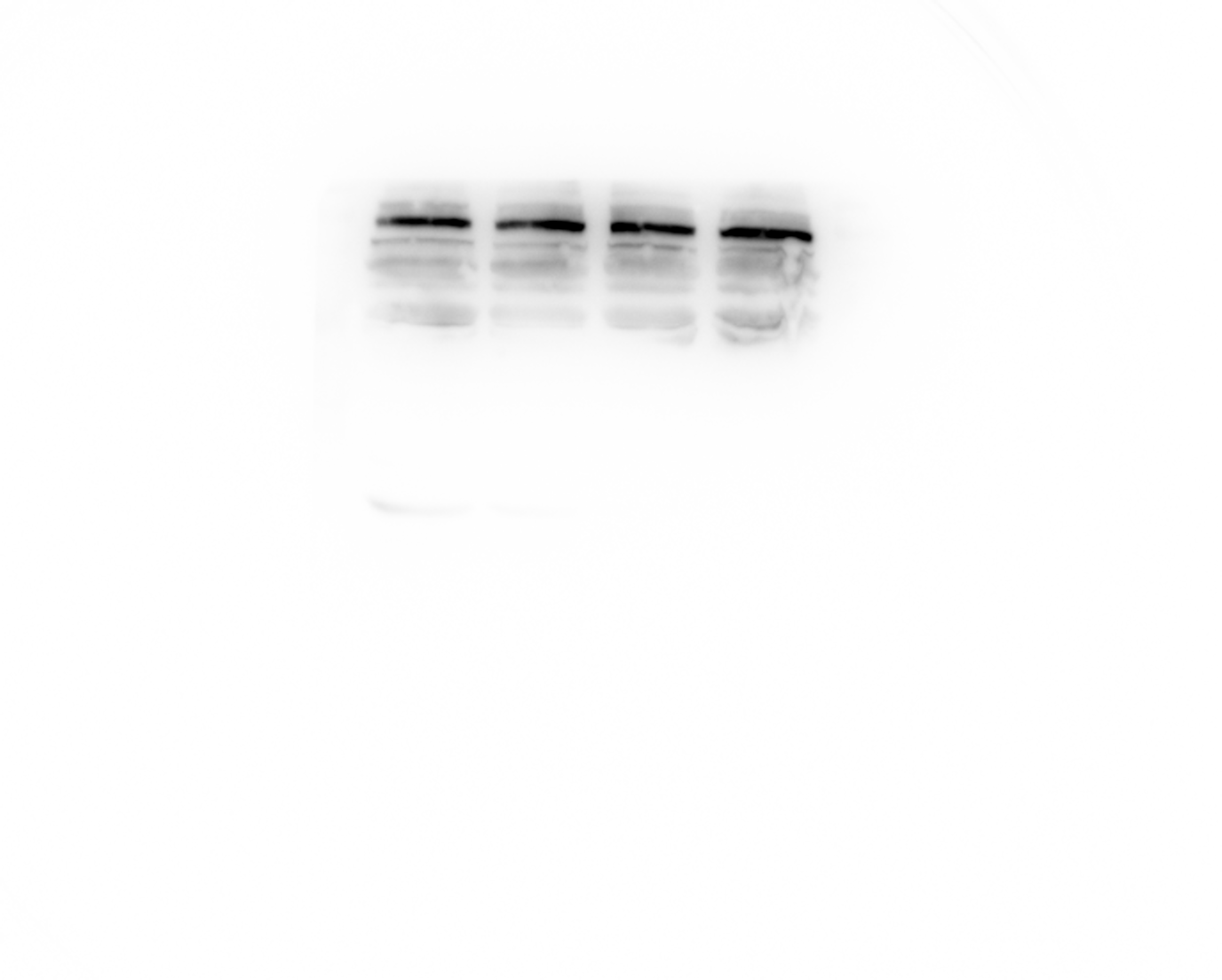

Supplement: Supplemental Information 9 [file peerj-13-19276-s009.zip › western blot-Total Cx43 EB1 N-cadherin 1/2-N-cadherin.Tif]

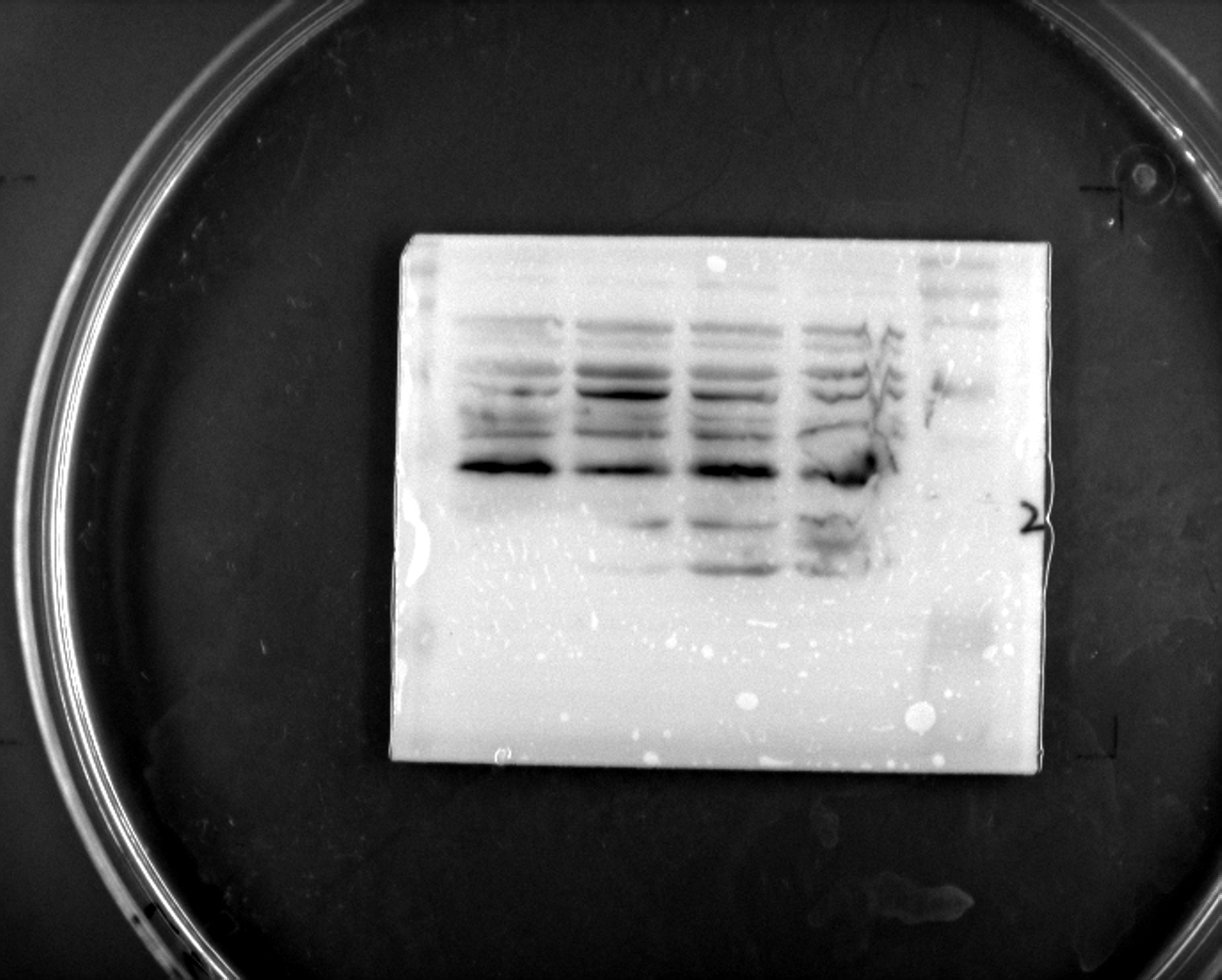

Supplement: Supplemental Information 9 [file peerj-13-19276-s009.zip › western blot-Total Cx43 EB1 N-cadherin 1/2-Tubulin-M.Tif]

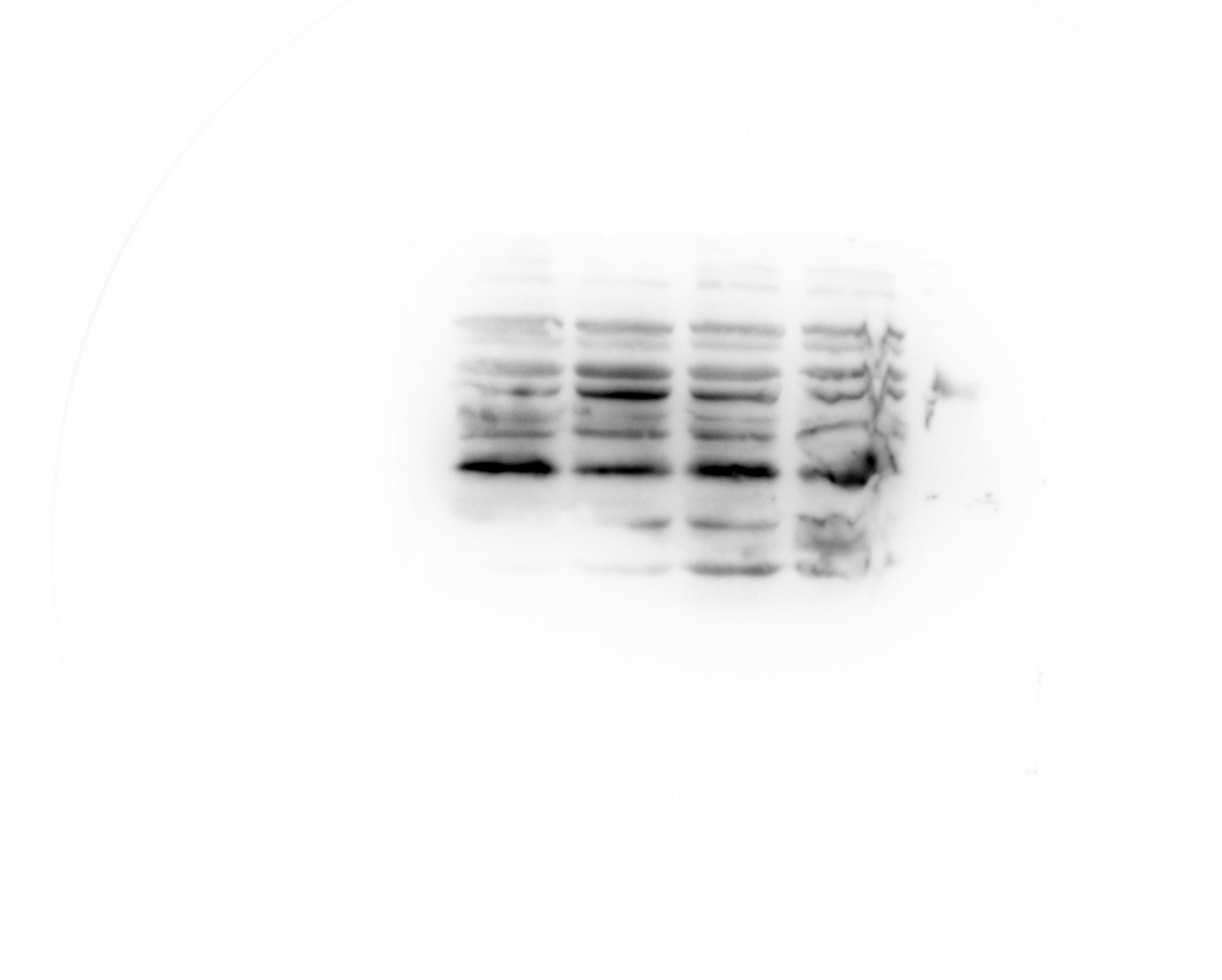

Supplement: Supplemental Information 9 [file peerj-13-19276-s009.zip › western blot-Total Cx43 EB1 N-cadherin 1/2-Tubulin.Tif]

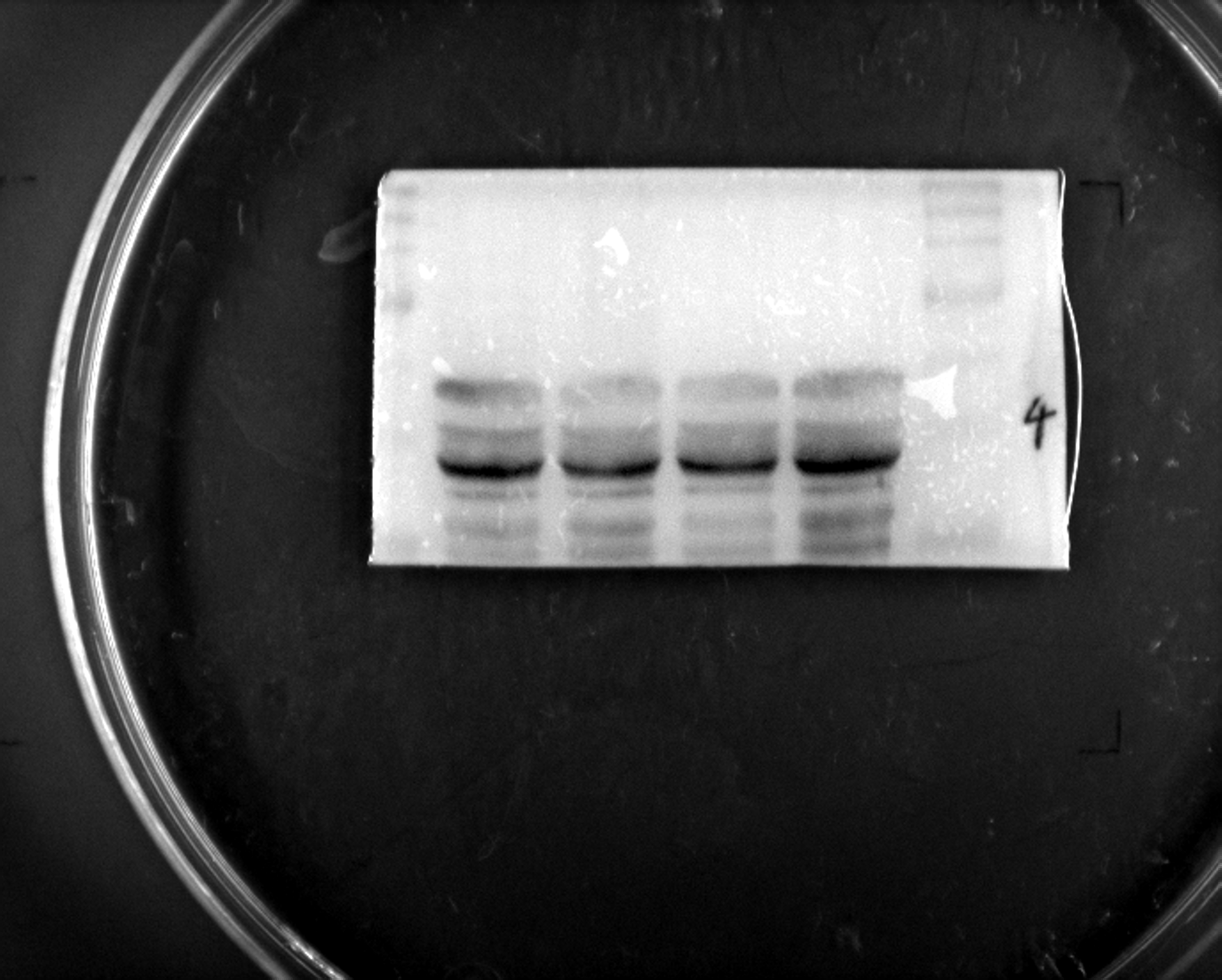

Supplement: Supplemental Information 9 [file peerj-13-19276-s009.zip › western blot-Total Cx43 EB1 N-cadherin 1/4-cx43-M.Tif]

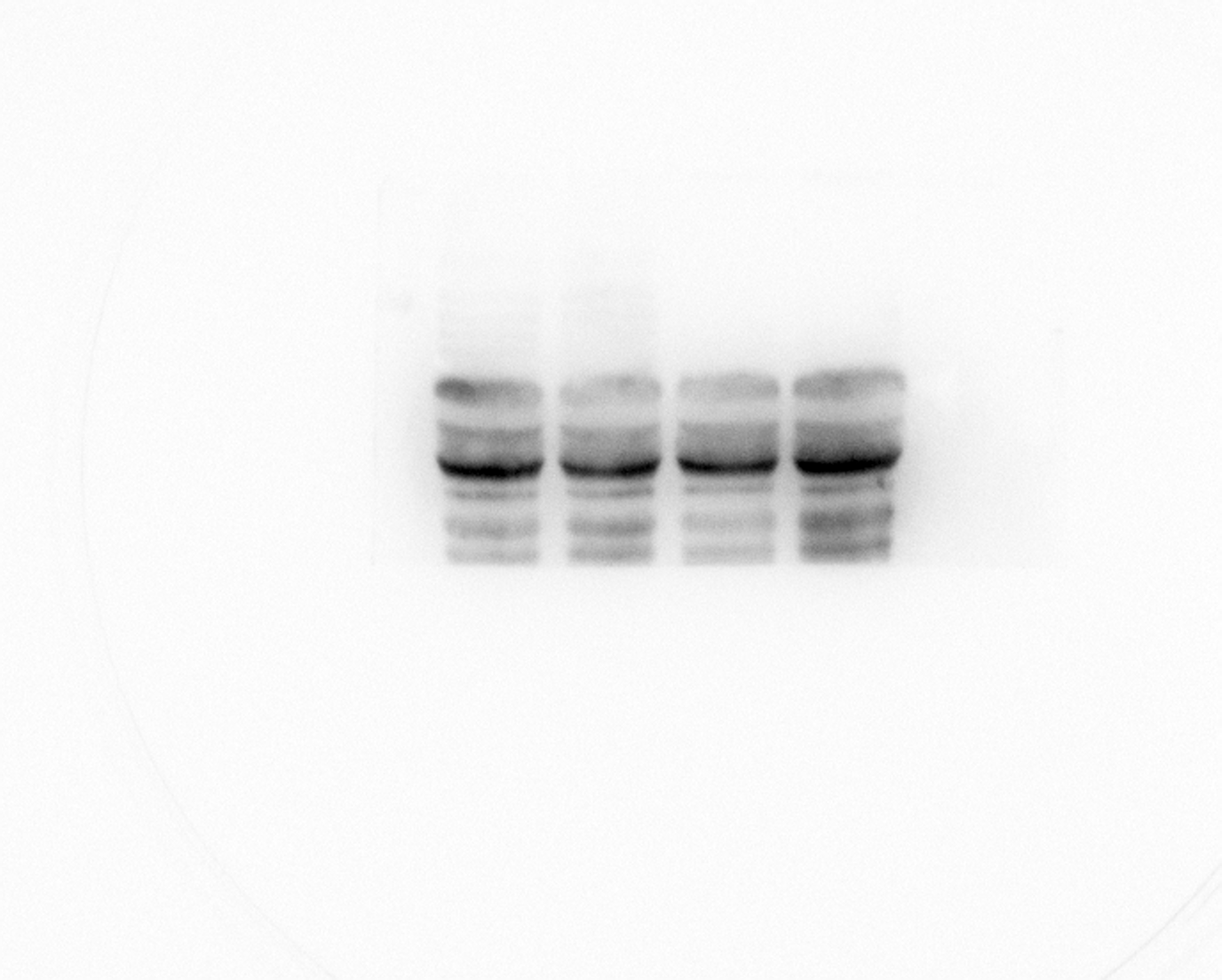

Supplement: Supplemental Information 9 [file peerj-13-19276-s009.zip › western blot-Total Cx43 EB1 N-cadherin 1/4-cx43.Tif]

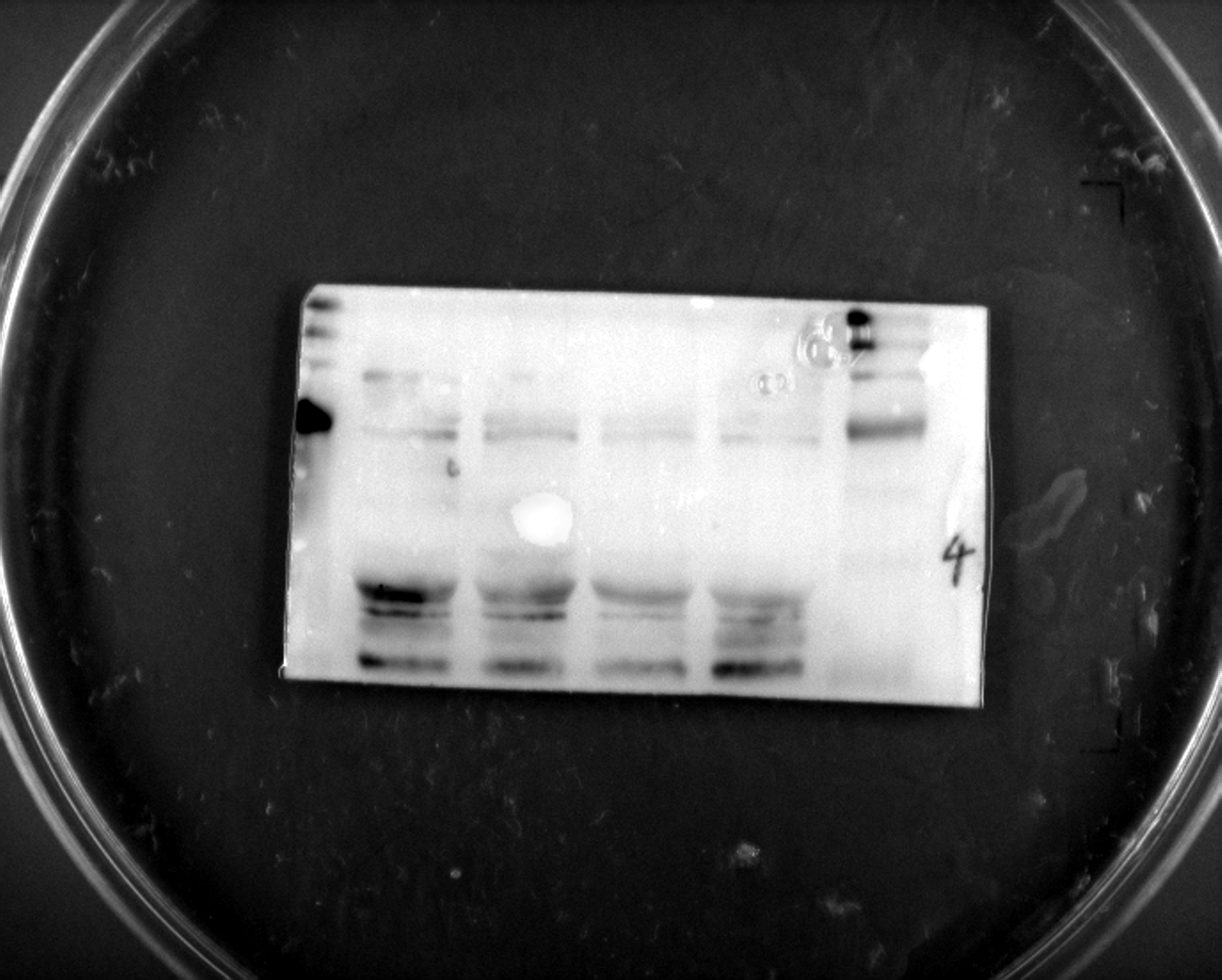

Supplement: Supplemental Information 9 [file peerj-13-19276-s009.zip › western blot-Total Cx43 EB1 N-cadherin 1/4-EB1-M.Tif]

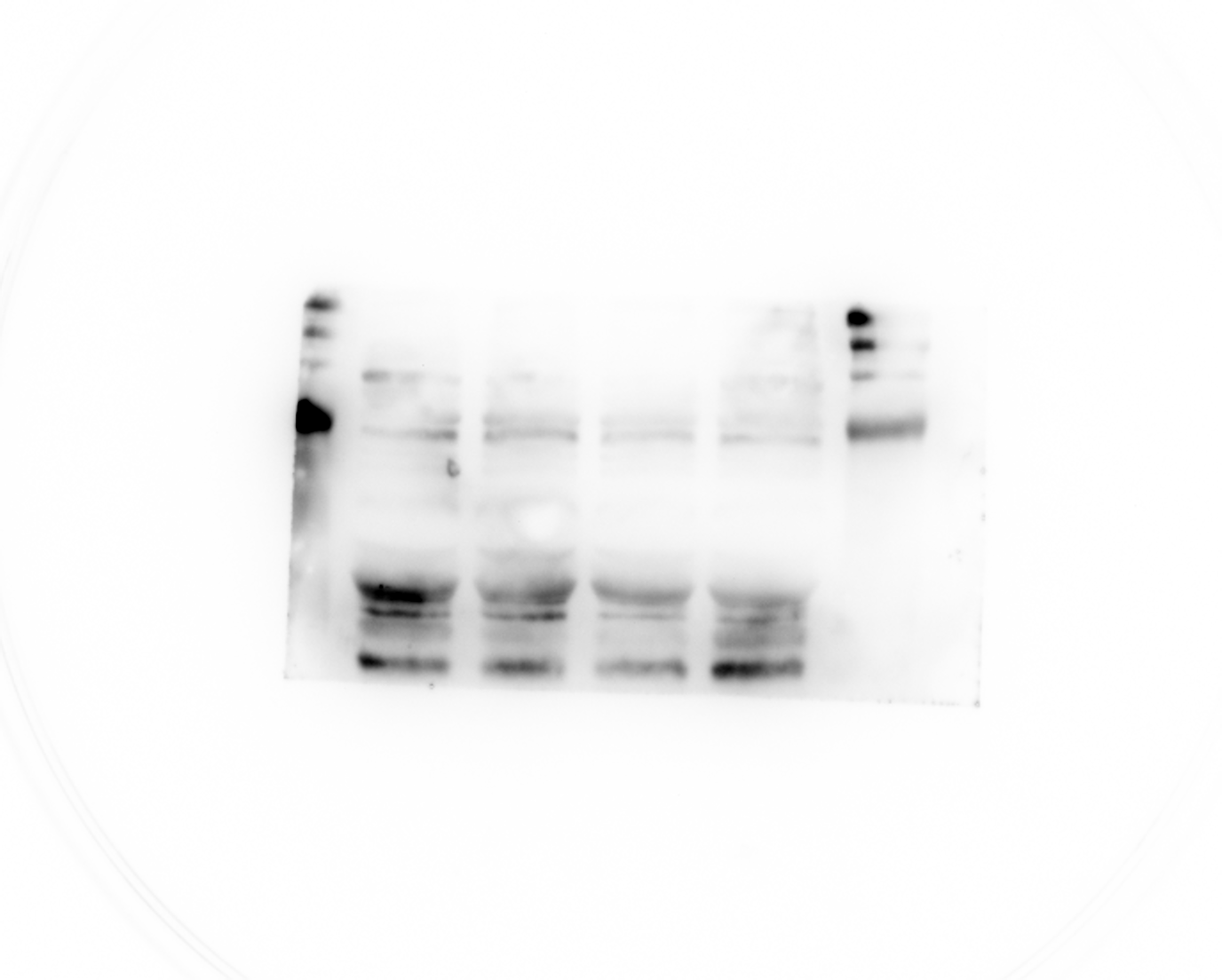

Supplement: Supplemental Information 9 [file peerj-13-19276-s009.zip › western blot-Total Cx43 EB1 N-cadherin 1/4-EB1.Tif]

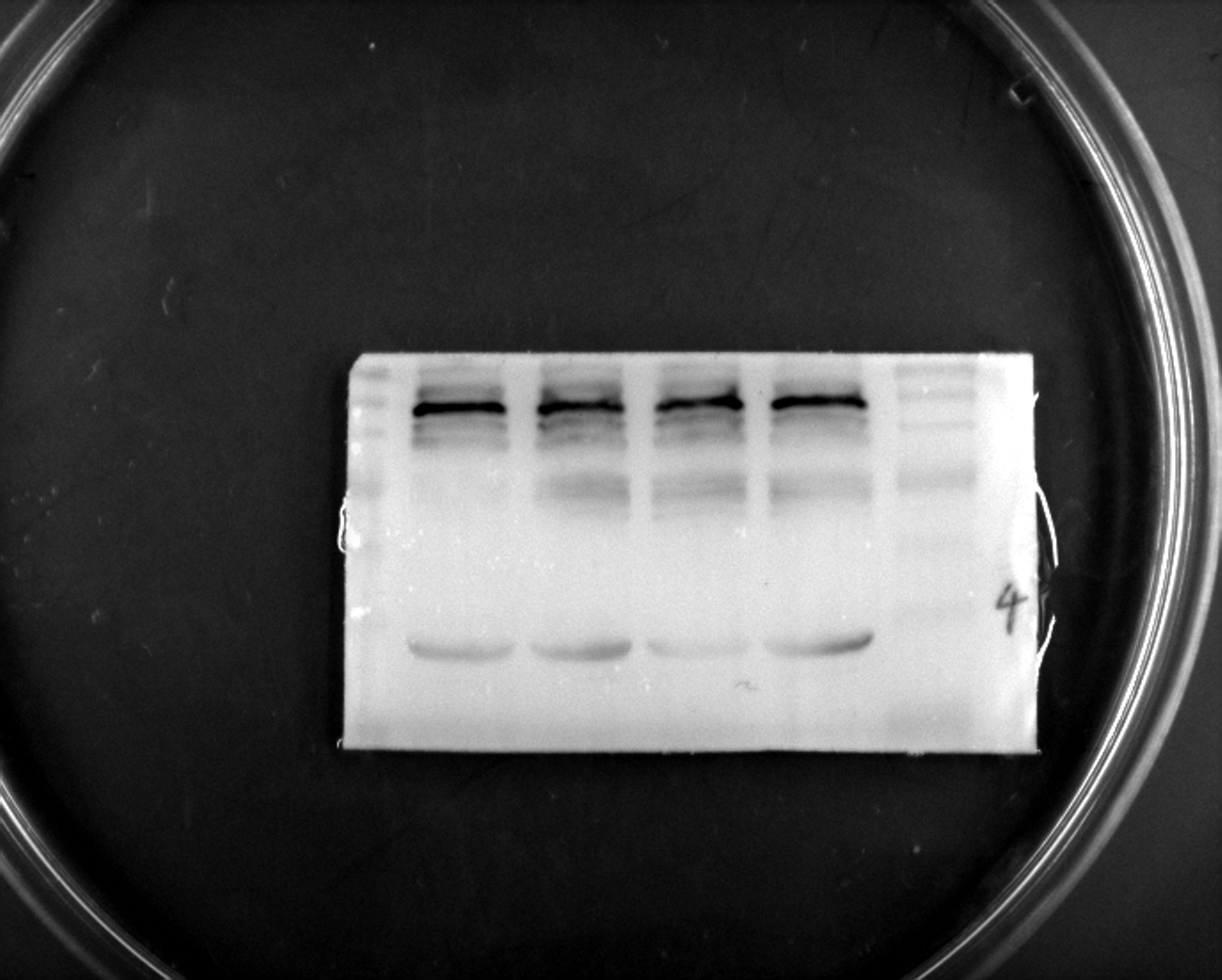

Supplement: Supplemental Information 9 [file peerj-13-19276-s009.zip › western blot-Total Cx43 EB1 N-cadherin 1/4-N-cadherin-M-used.Tif]

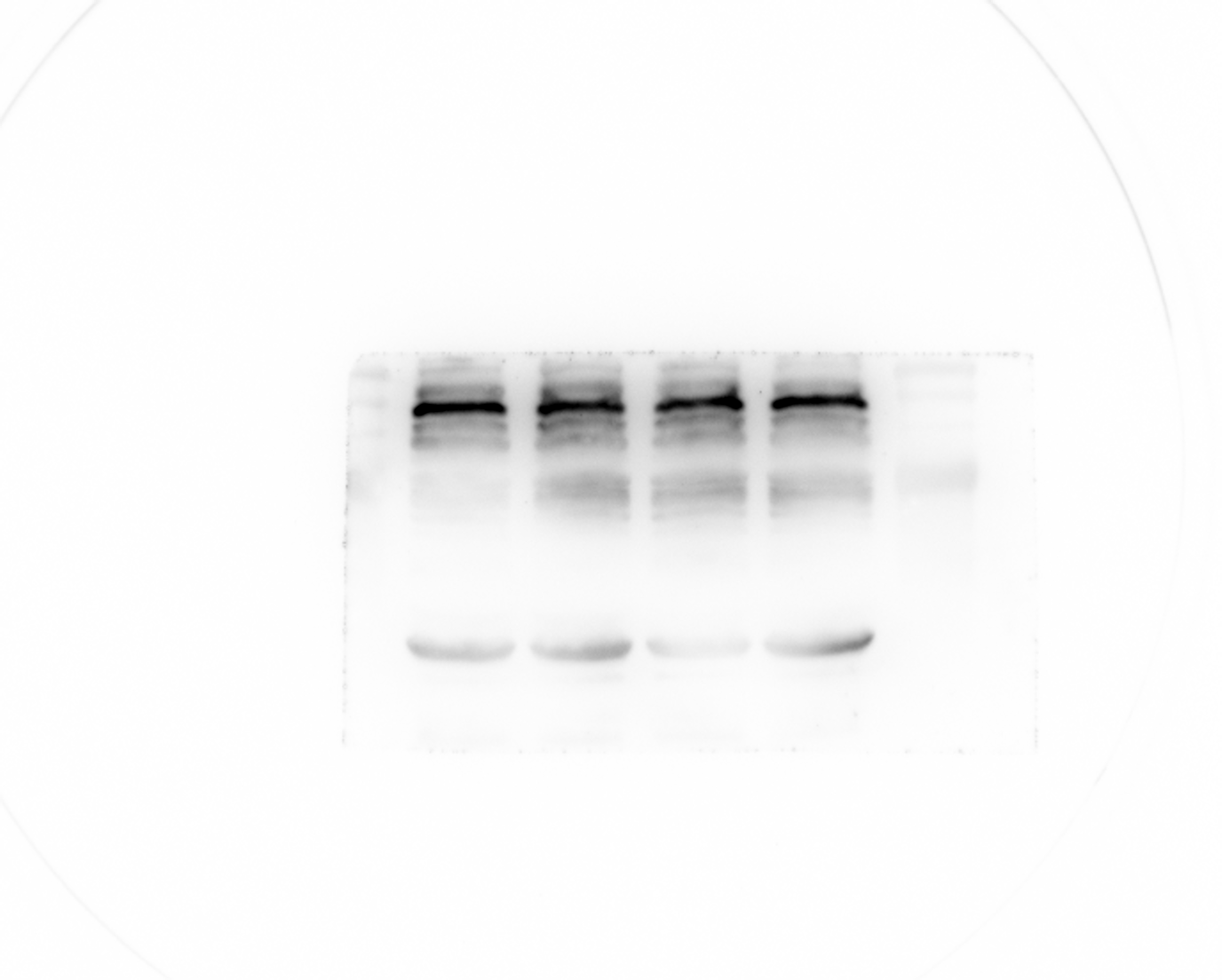

Supplement: Supplemental Information 9 [file peerj-13-19276-s009.zip › western blot-Total Cx43 EB1 N-cadherin 1/4-N-cadherin-used.Tif]

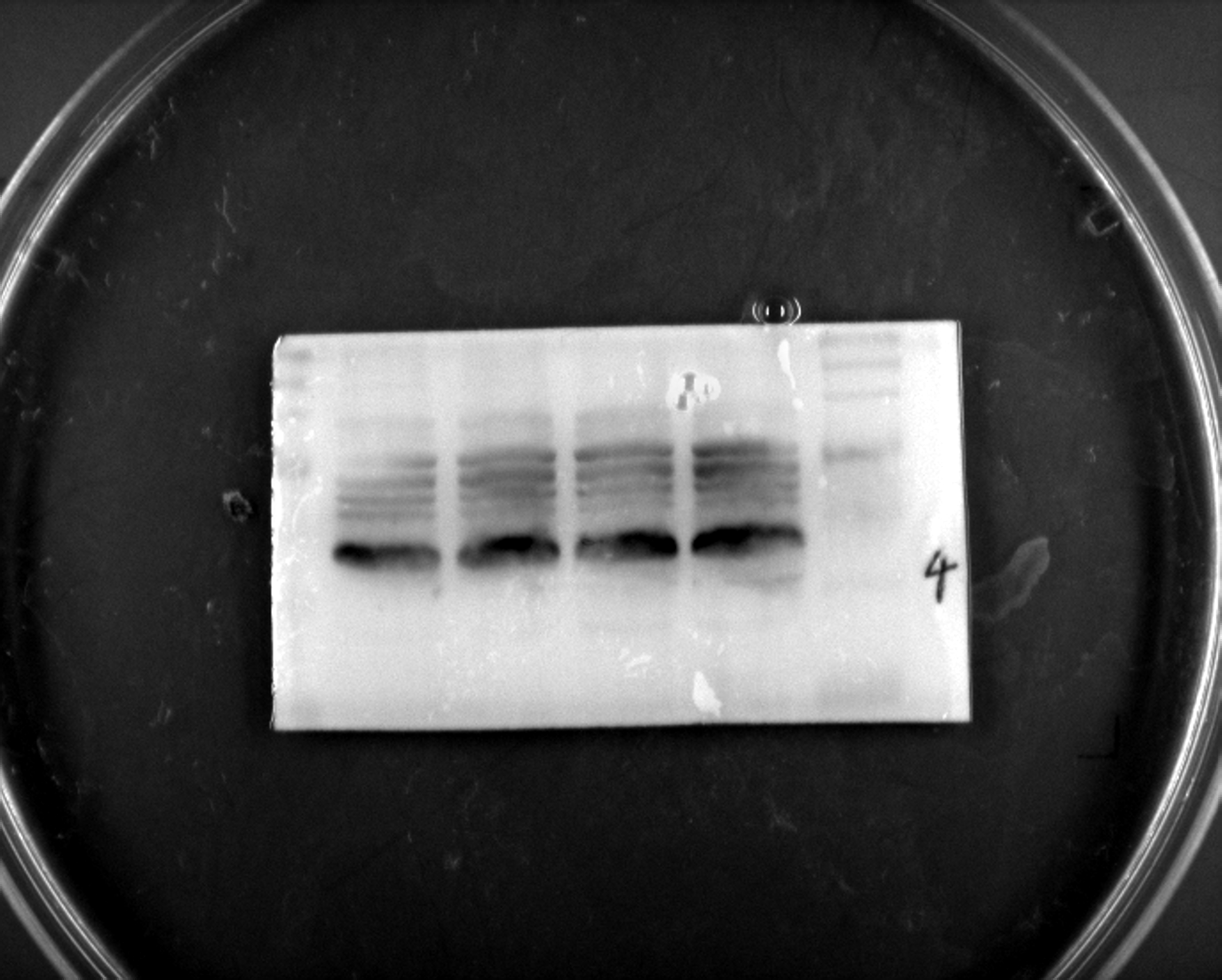

Supplement: Supplemental Information 9 [file peerj-13-19276-s009.zip › western blot-Total Cx43 EB1 N-cadherin 1/4-Tubulin-M.Tif]

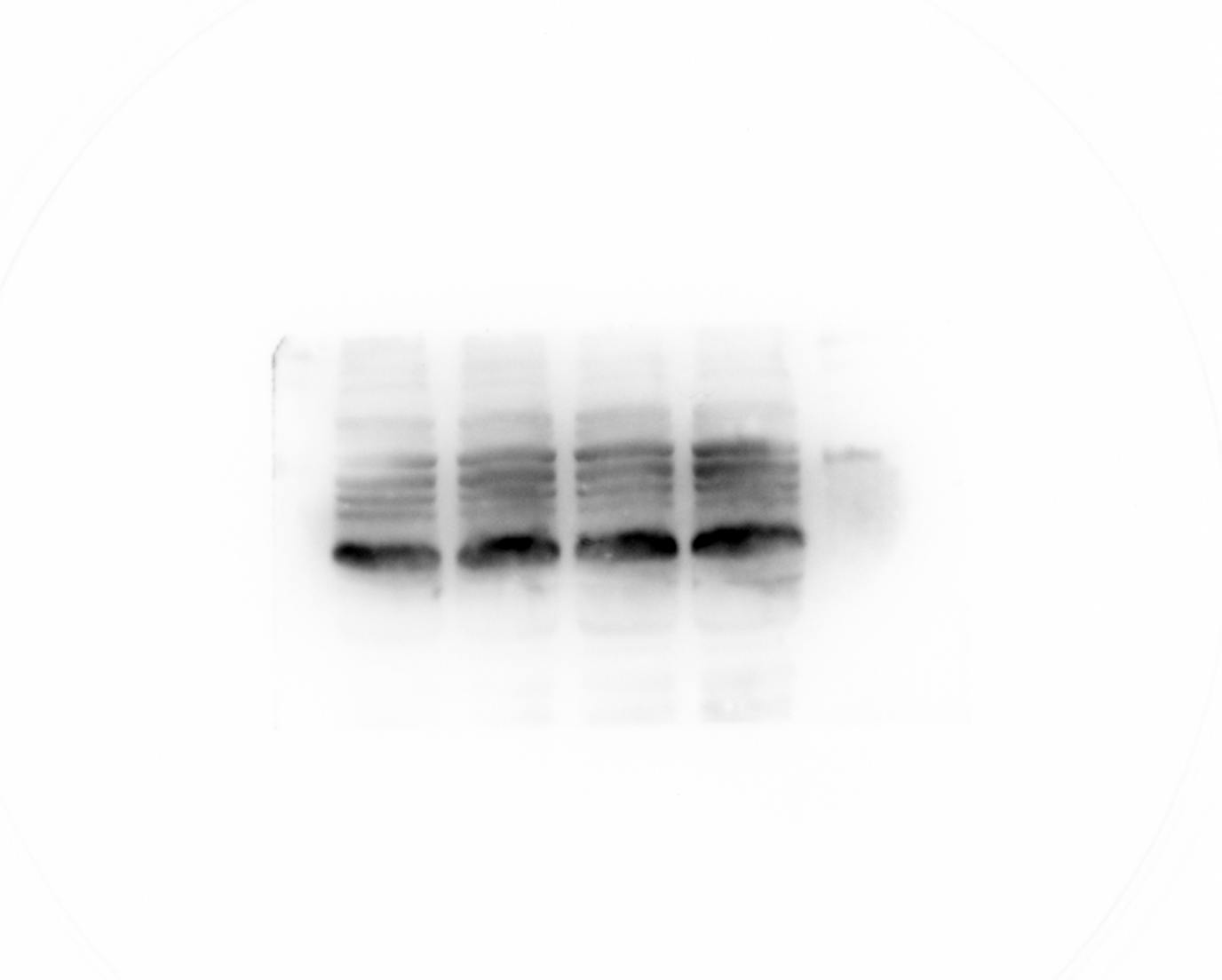

Supplement: Supplemental Information 9 [file peerj-13-19276-s009.zip › western blot-Total Cx43 EB1 N-cadherin 1/4-Tubulin.Tif]

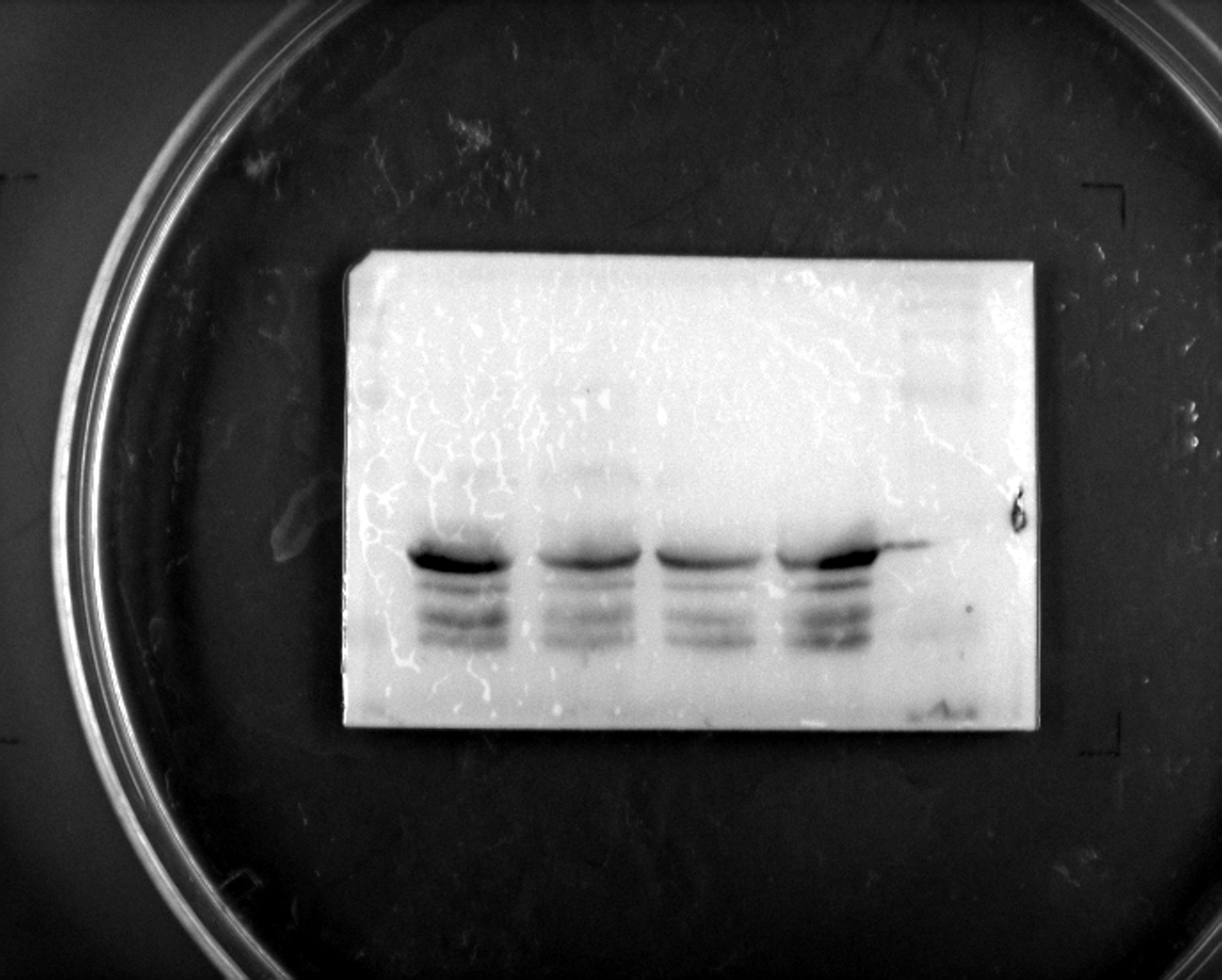

Supplement: Supplemental Information 9 [file peerj-13-19276-s009.zip › western blot-Total Cx43 EB1 N-cadherin 1/6-cx43-M.Tif]

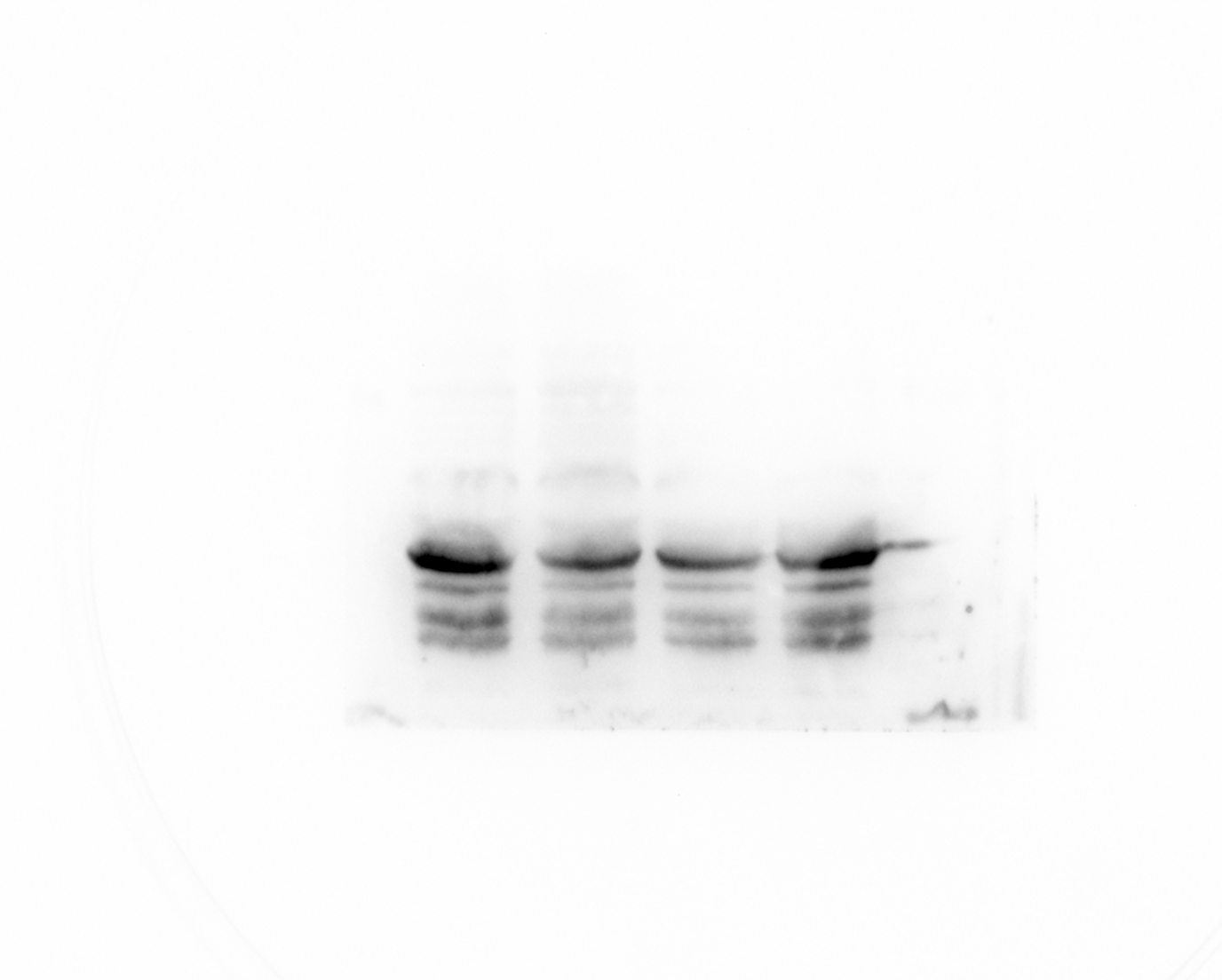

Supplement: Supplemental Information 9 [file peerj-13-19276-s009.zip › western blot-Total Cx43 EB1 N-cadherin 1/6-cx43.Tif]

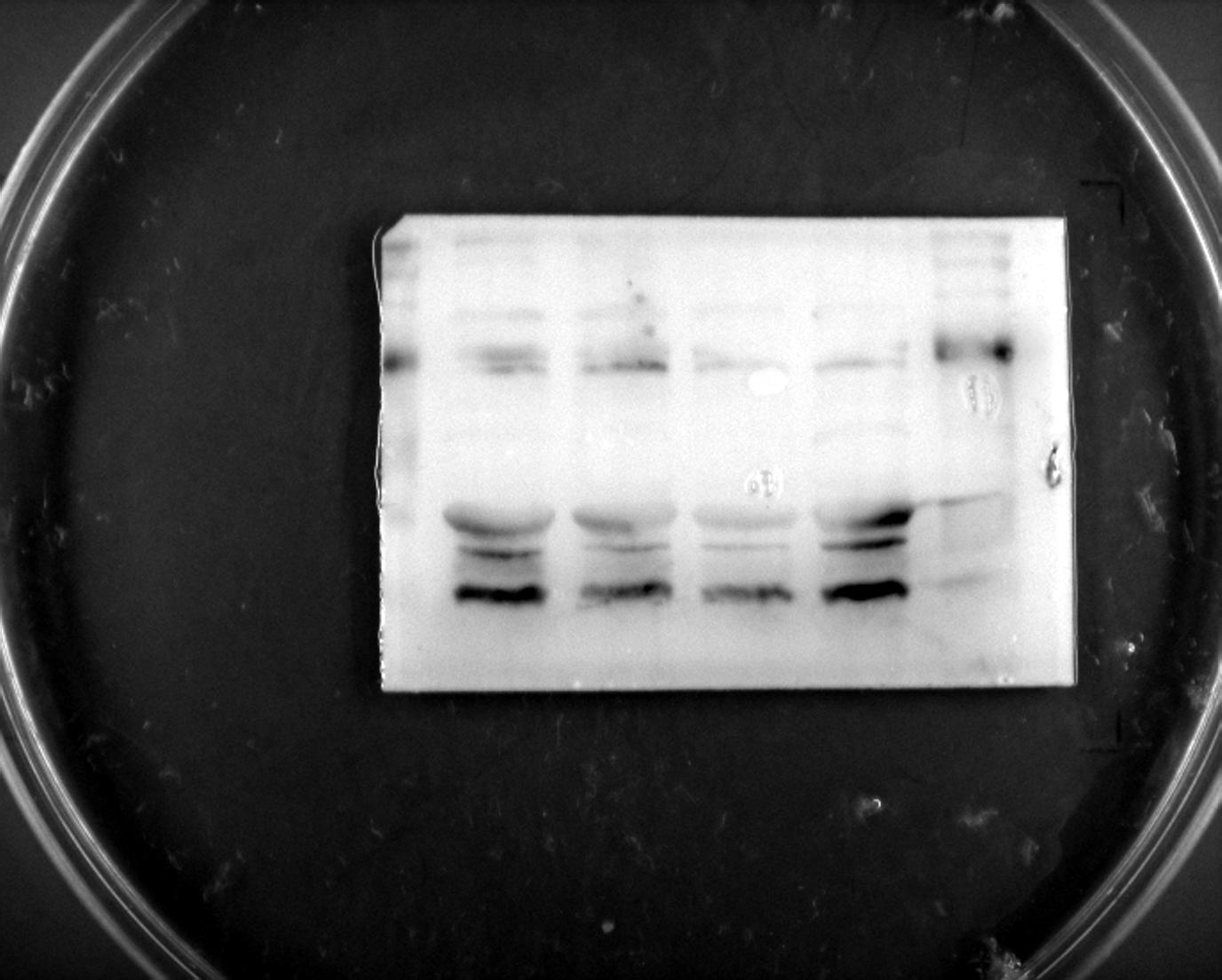

Supplement: Supplemental Information 9 [file peerj-13-19276-s009.zip › western blot-Total Cx43 EB1 N-cadherin 1/6-EB1-M.Tif]

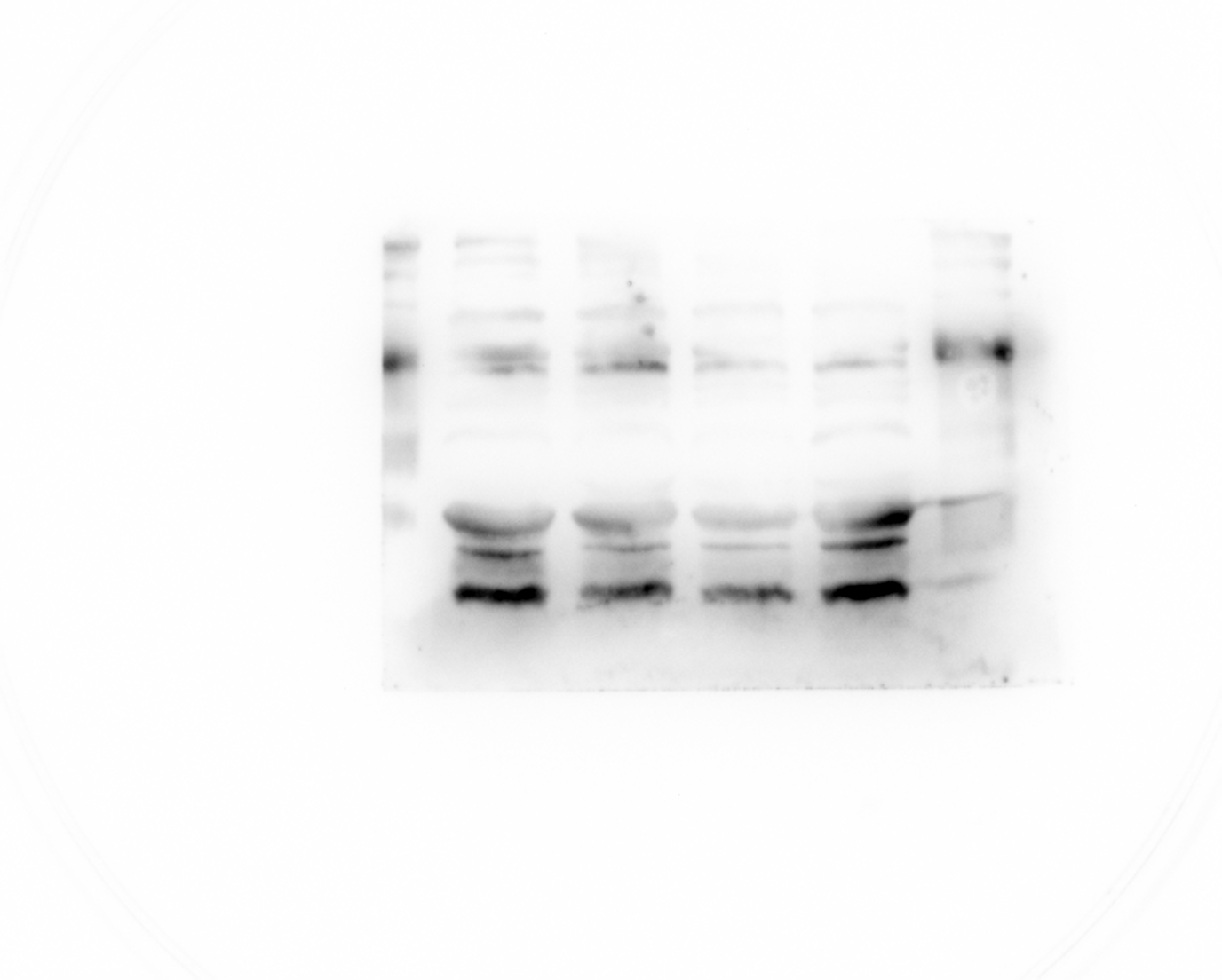

Supplement: Supplemental Information 9 [file peerj-13-19276-s009.zip › western blot-Total Cx43 EB1 N-cadherin 1/6-EB1.Tif]

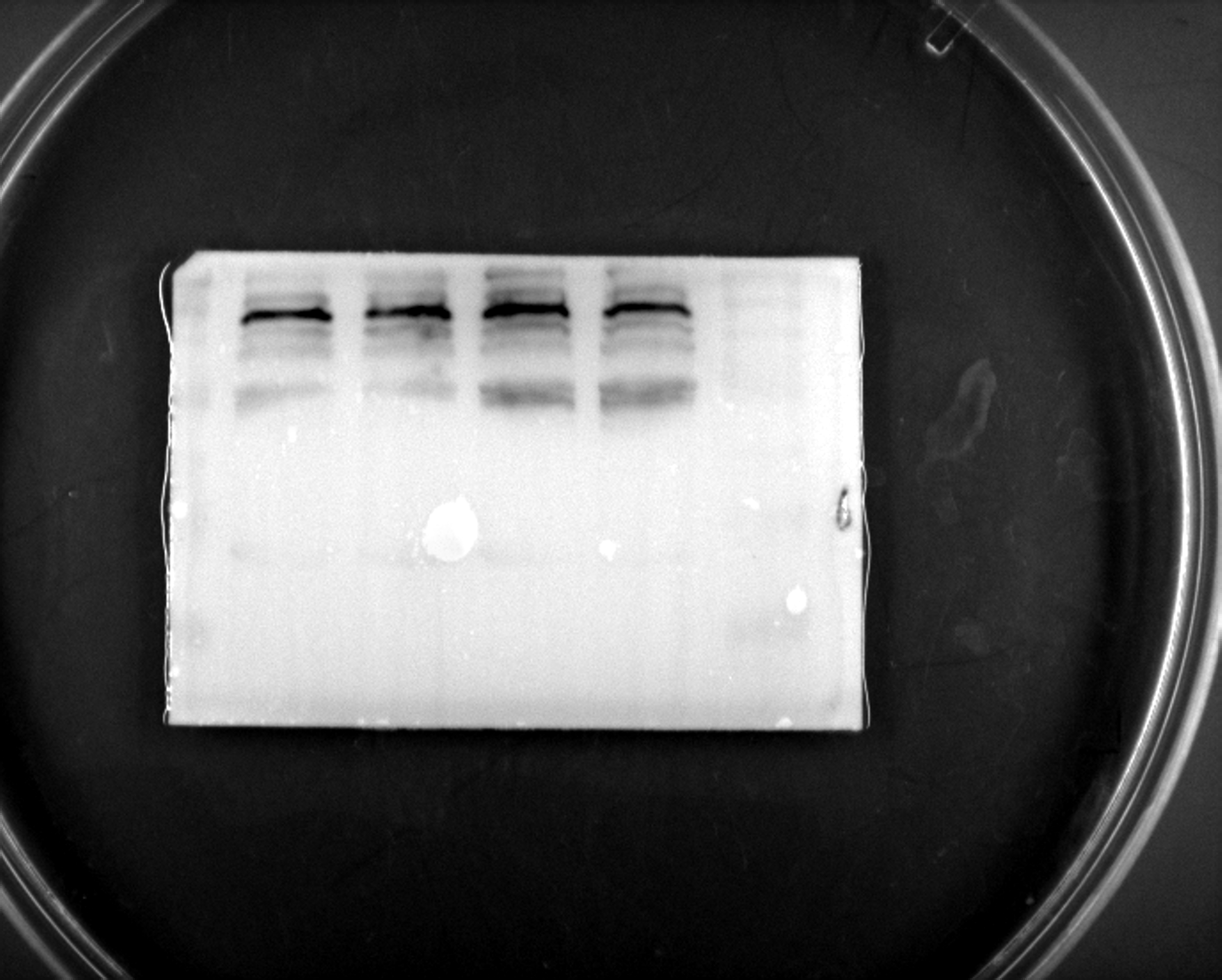

Supplement: Supplemental Information 9 [file peerj-13-19276-s009.zip › western blot-Total Cx43 EB1 N-cadherin 1/6-N-cadherin-M.Tif]

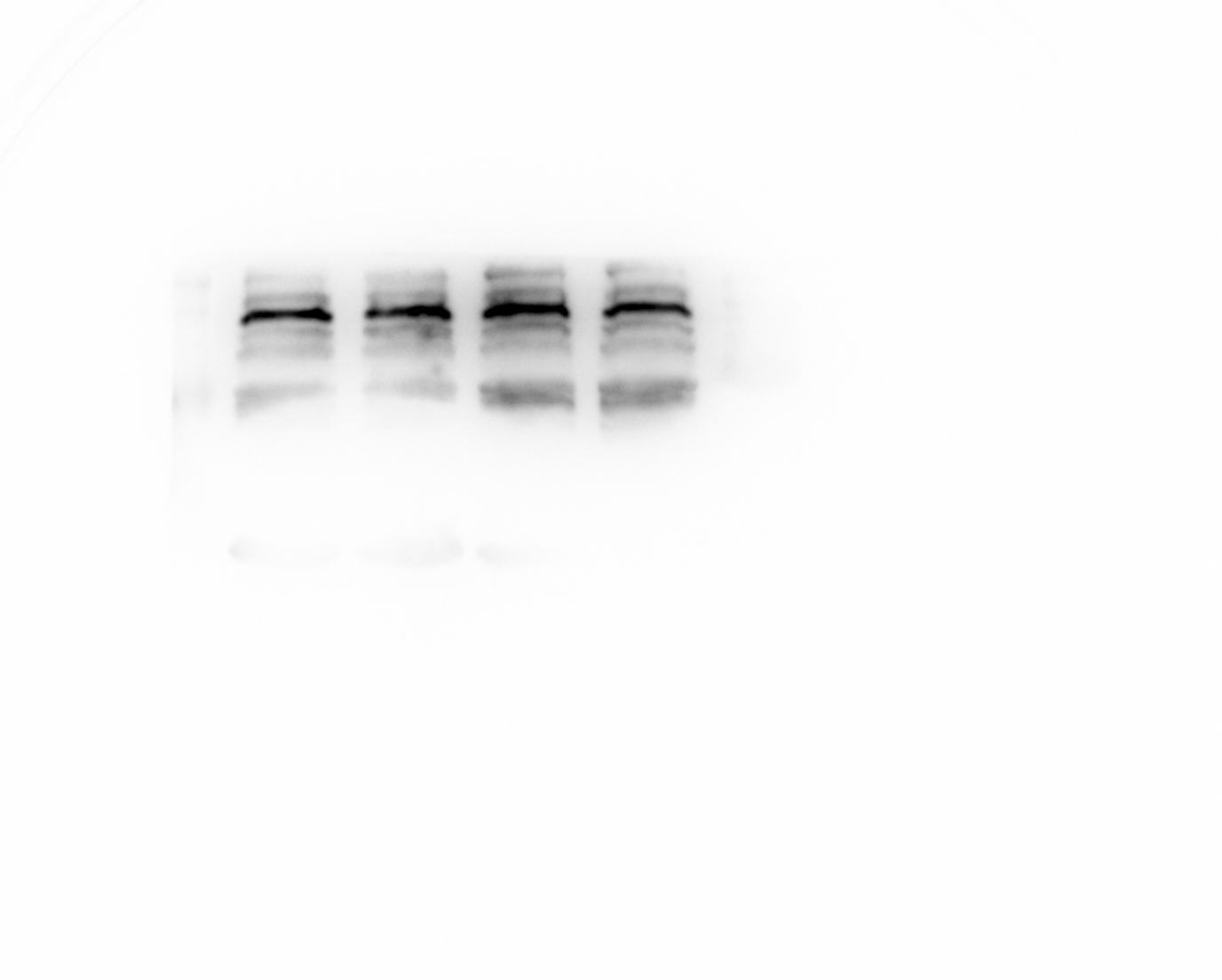

Supplement: Supplemental Information 9 [file peerj-13-19276-s009.zip › western blot-Total Cx43 EB1 N-cadherin 1/6-N-cadherin.Tif]

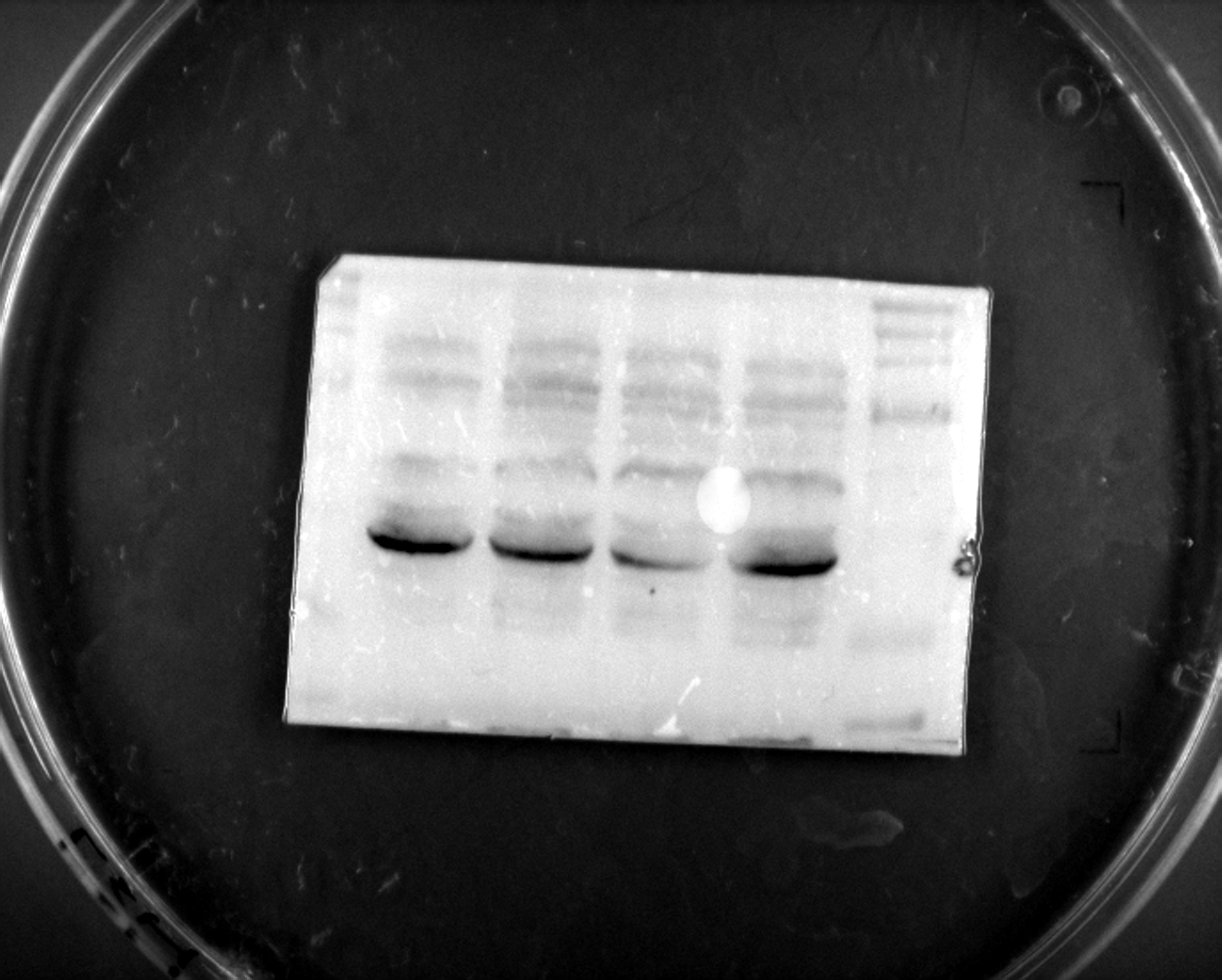

Supplement: Supplemental Information 9 [file peerj-13-19276-s009.zip › western blot-Total Cx43 EB1 N-cadherin 1/8-cx43-M.Tif]

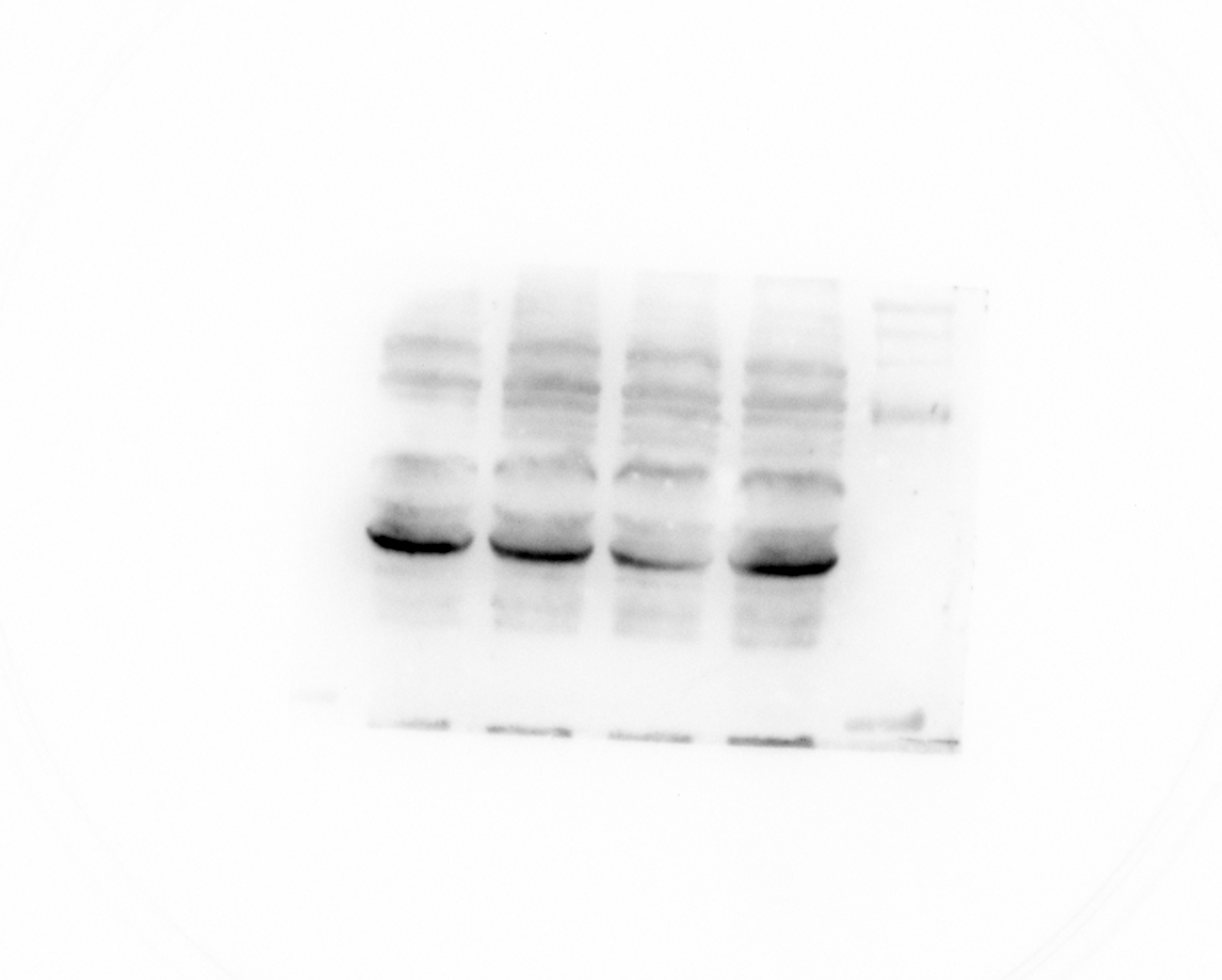

Supplement: Supplemental Information 9 [file peerj-13-19276-s009.zip › western blot-Total Cx43 EB1 N-cadherin 1/8-cx43.Tif]

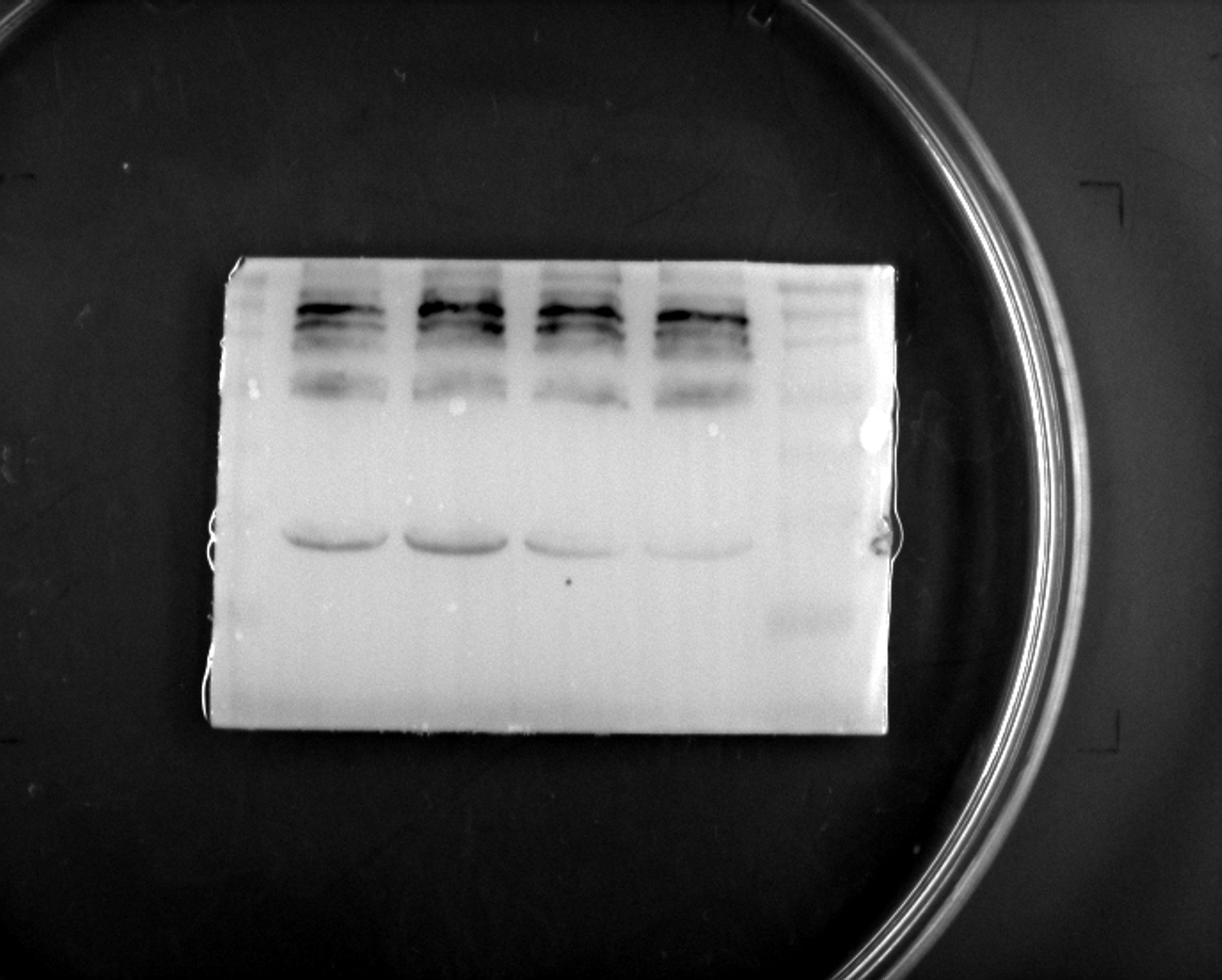

Supplement: Supplemental Information 9 [file peerj-13-19276-s009.zip › western blot-Total Cx43 EB1 N-cadherin 1/8-N-cadherin-M.Tif]

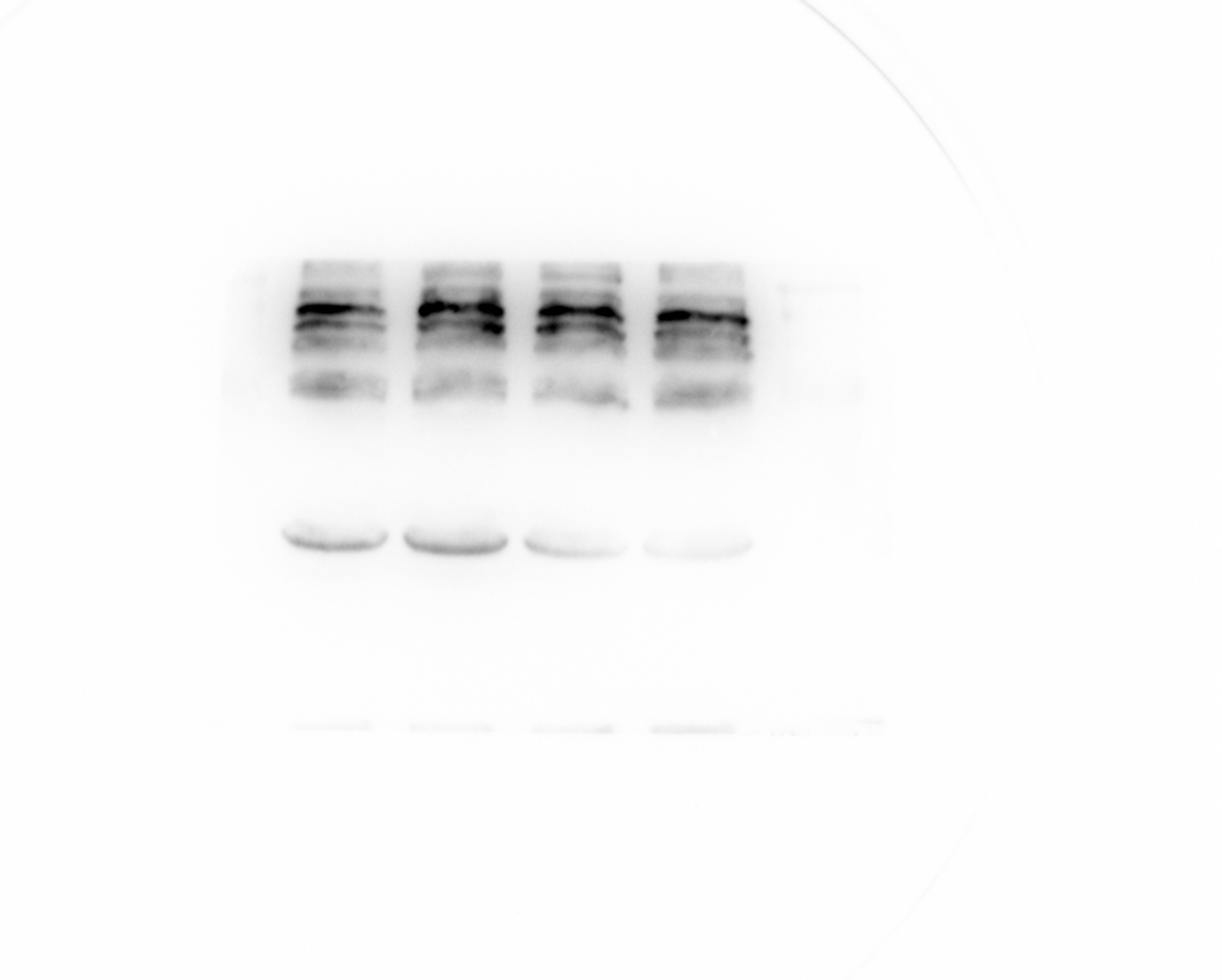

Supplement: Supplemental Information 9 [file peerj-13-19276-s009.zip › western blot-Total Cx43 EB1 N-cadherin 1/8-N-cadherin.Tif]

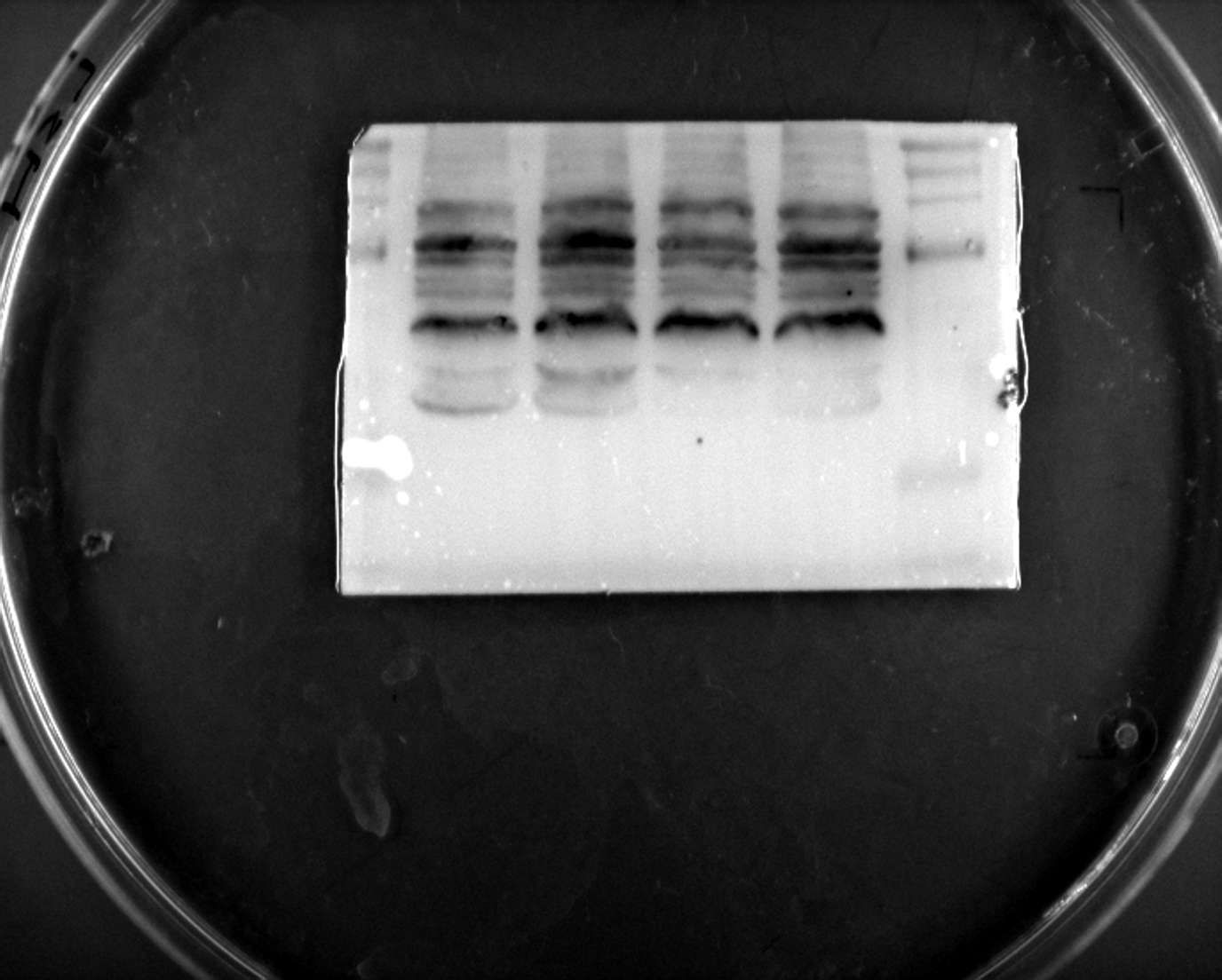

Supplement: Supplemental Information 9 [file peerj-13-19276-s009.zip › western blot-Total Cx43 EB1 N-cadherin 1/8-Tubulin-M.Tif]

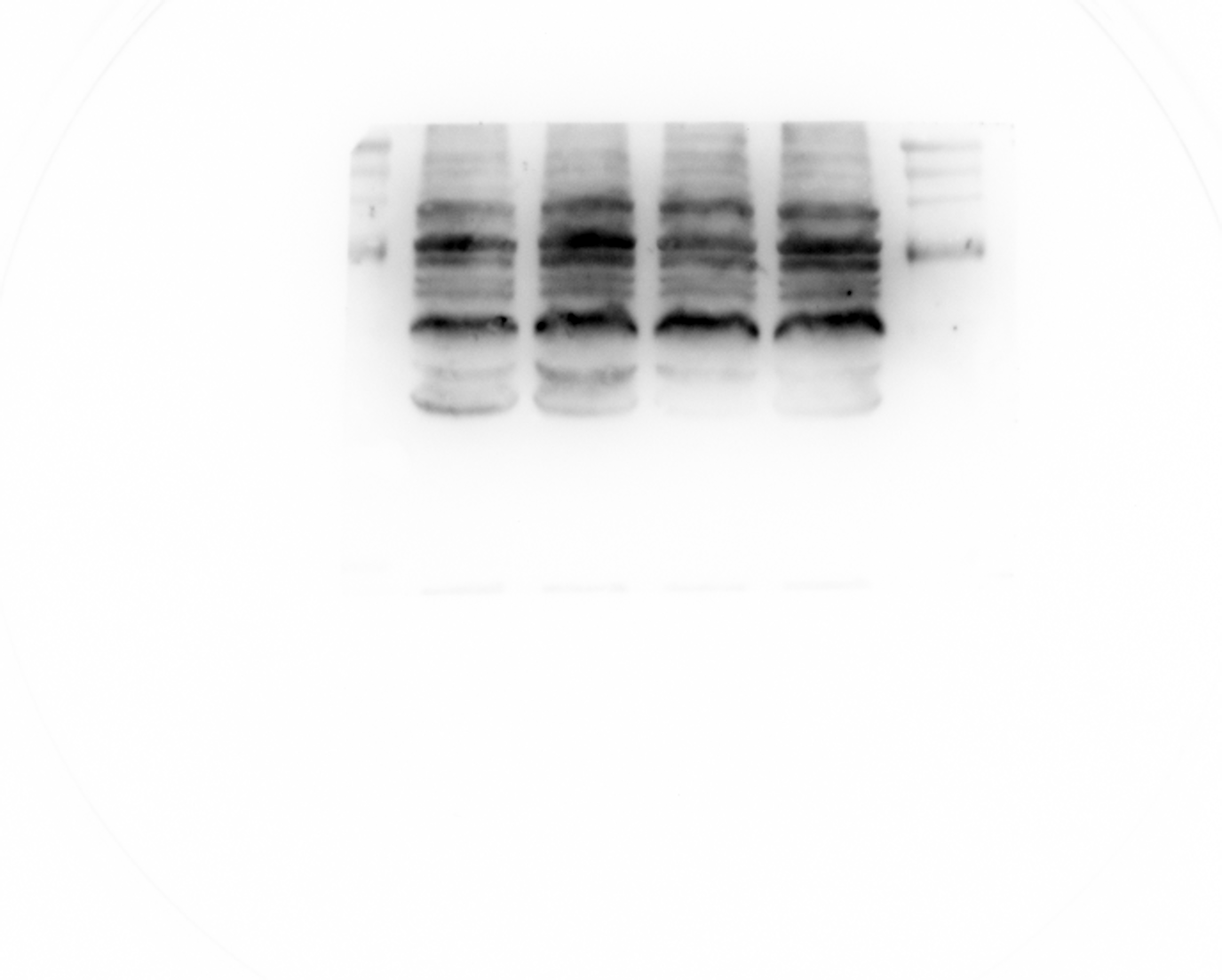

Supplement: Supplemental Information 9 [file peerj-13-19276-s009.zip › western blot-Total Cx43 EB1 N-cadherin 1/8-Tubulin.Tif]

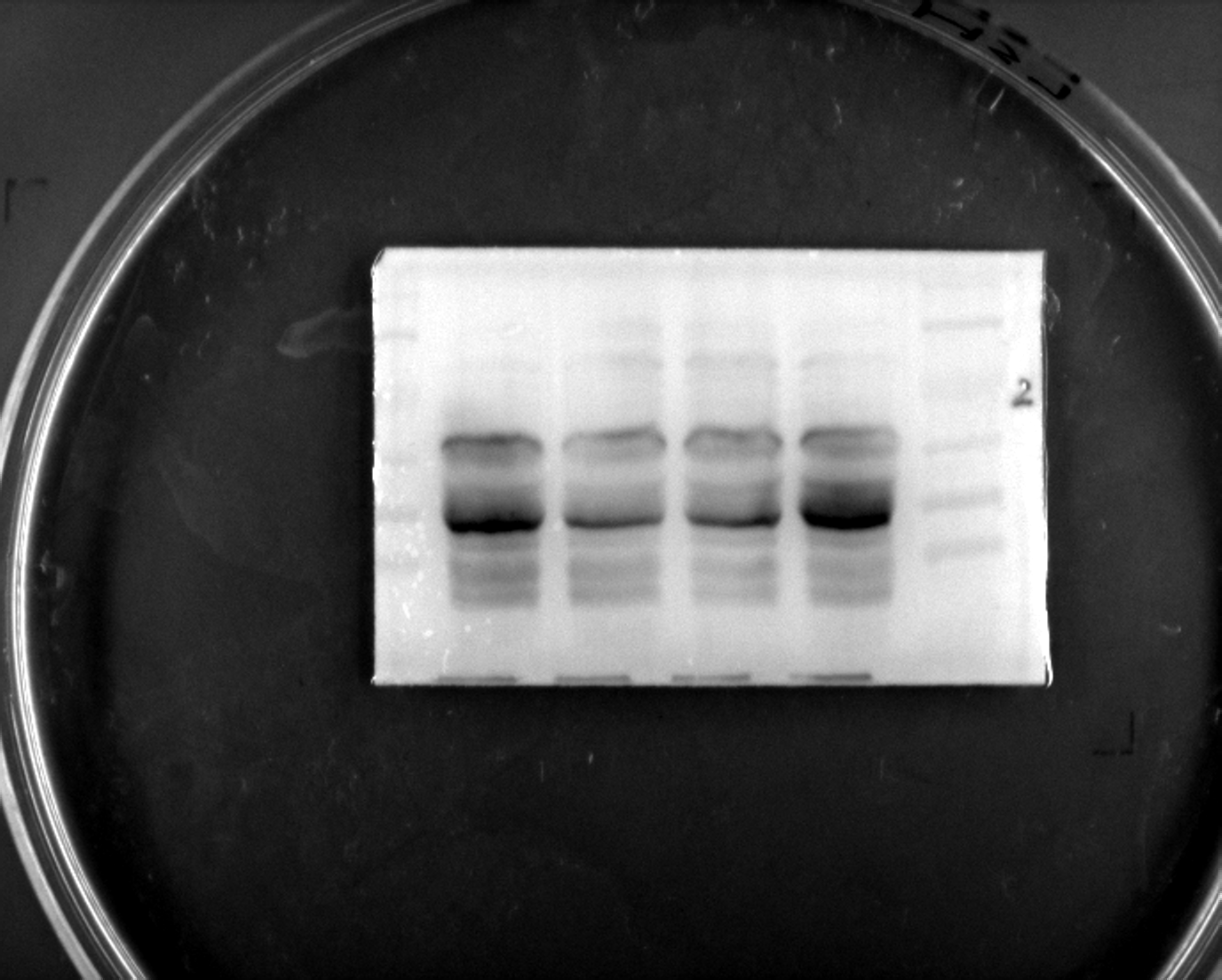

Supplement: Supplemental Information 10 [file peerj-13-19276-s010.zip › western blot-Total Cx43 EB1 N-cadherin 2/1-CX43-M.Tif]

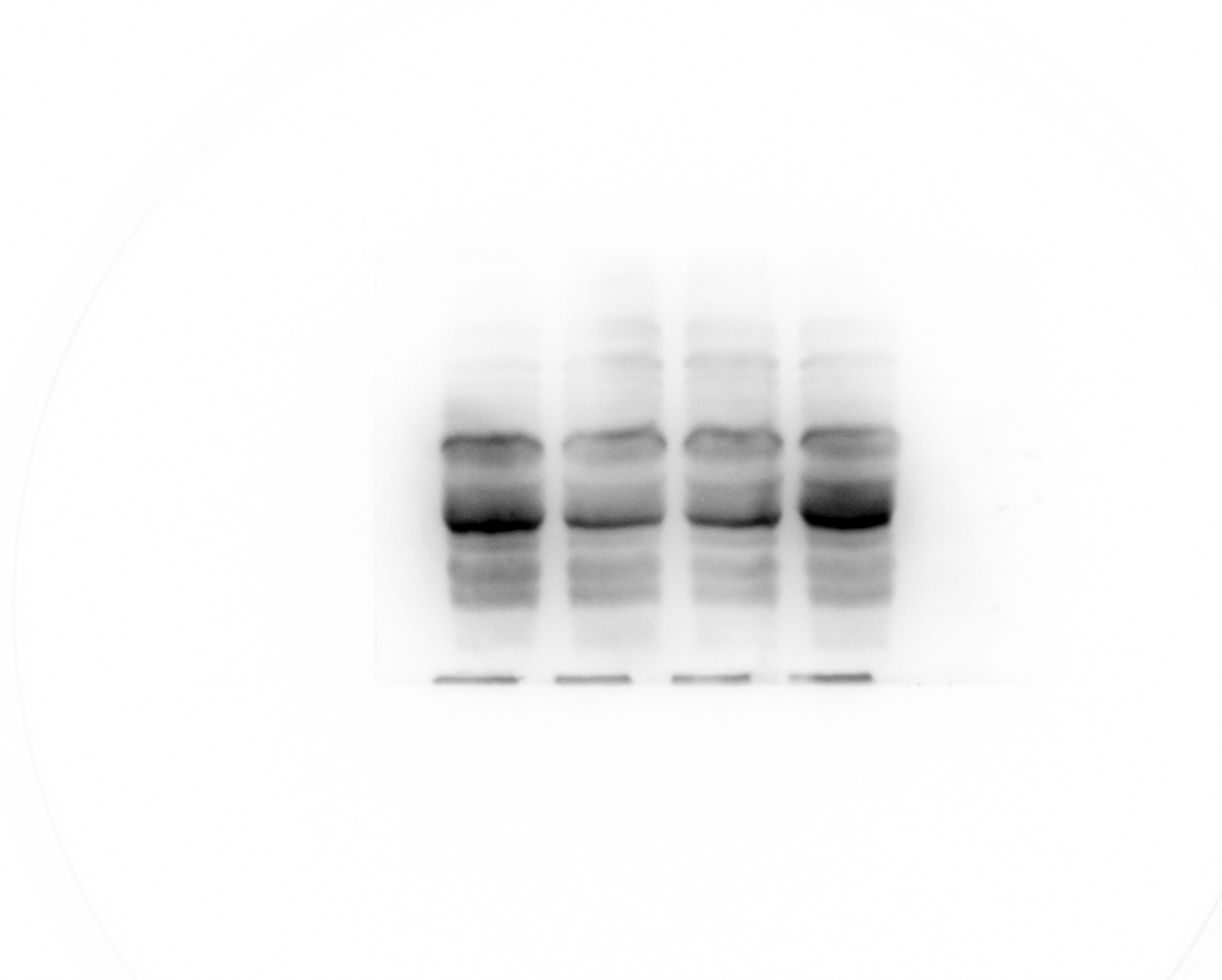

Supplement: Supplemental Information 10 [file peerj-13-19276-s010.zip › western blot-Total Cx43 EB1 N-cadherin 2/1-CX43.Tif]

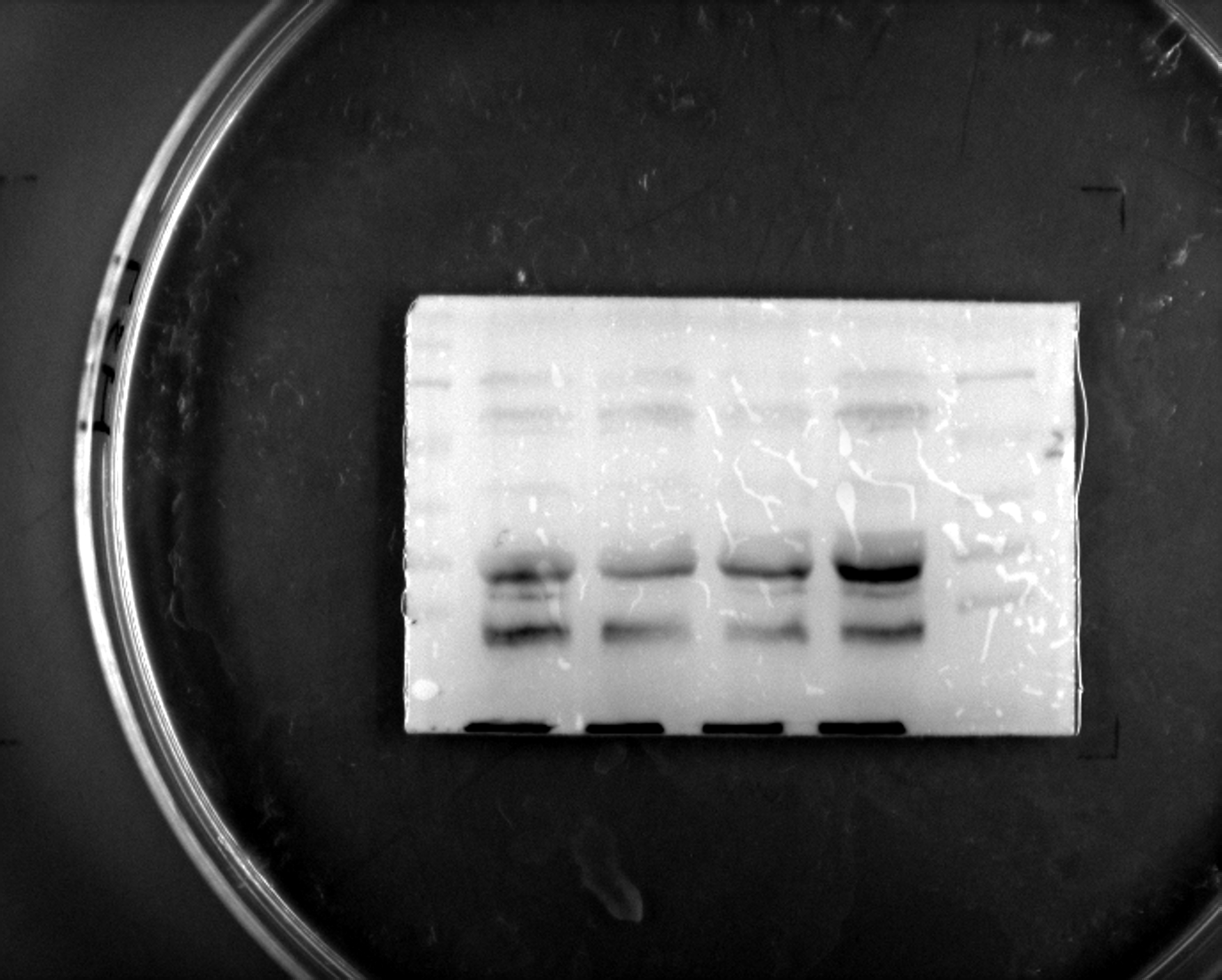

Supplement: Supplemental Information 10 [file peerj-13-19276-s010.zip › western blot-Total Cx43 EB1 N-cadherin 2/1-EB1-M.Tif]

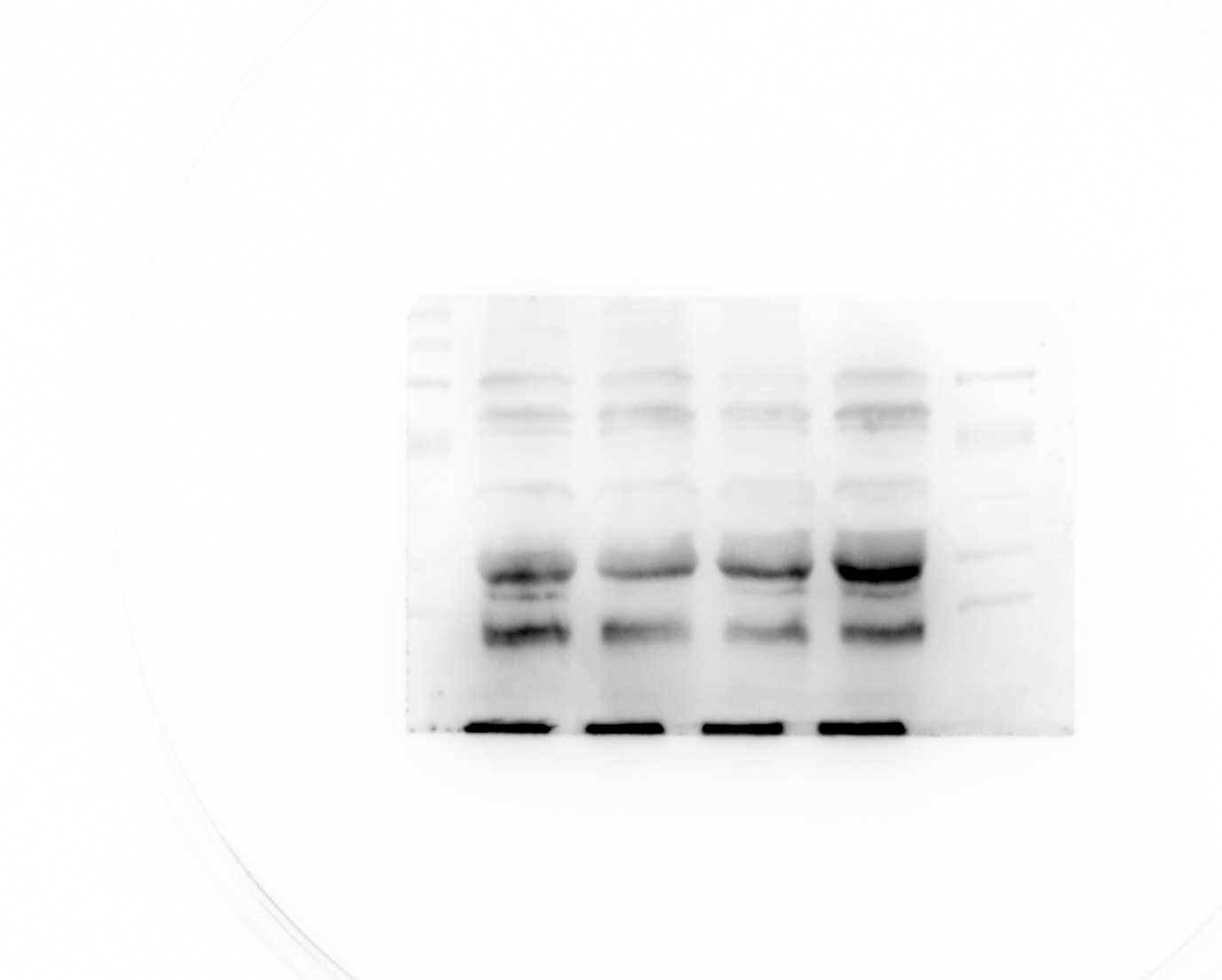

Supplement: Supplemental Information 10 [file peerj-13-19276-s010.zip › western blot-Total Cx43 EB1 N-cadherin 2/1-EB1.Tif]

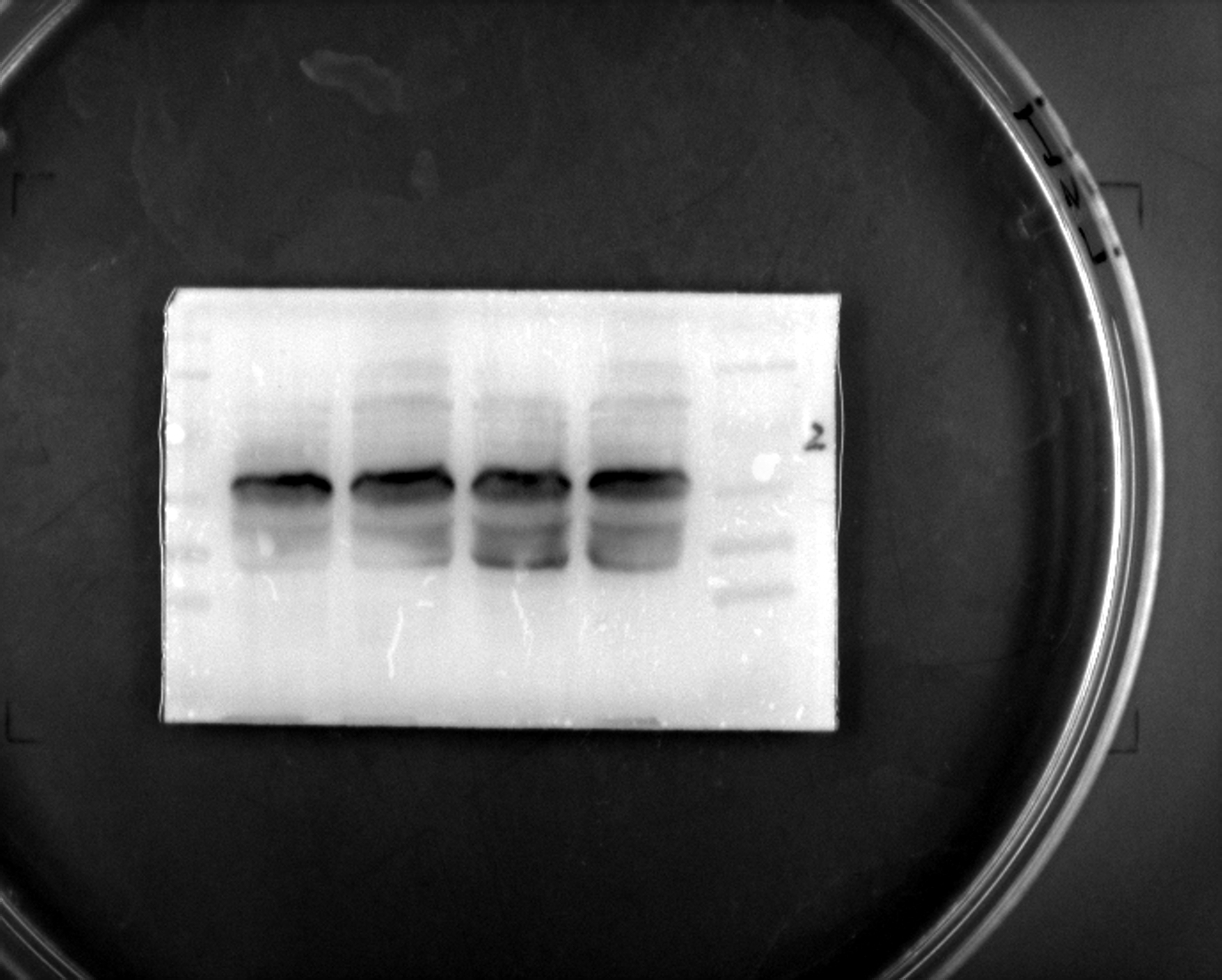

Supplement: Supplemental Information 10 [file peerj-13-19276-s010.zip › western blot-Total Cx43 EB1 N-cadherin 2/1-Tubulin-M.Tif]

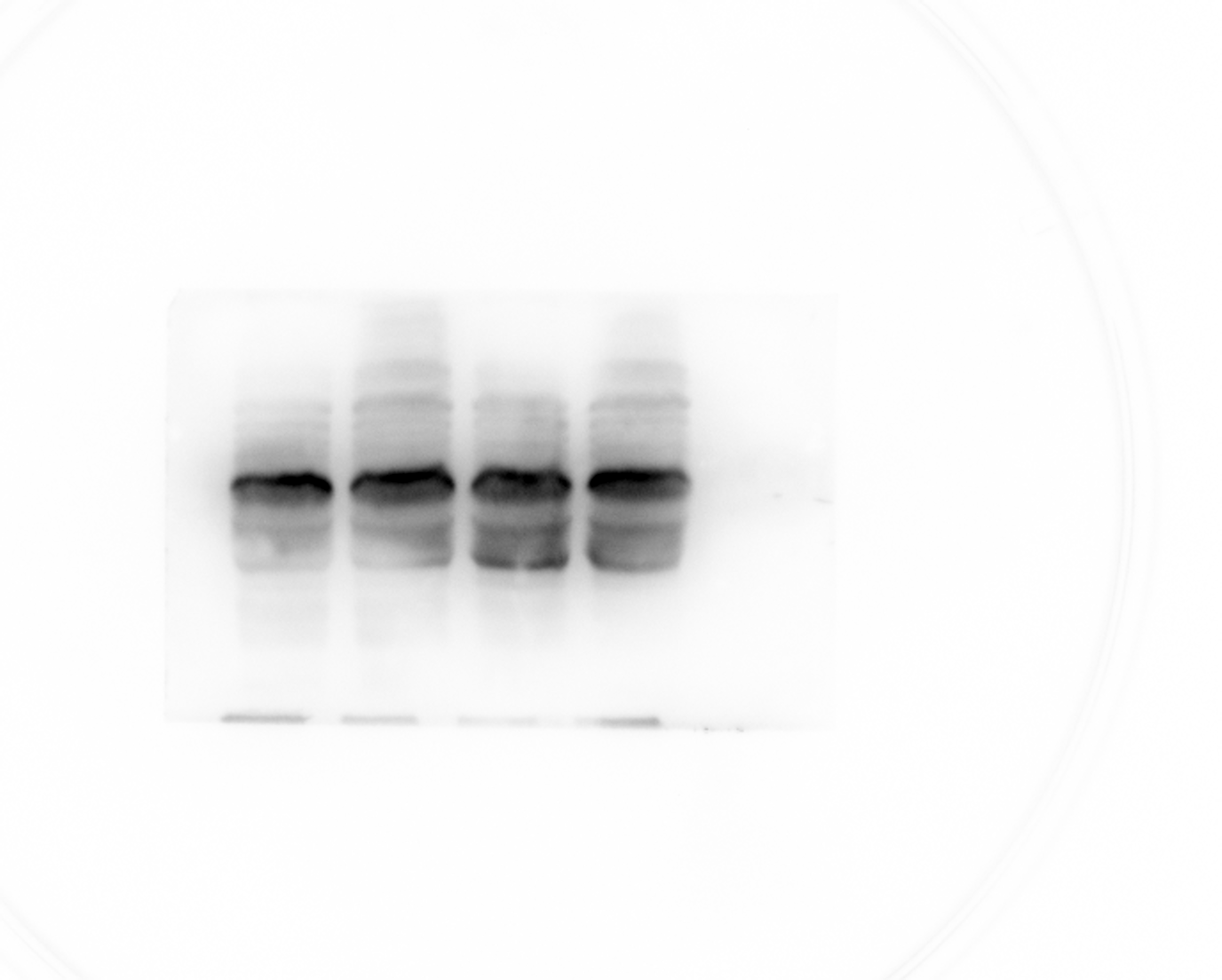

Supplement: Supplemental Information 10 [file peerj-13-19276-s010.zip › western blot-Total Cx43 EB1 N-cadherin 2/1-Tubulin.Tif]

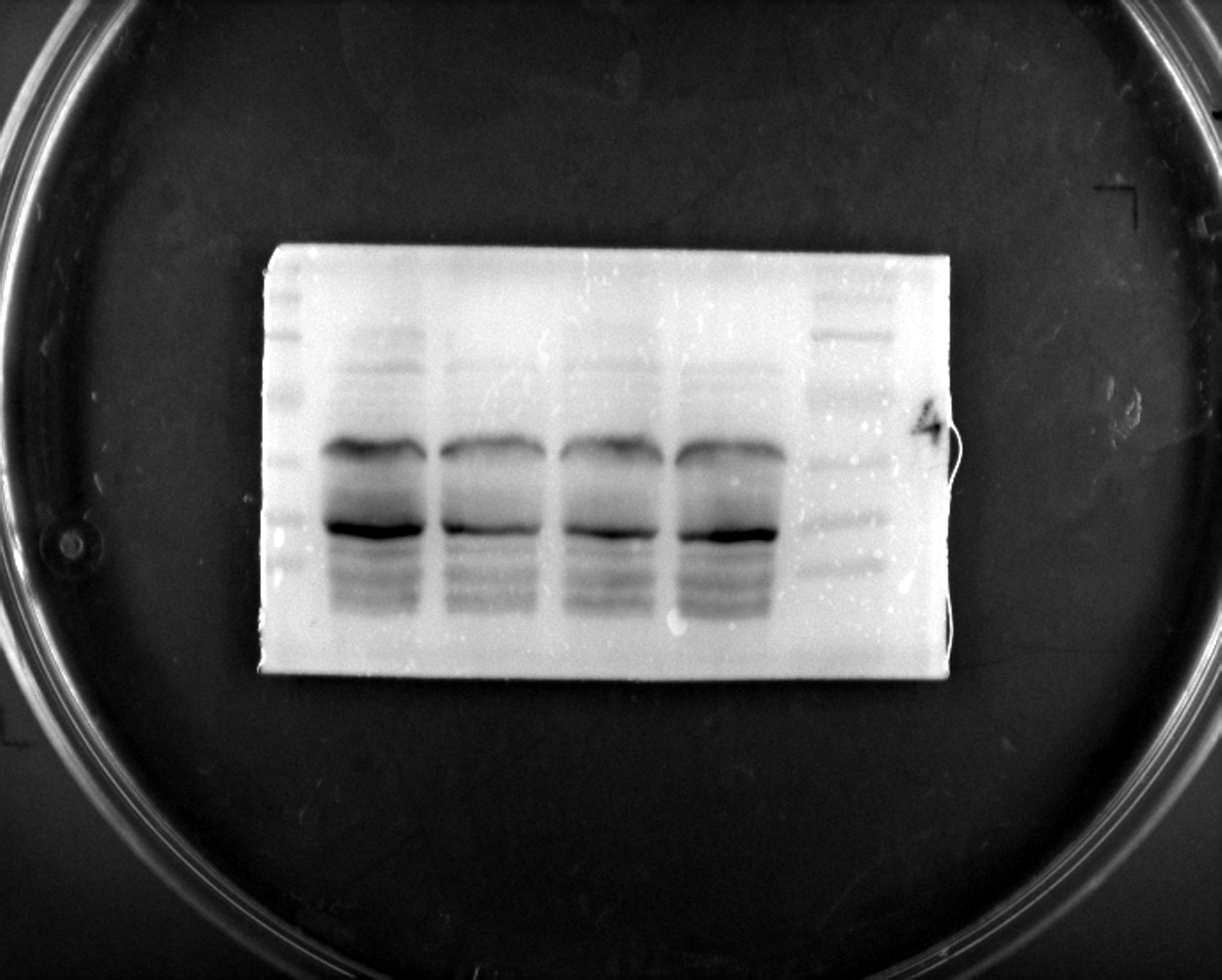

Supplement: Supplemental Information 10 [file peerj-13-19276-s010.zip › western blot-Total Cx43 EB1 N-cadherin 2/3-CX43-M.Tif]

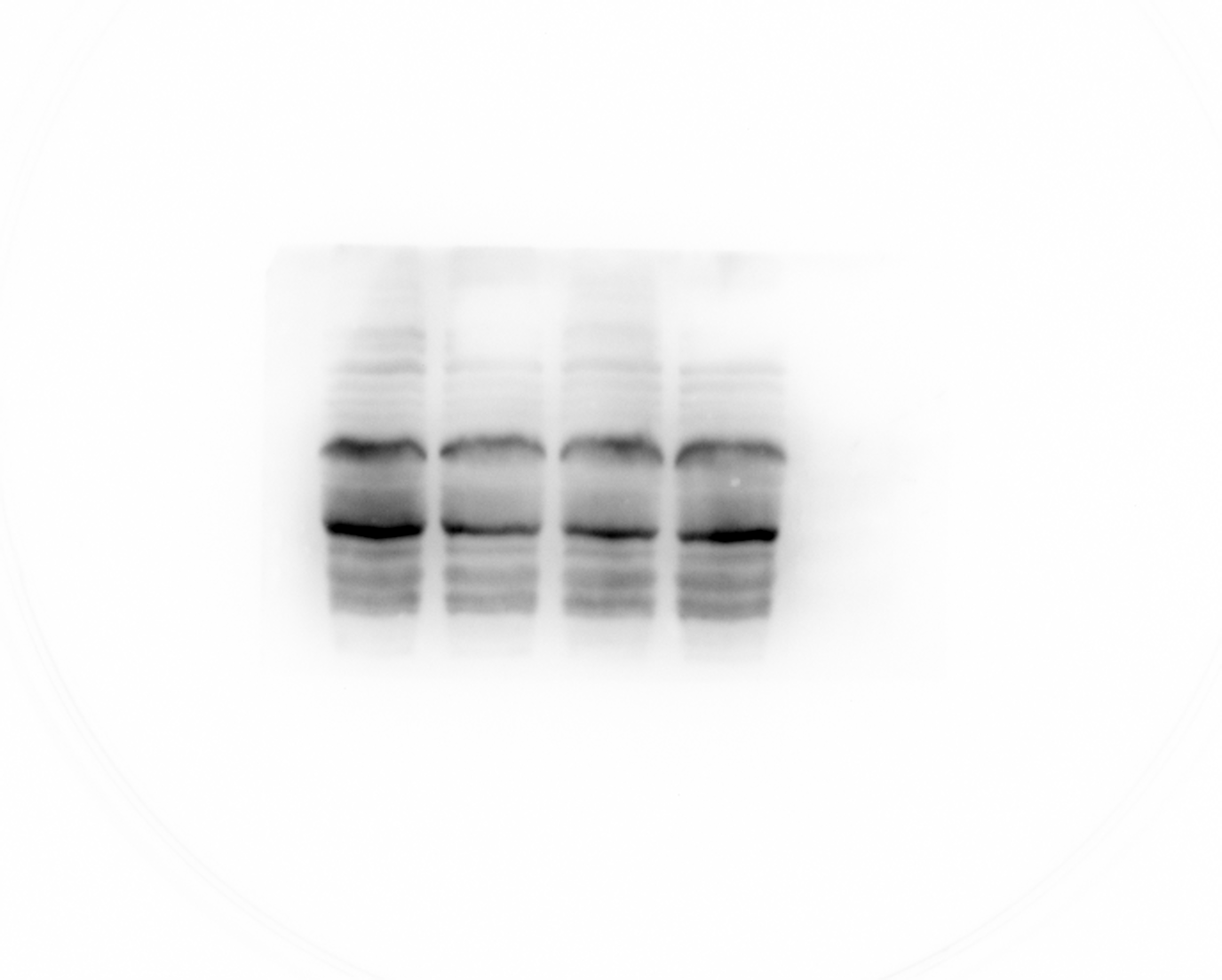

Supplement: Supplemental Information 10 [file peerj-13-19276-s010.zip › western blot-Total Cx43 EB1 N-cadherin 2/3-CX43.Tif]

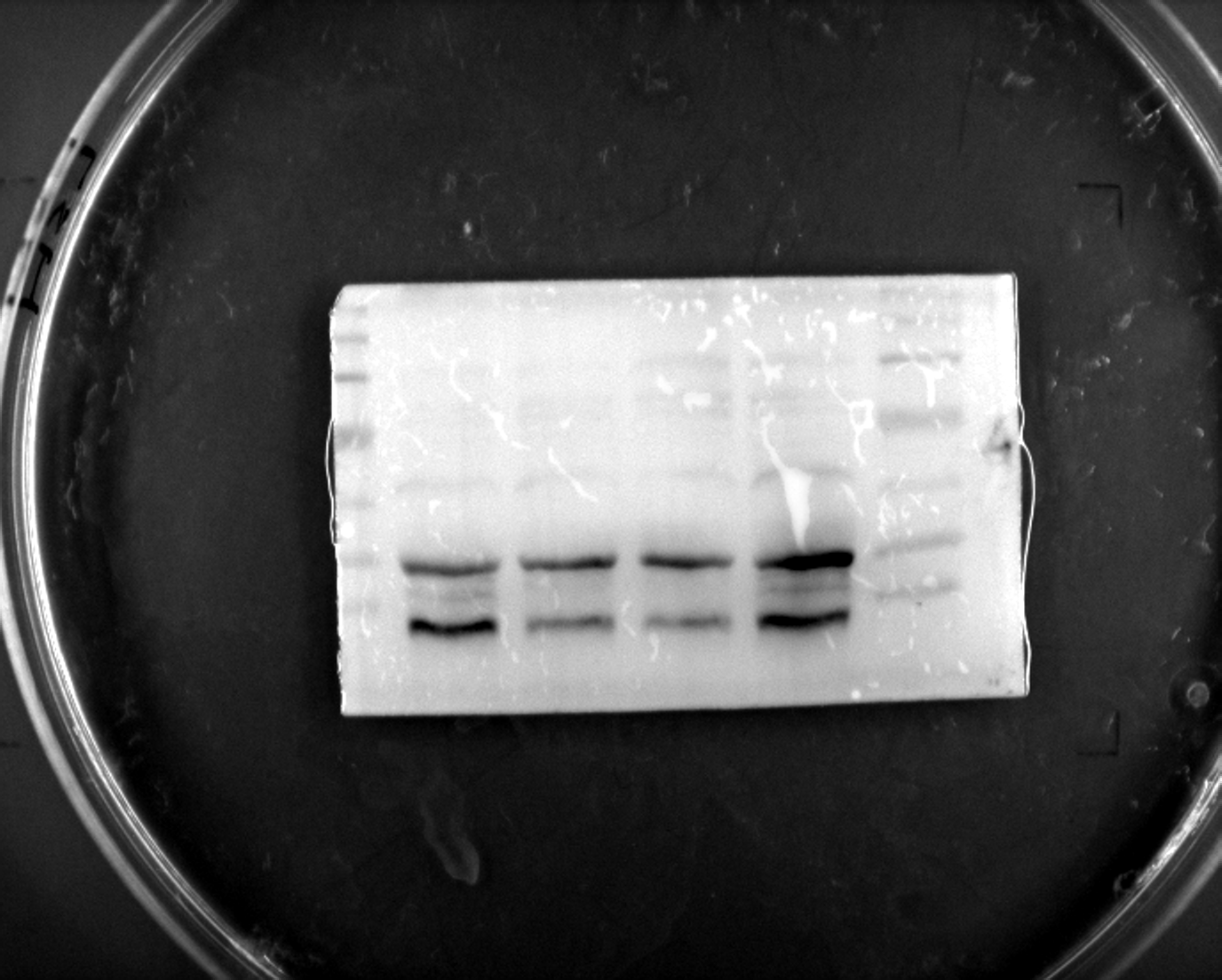

Supplement: Supplemental Information 10 [file peerj-13-19276-s010.zip › western blot-Total Cx43 EB1 N-cadherin 2/3-EB1-M.Tif]

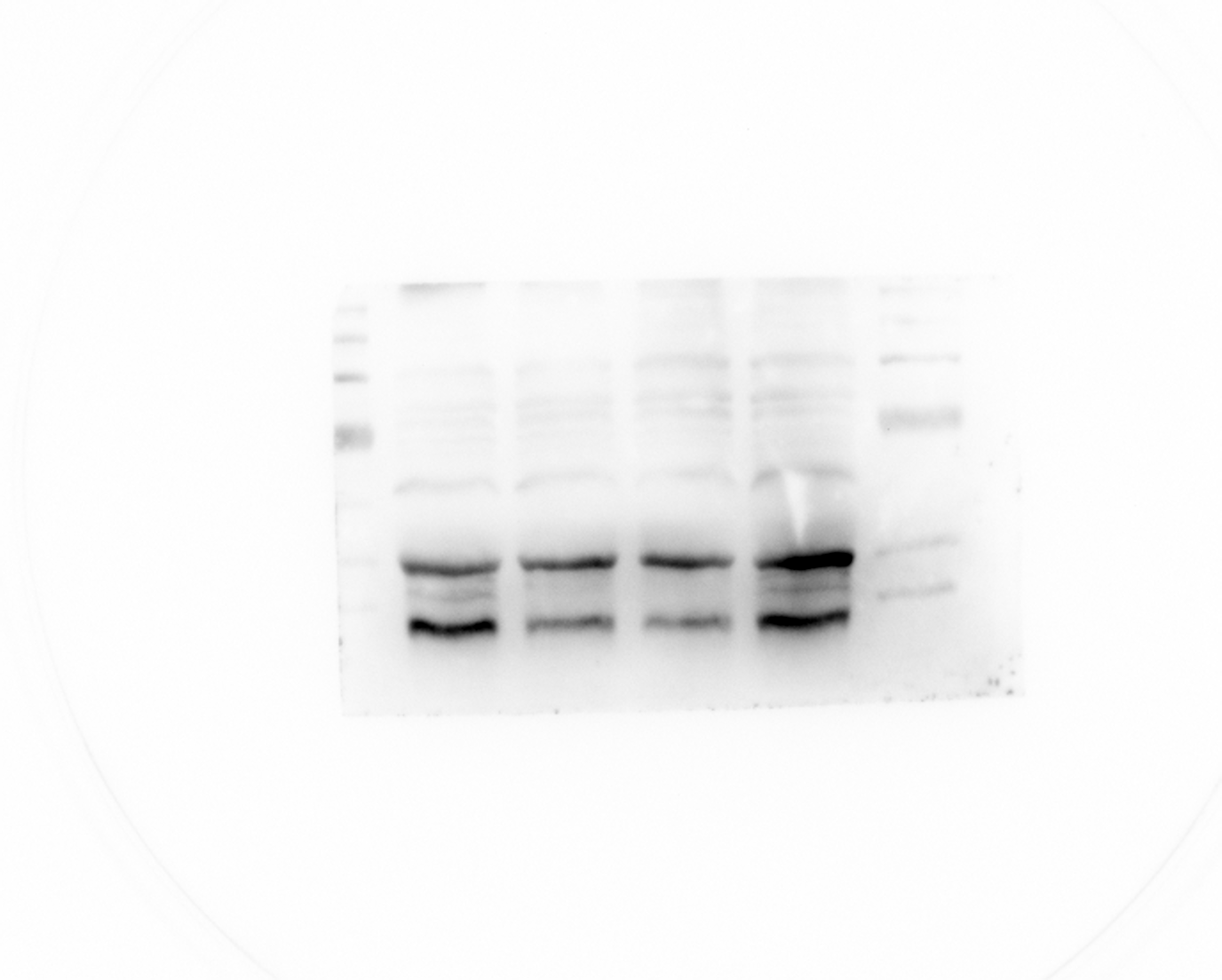

Supplement: Supplemental Information 10 [file peerj-13-19276-s010.zip › western blot-Total Cx43 EB1 N-cadherin 2/3-EB1.Tif]

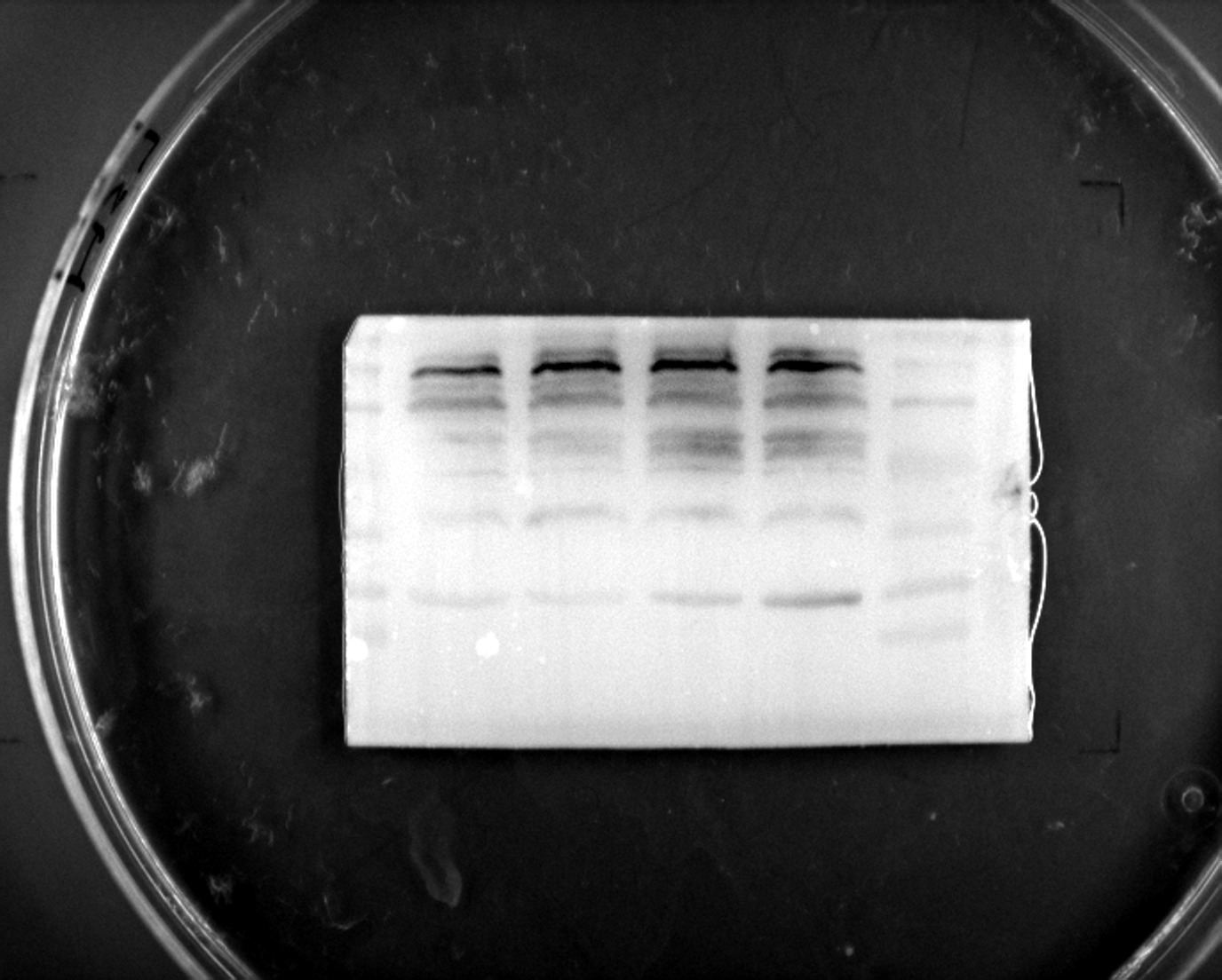

Supplement: Supplemental Information 10 [file peerj-13-19276-s010.zip › western blot-Total Cx43 EB1 N-cadherin 2/3-N-cadherin-M.Tif]

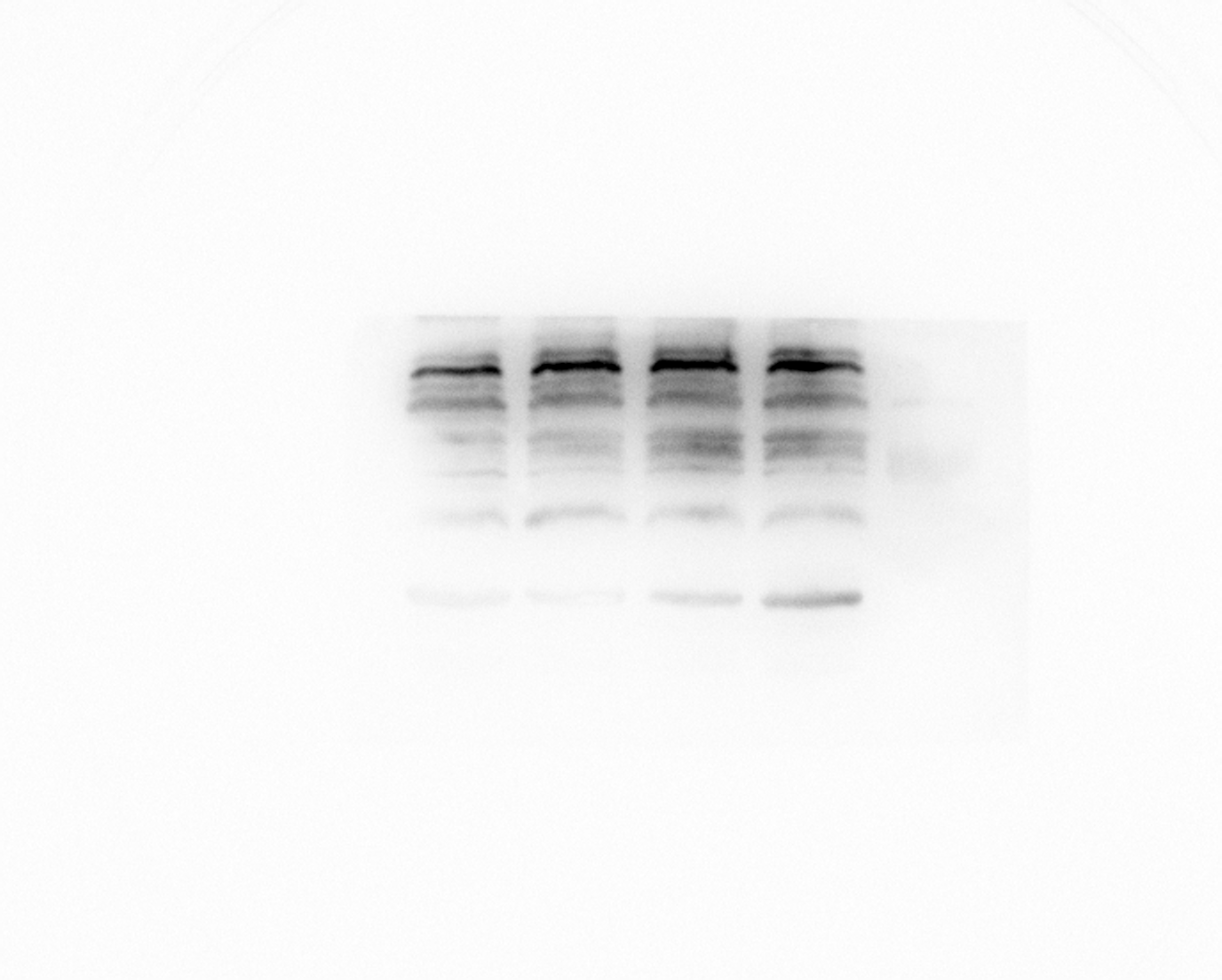

Supplement: Supplemental Information 10 [file peerj-13-19276-s010.zip › western blot-Total Cx43 EB1 N-cadherin 2/3-N-cadherin.Tif]

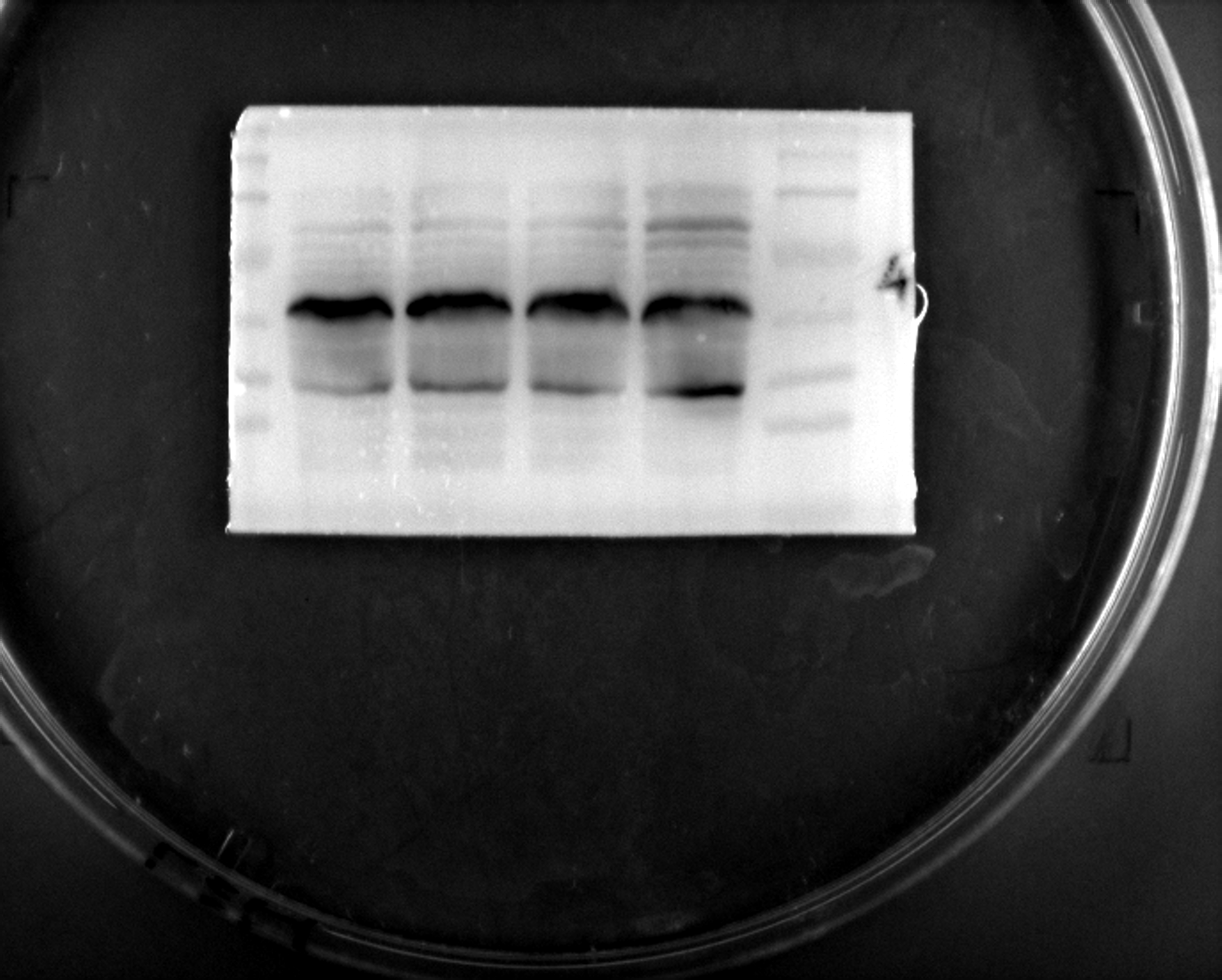

Supplement: Supplemental Information 10 [file peerj-13-19276-s010.zip › western blot-Total Cx43 EB1 N-cadherin 2/3-Tubulin-M.Tif]

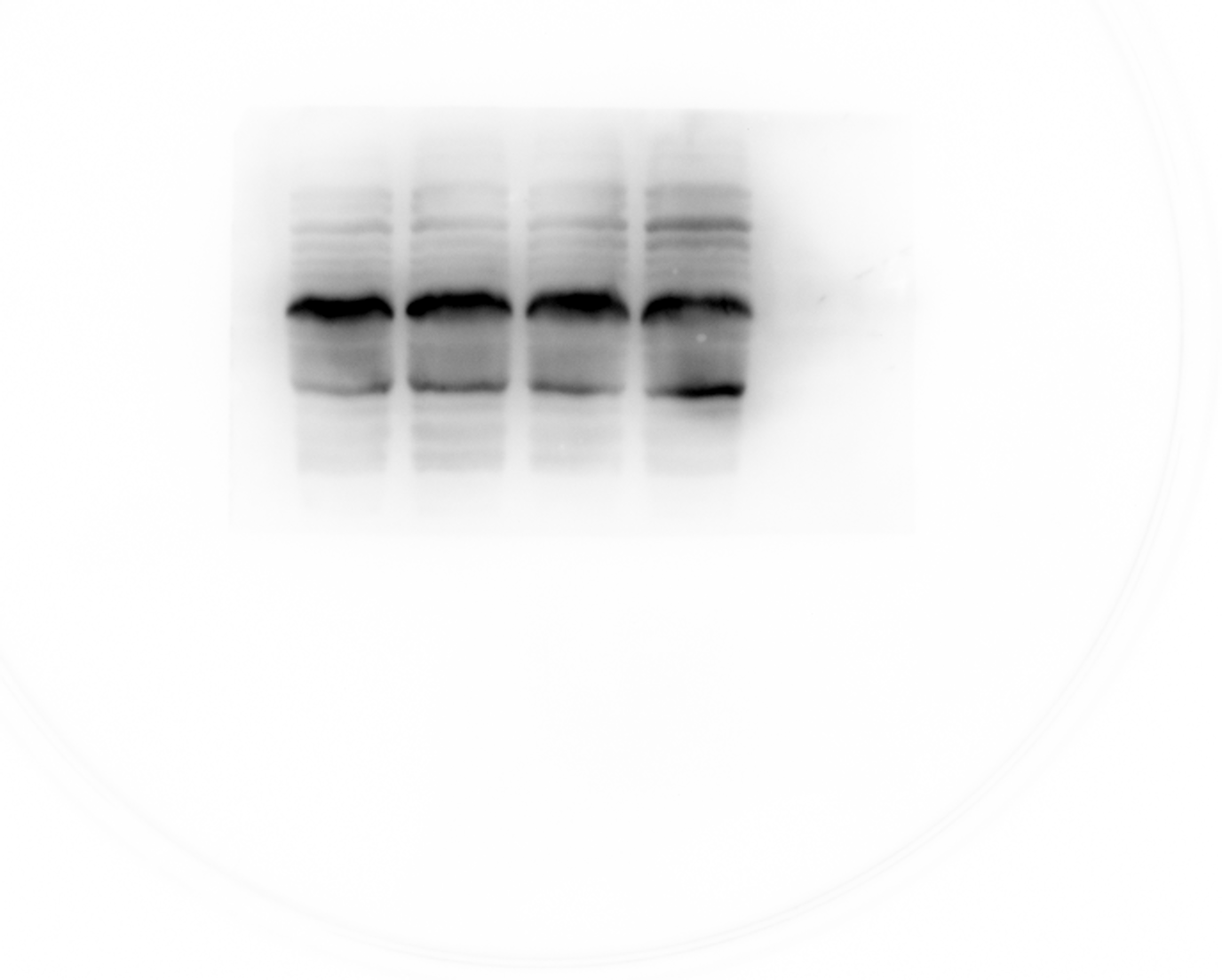

Supplement: Supplemental Information 10 [file peerj-13-19276-s010.zip › western blot-Total Cx43 EB1 N-cadherin 2/3-Tubulin.Tif]

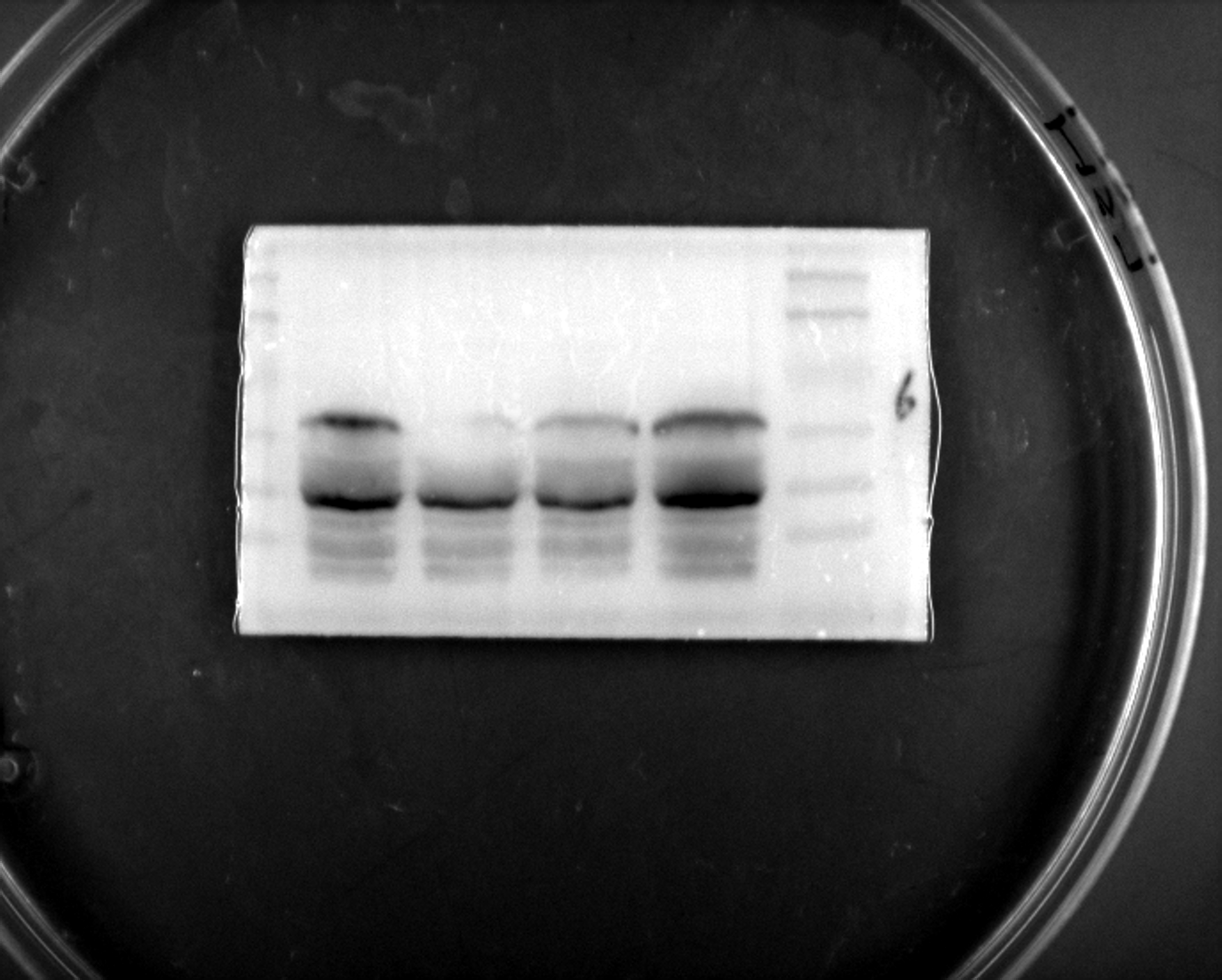

Supplement: Supplemental Information 10 [file peerj-13-19276-s010.zip › western blot-Total Cx43 EB1 N-cadherin 2/5-CX43-M.Tif]

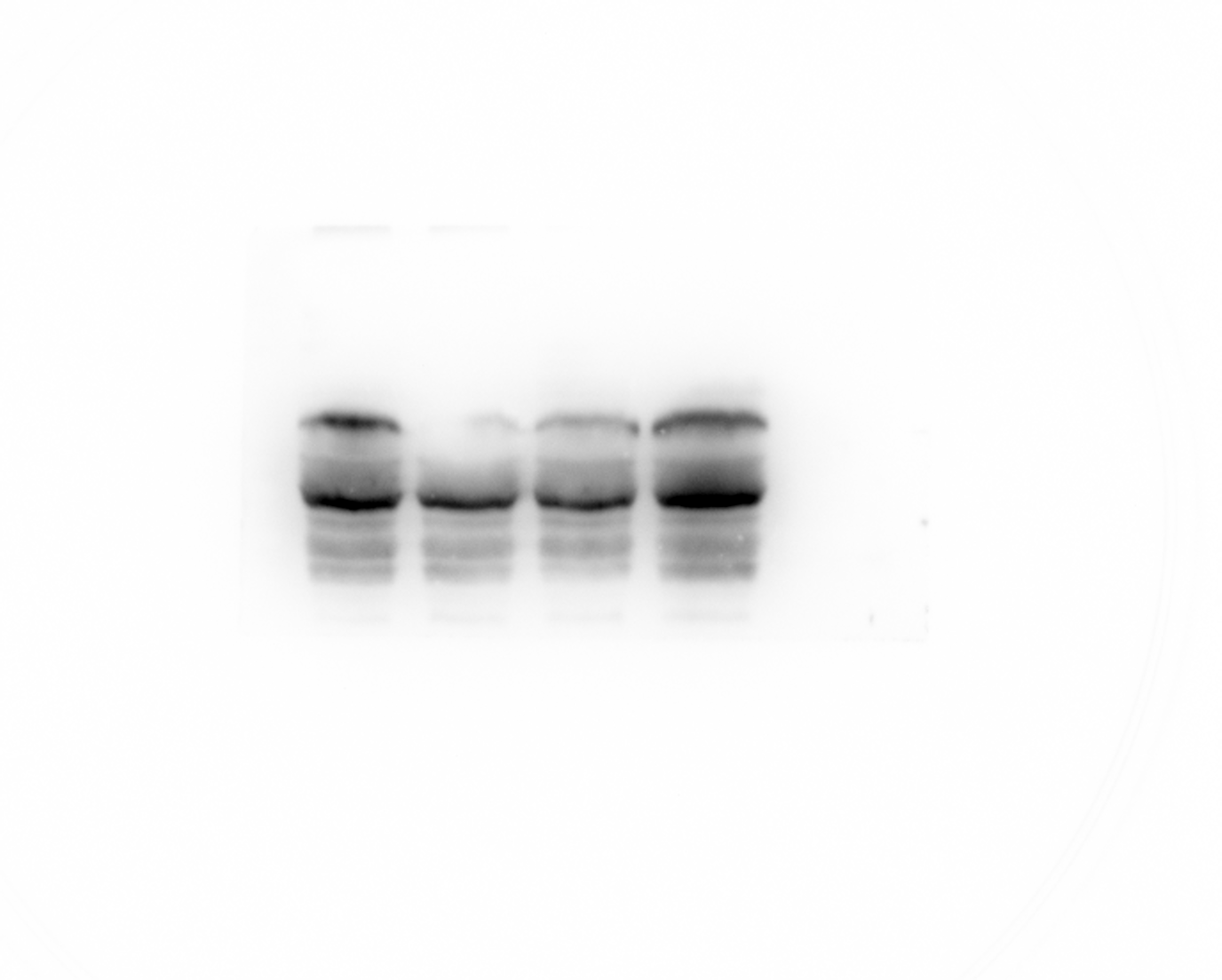

Supplement: Supplemental Information 10 [file peerj-13-19276-s010.zip › western blot-Total Cx43 EB1 N-cadherin 2/5-CX43.Tif]

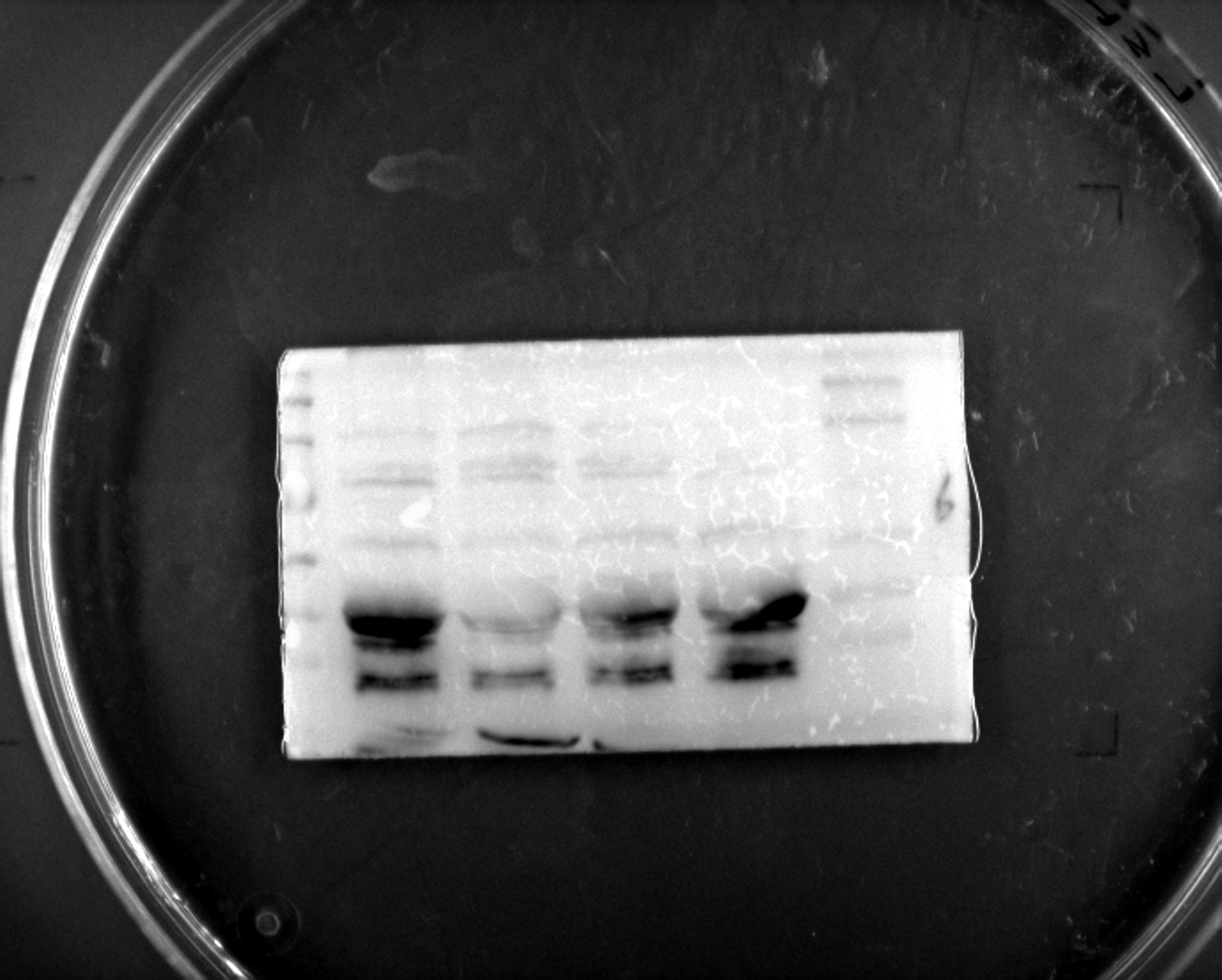

Supplement: Supplemental Information 10 [file peerj-13-19276-s010.zip › western blot-Total Cx43 EB1 N-cadherin 2/5-EB1-M.Tif]

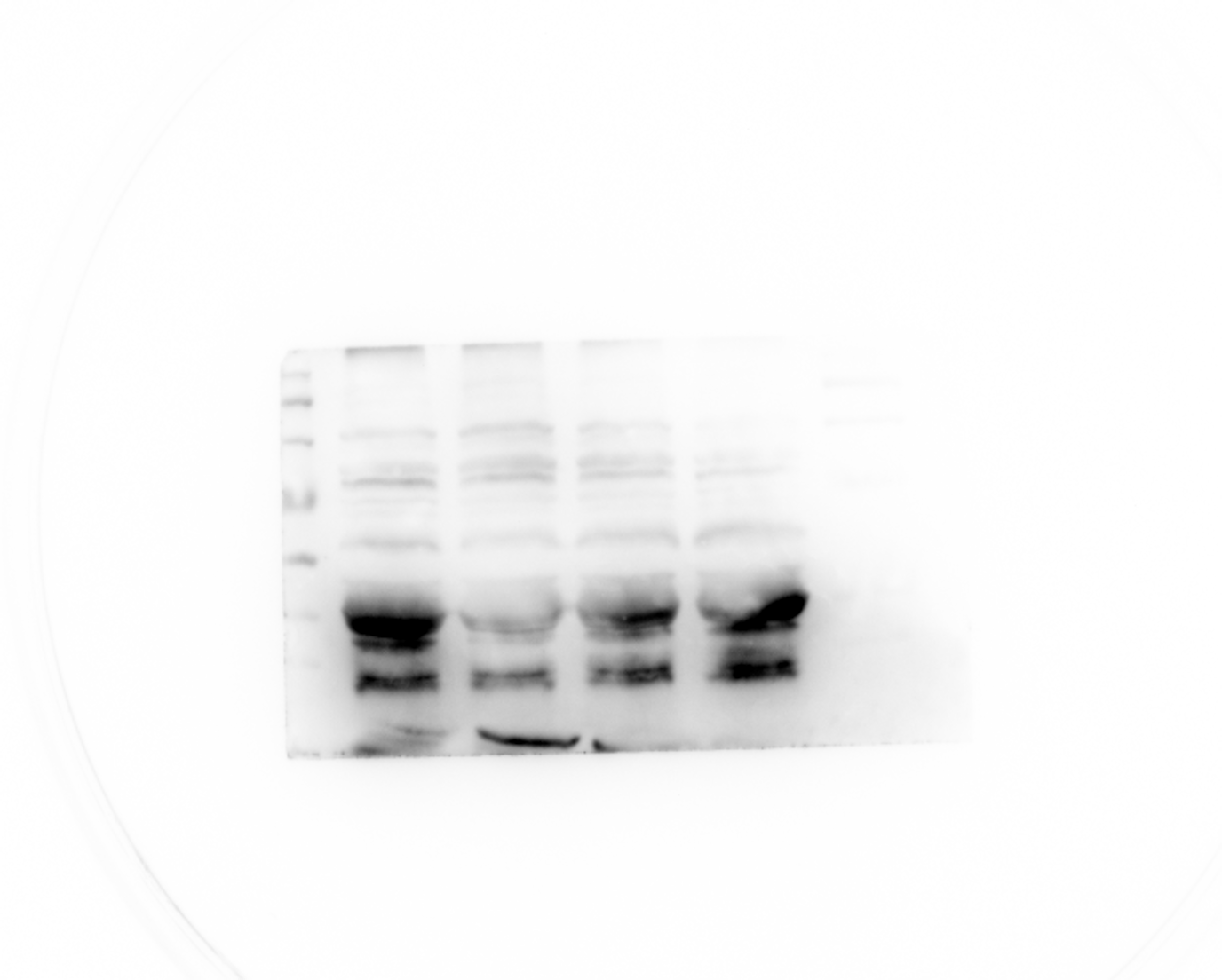

Supplement: Supplemental Information 10 [file peerj-13-19276-s010.zip › western blot-Total Cx43 EB1 N-cadherin 2/5-EB1.Tif]

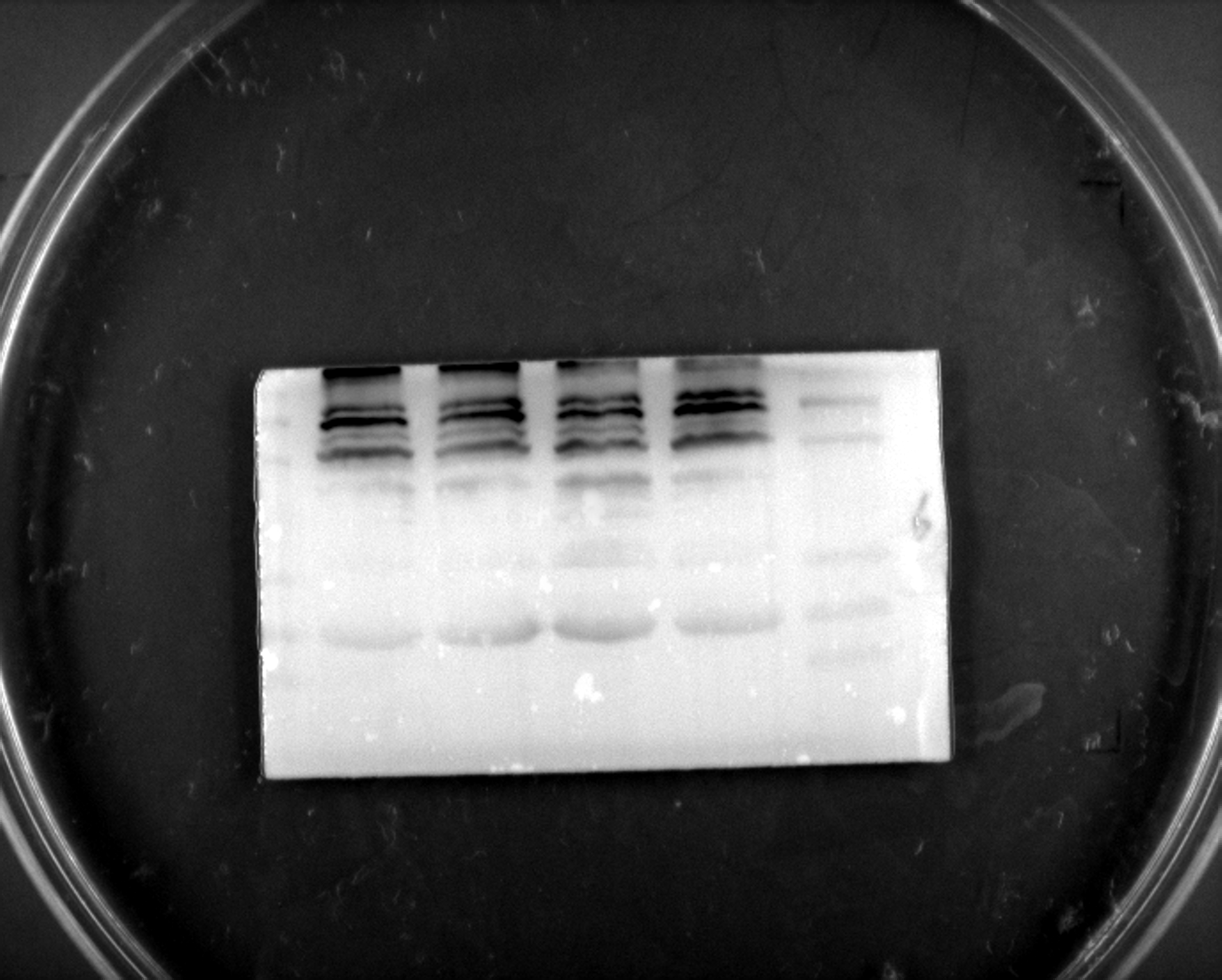

Supplement: Supplemental Information 10 [file peerj-13-19276-s010.zip › western blot-Total Cx43 EB1 N-cadherin 2/5-N-cadherin-M.Tif]

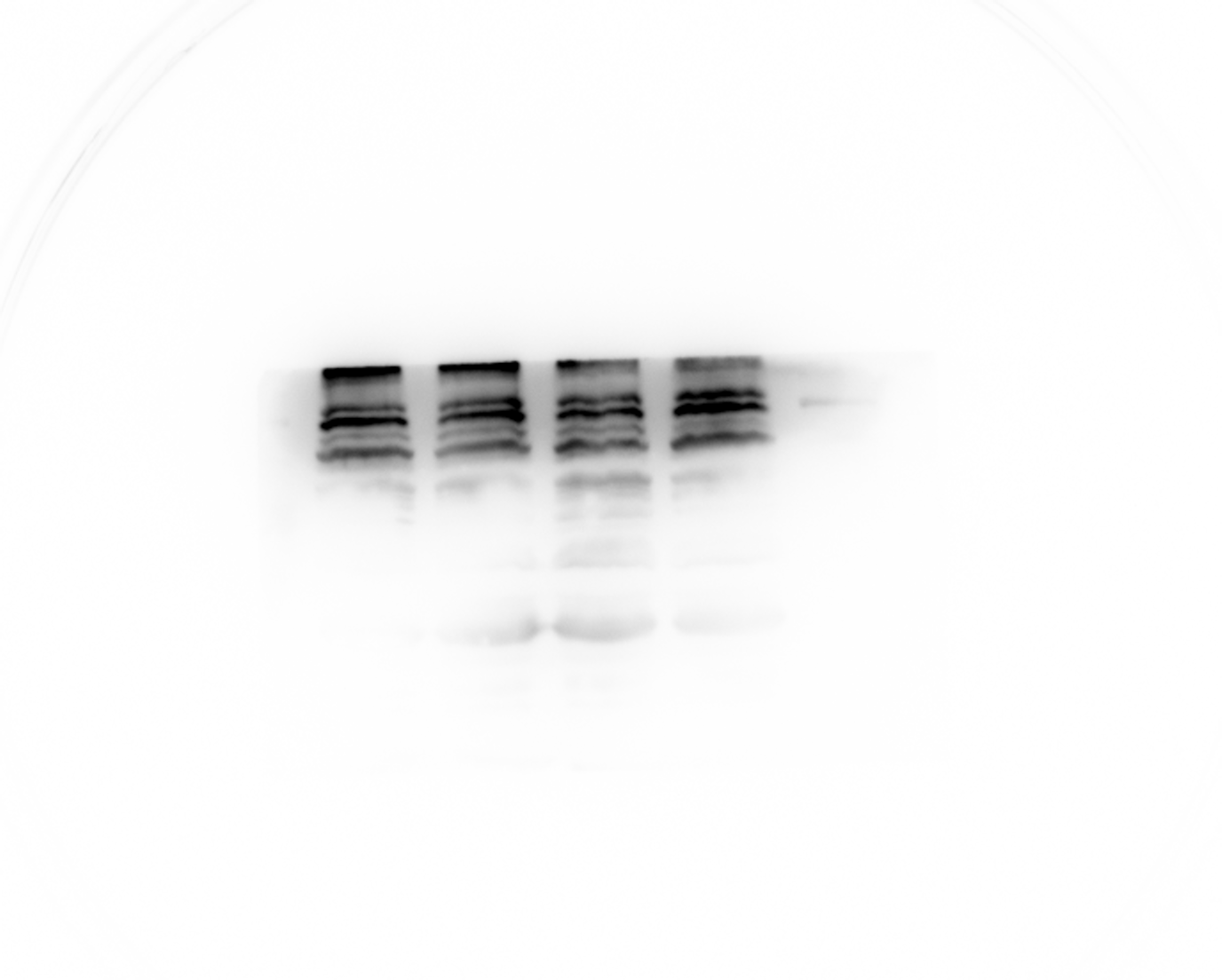

Supplement: Supplemental Information 10 [file peerj-13-19276-s010.zip › western blot-Total Cx43 EB1 N-cadherin 2/5-N-cadherin.Tif]
