# Supplementary figures and images for: Downregulation of EB1 impedes Cx43 localization and cardiac conduction after hypothermic ischemia-reperfusion in rats (part 4 of 5)
Source: PeerJ. 2025 Apr 14;13:e19276. doi: 10.7717/peerj.19276 (PMC12005192; doi:10.7717/peerj.19276)

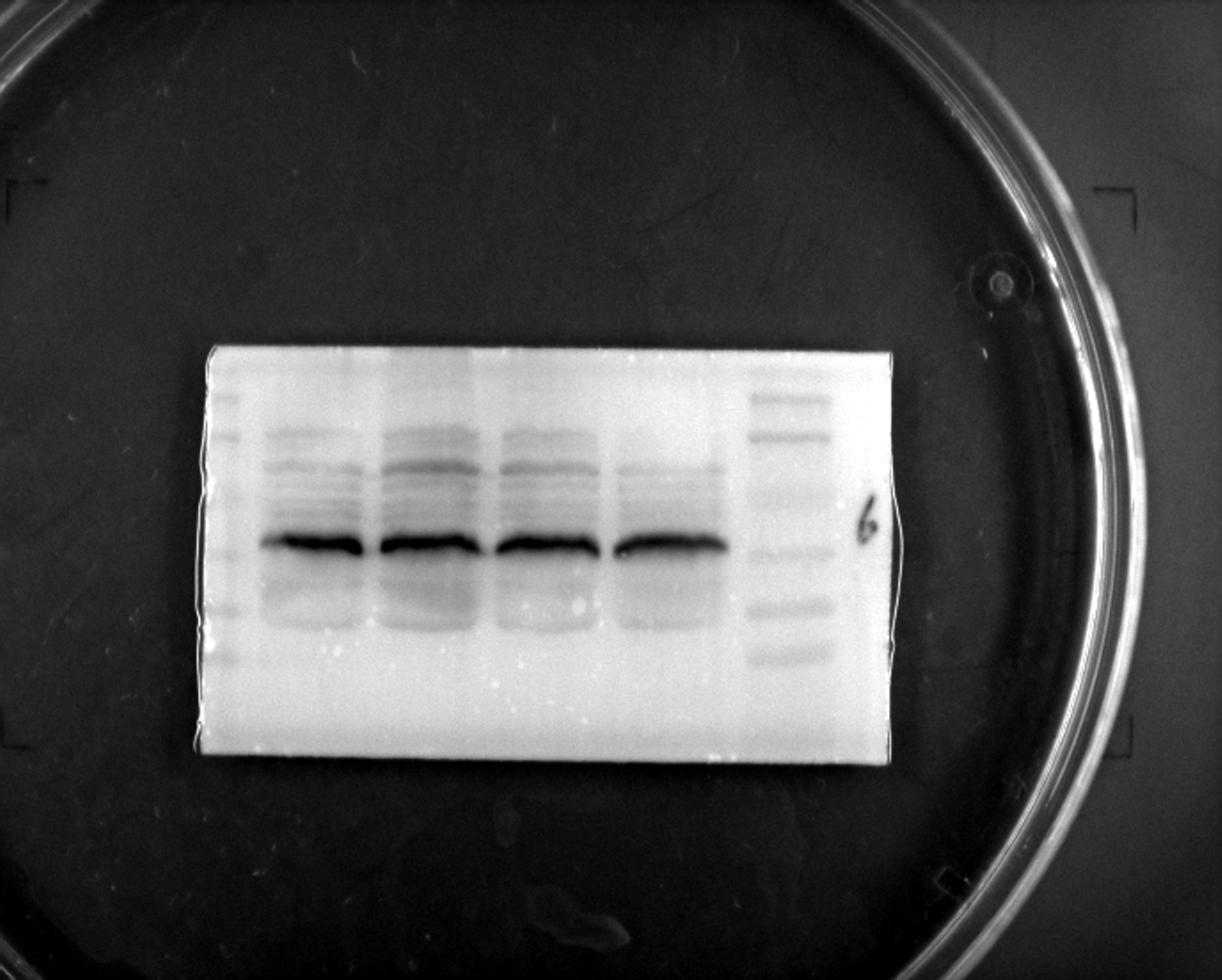

Supplement: Supplemental Information 10 [file peerj-13-19276-s010.zip › western blot-Total Cx43 EB1 N-cadherin 2/5-Tubulin-M.Tif]

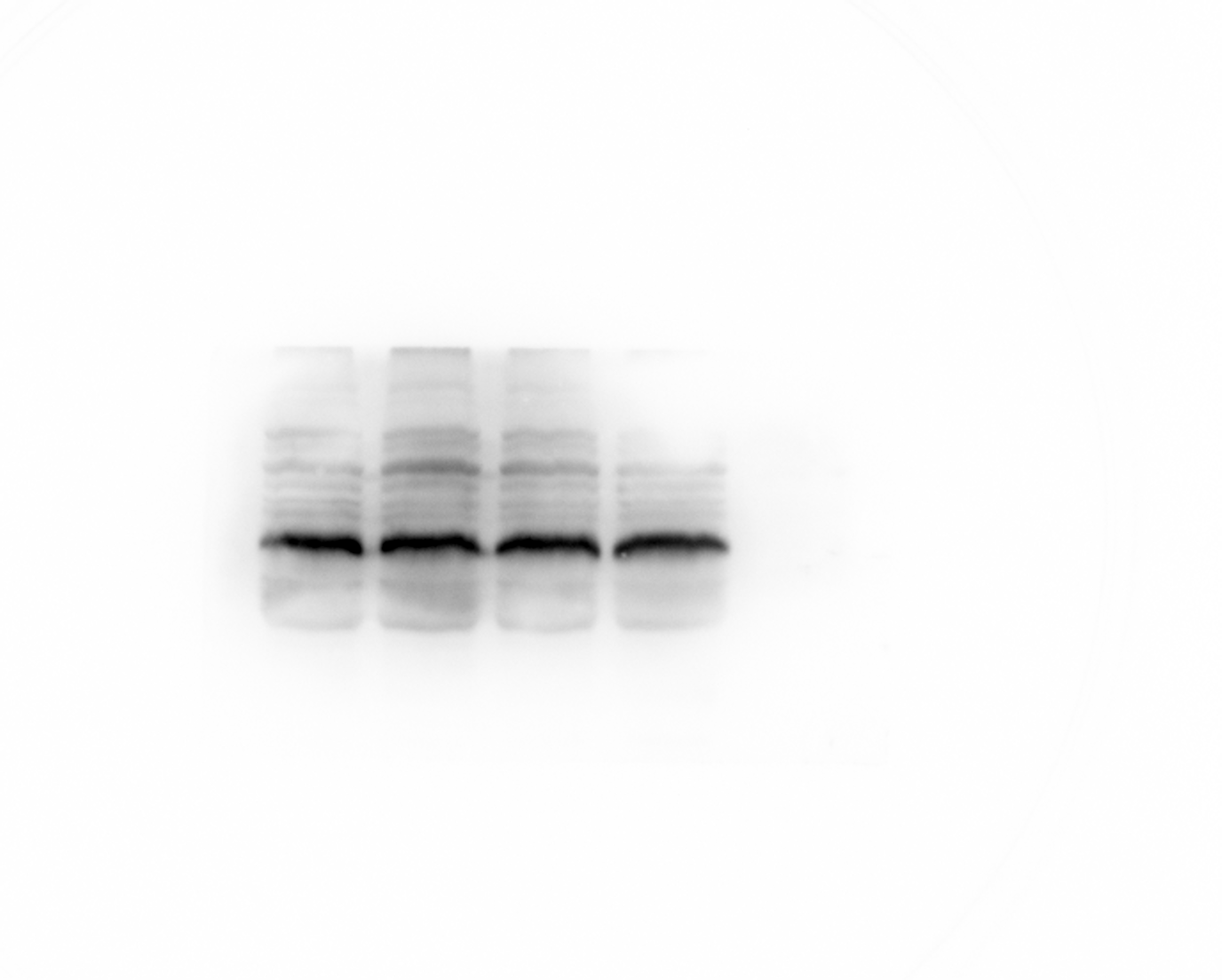

Supplement: Supplemental Information 10 [file peerj-13-19276-s010.zip › western blot-Total Cx43 EB1 N-cadherin 2/5-Tubulin.Tif]

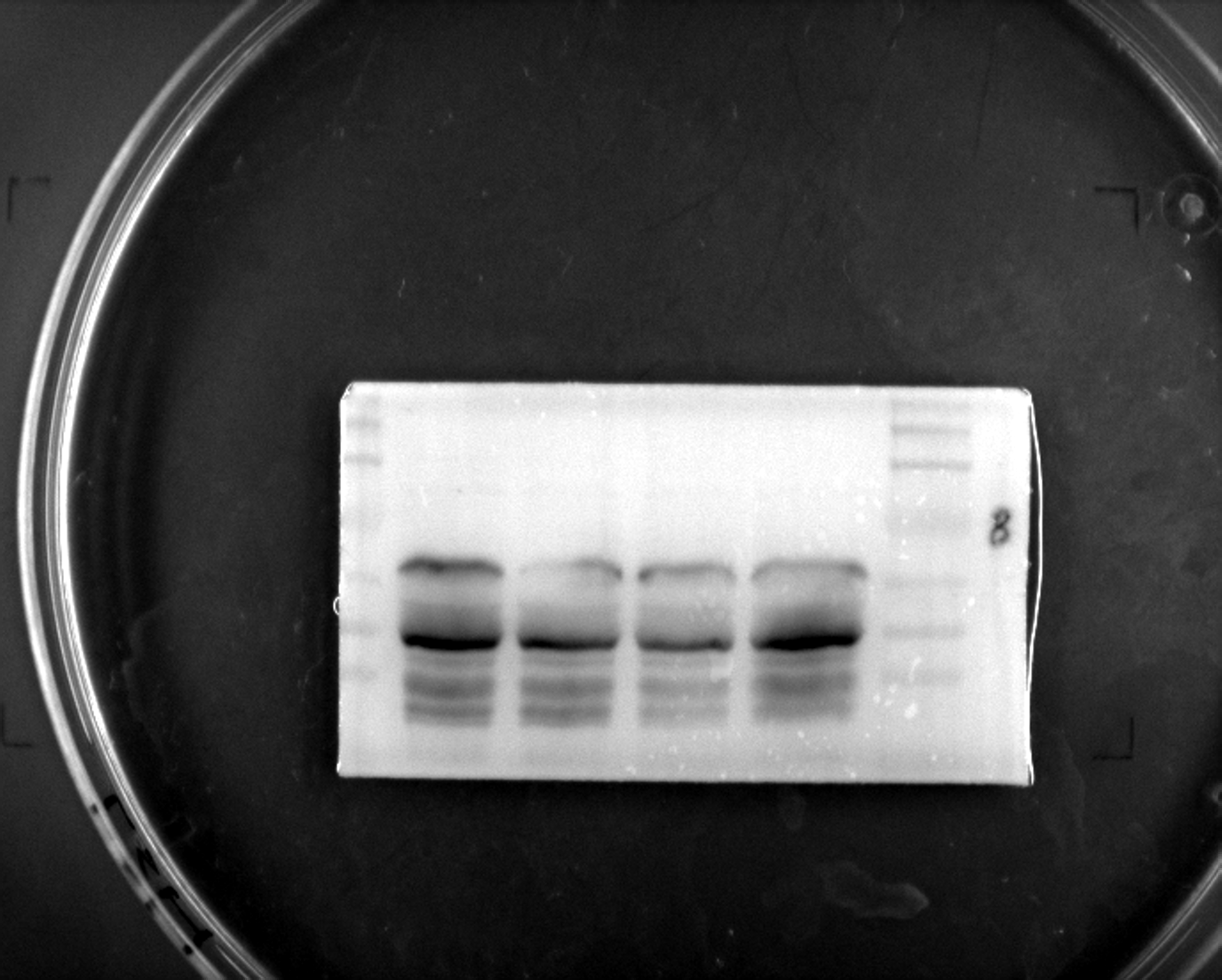

Supplement: Supplemental Information 10 [file peerj-13-19276-s010.zip › western blot-Total Cx43 EB1 N-cadherin 2/7-CX43-M.Tif]

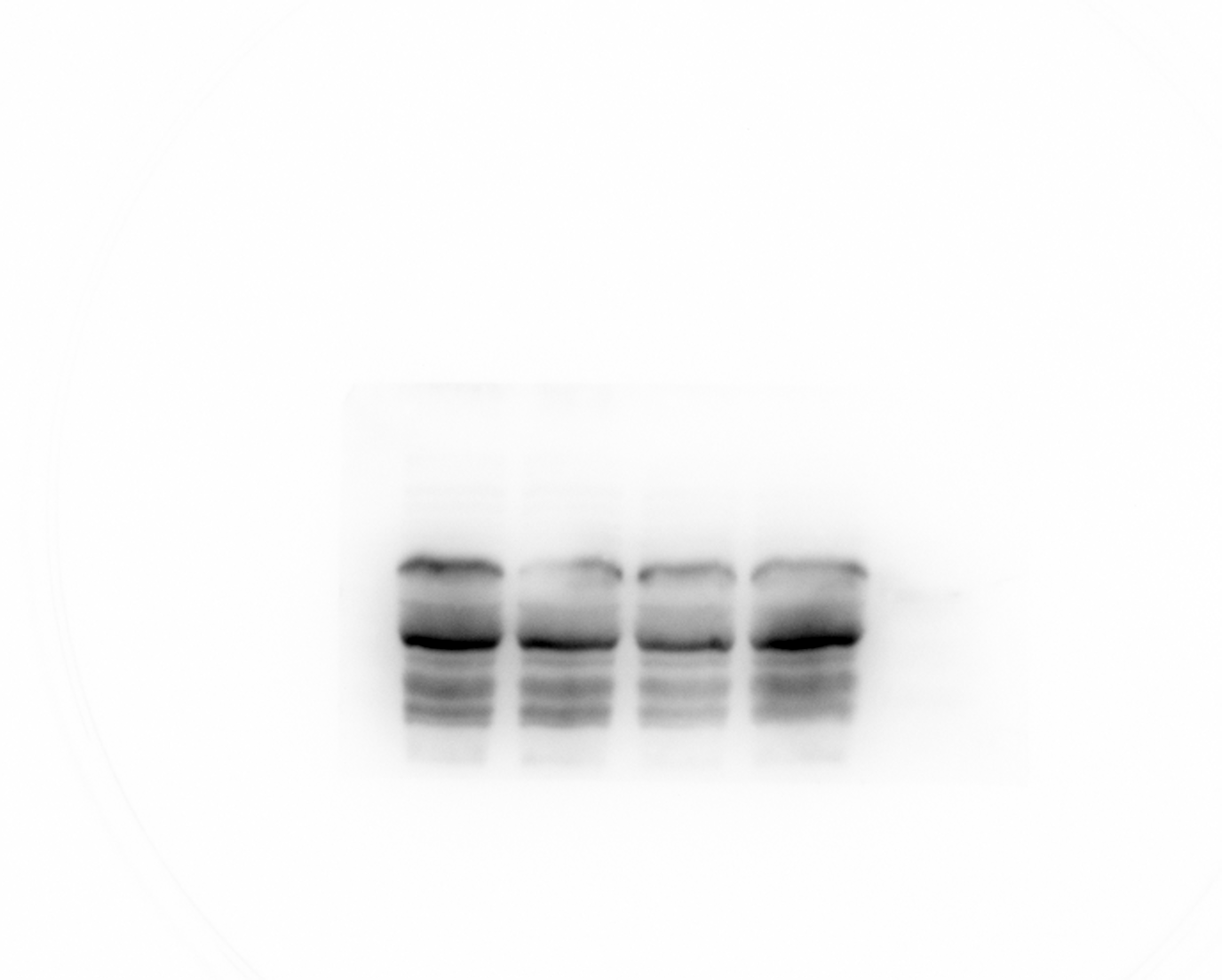

Supplement: Supplemental Information 10 [file peerj-13-19276-s010.zip › western blot-Total Cx43 EB1 N-cadherin 2/7-CX43.Tif]

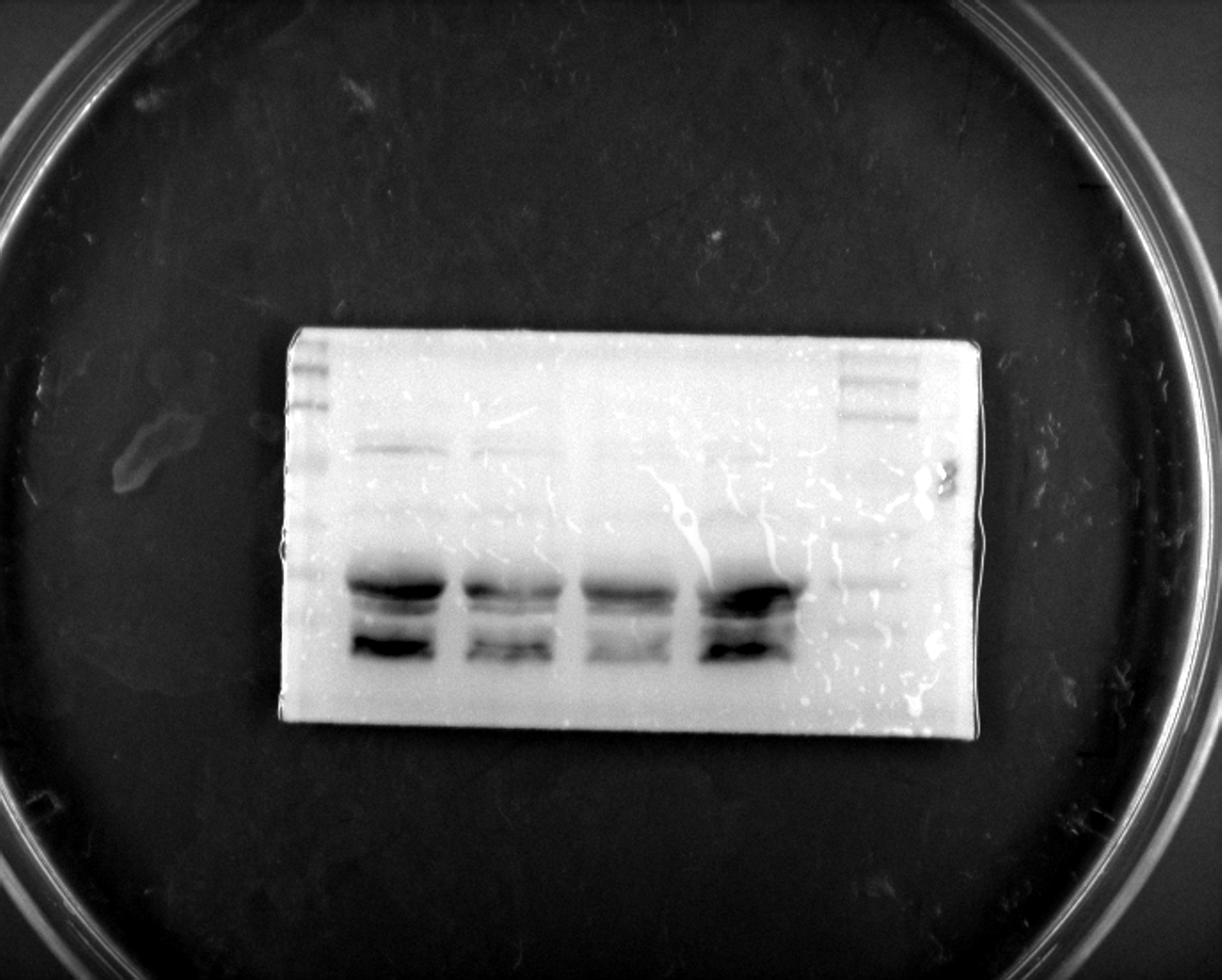

Supplement: Supplemental Information 10 [file peerj-13-19276-s010.zip › western blot-Total Cx43 EB1 N-cadherin 2/7-EB1-M.Tif]

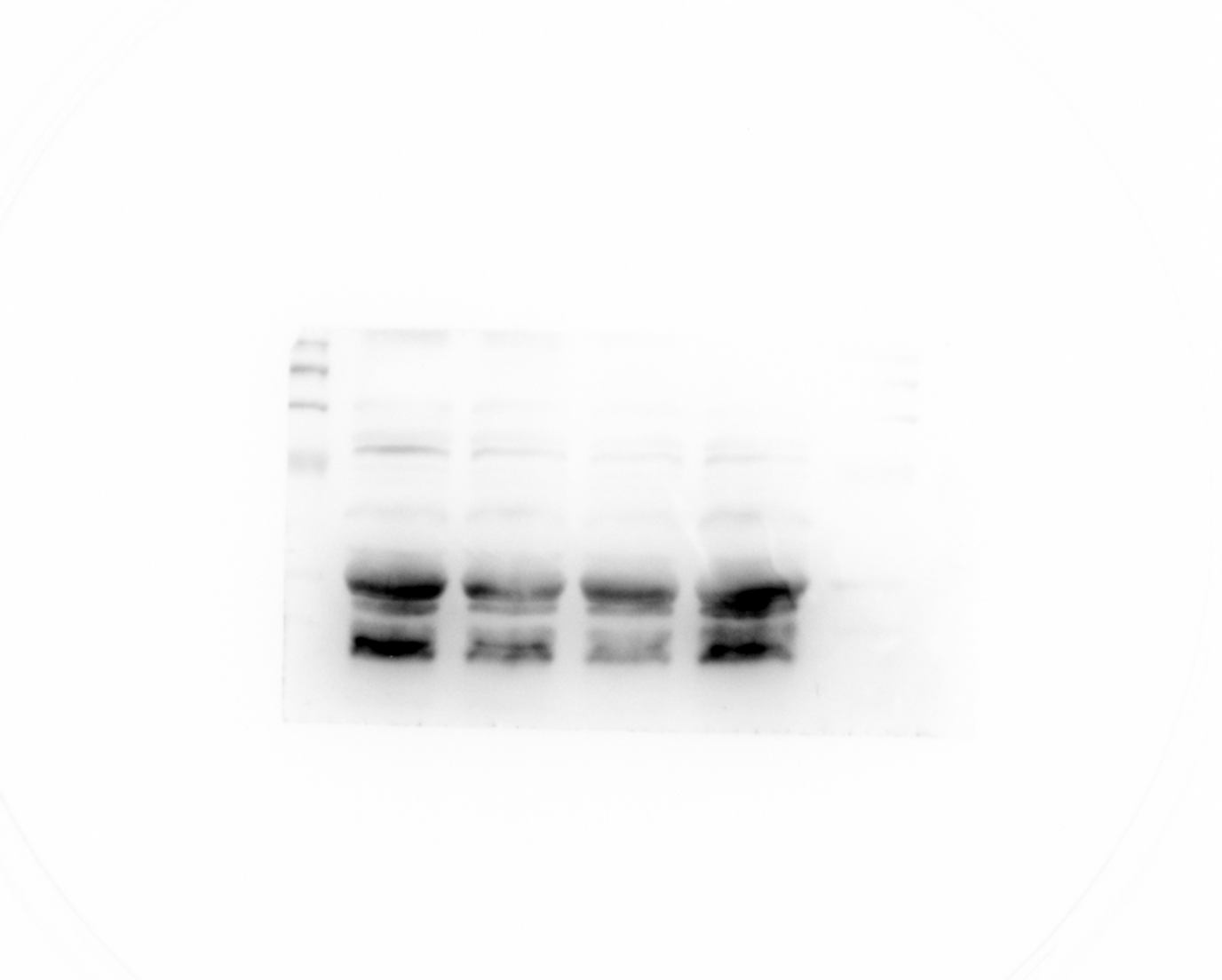

Supplement: Supplemental Information 10 [file peerj-13-19276-s010.zip › western blot-Total Cx43 EB1 N-cadherin 2/7-EB1.Tif]

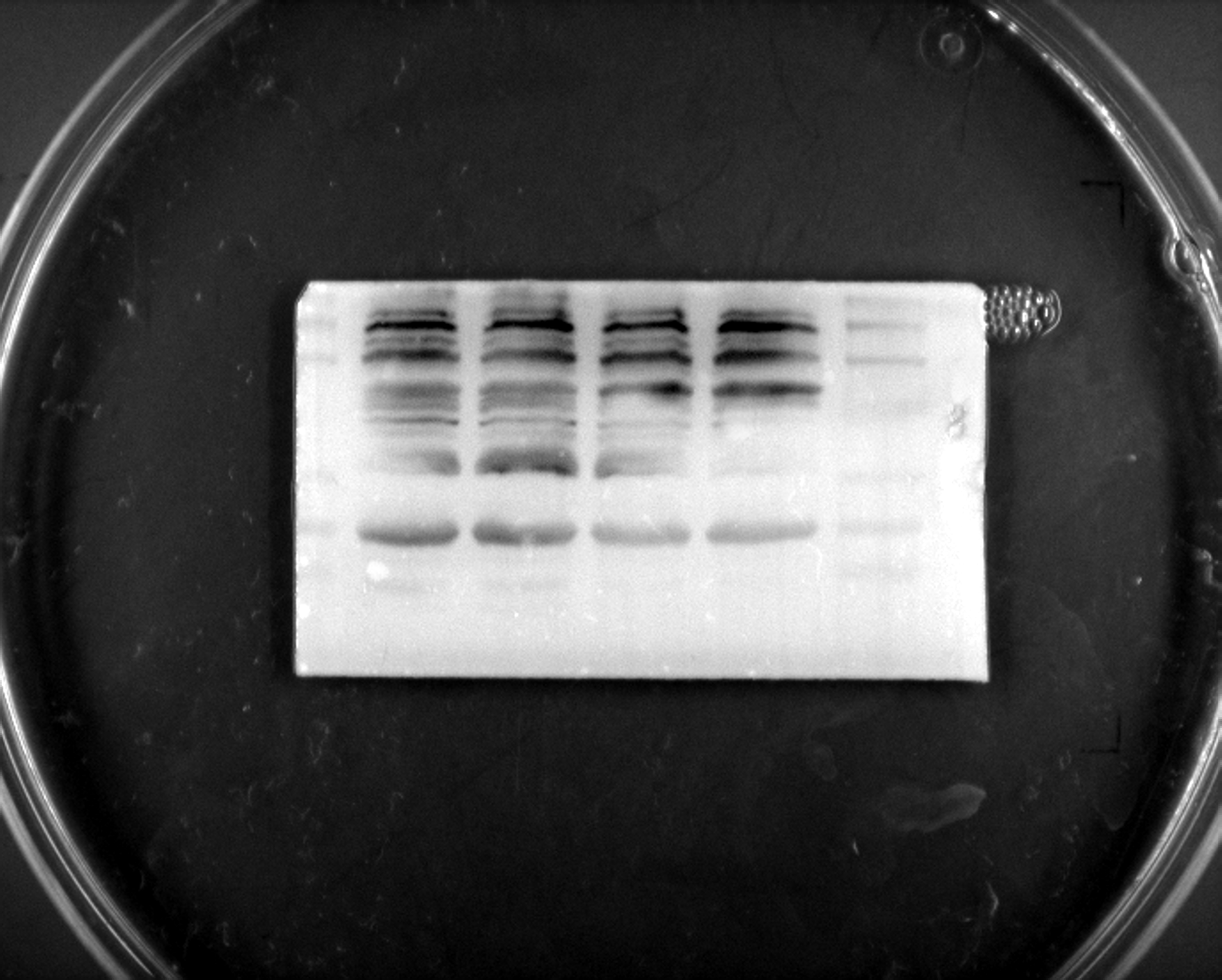

Supplement: Supplemental Information 10 [file peerj-13-19276-s010.zip › western blot-Total Cx43 EB1 N-cadherin 2/7-N-cadherin-M.Tif]

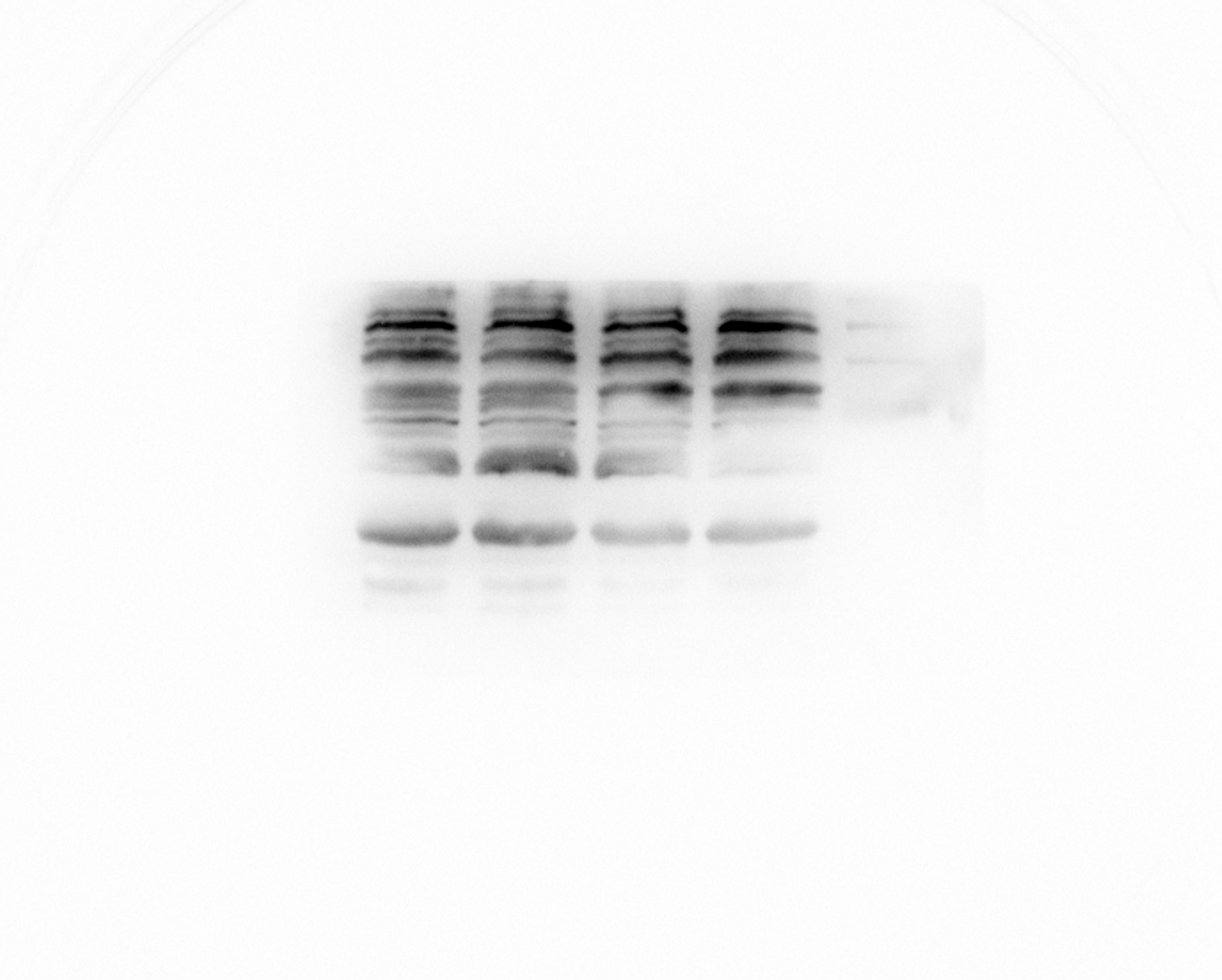

Supplement: Supplemental Information 10 [file peerj-13-19276-s010.zip › western blot-Total Cx43 EB1 N-cadherin 2/7-N-cadherin.Tif]

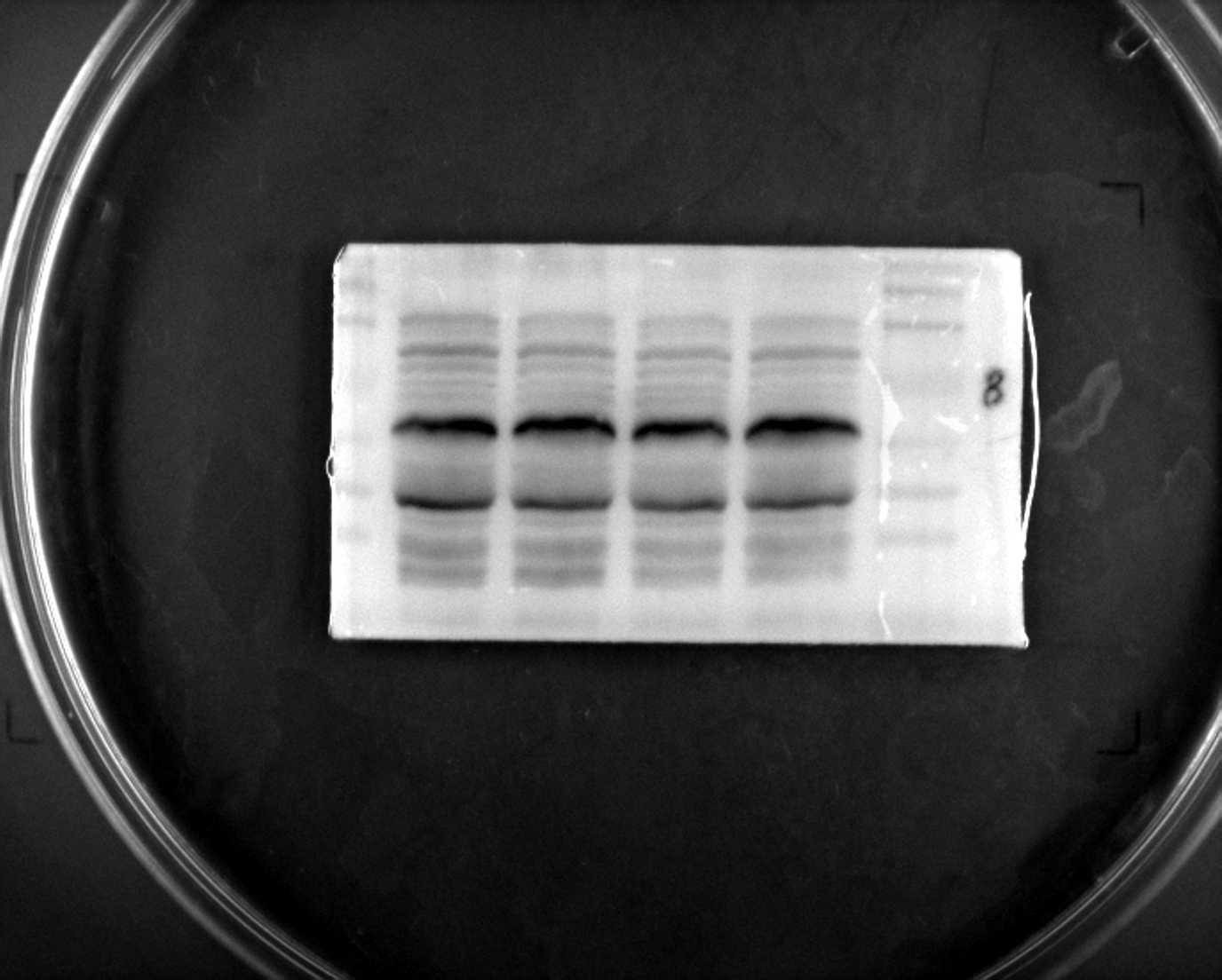

Supplement: Supplemental Information 10 [file peerj-13-19276-s010.zip › western blot-Total Cx43 EB1 N-cadherin 2/7-Tubulin-M.Tif]

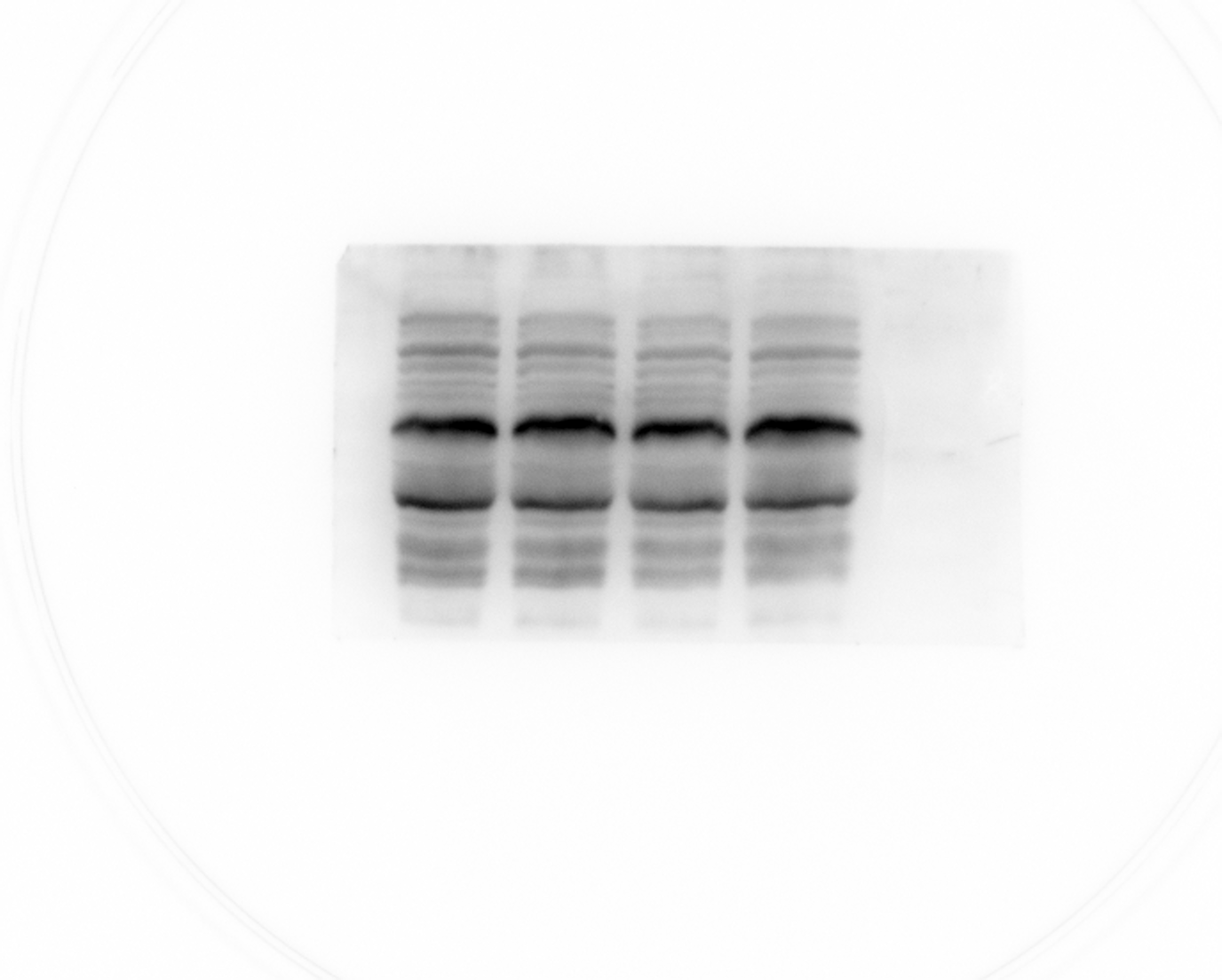

Supplement: Supplemental Information 10 [file peerj-13-19276-s010.zip › western blot-Total Cx43 EB1 N-cadherin 2/7-Tubulin.Tif]

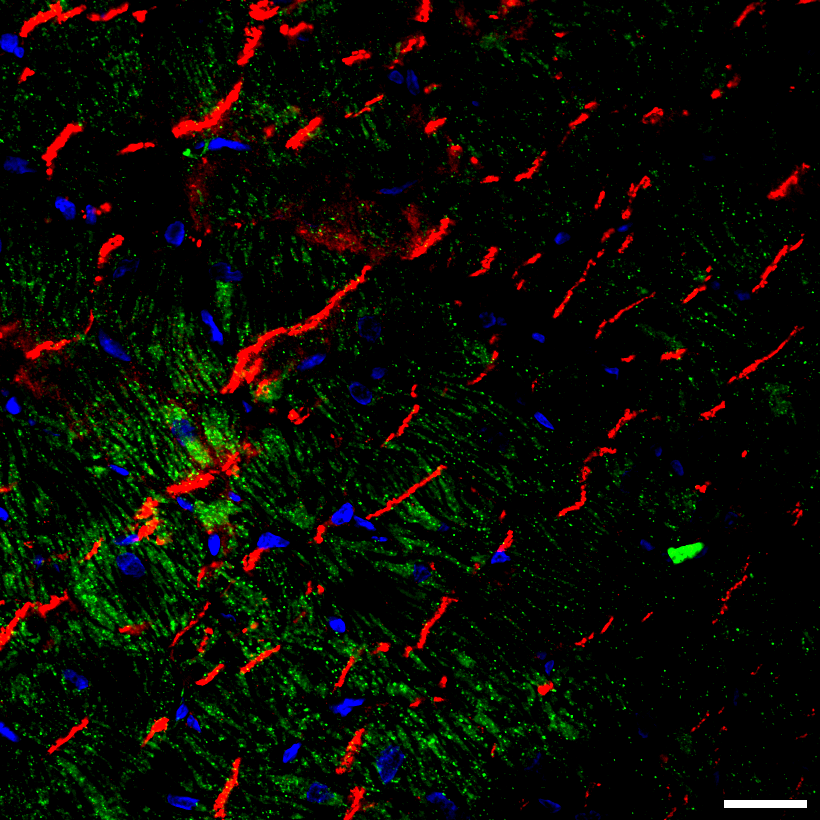

Supplement: Supplemental Information 11 [file peerj-13-19276-s011.zip › immunofluorescence EB1 - N-cadherin-C/C1-1.tif]

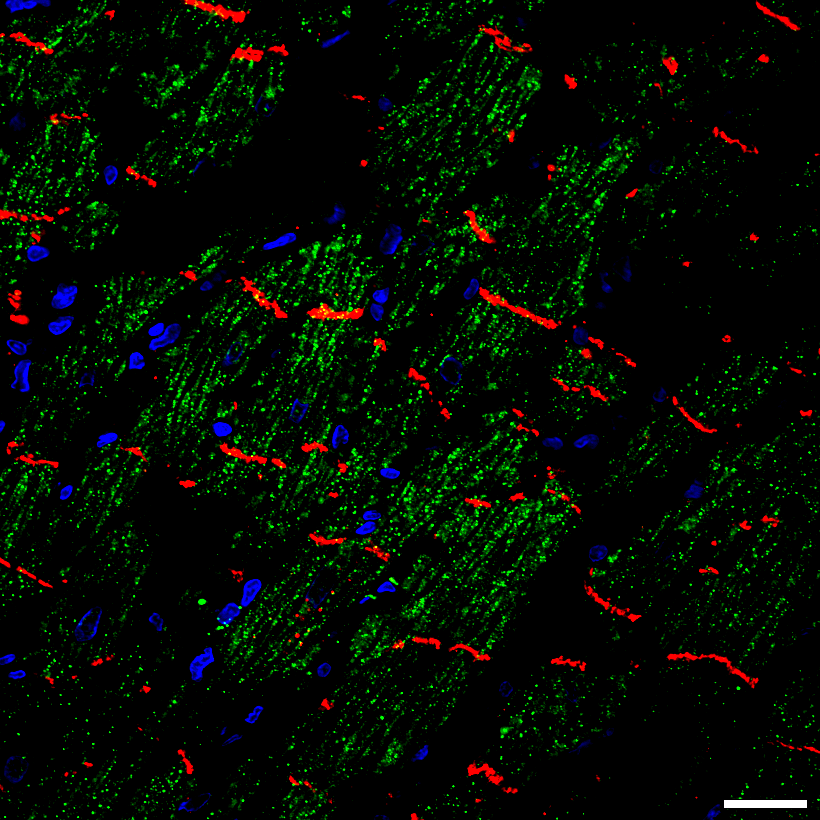

Supplement: Supplemental Information 11 [file peerj-13-19276-s011.zip › immunofluorescence EB1 - N-cadherin-C/C1-2.tif]

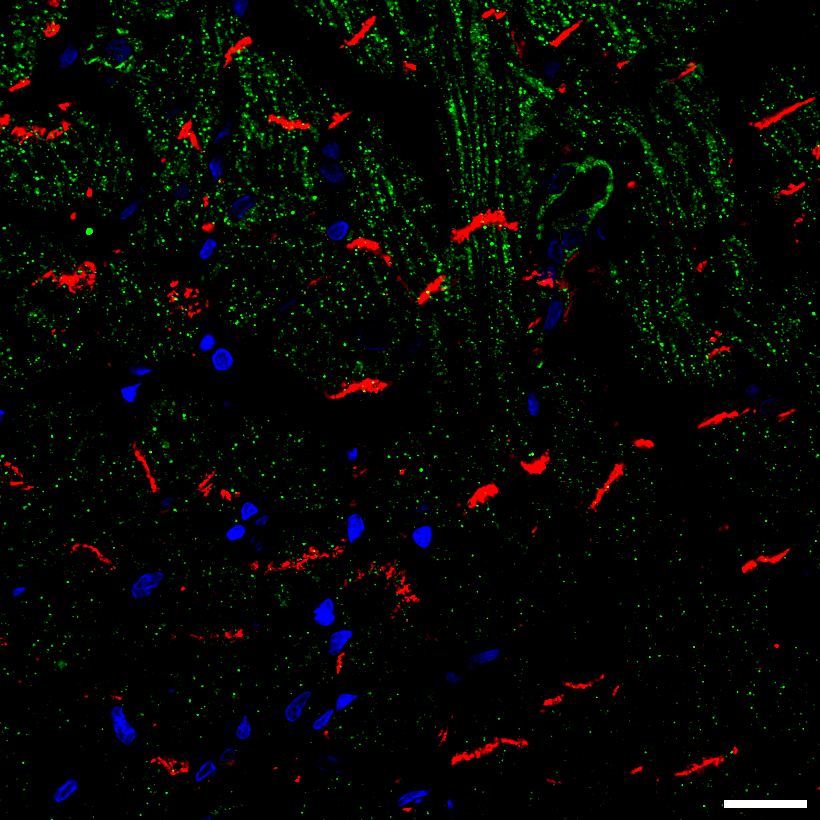

Supplement: Supplemental Information 11 [file peerj-13-19276-s011.zip › immunofluorescence EB1 - N-cadherin-C/C2-1.tif]

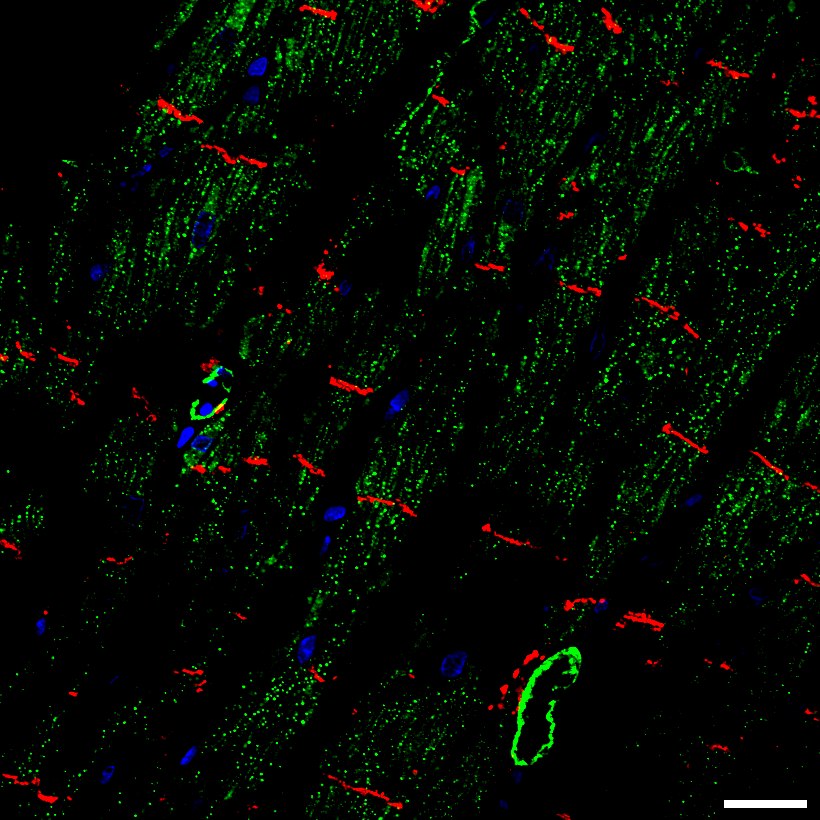

Supplement: Supplemental Information 11 [file peerj-13-19276-s011.zip › immunofluorescence EB1 - N-cadherin-C/C2-2.tif]

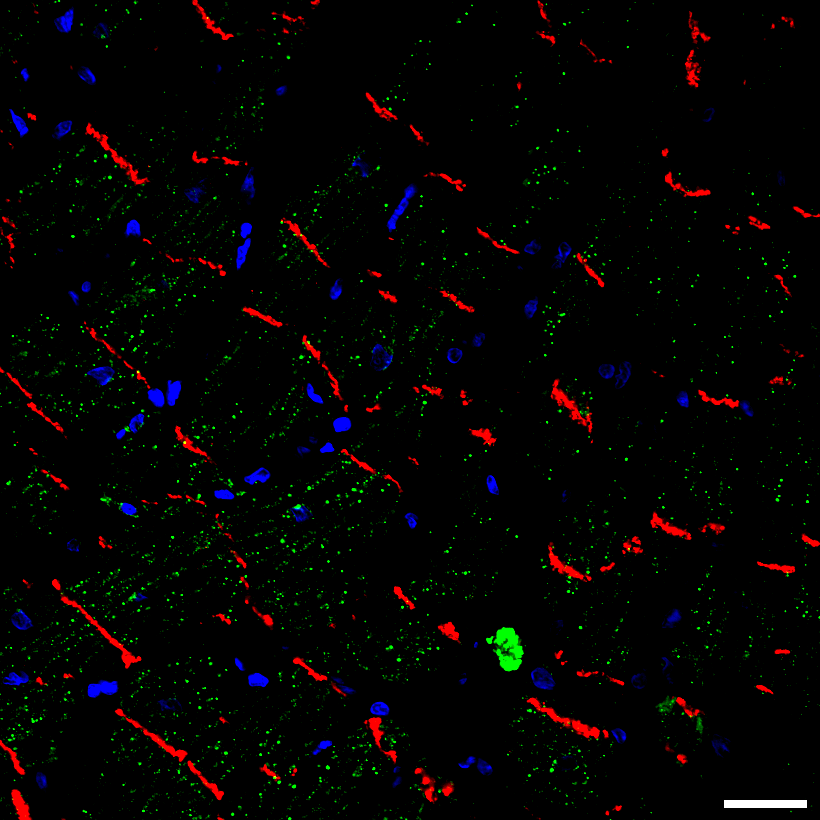

Supplement: Supplemental Information 11 [file peerj-13-19276-s011.zip › immunofluorescence EB1 - N-cadherin-C/C3-1.tif]

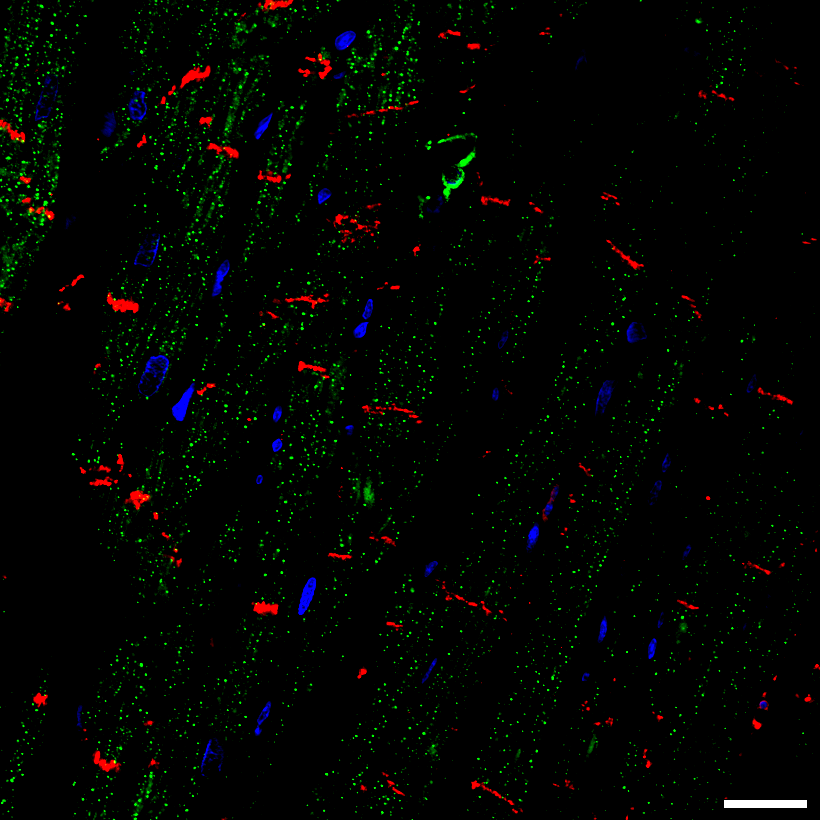

Supplement: Supplemental Information 11 [file peerj-13-19276-s011.zip › immunofluorescence EB1 - N-cadherin-C/C3-2.tif]

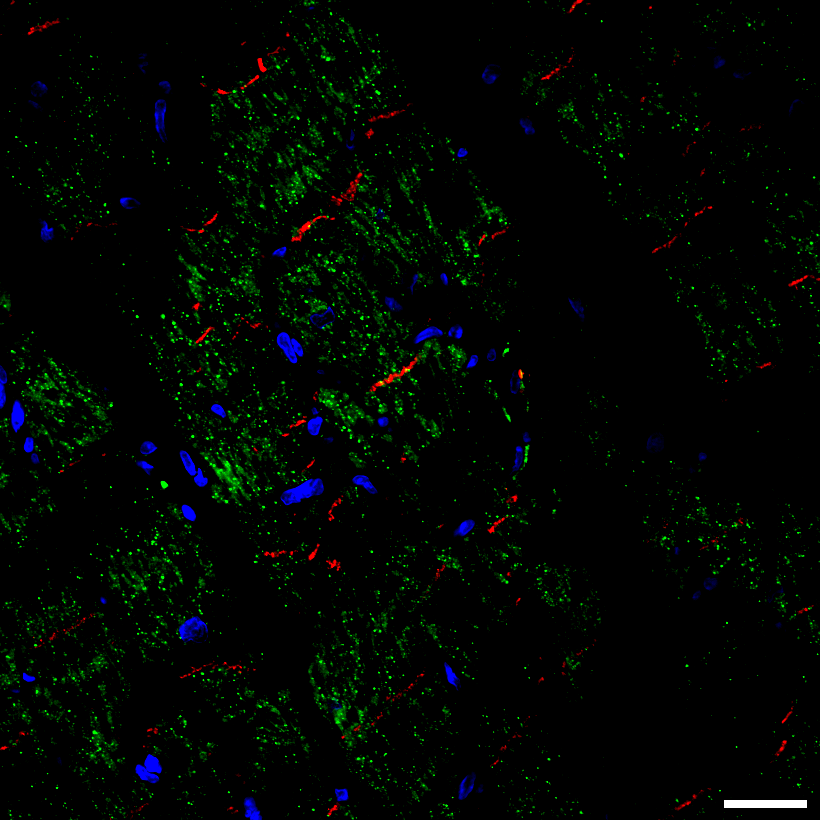

Supplement: Supplemental Information 11 [file peerj-13-19276-s011.zip › immunofluorescence EB1 - N-cadherin-C/C4-1.tif]

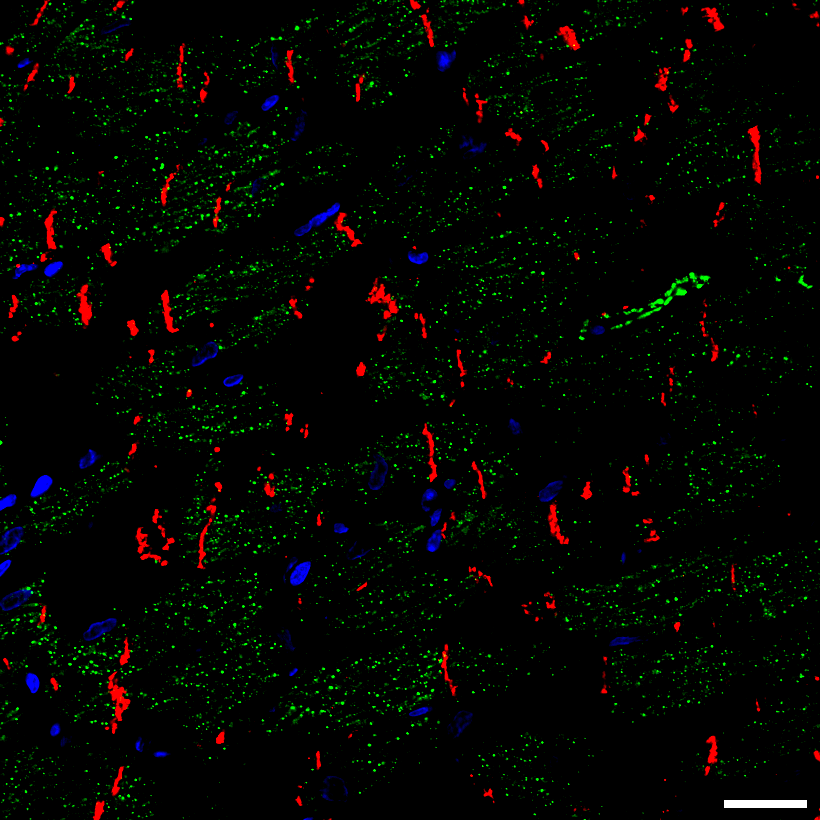

Supplement: Supplemental Information 11 [file peerj-13-19276-s011.zip › immunofluorescence EB1 - N-cadherin-C/C4-2.tif]

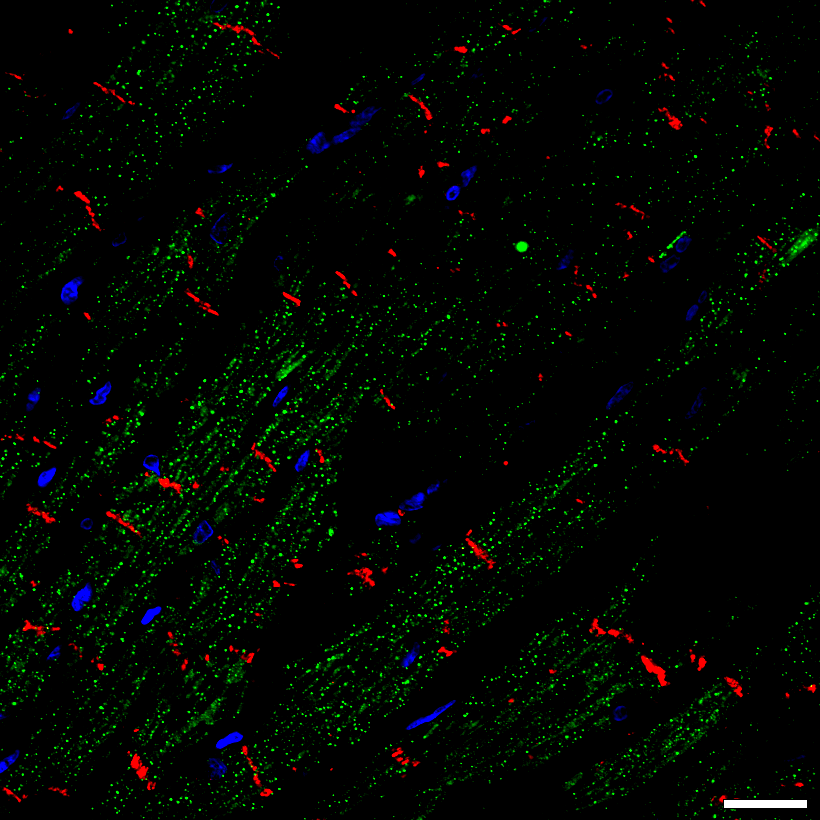

Supplement: Supplemental Information 11 [file peerj-13-19276-s011.zip › immunofluorescence EB1 - N-cadherin-C/C5-1.tif]

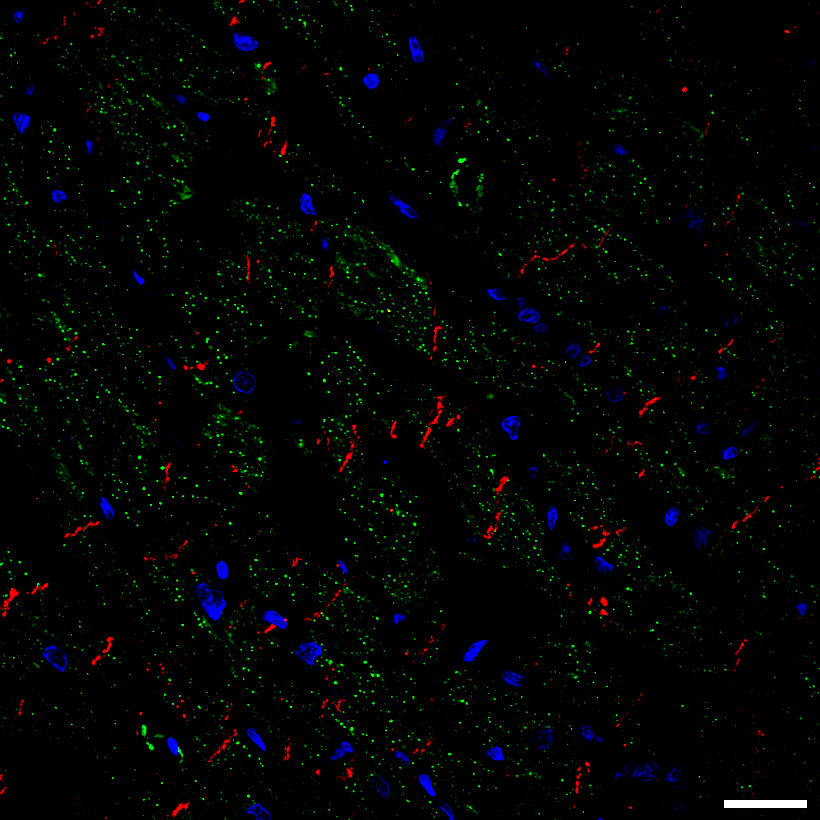

Supplement: Supplemental Information 11 [file peerj-13-19276-s011.zip › immunofluorescence EB1 - N-cadherin-C/C5-2.tif]

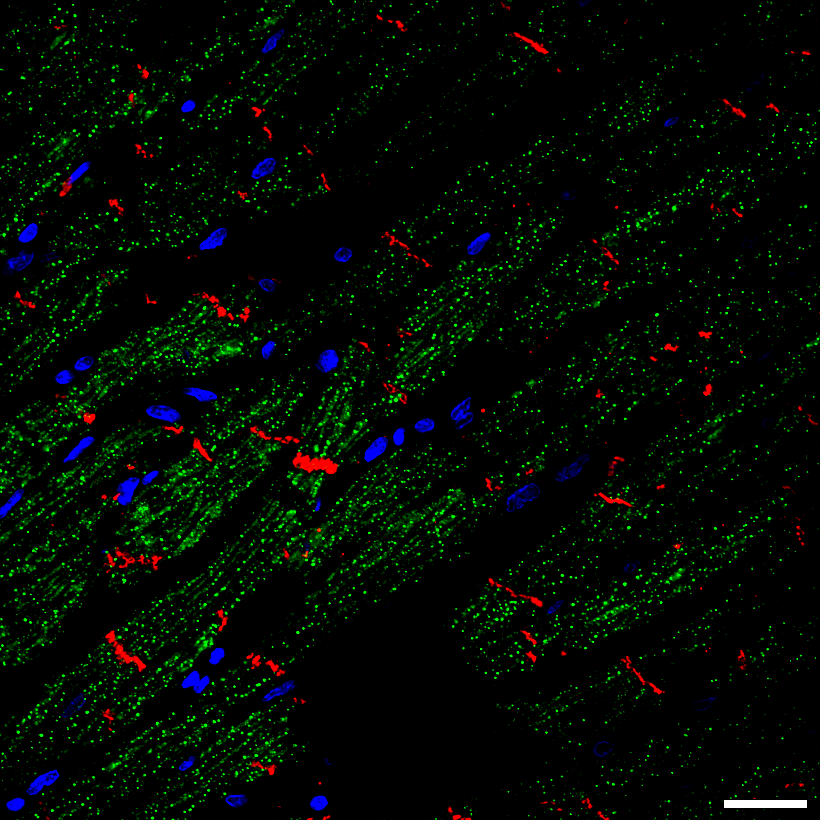

Supplement: Supplemental Information 11 [file peerj-13-19276-s011.zip › immunofluorescence EB1 - N-cadherin-C/C6-1.tif]

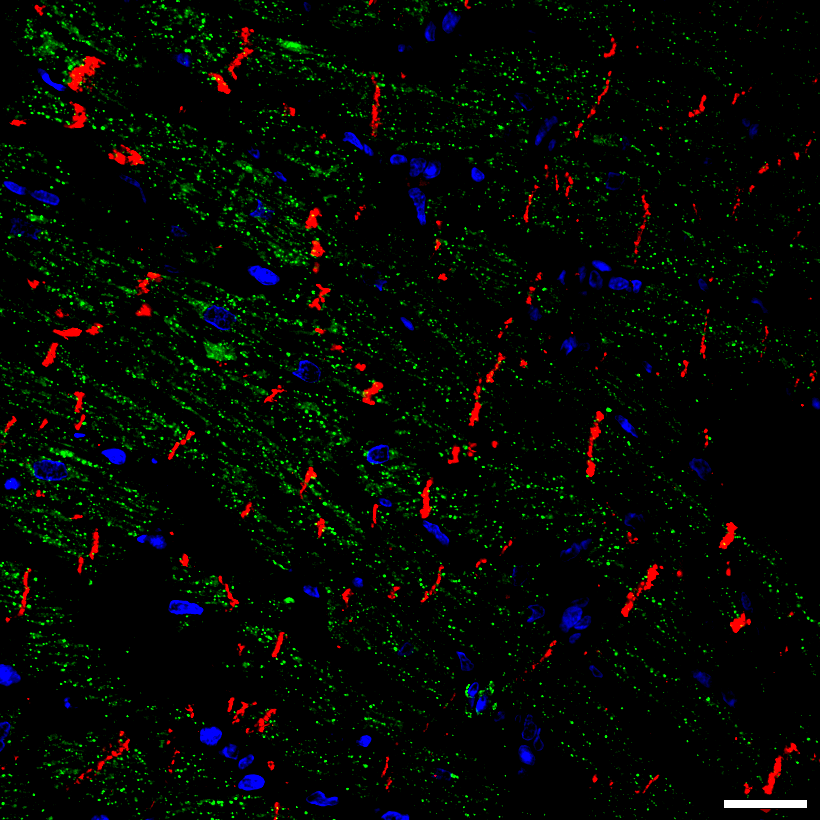

Supplement: Supplemental Information 11 [file peerj-13-19276-s011.zip › immunofluorescence EB1 - N-cadherin-C/C6-2.tif]

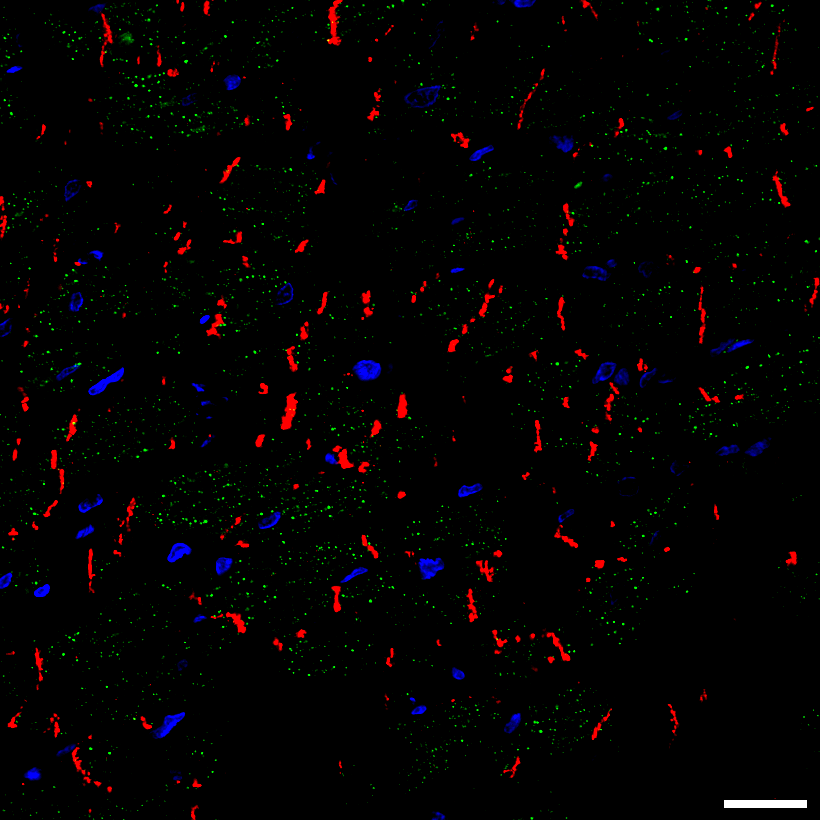

Supplement: Supplemental Information 12 [file peerj-13-19276-s012.zip › immunofluorescence EB1 - N-cadherin-I/R/I/R-7_01.tif]

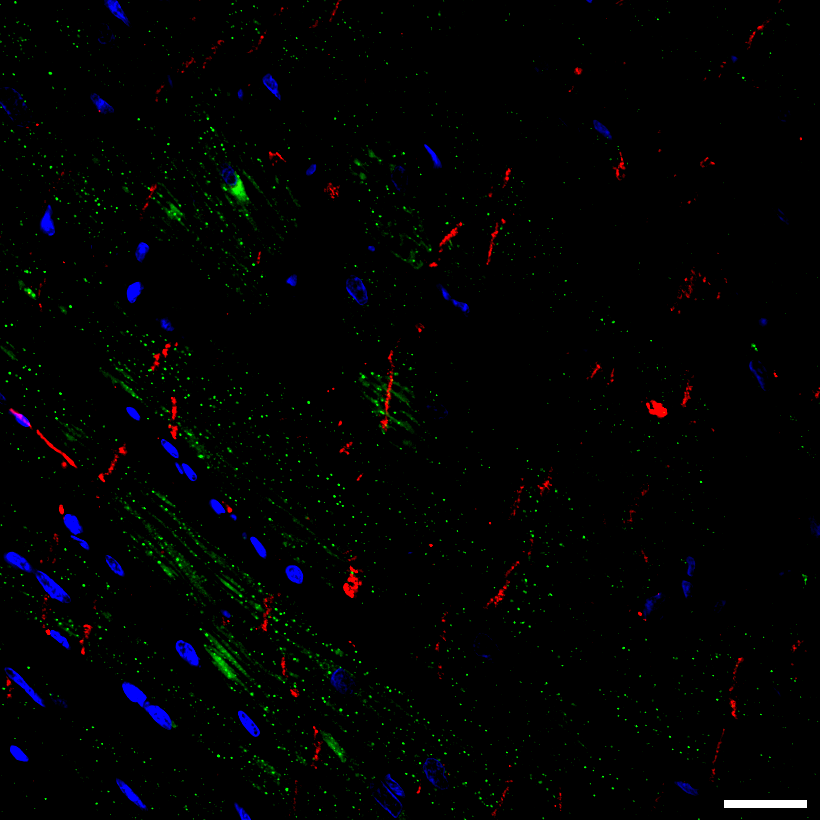

Supplement: Supplemental Information 12 [file peerj-13-19276-s012.zip › immunofluorescence EB1 - N-cadherin-I/R/I/R-7_02.tif]

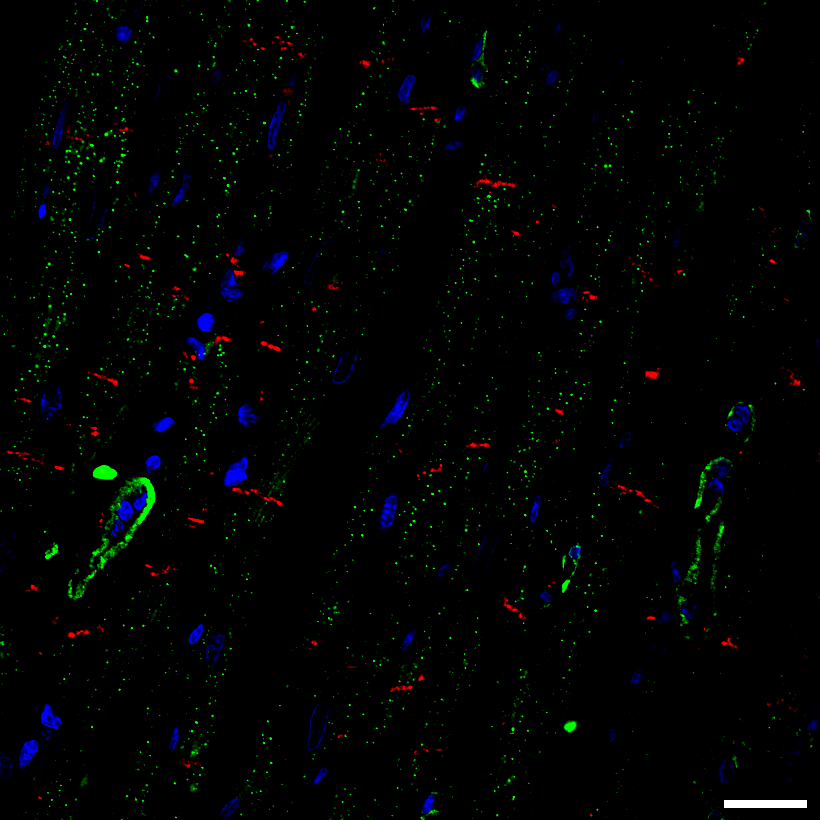

Supplement: Supplemental Information 12 [file peerj-13-19276-s012.zip › immunofluorescence EB1 - N-cadherin-I/R/I/R-8_1.tif]

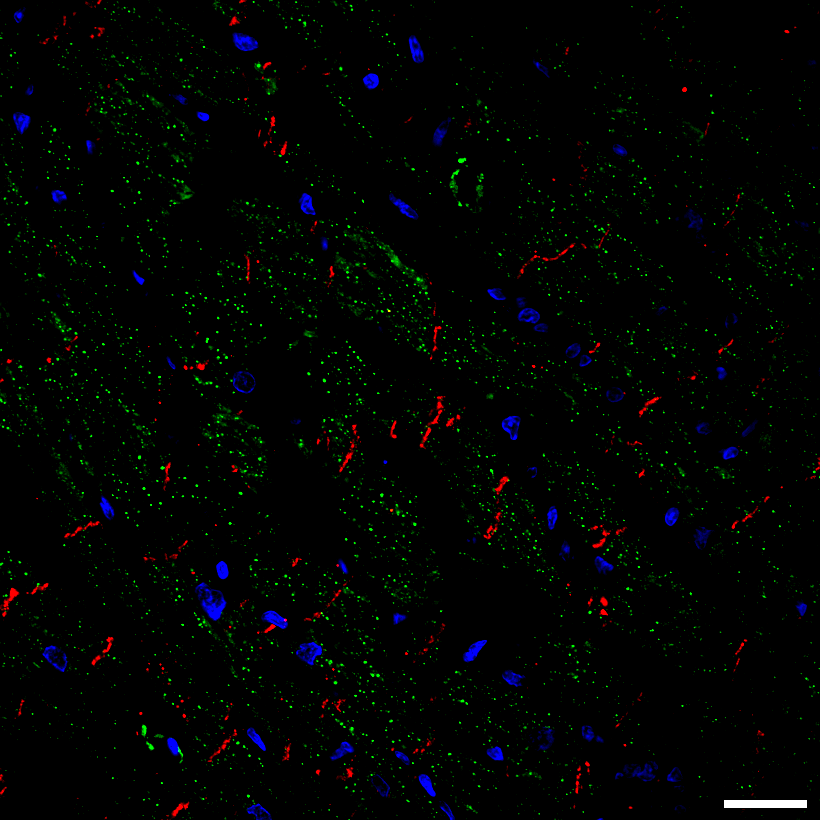

Supplement: Supplemental Information 12 [file peerj-13-19276-s012.zip › immunofluorescence EB1 - N-cadherin-I/R/I/R-8_2.tif]

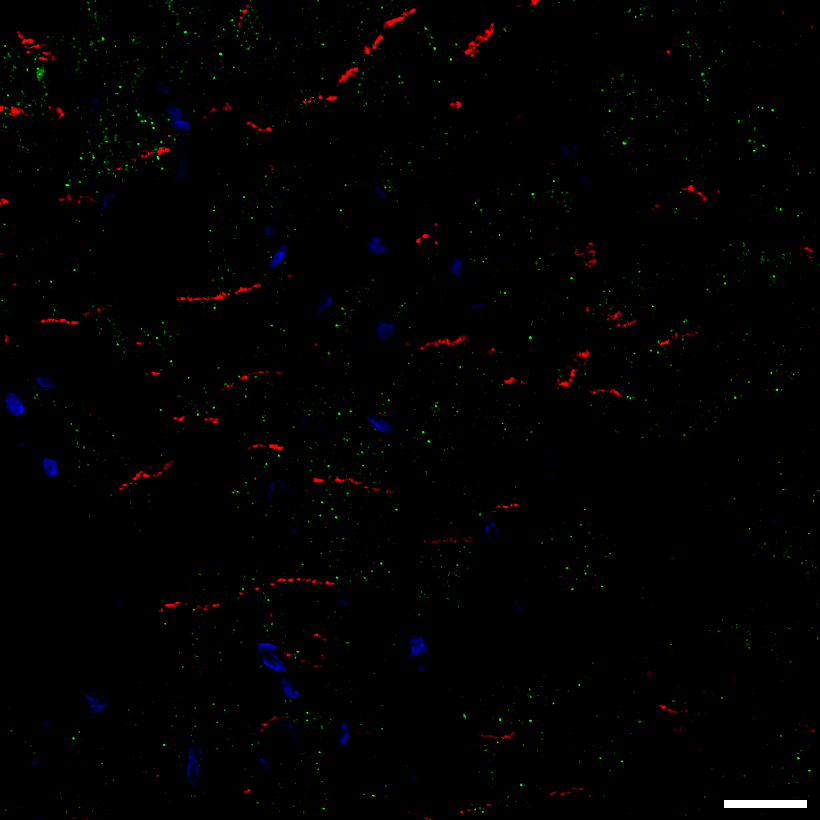

Supplement: Supplemental Information 12 [file peerj-13-19276-s012.zip › immunofluorescence EB1 - N-cadherin-I/R/I/R1-1.tif]

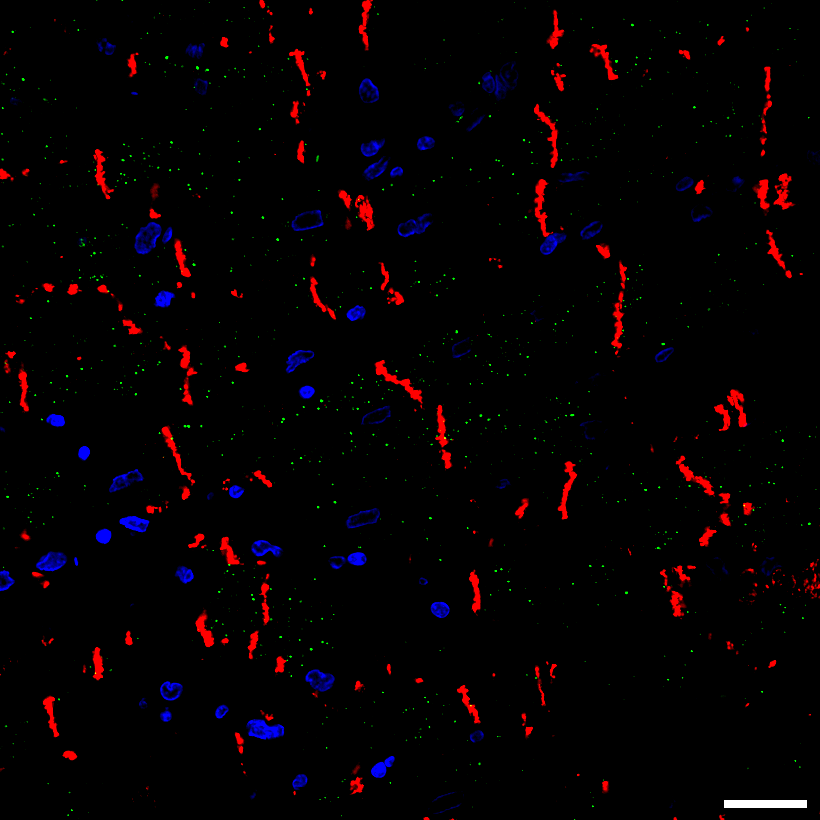

Supplement: Supplemental Information 12 [file peerj-13-19276-s012.zip › immunofluorescence EB1 - N-cadherin-I/R/I/R1-2.tif]

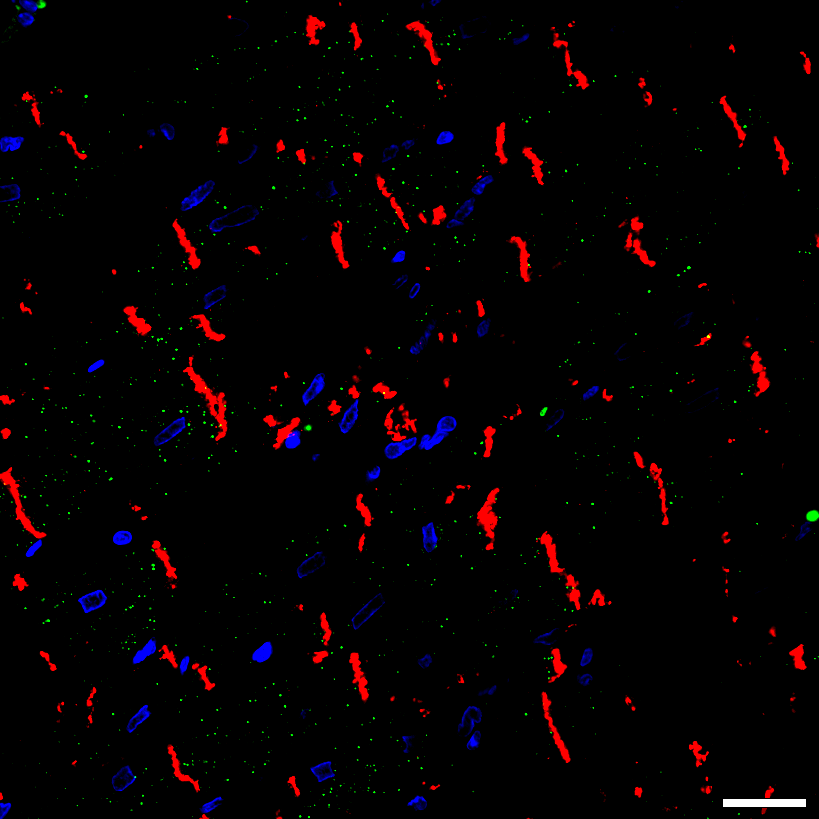

Supplement: Supplemental Information 12 [file peerj-13-19276-s012.zip › immunofluorescence EB1 - N-cadherin-I/R/I/R2-1.tif]

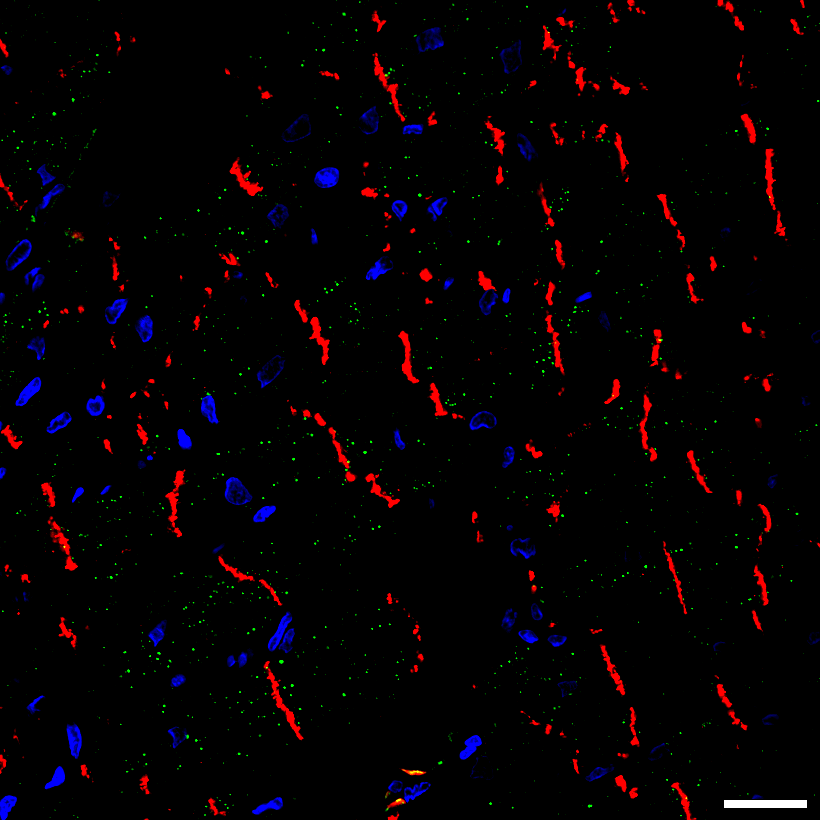

Supplement: Supplemental Information 12 [file peerj-13-19276-s012.zip › immunofluorescence EB1 - N-cadherin-I/R/I/R2-2.tif]

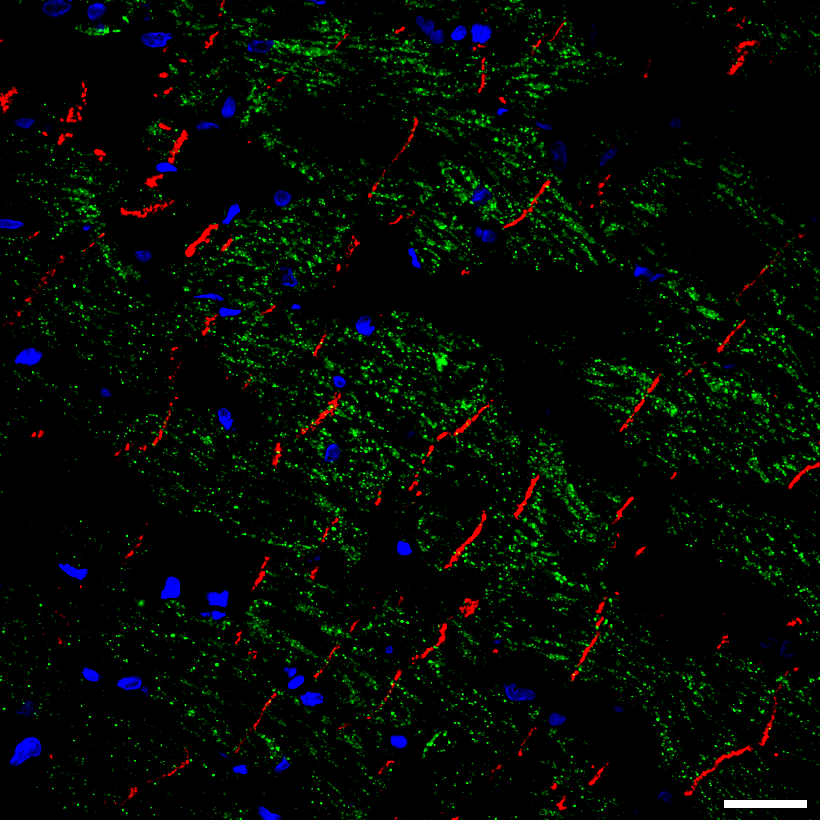

Supplement: Supplemental Information 12 [file peerj-13-19276-s012.zip › immunofluorescence EB1 - N-cadherin-I/R/I/R2_04.tif]

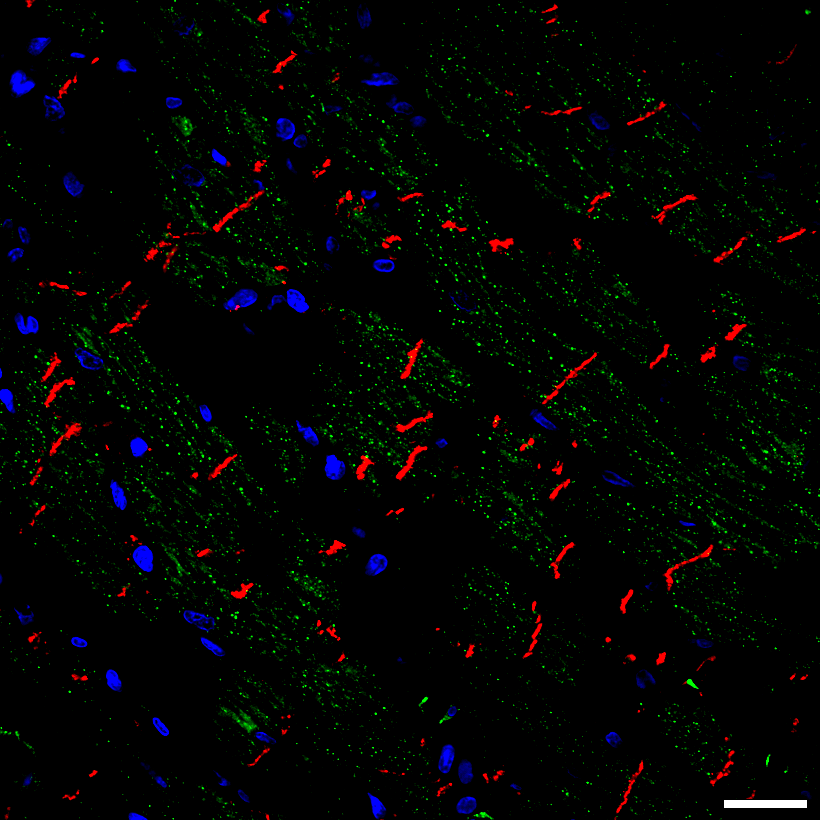

Supplement: Supplemental Information 12 [file peerj-13-19276-s012.zip › immunofluorescence EB1 - N-cadherin-I/R/I/R2_06.tif]

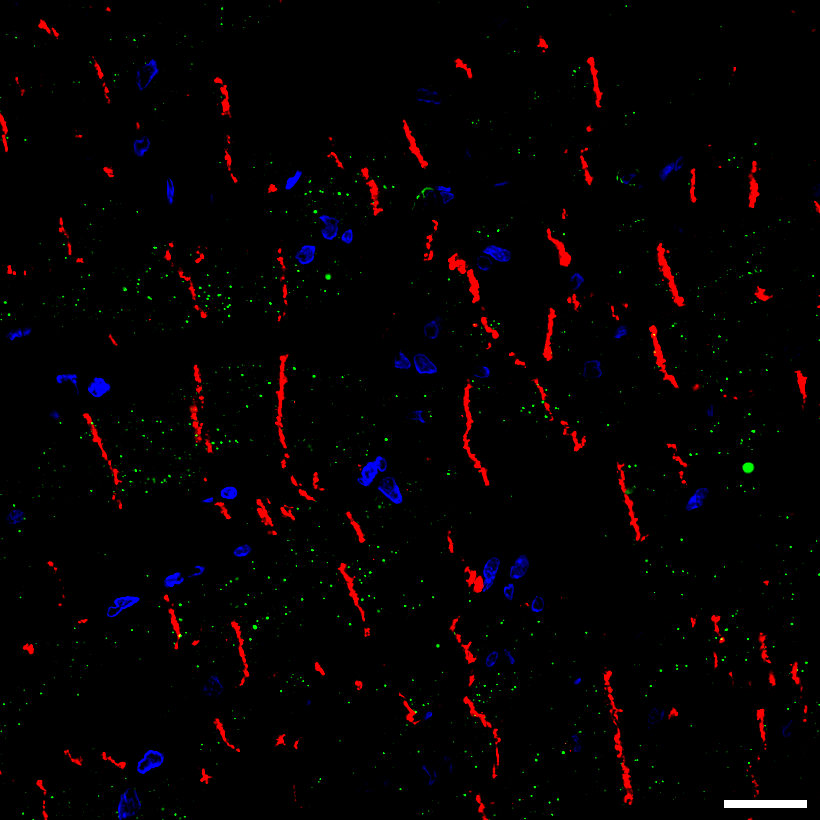

Supplement: Supplemental Information 12 [file peerj-13-19276-s012.zip › immunofluorescence EB1 - N-cadherin-I/R/I/R3-1.tif]

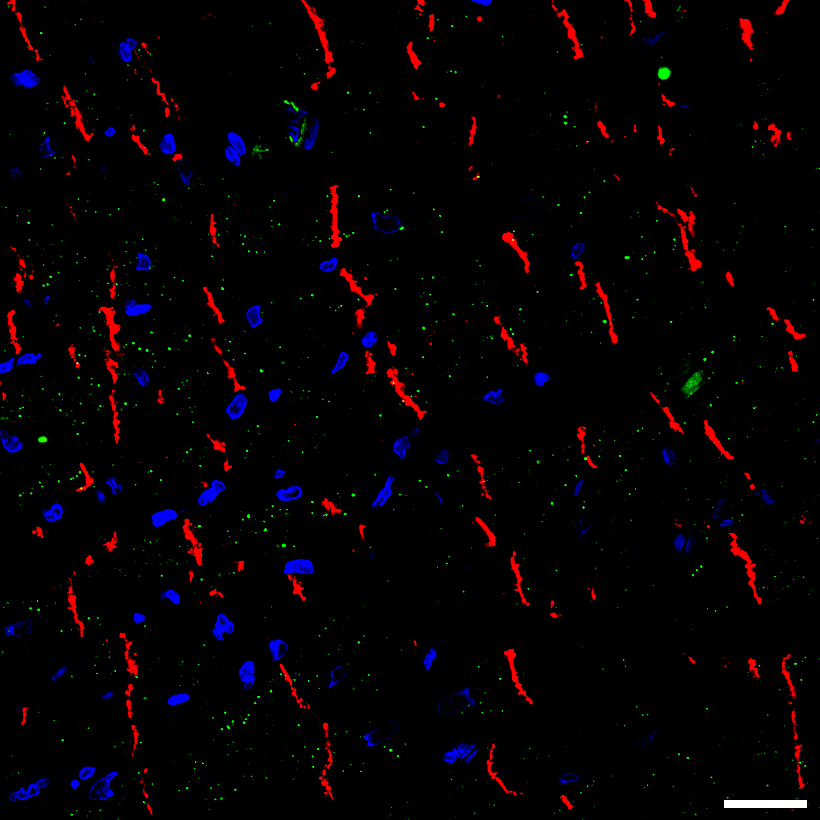

Supplement: Supplemental Information 12 [file peerj-13-19276-s012.zip › immunofluorescence EB1 - N-cadherin-I/R/I/R3-2.tif]

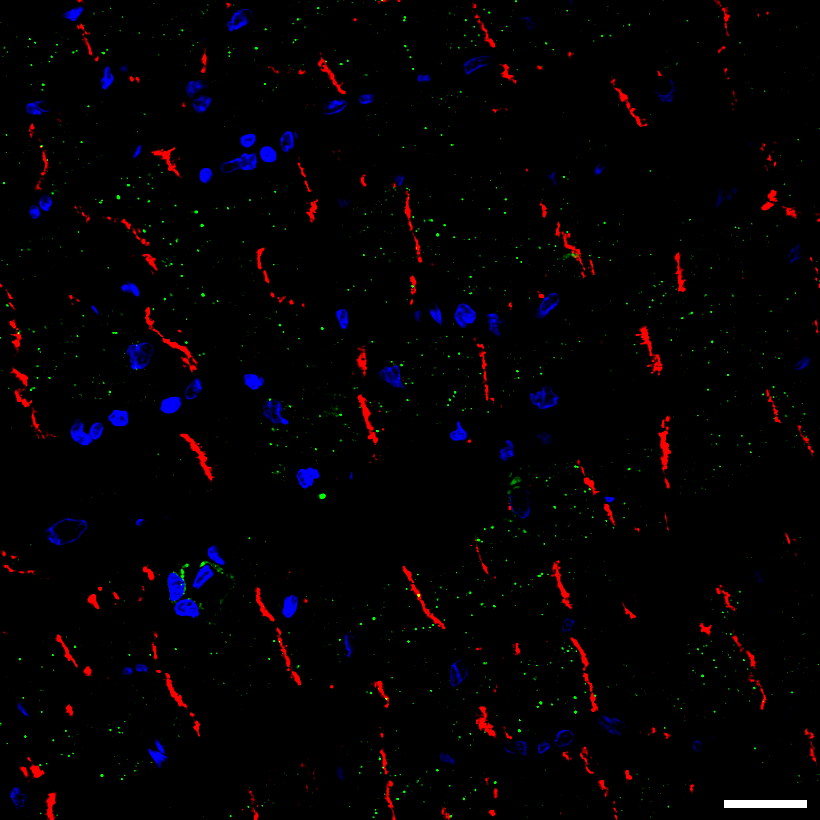

Supplement: Supplemental Information 12 [file peerj-13-19276-s012.zip › immunofluorescence EB1 - N-cadherin-I/R/I/R4-1.tif]

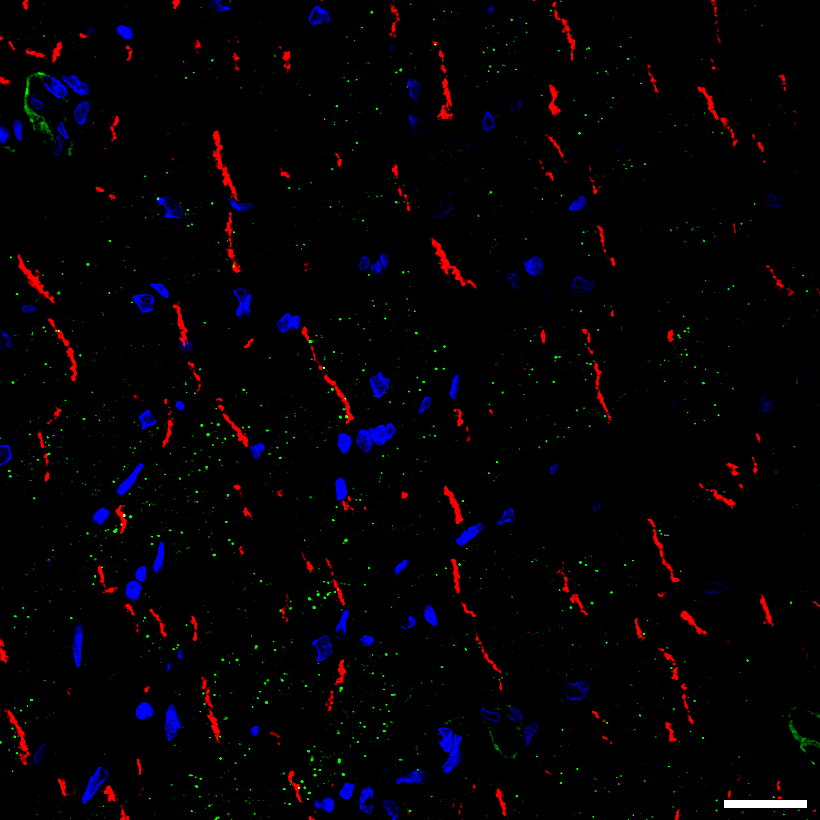

Supplement: Supplemental Information 12 [file peerj-13-19276-s012.zip › immunofluorescence EB1 - N-cadherin-I/R/I/R4-2.tif]

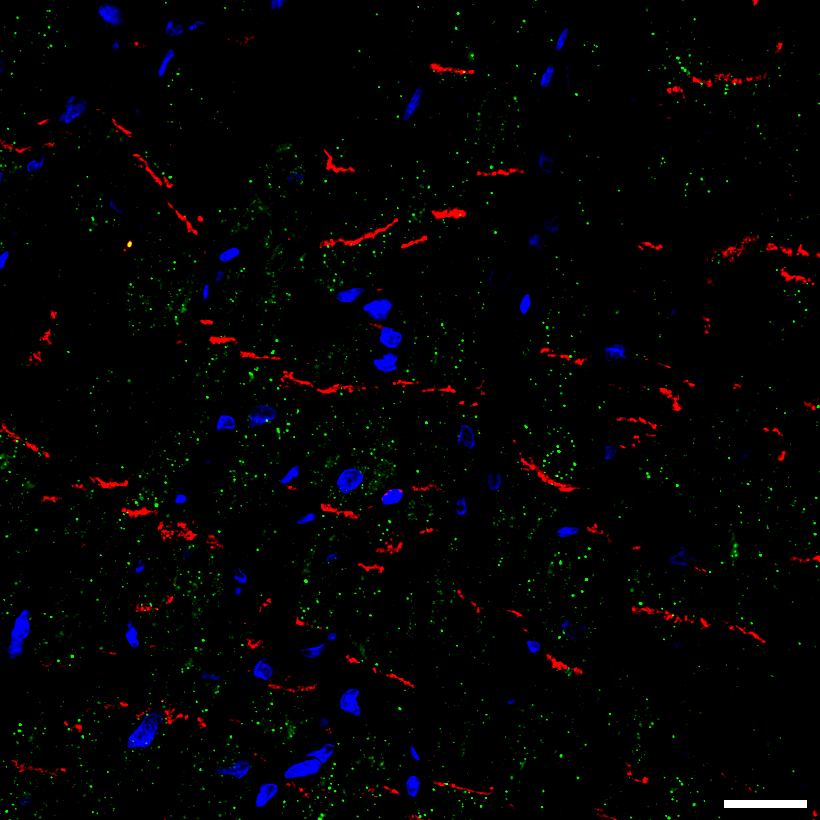

Supplement: Supplemental Information 12 [file peerj-13-19276-s012.zip › immunofluorescence EB1 - N-cadherin-I/R/I/R5-1.tif]

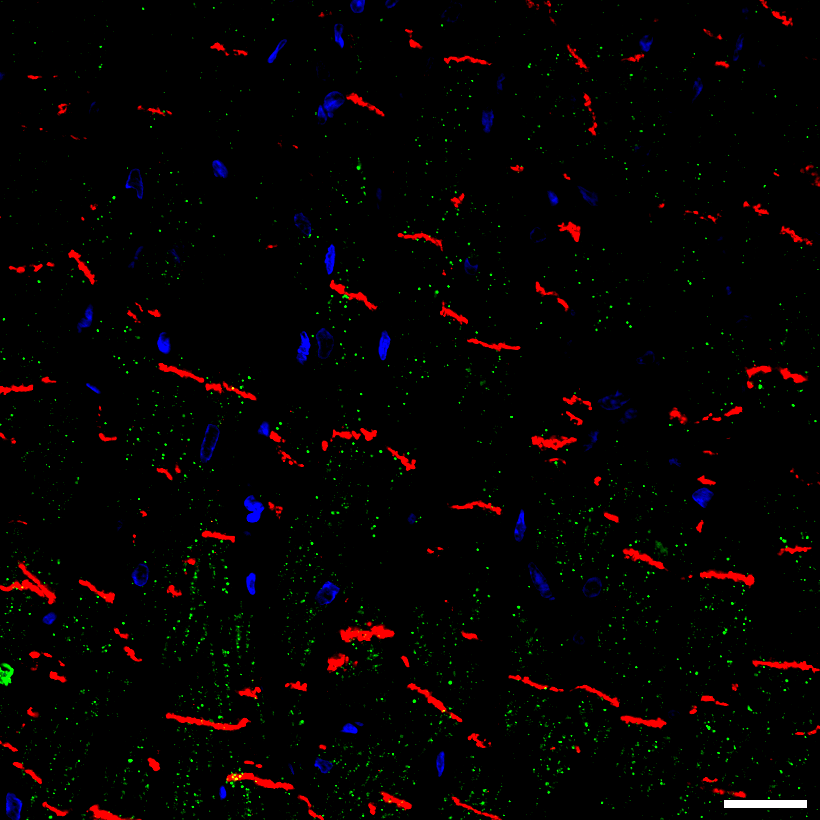

Supplement: Supplemental Information 12 [file peerj-13-19276-s012.zip › immunofluorescence EB1 - N-cadherin-I/R/I/R5-2.tif]

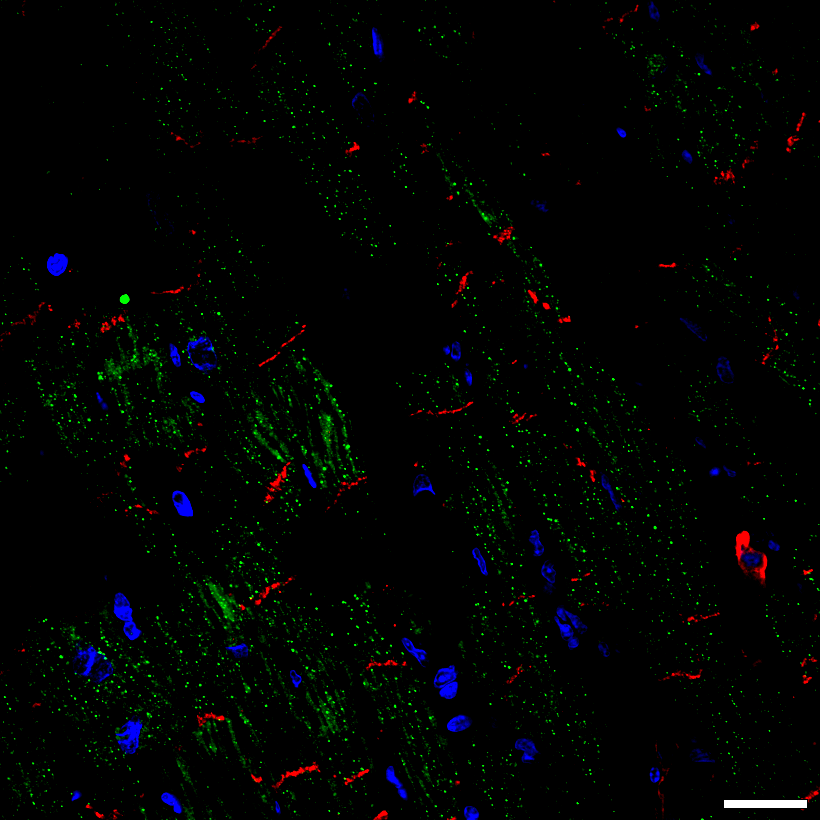

Supplement: Supplemental Information 12 [file peerj-13-19276-s012.zip › immunofluorescence EB1 - N-cadherin-I/R/I/R6-1.tif]

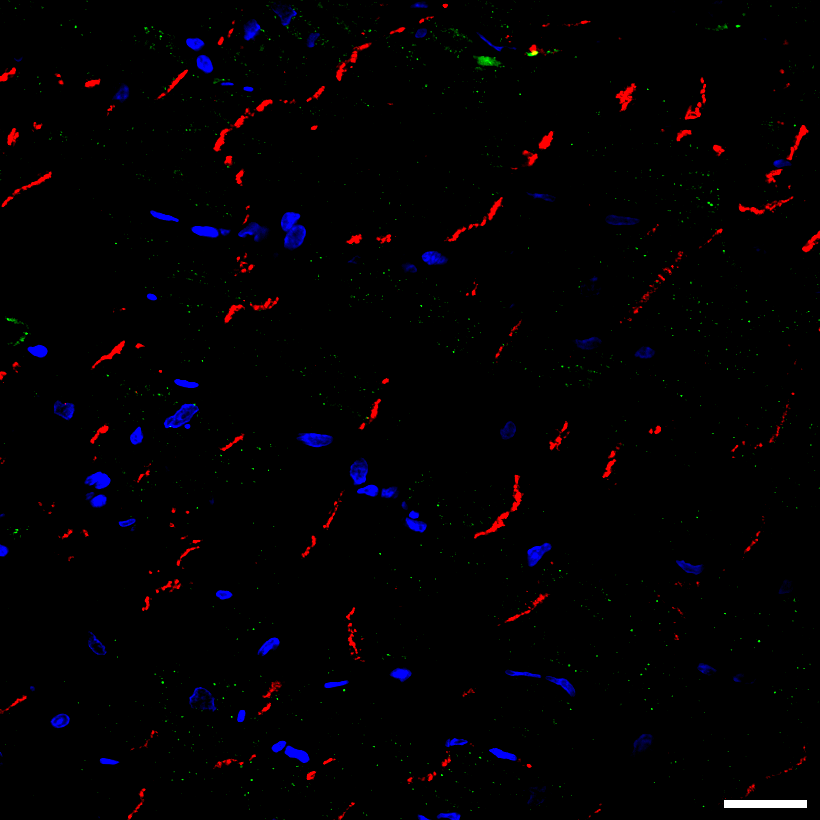

Supplement: Supplemental Information 12 [file peerj-13-19276-s012.zip › immunofluorescence EB1 - N-cadherin-I/R/I/R6-2.tif]

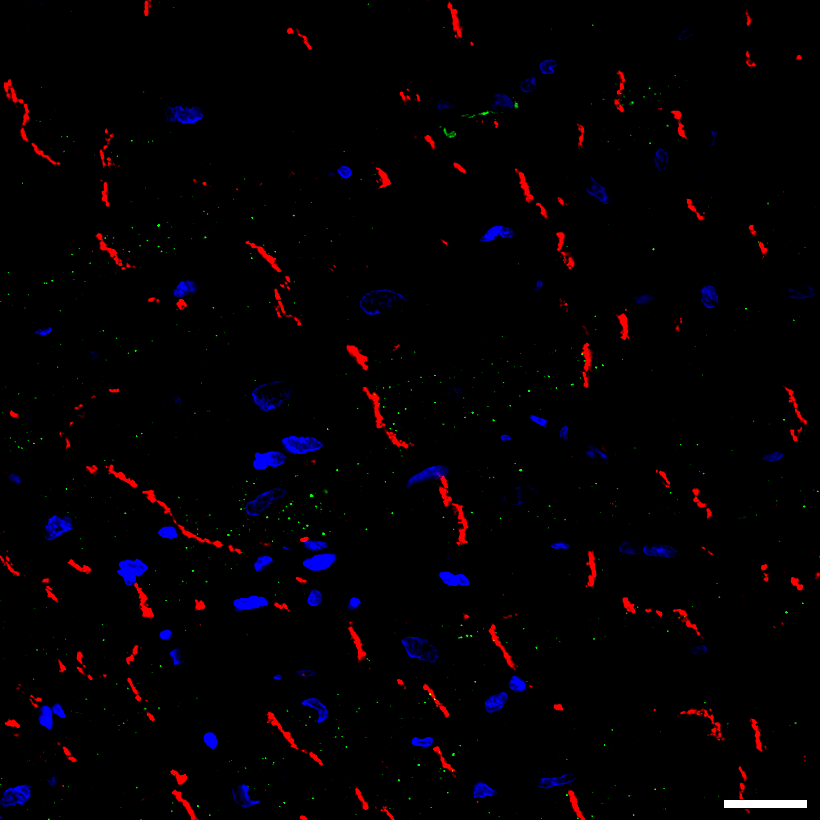

Supplement: Supplemental Information 13 [file peerj-13-19276-s013.zip › immunofluorescence EB1 - N-cadherin-AAV9-CON/AAV9-CON1-1.tif]

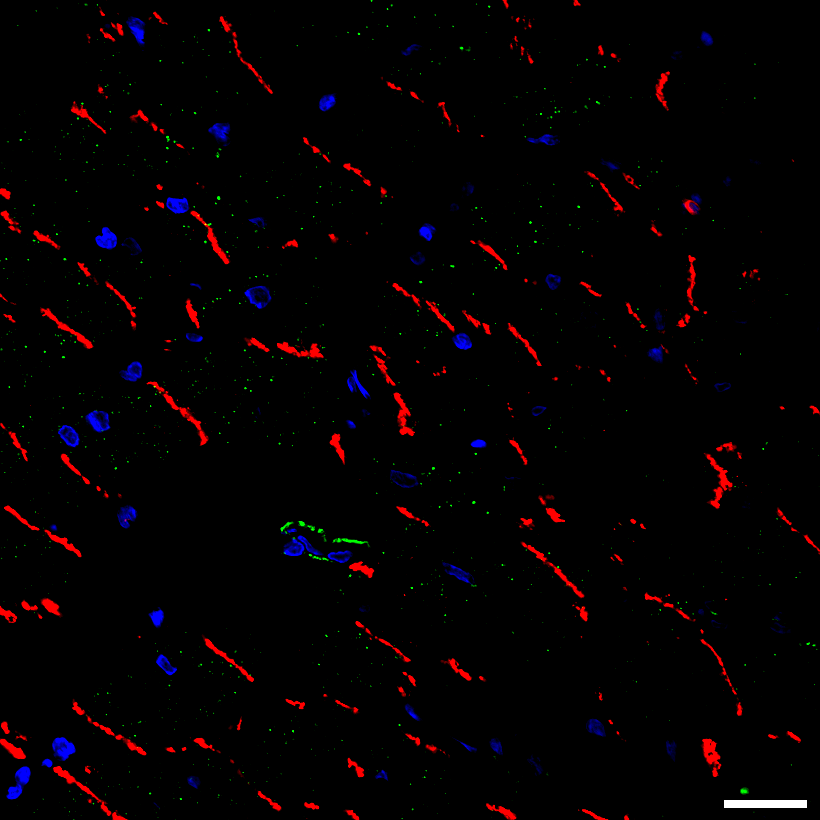

Supplement: Supplemental Information 13 [file peerj-13-19276-s013.zip › immunofluorescence EB1 - N-cadherin-AAV9-CON/AAV9-CON1-2.tif]

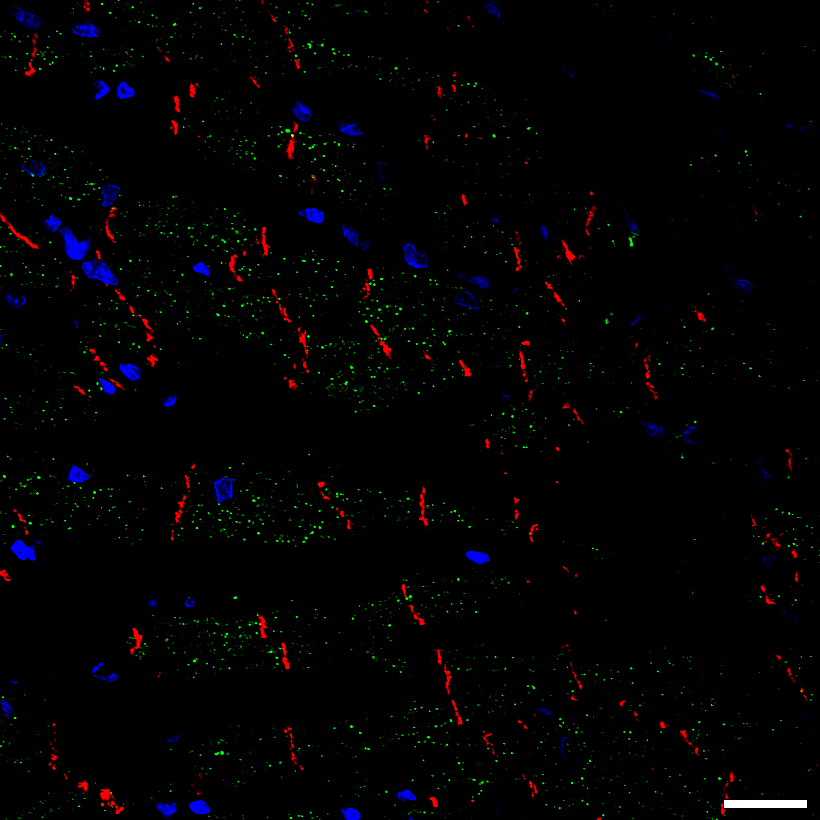

Supplement: Supplemental Information 13 [file peerj-13-19276-s013.zip › immunofluorescence EB1 - N-cadherin-AAV9-CON/AAV9-CON2-1.tif]

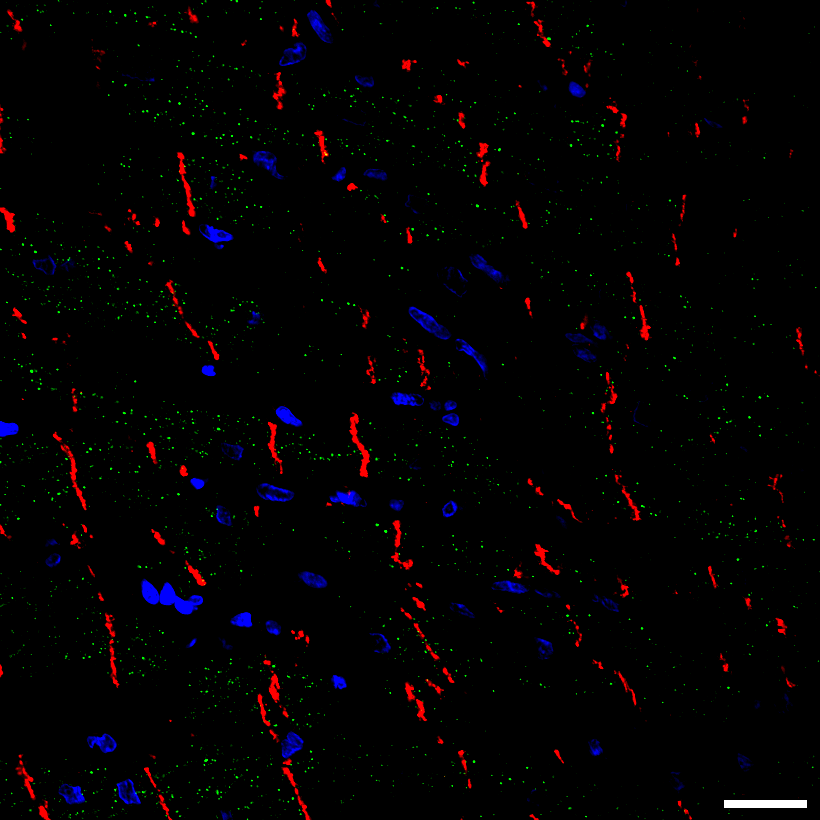

Supplement: Supplemental Information 13 [file peerj-13-19276-s013.zip › immunofluorescence EB1 - N-cadherin-AAV9-CON/AAV9-CON2-2.tif]

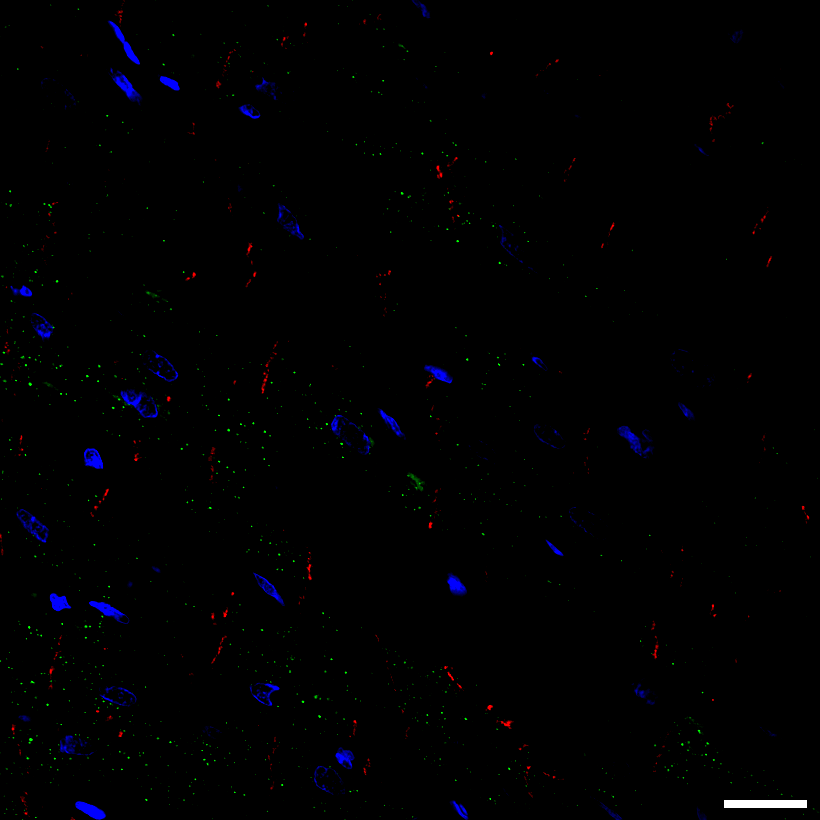

Supplement: Supplemental Information 13 [file peerj-13-19276-s013.zip › immunofluorescence EB1 - N-cadherin-AAV9-CON/AAV9-CON3-1.tif]

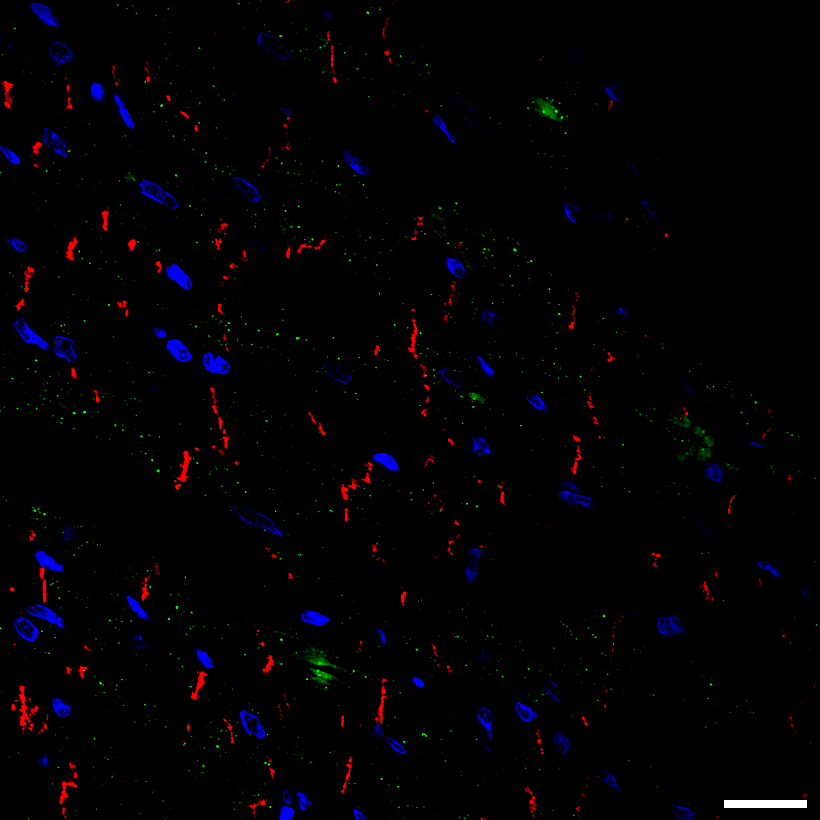

Supplement: Supplemental Information 13 [file peerj-13-19276-s013.zip › immunofluorescence EB1 - N-cadherin-AAV9-CON/AAV9-CON3-2.tif]

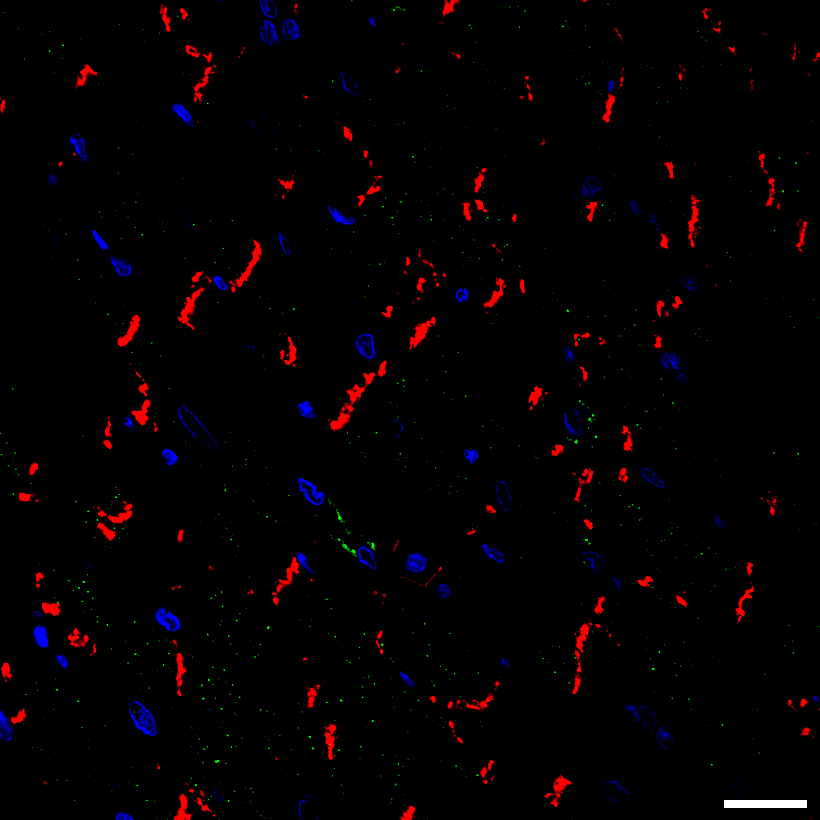

Supplement: Supplemental Information 13 [file peerj-13-19276-s013.zip › immunofluorescence EB1 - N-cadherin-AAV9-CON/AAV9-CON4-1.tif]

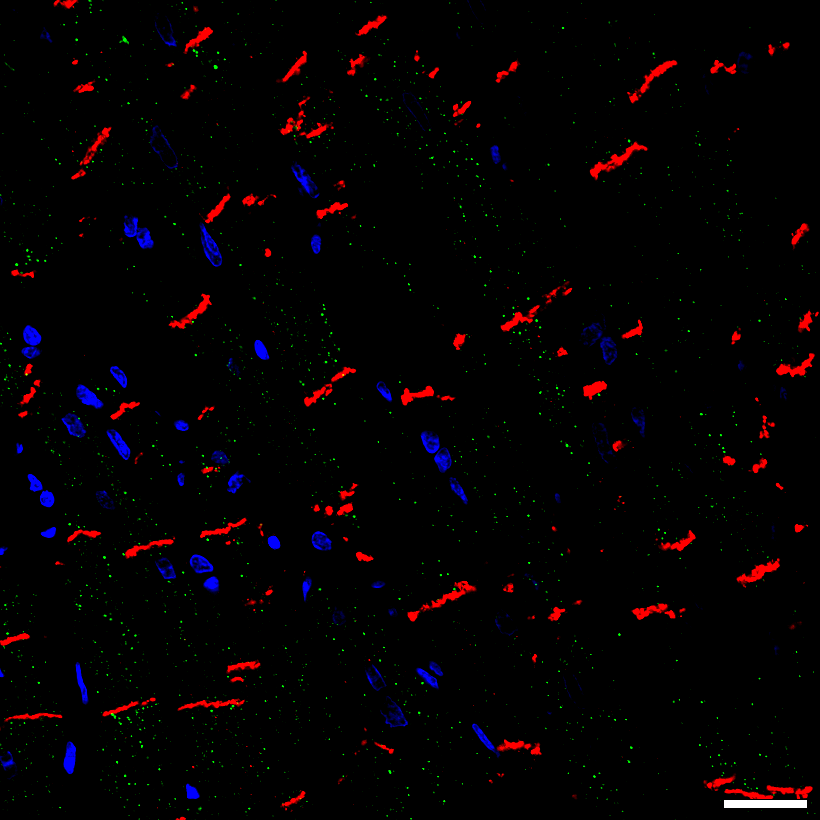

Supplement: Supplemental Information 13 [file peerj-13-19276-s013.zip › immunofluorescence EB1 - N-cadherin-AAV9-CON/AAV9-CON4-2.tif]

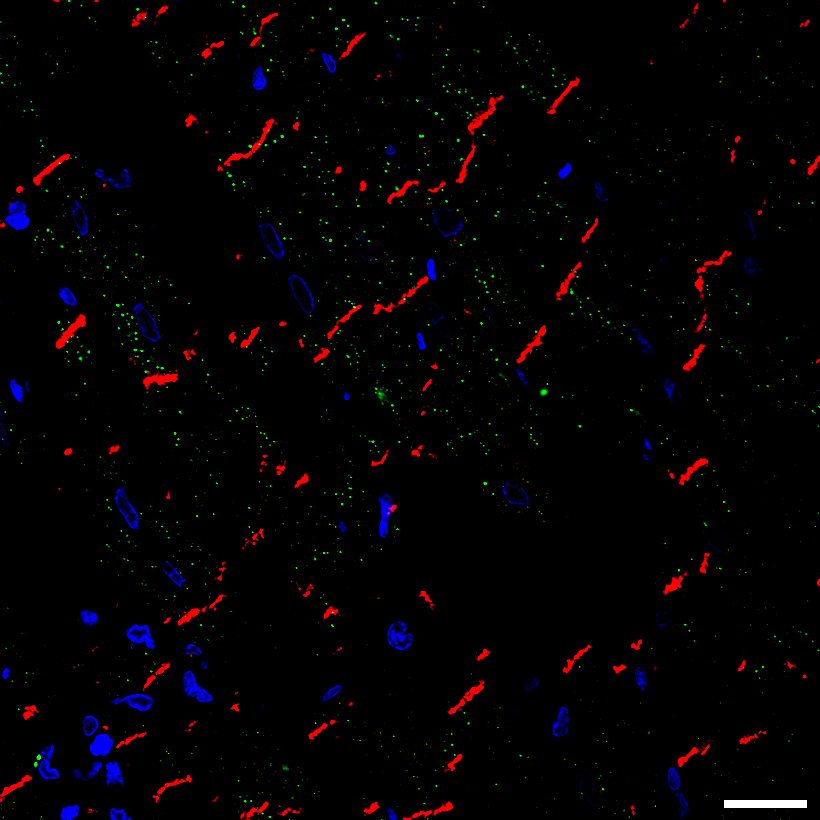

Supplement: Supplemental Information 13 [file peerj-13-19276-s013.zip › immunofluorescence EB1 - N-cadherin-AAV9-CON/AAV9-CON5-1.tif]

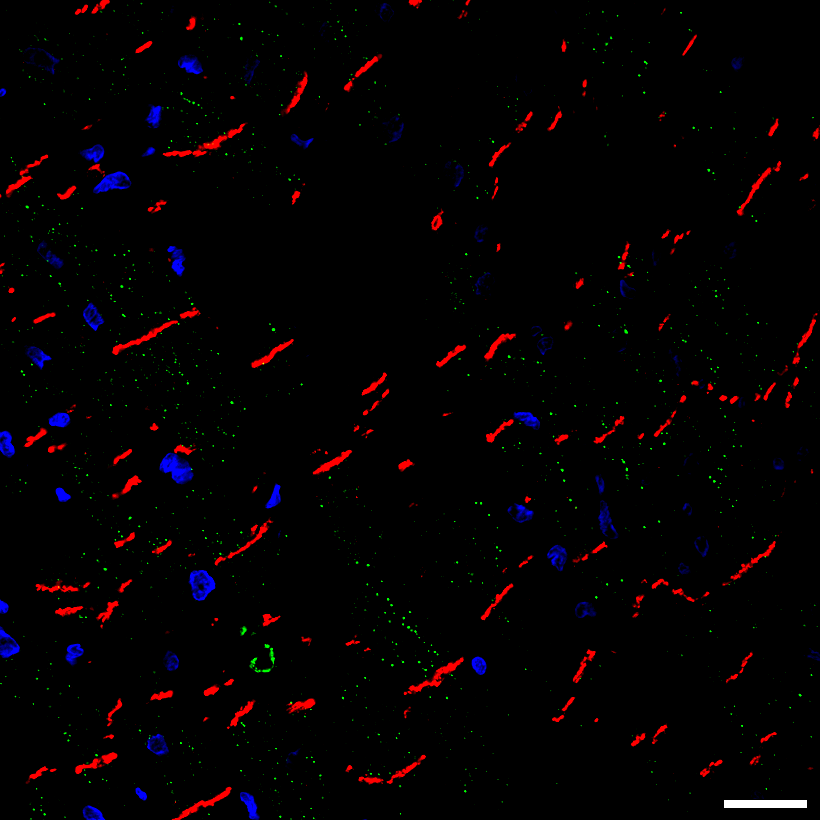

Supplement: Supplemental Information 13 [file peerj-13-19276-s013.zip › immunofluorescence EB1 - N-cadherin-AAV9-CON/AAV9-CON5-2.tif]

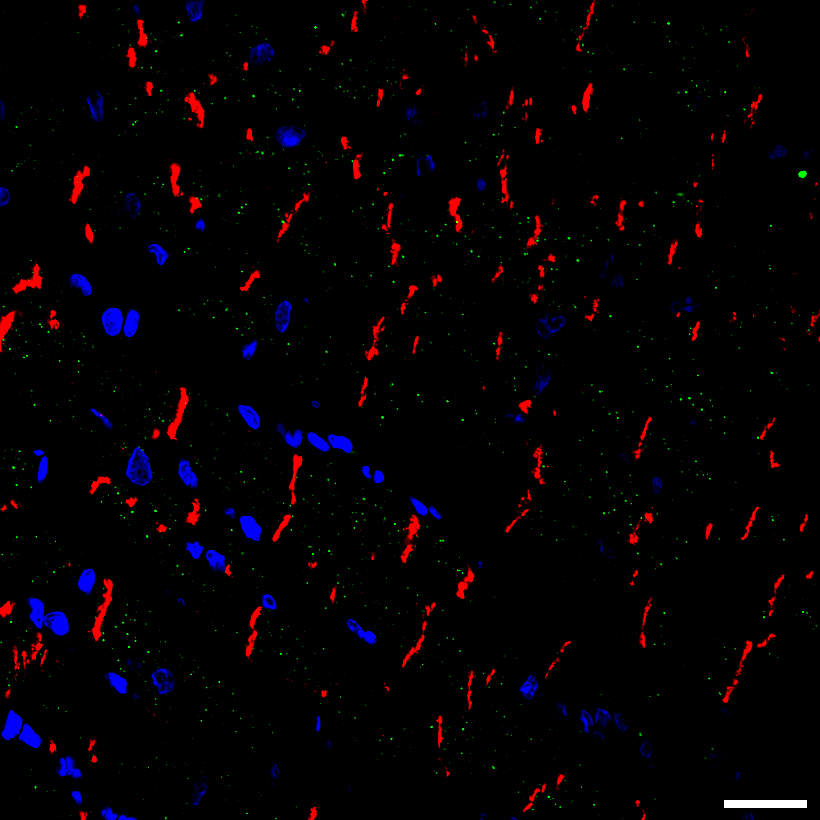

Supplement: Supplemental Information 13 [file peerj-13-19276-s013.zip › immunofluorescence EB1 - N-cadherin-AAV9-CON/AAV9-CON6-1.tif]

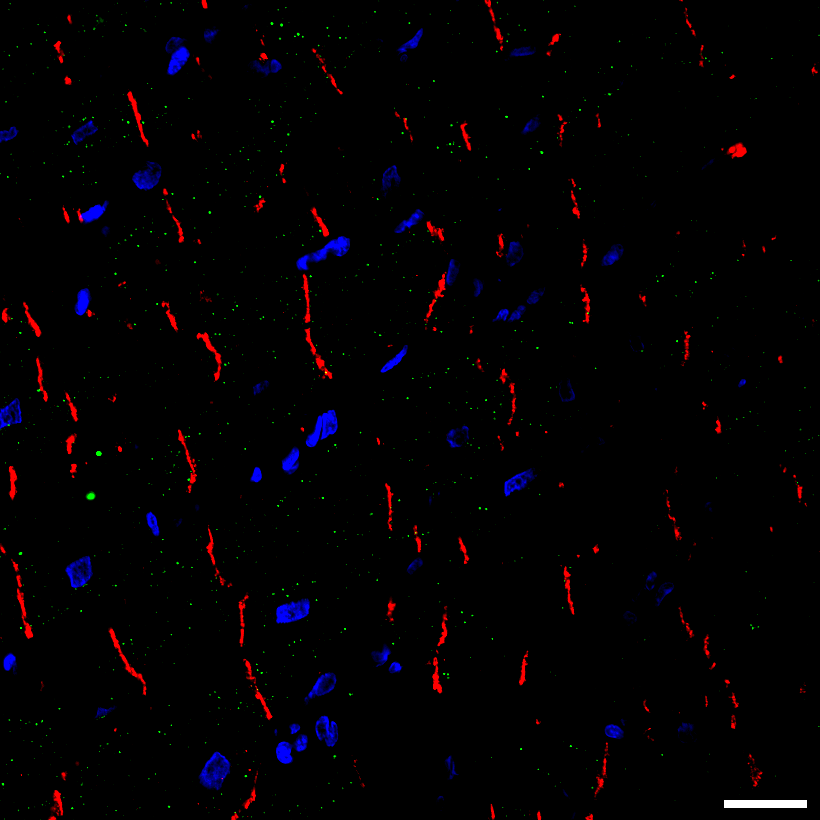

Supplement: Supplemental Information 13 [file peerj-13-19276-s013.zip › immunofluorescence EB1 - N-cadherin-AAV9-CON/AAV9-CON6-2.tif]

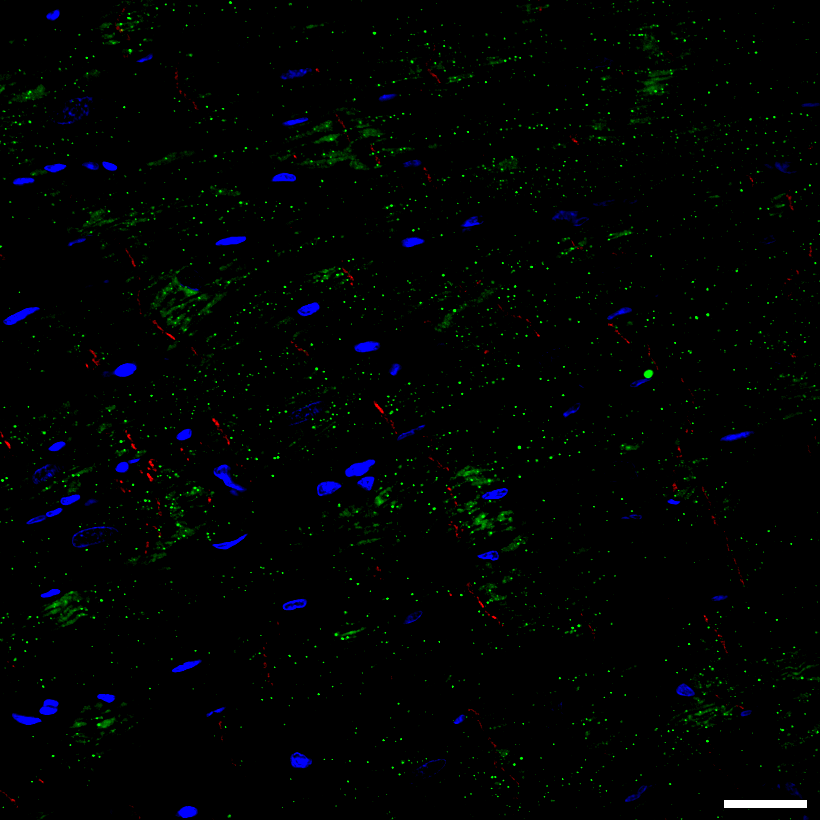

Supplement: Supplemental Information 13 [file peerj-13-19276-s013.zip › immunofluorescence EB1 - N-cadherin-AAV9-CON/AAV9-CON7-1.tif]

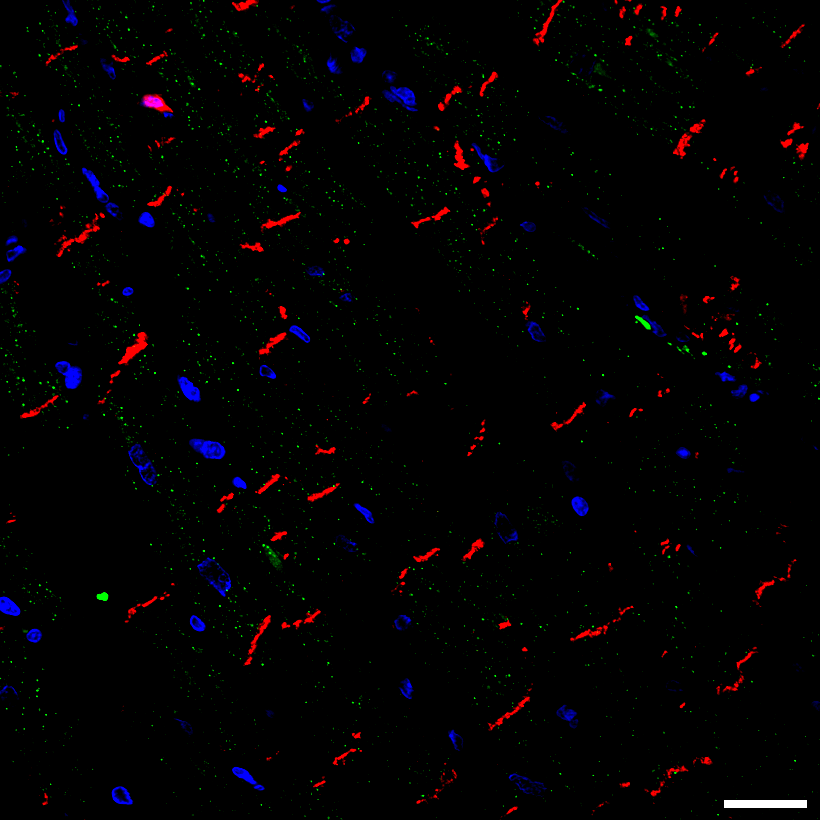

Supplement: Supplemental Information 13 [file peerj-13-19276-s013.zip › immunofluorescence EB1 - N-cadherin-AAV9-CON/AAV9-CON7-2.tif]

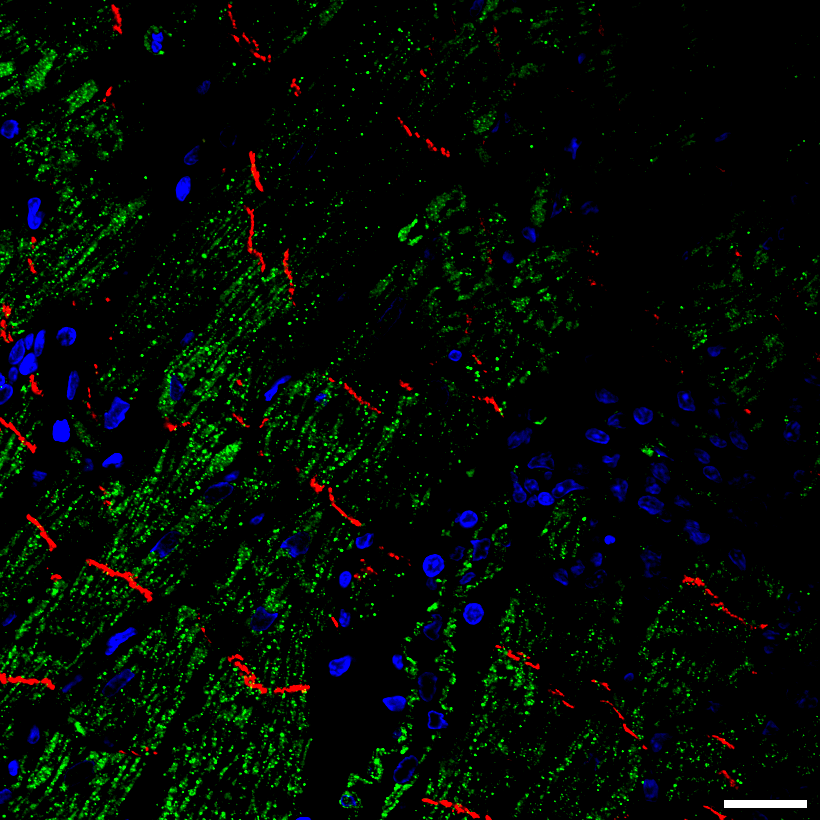

Supplement: Supplemental Information 14 [file peerj-13-19276-s014.zip › immunofluorescence EB1 - N-cadherin-AAV9-EB1/AAV9-EB1 1-1.tif]

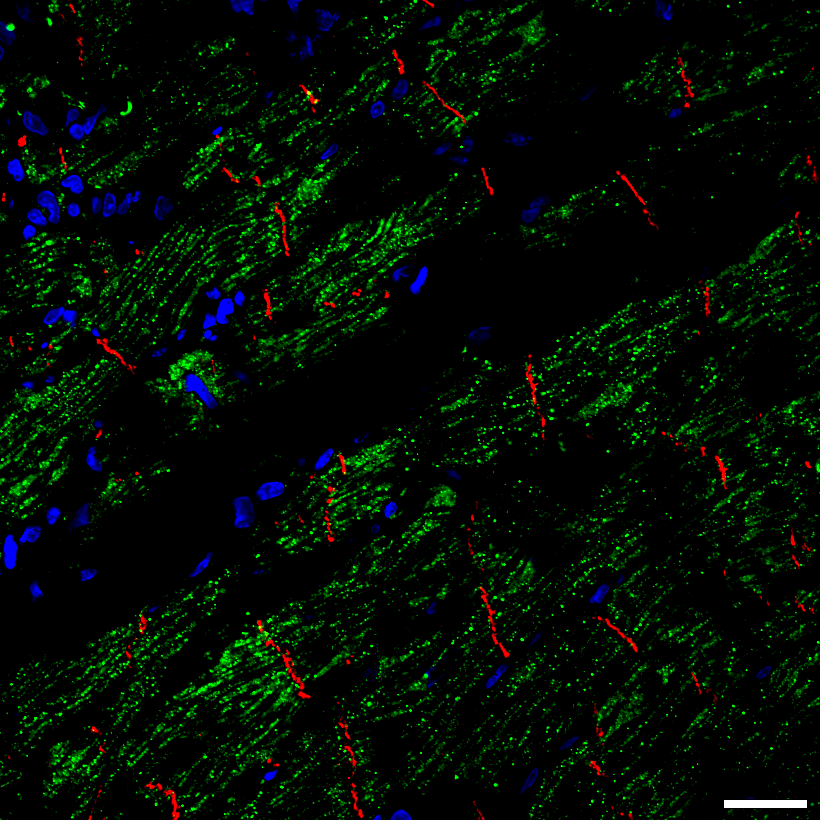

Supplement: Supplemental Information 14 [file peerj-13-19276-s014.zip › immunofluorescence EB1 - N-cadherin-AAV9-EB1/AAV9-EB1 1-2.tif]

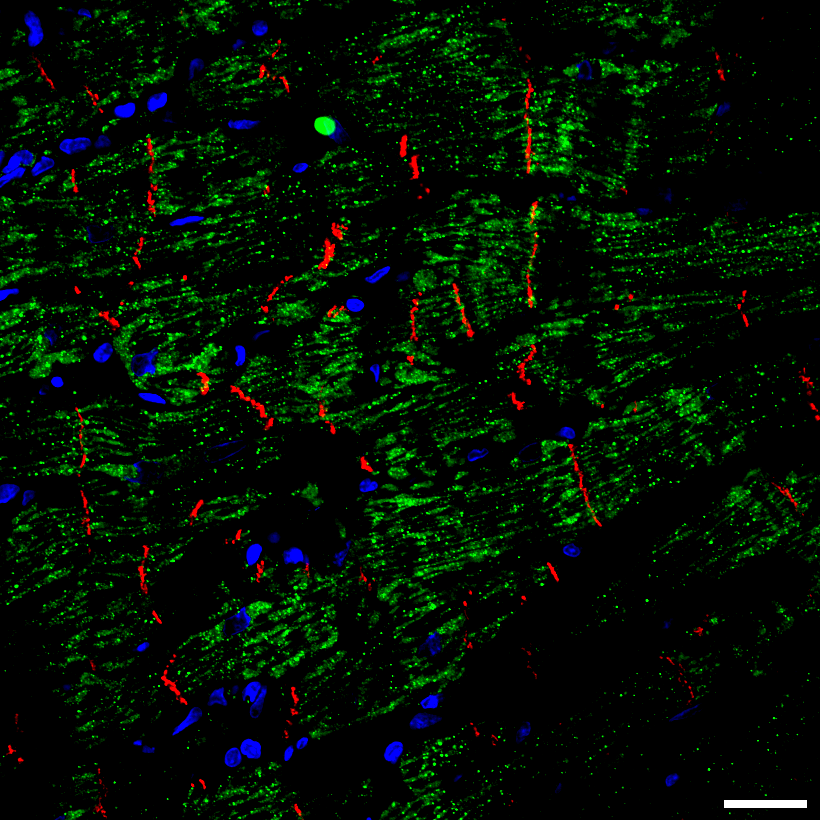

Supplement: Supplemental Information 14 [file peerj-13-19276-s014.zip › immunofluorescence EB1 - N-cadherin-AAV9-EB1/AAV9-EB1 2-1.tif]

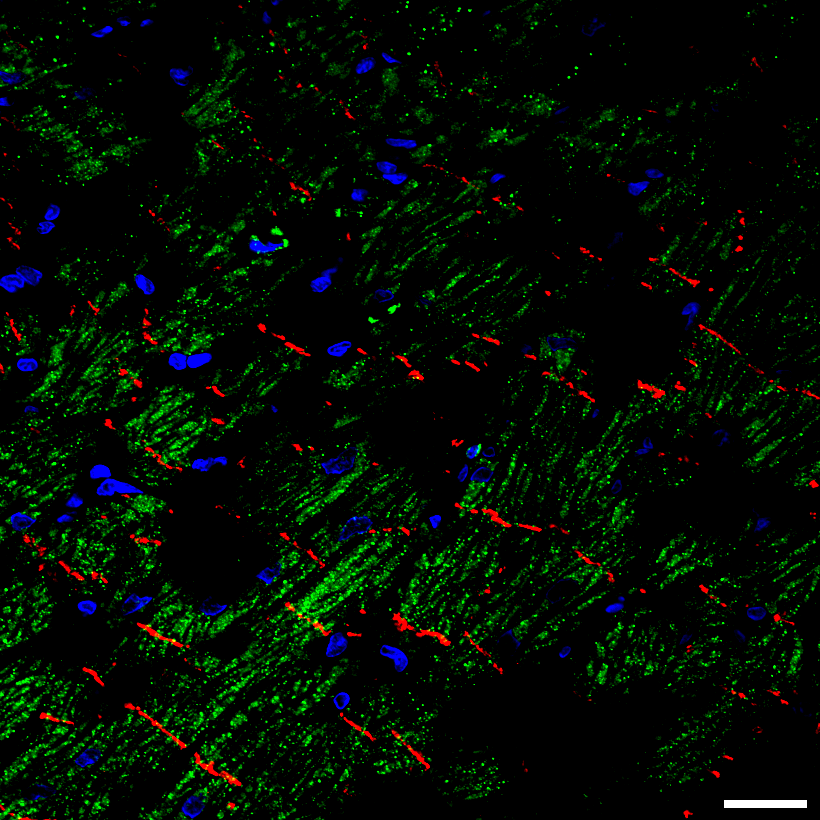

Supplement: Supplemental Information 14 [file peerj-13-19276-s014.zip › immunofluorescence EB1 - N-cadherin-AAV9-EB1/AAV9-EB1 2-2.tif]

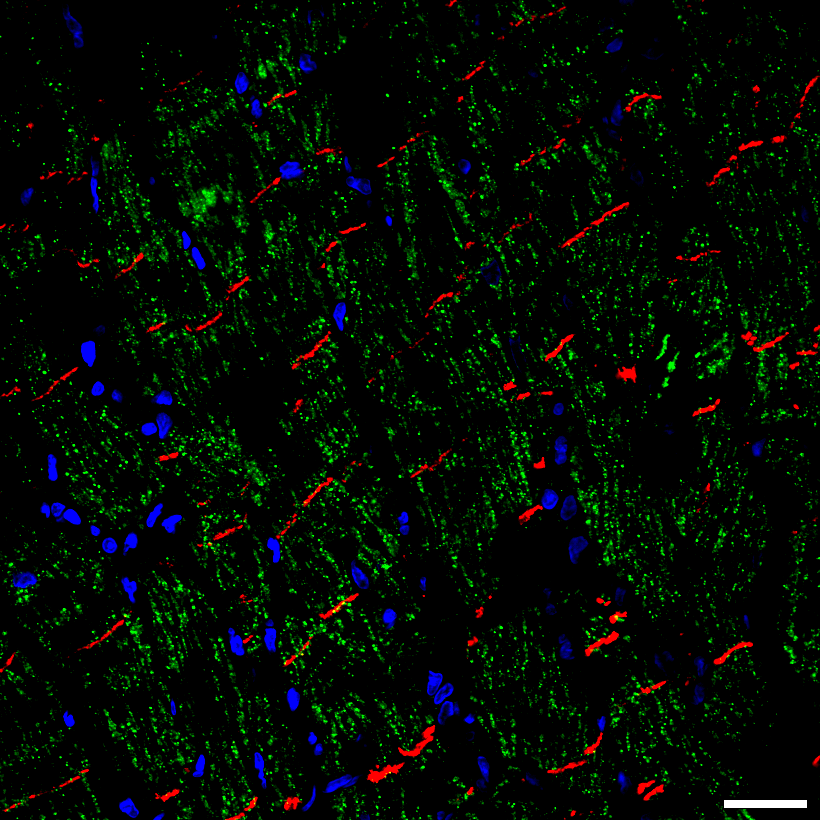

Supplement: Supplemental Information 14 [file peerj-13-19276-s014.zip › immunofluorescence EB1 - N-cadherin-AAV9-EB1/AAV9-EB1 3-1.tif]

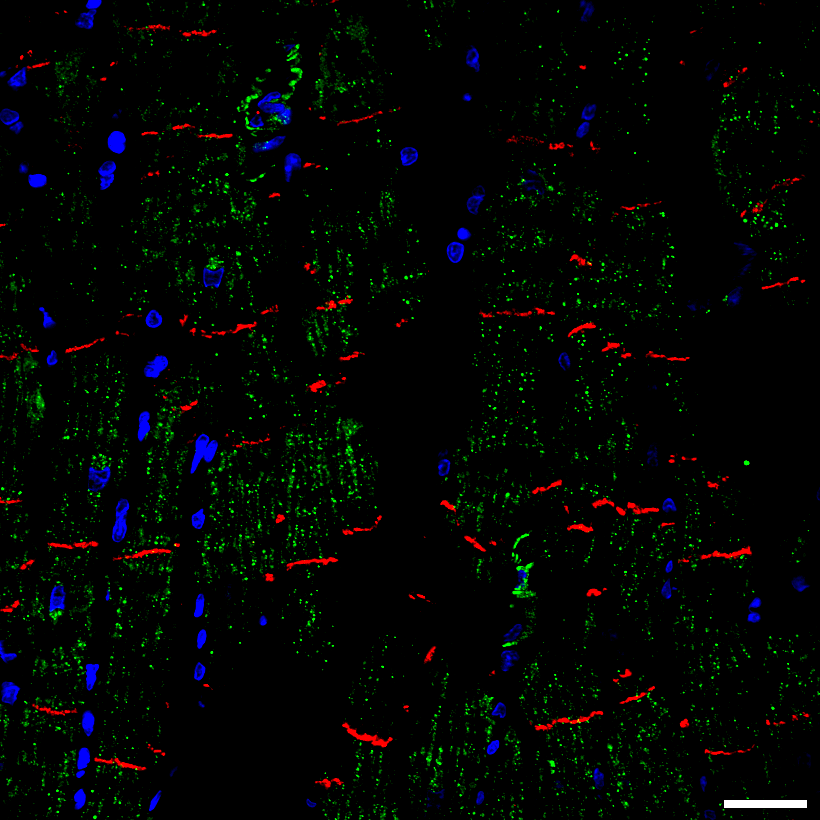

Supplement: Supplemental Information 14 [file peerj-13-19276-s014.zip › immunofluorescence EB1 - N-cadherin-AAV9-EB1/AAV9-EB1 3-2.tif]

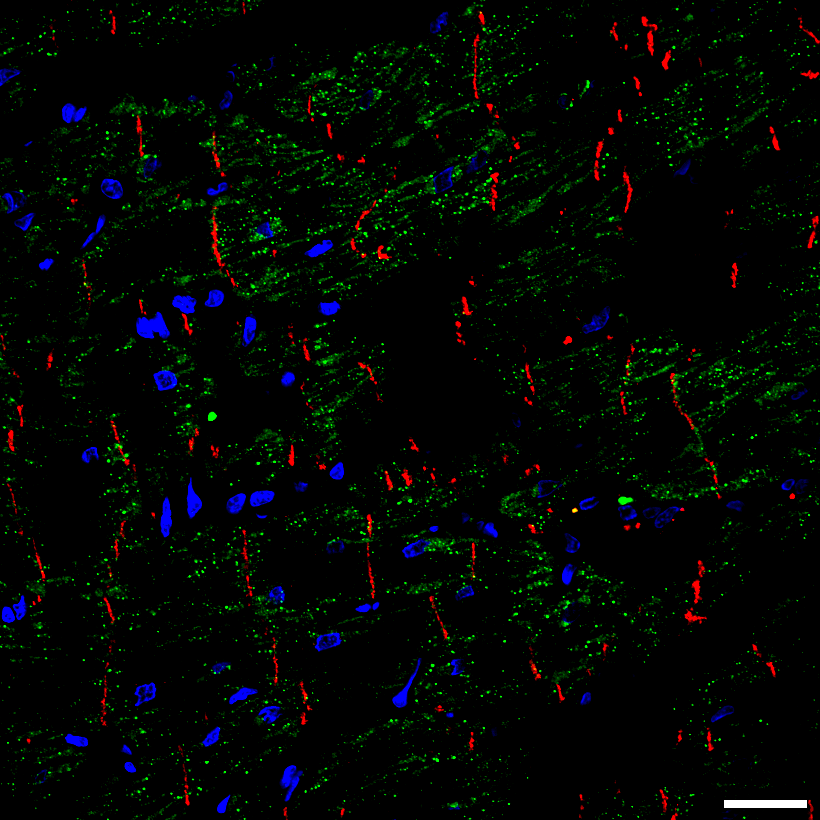

Supplement: Supplemental Information 14 [file peerj-13-19276-s014.zip › immunofluorescence EB1 - N-cadherin-AAV9-EB1/AAV9-EB1 4-1.tif]

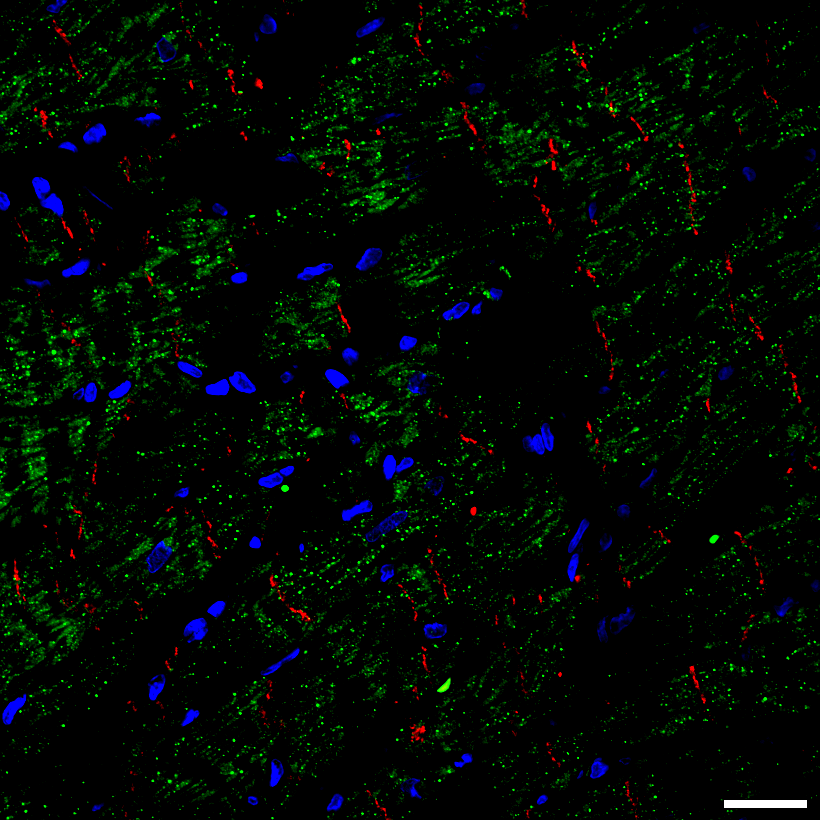

Supplement: Supplemental Information 14 [file peerj-13-19276-s014.zip › immunofluorescence EB1 - N-cadherin-AAV9-EB1/AAV9-EB1 4-2.tif]

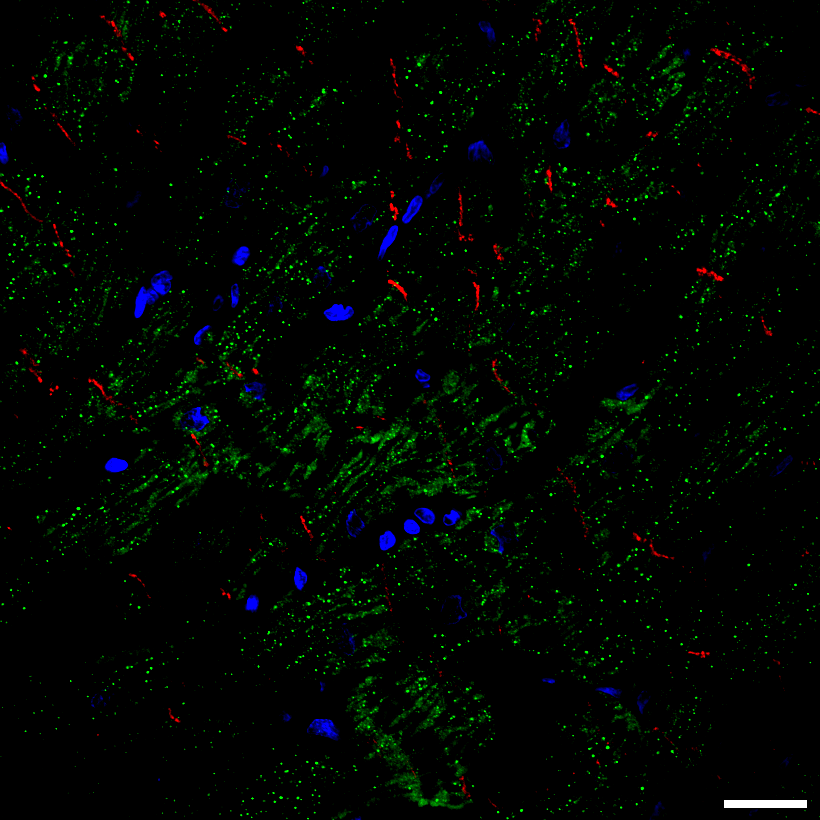

Supplement: Supplemental Information 14 [file peerj-13-19276-s014.zip › immunofluorescence EB1 - N-cadherin-AAV9-EB1/AAV9-EB1 5-1.tif]

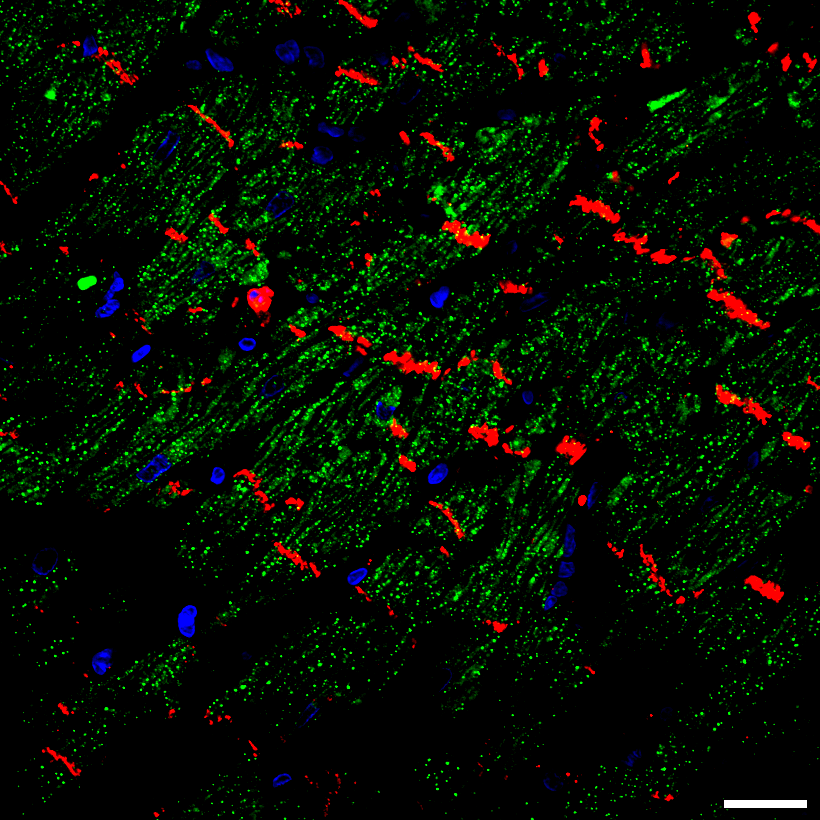

Supplement: Supplemental Information 14 [file peerj-13-19276-s014.zip › immunofluorescence EB1 - N-cadherin-AAV9-EB1/AAV9-EB1 5-2.tif]

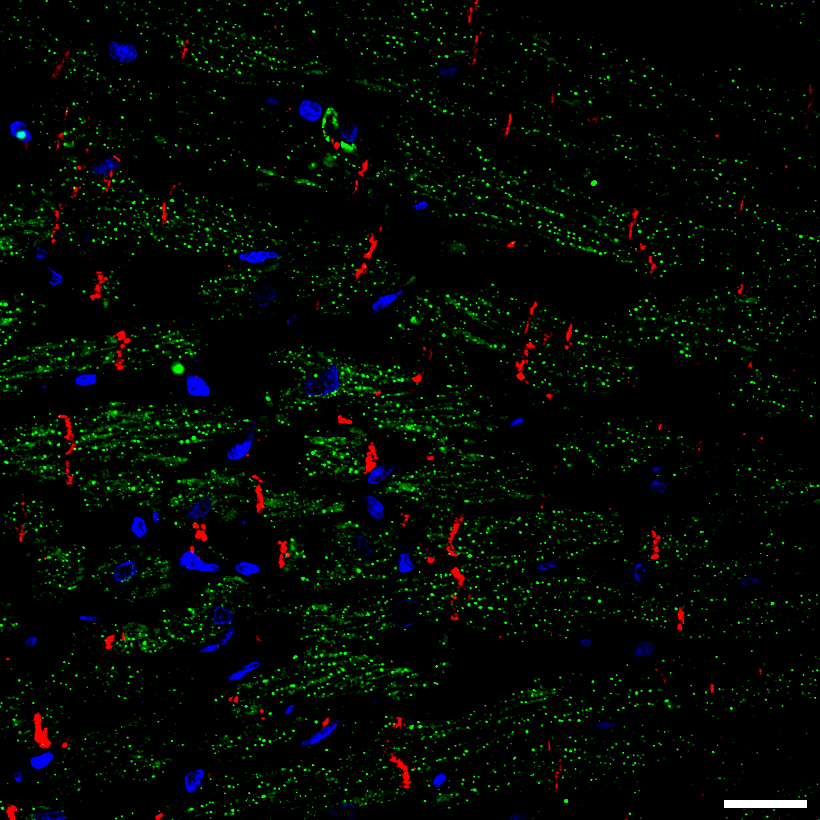

Supplement: Supplemental Information 14 [file peerj-13-19276-s014.zip › immunofluorescence EB1 - N-cadherin-AAV9-EB1/AAV9-EB1 6-1.tif]

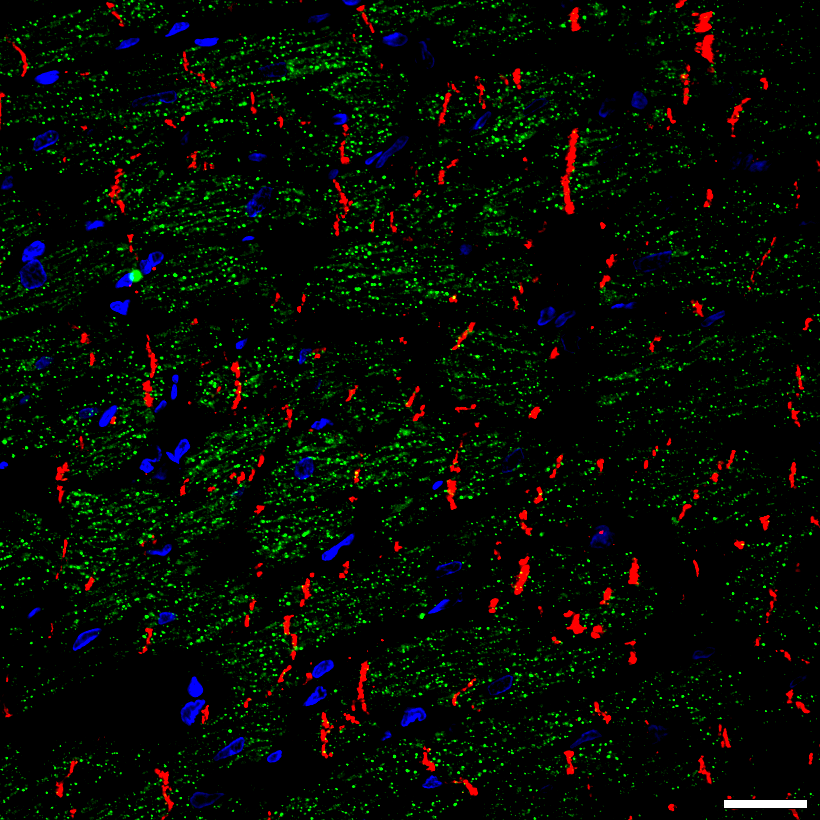

Supplement: Supplemental Information 14 [file peerj-13-19276-s014.zip › immunofluorescence EB1 - N-cadherin-AAV9-EB1/AAV9-EB1 6-2.tif]

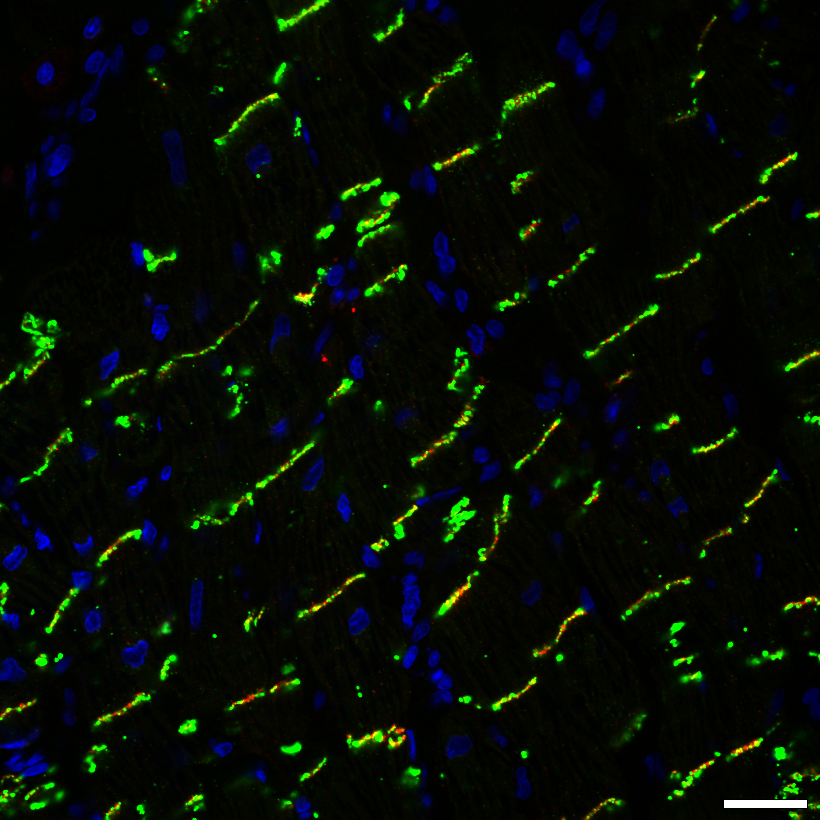

Supplement: Supplemental Information 15 [file peerj-13-19276-s015.zip › immunofluorescence C(Cx43-N-cadherin)/C1-1.tif]

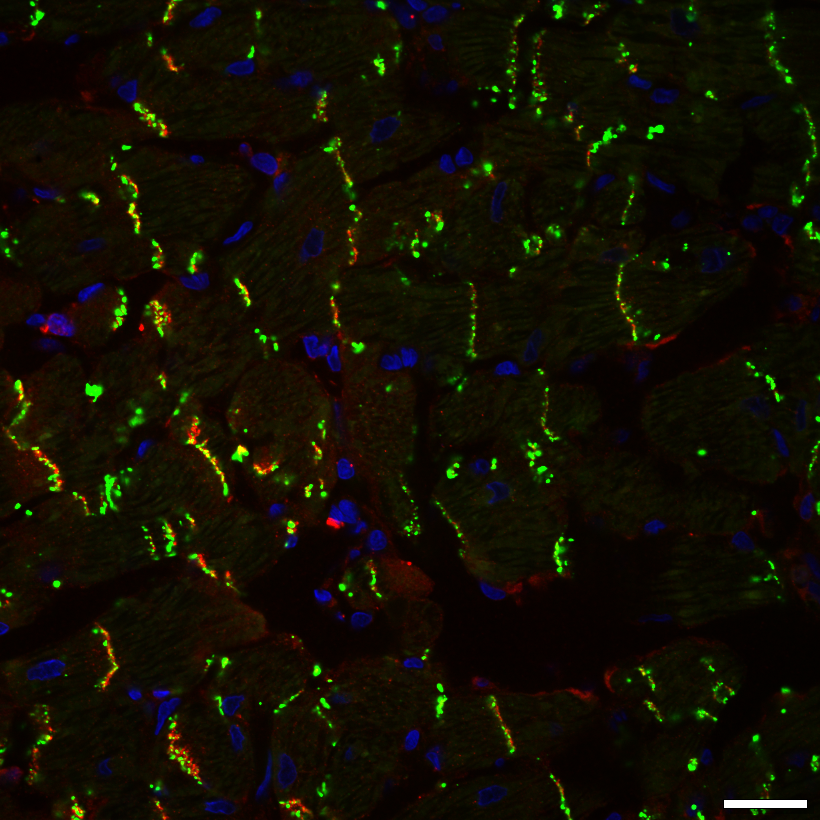

Supplement: Supplemental Information 15 [file peerj-13-19276-s015.zip › immunofluorescence C(Cx43-N-cadherin)/C1-2.tif]

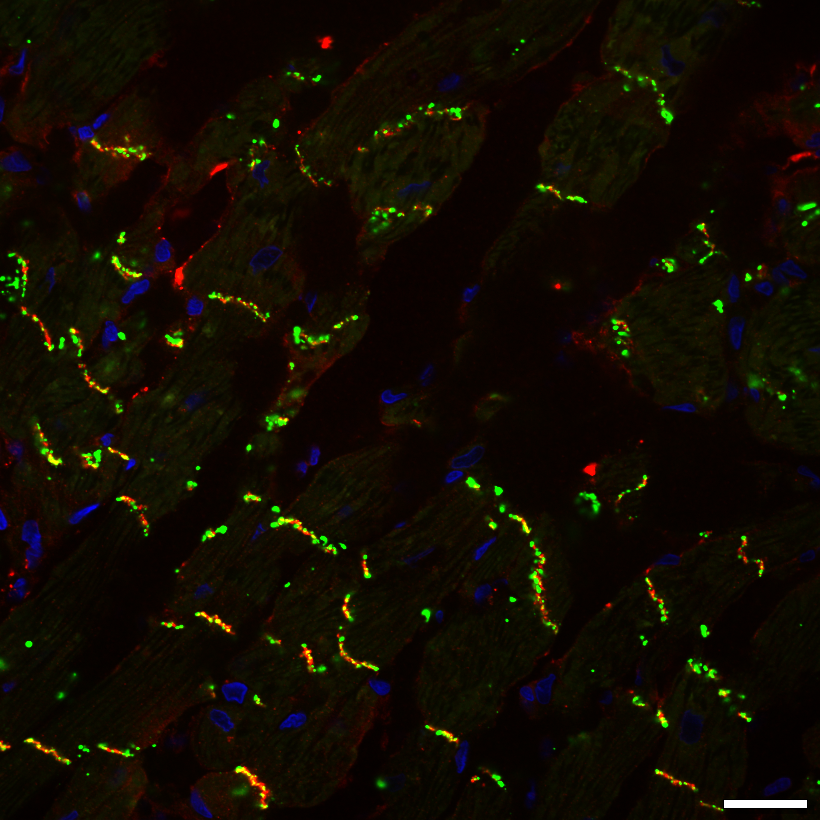

Supplement: Supplemental Information 15 [file peerj-13-19276-s015.zip › immunofluorescence C(Cx43-N-cadherin)/C2-1.tif]

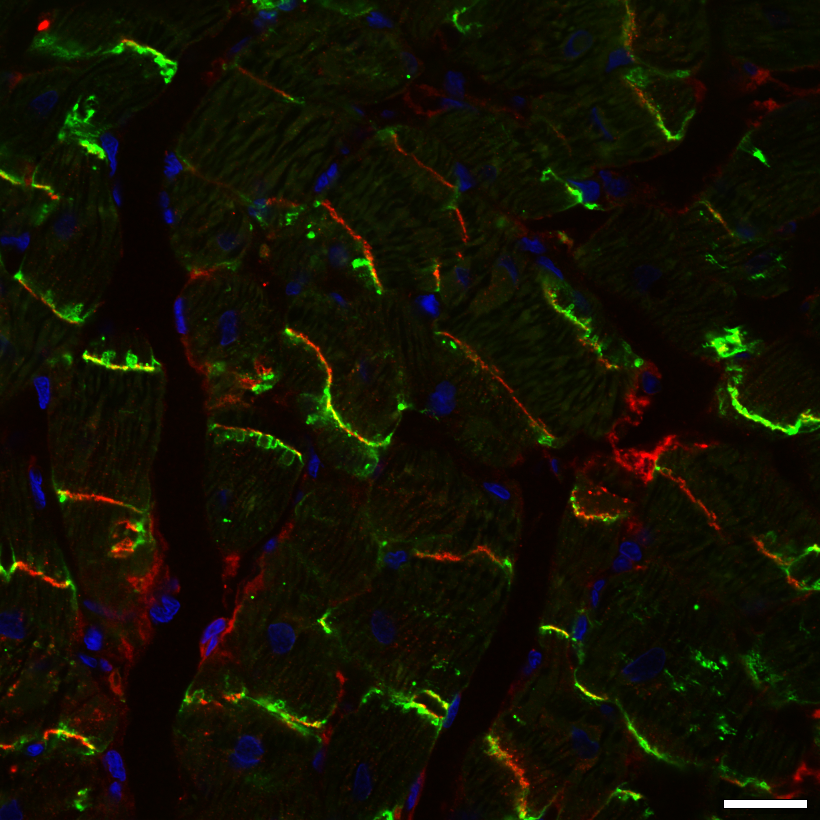

Supplement: Supplemental Information 15 [file peerj-13-19276-s015.zip › immunofluorescence C(Cx43-N-cadherin)/C2-2.tif]

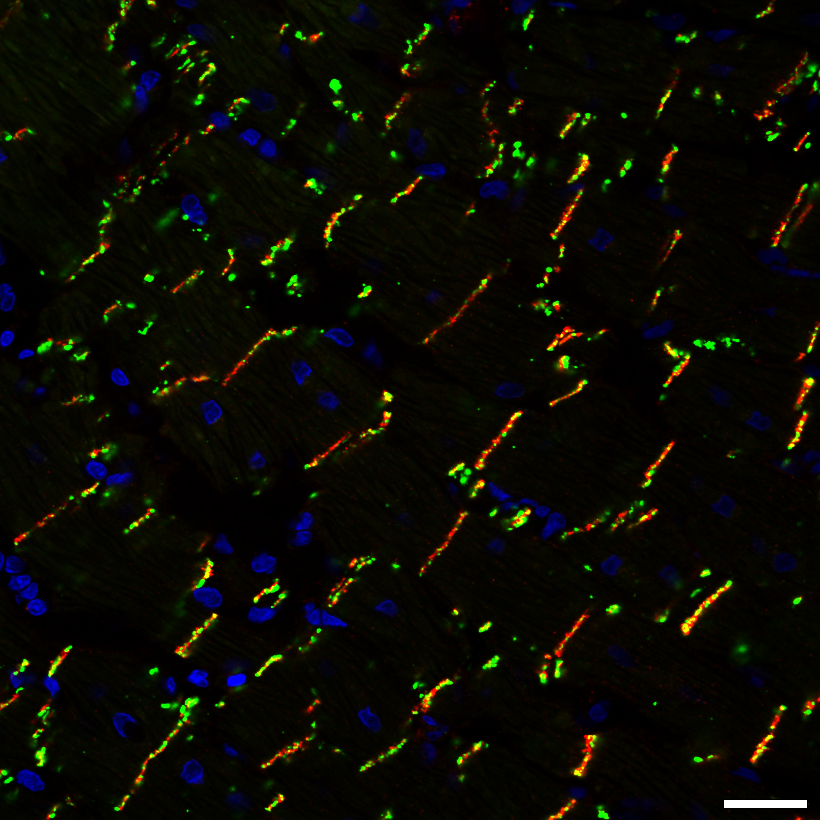

Supplement: Supplemental Information 15 [file peerj-13-19276-s015.zip › immunofluorescence C(Cx43-N-cadherin)/C3-1.tif]

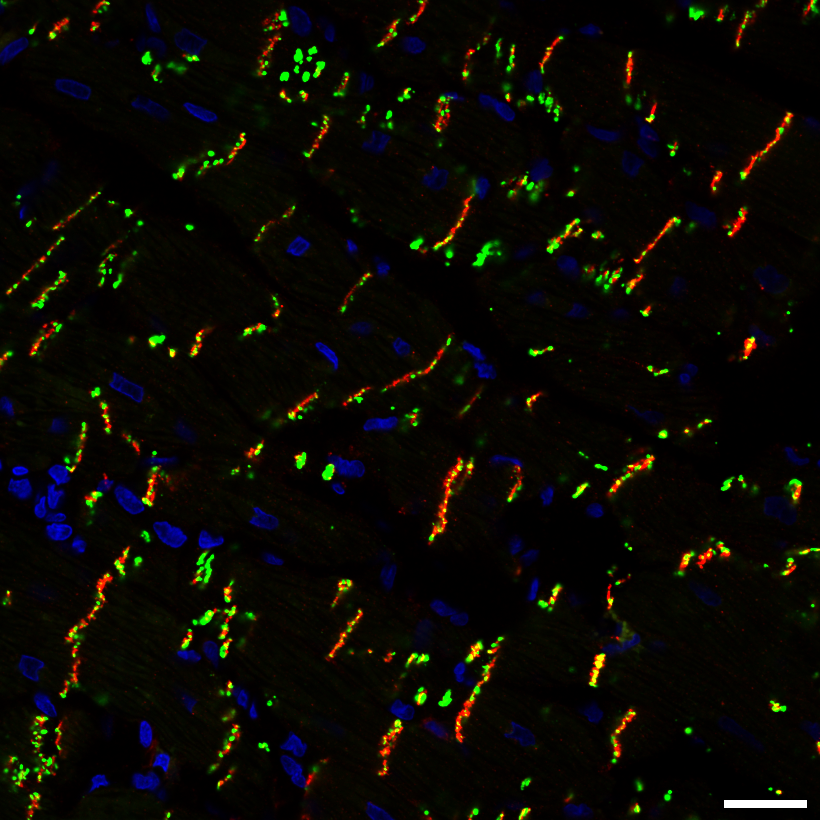

Supplement: Supplemental Information 15 [file peerj-13-19276-s015.zip › immunofluorescence C(Cx43-N-cadherin)/C3-2.tif]

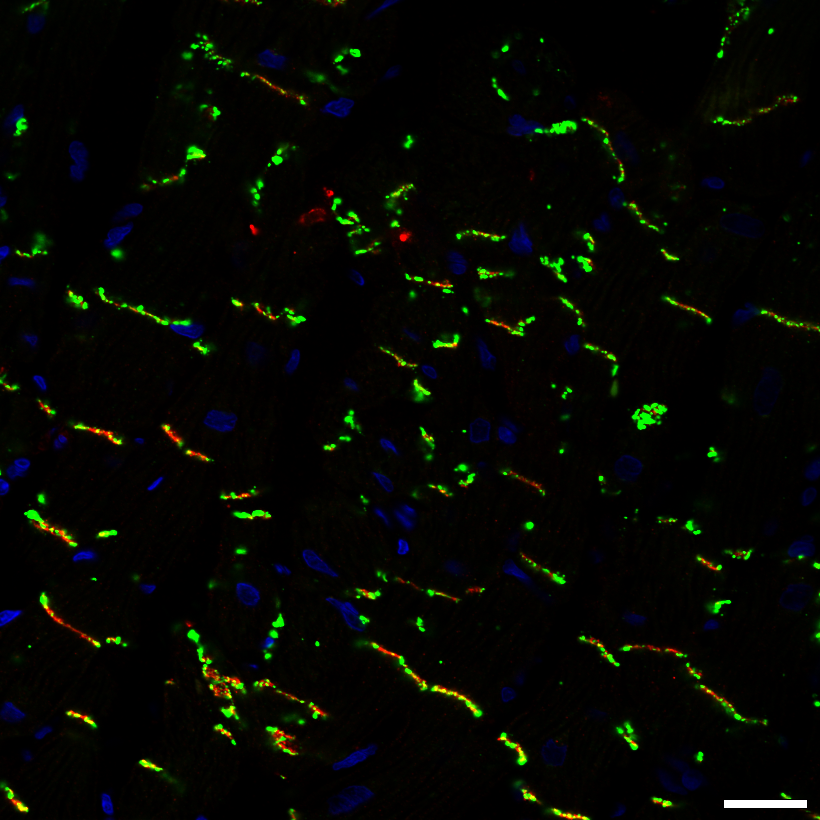

Supplement: Supplemental Information 15 [file peerj-13-19276-s015.zip › immunofluorescence C(Cx43-N-cadherin)/C3-3.tif]

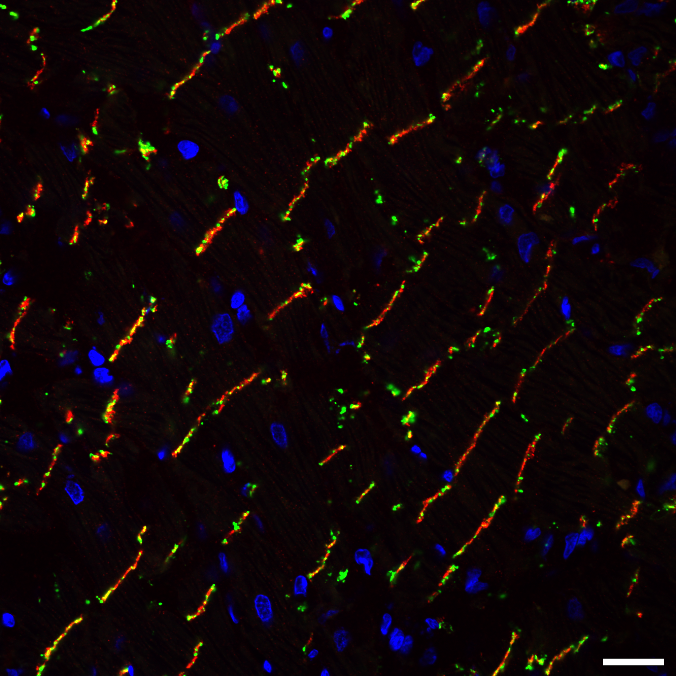

Supplement: Supplemental Information 15 [file peerj-13-19276-s015.zip › immunofluorescence C(Cx43-N-cadherin)/C3-4.tif]

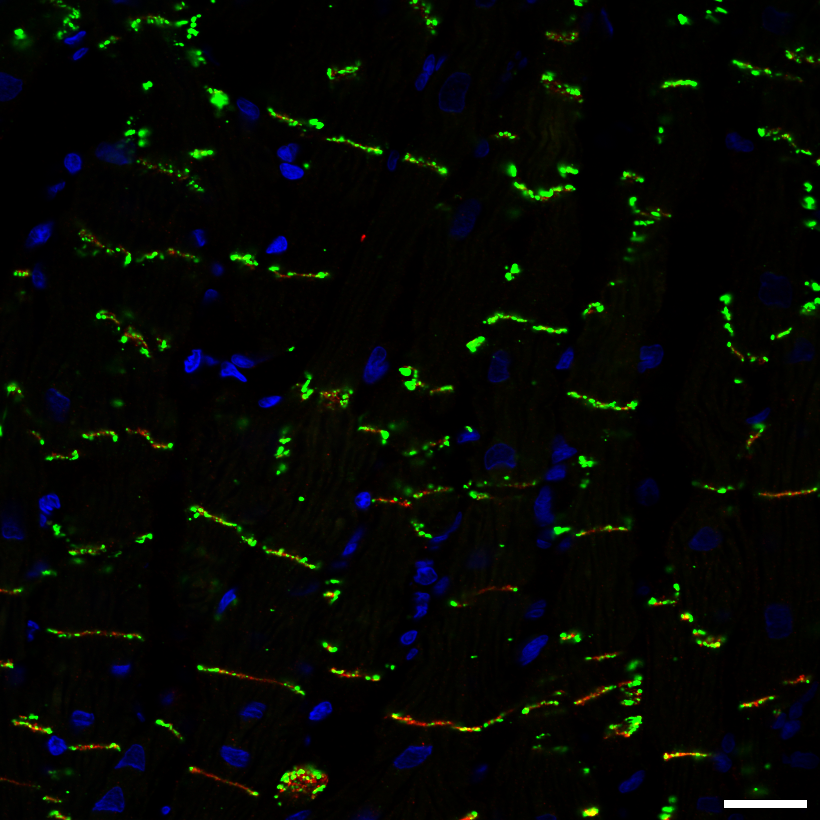

Supplement: Supplemental Information 15 [file peerj-13-19276-s015.zip › immunofluorescence C(Cx43-N-cadherin)/C4-1.tif]

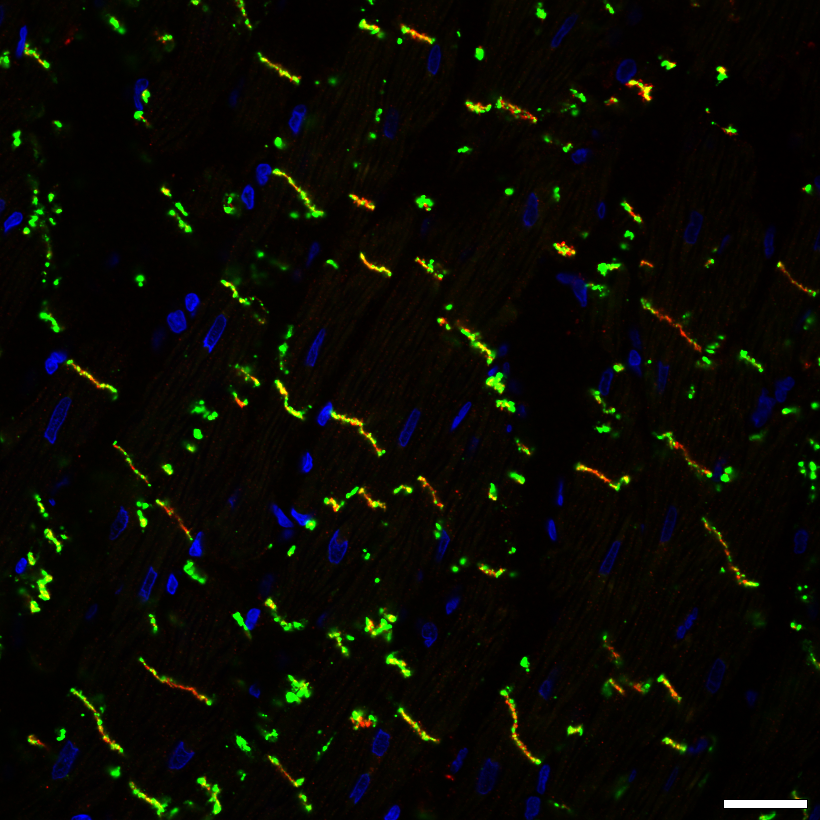

Supplement: Supplemental Information 15 [file peerj-13-19276-s015.zip › immunofluorescence C(Cx43-N-cadherin)/C4-2.tif]

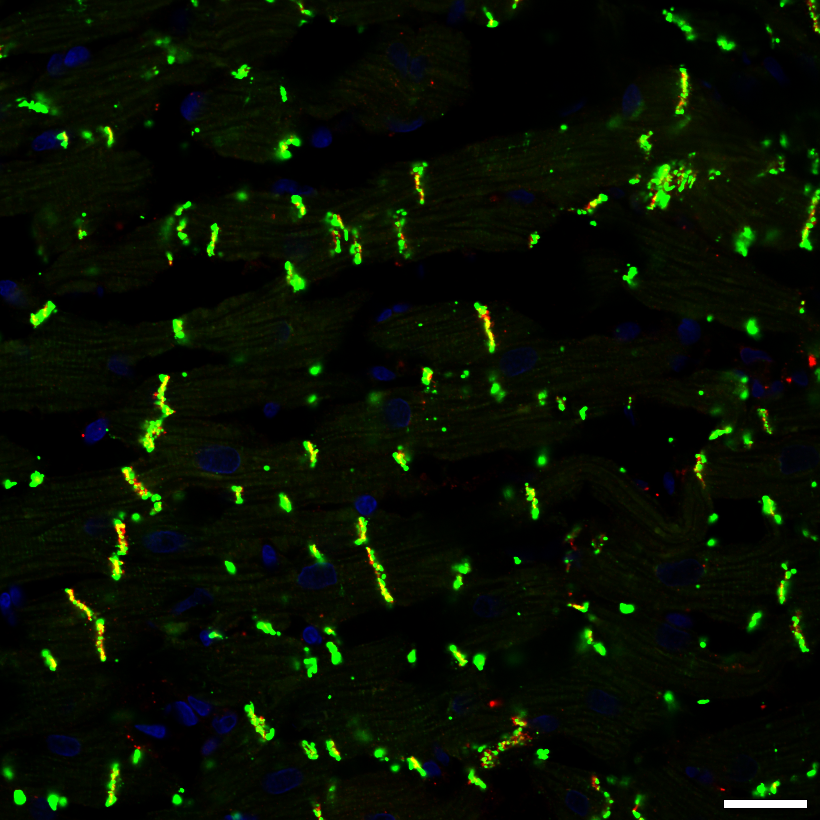

Supplement: Supplemental Information 15 [file peerj-13-19276-s015.zip › immunofluorescence C(Cx43-N-cadherin)/C4-3.tif]

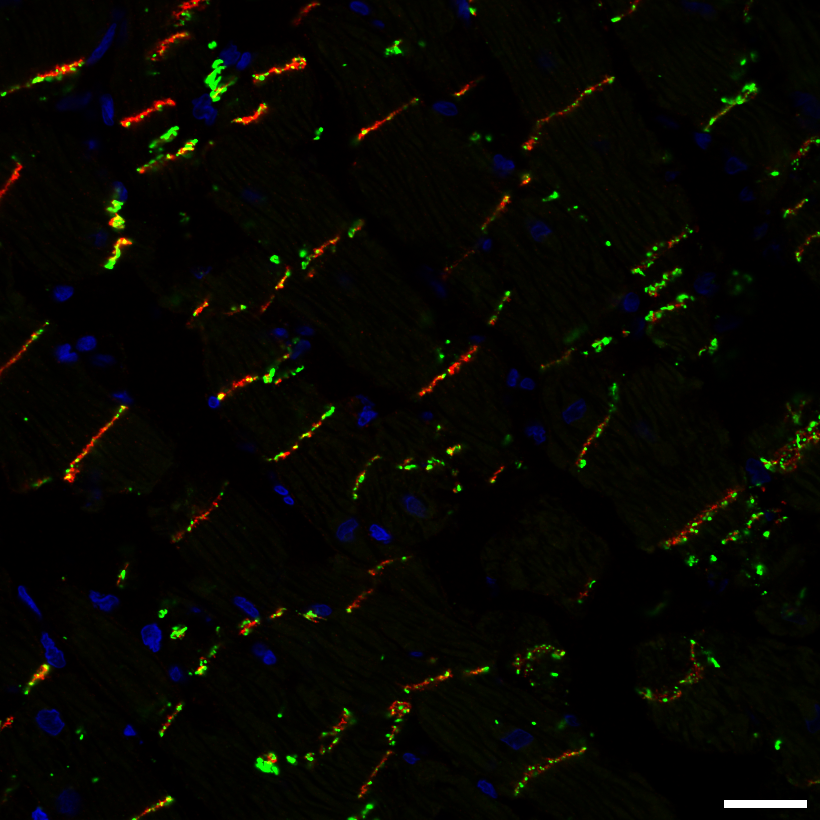

Supplement: Supplemental Information 15 [file peerj-13-19276-s015.zip › immunofluorescence C(Cx43-N-cadherin)/C5-1.tif]

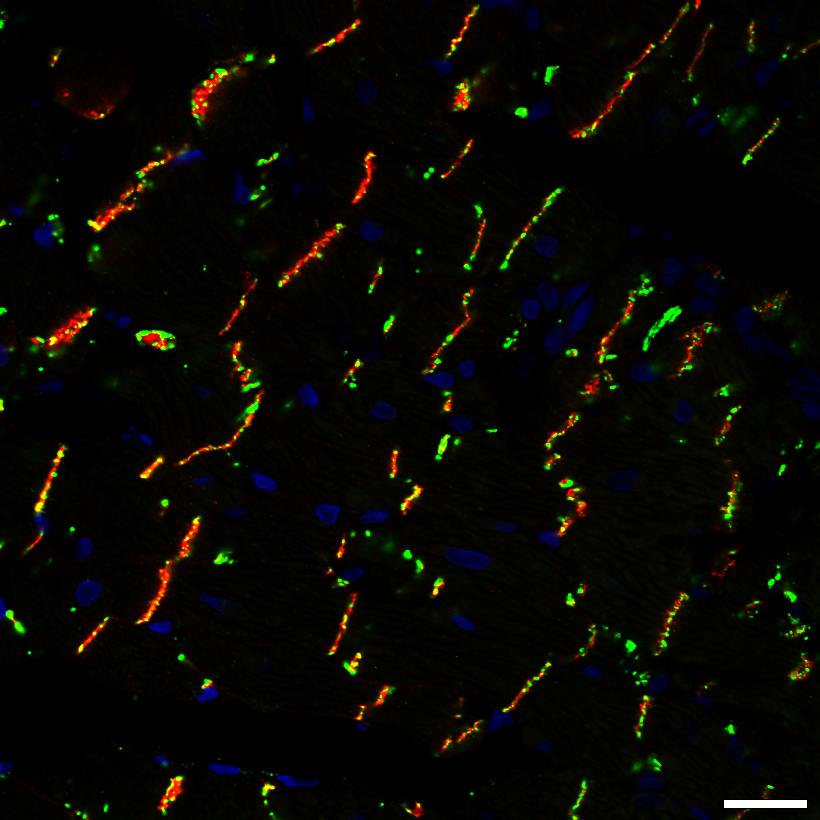

Supplement: Supplemental Information 15 [file peerj-13-19276-s015.zip › immunofluorescence C(Cx43-N-cadherin)/C5-2.tif]

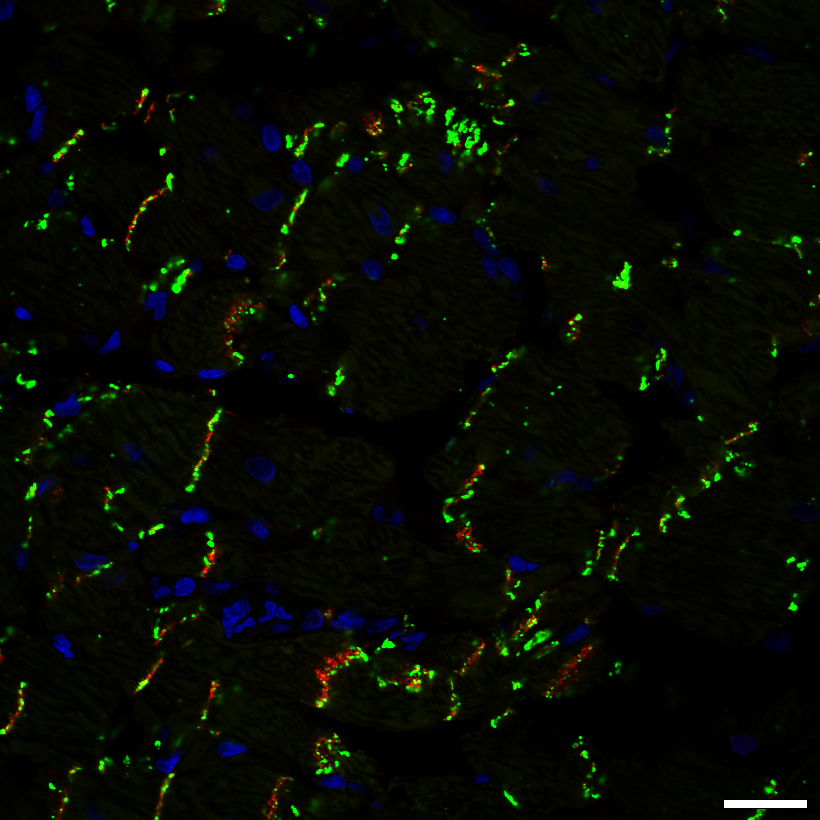

Supplement: Supplemental Information 15 [file peerj-13-19276-s015.zip › immunofluorescence C(Cx43-N-cadherin)/C6-1.tif]

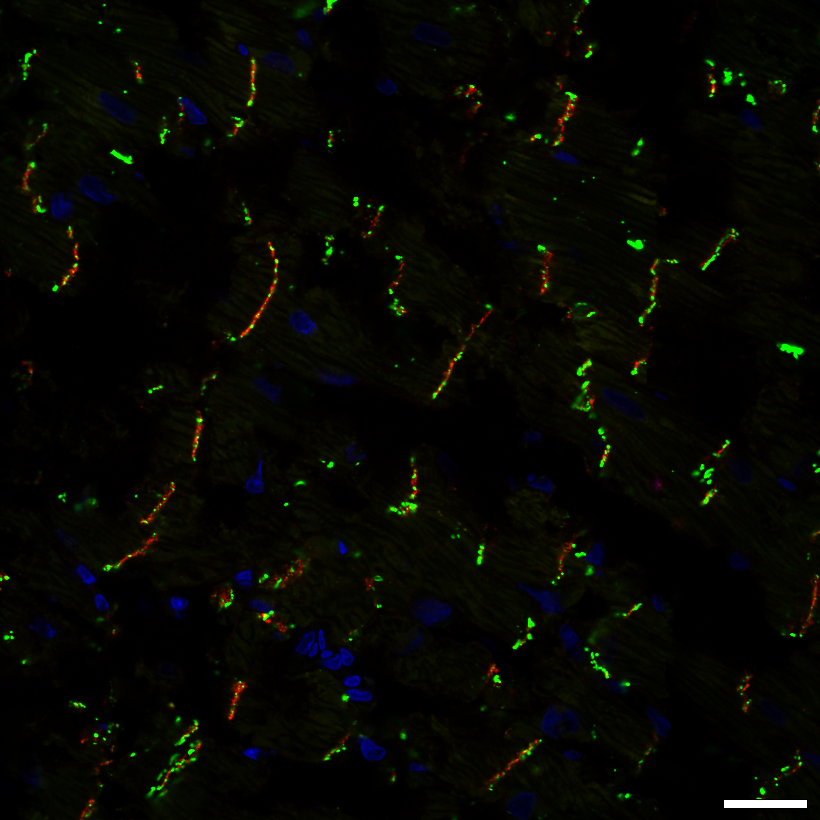

Supplement: Supplemental Information 15 [file peerj-13-19276-s015.zip › immunofluorescence C(Cx43-N-cadherin)/C6-2.tif]

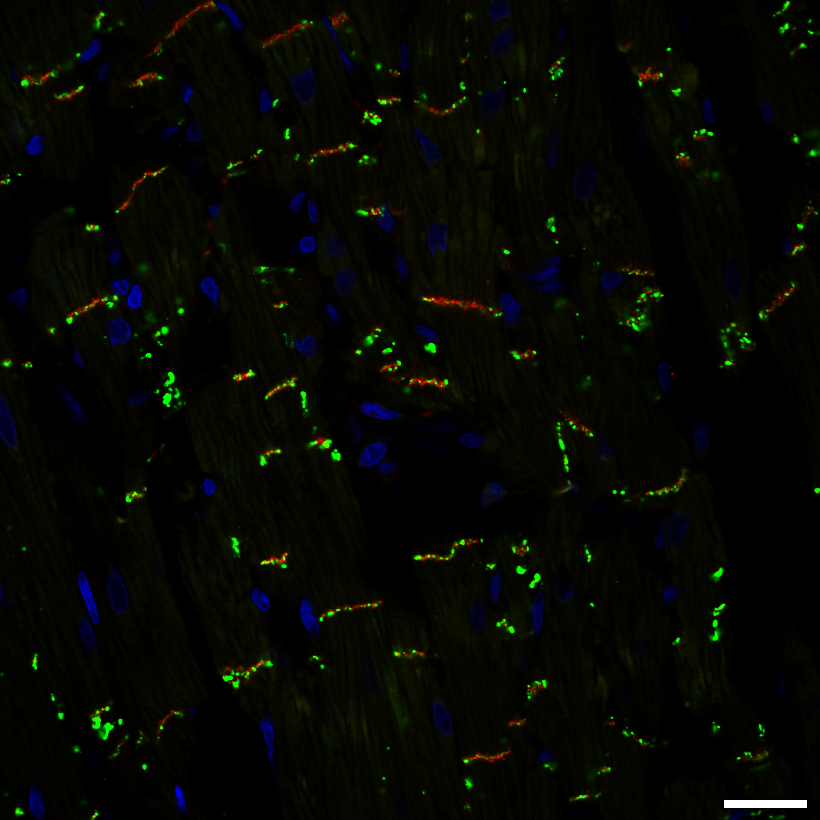

Supplement: Supplemental Information 15 [file peerj-13-19276-s015.zip › immunofluorescence C(Cx43-N-cadherin)/C7-1.tif]

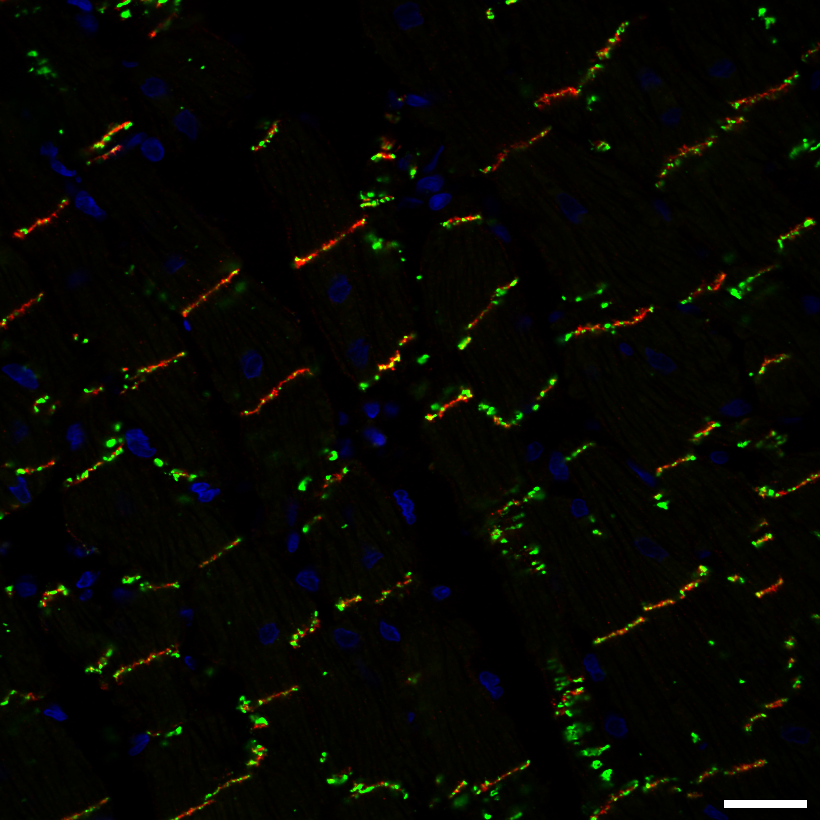

Supplement: Supplemental Information 15 [file peerj-13-19276-s015.zip › immunofluorescence C(Cx43-N-cadherin)/C7-2.tif]

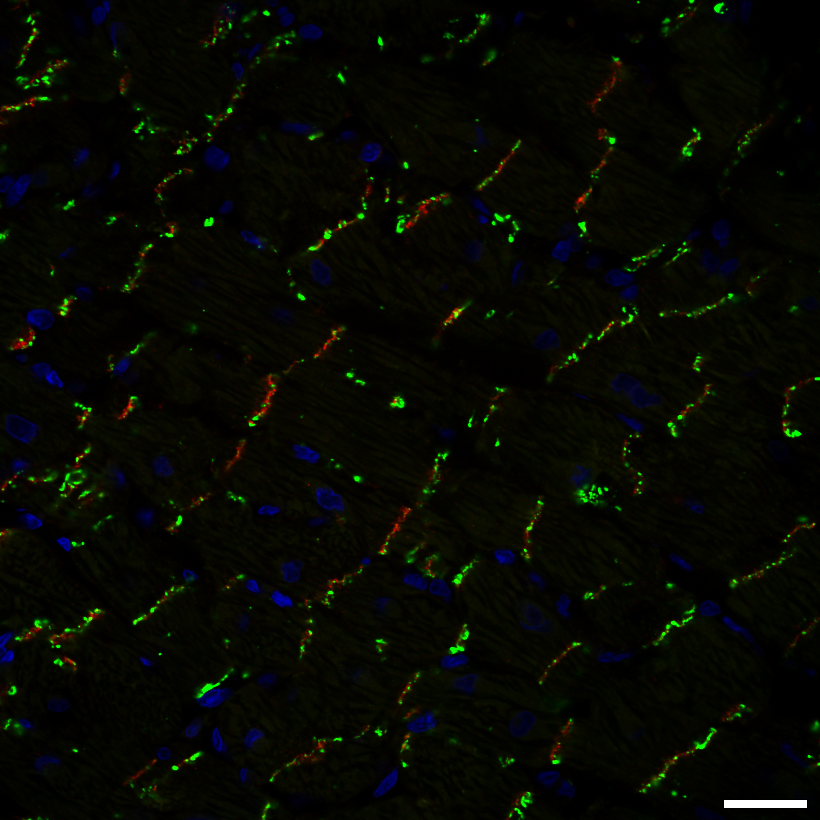

Supplement: Supplemental Information 15 [file peerj-13-19276-s015.zip › immunofluorescence C(Cx43-N-cadherin)/C8-1.tif]

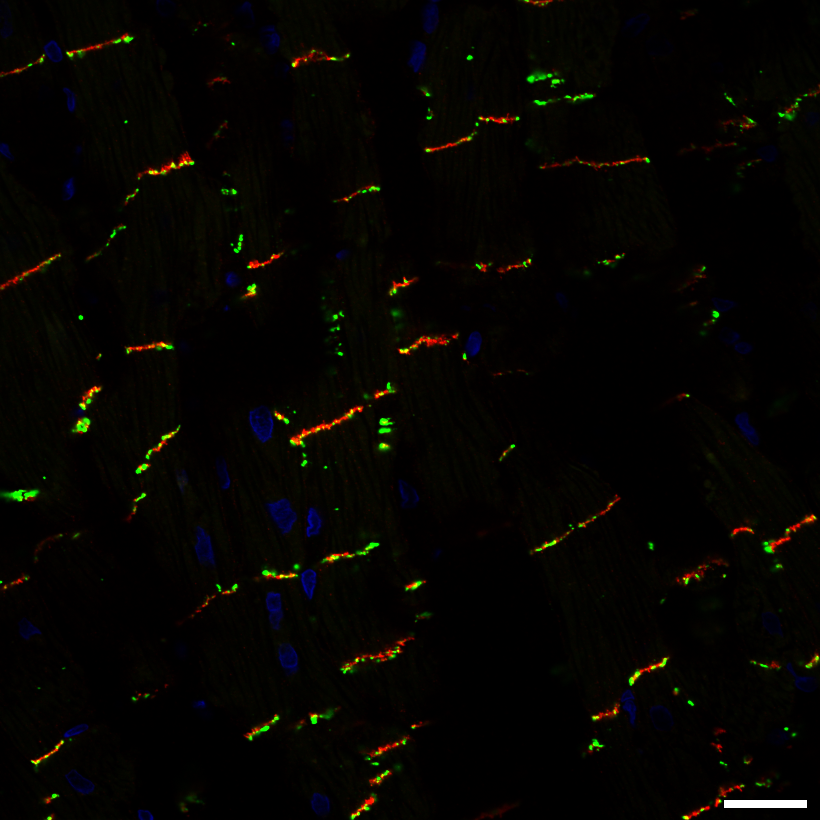

Supplement: Supplemental Information 15 [file peerj-13-19276-s015.zip › immunofluorescence C(Cx43-N-cadherin)/C8-2.tif]

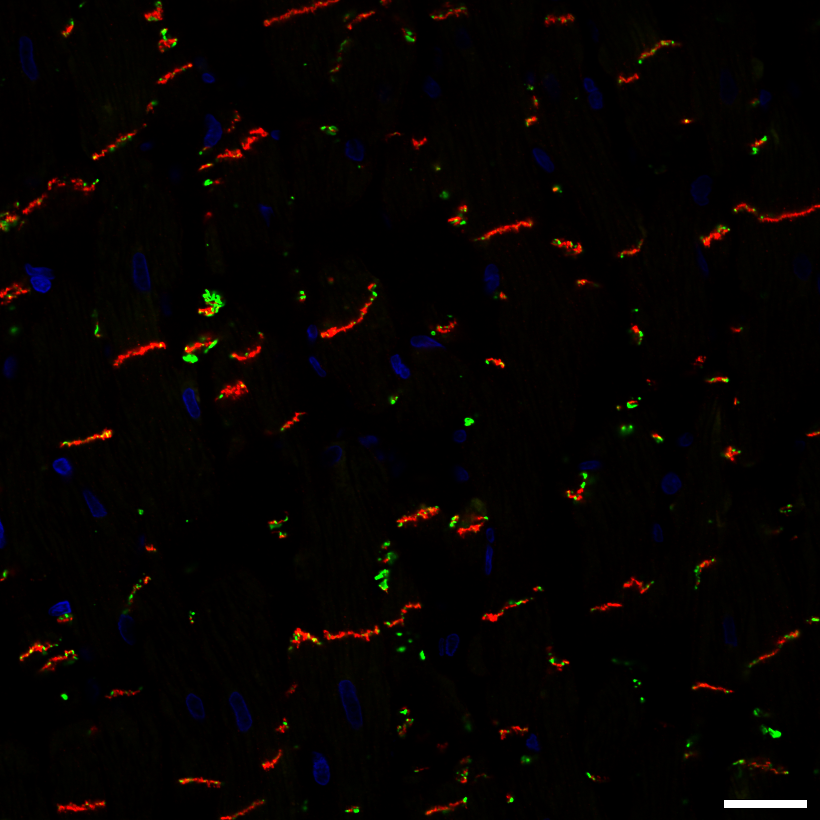

Supplement: Supplemental Information 16 [file peerj-13-19276-s016.zip › immunofluorescence I/R(Cx43-N-cadherin)/I/R1-1.tif]

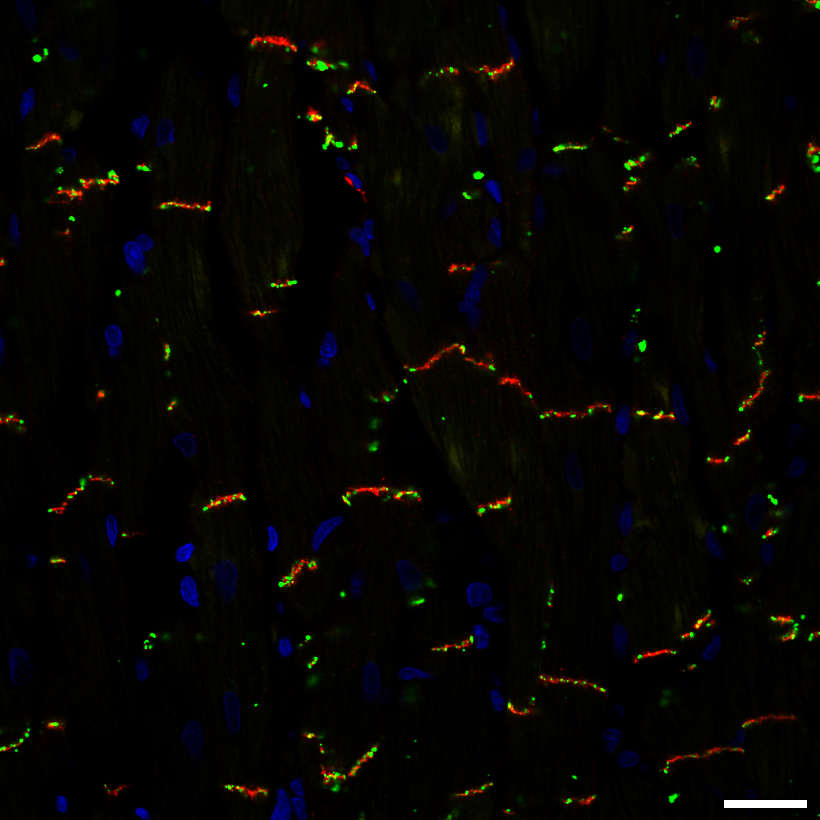

Supplement: Supplemental Information 16 [file peerj-13-19276-s016.zip › immunofluorescence I/R(Cx43-N-cadherin)/I/R1-2.tif]

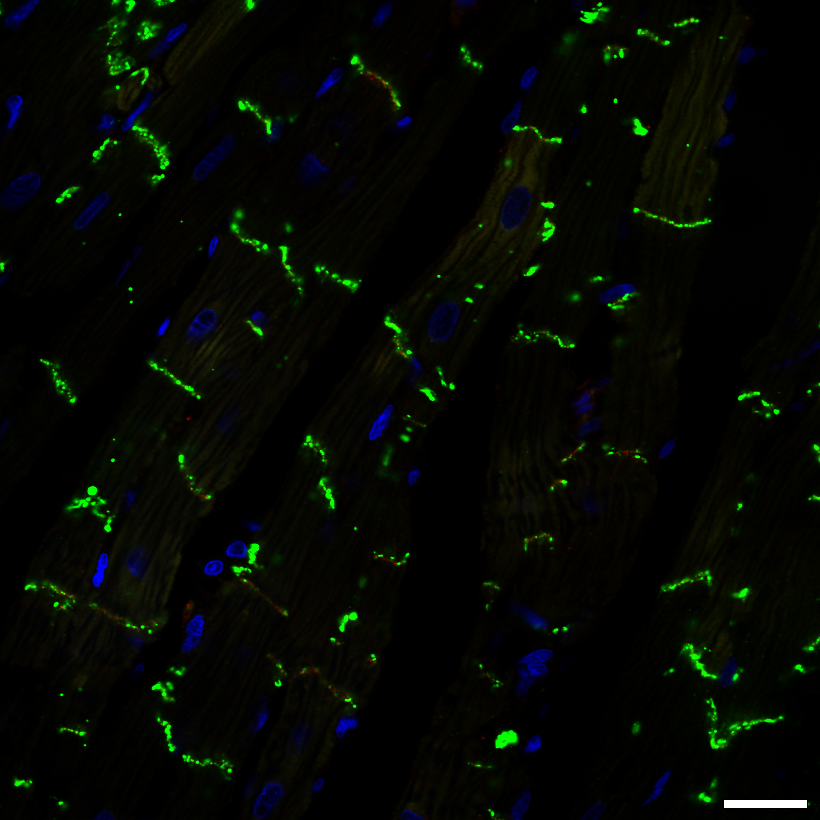

Supplement: Supplemental Information 16 [file peerj-13-19276-s016.zip › immunofluorescence I/R(Cx43-N-cadherin)/I/R2-1.tif]

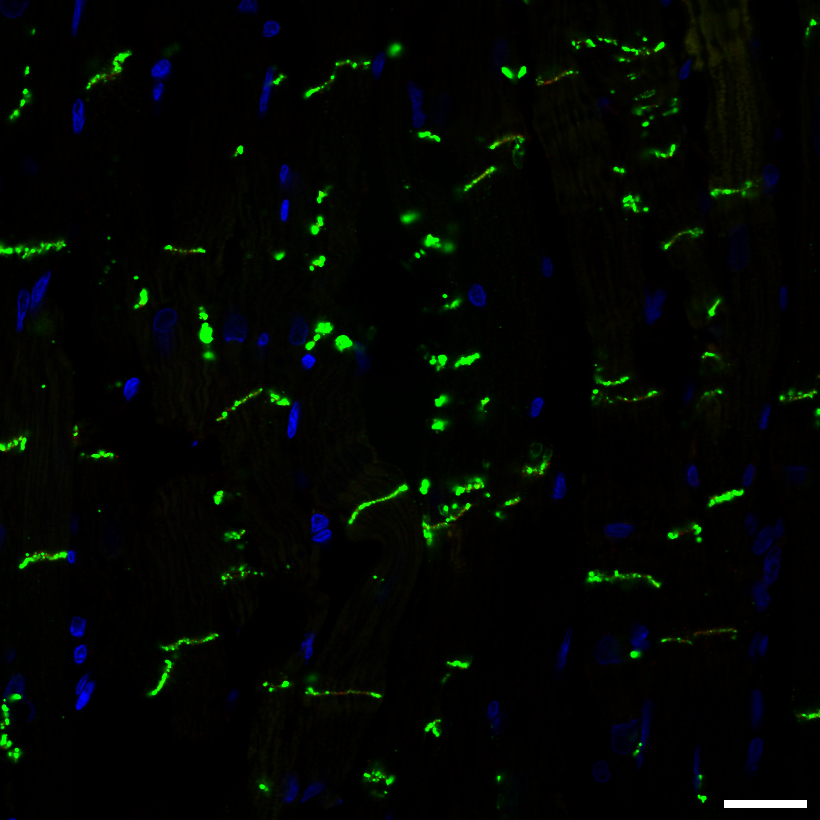

Supplement: Supplemental Information 16 [file peerj-13-19276-s016.zip › immunofluorescence I/R(Cx43-N-cadherin)/I/R2-2.tif]

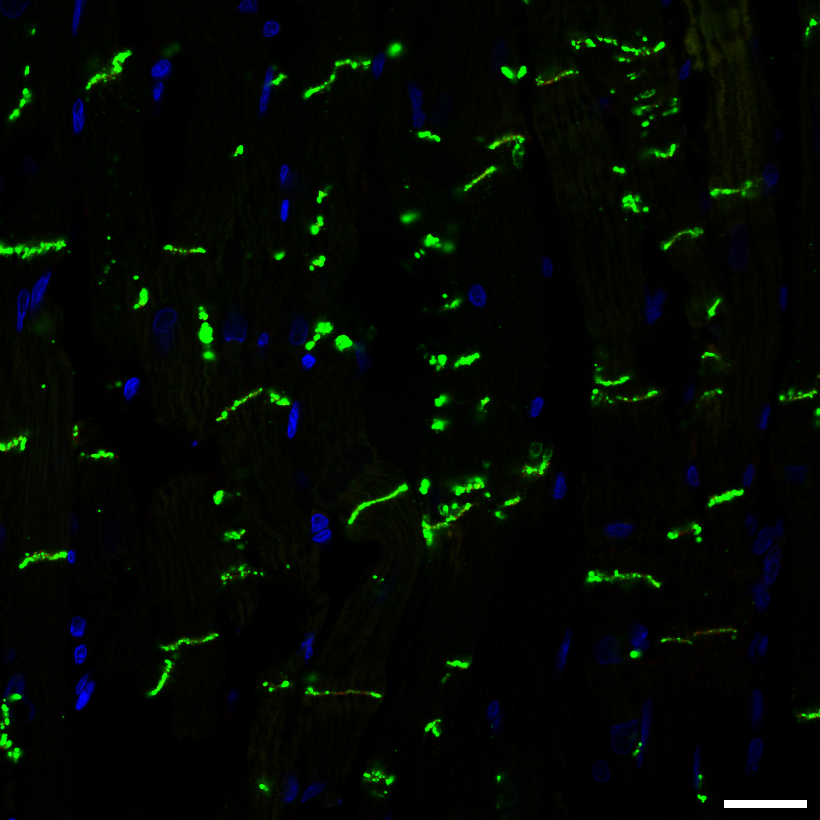

Supplement: Supplemental Information 16 [file peerj-13-19276-s016.zip › immunofluorescence I/R(Cx43-N-cadherin)/I/R2-3.tif]

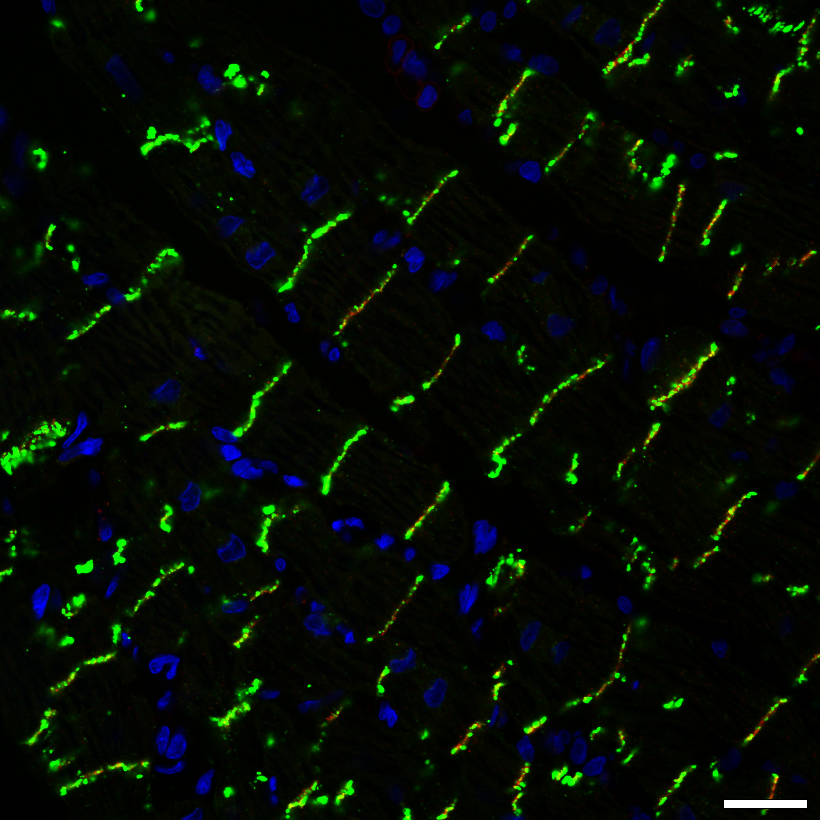

Supplement: Supplemental Information 16 [file peerj-13-19276-s016.zip › immunofluorescence I/R(Cx43-N-cadherin)/I/R3-1.tif]

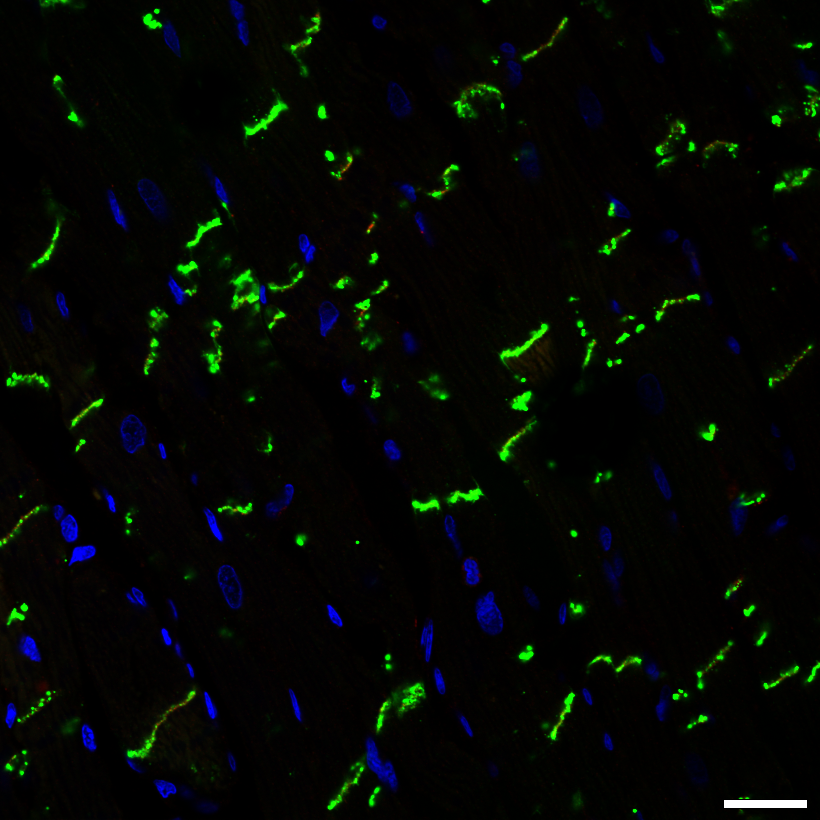

Supplement: Supplemental Information 16 [file peerj-13-19276-s016.zip › immunofluorescence I/R(Cx43-N-cadherin)/I/R3-2.tif]

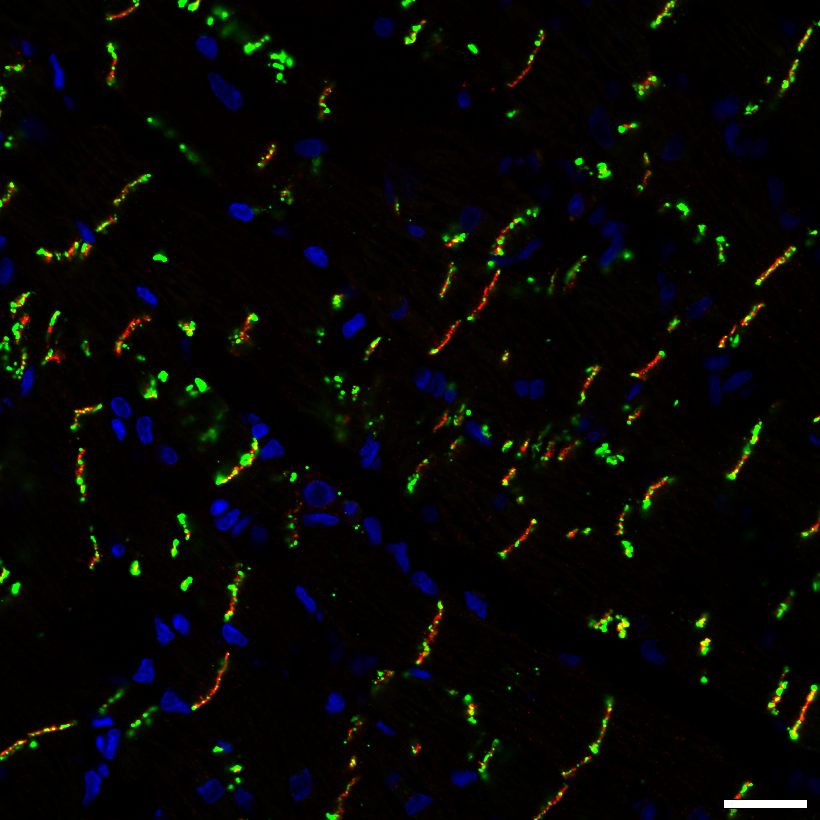

Supplement: Supplemental Information 16 [file peerj-13-19276-s016.zip › immunofluorescence I/R(Cx43-N-cadherin)/I/R4-1.tif]

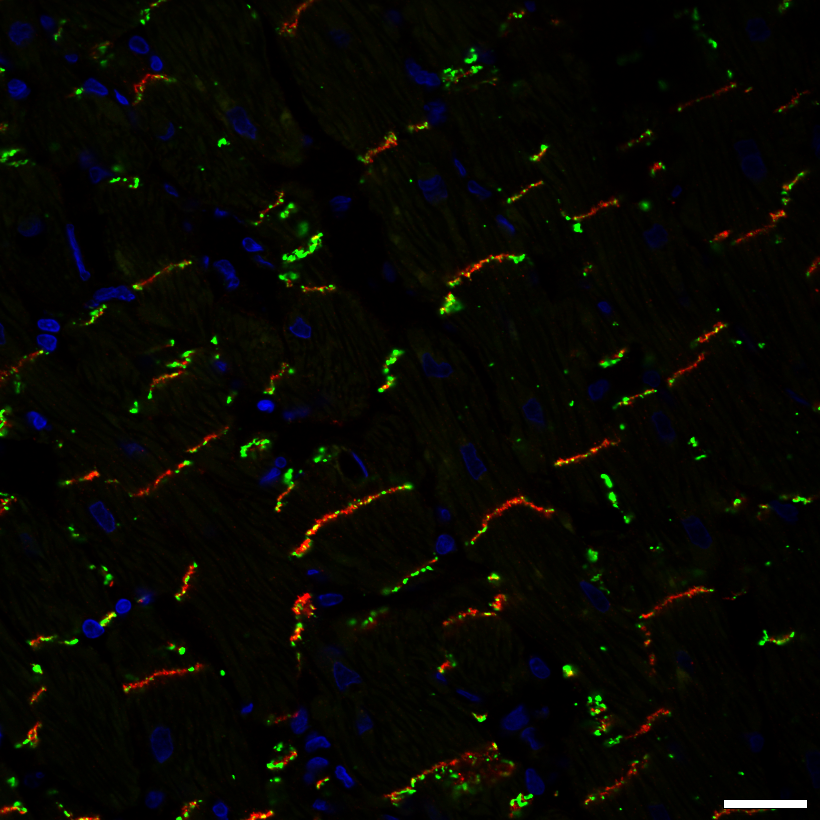

Supplement: Supplemental Information 16 [file peerj-13-19276-s016.zip › immunofluorescence I/R(Cx43-N-cadherin)/I/R4-2.tif]

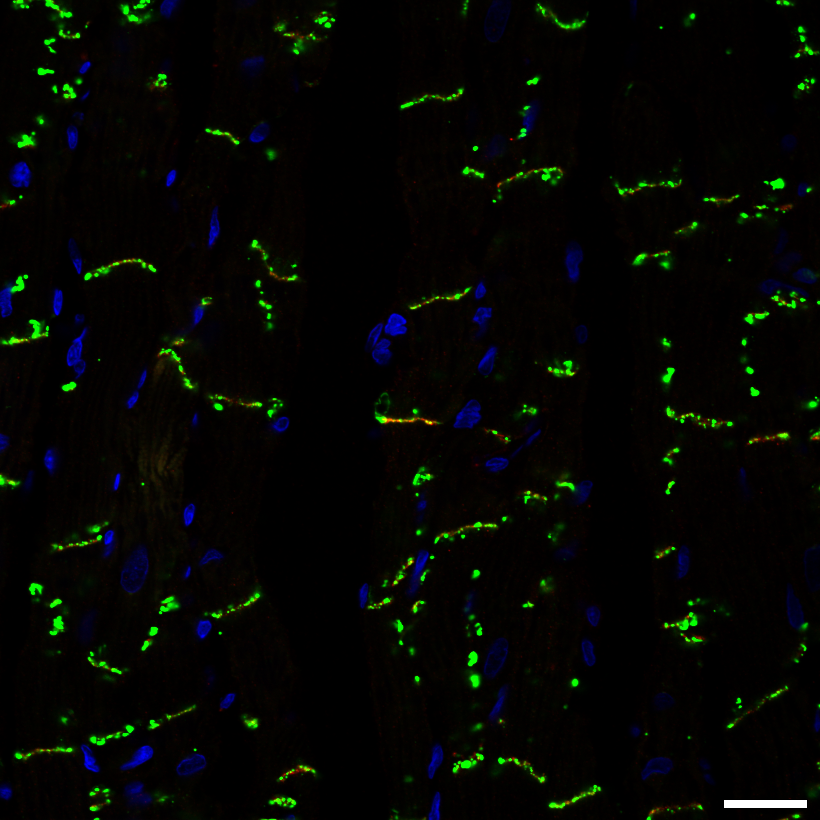

Supplement: Supplemental Information 16 [file peerj-13-19276-s016.zip › immunofluorescence I/R(Cx43-N-cadherin)/I/R5-1.tif]

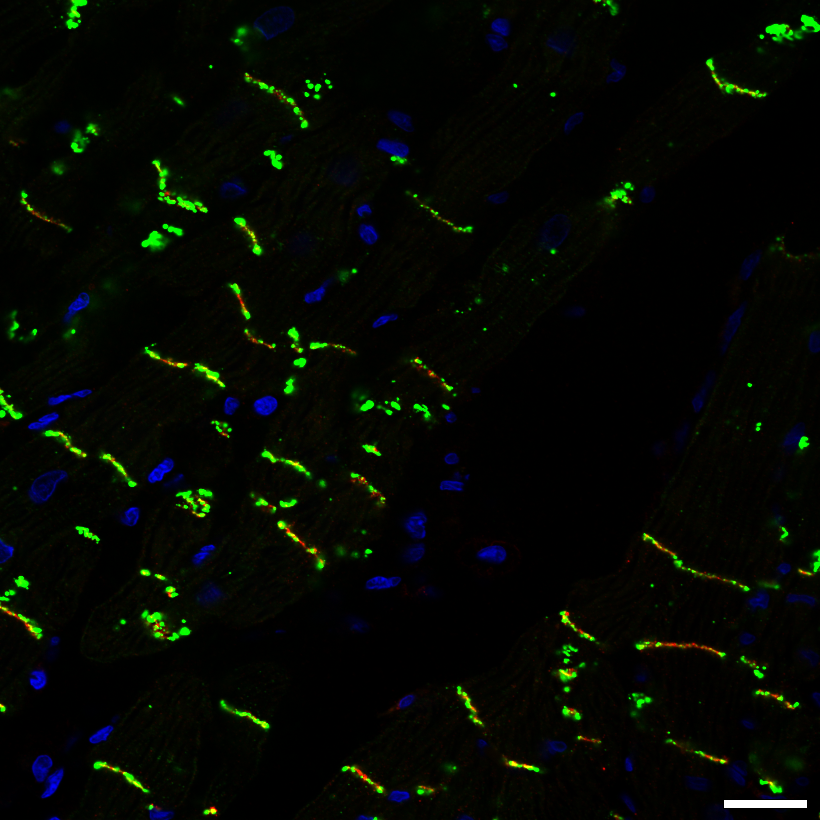

Supplement: Supplemental Information 16 [file peerj-13-19276-s016.zip › immunofluorescence I/R(Cx43-N-cadherin)/I/R5-2.tif]

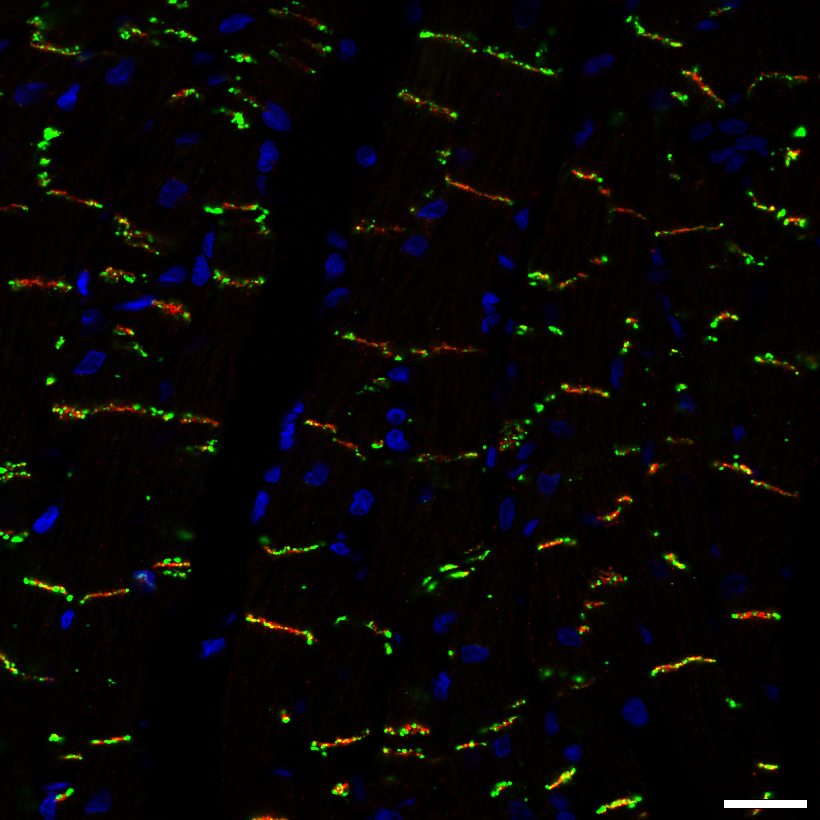

Supplement: Supplemental Information 16 [file peerj-13-19276-s016.zip › immunofluorescence I/R(Cx43-N-cadherin)/I/R6-1.tif]

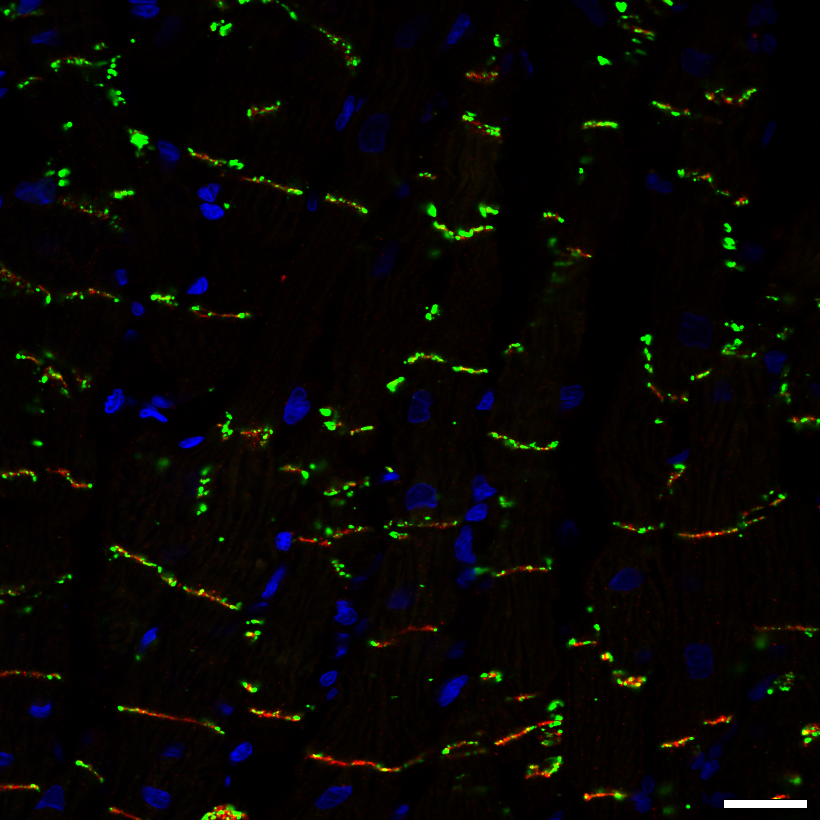

Supplement: Supplemental Information 16 [file peerj-13-19276-s016.zip › immunofluorescence I/R(Cx43-N-cadherin)/I/R6-2.tif]

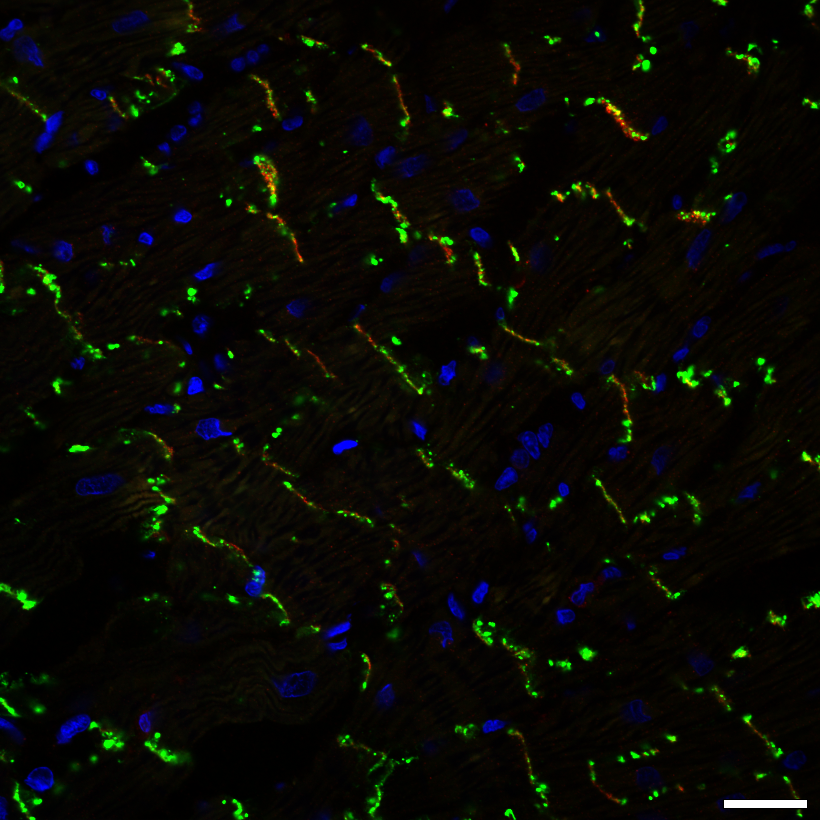

Supplement: Supplemental Information 16 [file peerj-13-19276-s016.zip › immunofluorescence I/R(Cx43-N-cadherin)/I/R6-3.tif]

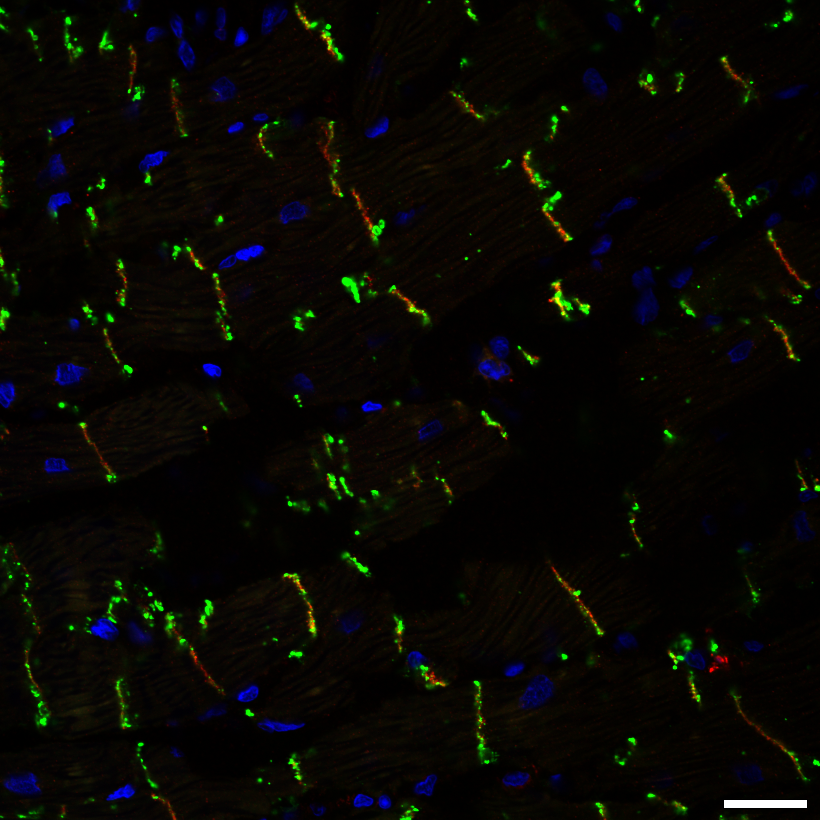

Supplement: Supplemental Information 16 [file peerj-13-19276-s016.zip › immunofluorescence I/R(Cx43-N-cadherin)/I/R7-1.tif]
