# Supplementary figures and images for: Downregulation of EB1 impedes Cx43 localization and cardiac conduction after hypothermic ischemia-reperfusion in rats (part 5 of 5)
Source: PeerJ. 2025 Apr 14;13:e19276. doi: 10.7717/peerj.19276 (PMC12005192; doi:10.7717/peerj.19276)

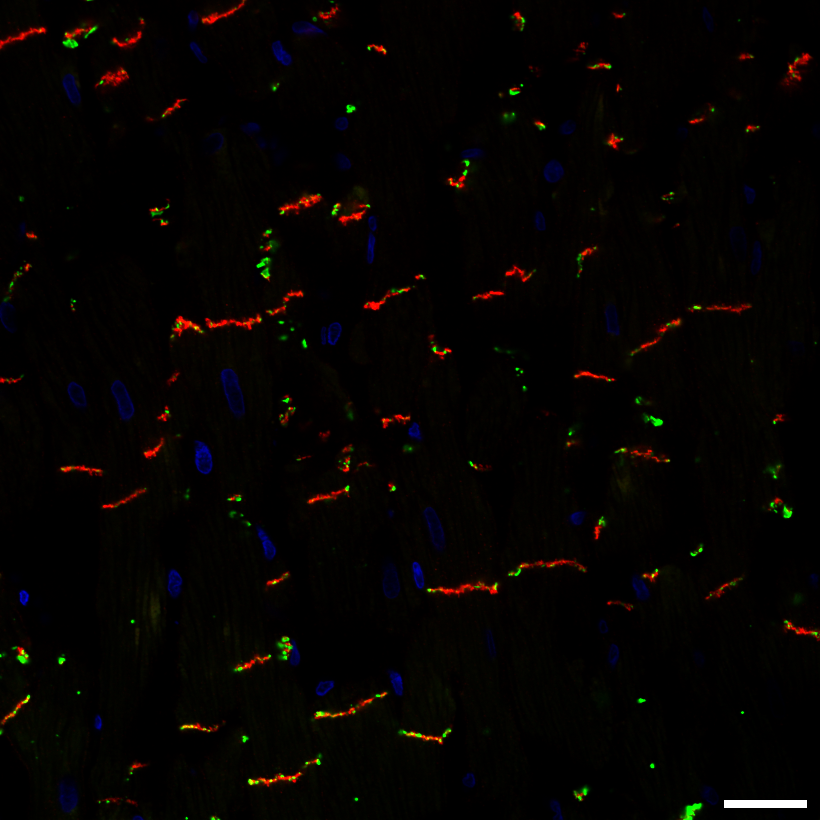

Supplement: Supplemental Information 16 [file peerj-13-19276-s016.zip › immunofluorescence I/R(Cx43-N-cadherin)/I/R7-2.tif]

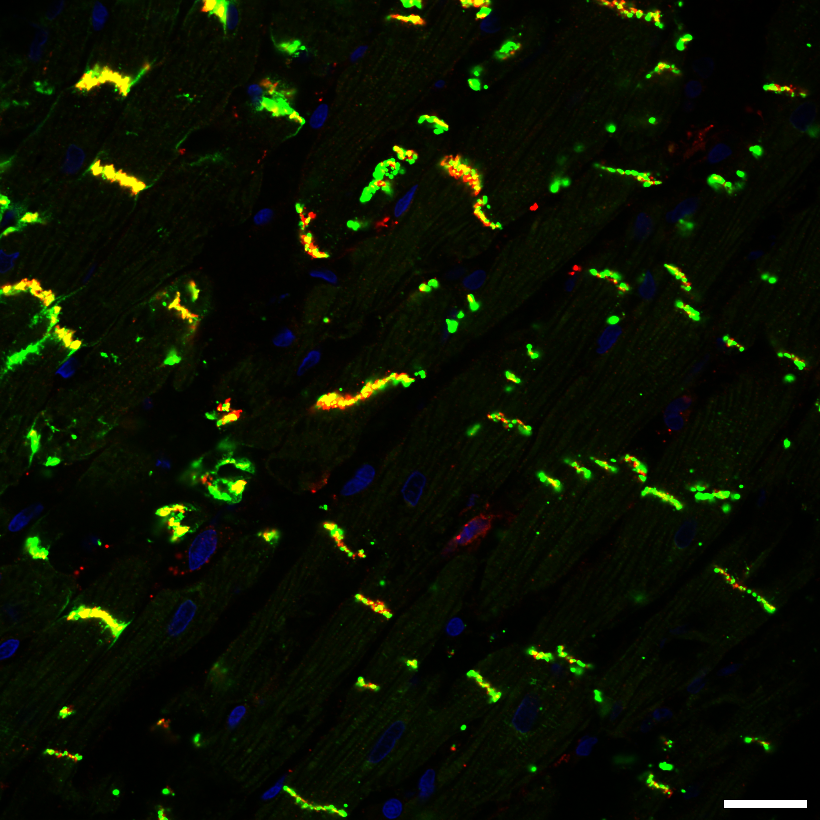

Supplement: Supplemental Information 16 [file peerj-13-19276-s016.zip › immunofluorescence I/R(Cx43-N-cadherin)/I/R8.tif]

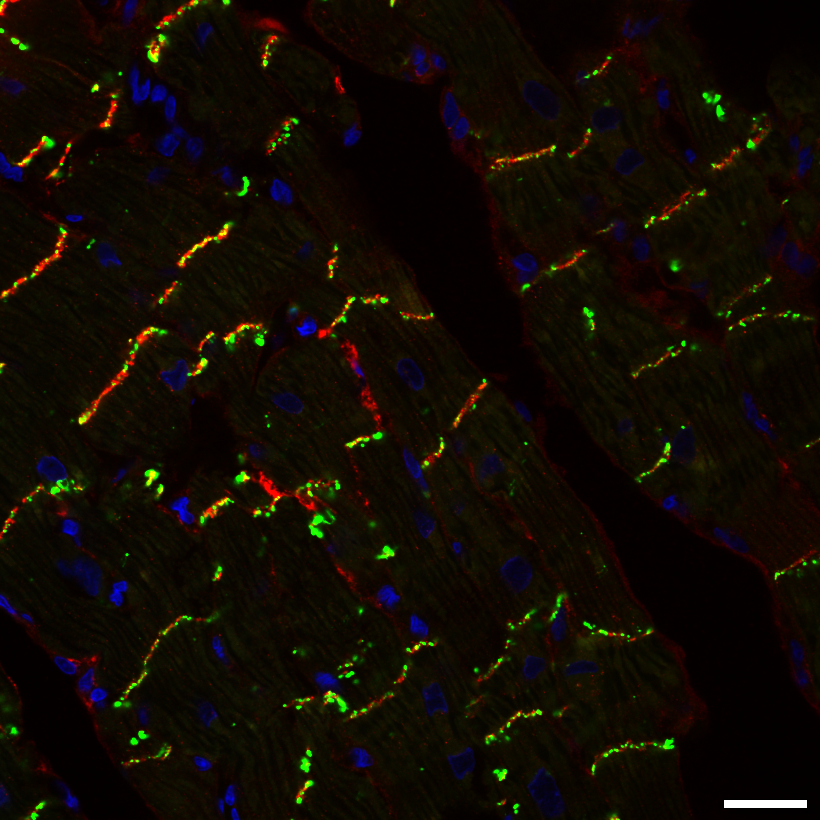

Supplement: Supplemental Information 17 [file peerj-13-19276-s017.zip › immunofluorescence AAV9-CON(Cx43-N-cadherin)/AAV9-CON1-1.tif]

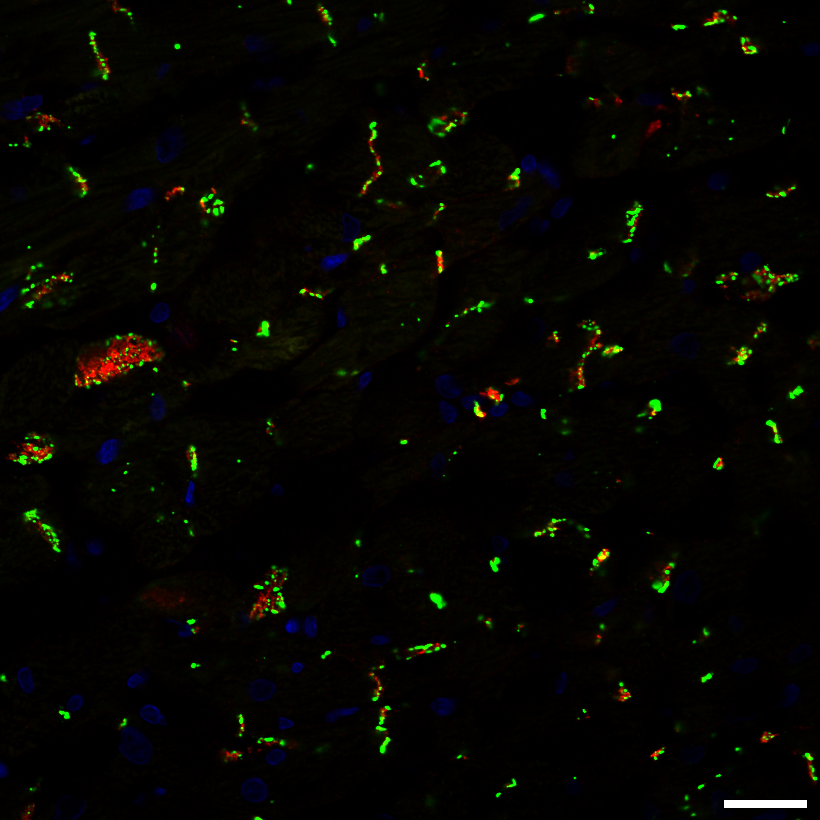

Supplement: Supplemental Information 17 [file peerj-13-19276-s017.zip › immunofluorescence AAV9-CON(Cx43-N-cadherin)/AAV9-CON1-2.tif]

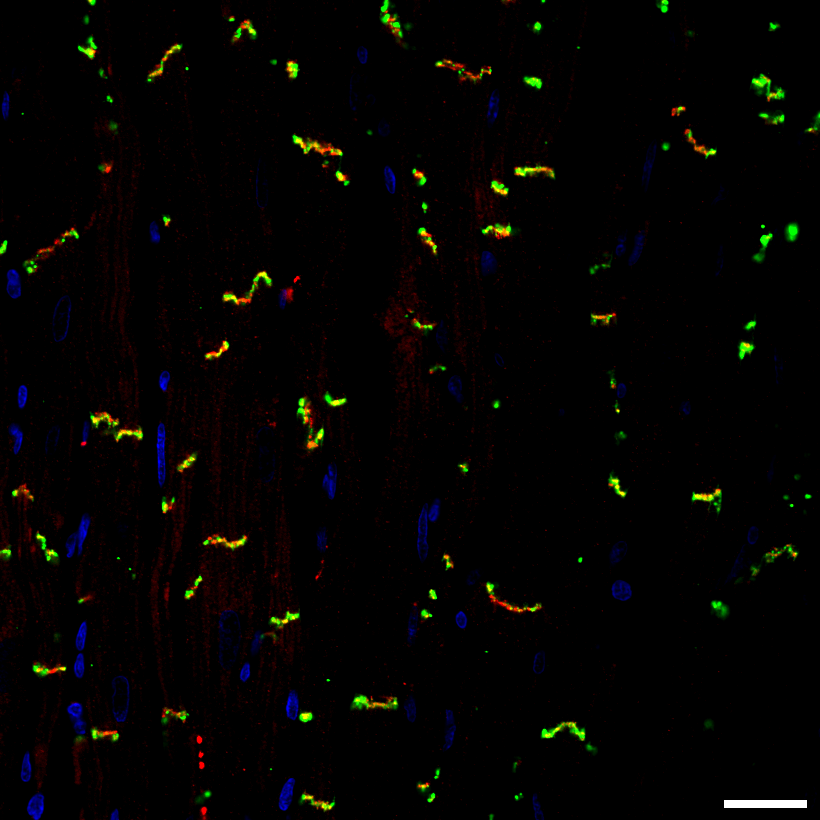

Supplement: Supplemental Information 17 [file peerj-13-19276-s017.zip › immunofluorescence AAV9-CON(Cx43-N-cadherin)/AAV9-CON2-1.tif]

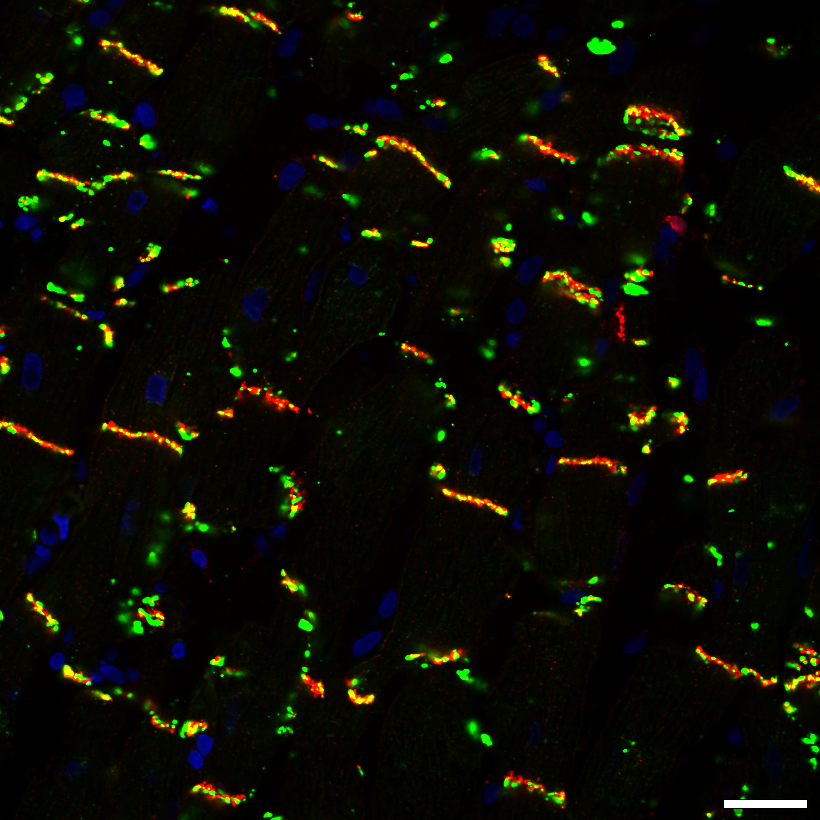

Supplement: Supplemental Information 17 [file peerj-13-19276-s017.zip › immunofluorescence AAV9-CON(Cx43-N-cadherin)/AAV9-CON2-2.tif]

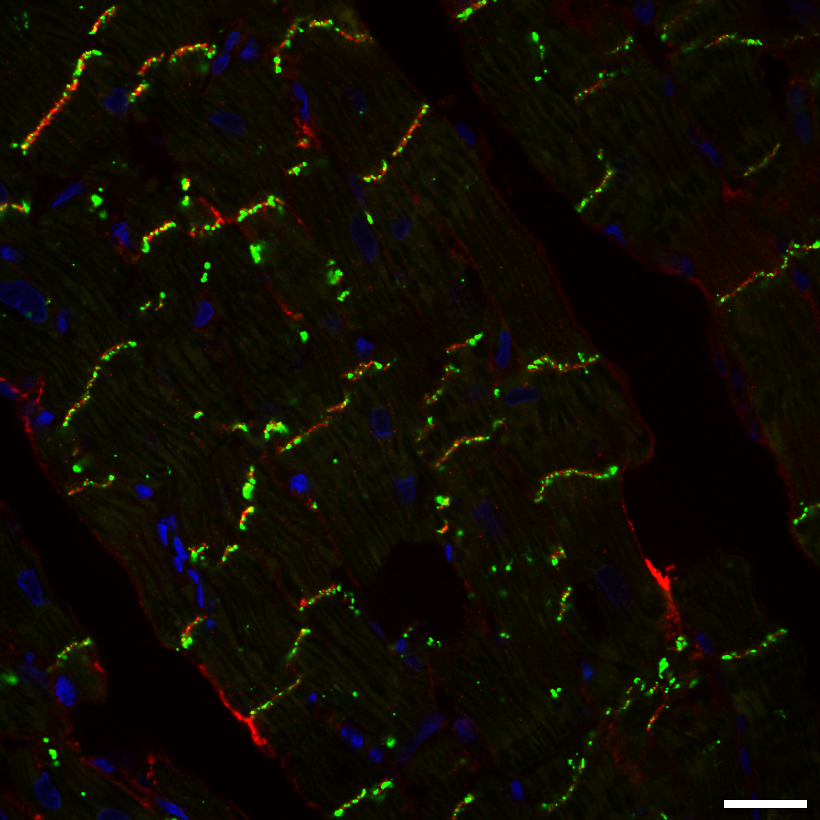

Supplement: Supplemental Information 17 [file peerj-13-19276-s017.zip › immunofluorescence AAV9-CON(Cx43-N-cadherin)/AAV9-CON3-1.tif]

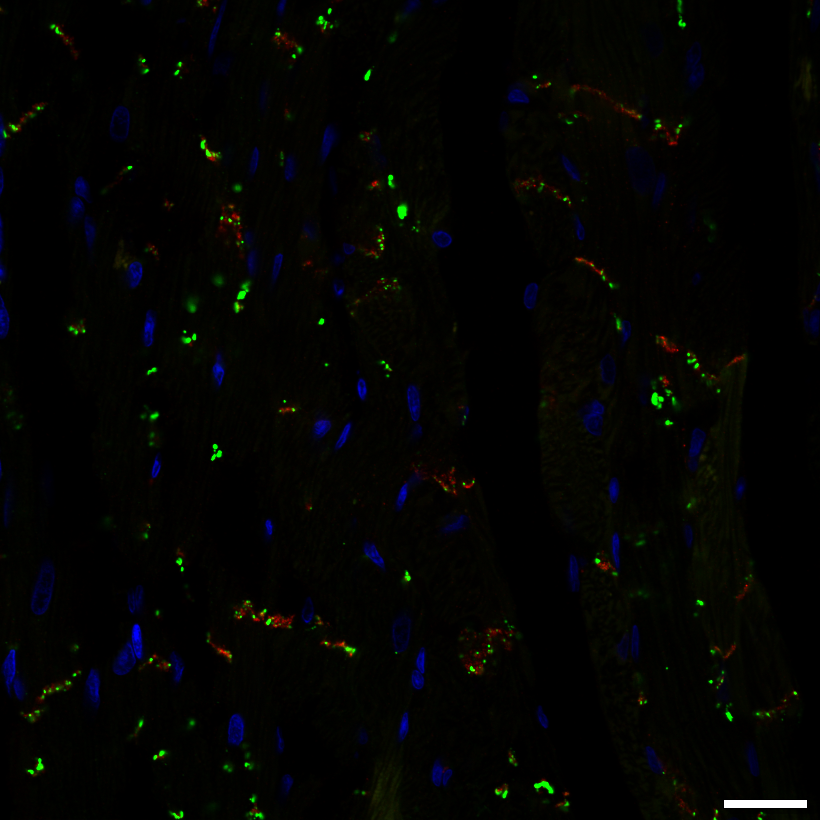

Supplement: Supplemental Information 17 [file peerj-13-19276-s017.zip › immunofluorescence AAV9-CON(Cx43-N-cadherin)/AAV9-CON3-2.tif]

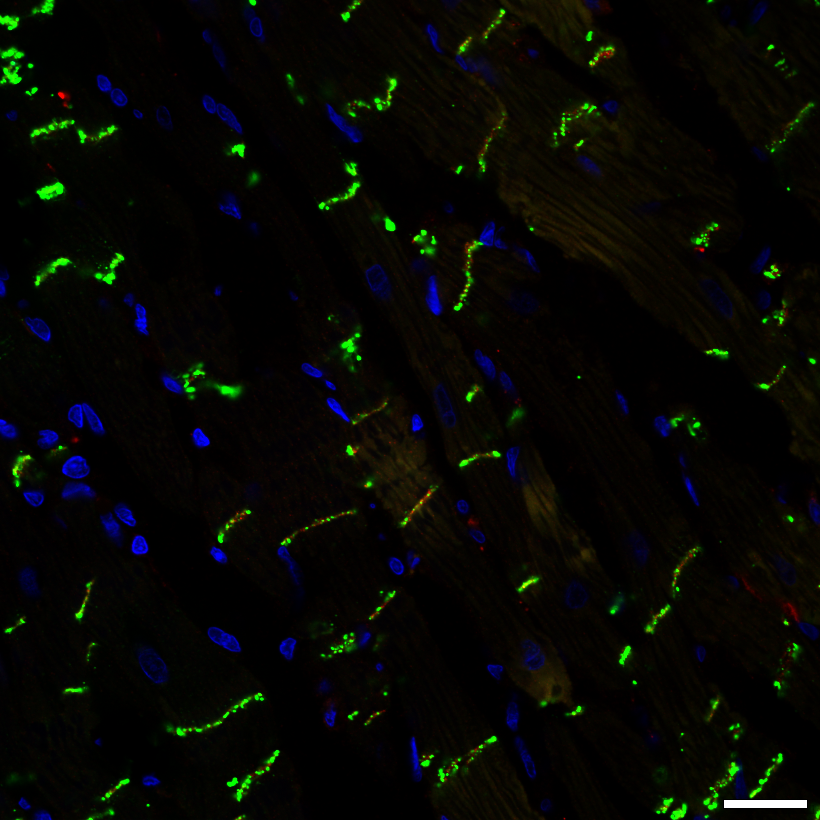

Supplement: Supplemental Information 17 [file peerj-13-19276-s017.zip › immunofluorescence AAV9-CON(Cx43-N-cadherin)/AAV9-CON4-1.tif]

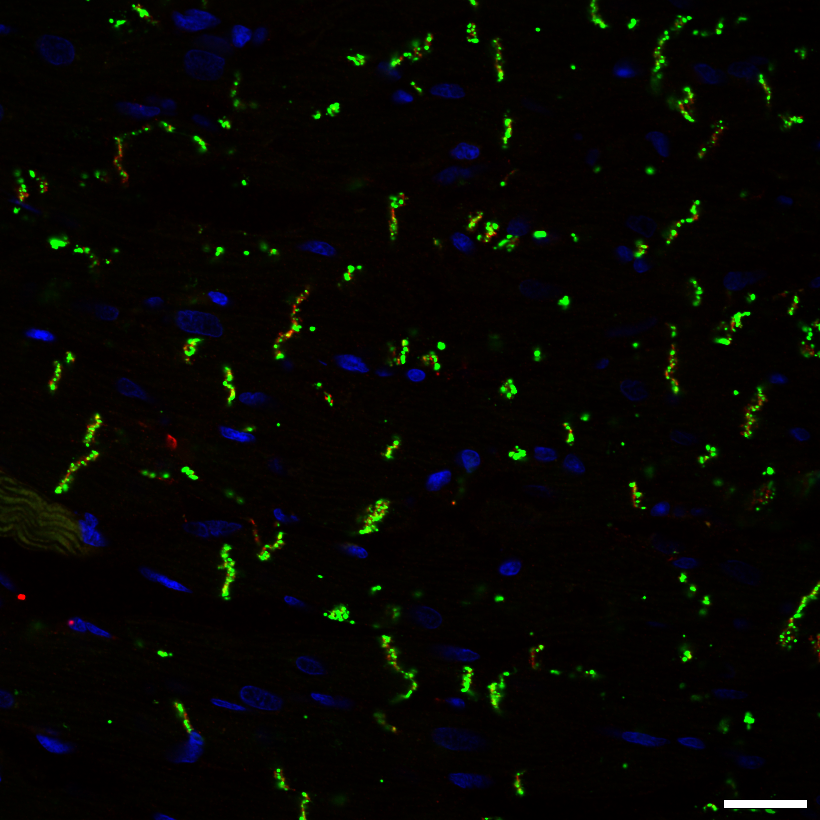

Supplement: Supplemental Information 17 [file peerj-13-19276-s017.zip › immunofluorescence AAV9-CON(Cx43-N-cadherin)/AAV9-CON4-2.tif]

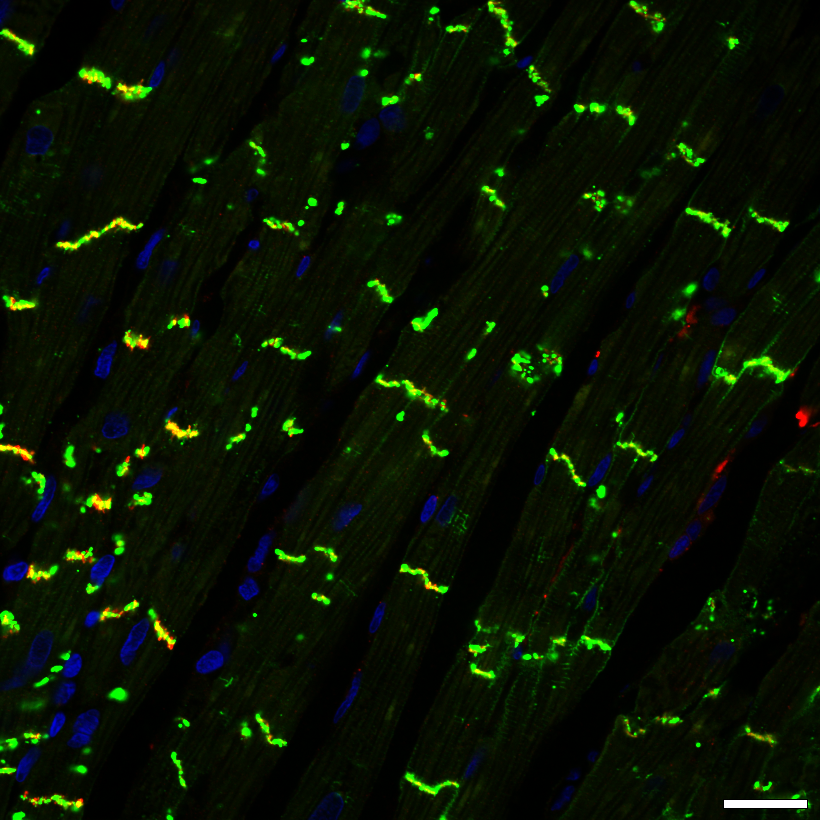

Supplement: Supplemental Information 17 [file peerj-13-19276-s017.zip › immunofluorescence AAV9-CON(Cx43-N-cadherin)/AAV9-CON5-1.tif]

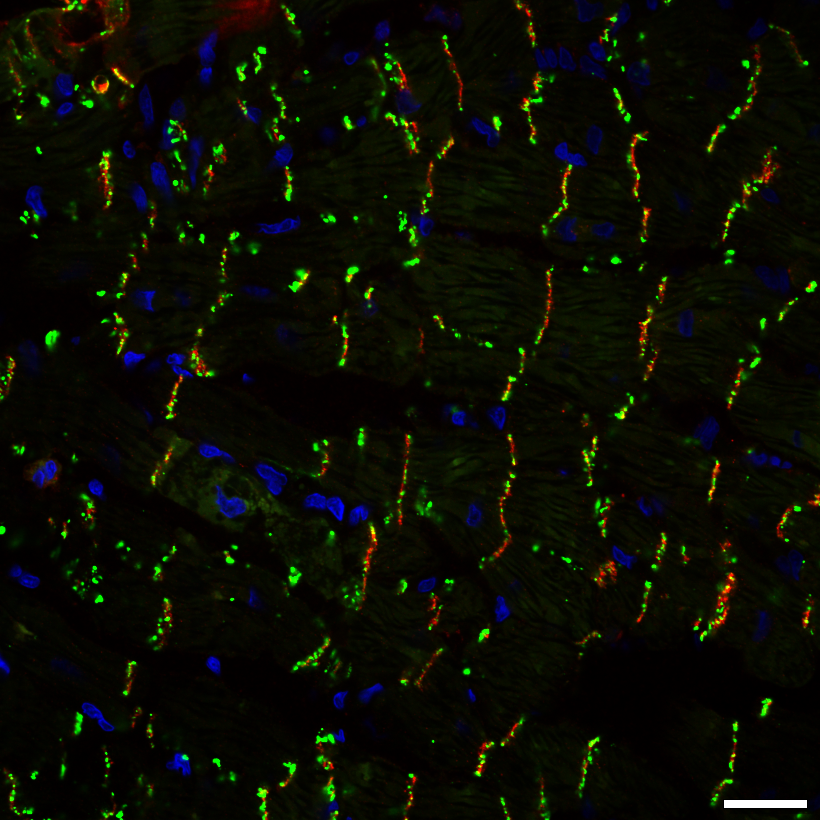

Supplement: Supplemental Information 17 [file peerj-13-19276-s017.zip › immunofluorescence AAV9-CON(Cx43-N-cadherin)/AAV9-CON5-2.tif]

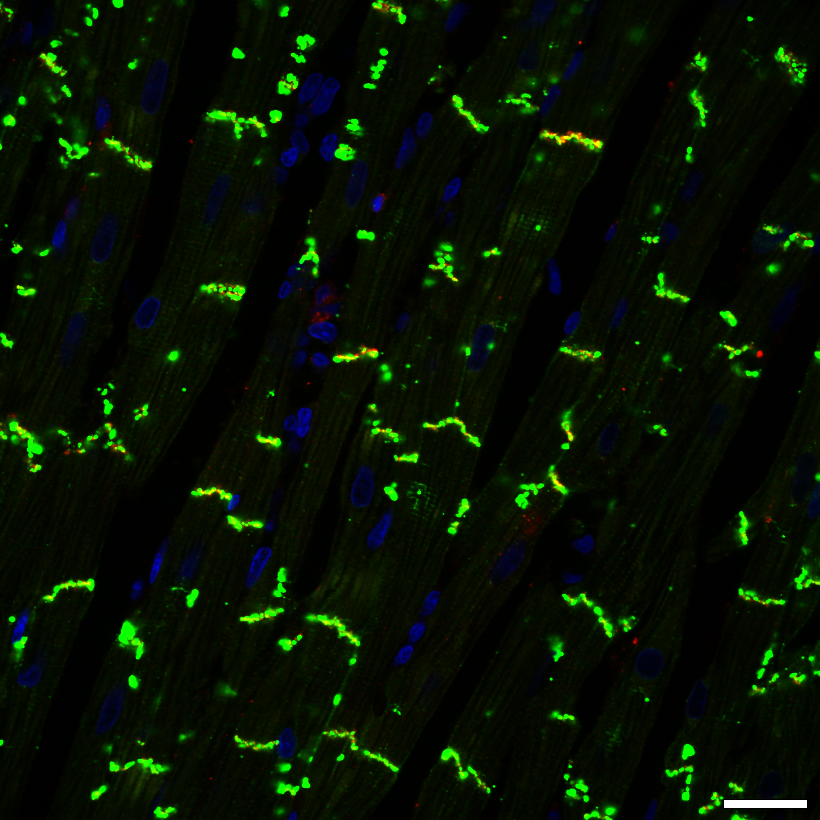

Supplement: Supplemental Information 17 [file peerj-13-19276-s017.zip › immunofluorescence AAV9-CON(Cx43-N-cadherin)/AAV9-CON5-3.tif]

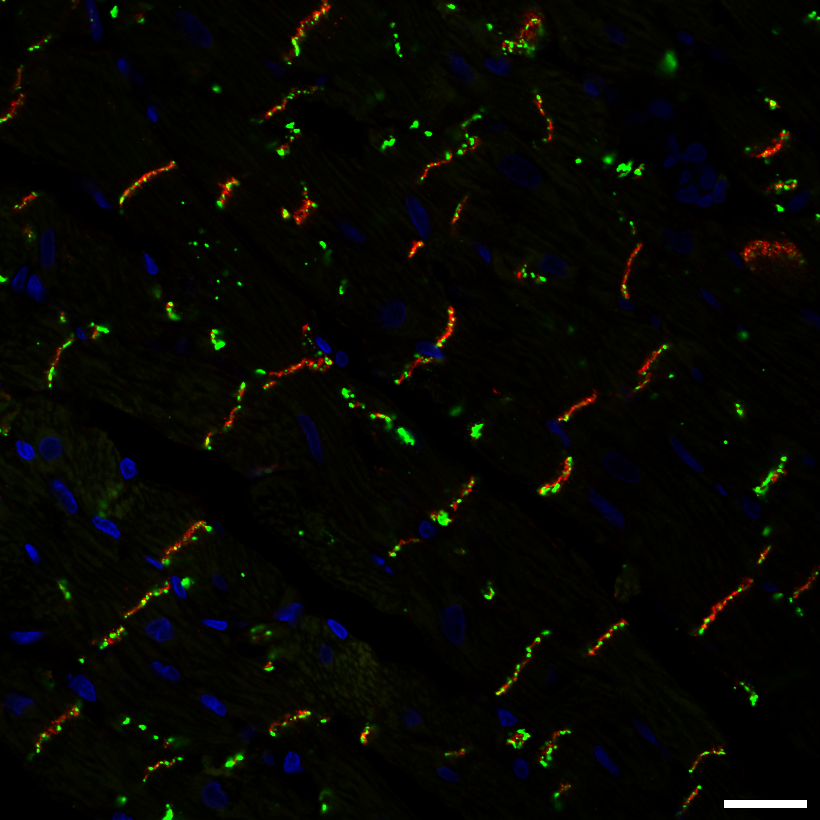

Supplement: Supplemental Information 17 [file peerj-13-19276-s017.zip › immunofluorescence AAV9-CON(Cx43-N-cadherin)/AAV9-CON6-1.tif]

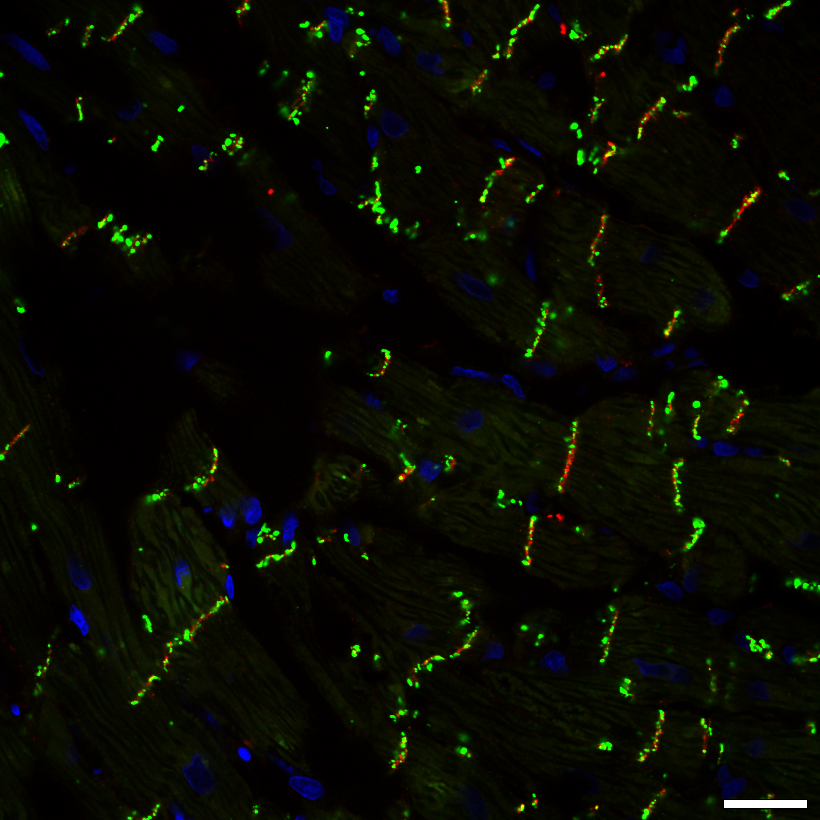

Supplement: Supplemental Information 17 [file peerj-13-19276-s017.zip › immunofluorescence AAV9-CON(Cx43-N-cadherin)/AAV9-CON6-2.tif]

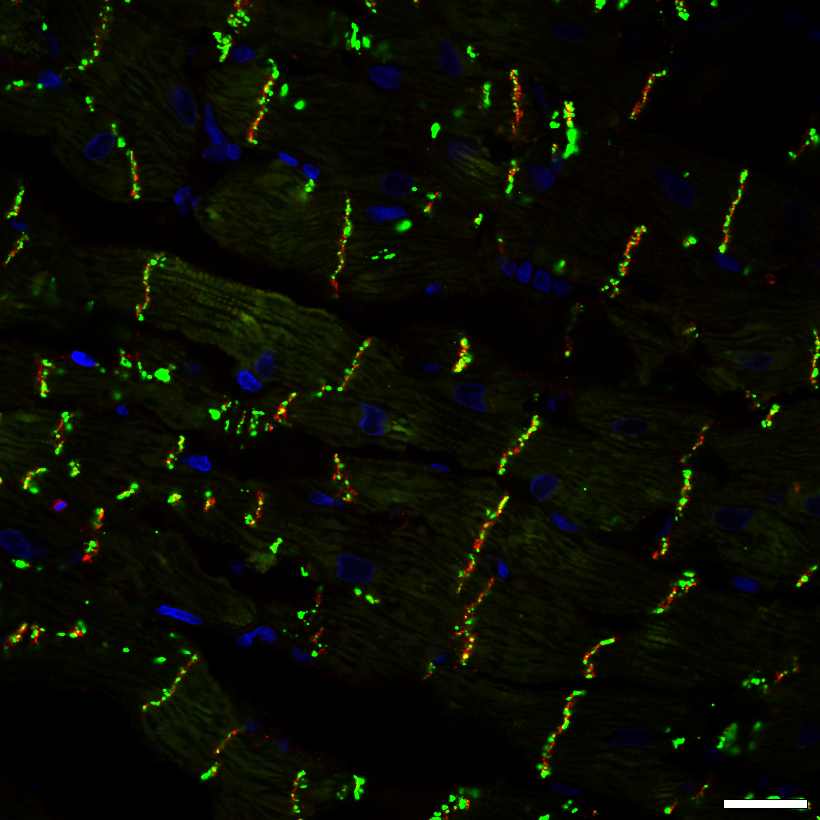

Supplement: Supplemental Information 17 [file peerj-13-19276-s017.zip › immunofluorescence AAV9-CON(Cx43-N-cadherin)/AAV9-CON6-3.tif]

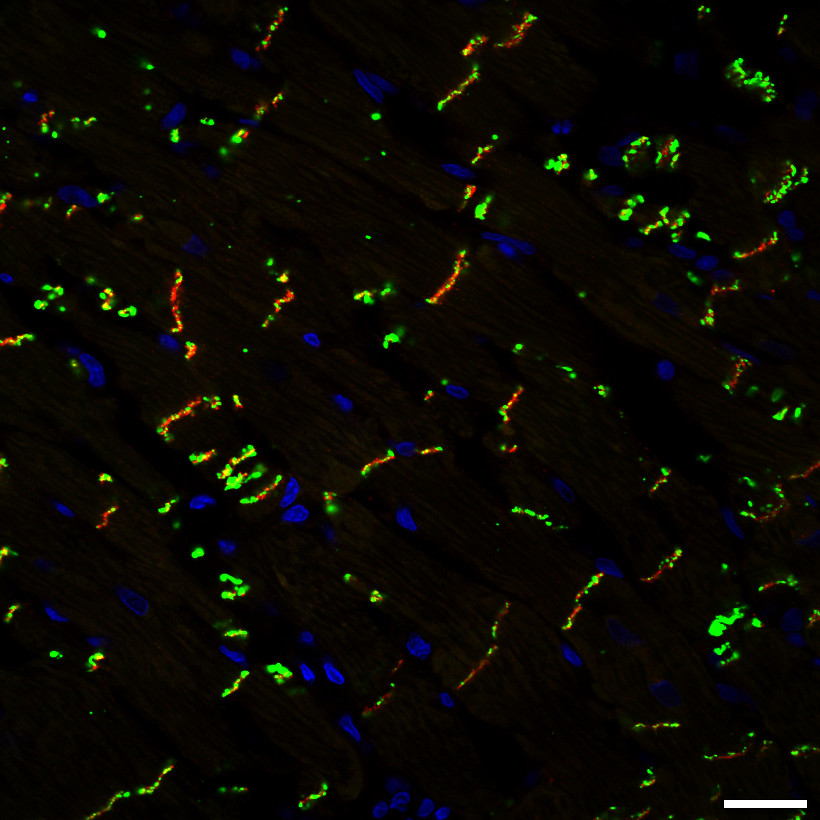

Supplement: Supplemental Information 17 [file peerj-13-19276-s017.zip › immunofluorescence AAV9-CON(Cx43-N-cadherin)/AAV9-CON7-1.tif]

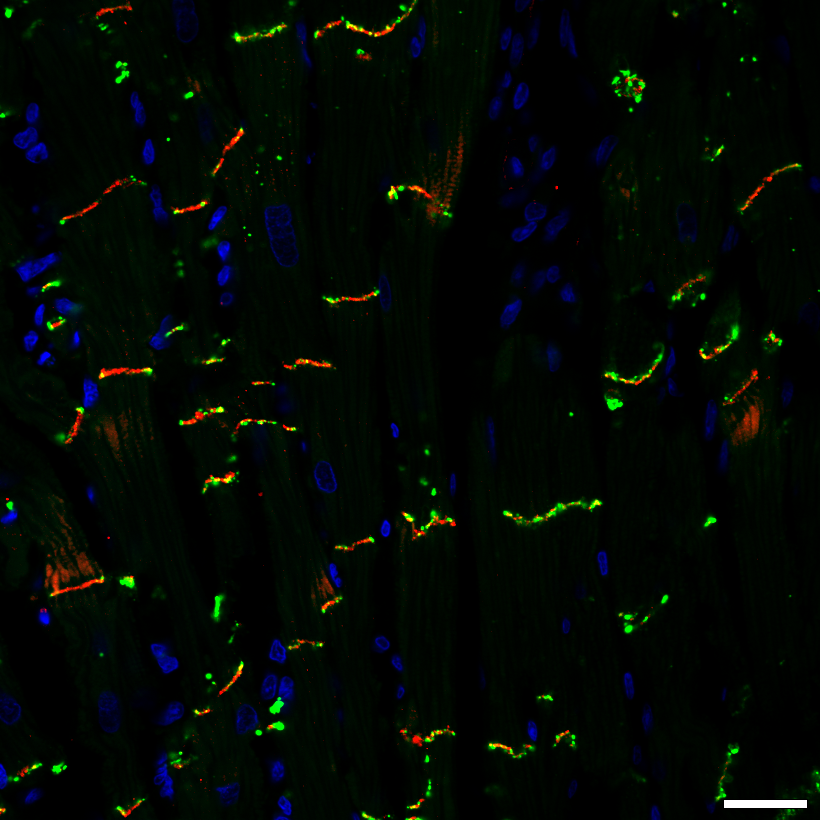

Supplement: Supplemental Information 17 [file peerj-13-19276-s017.zip › immunofluorescence AAV9-CON(Cx43-N-cadherin)/AAV9-CON7-2.tif]

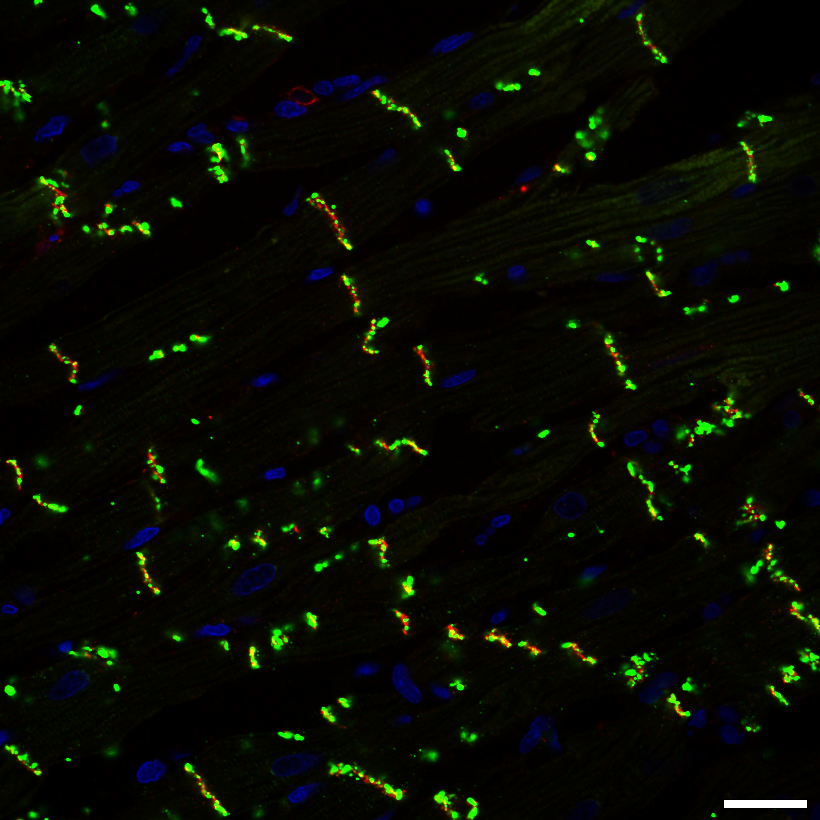

Supplement: Supplemental Information 17 [file peerj-13-19276-s017.zip › immunofluorescence AAV9-CON(Cx43-N-cadherin)/AAV9-CON7-3.tif]

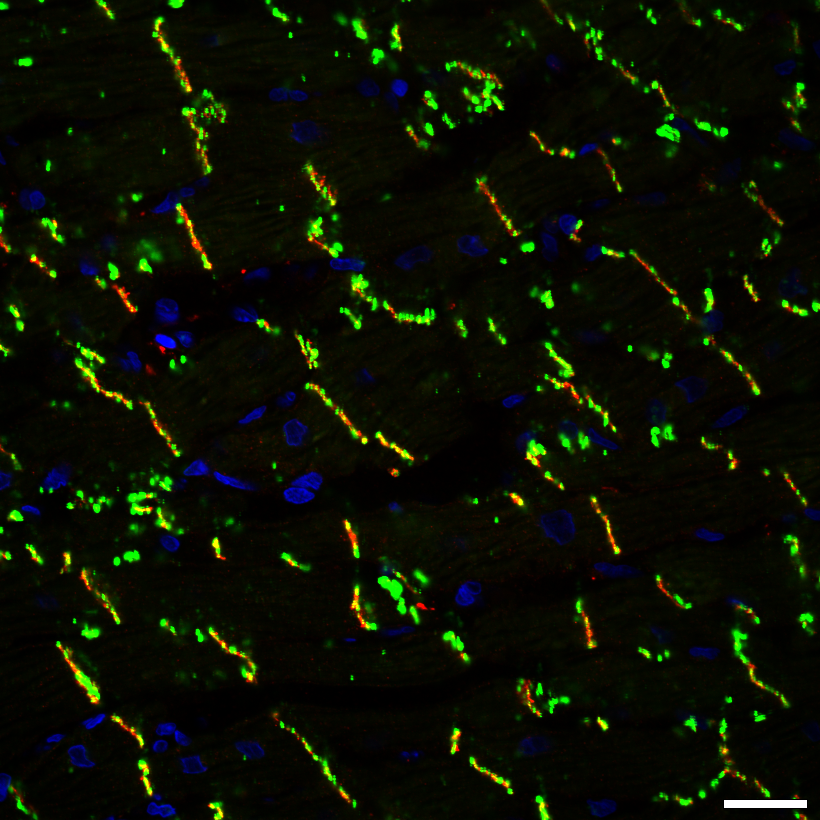

Supplement: Supplemental Information 18 [file peerj-13-19276-s018.zip › immunofluorescence AAV9-EB1(Cx43-N-cadherin)/AAV9-EB1 1-1.tif]

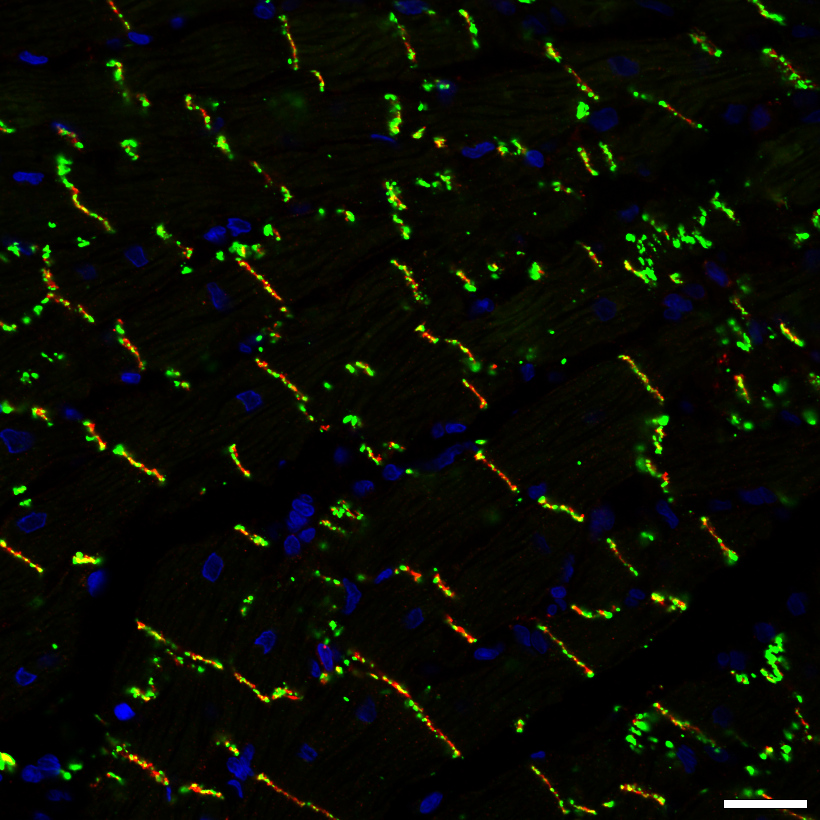

Supplement: Supplemental Information 18 [file peerj-13-19276-s018.zip › immunofluorescence AAV9-EB1(Cx43-N-cadherin)/AAV9-EB1 1-2.tif]

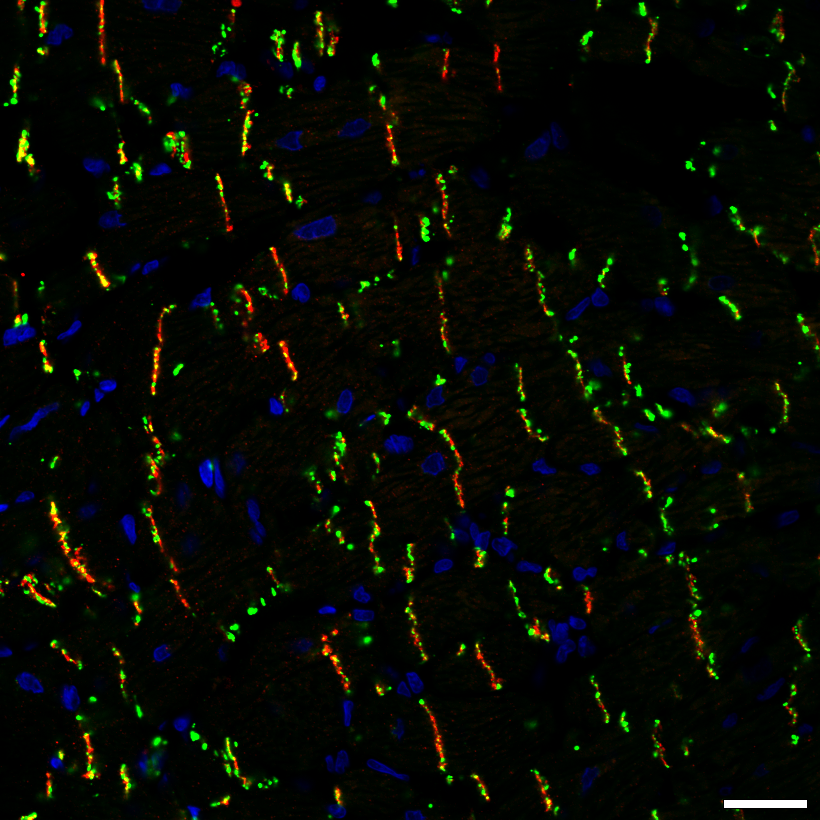

Supplement: Supplemental Information 18 [file peerj-13-19276-s018.zip › immunofluorescence AAV9-EB1(Cx43-N-cadherin)/AAV9-EB1 2-1.tif]

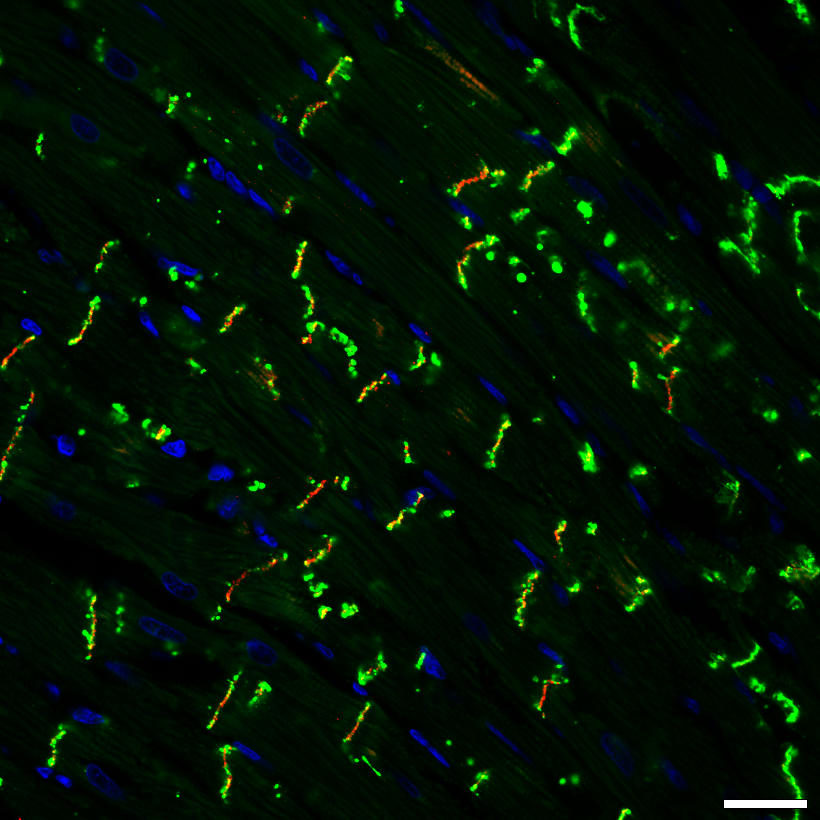

Supplement: Supplemental Information 18 [file peerj-13-19276-s018.zip › immunofluorescence AAV9-EB1(Cx43-N-cadherin)/AAV9-EB1 2-2.tif]

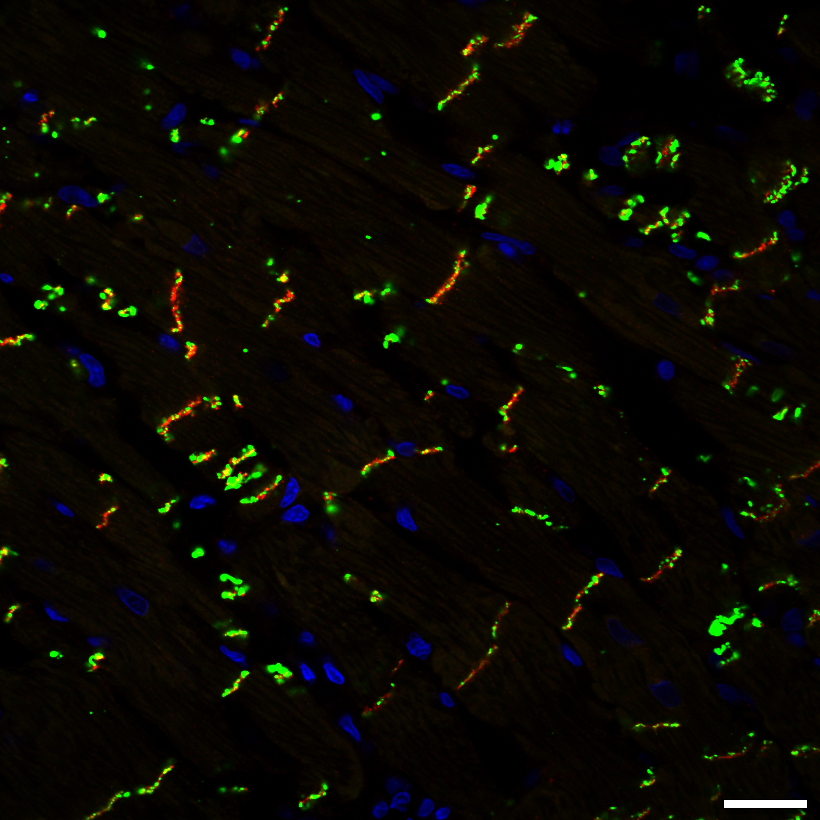

Supplement: Supplemental Information 18 [file peerj-13-19276-s018.zip › immunofluorescence AAV9-EB1(Cx43-N-cadherin)/AAV9-EB1 3-1.tif]

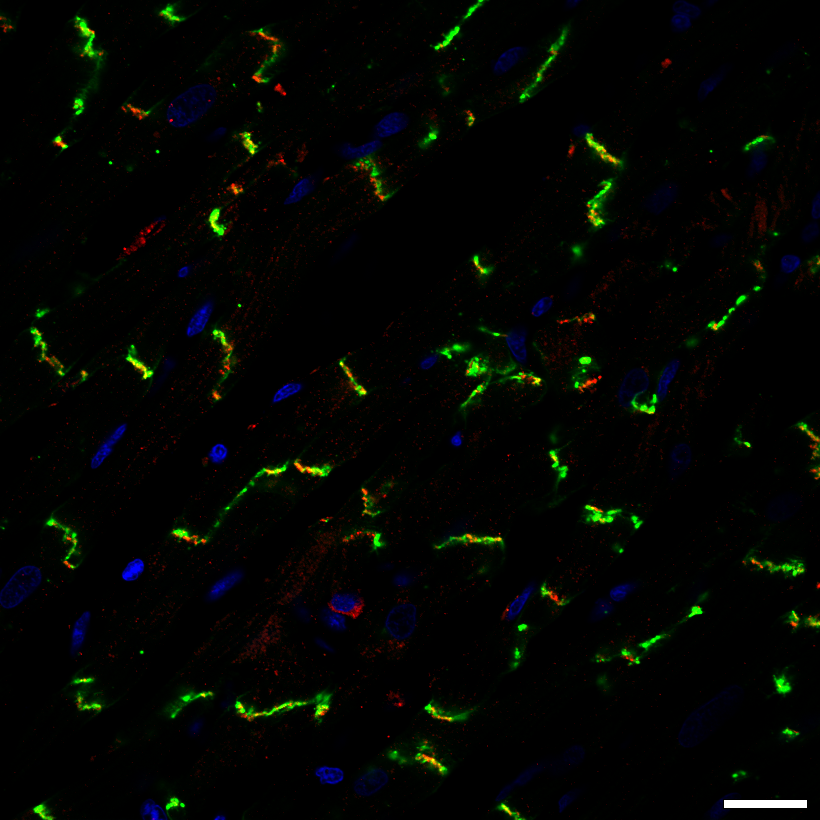

Supplement: Supplemental Information 18 [file peerj-13-19276-s018.zip › immunofluorescence AAV9-EB1(Cx43-N-cadherin)/AAV9-EB1 3-2.tif]

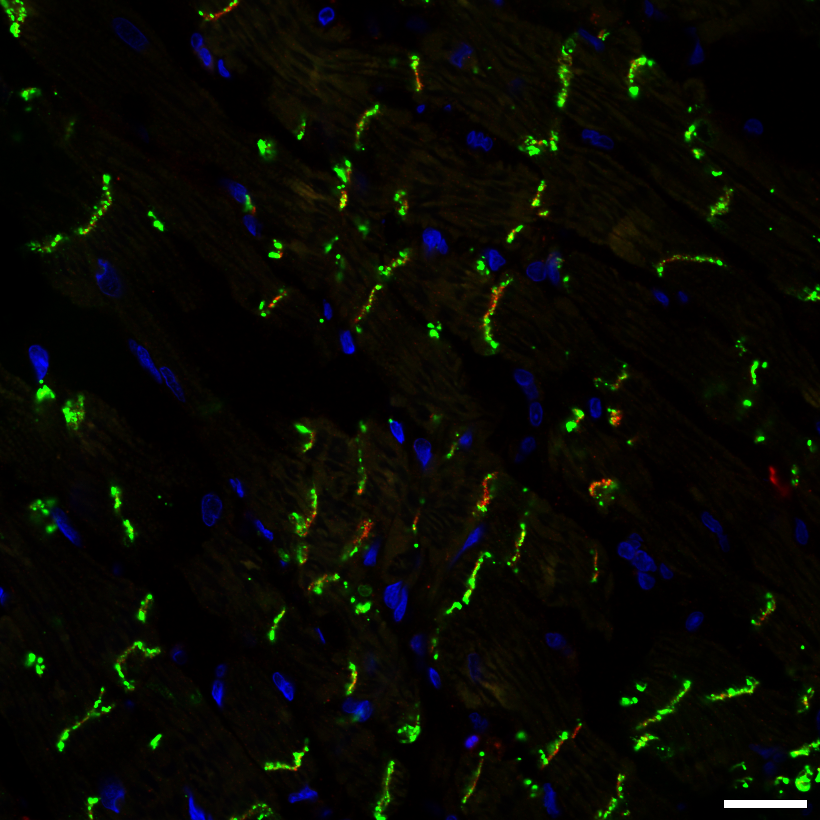

Supplement: Supplemental Information 18 [file peerj-13-19276-s018.zip › immunofluorescence AAV9-EB1(Cx43-N-cadherin)/AAV9-EB1 4-1.tif]

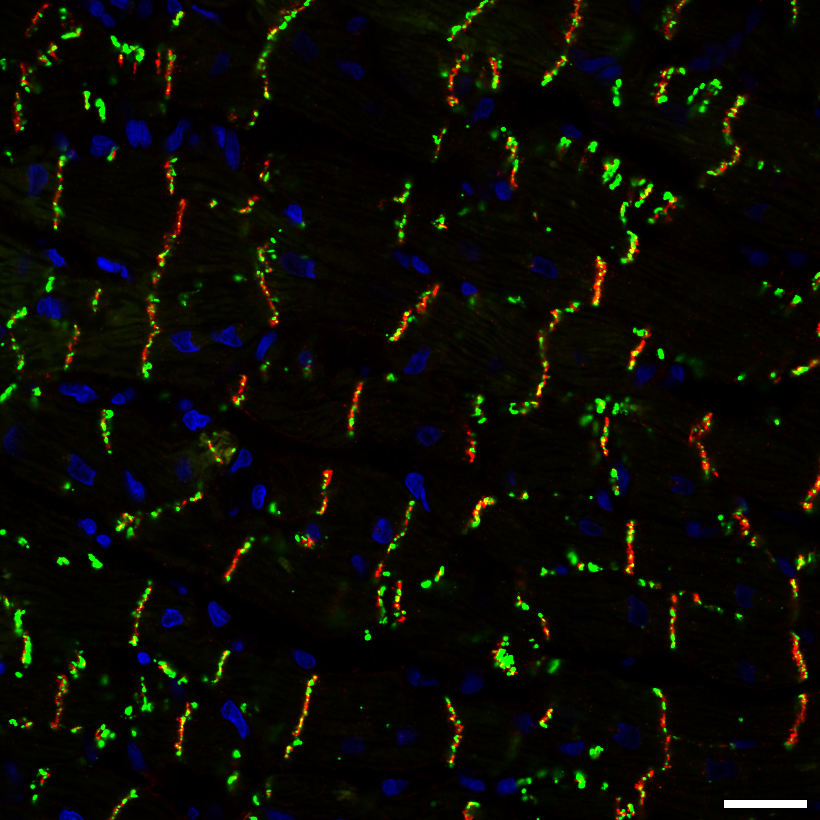

Supplement: Supplemental Information 18 [file peerj-13-19276-s018.zip › immunofluorescence AAV9-EB1(Cx43-N-cadherin)/AAV9-EB1 4-2.tif]

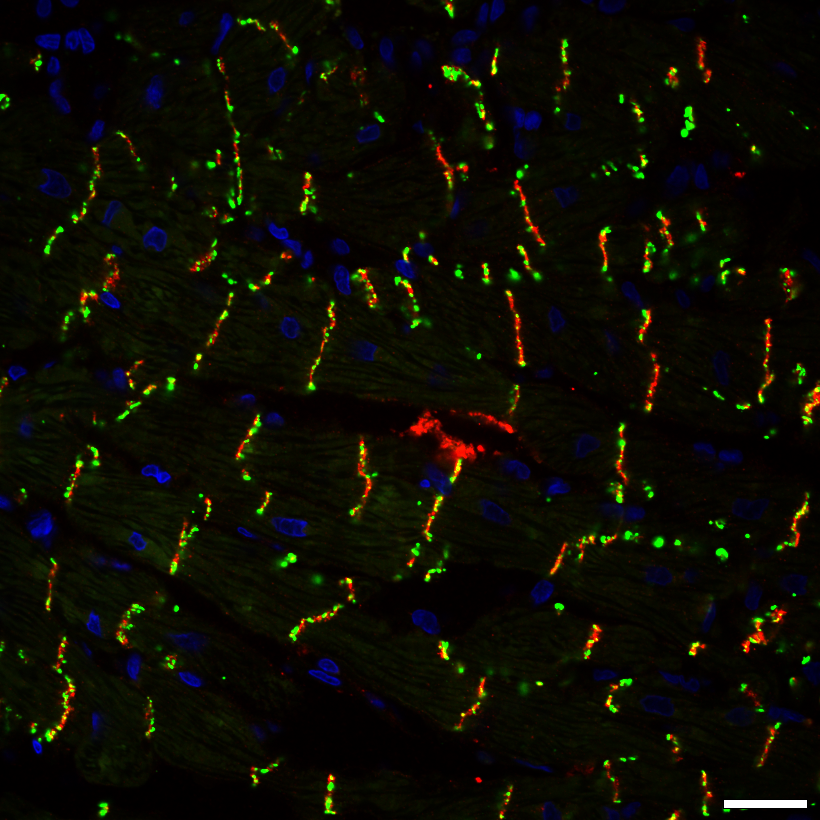

Supplement: Supplemental Information 18 [file peerj-13-19276-s018.zip › immunofluorescence AAV9-EB1(Cx43-N-cadherin)/AAV9-EB1 5-1.tif]

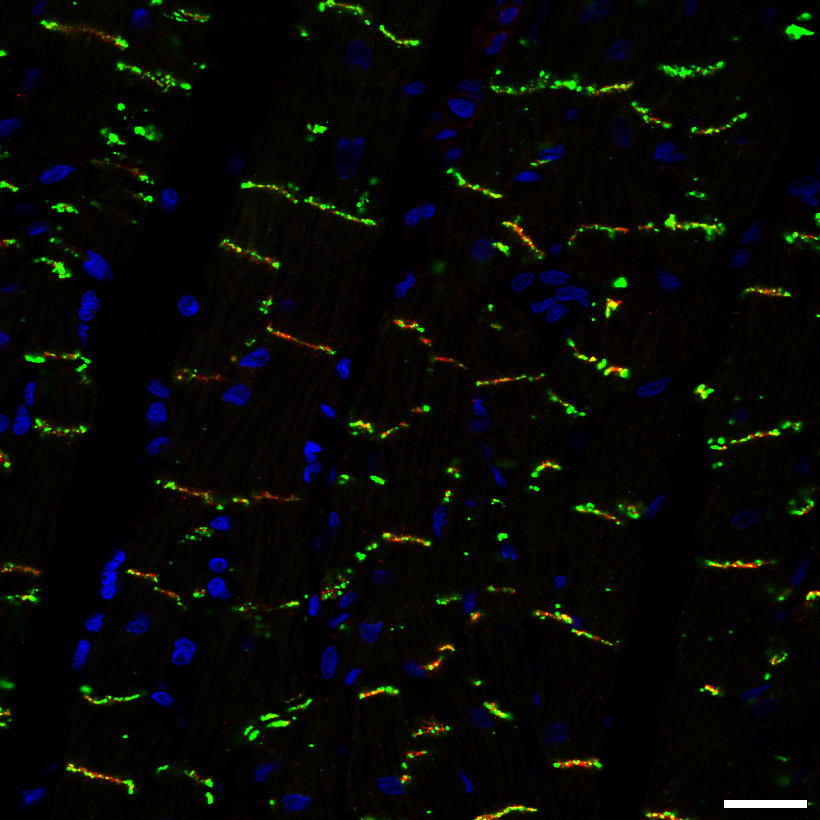

Supplement: Supplemental Information 18 [file peerj-13-19276-s018.zip › immunofluorescence AAV9-EB1(Cx43-N-cadherin)/AAV9-EB1 5-2.tif]

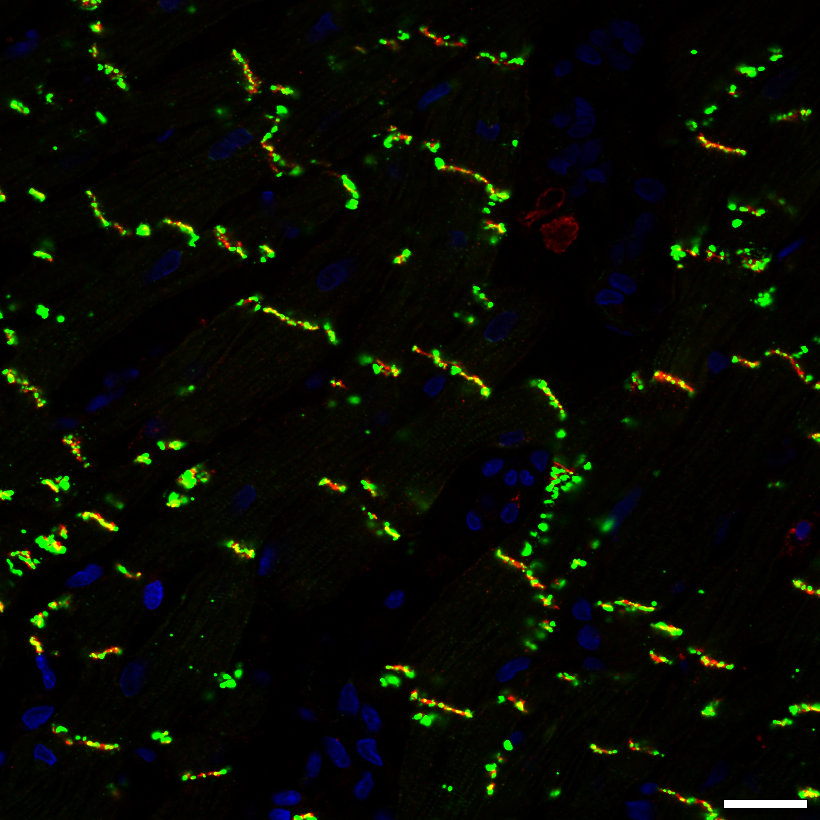

Supplement: Supplemental Information 18 [file peerj-13-19276-s018.zip › immunofluorescence AAV9-EB1(Cx43-N-cadherin)/AAV9-EB1 6-1.tif]

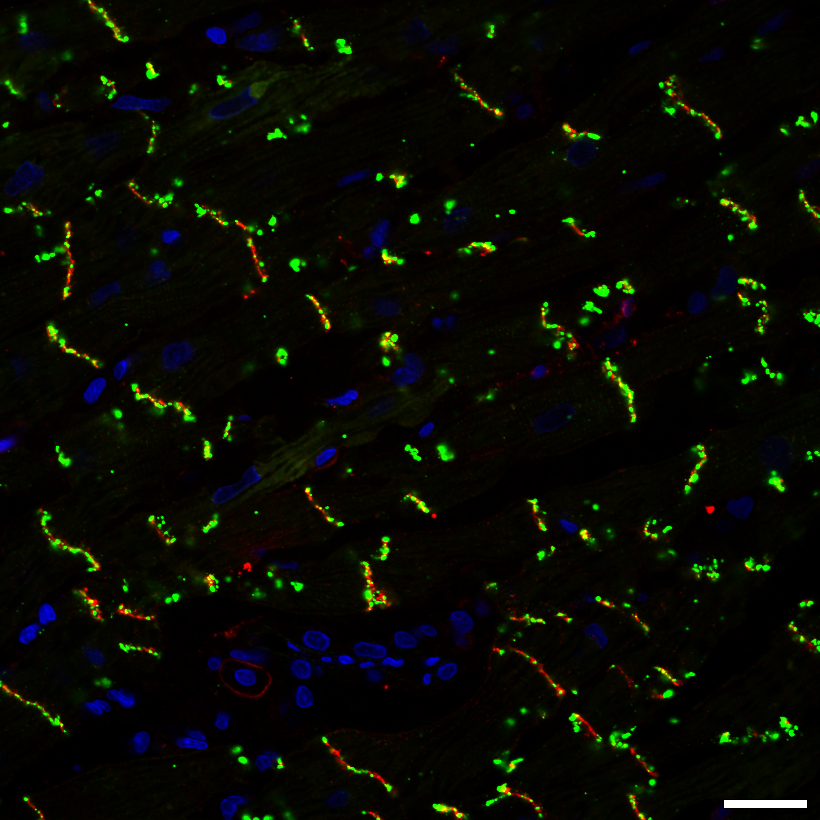

Supplement: Supplemental Information 18 [file peerj-13-19276-s018.zip › immunofluorescence AAV9-EB1(Cx43-N-cadherin)/AAV9-EB1 6-2.tif]

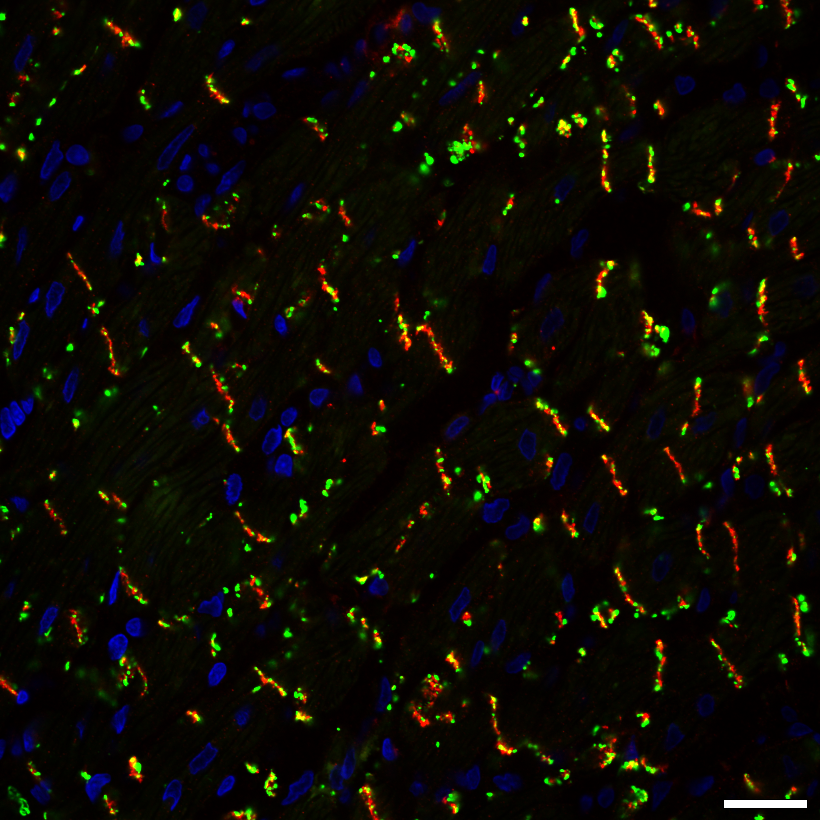

Supplement: Supplemental Information 18 [file peerj-13-19276-s018.zip › immunofluorescence AAV9-EB1(Cx43-N-cadherin)/AAV9-EB1 7-1.tif]

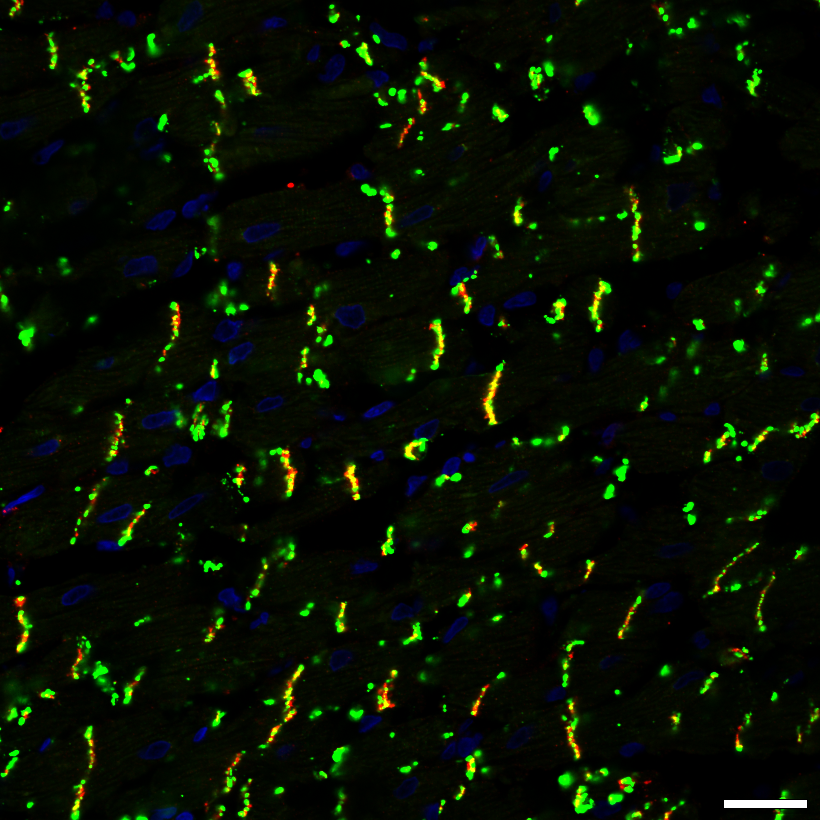

Supplement: Supplemental Information 18 [file peerj-13-19276-s018.zip › immunofluorescence AAV9-EB1(Cx43-N-cadherin)/AAV9-EB1 7-2.tif]

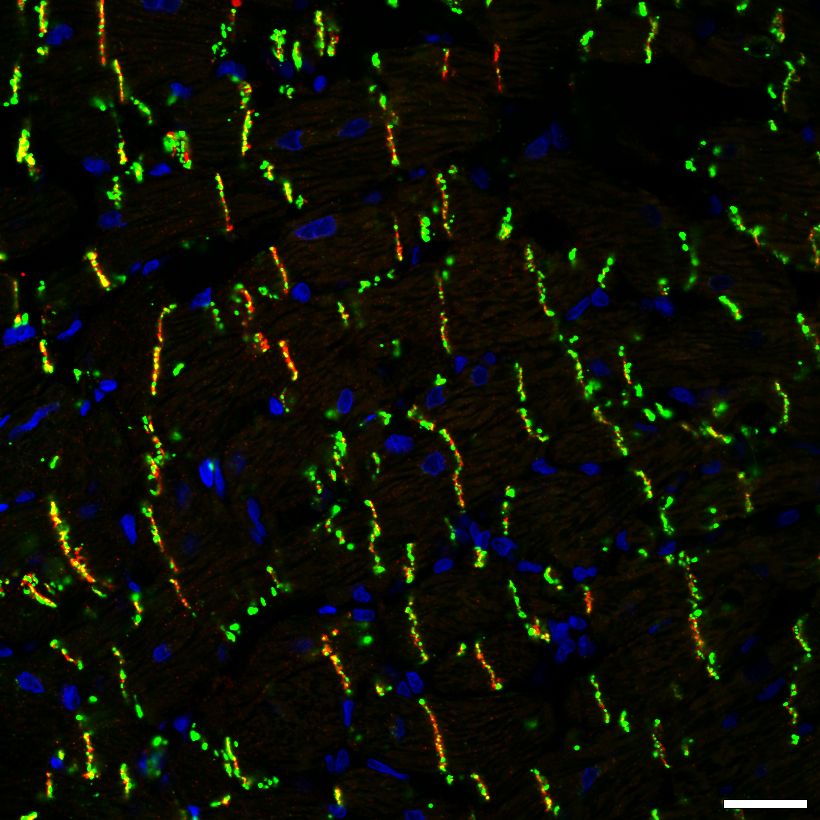

Supplement: Supplemental Information 18 [file peerj-13-19276-s018.zip › immunofluorescence AAV9-EB1(Cx43-N-cadherin)/AAV9-EB1 7-3.tif]

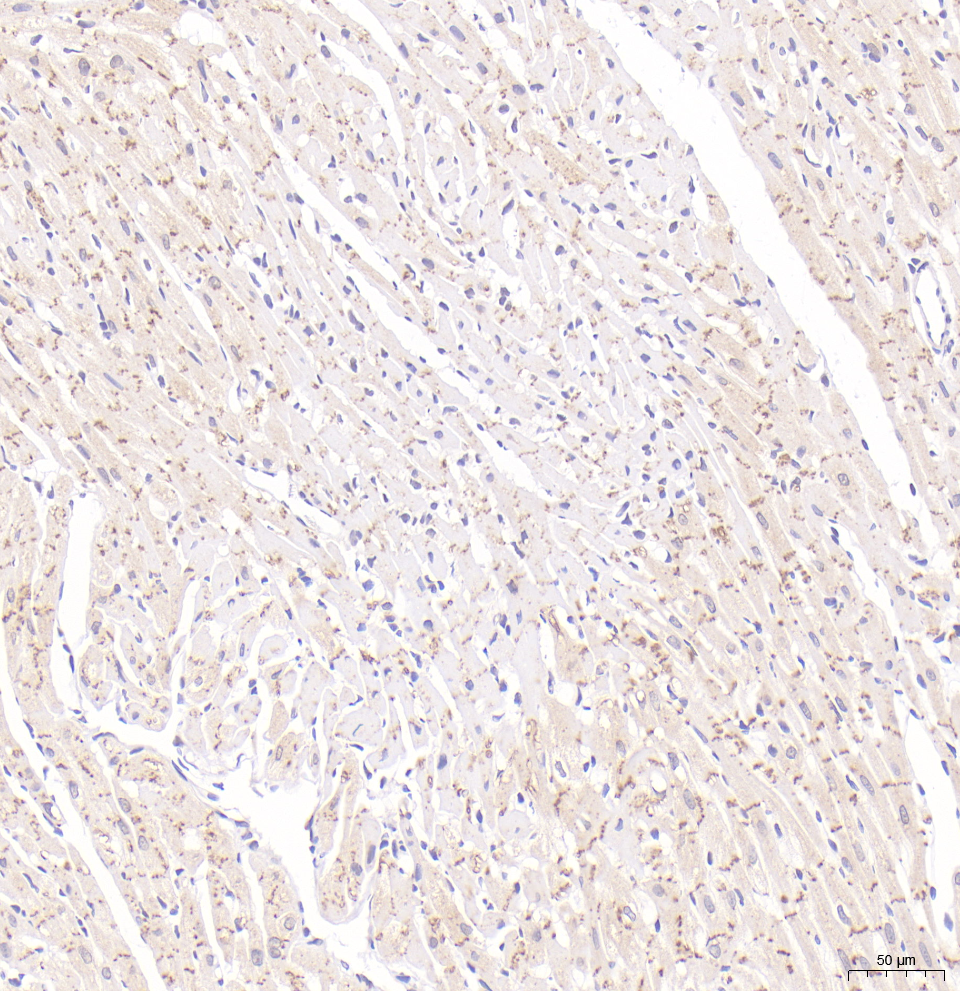

Supplement: Supplemental Information 21 [file peerj-13-19276-s021.zip › 1.immunohistochemical-C/C1-1.tif]

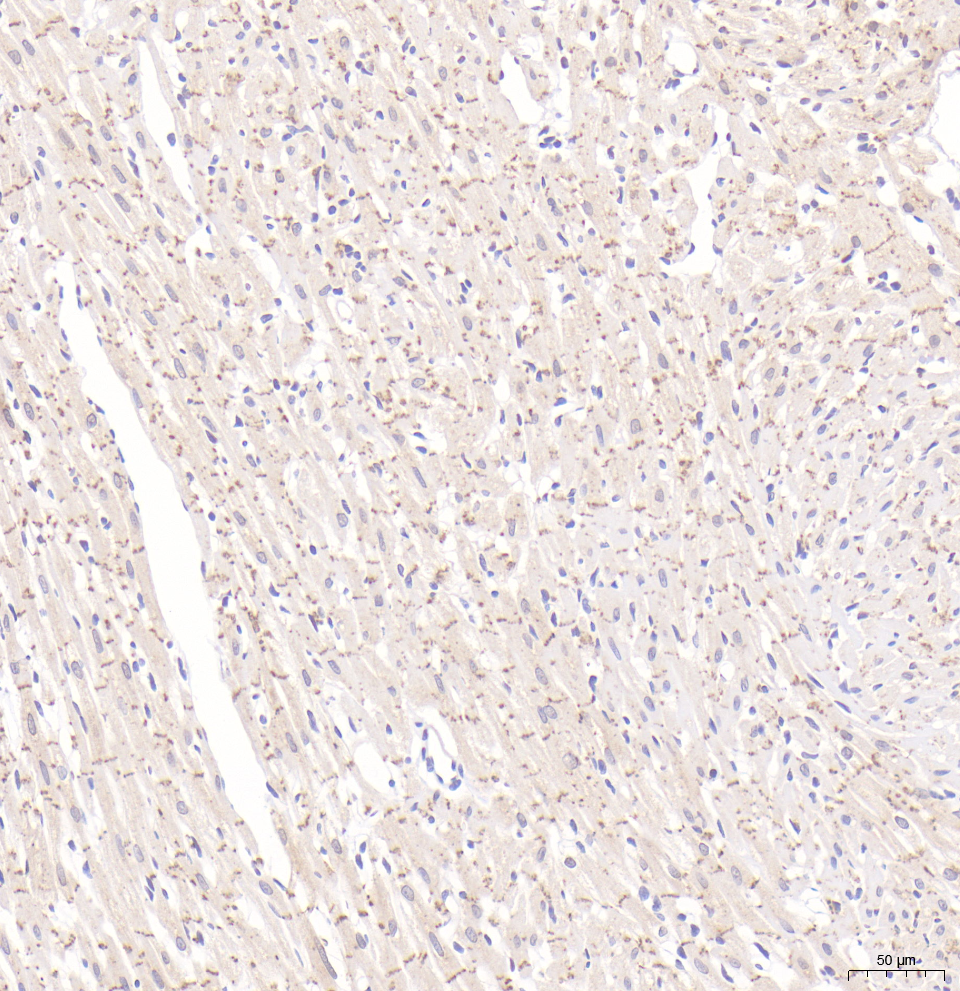

Supplement: Supplemental Information 21 [file peerj-13-19276-s021.zip › 1.immunohistochemical-C/C1-3.tif]

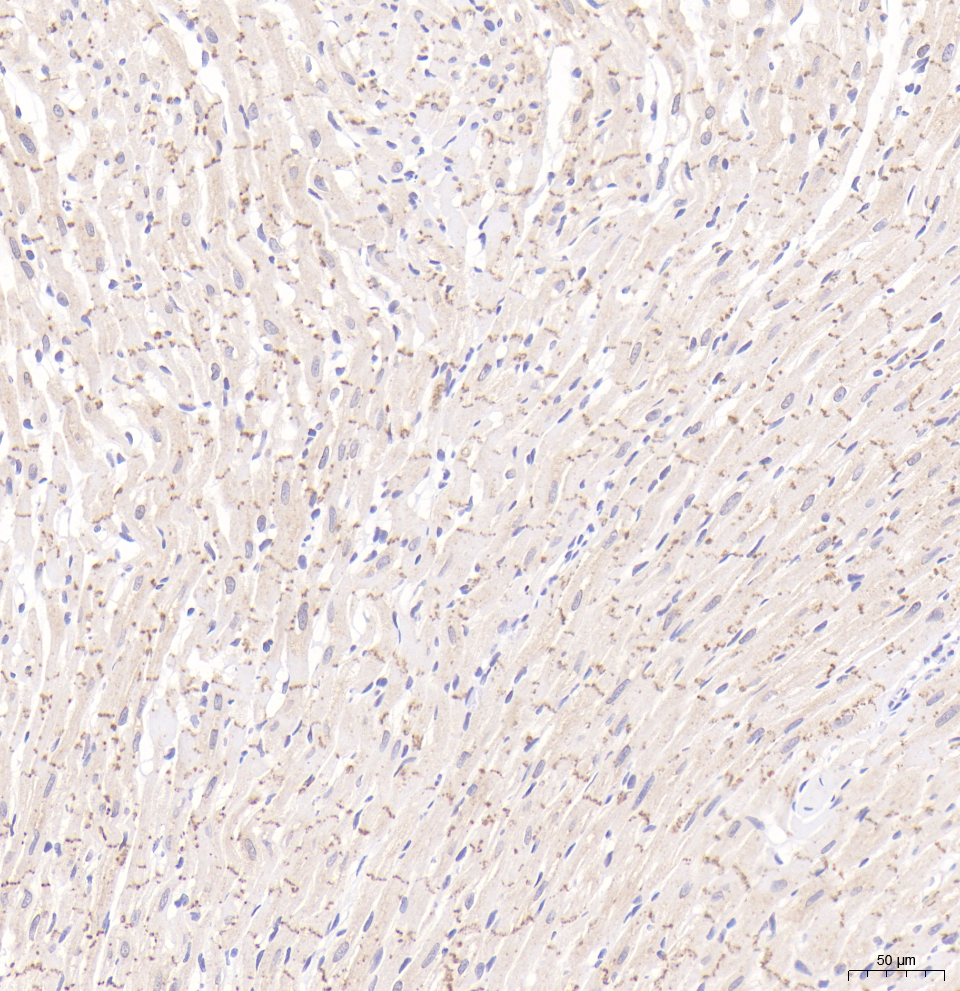

Supplement: Supplemental Information 21 [file peerj-13-19276-s021.zip › 1.immunohistochemical-C/C1-4.tif]

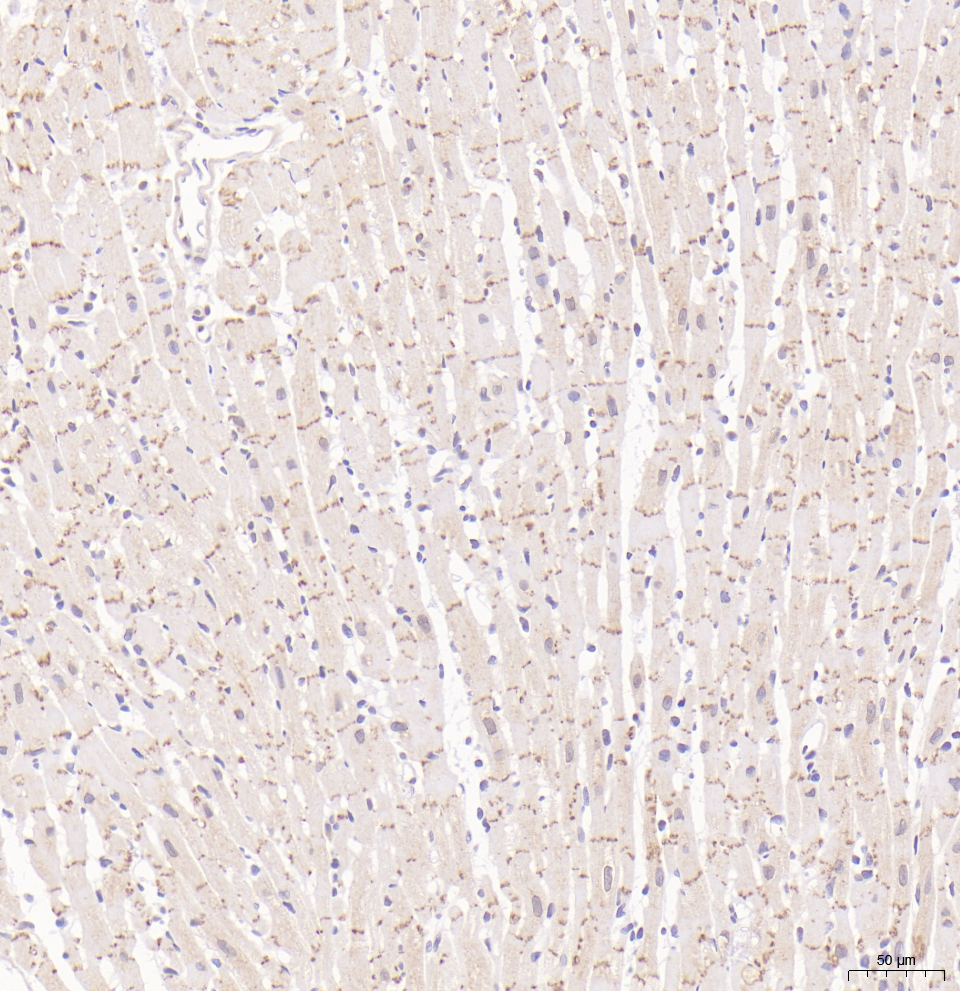

Supplement: Supplemental Information 21 [file peerj-13-19276-s021.zip › 1.immunohistochemical-C/C2-1.tif]

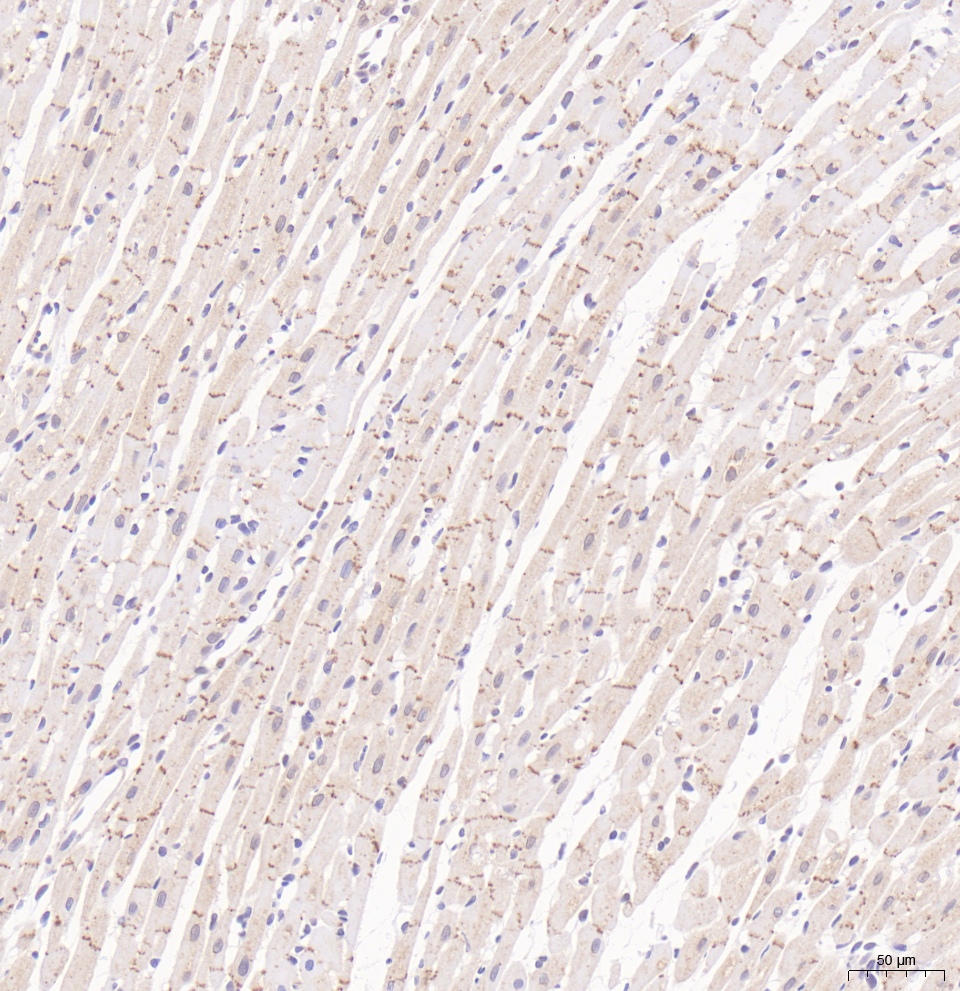

Supplement: Supplemental Information 21 [file peerj-13-19276-s021.zip › 1.immunohistochemical-C/C2-2.tif]

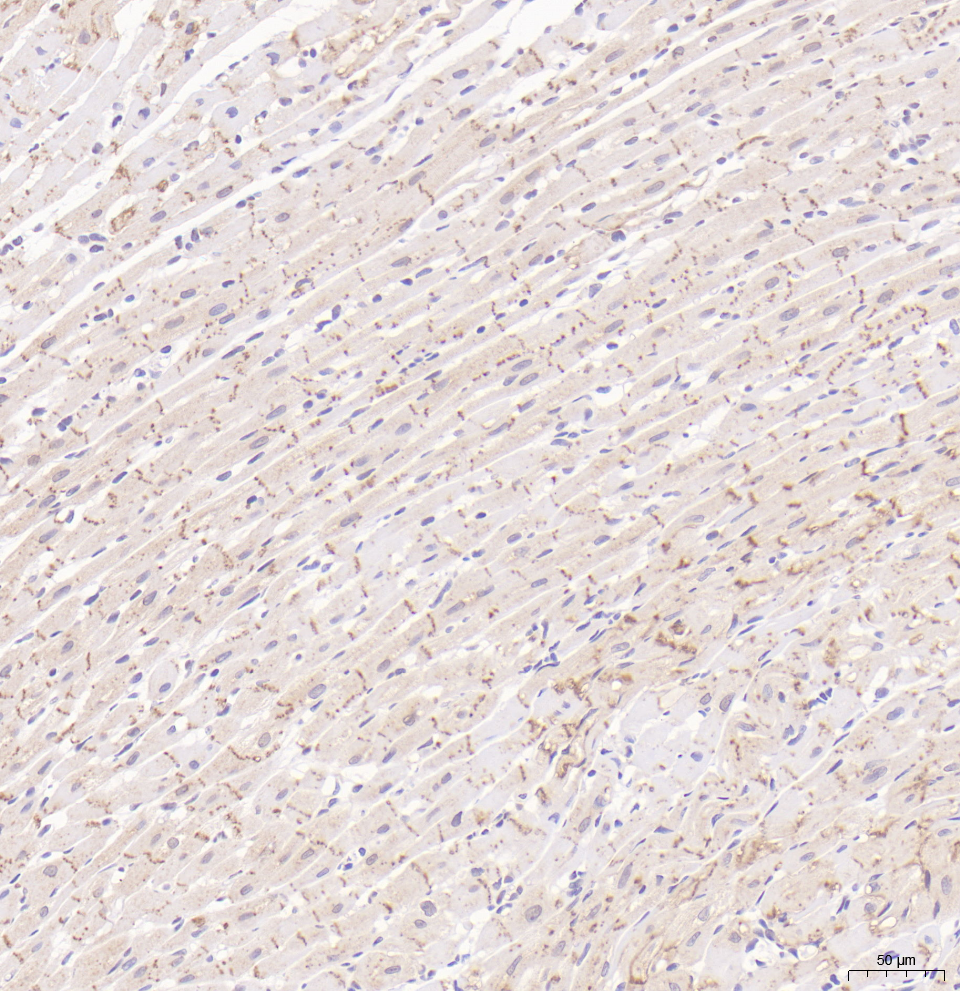

Supplement: Supplemental Information 21 [file peerj-13-19276-s021.zip › 1.immunohistochemical-C/C2-3.tif]

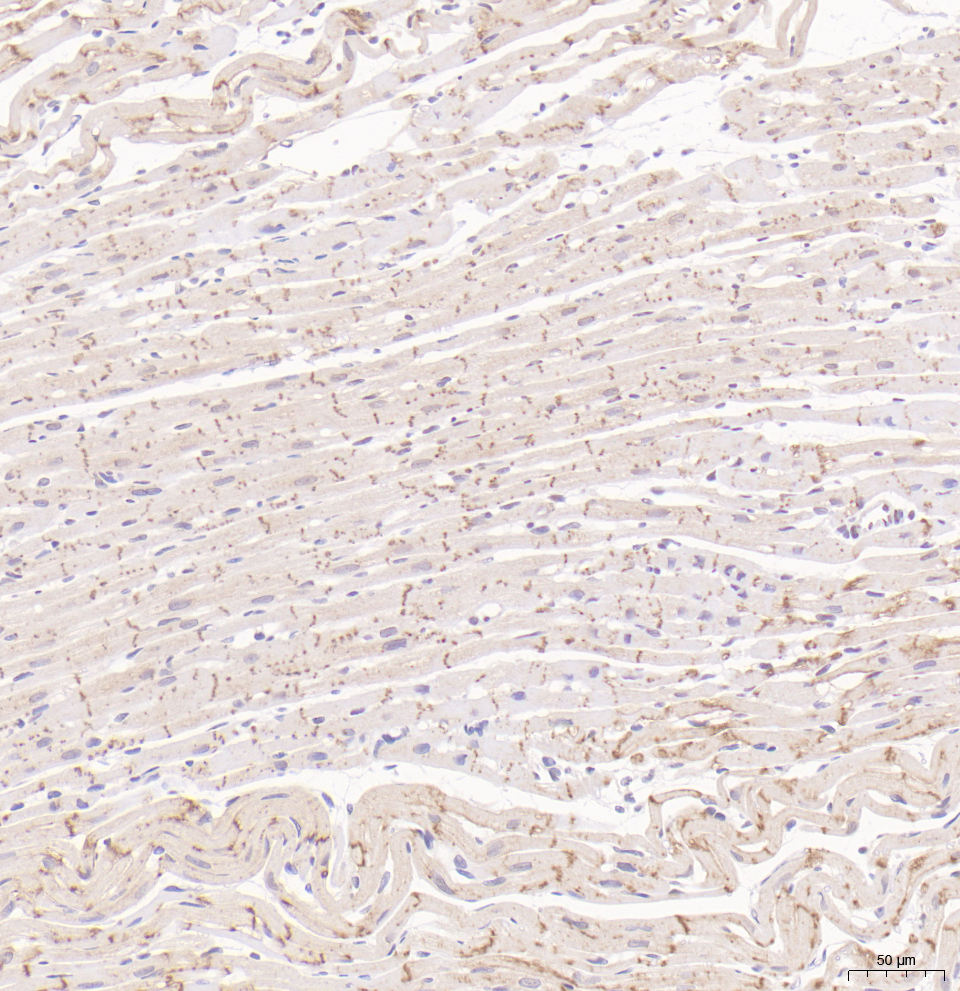

Supplement: Supplemental Information 22 [file peerj-13-19276-s022.zip › 2.immunohistochemical-C/C3-1.tif]

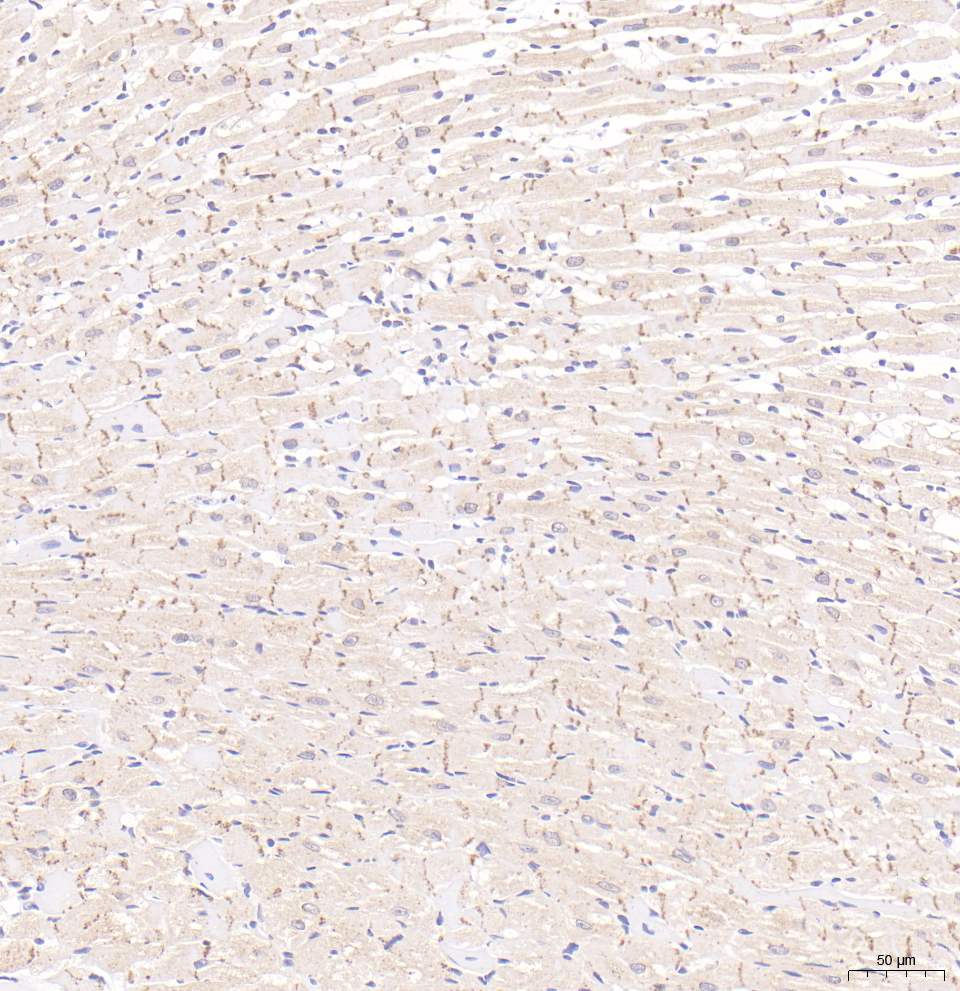

Supplement: Supplemental Information 22 [file peerj-13-19276-s022.zip › 2.immunohistochemical-C/C3-2.tif]

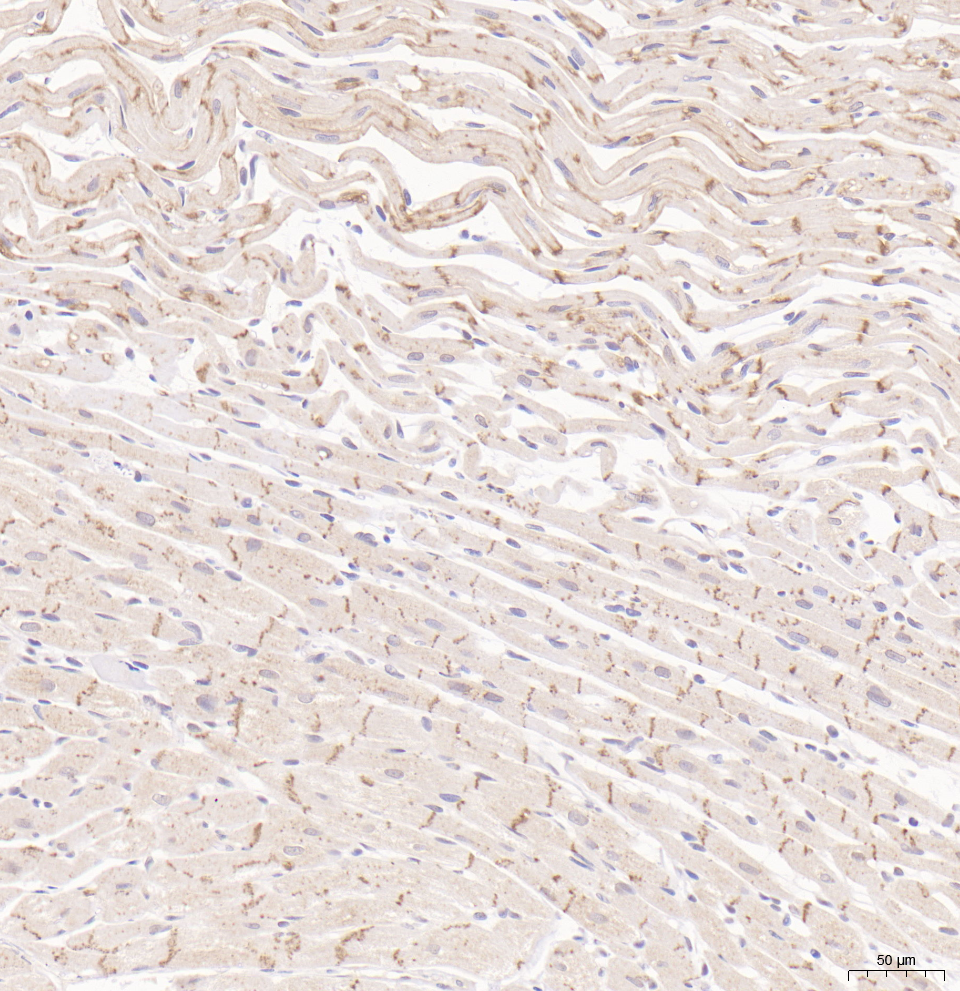

Supplement: Supplemental Information 22 [file peerj-13-19276-s022.zip › 2.immunohistochemical-C/C3-3.tif]

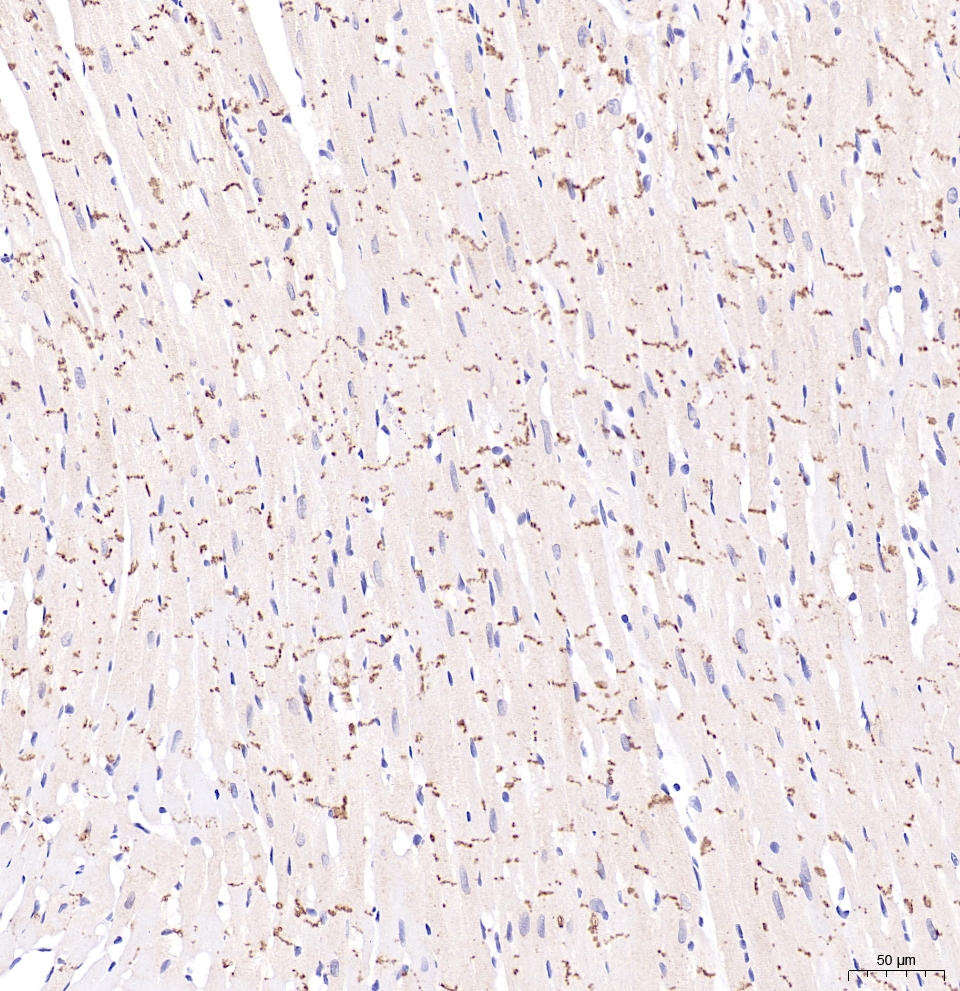

Supplement: Supplemental Information 22 [file peerj-13-19276-s022.zip › 2.immunohistochemical-C/C4-1.tif]

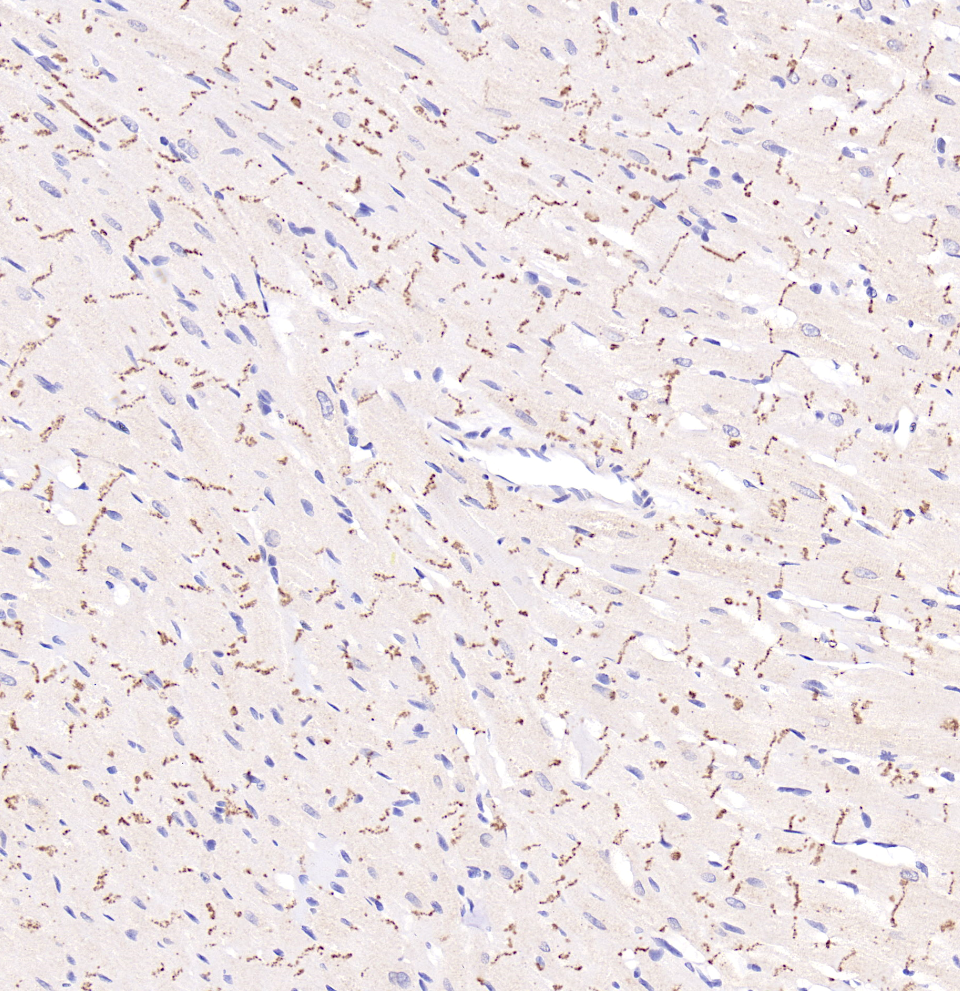

Supplement: Supplemental Information 22 [file peerj-13-19276-s022.zip › 2.immunohistochemical-C/C4-2.tif]

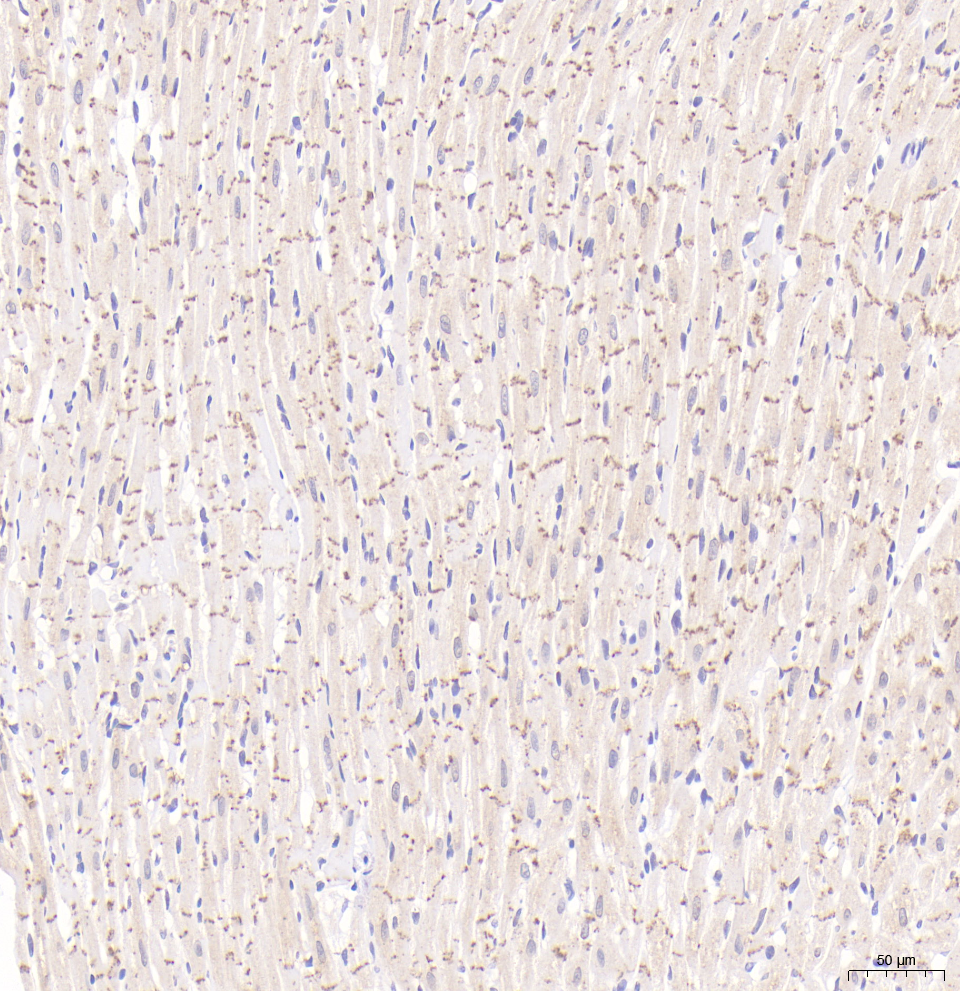

Supplement: Supplemental Information 22 [file peerj-13-19276-s022.zip › 2.immunohistochemical-C/C4-3.tif]

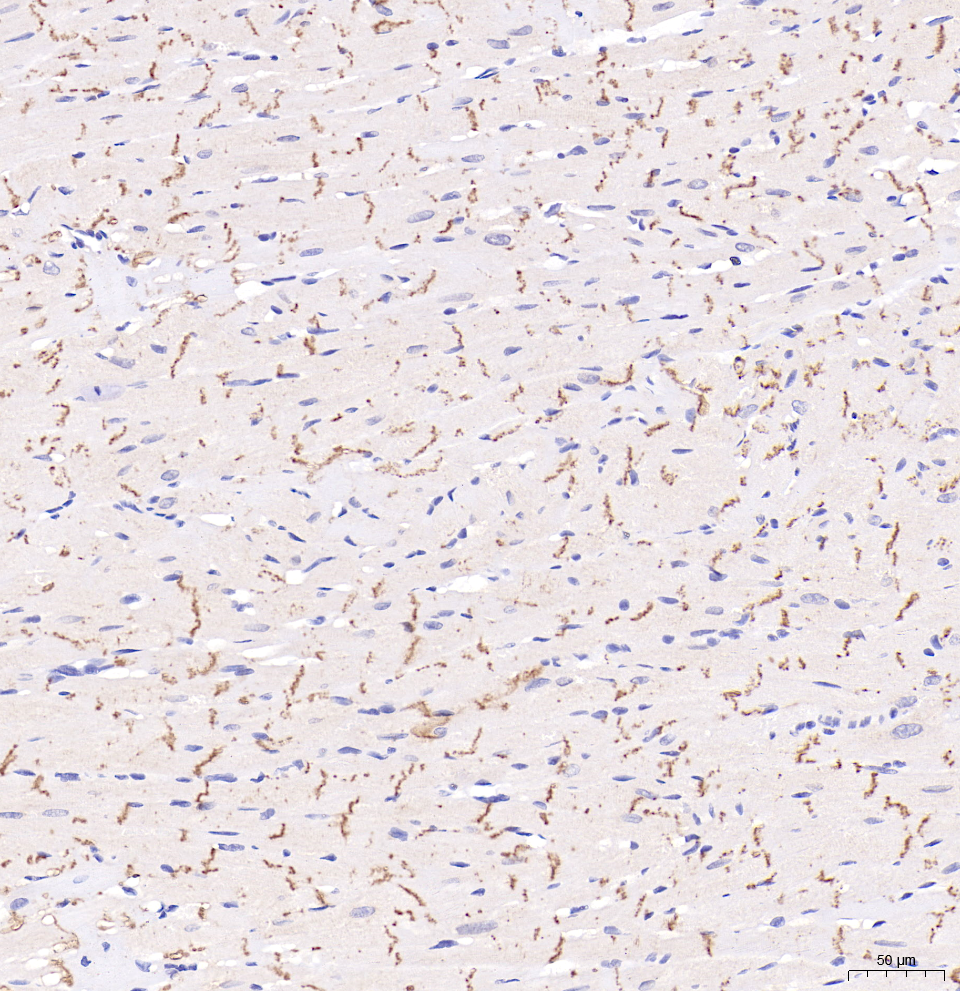

Supplement: Supplemental Information 22 [file peerj-13-19276-s022.zip › 2.immunohistochemical-C/C5-1.tif]

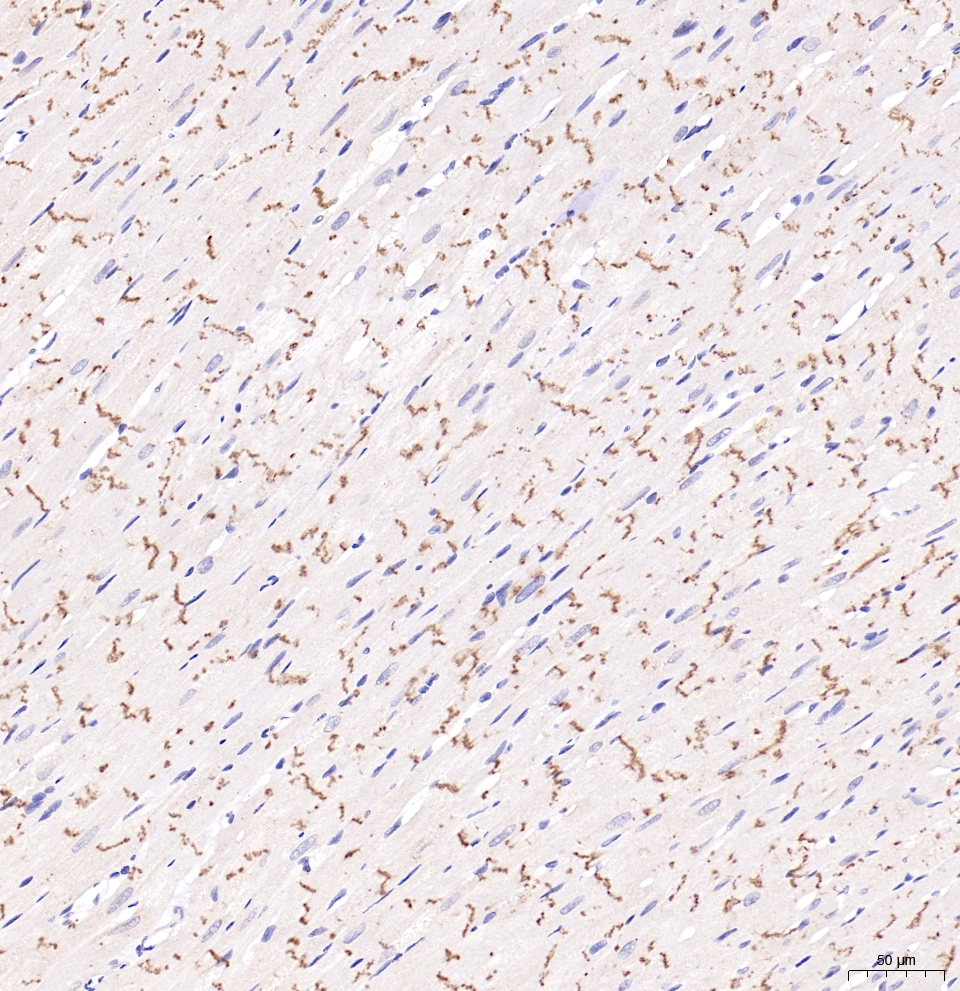

Supplement: Supplemental Information 22 [file peerj-13-19276-s022.zip › 2.immunohistochemical-C/C5-2.tif]

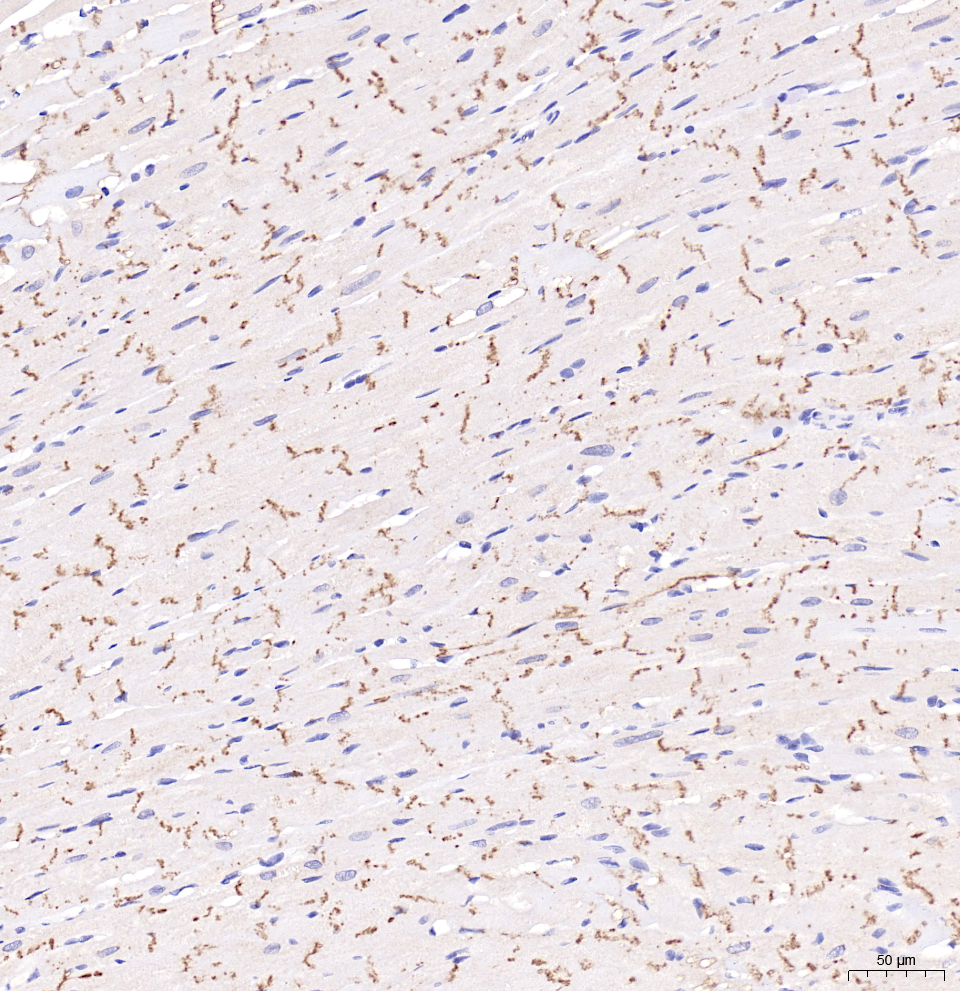

Supplement: Supplemental Information 22 [file peerj-13-19276-s022.zip › 2.immunohistochemical-C/C5-3.tif]

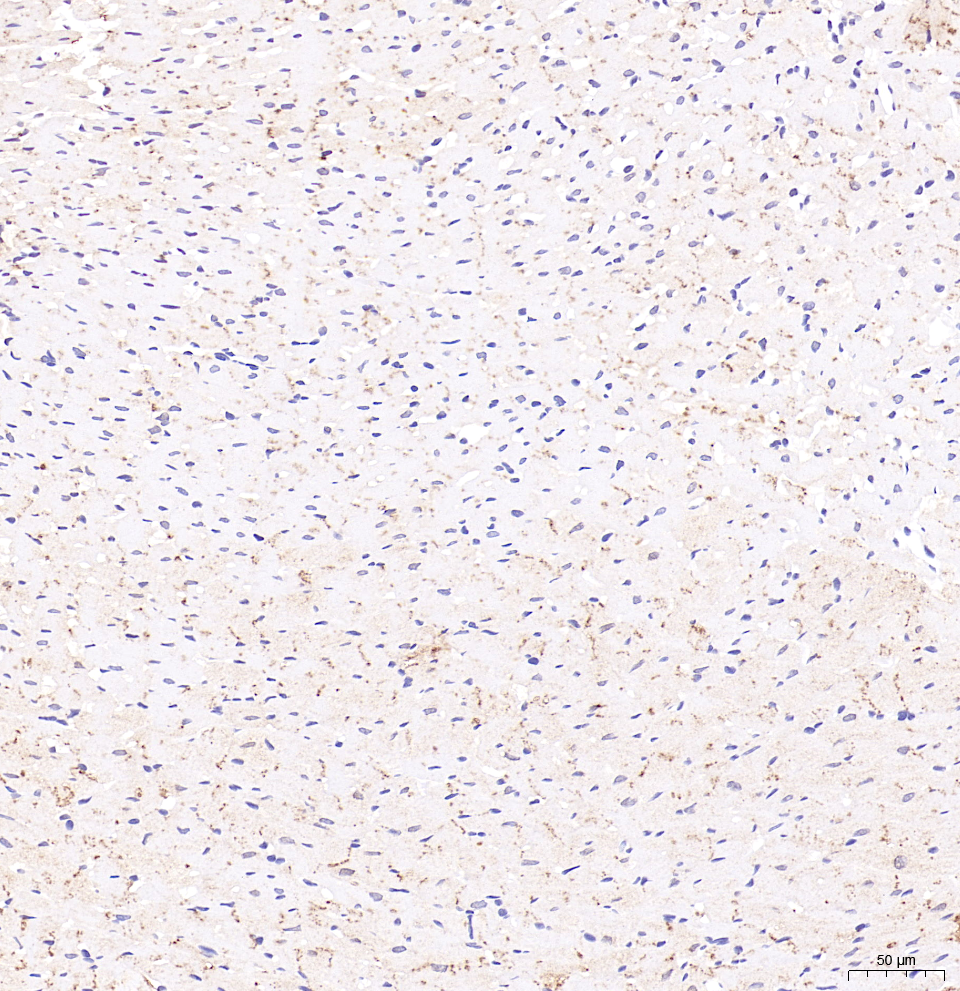

Supplement: Supplemental Information 23 [file peerj-13-19276-s023.zip › 1.immunohistochemical-I/R/I/R1-1.tif]

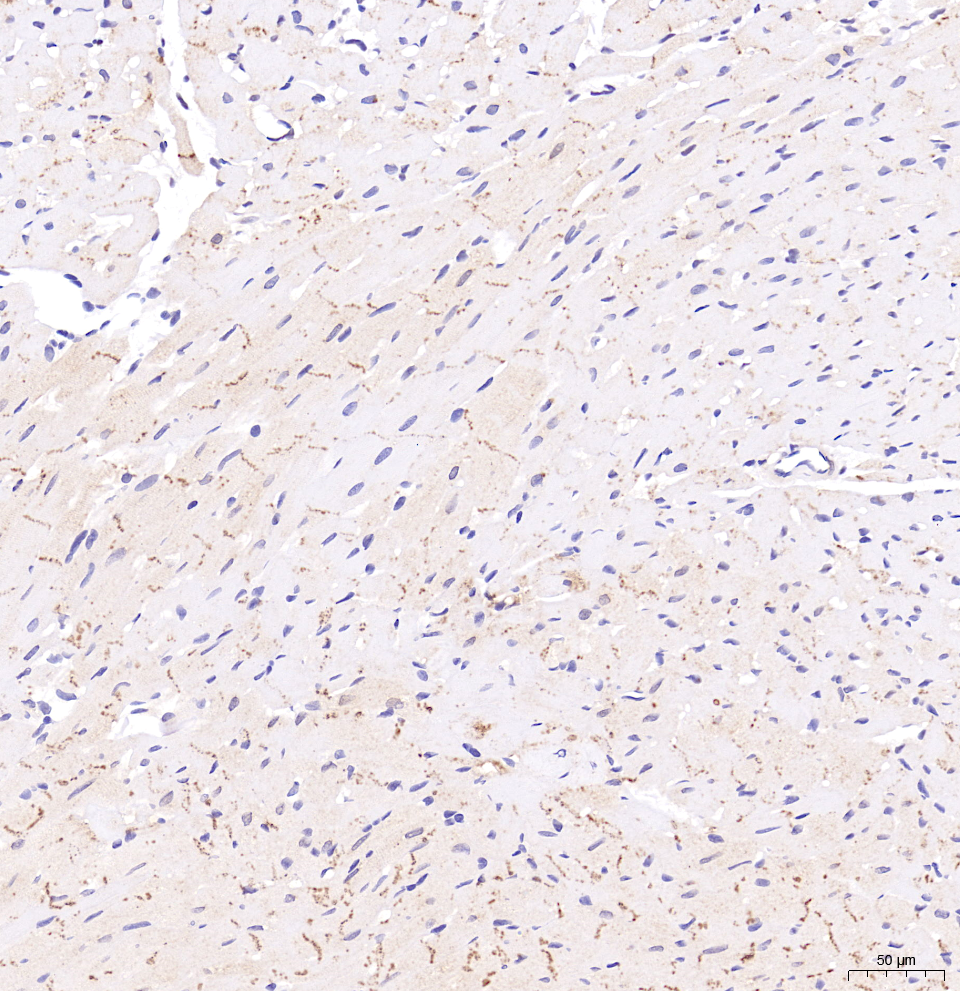

Supplement: Supplemental Information 23 [file peerj-13-19276-s023.zip › 1.immunohistochemical-I/R/I/R1-2.tif]

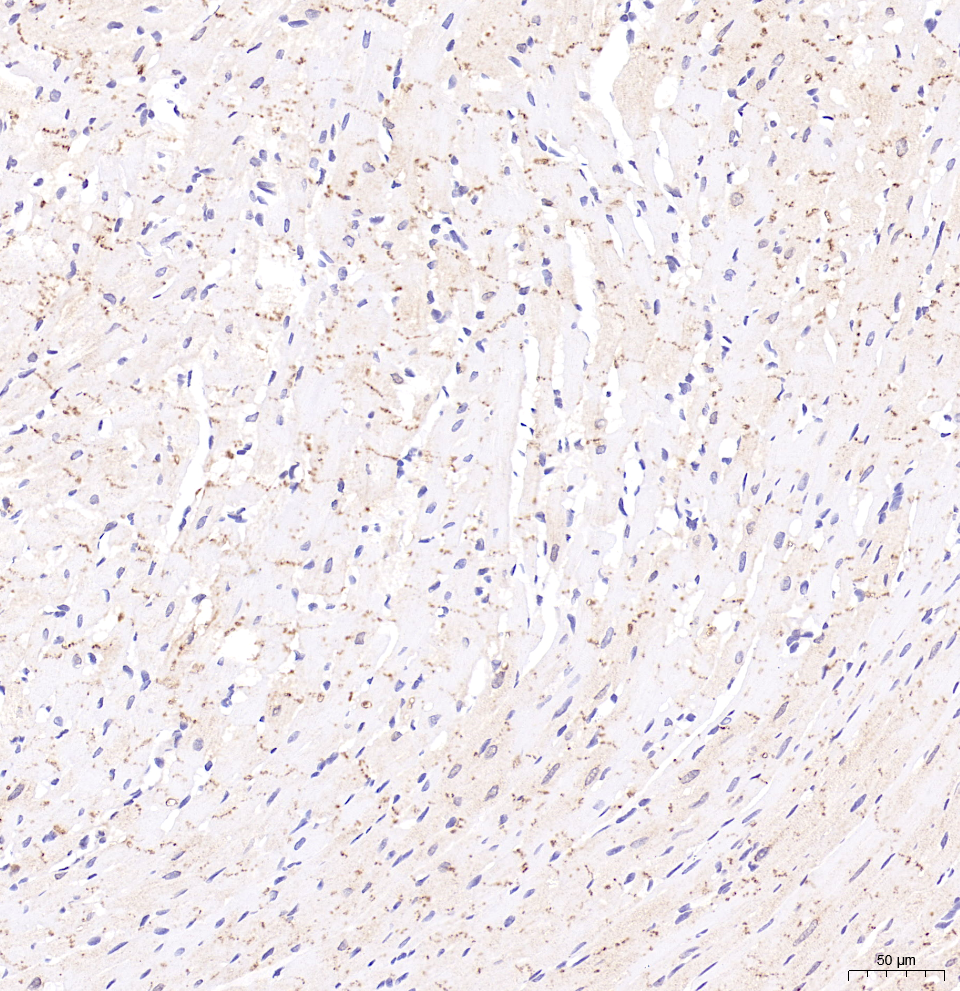

Supplement: Supplemental Information 23 [file peerj-13-19276-s023.zip › 1.immunohistochemical-I/R/I/R1-3.tif]

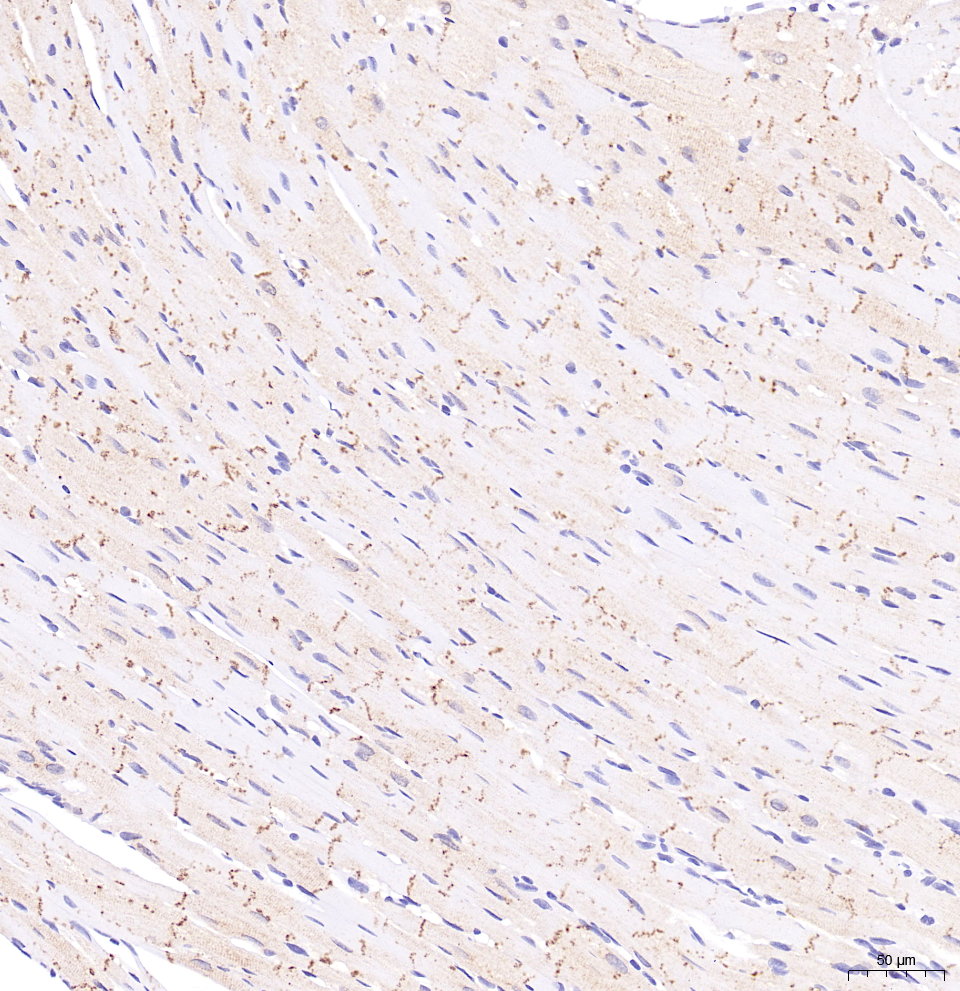

Supplement: Supplemental Information 23 [file peerj-13-19276-s023.zip › 1.immunohistochemical-I/R/I/R3-1.tif]

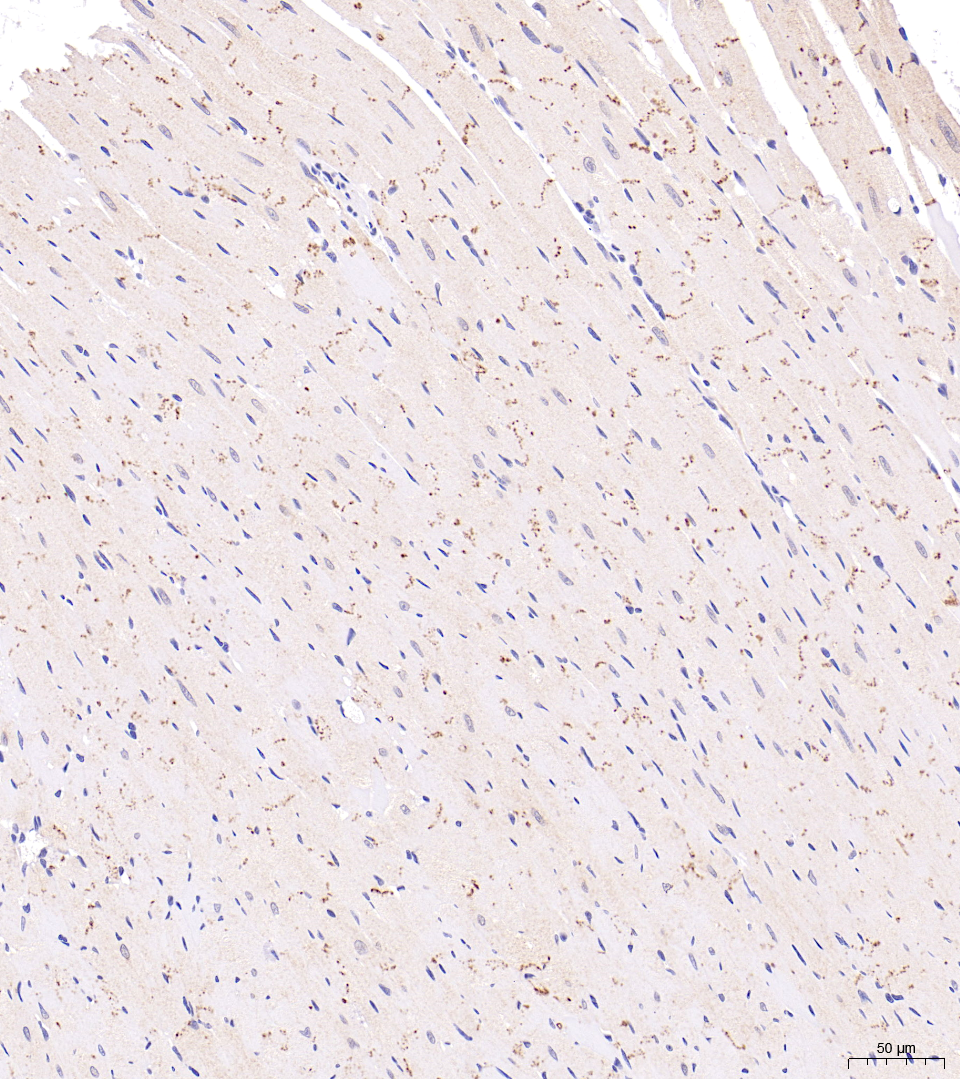

Supplement: Supplemental Information 23 [file peerj-13-19276-s023.zip › 1.immunohistochemical-I/R/I/R3-2.tif]

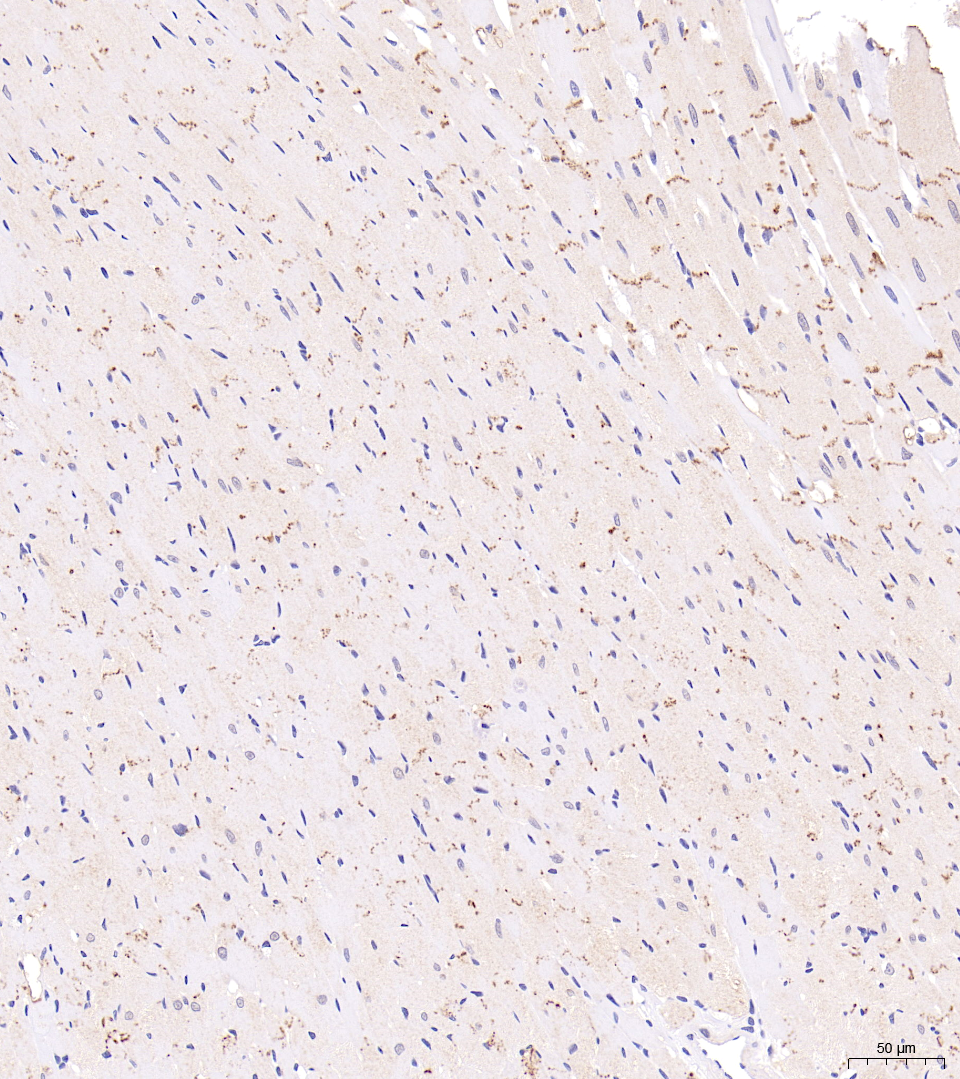

Supplement: Supplemental Information 23 [file peerj-13-19276-s023.zip › 1.immunohistochemical-I/R/I/R3-3.tif]

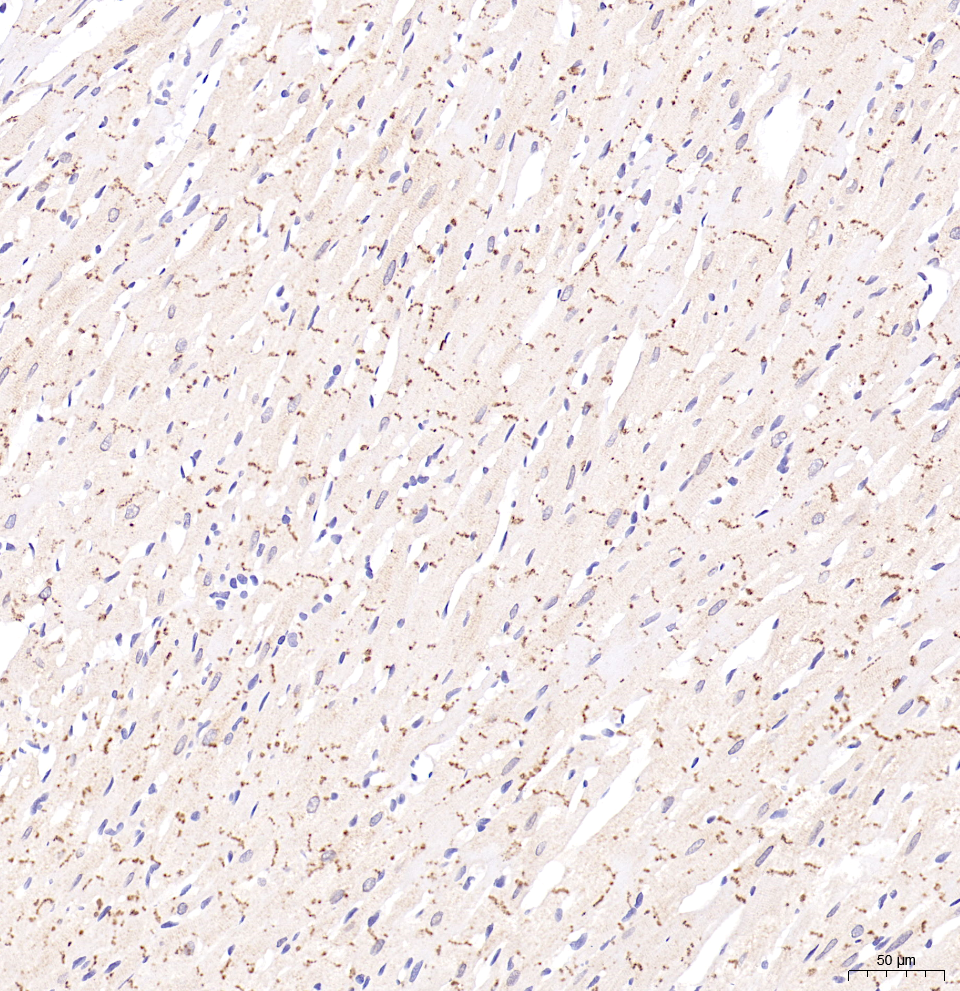

Supplement: Supplemental Information 23 [file peerj-13-19276-s023.zip › 1.immunohistochemical-I/R/I/R4- 2.tif]

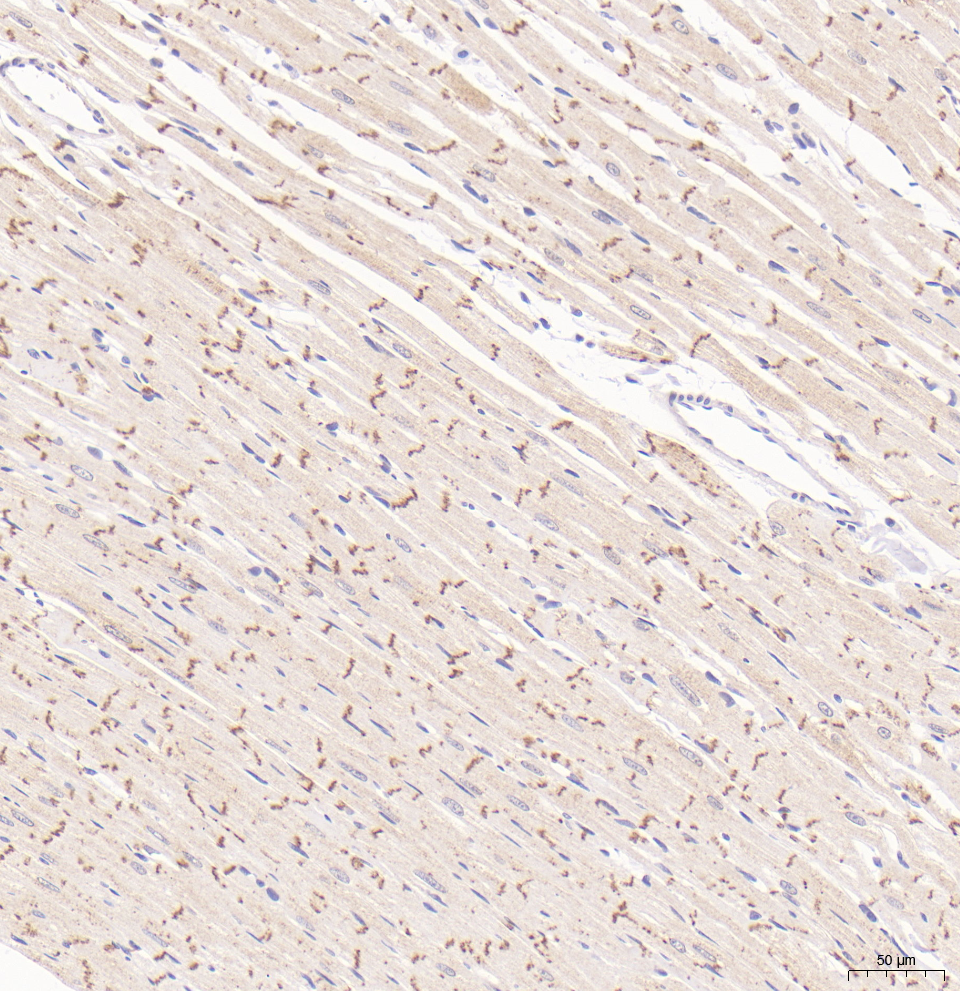

Supplement: Supplemental Information 24 [file peerj-13-19276-s024.zip › 2.immunohistochemical-I/R/I/R5-1.tif]

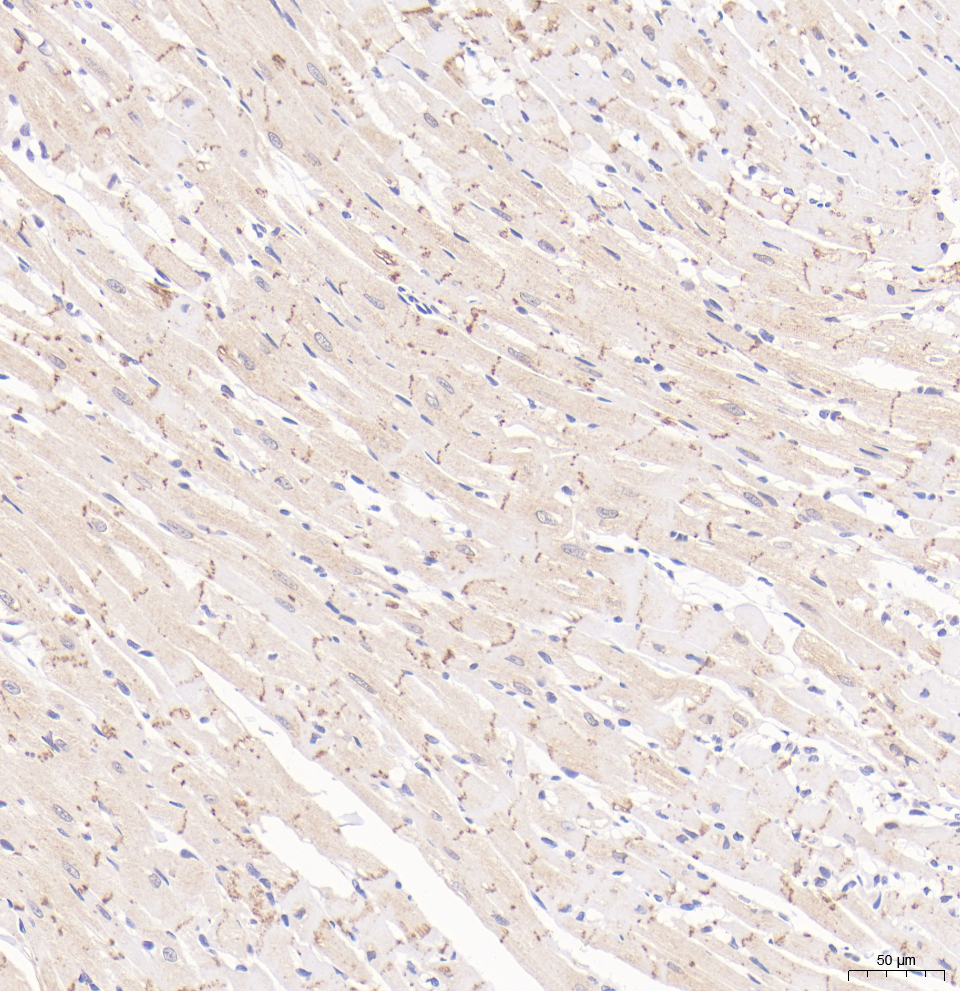

Supplement: Supplemental Information 24 [file peerj-13-19276-s024.zip › 2.immunohistochemical-I/R/I/R5-2.tif]

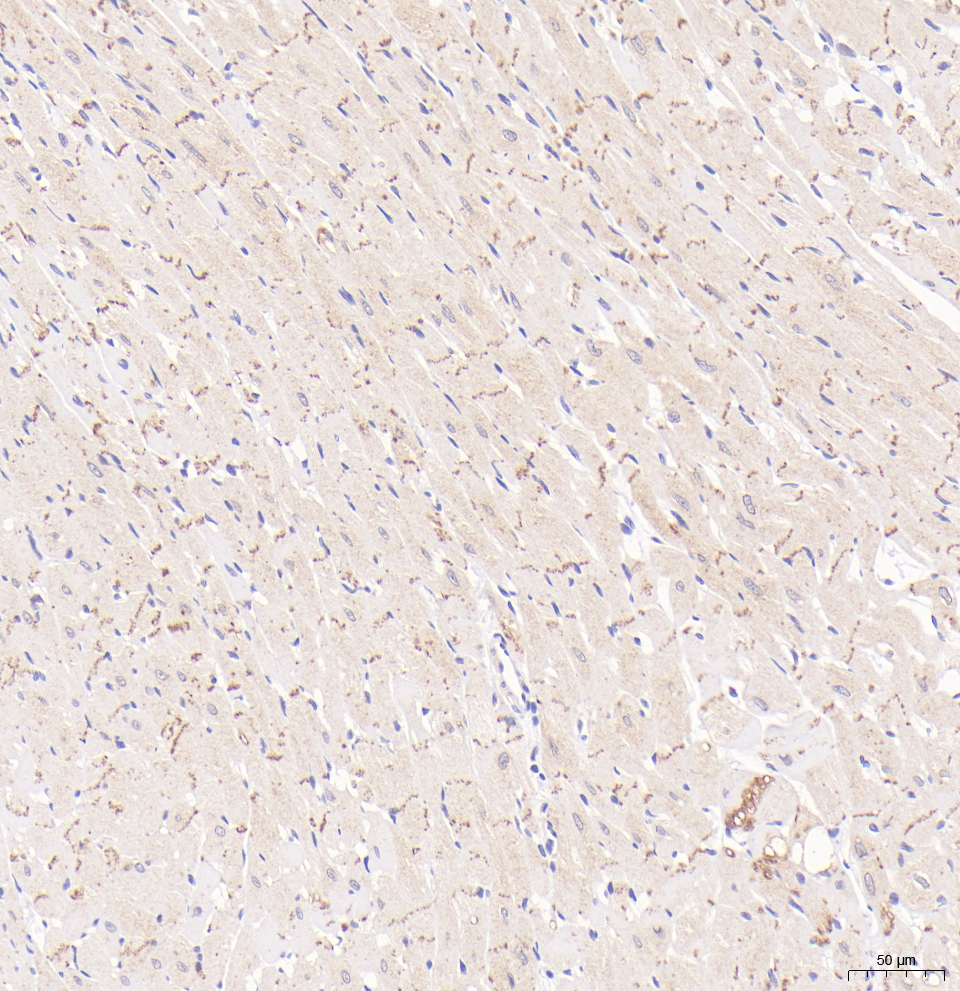

Supplement: Supplemental Information 24 [file peerj-13-19276-s024.zip › 2.immunohistochemical-I/R/I/R5-3.tif]

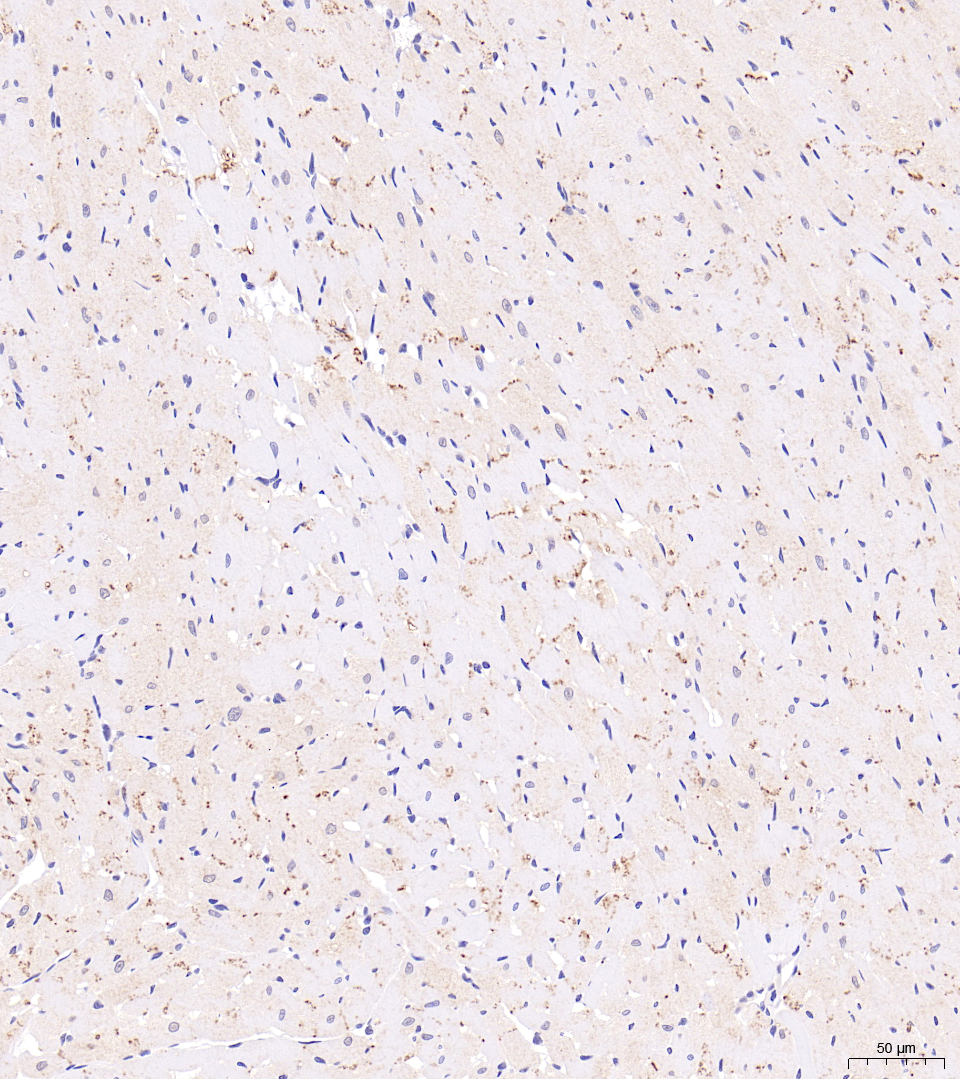

Supplement: Supplemental Information 24 [file peerj-13-19276-s024.zip › 2.immunohistochemical-I/R/I/R6-1.tif]

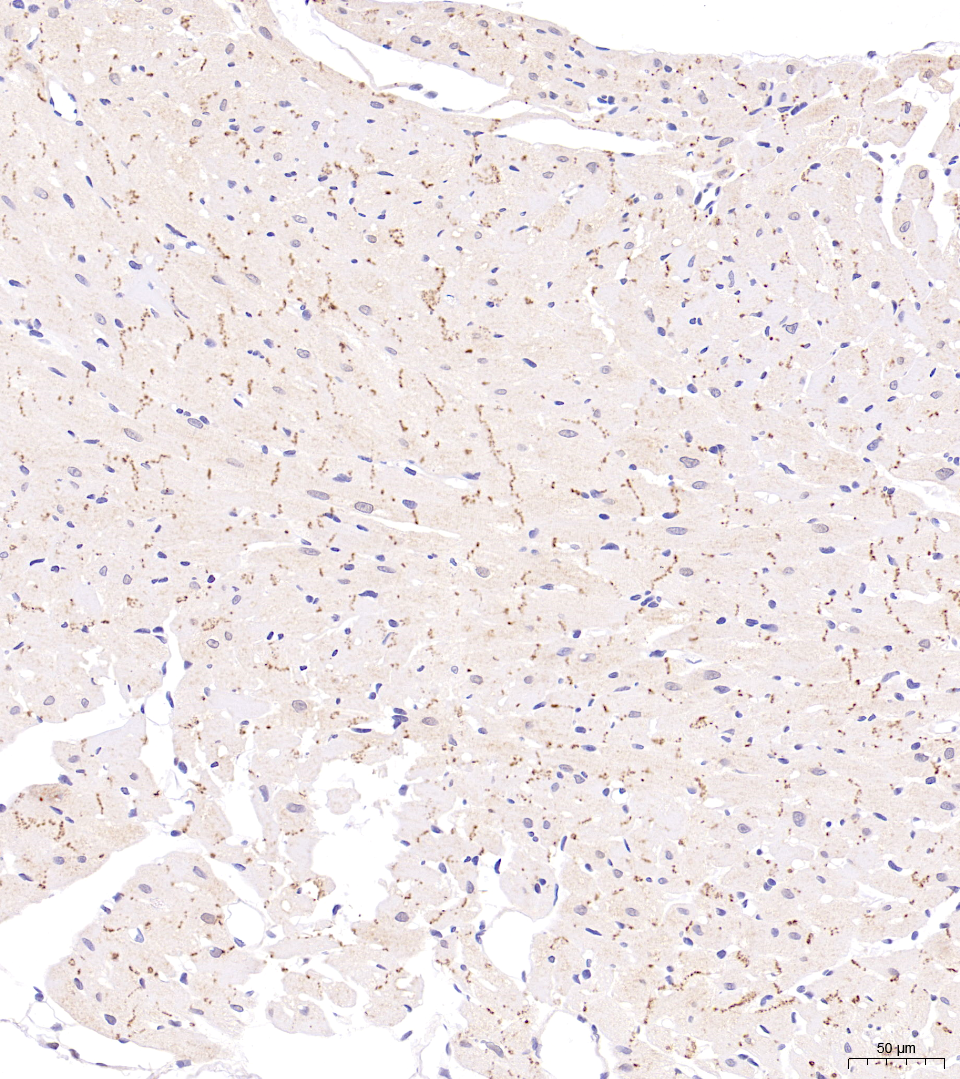

Supplement: Supplemental Information 24 [file peerj-13-19276-s024.zip › 2.immunohistochemical-I/R/I/R6-2.tif]

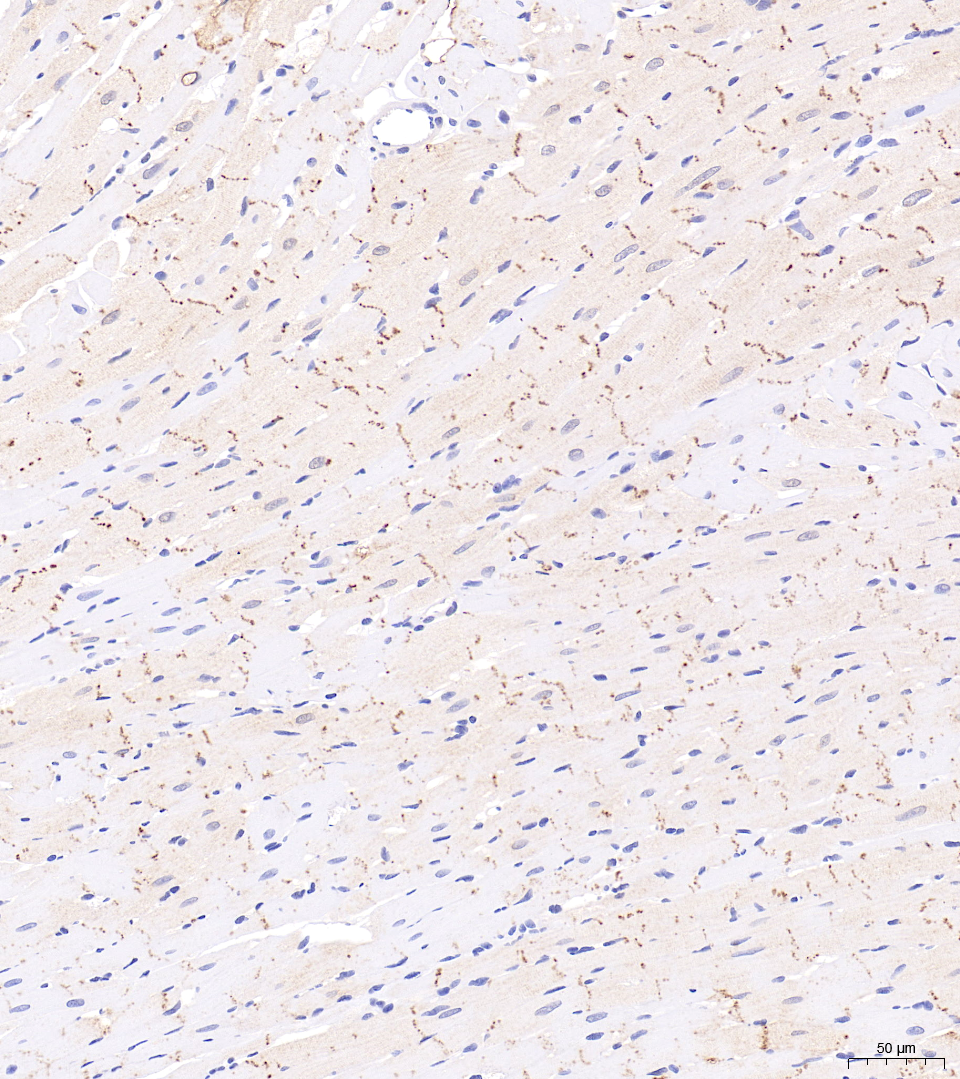

Supplement: Supplemental Information 24 [file peerj-13-19276-s024.zip › 2.immunohistochemical-I/R/I/R6-3.tif]

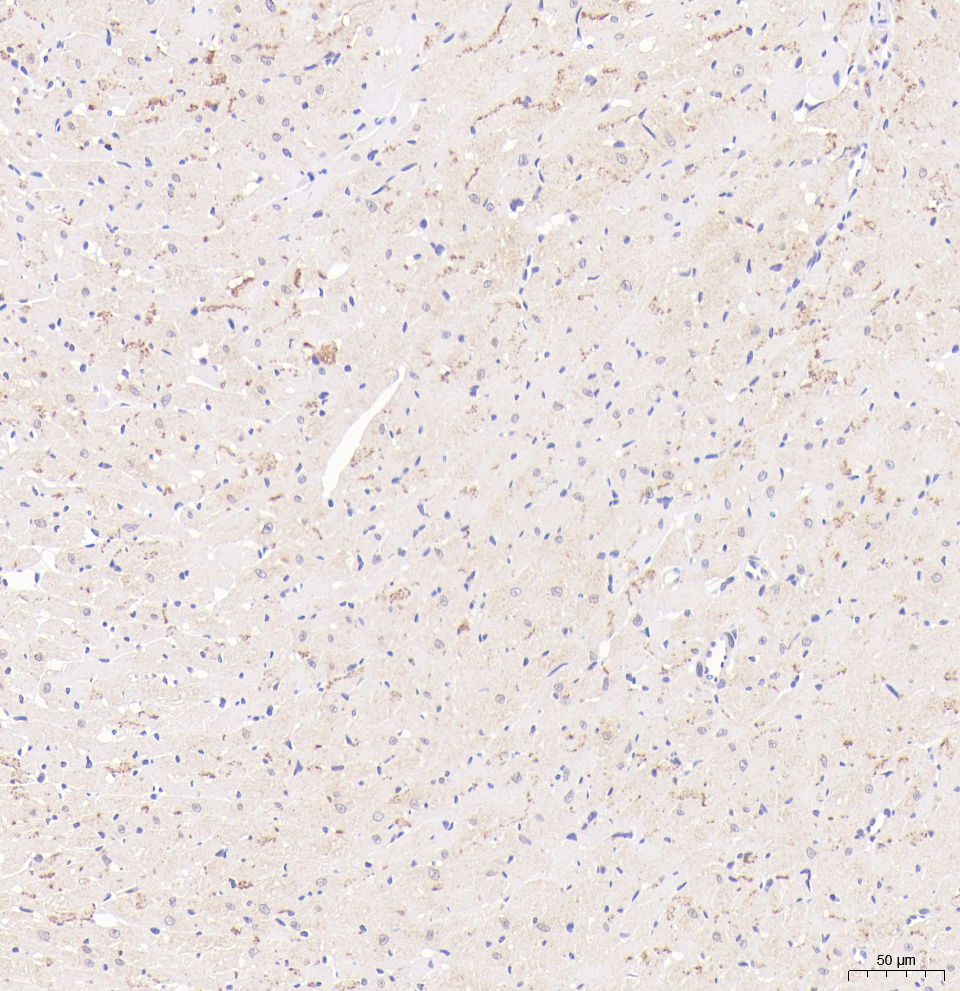

Supplement: Supplemental Information 24 [file peerj-13-19276-s024.zip › 2.immunohistochemical-I/R/I/R7-1.tif]

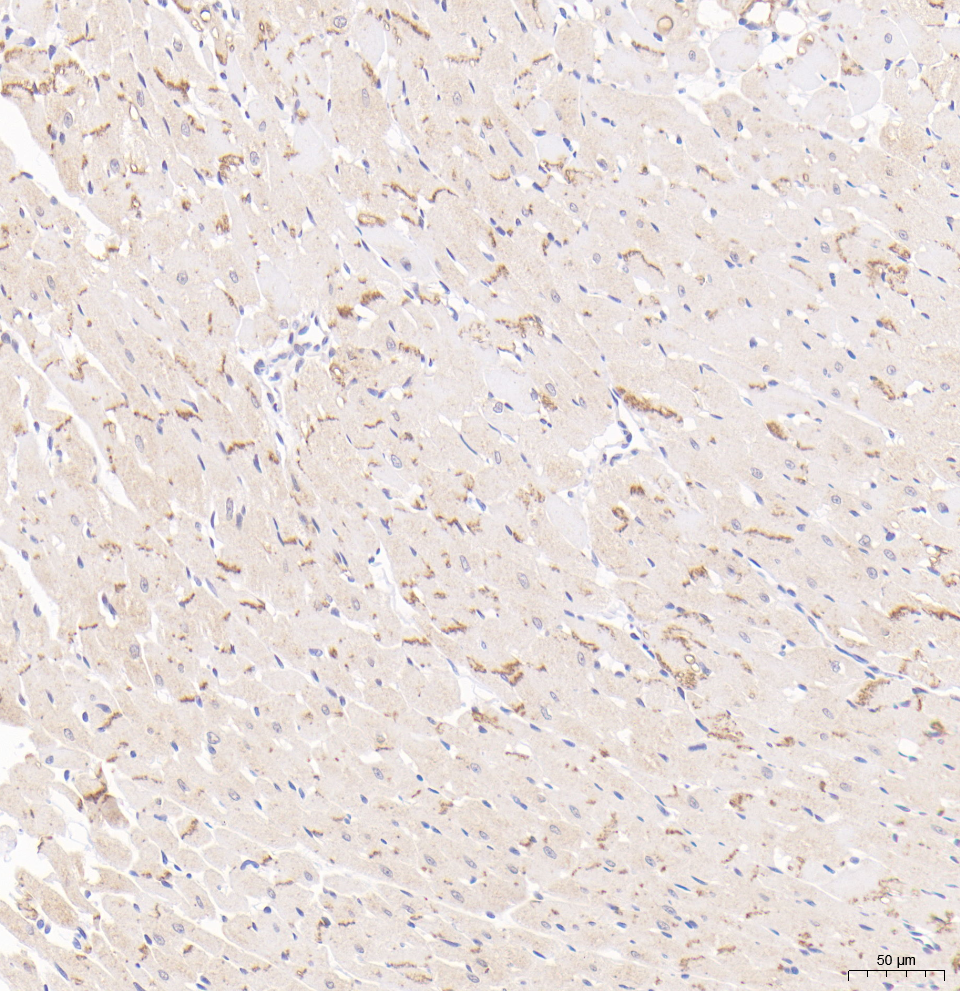

Supplement: Supplemental Information 24 [file peerj-13-19276-s024.zip › 2.immunohistochemical-I/R/I/R7-2.tif]

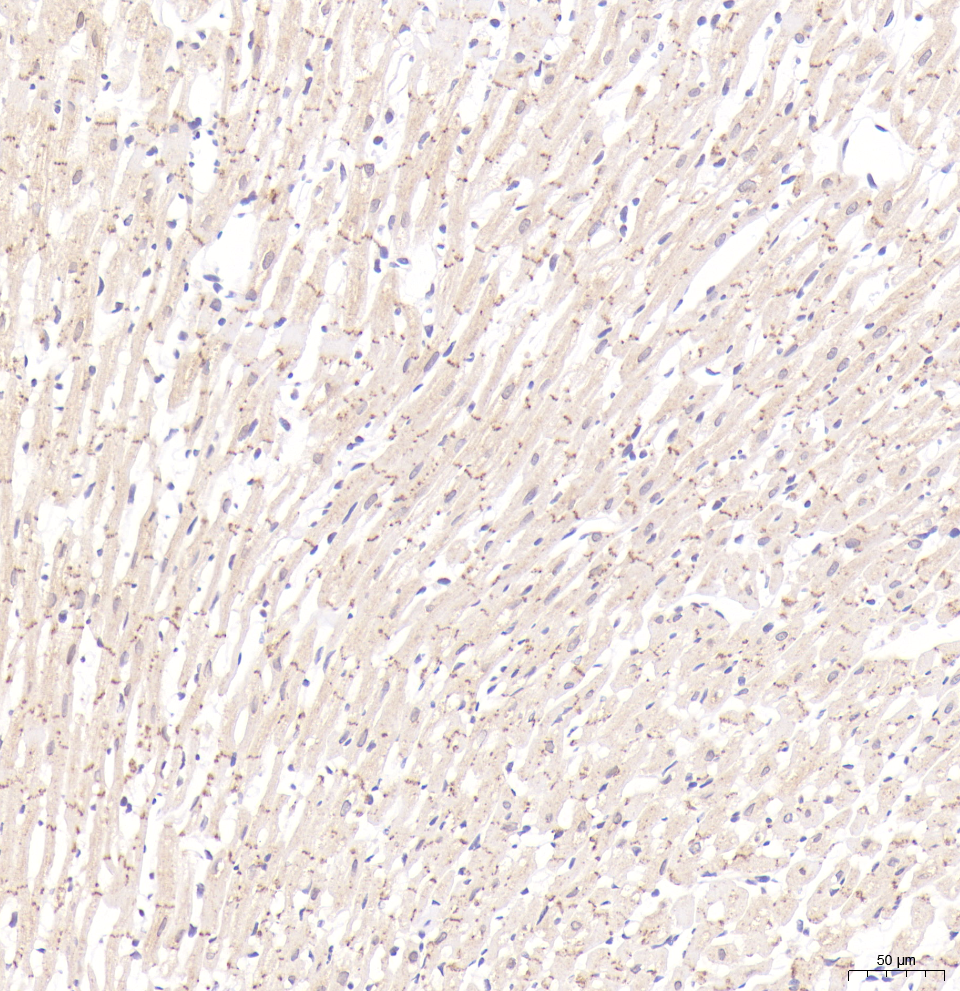

Supplement: Supplemental Information 25 [file peerj-13-19276-s025.zip › 1.immunohistochemical-AAV9-CON/AAV9-CON1-1.tif]

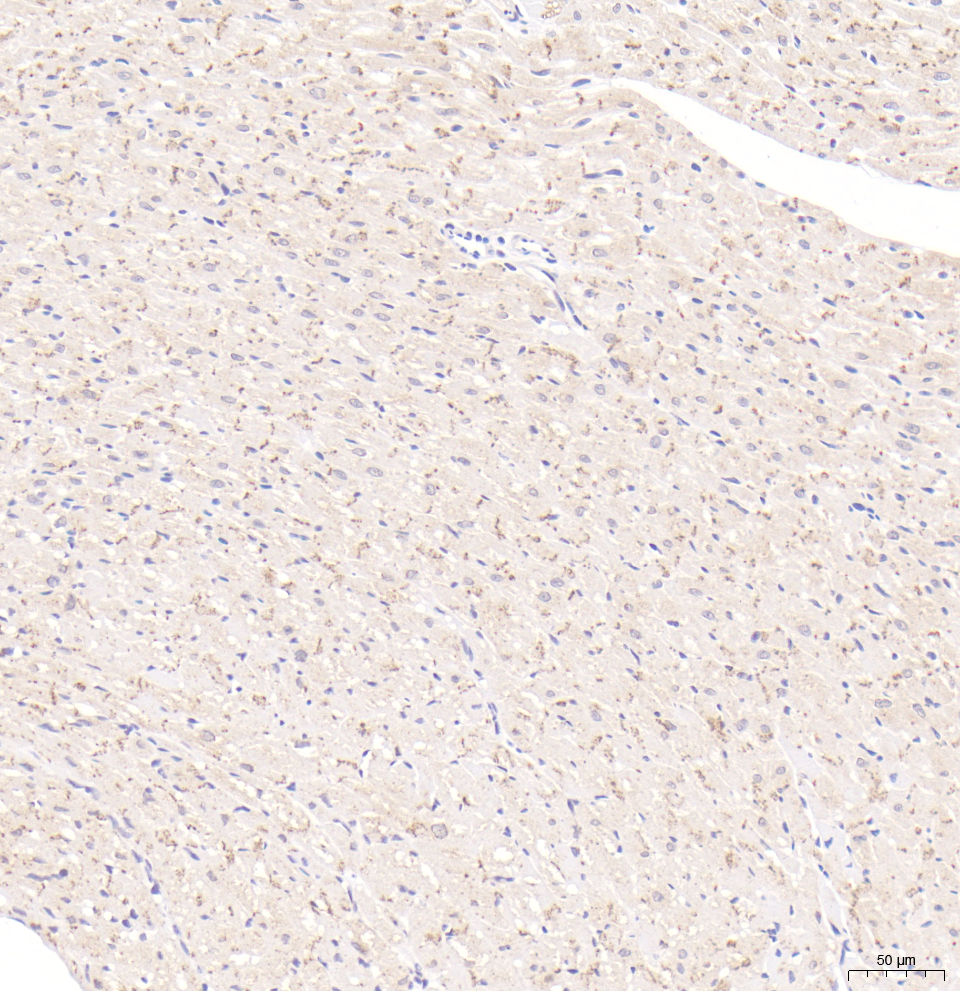

Supplement: Supplemental Information 25 [file peerj-13-19276-s025.zip › 1.immunohistochemical-AAV9-CON/AAV9-CON1-2.tif]

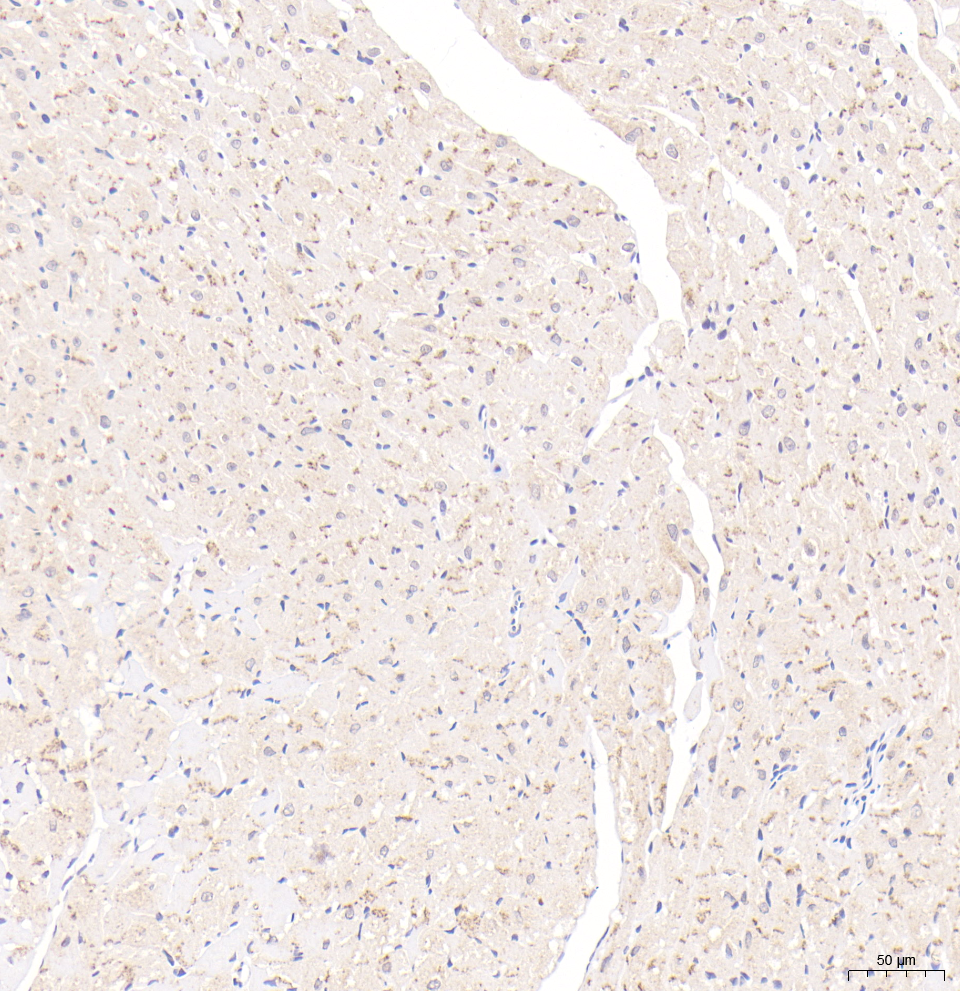

Supplement: Supplemental Information 25 [file peerj-13-19276-s025.zip › 1.immunohistochemical-AAV9-CON/AAV9-CON1-3.tif]

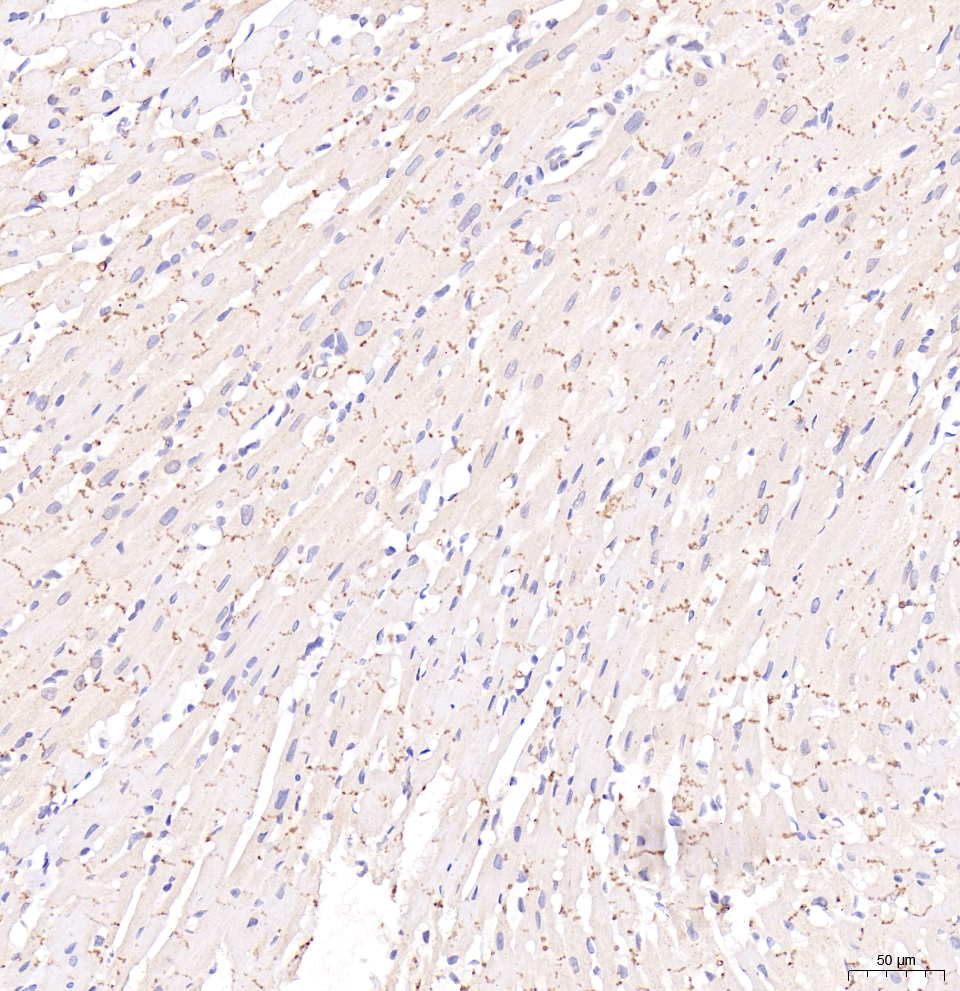

Supplement: Supplemental Information 25 [file peerj-13-19276-s025.zip › 1.immunohistochemical-AAV9-CON/AAV9-CON3-1.tif]

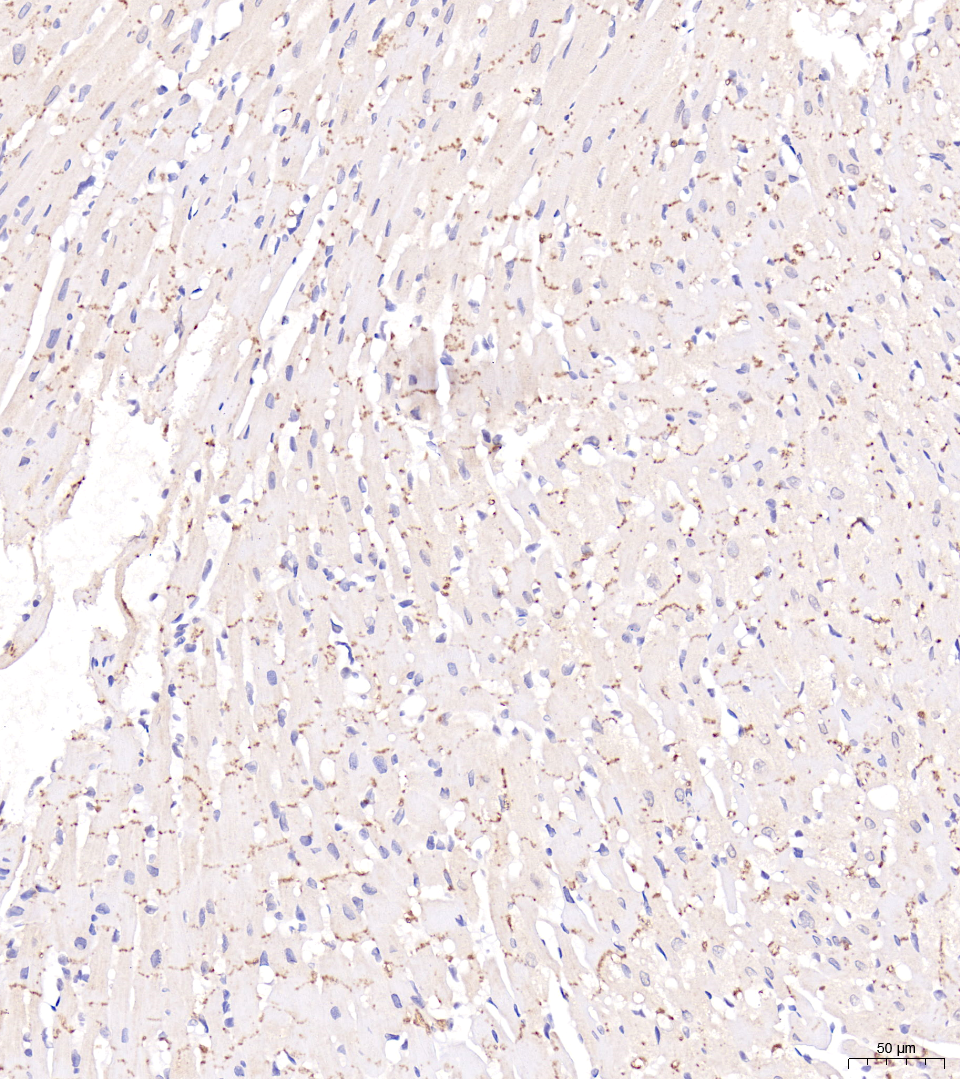

Supplement: Supplemental Information 25 [file peerj-13-19276-s025.zip › 1.immunohistochemical-AAV9-CON/AAV9-CON3-2.tif]

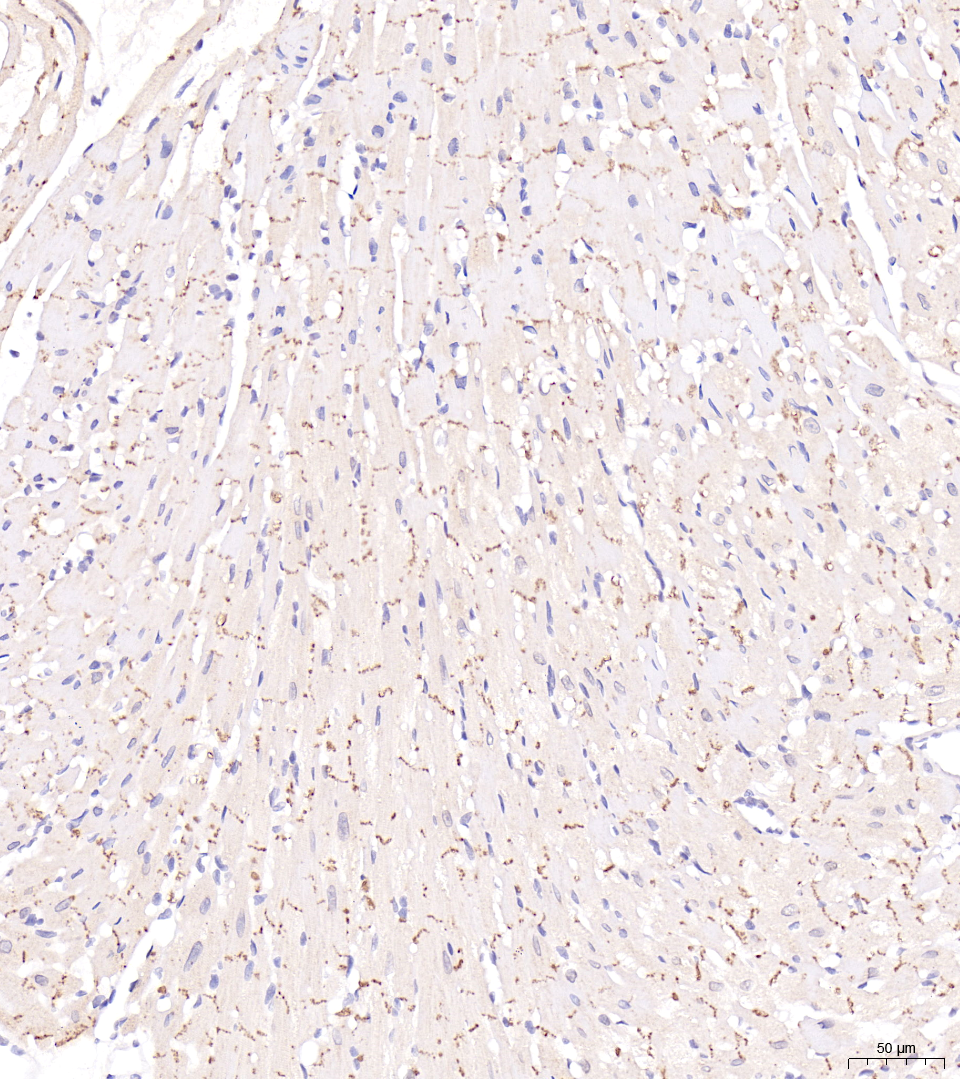

Supplement: Supplemental Information 25 [file peerj-13-19276-s025.zip › 1.immunohistochemical-AAV9-CON/AAV9-CON3-3.tif]

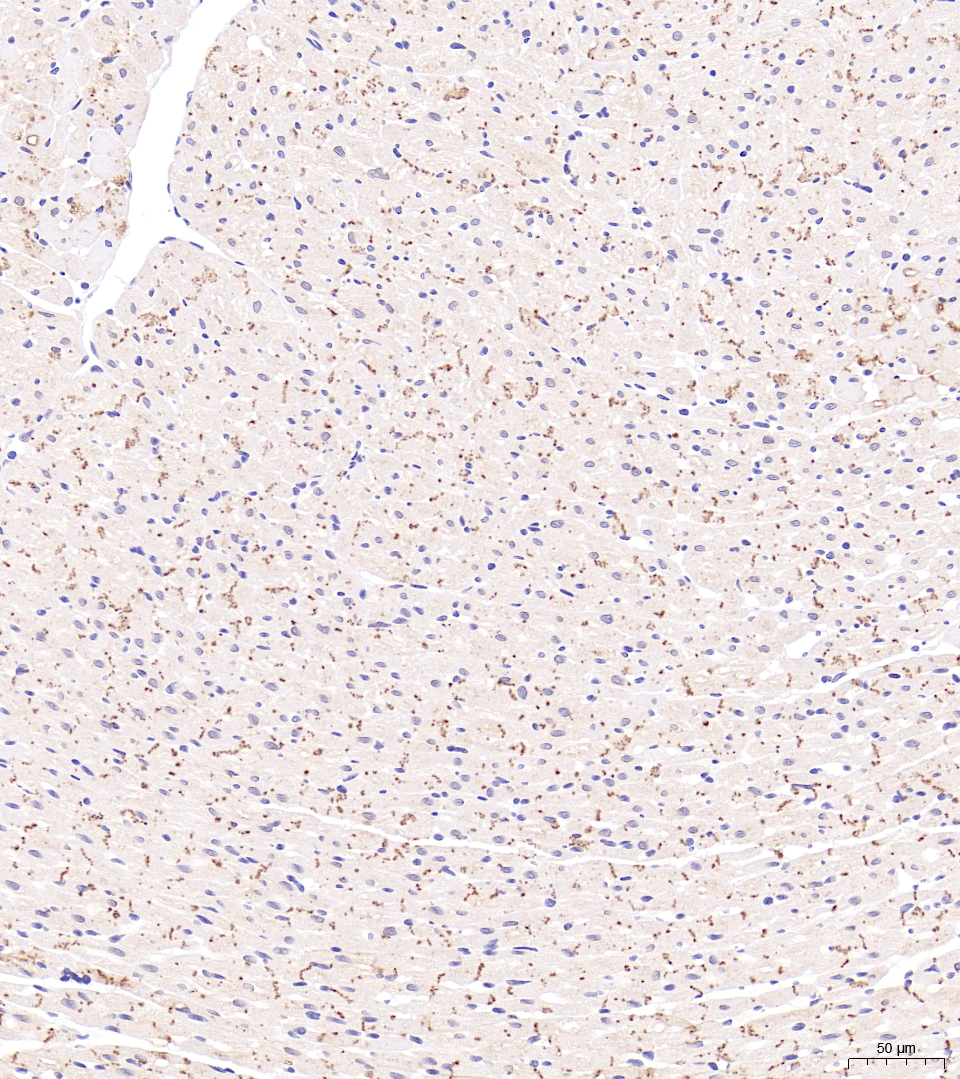

Supplement: Supplemental Information 25 [file peerj-13-19276-s025.zip › 1.immunohistochemical-AAV9-CON/AAV9-CON4-1.tif]

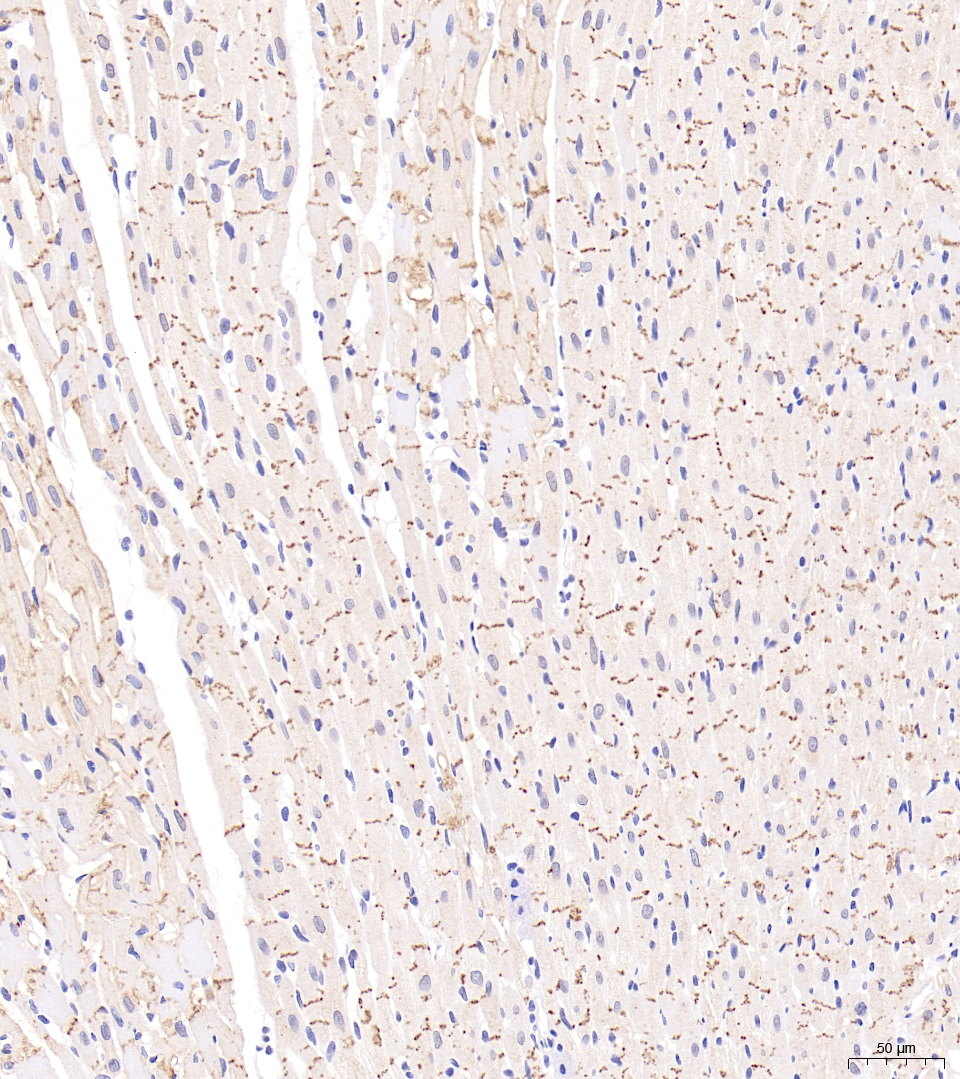

Supplement: Supplemental Information 26 [file peerj-13-19276-s026.zip › 2.immunohistochemical-AAV9-CON/AAV9-CON4-2.tif]

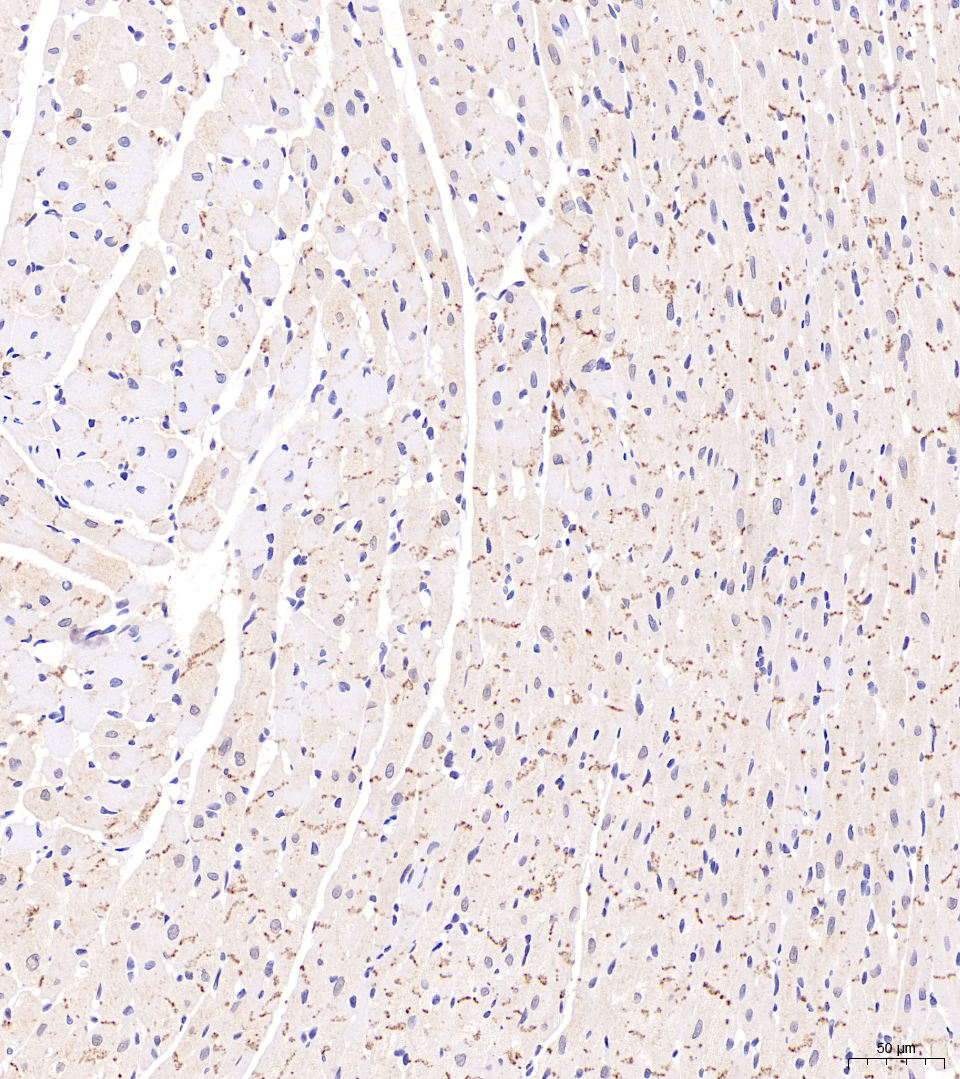

Supplement: Supplemental Information 26 [file peerj-13-19276-s026.zip › 2.immunohistochemical-AAV9-CON/AAV9-CON4-3.tif]

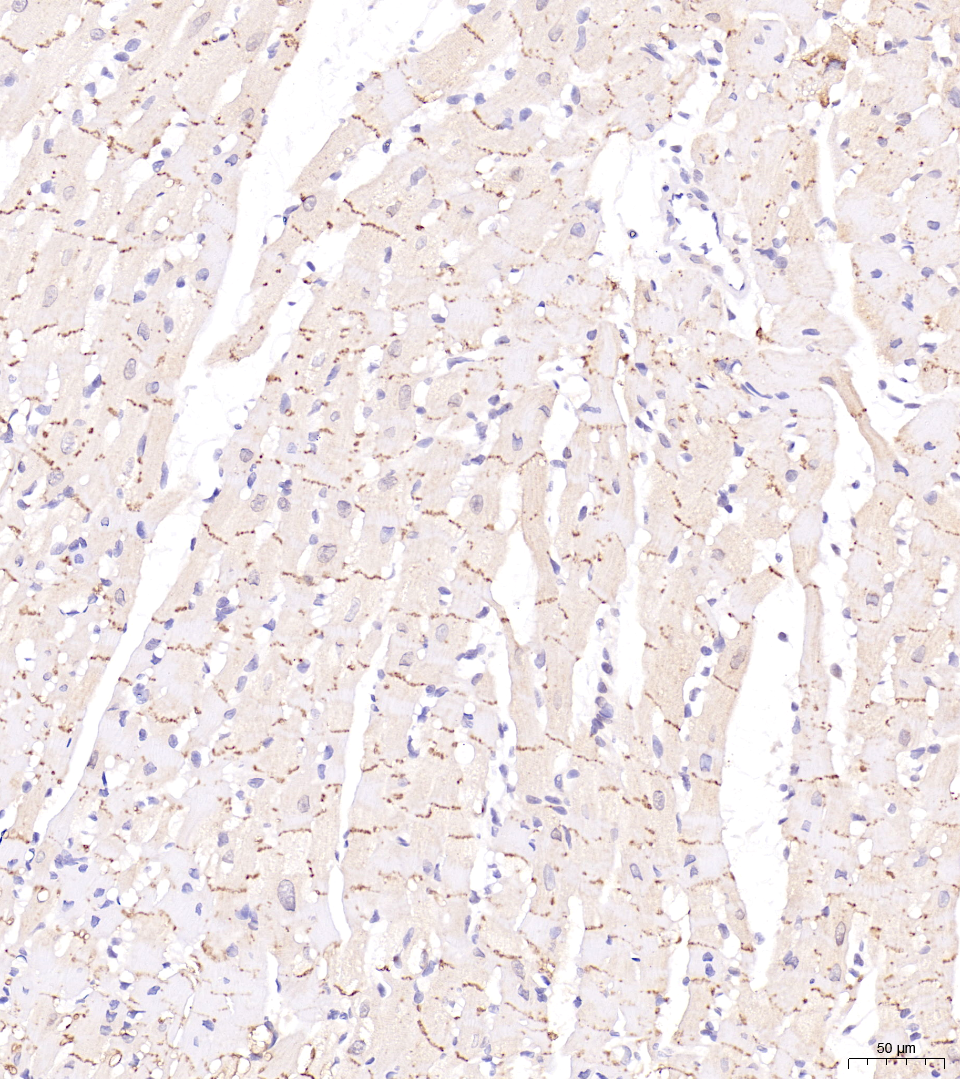

Supplement: Supplemental Information 26 [file peerj-13-19276-s026.zip › 2.immunohistochemical-AAV9-CON/AAV9-CON5-1.tif]

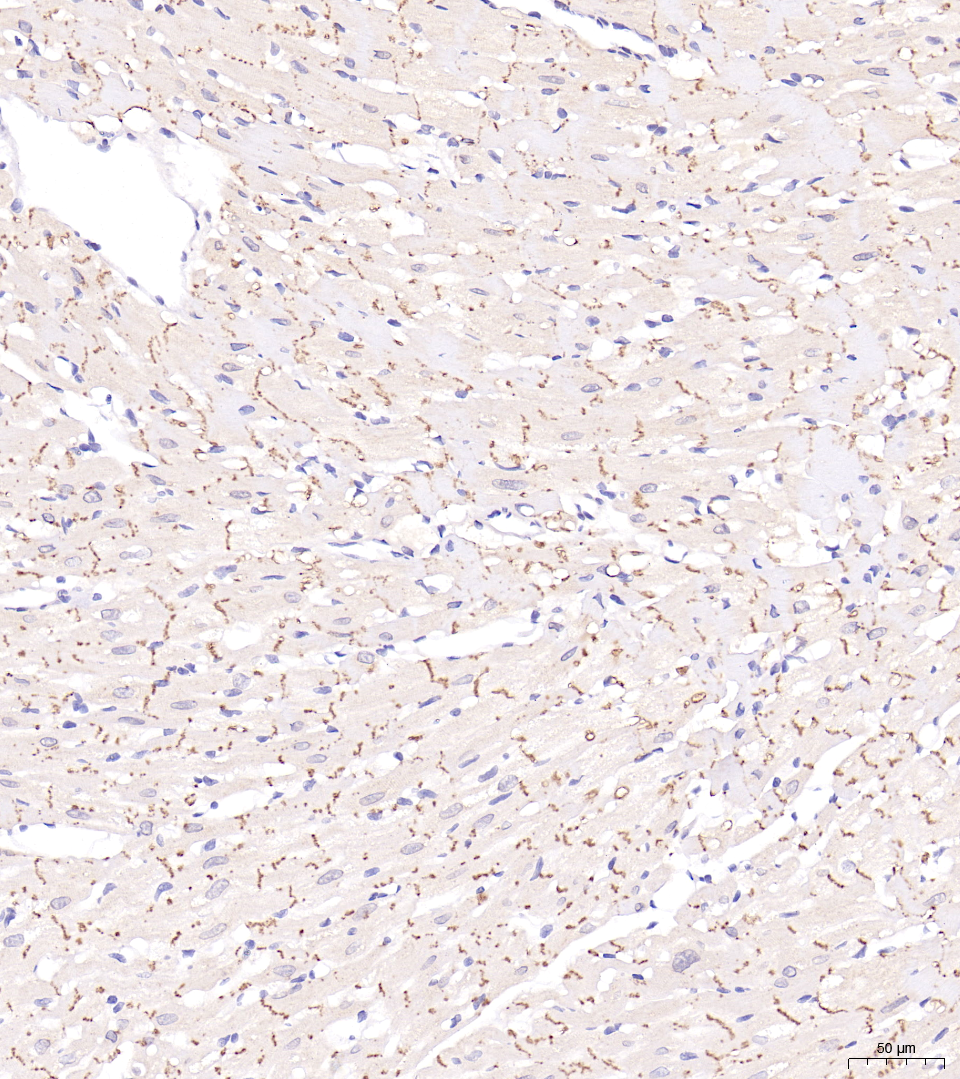

Supplement: Supplemental Information 26 [file peerj-13-19276-s026.zip › 2.immunohistochemical-AAV9-CON/AAV9-CON5-2.tif]

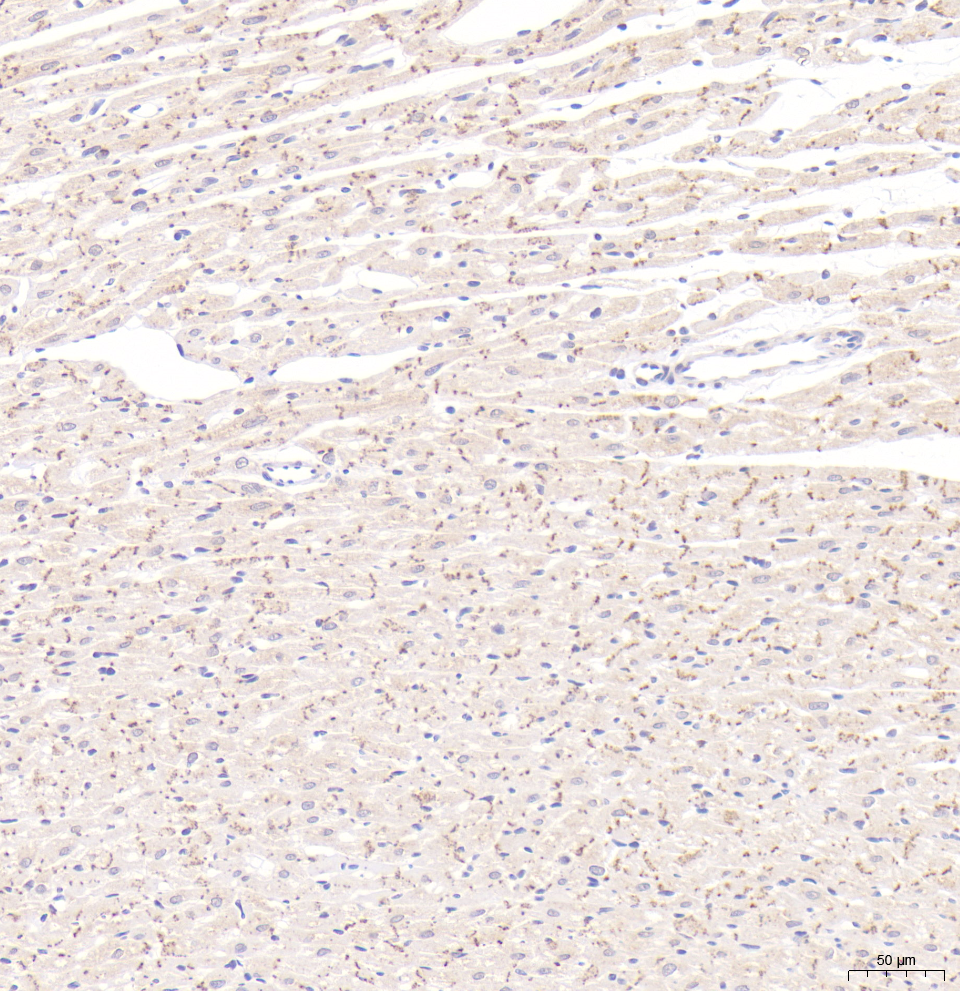

Supplement: Supplemental Information 26 [file peerj-13-19276-s026.zip › 2.immunohistochemical-AAV9-CON/AAV9-CON6-1.tif]

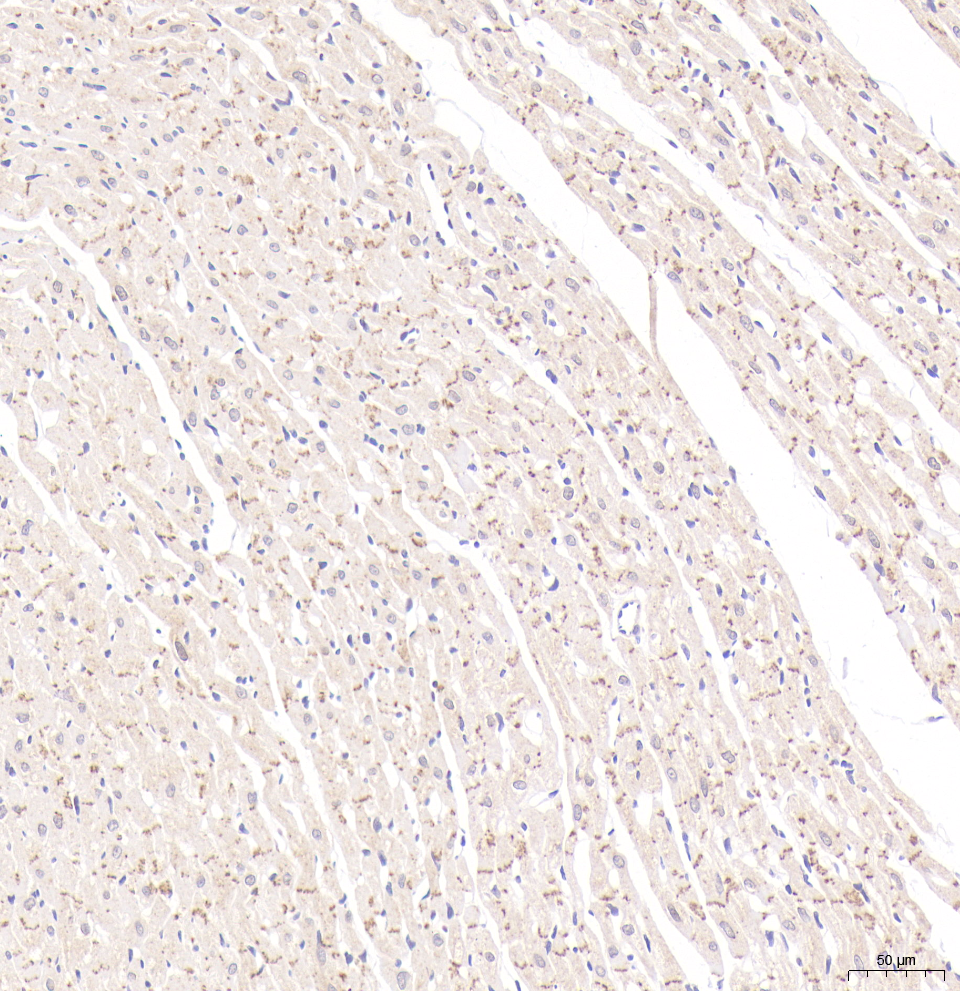

Supplement: Supplemental Information 26 [file peerj-13-19276-s026.zip › 2.immunohistochemical-AAV9-CON/AAV9-CON6-2.tif]

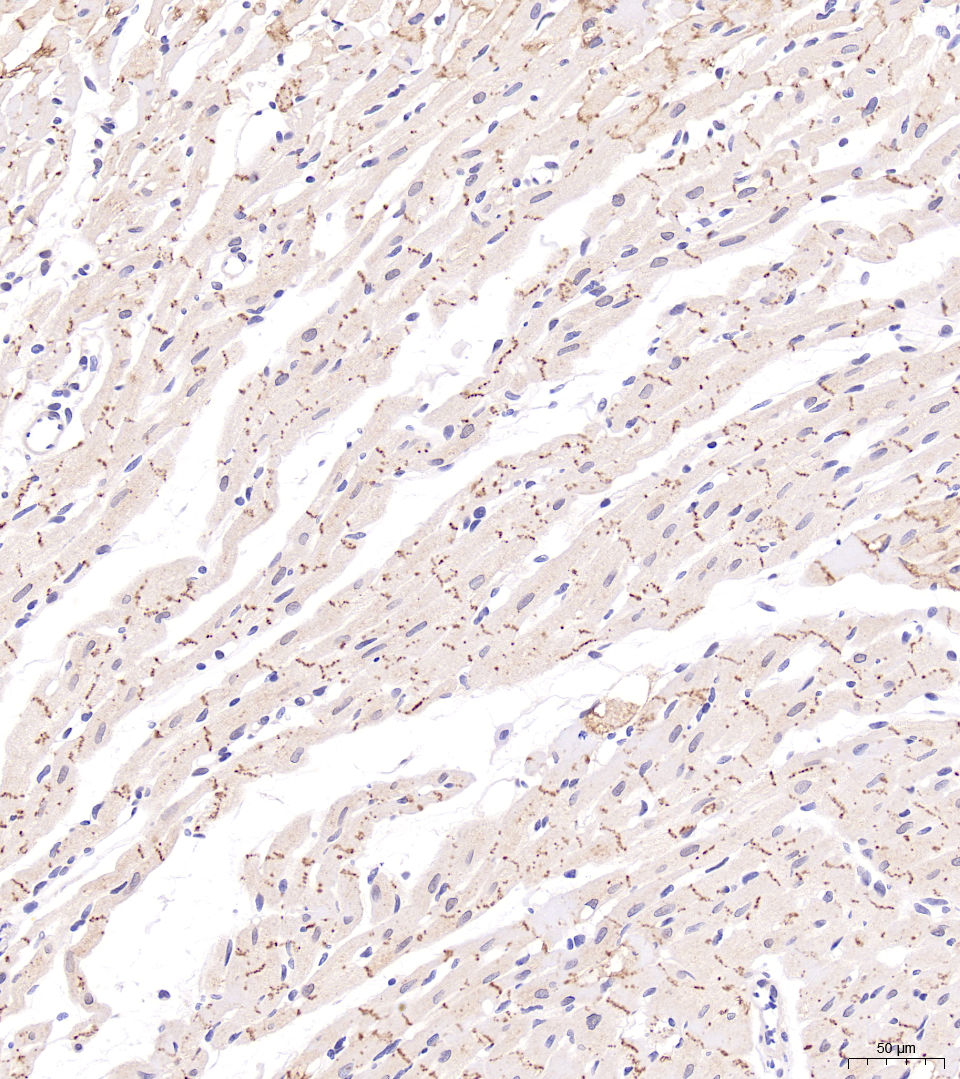

Supplement: Supplemental Information 26 [file peerj-13-19276-s026.zip › 2.immunohistochemical-AAV9-CON/AAV9-CON7-1.tif]

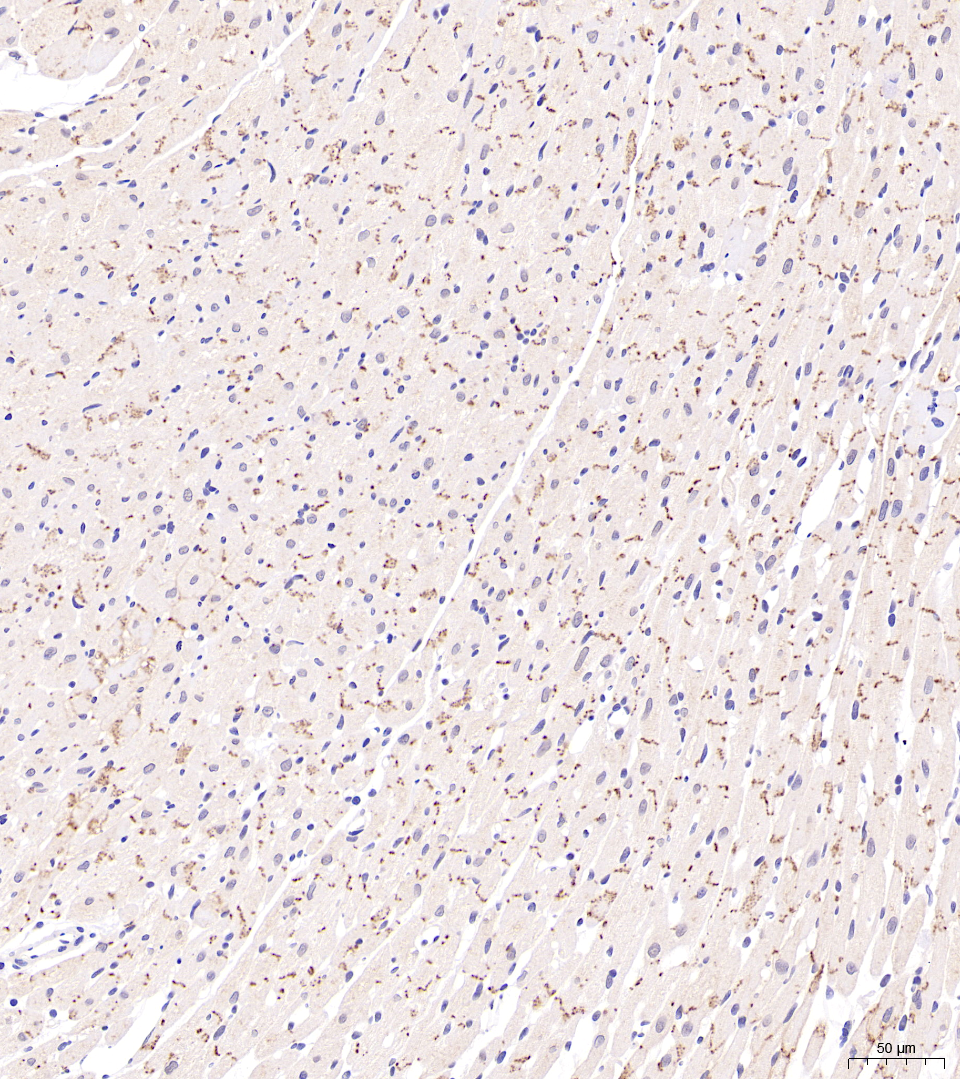

Supplement: Supplemental Information 26 [file peerj-13-19276-s026.zip › 2.immunohistochemical-AAV9-CON/AAV9-CON7-2.tif]

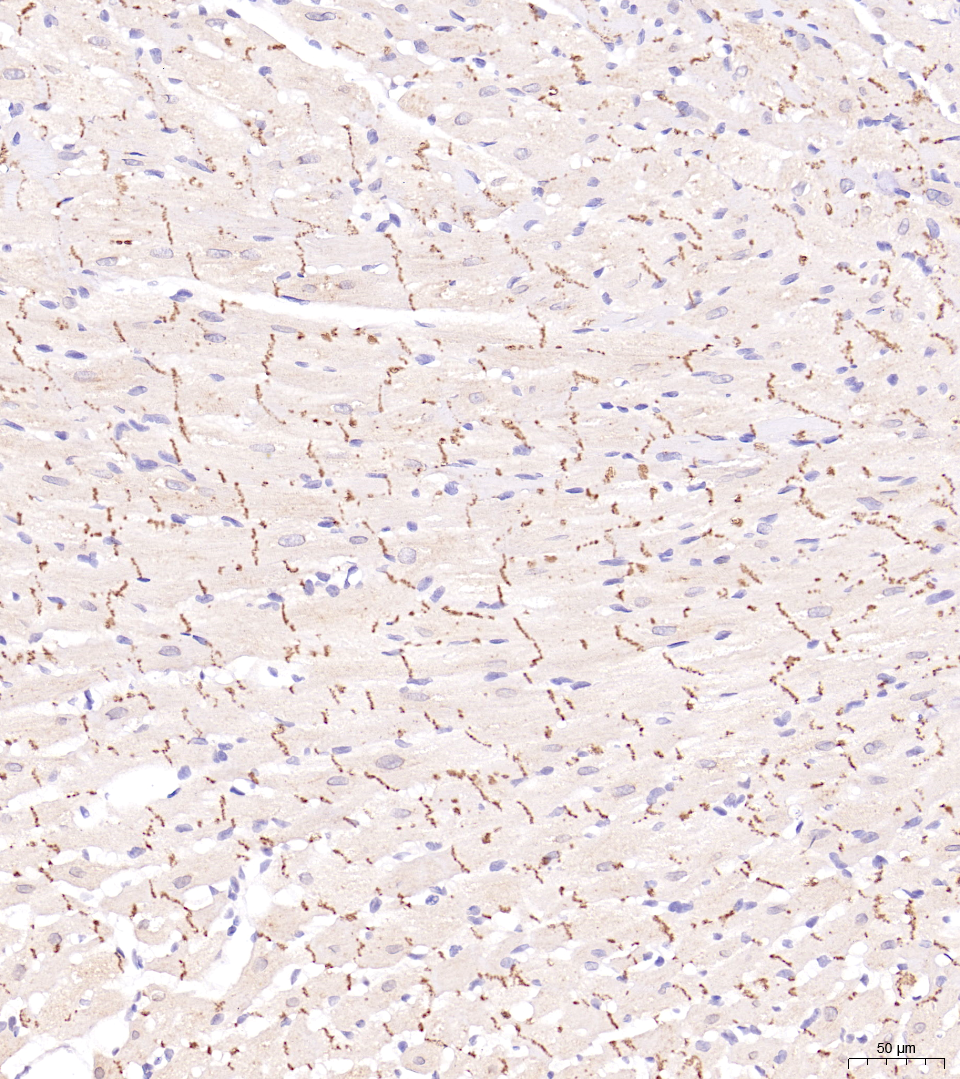

Supplement: Supplemental Information 27 [file peerj-13-19276-s027.zip › 1.immunohistochemical-AAV9-EB1/AAV9-EB1 1-1.tif]

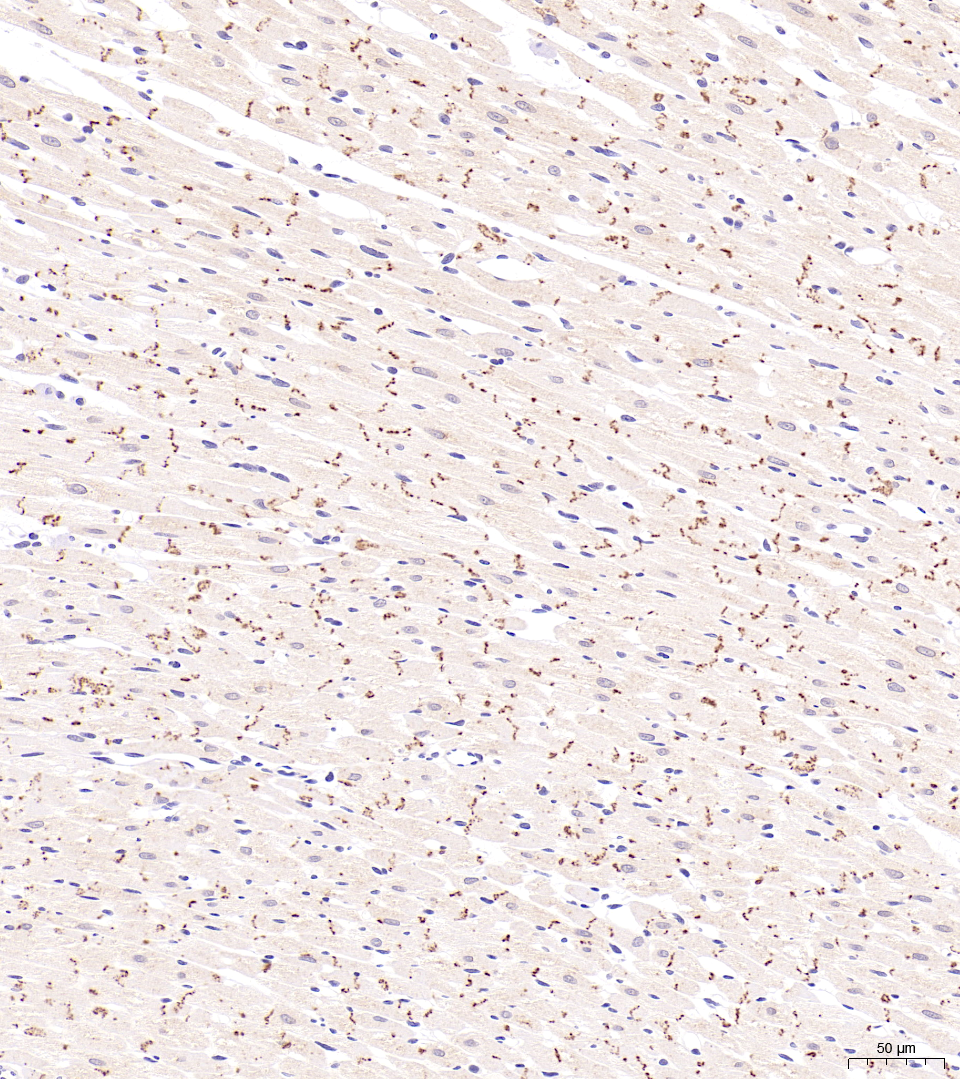

Supplement: Supplemental Information 27 [file peerj-13-19276-s027.zip › 1.immunohistochemical-AAV9-EB1/AAV9-EB1 1-2.tif]

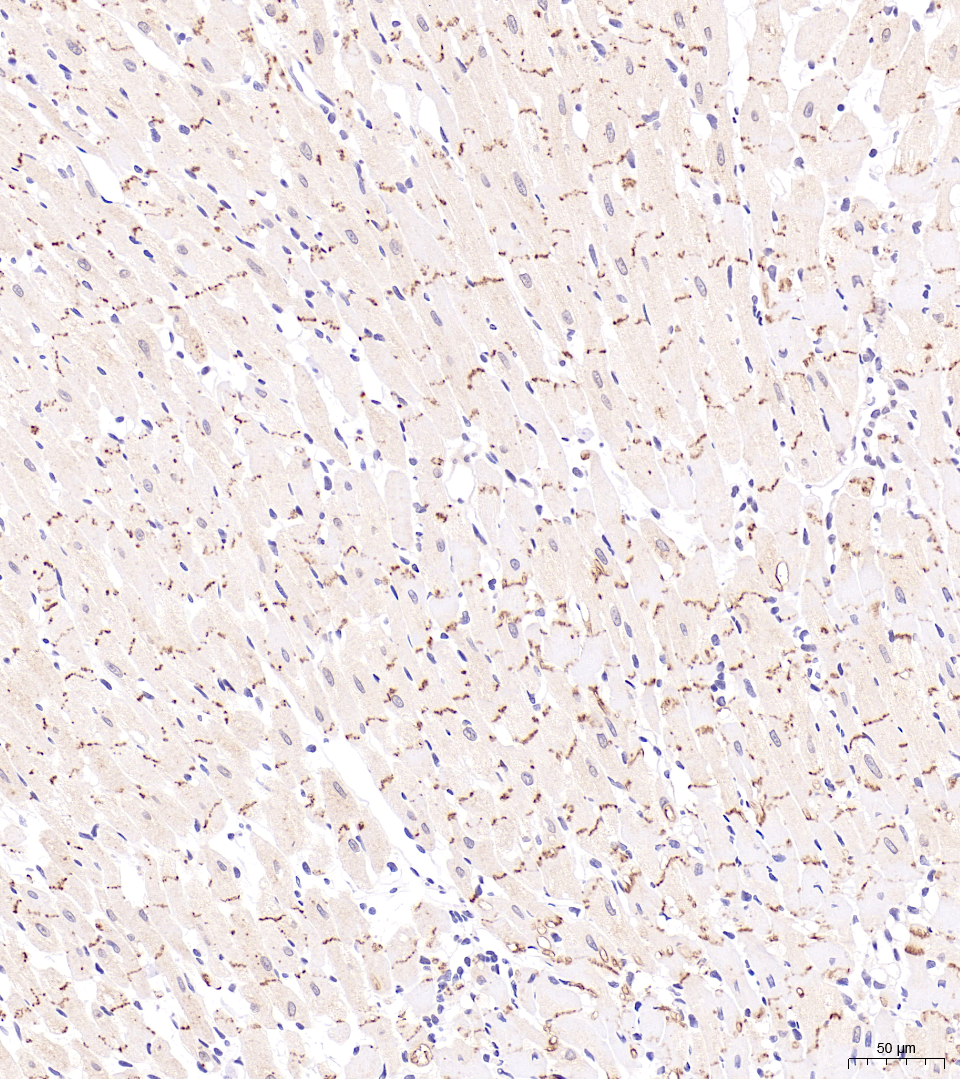

Supplement: Supplemental Information 27 [file peerj-13-19276-s027.zip › 1.immunohistochemical-AAV9-EB1/AAV9-EB1 1-3.tif]

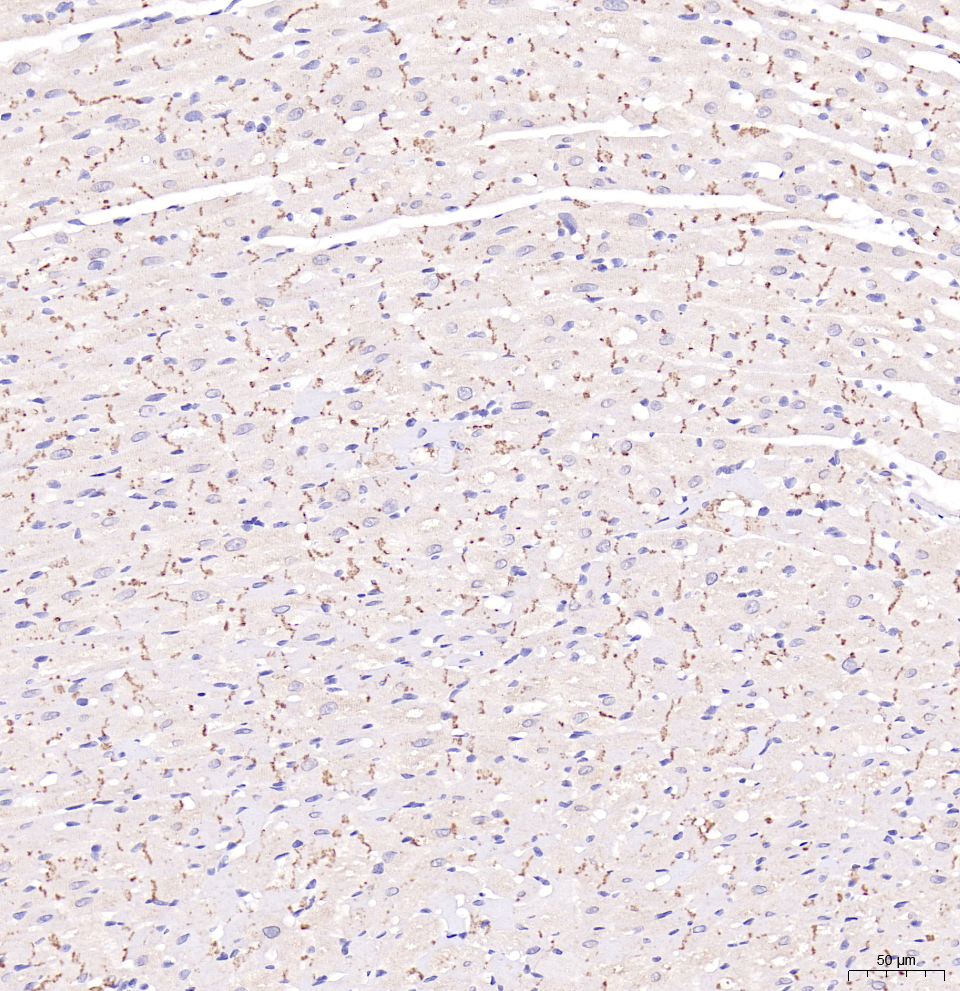

Supplement: Supplemental Information 27 [file peerj-13-19276-s027.zip › 1.immunohistochemical-AAV9-EB1/AAV9-EB1 3-1.tif]

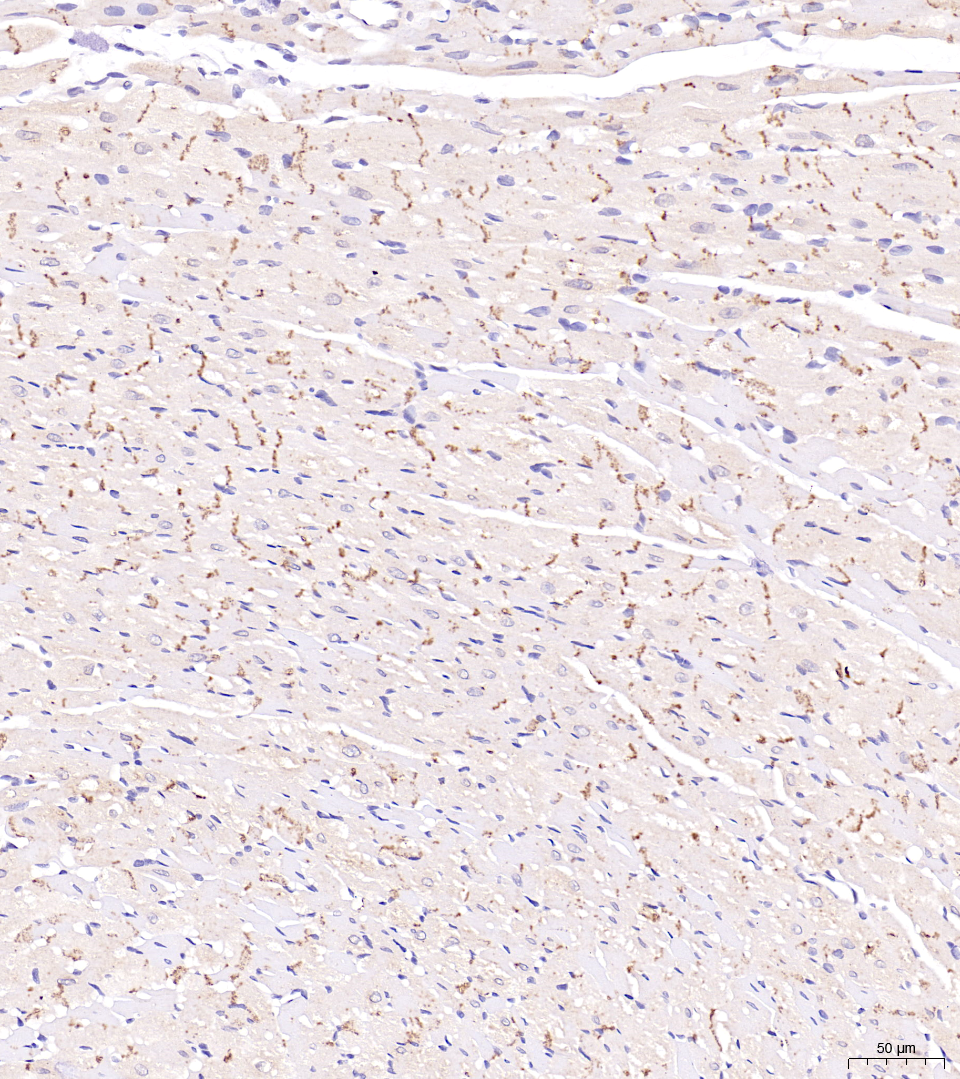

Supplement: Supplemental Information 27 [file peerj-13-19276-s027.zip › 1.immunohistochemical-AAV9-EB1/AAV9-EB1 3-2.tif]

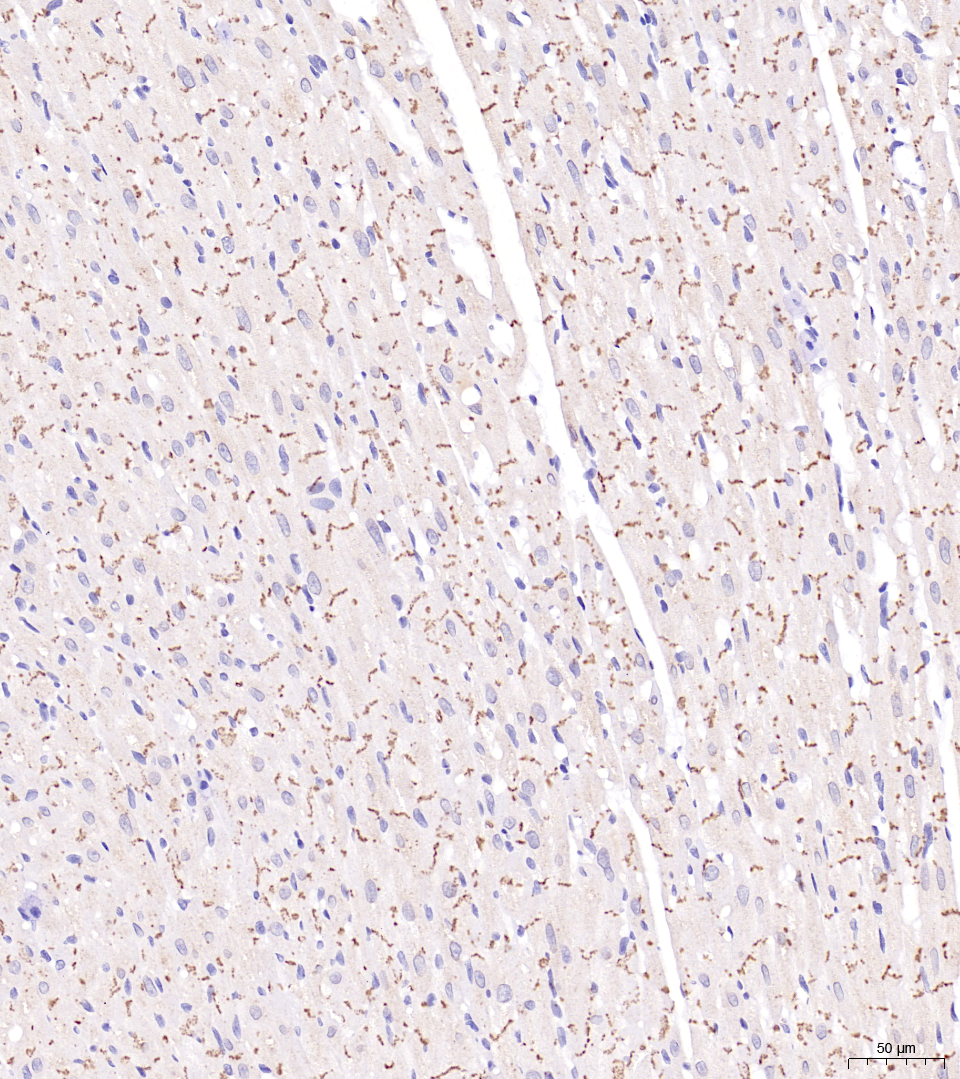

Supplement: Supplemental Information 27 [file peerj-13-19276-s027.zip › 1.immunohistochemical-AAV9-EB1/AAV9-EB1 3-3.tif]

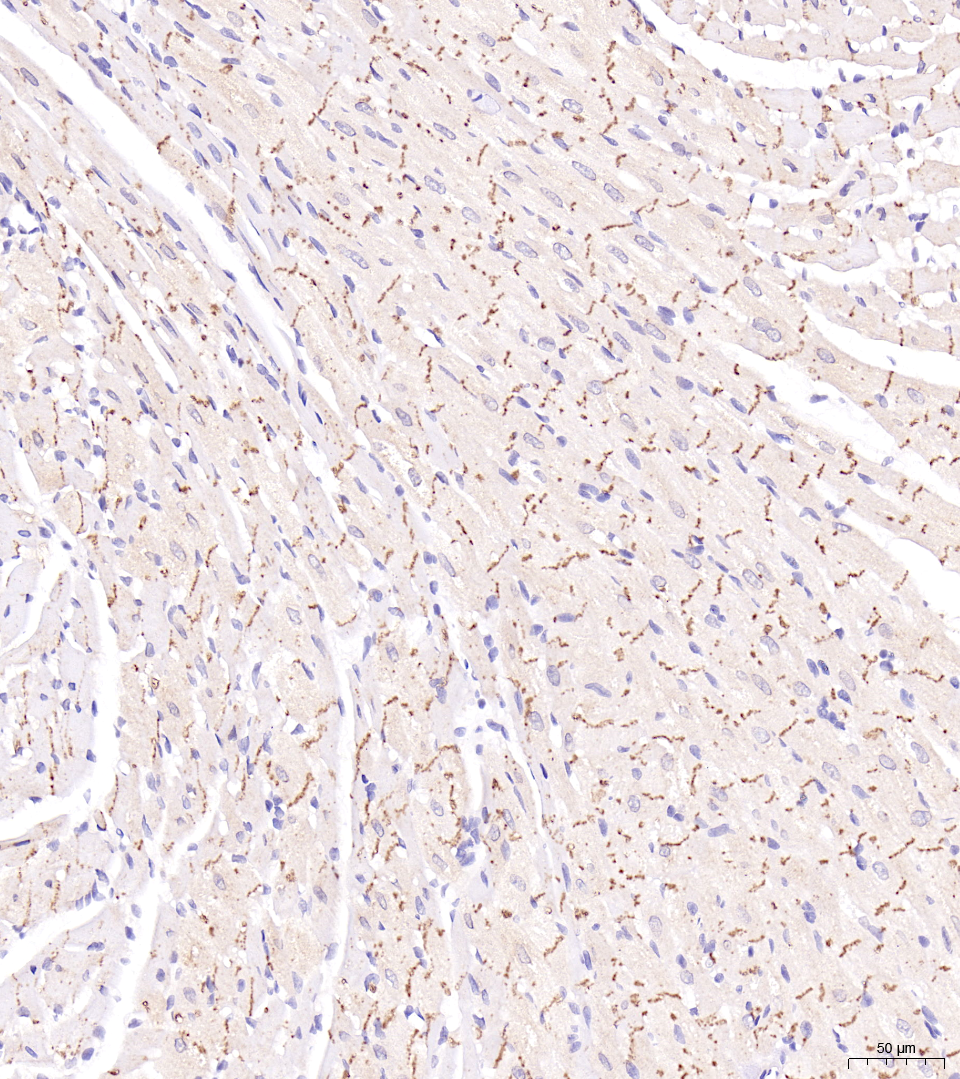

Supplement: Supplemental Information 27 [file peerj-13-19276-s027.zip › 1.immunohistochemical-AAV9-EB1/AAV9-EB1 4-1.tif]

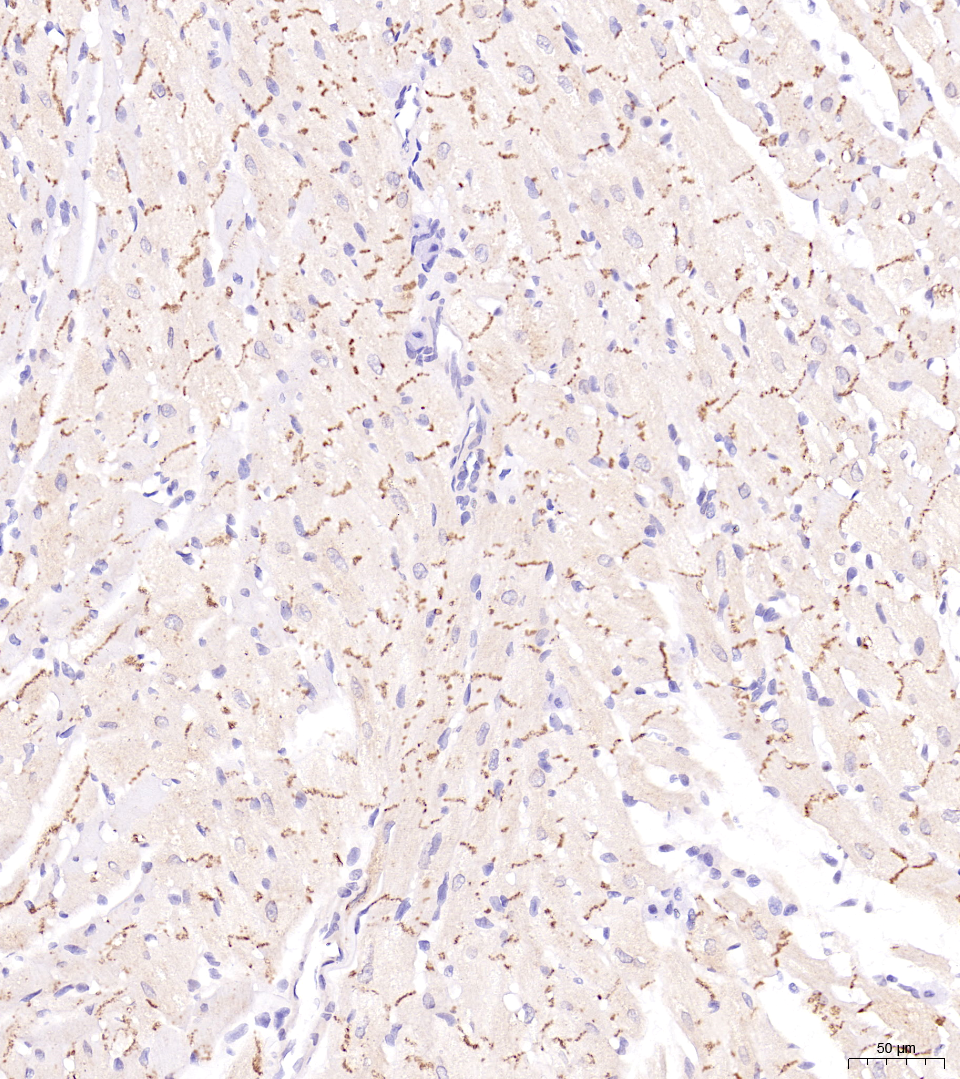

Supplement: Supplemental Information 27 [file peerj-13-19276-s027.zip › 1.immunohistochemical-AAV9-EB1/AAV9-EB1 4-2.tif]

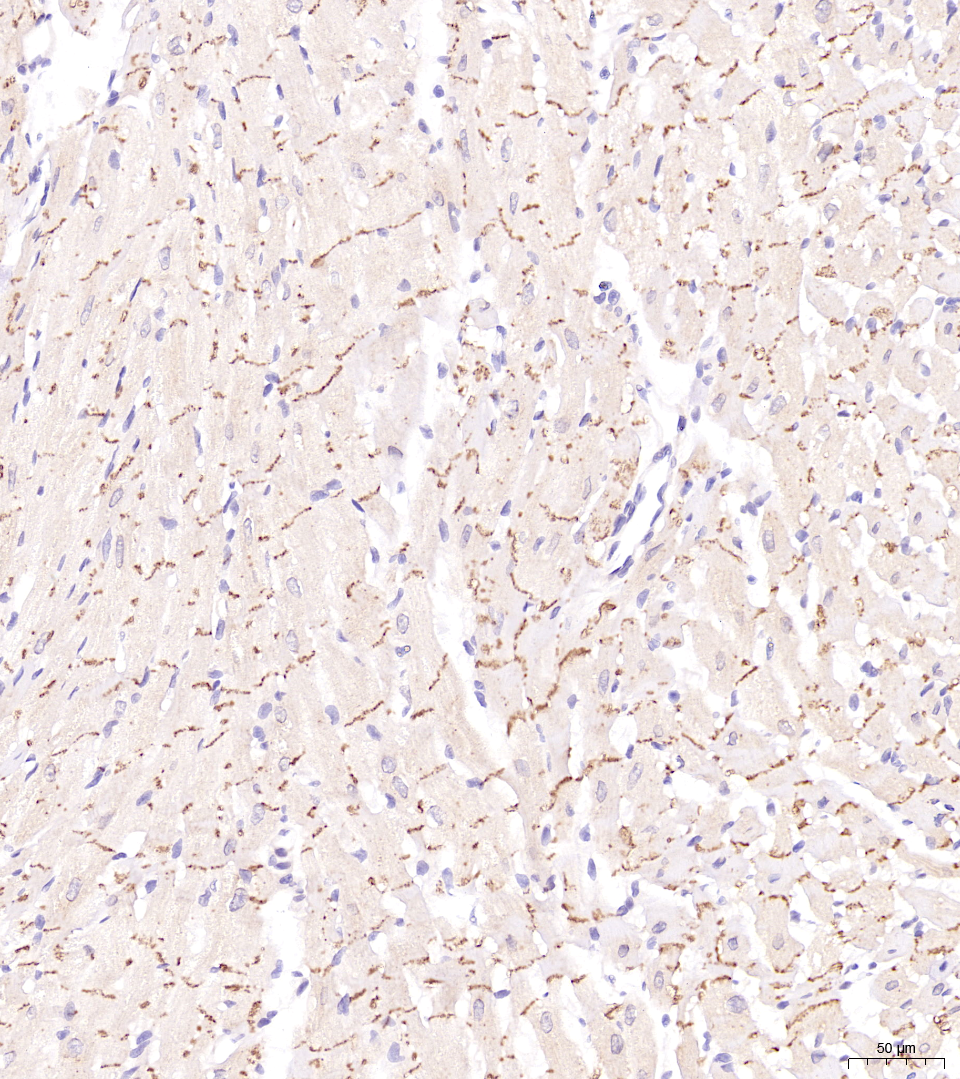

Supplement: Supplemental Information 28 [file peerj-13-19276-s028.zip › 2.immunohistochemical-AAV9-EB1/AAV9-EB1 4-3.tif]

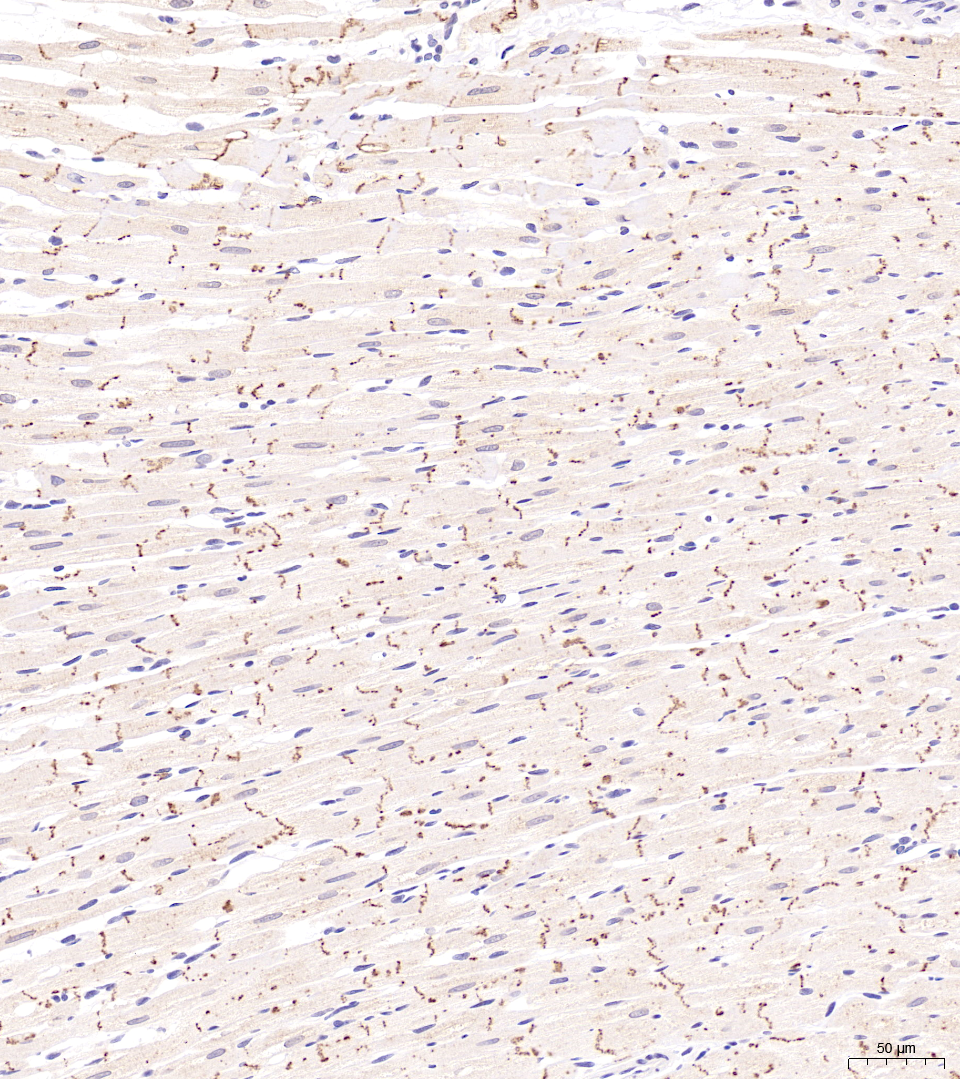

Supplement: Supplemental Information 28 [file peerj-13-19276-s028.zip › 2.immunohistochemical-AAV9-EB1/AAV9-EB1 5-1.tif]

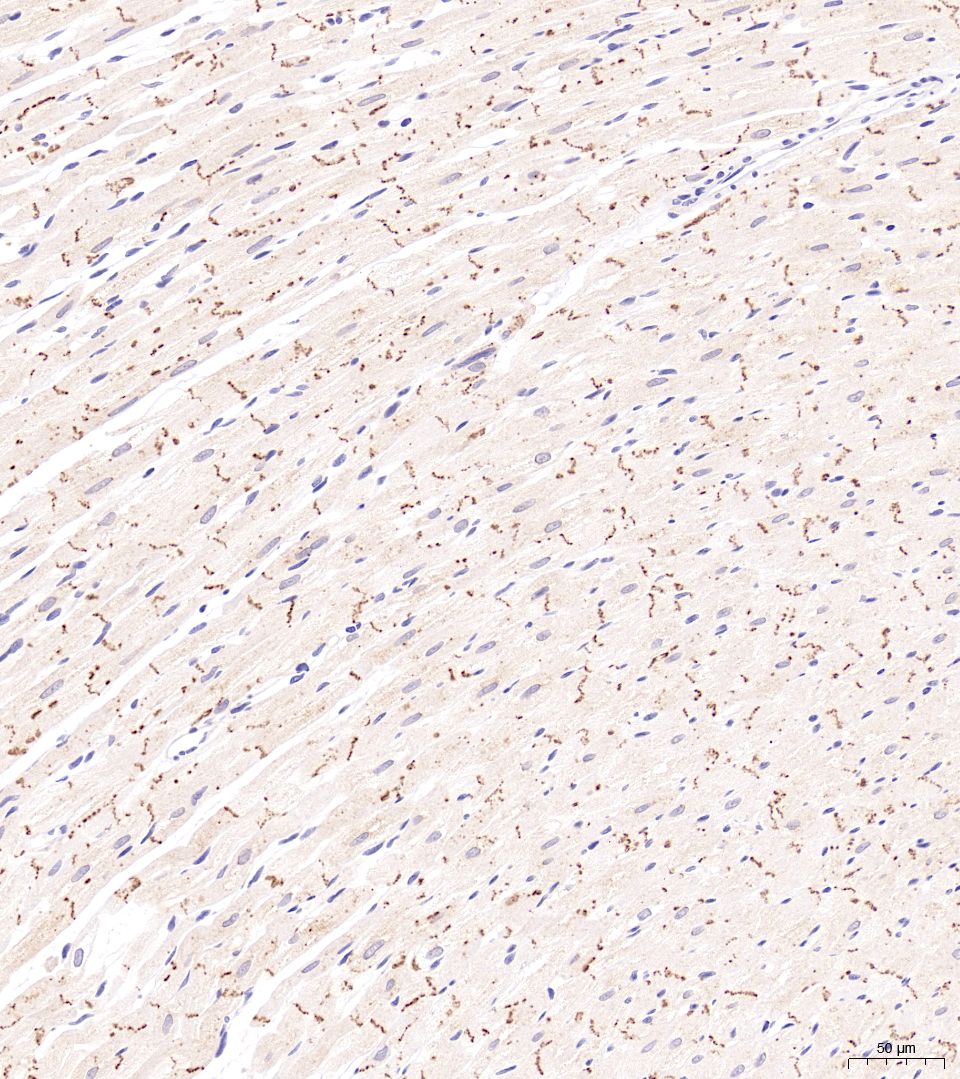

Supplement: Supplemental Information 28 [file peerj-13-19276-s028.zip › 2.immunohistochemical-AAV9-EB1/AAV9-EB1 5-2.tif]

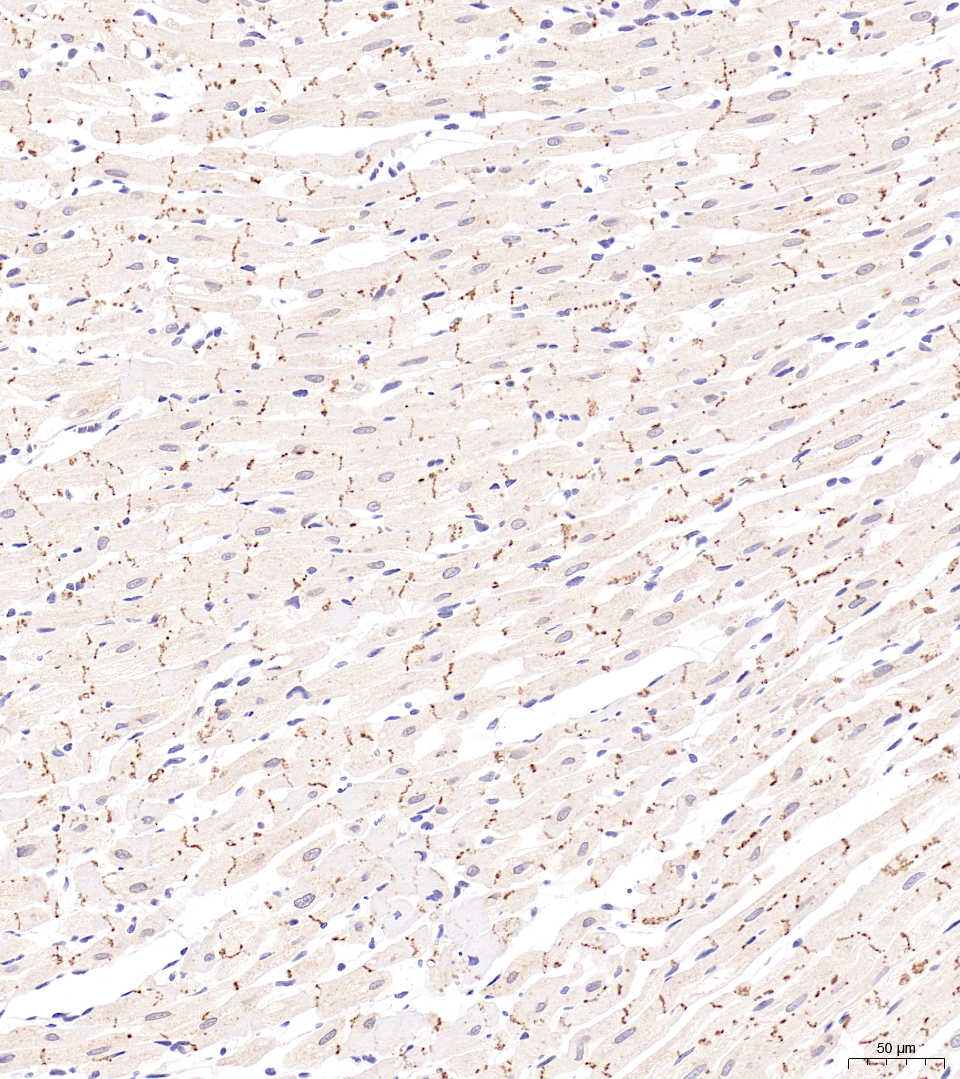

Supplement: Supplemental Information 28 [file peerj-13-19276-s028.zip › 2.immunohistochemical-AAV9-EB1/AAV9-EB1 5-3.tif]

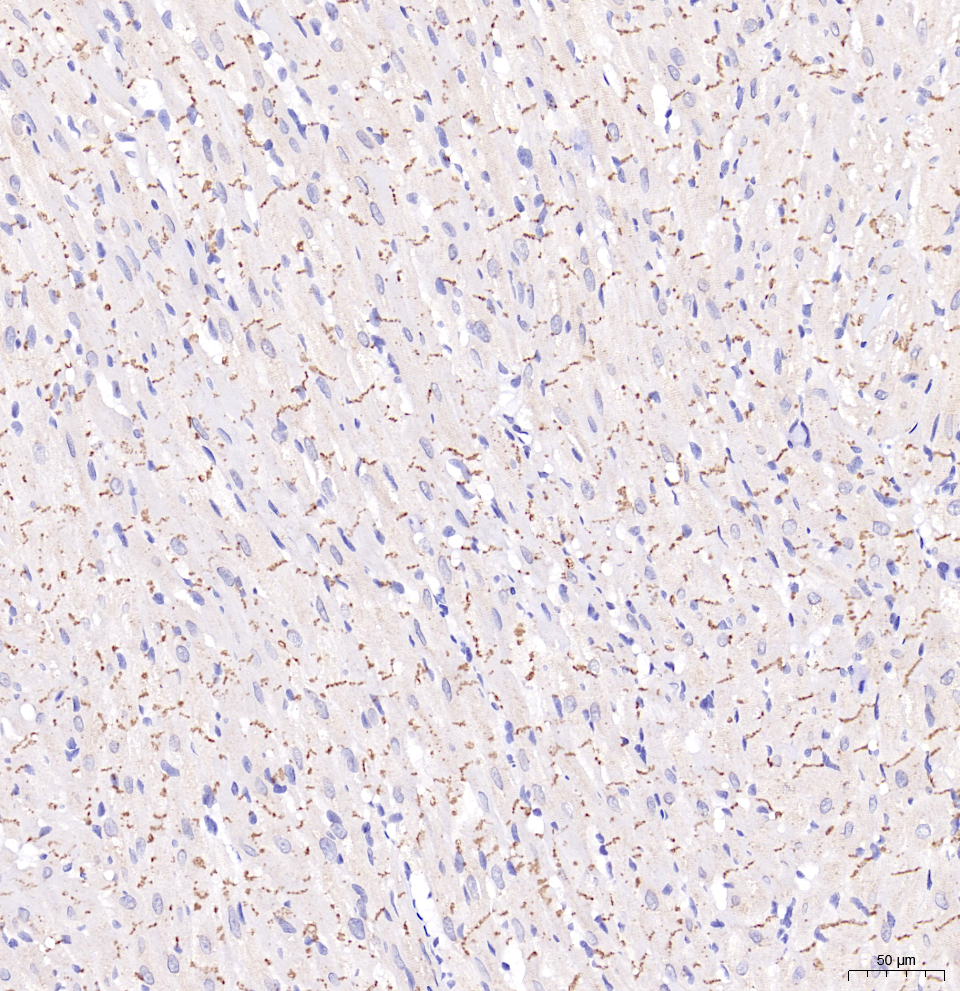

Supplement: Supplemental Information 28 [file peerj-13-19276-s028.zip › 2.immunohistochemical-AAV9-EB1/AAV9-EB1 6-1.tif]

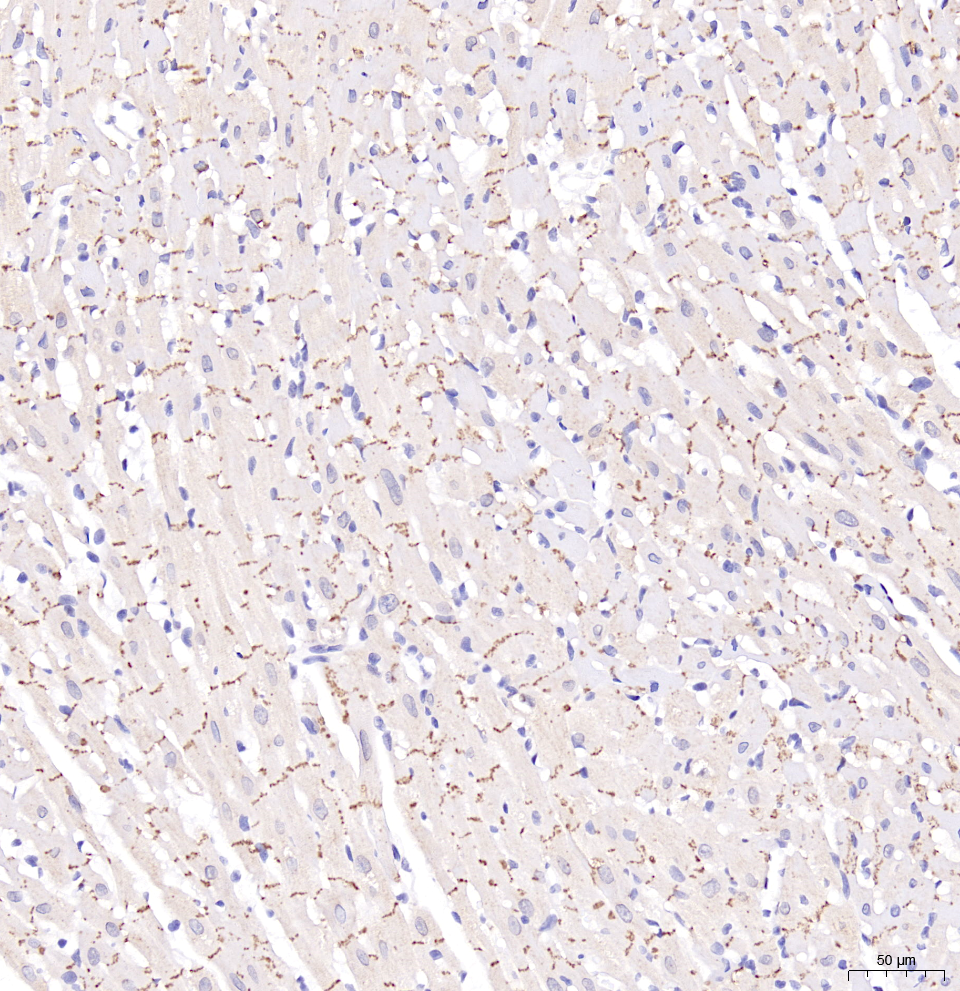

Supplement: Supplemental Information 28 [file peerj-13-19276-s028.zip › 2.immunohistochemical-AAV9-EB1/AAV9-EB1 6-2.tif]

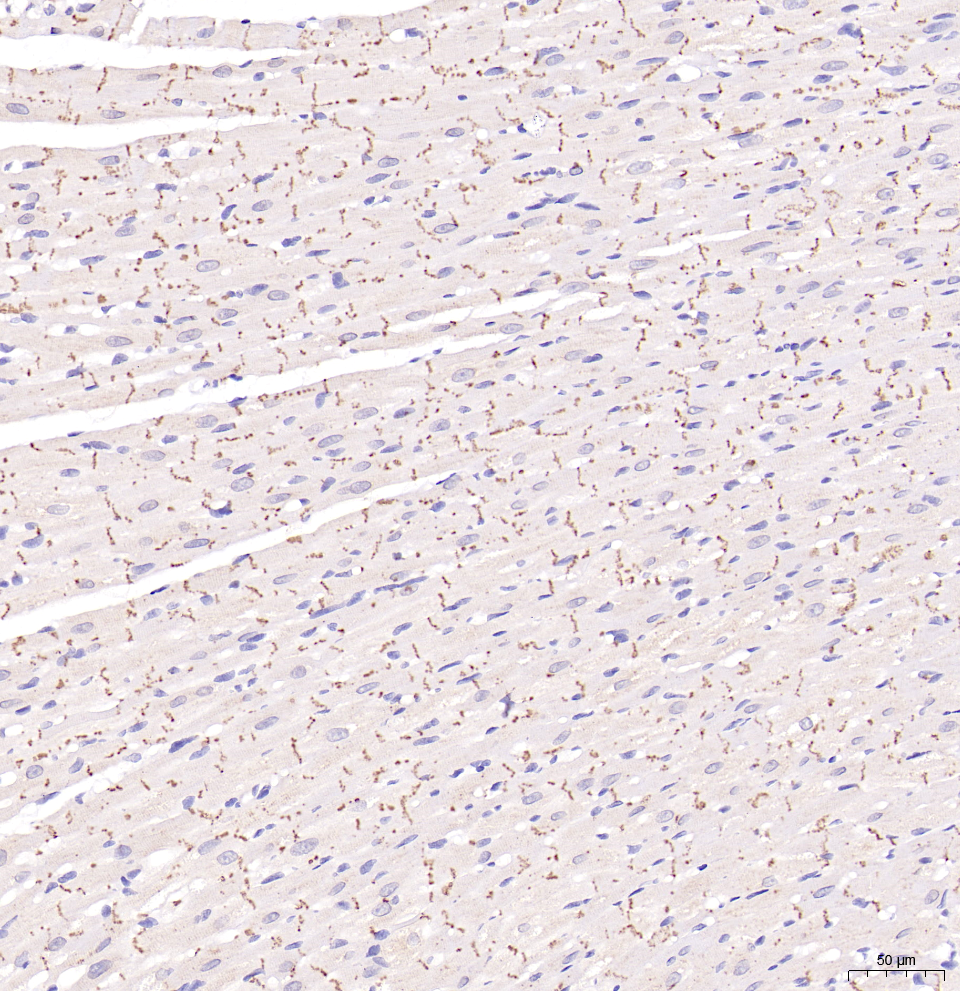

Supplement: Supplemental Information 28 [file peerj-13-19276-s028.zip › 2.immunohistochemical-AAV9-EB1/AAV9-EB1 6-3.tif]
